# Supplementary figures and images for: Feasibility study for supporting medication adherence for adults with cystic fibrosis: mixed-methods process evaluation
Source: BMJ Open. 2020 Oct 27;10(10):e039089. doi: 10.1136/bmjopen-2020-039089 (PMC7592300; doi:10.1136/bmjopen-2020-039089)

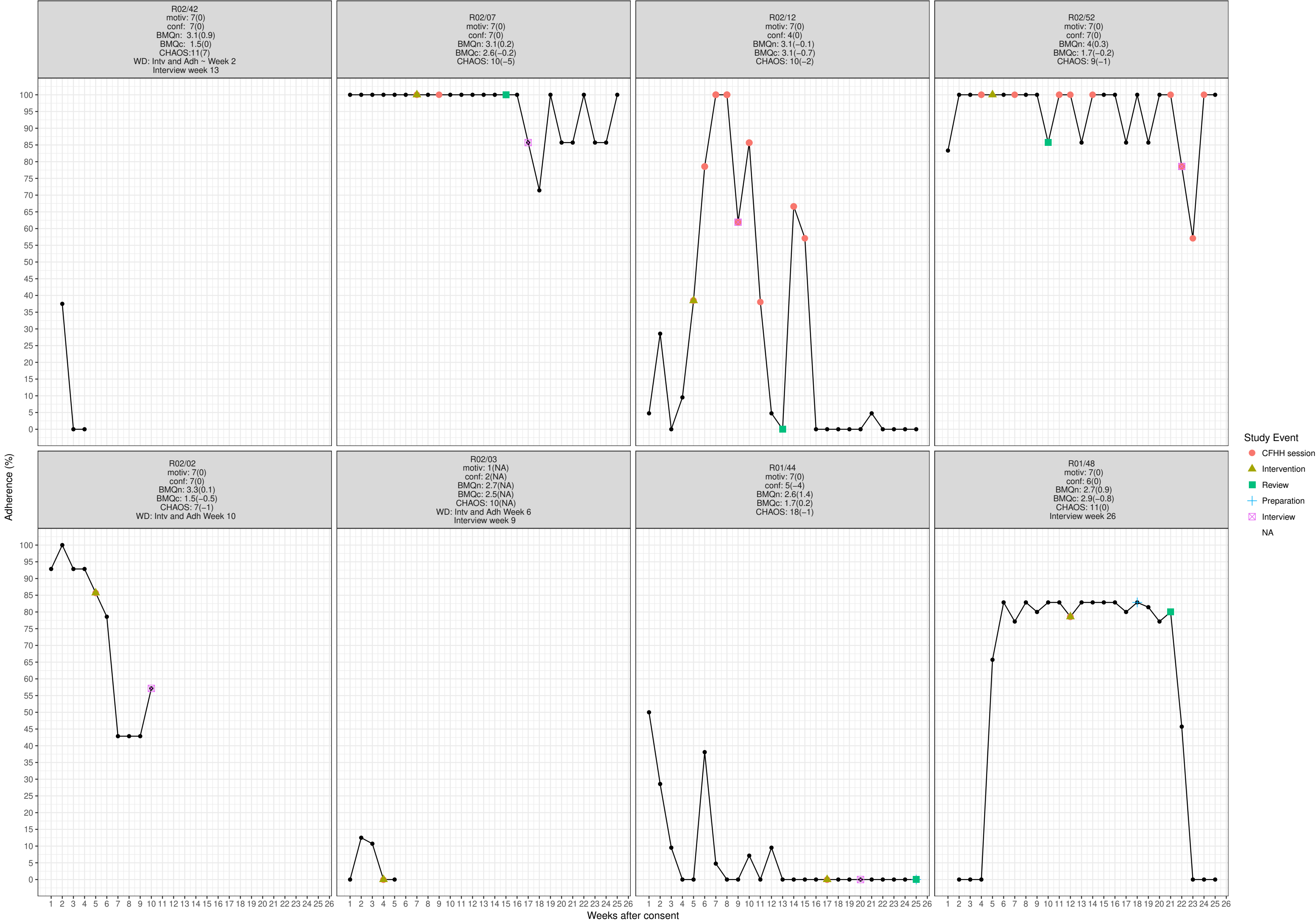

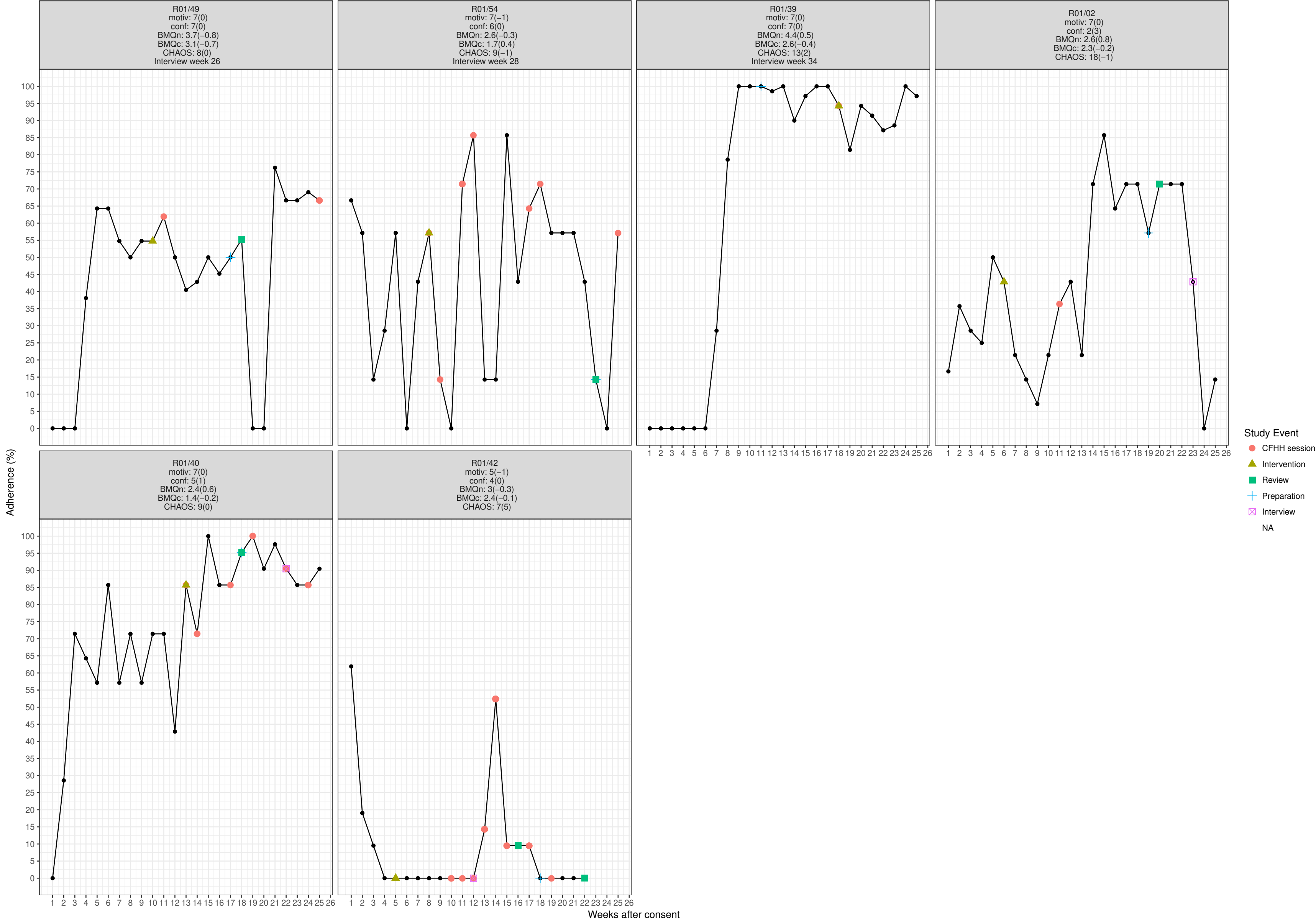

Supplement: Supplementary data [file bmjopen-2020-039089supp001.pdf]
