# Supplementary material for: Feasibility study for supporting medication adherence for adults with cystic fibrosis: mixed-methods process evaluation
Source: BMJ Open. 2020 Oct 27;10(10):e039089. doi: 10.1136/bmjopen-2020-039089 (PMC7592300; doi:10.1136/bmjopen-2020-039089)
Supplement: Supplementary data [file bmjopen-2020-039089supp003.pdf]

Additional File 03 - Quantitative results from process evaluation

Table a. Key dates in process evaluation by participant

Study ID	Interview Date	Baseline date	5 month follow up date	Date of first intervention meeting	Time in the trial at interview (days)	Time since first intervention session at interview (days)	Time in trial at follow up (days)
R02/02	13/09/2016	06/07/2016	10/11/2016	05/08/2016	69	39	127
R02/03	09/09/2016	08/07/2016	NA	05/08/2016	63	35	NA
R02/42	12/10/2016	15/07/2016	21/12/2016	NA	89	NA	159
R02/07	15/11/2016	12/07/2016	12/12/2016	09/09/2016	126	67	153
R02/12	02/11/2016	14/07/2016	03/01/2017	05/10/2016	111	28	173
R02/52	03/02/2017	04/07/2016	22/11/2016	05/10/2016	214	121	141
R01/44	01/12/2016	07/07/2016	16/11/2016	08/11/2016	147	23	132

R01/48	17/01/2017	04/07/2016	15/11/2016	13/10/2016	197	96	134
R01/49	30/01/2017	22/07/2016	07/12/2016	10/10/2016	192	112	138
R01/54	21/03/2017	25/07/2016	13/12/2016	02/11/2016	239	139	141
R01/39	27/02/2017	02/08/2016	21/12/2016	03/11/2016	209	116	141
R01/02	06/12/2016	31/08/2016	25/01/2017	15/08/2016	97	113	147
R01/40	05/12/2016	05/09/2016	17/02/2017	05/10/2016	91	61	165
R01/42	03/10/2016	12/09/2016	15/02/2017	15/08/2016	21	49	156

Table b. Interventionist-generated motivation data (intervention arm) R02/42, R02/49, R02/15 and R01/48 were all missing

Participant ID	Date	Consent Visit Motivation Rating	Was Participant motivation too low Answer Yes/No
R02/39	05.08.16	7	No
R02/40	23.08.16	4	No
R02/02	05.08.16	7	No
R02/03	03.08.16	1	No
R02/43	12.08.16	7	No
R02/05	22.08.16	5	No
R02/45	18.08.16	7	No
R02/07	09.08.16	7	No
R02/48	05.10.16	7	No
R02/10	14.09.16	7	No
R02/11	28.09.16	7	No
R02/50	26.09.16	7	No
R02/12	05.10.16	7	No
R02/52	03.10.16	7	No
R01/39	03.11.16	7	Page missing from report
R01/02	16.09.16	7	No
R01/03	03.10.16	5	No
R01/40	05.10.16	7	Page missing from report
R01/42	15.08.16	5	Page missing from report
R01/44	08.11.16	7	Page missing from report
R01/47	10.10.16	5	Yes
R01/06	10.10.16	7	Page missing from report
R01/49	17.10.16	7	No
R01/08	01.11.16	7	Page missing from report
R01/50	Missing report		
R01/53	29.11.16	7	Not ticked
R01/54	Missing report		
R01/10	10.11.16	2	Not ticked
R01/57	31.10.16	0	Yes

Table c. Engagement

	Adherence data collected (did not withdraw from data collection before 6m) n(%)	Total CFHH sessions Median (IQR)	Baseline adherence Median (IQR)
**Overall (n=33)**	29(88%)	3(1,8)	20(2.1,47.8)
**Qualitative case studies**			
**High adherence at end**			
R01/39	Yes	1	0
R02/07	Yes	2	96.7
R01/40	Yes	9	43.1
R02/52	Yes	13	96.6
**Moderate adherence at end**			
R01/49	Yes	4	13.2
**Low adherence at end**			
R01/54	Yes	11	44.8
R01/02	Yes	1	30.2
R01/48	Yes	3	1.8
R02/12	Yes	44	10.2
R02/03	No	3	5.4
R01/44	Yes	1	19.5
**Withdrawn**			
R01/42	Yes	41	21.1
R02/02	No	3	92.5
R02/42	No	0	4.2

Note: R02/42, R02/02 withdrew from adherence data collection and from the intervention and R02/03 was lost to follow-up. R01/42 did not withdraw from data collection until the end of the study; they did not contribute sufficient data for the 150-180 day period.

Table d. Activities: all participants

	Self-monitoring adherence	Tailored education about treatment	Tailored patient stories (videos)	Personalised action plan/Personalised goal-setting	Tailored problem-solving		Goal review; Rewards
	Clicks How am I doing?	Clicks Toolkit	Clicks Videos	Clicks Action Plan	Clicks Problem Solving	Clicks Coping Plan	Review sessions with Interventionist
**Mean (SD)†/Median* (IQR) overall (n=33)**	11(5 , 30)*	3(0 , 7)*	2(1 , 3)*	2(1 , 7)*	3(0 , 8)*	1(0 , 3)*	1(0.5)†
**Qualitative case studies**							
**High adherence at end**							
R01/39	8	3	1	1	0	1	1
R02/07	5	1	1	1	2	0	1
R01/40	52	0	1	1	3	0	1
R02/52	70	5	3	1	17	1	1
**Medium adherence at end**							
R01/49	30	2	1	0	1	0	1
**Low adherence at end**							
R01/54	24	4	5	3	4	2	1
R01/02	3	0	1	2	0	1	2
R01/48	38	6	2	7	7	1	1
R02/12	98	12	10	13	14	8	1
R02/03	15	12	1	25	1	14	1
R01/44	11	0	2	4	8	3	1
**Withdrawn**							
R01/42	69	18	9	16	20	3	2
R02/02	3	7	1	8	8	7	1
R02/42	0	0	0	0	0	0	0

Table e. Activities: highly motivated participants

(Those who answered 'No' to question, 'Was the participant motivation too low) n=17. Some of these were missing or not answered n=14, only 2 answered 'Yes'.

	Self-monitoring adherence	Tailored education about treatment	Tailored patient stories (videos)	Personalised action plan/Personalised goal-setting	Tailored problem-solving		Goal review; Rewards
	Clicks How am I doing?	Clicks Toolkit	Clicks Videos	Clicks Action Plan	Clicks Problem Solving	Clicks Coping Plan	Review sessions with Interventionist
**High motivation Mean (SD)†/Median* (IQR) overall (n=17)**	16 (5 33)*	5 (2 ,12)*	3 (1 , 4)*	4 (2 , 12)*	4(2 , 11)*	1(1 , 7)*	1.12(0.33)†
**Qualitative case studies (high motivation)**							
**R02/07**	5	1	1	1	2	0	1
**R02/52**	70	5	3	1	17	1	1
**R01/49**	30	2	1	0	1	0	1
**R01/02**	3	0	1	2	0	1	2
**R02/12**	98	12	10	13	14	8	1
**R02/03**	15	12	1	25	1	14	1
**R02/02**	3	7	1	8	8	7	1

Table f. Process Outcomes

	Accurate awareness of adherence	Increased Motivation	Increased necessity and decreased concern / beliefs Motivation		Increased self-efficacy /	Motivation	Increased habit /	Reduced CHAOS	Reduced barriers
	Subjective adherence (0-100): Medication Adherence Data Questionnaire	Change in BMQ question 'I want to do all my prescribed medications in the next 2 weeks (0-7)	Change in BMQ Necessities score (2-5)	Change in BMQ Concerns score (1-3)	Change in BMQ question 'I am confident I can do all my prescribed medications in the next 2 weeks (0-7)	Change in PAM activation score (0-100)	Change in SRBAI score (0-28)	Change in CHAOS score (0-24)	Change in no. of BMQ barriers ticked (0-6)
**n Overall**	30	31	31	31	31	31	31	31	31
**Mean (SD) overall**	2.07(27.87)	-0.1(1.27)	0.26(0.58)	-0.19(0.31)	0.06(1.79)	-2.38(14.01)	0.32(3.92)	0.1(2.75)	-1.84(3.44)
**Qualitative case studies**									
	baseline(change) %	**baseline (change)**			**baseline (change)**				
**High adherence at end**									
R01/39	85(14)	7(0)	0.5	-0.4	7(0)	-5.9	-2	2	-4
R02/07	100(-2)	7(0)	0.2	-0.2	7(0)	0	1	-5	-3
R01/40	92(8)	7(0)	0.6	-0.2	5(1)	7.2	-9	0	1
R02/52	95(-25)	7(0)	0.3	-0.2	7(0)	4.9	3	-1	1

R01/49	100(0)	7(0)	-0.8	-0.7	7(0)	9.9	-1	0	-4
**Low adherence at end**									
R01/54	60(-10)	7(-1)	-0.3	0.4	6(0)	-7.9	1	-1	6
R01/02	55(16)	7(0)	0.8	-0.2	2(3)	0	-1	-1	-2
R01/48	0(100)	7(0)	0.9	-0.8	6(0)	0	0	0	-2
R02/12	NA	7(0)	-0.1	-0.7	4(0)	14.6	-3	-2	-5
R02/03	50(NA)	1(NA)	NA	NA	2(NA)	NA	NA	NA	NA
R01/44	0(0)	7(0)	1.4	0.2	5(-4)	-16.6	0	-1	-5
**Withdrawn**									
R01/42	0(0)	5(-1)	-0.3	-0.1	4(0)	-5	-1	5	-6
R02/02	80(10)	7(0)	0.1	-0.5	7(0)	9.2	2	-1	1
R02/42	100(0)	7(0)	0.9	0	7(0)	-12.1	1	7	-1

Table g. Intermediate Outcomes

	End of trial adherence (day 150-180)✦	Change in Objective adherence✦ (%)	Change in FEV1	Number of exacerbations in 6 months
**Mean (SD)⁺/ Median (IQR)* overall (n=33)**	34.7 (0.4 ,78)*	1.25(-5.8 , 36.3)*	0.1(0.51)⁺	1(0 , 2)*
**Qualitative case studies**				
**High adherence at end**				
**R01/39**	95.2	95.16	-0.02	1
**R02/07**	93.5	-3.12	NA	0
**R01/40**	88.2	45.07	0.22	0
**R02/52**	83.9	-12.68	-0.13	0
**Medium adherence at end**				
**R01/49**	68.3	55.06	-0.12	3
**Low adherence at end**				
**R01/54**	29	-15.8	-0.03	2
**R01/02**	29	-1.14	0	0
**R01/48**	5.2	3.34	1.07	0
**R02/12**	0	-10.23	-0.21	0
**R02/03**	0	-5.42	NA	NA
**R01/44**	0	-19.54	0.9	1
**Withdrawn**				
**R01/42**	NA	NA	0	0
**R02/02**	NA	NA	-0.04	3
**R02/42**	NA	NA	0.35	1

✦Normative numerator adjusted adherence
