# Supplementary material for: Feasibility study for supporting medication adherence for adults with cystic fibrosis: mixed-methods process evaluation
Source: BMJ Open. 2020 Oct 27;10(10):e039089. doi: 10.1136/bmjopen-2020-039089 (PMC7592300; doi:10.1136/bmjopen-2020-039089)
Supplement: Supplementary data [file bmjopen-2020-039089supp004.pdf]

Additional File 04: Joint display table (data sources in **bold**)

#	Logic model column / construct	Quantitative	Qualitative	Convergence code
	**INPUTS**			
1	MDT introduction to CFHealthHub	-	**Chief investigator reported:** introducing MDT to concept behind and application of CFHH.	-
2	CF Clinicians aware of the importance of monitoring adherence	-	**Chief investigator reported:** briefing collaborating MDTs. Reported change agents at centres internalised idea; some residual scepticism among senior physicians.	-
3	Prescription data	Study team found adherence levels of over 100% **(Implementation log, 01 Dec 16)**	Late identification of prescription changes found to be responsible. **(Minutes, Trial Management Group Meeting 10 Jan 17)**	Expansion
4	Chipped nebuliser	-	Devices ordered centrally by CTRU were delivered to sites on 20th May 2016 and processed for distribution on 23rd June 2016. **(Project manager emails)**	-
5	Qualcom-Hub (docking & upload)	-	Devices ordered centrally by CTRU were delivered to sites on 20th May2016 and processed for distribution on 23rd June 2016 **(Project manager emails)**	-
6	CFHealthHub website/app	-	Available, but under development through trial **(Additional File 01)**	-
7	COM-BMQ questionnaire	COM-BMQ questionnaire data was collected at baseline for all consenting participants	-	-

	responses	**(Additional File 04 - Table 8)**		
8	Intervention manual	-	High levels of interventionist satisfaction with manual. R01 Interventionist 1 remarked that, "all the stuff in the manuals was really good."	-
9	Interventionist training programme	**Structured questionnaire on interventionist confidence after training programme:** Interventionists (n=5) all averaged >8 for confidence across 11 questions. Isolated scores of <8 occurred three times: viewing charts/tables, completing report forms and understanding online training/assessment.	**In interviews**, interventionists reported high levels of satisfaction; one requested for more integration of research and intervention procedures. R01 Interventionist 1 remarked "You had the manual but I was missing bits". She wanted more case studies and mock patients in the training to compensate for this. An interventionist (R01 MDT member 1), who was a social worker by background, found the training very good, indicating that it the training had acceptability beyond physiotherapists.	Expansion
10	Interventionist support	-	**Research team member (MH) reported** giving mentorship and that one site/trust received more support from the PI than the other. The main interventionist at the other site received support from the part-time interventionist who was a member of the multi-disciplinary team.	-
11	Competency/Fidelity assessment	**Structured instrument for the assessment of interventionist competence:** Digital recordings were made and assessed for fidelity by MA, MH and JB. Fidelity assessment instrument modified after discussion, in advance of use on full-scale RCT.	-	-

12	Motivated and effective interventionists	-	**In interviews**, interventionists reported that they were enthusiastic about the intervention	-
	**ENGAGEMENT**			
13	Clinicians accessing adherence data*	Clinicians did not access CFHH. **(CFHH Click analytics)**	**In interviews**, interventionists talked about run charts occasionally being viewed when brought to MDT meetings by interventionists.	Confirmation
14	Adherence data tracking	**CFHH click analytics** showed interventionists accessing data before meetings	This was confirmed **in interviews**.	Confirmation
15	Participant accessing CFHealthHub	**Click analytics:** The median number of sessions over 5 (+/- 1) months was 3 (interquartile range 1 to 8, range 1-44, **Additional File 05 - Table c**), with a mean duration of 36.1 (SD=23.9) minutes. The mean total duration of interaction time across the study was 49.3 (SD 44.8) minutes. The mean length of an interaction was 12.4 (SD=9.6) minutes. The median number of days in the trial with interactions was 2 (IQR=1,7).	Lack of usability was explained **in interviews** by initially difficult login procedures and the lack of a mobile app for most of the pilot trial, leading participants to access an unsatisfactory desktop version on their mobile.	Expansion
16	Push notifications/reminders each week*	-	**Programmer reported that** automated push notifications not available during pilot trial. **In interviews**, one participant and one interventionist, reported the spontaneous development of informal push notifications in which the interventionist was ringing up and praising the participant for accomplishments, thereby building the relationship.	-
17	CFHealthHub Intervention sessions delivered according	**Collected via project-specific structured fidelity assessment instrument (#11).** After discussion	**Fidelity observations indicated:** limited discussion of motivations; communication style	Expansion

	to Manual (Fidelity)	between MA, MH and JB summary scores were agreed for delivery of content 100% and quality of delivery: 60-92%. Co-author Judy Bradley is intending to publish this work elsewhere.	sometimes paternalistic rather than autonomy-enabling; insufficient attention to most active ingredients.	
18	Initial session, and then review at each clinic visit	**Collected via click analytics.** Patient run charts reveal a disparity in when and whether these happened **(Additional File 07)**.	-	-
	**ACTIVITIES**			
19	Clinicians monitor adherence	-	Clinician access to adherence data was sporadic (see #13) and **staff interviews confirmed** that it was not monitored. **In an interview**, participant R01/02 described the research intervention as “parallel rather than integrated” with mainstream clinical management.	-
	Intervention components for all participants			
20	Self-monitoring adherence	**Click analytics:** 'How am I doing?' pages were the most frequently visited in terms of the total number of clicks during the trial. 30 (90.9%) participants clicked a median of 11 (range 5-30) times in 5 months, but sometimes in a single session (**Additional File 05 – Table d**). Access did not always result in good alignment between subjective and objective adherence (**Additional File 05 – Tables f and g respectively**).	**In interviews**, moderate and frequent users said they mostly valued this page for self-monitoring.	Expansion
21	Tailored education about treatment	**Click analytics:** Toolkit clicked a median 3 (range 0-7) times **(Additional File 05 – Table d)**.	**In participant interviews**, the DNASE video was popular. Other pages were accessed	Expansion

			infrequently or when issues arose, when the information was viewed as "more down to earth" (R02/07) than technical manuals.	
22	Tailored patient stories (videos)	**Click analytics:** 'Talking heads' videos accessed a median 2 (range 1-3) times **(Additional File 05 – Table d)**.	**In participant interviews**, these videos divided opinion. Some participants liked to know that they were not alone; others did not want to see videos of others with CF.	Expansion
	Intervention components for those with adequate motivation			
23	Personalised goal-setting	**Click analytics:** Participants set target adherence levels in CFHH **(Additional File 05 – Table 3)**.	**In interviews**, participants reported goal-setting, but it was not clear how much it came from patients and how much from interventionists.	Expansion
24	Goal review	**Click analytics:** Mean (SD) review sessions 1 (0.5) **(Additional File 05 – Table e)**.	-	-
25	Personalised action plan	**Click analytics:** Action plan pages clicked on median 2 (inter-quartile range 1-7) times **(Additional File 05 – Table e)**.	Disliked by some participants who, the interventionist from centre R01 **reported during an interview**, found writing down action plans like "being at school"	Expansion
26	Tailored problem-solving	**Click analytics:** Problem solving and coping plan pages clicked on median 3 (inter-quartile range 0-8) and 1 (0-3) times respectively **(Additional File 05 – Table e)**.	**In interviews**, one participant realised that when she goes to her friend's house, rather than missing a treatment she could do it in the car or anywhere. One interventionist from centre R02 thought it important that the information was	Expansion

			"there if you need it" for patients.	
	**IMMEDIATE OUTCOMES**			
27	Medical care informed by adherence	**-**	**Interviews** with PIs found that the trial and intervention ran alongside usual care rather than being informed by it (see also #13, #19).	-
	For all participants			
28	Acute awareness of adherence / increased Motivation	Answers to the **subjective adherence question (Additional File 05 – Table f)** were well aligned with **run charts (Additional File 07)** in those with high adherence. Alignment was more variable in those with moderate and poor adherence.	**In interviews**, some with high adherence used the CFHH "How am I doing page" (run charts) as a check (R02/07, R01/40); other high adherers did not (R01/49). Some felt that it increased their adherence, acknowledging that monitoring meant that they had, "…better make an effort here".	Expansion
29	Increased necessity and decreased concern	No change in the group averages for the **COM-BMQ** (incorporating Beliefs about Medicines Questionnaire - specific (Nebuliser adherence) 21-item validated self-report tool[1]) or Patient Activation Measure (**PAM-13**) (Health Style Assessment) assessment of patient knowledge, skill, and confidence for self-management[2]. **(Additional File 05 – Table f)**	-	-
30	Increased self-efficacy / Motivation	No change in the group averages for a single question about confidence to adhere or the PAM-13. **(Additional File 05 – Table f)**	-	-
	For those with adequate			

	motivation			
31	Increased self-efficacy/ Motivation	No change in the group averages for a single question about confidence to adhere or the **PAM-13**. **(Additional File 05 – Table f)**	-	-
32	Increased habit / Reduced CHAOS	No change in the group averages for **Self-Report Behavioural Automaticity Index (SRBAI)** automaticity-specific subscale of the Self Report Habit index to capture habit-based behaviour patterns[3] or in the assessment of routine measure of **life chaos**[4]. **(Additional File 05 – Table f)**	-	-
33	Reduced barriers	No change in the group averages for The Beliefs about Medicines Questionnaire - specific (Nebuliser adherence) (**BMQ 21-item**) **(Additional File 05 – Table f)**	The tailored problem-solving modules (#26) were not widely used but, **in interviews**, party plans and nebuliser guides were cited as having removed barriers by those who did use this content. For instance, one participant was able to find the technical name for a part of a nebuliser for which he needed to order a replacement.	Expansion
	**INTERMEDIATE OUTCOMES**			
34	Treatment optimisation	-	**Interview data** revealed patients to be behaving in unexpected ways, for instance taking holidays from their treatment or not taking medication as prescribed.	-
35	Increased adherence	**Nebuliser data via CFHH:** Mean adherence across all participants was 10 (95% CI: -5.2 to 25.2) percent higher in the intervention than in the	-	-

		control arm. Within the case study participants (all intervention), an increase of 7.5% (95% CI: -8.2-23.1) in simple normative adherence with numerator adjustment can be observed in the intervention arm. Following month 1, adherence is consistently higher in the intervention arm with the greatest difference observed in month 5 (mean difference: 10.8, 95% CI: -11.44, 22.9). These differences would indicate a potentially clinically important difference between the intervention and usual care arms.		

[1] R. Horne, J. Weinman, M. Hankins, The beliefs about medicines questionnaire: The development and evaluation of a new method for assessing the cognitive representation of medication, Psychol. Health. 14 (1999) 1–24. doi:10.1080/08870449908407311.

[2] J.H. Hibbard, E.R. Mahoney, J. Stockard, M. Tusler, Development and testing of a short form of the patient activation measure., Health Serv. Res. 40 (2005) 1918–30. doi:10.1111/j.1475-6773.2005.00438.x.

[3] B. Verplanken, S. Orbell, Reflections on Past Behavior: A Self-Report Index of Habit Strength1, J. Appl. Soc. Psychol. 33 (2003) 1313–1330. doi:10.1111/j.1559-1816.2003.tb01951.x.

[4] M.D. Wong, C. a. Sarkisian, C. Davis, J. Kinsler, W.E. Cunningham, The Association Between Life Chaos, Health Care Use, and Health Status Among HIV-Infected Persons, J. Gen. Intern. Med. 22 (2007) 1286–1291. doi:10.1007/s11606-007-0265-6.
