# Supplementary material for: Feasibility study for supporting medication adherence for adults with cystic fibrosis: mixed-methods process evaluation
Source: BMJ Open. 2020 Oct 27;10(10):e039089. doi: 10.1136/bmjopen-2020-039089 (PMC7592300; doi:10.1136/bmjopen-2020-039089)
Supplement: Supplementary data [file bmjopen-2020-039089supp005.pdf]

Additional File 05 - Case-ordered descriptive matrix for fourteen case studies

Qualitative findings in italics. Otherwise, motivation, confidence, necessities, concerns, life chaos and subjective adherence (baselines and process outcomes) from self-report instruments (see Methods and Additional File 04). Engagement, activities and data captured by CFHealthHub.

**Case / baselines / context**	**Engagement**	**Activities**	**Process outcomes**	**Intermediate outcomes**
**High adherence (average >80%) in last month of trial**				
**R01/39.** High motivation, confidence and necessities, medium concerns, quite high chaos. *They got a lot of information about CF from other websites.*	Used CFHH once. *Very engaged with interventionist and trial.*	*Didn't make plans – felt it was her responsibility to adapt her life; found others monitoring helpful. Didn't like videos or social aspects of website because of the reminder of her mortality.*	*Knowledge that clinicians could access treatment adherence information provided extra motivation to adhere.*	End of trial adherence 95% (95% improvement).
**R02/07.** High motivation, and confidence, medium-high necessity, medium concerns and chaos. Existing high adherer, *sees treatment as a "plan for longevity" rather than a "chore".*	Used CFHH twice. *Didn't find it useful or like the videos (doesn't want to see negative side of CF).*	Made action plan, accessed some modules once. *Found goal-setting with interventionist helpful.*	Little change as already, motivated. Reduced CHAOS and barriers.	End of trial adherence 93% (3% decline).
**R01/40.** High motivation, medium confidence and necessities, low concerns, medium-to-low chaos. *Was recruited soon after exacerbation.*	Had nine CFHH sessions. *"I've been logging on to track my progress... every two weeks to a month". Finds others monitoring him helpful.*	Frequent self-monitoring. *Compensates for slippages by planning to do the rest of his doses.*	Motivation already high, but habit lacking. *Intervention has made him think about adherence more than he did before.*	End of trial adherence 88% (45% improvement). Variance over trial, but trajectory.
**R02/52.** High motivation, confidence and necessity, low concerns, low-medium chaos. Existing good adherer; *wanted something like a fitness tracker with feedback - messages on performance.*	13 CFHH sessions. *Liked the more portable nebuliser, could take it away on work.* CFHH session that precedes interventionist visit explained by interventionist testing login details.	Frequent self-monitoring, regular use of tailored education and problem solving (fixing nebuliser problems) and some use of videos. *Wanted it expanding to physical activity.*	Motivation already high. Increased habit.	End of trial adherence 83% (12% decline).

Case / baselines / context	Engagement	Activities	Process outcomes	Intermediate outcomes
**Moderate adherence (average 50-80%) in last month of trial**				
**R01/49.** High motivation, confidence, medium-high necessity and concerns low chaos. *Participated to 'prove' themselves to their physiotherapist*; poor awareness of own adherence not improved over course of trial.	4 CFHH sessions	Used problem-solving modules and self-monitoring, but no action plan.	Increased motivation, reduced barriers.	End of trial adherence 68% (55% improvement). An important improvement from low adherence, but subjective adherence still poorly 'calibrated' with objective adherence.
**Poor adherence (>50%) in last month of trial**				
**R01/54.** Professed high motivation and confidence, medium necessity, low concerns, medium to low chaos. *Wants the doctor "to notice" that they are adherent to their treatment, demotivated by the fact they don't.*	44 CFHH sessions. *Appreciative of extrinsic motivation from face-to-face contact with interventionist.*	Frequent self-monitoring; initially high use of action plans and problem solving. *Dislikes 'talking heads' videos.*	More barriers by the end of the trial.	End of trial adherence 29% (16% decline), but run chart shows huge variance week by week.
**R01/02.** High motivation, low confidence, medium necessity and concerns, high chaos. *Dissatisfaction at service reconfiguration: moved across from Poole to Southampton during trial. Upset that wider team isn't noticing their adherence.*	Used CFHH once *but had technical problems. Appreciative of interventionist: "Having a personal contact and someone to guide you through it is really useful" Wider team not talking about adherence: "parallel rather than integrated".*	Two review sessions with interventionist.	Reduced CHAOS and barriers; increased self-efficacy	Lack of pre-post change not contradicted by the run chart which shows improvement.

Hind D, *et al. BMJ Open* 2020; 10:e039089. doi: 10.1136/bmjopen-2020-039089

**Case / baselines / context**	**Engagement**	**Activities**	**Process outcomes**	**Intermediate outcomes**
**R01/48.** professed high motivation and confidence, medium-high necessities and concerns; medium chaos. *This 69-year old doesn't like nebulising; "can't teach an old dog new tricks". No belief in benefit of nebulised medication. Poor awareness of own adherence. Altruistic trial participant.*	Used CFHH three times. *Access problems (passwords, etc) - gave up.*	Some engagement with toolkit, action plans and problem-solving, *didn't like the videos.* Engagement drops off as soon as the last meeting over.	No change in process outcomes.	End of trial adherence 5% (3% improvement). *Said was making an effort for the trial.* In line with this, objective adherence was high (~80%) for weeks 6-21
**R02/12.** High motivation, medium to low confidence, medium to high necessity and concerns, medium chaos.	Started off engaged, lots of CFHH use and two intervention sessions in first 100 days, nothing thereafter.	Made plans, liked website, checked graphs. *Liked face-to-face interaction with interventionist.*	Decreased chaos and barriers but also decreased habit.	Initial improvement in adherence (up to 100% between weeks seven and nine after first intervention not sustained over time. Review stimulates brief improvement at week 15, again not sustained.

Case / baselines / context	Engagement	Activities	Process outcomes	Intermediate outcomes
**R02/03.** Low motivation and confidence, medium necessities, concerns and chaos. *Treatment is something that he has to do but doesn't want to do it, or think about CF. Forgets about treatment because of busy lifestyle. Prioritises other things above health. Knows that this doesn't end well, but no readiness to change.*	Minimal short-term engagement with CFHH. *Interventionist notes that participant has always been difficult to get hold of.*	Made action and coping plans, checked graphs.	No process data at follow-up.	Withdrew from treatment early.
**R01/44.** High motivation, medium confidence, necessities, low concerns, high chaos *("I can't seem to get into a routine")*. *Recruited during exacerbation:* baseline artificially high. Intervention 1 visit didn't happen until Week 17. *Participant describes self as "uncompliant" except around inpatient stays.*	One CFHH session (at intervention visit 1). Interventionist appears not to have done correct preparation. Only participant rated by an interventionist as having inadequate motivation.	*Participant confirms that he made action plan, coping plan and checked graphs with interventionist but chaotic lifestyle and low motivation prevented further use. Admits only has a routine in hospital.*	No change in process variables.	Initial spikes of adherence not sustained over time.

**Withdrawn**				
**Case / baselines / context**	**Engagement**	**Activities**	**Process outcomes**	**Intermediate outcomes**
**R01/42.** Medium motivation, low confidence, medium-high necessity, medium concerns, low chaos. *Originally an i-neb user. Does not think nebulising three times a day is achievable. Moved house during study. No broadband – so didn't do nebulisations.*	*Loved the website and shared it.* 41 CFHH sessions. *Intervention visit 1 reported to be chaotic.*	Made action plan.	Little change in process variables.	Interview might have triggered brief increase in nebuliser use, when *participant realised nebulisations were being logged even when he wasn't plugging it in.*
**R02/02.** High motivation and confidence, medium-high necessity low concerns and chaos. *Interview shows them to be motivated by interventionist visit and qualitative interview (Hawthorne effect).* Subjective adherence poorly aligned to objective adherence.	Limited engagement. Three CFHH Sessions all on the same day.	Made an action plan *but reported that she didn't set goals because she thought she her adherence was already good.*	Little change in process variables.	Adherence run chart starts off high, but drops off quickly. Interview might have triggered brief increase in nebuliser use. Withdrew from collection of nebuliser data collection.
**R02/42.** High motivation and confidence, medium to high necessity, low concerns, medium chaos	Withdrew - *didn't like the eTrac nebuliser - delivering the drug too quickly made them cough. Interventionist encouraged discontinuation.*	*Didn't look at the website.*	No change in process outcomes	Assumed no change in adherence, but objective lacking.

Hind D, *et al. BMJ Open* 2020; 10:e039089. doi: 10.1136/bmjopen-2020-039089
