# Supplementary material for: Feasibility study for supporting medication adherence for adults with cystic fibrosis: mixed-methods process evaluation
Source: BMJ Open. 2020 Oct 27;10(10):e039089. doi: 10.1136/bmjopen-2020-039089 (PMC7592300; doi:10.1136/bmjopen-2020-039089)
Supplement: Supplementary data [file bmjopen-2020-039089supp006.pdf]

ACtiF Pilot Statistical Report

L Mandefield

Methods

Outcomes

Feasibility outcomes

The primary objective of this study was to determine the feasibility of proceeding to a definitive trial. An external pilot randomised controlled trial to determine feasibility of a randomised controlled trial based on objective stop-go criteria related to:

(a) participant recruitment;

(b) participant retention; and,

(c) quality of primary outcome data at 5 (+/- 1) months post randomisation.

These were assessed by

i. The number of screened, eligible and recruited participants per month, per centre and overall;

ii. The number and percentage of participants who complete their 5(+/-1) month post randomisation follow up;

iii. The number of Fuchs criteria by exacerbation.

Clinical outcomes

The primary clinical outcome measure was the number of pulmonary exacerbations in the 5 (+/-1) month post-baseline follow-up period, defined according to a modified version of the Fuchs criteria. The original Fuchs criteria was 4 out of 16 symptoms leading to IV antibiotic treatment. An exacerbation of respiratory symptoms will be said to have occurred when a participant was treated with parenteral antibiotics for any one of the following 12 signs or symptoms:

1. change in sputum;
2. new or increased hemoptysis;
3. increased cough;
4. increased dyspnea;
5. malaise, fatigue, or lethargy;
6. temperature above 38 °C;
7. anorexia or weight loss;

8. sinus pain or tenderness;

9. change in sinus discharge.

10. change in physical examination of the chest, derived from notes by site staff.

11. decrease in pulmonary function by 10 percent or more from a previously recorded value, derived from notes by site staff; or,

12. radiographic changes indicative of pulmonary infection, derived from notes by site staff.

The trial interventionist or prescribing clinician/nurse will collect data on the "exacerbations" form at the point of a participant starting a course of IV antibiotics.

The following secondary outcomes were also collected at baseline and 5 (+/-1) month follow up:

1. Body Mass Index (BMI).
2. Forced expiratory volume in 1 second (FEV1): standardised spirometry as a measure of condition severity.
3. EuroQol EQ-5D-5L: generic health status measure for health economic analysis.
4. The Patient Activation Measure (PAM-13): assessment of patient knowledge, skill, and confidence for self-management.
5. Confusion, Hubbub, and Order Scale (CHAOS 6-item): measure of life chaos.
6. Medication Adherence Data-3 items (MAD-3)
7. Self-Report Behavioural Automaticity Index (SRBAI)
8. Cystic Fibrosis Questionnaire-Revised (CFQ-R): disease specific health-related quality of life instrument.
9. The Patient Health Questionnaire depression scale (PHQ-8): severity measure for depressive disorders.
10. The General Anxiety Disorder 7-item anxiety scale (GAD-7): severity measure for anxiety.
11. The Capability Opportunity Motivation Behaviour Beliefs Questionnaire (COM- BMQ): This questionnaire incorporates:

a. The Beliefs about Medicines Questionnaire - specific (Nebuliser adherence) (BMQ 21-item): a validated self-report tool, customised by the author to identify perceived necessities and concerns for nebuliser treatment.
b. The following project-specific items: one additional belief item, one intention item, one confidence item, and a list of barriers. These will serve as a tailoring tool for the intervention and also as a secondary outcome measure. 12.Subjective adherence single question: self-report estimate of adherence as a percentage. Self-reported problems: identification of capability and opportunity barriers to nebuliser adherence

13. Concomitant medications: bespoke instrument, designed for this research project.
14. Resource use form: interventionist collects data from a combination of hospital notes and the NHS patient electronic system to determine 1) inpatient IV days 2) Routine clinic visits 3) Unscheduled outpatient contacts 3) unscheduled inpatient stays.
15. Prescription: a monthly prescription check to both check for data transfer to CFHealthHub and review for an indication that the prescription has changed or indication of microorganism e.g.
16. Adherence to prescribed medication
17. Any treatment with IV antibiotics

Sample Size

Sample size calculation was based on estimating parameters within a certain amount of precision rather than hypothesis testing. The sample size for a feasibility study should be adequate to estimate the uncertain critical parameters (standard deviations for continuous outcomes; consent rates, event rates, attrition rates for binary outcomes) needed to inform the design of the full RCT with sufficient precision.

To assess recruitment rate, the external pilot RCT ran in two CF units for 12 months, with four months recruitment, one months 'run-in' period (the period between the consent and baseline visit), and 5 (+/-1) months follow up. To match the proposed recruitment rate of the main RCT, the target sample over the four months for which the pilot RCT was open, was 32 per centre (64 in total from the two pilot centres). We aimed to see a minimum of 75% of the recruitment target to be confident of the trial viability i.e. at least 48 patients in total consented and randomized in four months' of recruitment from two centres.

Randomisation

Randomisation was conducted using a computer generated pseudo-random list with random permuted blocks of varying sizes, created and hosted by the Sheffield CTRU in accordance with their Standard Operating Procedures (SOPs) and was held on a secure server. ACtiF participants will be randomised in a 1:1 ratio, intervention to control arms, stratified by:

- Site;
- Number of IV days in previous 12 months as collected at consent visit (two categories will be (i) less than or equal to 14 days and (ii) greater than 14 days).

Study researchers accessed the allocation for each participant by logging in to the remote, secure internet-based randomisation system. Once a participant had consented to the study, the researcher logged into the randomisation system and entered basic demographic information. After this information had been entered the allocation for that participant was then revealed to the researcher.

Block randomisation with randomly varying block size of 2, 4 and 6 was used so that the sequence of allocation could not be predicted. The block sizes were determined by the trial statistician and block size was not revealed to any other member of the study team.

Blinding

The trial statisticians remained blind until data freeze, at which point unblinded data was presented to them so checks could be carried out.

Statistical Methods

All statistical analyses were performed in R version 3.3.1.

Analysis Populations

The ITT population includes all participants for whom consent was obtained and who were randomised to treatment, regardless of whether they received the intervention or not. This is the

primary analysis set and endpoints were summarised for the ITT population unless otherwise stated.

Participant Flow

A CONSORT flow diagram was used to display data completeness and patient flow from first contact to final follow up.

The number of participants recruited at each centre each month was presented. The number of participants who withdrew consent from the trial, withdrew from the intervention, withdrew from collection of the primary outcome, withdrew consent from adherence data collection and who were lost to follow up were presented overall, by treatment arm and site. The reasons for attrition, where given, were presented.

Patient reported outcome measures (PROMS)

The following PROMS were completed at baseline and 5 (+/-1) month follow up visit. For detailed methods of how these questionnaires were scored, please see the appendix.

Data completeness

A CONSORT flow diagram was used to display data completeness and patient throughput from first contact to final follow up.

Baseline characteristics

Participants' demographics (age, sex, ethnicity, IMD decile), physical measurements (weight, height, BMI), clinical measurements (FEV1, IV days in last registry year, Pseudomonas status, Adherence in first 2 weeks, Subjective adherence, Medication, Treatment burden) patient reported outcomes (EQ-5D-5L, PAM-13, CHAOS, MAD-3,SRBAI, CFQ-R, GAD-7, COMBMQ, PHQ-8). Imbalance between treatment arms was not tested statistically but were reported descriptively.

Primary effectiveness analysis of clinical outcomes

The primary endpoint of the study is the number of exacerbations in a 5 (+/- 1) month period. Exacerbations were defined as being treated with IV antibiotics and meeting at least 1 Fuchs criteria.

The number of exacerbations by participant were presented. The number and percentage of exacerbations with each Fuchs criteria were presented. The length of IV course was summarised by intervention arm for all exacerbations and for participants experiencing exacerbations.

The primary effectiveness analysis used a negative binomial model and included all exacerbations in a 6 month follow up period. Participants who were not followed for this length were excluded. An adjusted model included IV days in the previous 12 months as a covariate. Although not prespecified, a further sensitivity analysis was carried out. This model included the number of days followed up as an offset. This allowed all consenting participants to be included. An adjusted offset model included IV days in the previous 12 months as a covariate.

Secondary effectiveness analysis of clinical outcomes

Patient reported outcome measures

Secondary outcomes were measured at baseline and 5 (+/-1) months post randomisation. The mean difference between treatment arms was calculated for each of the secondary outcomes, along with 95% confidence intervals using a multiple linear regression model. Adjustment for baseline and site was carried out and both unadjusted and adjusted results were presented.

Adherence to medication

The time of inhalations of medication was recorded via chipped nebulisers. This data along with prescription data was used to calculate a number of different adherence measures. Adherence in people with CF is of key importance. For this reason, it was decided that 7 separate measures of adherence to prescribed medication were to be presented:

1. Total doses;
2. Unadjusted adherence;
3. Simple normative adherence (without numerator adjustment);
4. Sophisticated normative adherence (without numerator adjustment);
5. Simple normative adherence (with numerator adjustment);
6. Sophisticated normative adherence (with numerator adjustment);
7. Subjective single adherence.

Measures 1-6 are calculated daily based on the chipped nebuliser data and the dose prescribed that day. Means can be calculated for set periods, e.g. weekly.

The specific calculations of these adherence measured are described below.

Total doses taken

As a basic, unadjusted measure of adherence, the total number of doses taken for the time period will be calculated.

Unadjusted adherence

Adherence is typically calculated as the dose taken divided by the dose described per day.

Simple normative adherence (without numerator adjustment)

Quality of adherence reporting is dependent on the PWCF being prescribed the appropriate medications. Adjusting the denominator of the adherence calculation controls for treatment rationalisation to try reduce treatment burden, which is an approach often seen in people in CF. The simple normative adherence is calculated as follows:

1. If the participant does not have pseudomonas

- Minimum denominator is set at 1 treatment/day.

2. If the participant has chronic pseudomonas
- Minimum denominator is set at 3 treatments/day
3. The participant has chronic pseudomonas and intermittent inhaled antibiotic regimens
- Minimum denominator is 3 treatments/day during 28 day 'on' period
- Minimum denominator is 1 treatment/day during 28 day 'off' period
4. The participant has intermittent pseudomonas
- Minimum denominator is 3 treatments/day for 1 or 3 months depending on the eradication regime
- Minimum denominator is 1 treatment/day for the rest of the time

In calculating normative adherence an expected minimum prescription based on a patient's health state is needed. Most patients take a dose of a mucolytic, and patients meeting the criteria will take two doses of antibiotics. In adherence calculations, participants had their denominator amended to reflect their prescription. A complication arises in denominator adjustments when the antibiotic prescribed is one that is expected to be used in an alternating fashion (e.g. 28 days use, 28 days off). The antibiotic medications Aztreonam Lysine and Tobramycin are normally prescribed in this way; for patients with prescriptions for these medications with periods of more than 28 days without a prescription for an antibiotic, the denominator was adjusted to add in 2 doses / day. After 28 days of substituted antibiotic use, a 28 'day off' cycle was programmed. This cycle was continued until such time as another antibiotic prescription was present.

Sophisticated normative adherence (without numerator adjustment)

The sophisticated normative adherence is calculated as follows:

1. If someone has 'mild genotype', is pancreatic sufficient and has FEV1 > 90%, without Pseudomonas and used <= 14 days intravenous antibiotics in the past 1 year.
- There is no minimum target. Denominator is determined by the agreed prescription between clinicians and participants.
2. If someone is homozygous for class I-III CFTR mutation OR pancreatic insufficient OR FEV1 <= 90%, but without Pseudomonas and used <= 14 days intravenous antibiotics in the past 1 year. Minimum denominator is set at 1 treatment/day.
3. If the person has chronic pseudomonas AND/OR
- the person used > 14 days intravenous antibiotics in the previous year Minimum denominator is set at 3 treatments/day
4. If the person has chronic pseudomonas AND/OR used > 14 days intravenous antibiotics in the previous year but is on intermittent inhaled antibiotic regimens
- Minimum denominator is 3 treatments/day during 28 day 'on' period
- Minimum denominator is 1 treatment/day during 28 day 'off' period
5. If someone has intermittent pseudomonas but used <= 14 days intravenous antibiotics in the past 1 year

- Minimum denominator is 3 treatments/day for 1 or 3 months depending on the eradication regime
- Minimum denominator is 1 treatment/day (or 0, i.e. no minimum target) depending on their genotype, pancreatic status and FEV1 for the rest of the time.

Numerator adjustment in simple and sophisticated normative case

Numerator adjustment occurs only if a daily adherence measure is greater than 100%, thus the maximum daily adherence is set at 100%.

Subjective single adherence

All participants will be asked to estimate their adherence as a percentage at baseline, clinic visits, 5(+/-1) months and any further visits up to 30th April 2017. These subjective measures were presented separately. The question referred to the previous 2 weeks.

Adherence summaries

The mean and SD was calculated for each month of the trial by treatment arm. Weekly numerator adjusted normative adherence was calculated and a mean by treatment arm was calculated and presented as a line graph for the first 25 weeks from randomisation.

Intervention adherence

The intervention comprised of:

(a) a chipped nebuliser to collect adherence data

(b) access for participants and interventionist to the adherence data summaries

(c) an online platform (CFHealthHub) offering summaries of adherence and tailored modules to be used by the health professional when interacting with the participant and independently by the participant.

A number of metrics were collected from CFHealthHub including the timing and date of clicks and the page/module that was clicked on. Interactions with CFHH were defined as a series of clicks with no greater that 15 minute gaps between clicks. Length of each session was calculated and days with interactions were calculated by participant.

The mean, standard deviation (SD), median and interquartile range (IQR) for the CFHH metrics were calculated and presented by participant. The same summary statistics were also presented for length of all sessions. The timing of CFHH interactions in days from randomisation was plotted by participant. The number of clicks per page category (Home, How am I doing?, Treatment etc) was plotted in a bar chart and also presented in a table by participant and by session.

Date and time of sessions with the interventionist were also recorded. The number of sessions with an interventionist and the length of sessions by participant were summarised in a table.

Clinic visits

The number of clinic visits completed by each participant excluding consent and 5 month follow up was recorded. Summary statistics were presented by treatment arm to assess whether ascertainment bias occurred in the intervention arm.

Safety analysis

The number of Adverse Events (AEs) and Serious Adverse Events (SAEs) was recorded and presented by treatment arm. These events were further categorised by the type of adverse event and whether they were related to the intervention.

Protocol non compliances

The number and type of protocol non compliances were presented descriptively.

Summary of missing data

The number of missing values or scores for each of the primary and secondary outcomes was presented by baseline and 5 (+/-1) months post randomisation and by treatment arm. Furthermore, the number and percentage of missing items was presented for each of these questionnaires.

Results

Participant Flow

Participants were recruited for 4 months across 2 sites. The CONSORT flow diagram (Fig.1) shows the flow of participants through the trial. 32 participants were randomised at each site. 33 participants were randomised to the intervention arm and 31 participants were randomised to usual care. A total of 59 participants completed the 5 (+/- 1) month follow up visit (Intervention = 31, Usual care = 28).

A total of 8 participants discontinued the trial before the follow up visit (Intervention = 4, Usual care = 4). Of these discontinuations, 5 no longer had their adherence data collected and the same 5 participants did not have their primary outcome collected. Of those who did not continue with primary outcome collection, 2 participants died, 1 withdrew consent and 2 were lost to follow up.

Following the 5 (+/-1) month visit, adherence data and primary outcome data was collected. 2 participants withdrew from adherence data collection during this time (Intervention =1, Usual care =1). 59 participants completed primary outcome data collection up to study completion on 30th April 2017 (Intervention = 31, Usual care =28).

Recruitment by centre and month

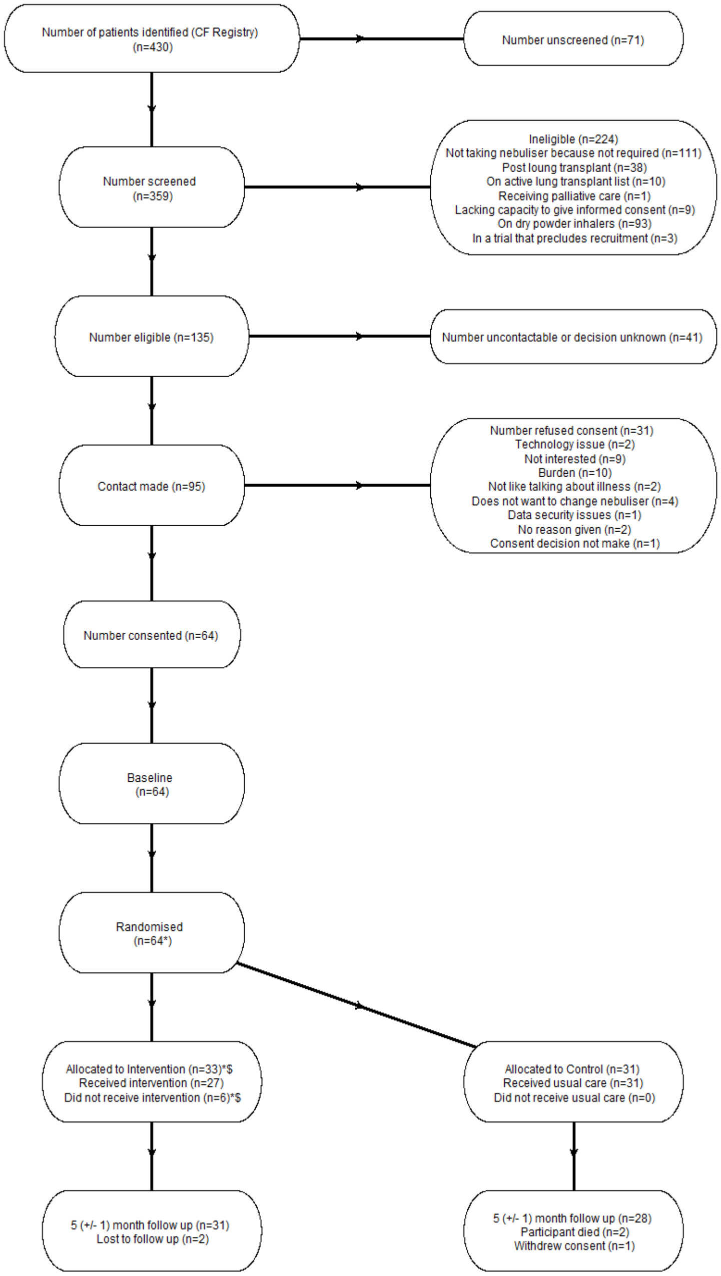

Figure 1: CONSORT flow diagram for ACtiF pilot study.

Table 1: Participants consented by centre and by month

	June 16	July 16	Aug 16	Sept 15	Total
Site A	4	16	7	5	32
Site B	2	17	5	8	32

Attrition by Centre and Treatment arm

Table 2: Attrition presented by treatment arm and site.

		n	Withdrew Consent (%)	Died (%)	Lost to Follow up (%)	Overall (%)
**Overall**		64	1(17%)	2(33%)	2(40%)	5(7.8%)
**Treatment arm**	Intervention	33	0(0%)	0(0%)	2(40%)	2(6.1%)
	Usual Care	31	1(20%)	2(40%)	0(0%)	3(9.7%)
**Site**	Site A	32	0(0%)	1(20%)	1(20%)	2(6.2%)
	Site B	32	1(20%)	1(20%)	1(20%)	3(9.4%)

Baseline characteristics

Table 4 shows the baseline characteristics of participants randomised by treatment arm. 33 participants were randomised to the intervention and 31 were randomised to usual care. The average age of participants was 29.7 (SD=11.5). Participants in the intervention arm were slightly older (median=28, IQR=(21,37)) than those in the usual care arm (median=26, IQR=(20,34)). Table 5 shows the CF measures presented by treatment arm. Tables 6-7 show the baseline questionnaire scores presented by treatment arm.

Baseline demographics

Table 3: Baseline demographics by treatment arm

	Intervention	Control	Overall
Age			
n	33	31	64
Mean(SD)	31.6(13.3)	27.8(8.9)	29.7(11.5)
Median(IQR)	28(21,37)	26(20,34)	27(21,36)
Min,Max	(16,69)	(16,50)	(16,69)
Sex			
Male	18(54.5%)	18(58.1%)	36(56.2%)
Female	15(45.5%)	13(41.9%)	28(43.8%)
Socioeconomic Status			
Most deprived	6(18.2%)	1(3.2%)	7(10.9%)
High deprivation	4(12.1%)	7(22.6%)	11(17.2%)
Average	8(24.2%)	8(25.8%)	16(25%)
Low deprivation	6(18.2%)	9(29%)	15(23.4%)
Least deprived	9(27.3%)	6(19.4%)	15(23.4%)
Weight (KG)			
n	33	31	64
Mean(SD)	65.5(18)	63.7(15.6)	64.6(16.8)
Median(IQR)	63(53,76)	62.9(49,74)	63(52.9,74.3)
Min,Max	(35,128)	(35.6,103.7)	(35,128)
Height (cm)			
n	33	31	64
Mean(SD)	168.6(10.5)	167.7(9.6)	168.2(10)
Median(IQR)	170(162,177)	168(159,175)	168.5(160.5,175.5)
Min,Max	(147,193)	(149,186)	(147,193)
BMI			
n	33	31	64
Mean(SD)	22.8(5)	22.4(4.3)	22.6(4.6)

Median(IQR)	22.2(19.7,25.3)	22.1(19.1,25.4)	22.1(19.55,25.35)
Min,Max	(15.8,42.8)	(16,33.9)	(15.8,42.8)

Table 4: Baseline CF measures by treatment arm

	Intervention	Control	Overall
No. of IV days in previous 12 months			
n	33	31	64
Mean(SD)	26.3(25.7)	26(22.1)	26.2(23.8)
Median(IQR)	17(7,44)	28(0,44)	17(7,44)
Min,Max	(0,117)	(0,70)	(0,117)
No. of participants requiring IV days in previous 12 months			
At least 1 IV day	26(78.8%)	23(74.2%)	49(76.6%)
Days since last IV start date			
n	31	28	59
Mean(SD)	168.7(245.2)	202.3(325.2)	184.6(283.9)
Median(IQR)	75(45,194)	100(24.5,219.5)	91(39,213)
Min,Max	(6,1085)	(7,1575)	(6,1575)
FEV1			
n	33	31	64
Mean(SD)	2(0.8)	2.3(1)	2.1(0.9)
Median(IQR)	1.9(1.4,2.4)	2.1(1.6,2.8)	1.9(1.5,2.7)
Min,Max	(0.8,4)	(0.6,5)	(0.6,5)
FEV1 % Predicted			
n	33	31	64
Mean(SD)	53.4(19.4)	61.4(22.7)	57.3(21.3)
Median(IQR)	49.2(39.4,61.9)	53.4(43,80)	49.6(41.9,76.7)
Min,Max	(26,103)	(23.2,100.7)	(23.2,103)
Clinician pseudomonas status			
Negative	15(45.5%)	8(26.7%)	23(36.5%)
Intermittent	3(9.1%)	3(10%)	6(9.5%)
Chronic	15(45.5%)	19(63.3%)	34(54%)
Leeds Criteria pseudomonas status			
Negative	15(45.5%)	10(33.3%)	25(39.7%)
Intermittent	4(12.1%)	4(13.3%)	8(12.7%)
Chronic	14(42.4%)	16(53.3%)	30(47.6%)

Subjective adherence			
n	23	20	43
Mean(SD)	65.6(40.1)	67.8(35.4)	66.6(37.6)
Median(IQR)	90(20,99)	80(45,99.5)	90(35,99)
Min,Max	(0,100)	(0,100)	(0,100)
Simple normative adherence (first 2 weeks)			
n	33	31	64
Mean(SD)	0.5(0)	0.5(0)	0.5(0)
Median(IQR)	0.5(0.5,0.5)	0.5(0.5,0.5)	0.5(0.5,0.5)
Min,Max	(0.5,0.5)	(0.5,0.5)	(0.5,0.5)
Treatment Burden			
Low	10(30.3%)	11(35.5%)	21(32.8%)
Medium	16(48.5%)	12(38.7%)	28(43.8%)
High	2(6.1%)	5(16.1%)	7(10.9%)

Baseline outcome measures

Table 5: Baseline outcome measures by treatment arm

	Intervention	Control	Overall
EQ5D-5L			
n	33	31	64
Mean(SD)	0.866(0.121)	0.822(0.151)	0.845(0.137)
Median(IQR)	0.901(0.767,0.951)	0.825(0.737,0.942)	0.872(0.752,0.946)
Min,Max	(0.53,1)	(0.486,1)	(0.486,1)
PAM-13			
n	33	31	64
Mean(SD)	60.4(11.2)	60(13.2)	60.2(12.1)
Median(IQR)	60.6(53.2,67.8)	58.1(48.9,67.8)	60.6(51,67.8)
Min,Max	(36.8,84.8)	(38.1,90.7)	(36.8,90.7)
CHAOS			
n	33	31	64
Mean(SD)	9.8(3.4)	10.1(4)	10(3.7)
Median(IQR)	10(8,11)	10(7,12)	10(8,11)
Min,Max	(4,18)	(4,20)	(4,20)
MAD-3			
n	32	30	62
Mean(SD)	9.8(3.3)	9(3.4)	9.4(3.4)

Median(IQR)	9(8,12.5)	9.5(6,11)	9(8,12)
Min,Max	(3,15)	(3,15)	(3,15)
SRBAI			
n	33	30	63
Mean(SD)	11.5(4.9)	10.2(5.6)	10.9(5.2)
Median(IQR)	12(8,16)	9(4,14)	10(7,15)
Min,Max	(4,20)	(4,20)	(4,20)
GAD-7			
n	33	31	64
Mean(SD)	4.1(4.5)	3.8(3.6)	3.9(4)
Median(IQR)	3(0,5)	3(1,7)	3(0.5,5.5)
Min,Max	(0,15)	(0,11)	(0,15)
PHQ-8			
n	33	31	64
Mean(SD)	7(4.9)	6.5(5.2)	6.8(5)
Median(IQR)	6(3,12)	6(3,8)	6(3,10.5)
Min,Max	(0,16)	(0,18)	(0,18)

Table 6: Baseline CFQR domains by treatment arm

	Intervention	Control	Overall
Physical Functioning			
n	33	31	64
Mean(SD)	48.5(34.8)	49.2(30.8)	48.9(32.7)
Median(IQR)	38(25,88)	42(17,83)	42(21,85.5)
Min,Max	(0,100)	(0,100)	(0,100)
Emotional Functioning			
n	33	31	64
Mean(SD)	70.2(21.1)	62.3(26.1)	66.4(23.8)
Median(IQR)	67(53,93)	67(40,80)	67(53,87)
Min,Max	(27,100)	(7,100)	(7,100)
Eating			
n	33	31	64
Mean(SD)	79.9(24.8)	74.6(27.7)	77.3(26.2)
Median(IQR)	89(67,100)	78(56,100)	89(61.5,100)
Min,Max	(0,100)	(0,100)	(0,100)
Social Functioning			
n	33	31	64

Mean(SD)	65(20.3)	59.6(26.2)	62.4(23.3)
Median(IQR)	67(50,78)	61(44,83)	67(44,83)
Min,Max	(17,100)	(11,100)	(11,100)
Body Image			
n	33	31	64
Mean(SD)	68.5(27.3)	64.9(31.7)	66.7(29.3)
Median(IQR)	78(56,89)	67(44,100)	78(44,89)
Min,Max	(0,100)	(0,100)	(0,100)
Treatment Burden			
n	33	31	64
Mean(SD)	50.5(16.5)	51.6(25.9)	51(21.4)
Median(IQR)	44(44,67)	56(33,67)	50(44,67)
Min,Max	(11,78)	(0,100)	(0,100)
Respiratory			
n	33	31	64
Mean(SD)	53.5(27.5)	54(27.3)	53.7(27.2)
Median(IQR)	50(33,78)	56(33,78)	56(33,78)
Min,Max	(0,100)	(6,100)	(0,100)
Digestion			
n	33	31	64
Mean(SD)	77.9(16.9)	80.4(26.4)	79.1(21.9)
Median(IQR)	78(67,89)	89(78,100)	89(67,100)
Min,Max	(44,100)	(0,100)	(0,100)
Role Functioning			
n	33	31	64
Mean(SD)	65.2(24.3)	64(25.9)	64.6(24.9)
Median(IQR)	67(50,83)	67(42,83)	67(50,83)
Min,Max	(0,100)	(8,100)	(0,100)
Vitality			
n	33	31	64
Mean(SD)	37.8(22.8)	40.6(22)	39.2(22.3)
Median(IQR)	33(17,50)	42(25,58)	42(25,58)
Min,Max	(8,92)	(0,75)	(0,92)
Health Perceptions			
n	33	31	64
Mean(SD)	47.8(27.7)	51.6(24.9)	49.6(26.3)
Median(IQR)	44(22,67)	56(33,67)	44(33,67)

Min,Max	(0,100)	(0,100)	(0,100)
Weight			
n	33	31	64
Mean(SD)	70.7(36.1)	63.4(39.8)	67.2(37.9)
Median(IQR)	100(33,100)	67(33,100)	83.5(33,100)
Min,Max	(0,100)	(0,100)	(0,100)

Table 7: Baseline COM-BMQ domains by treatment arm

	Intervention	Control	Overall
COM BMQ Necessities			
n	33	31	64
Mean(SD)	3.2(0.7)	3.4(0.8)	3.3(0.8)
Median(IQR)	3.1(2.7,3.7)	3.3(2.9,4.1)	3.1(2.7,4)
Min,Max	(2,4.9)	(2,4.7)	(2,4.9)
COM BMQ Concerns			
n	33	31	64
Mean(SD)	2.1(0.6)	2.2(0.6)	2.1(0.6)
Median(IQR)	2.1(1.5,2.6)	2.1(1.7,2.6)	2.1(1.6,2.6)
Min,Max	(1.2,3.4)	(1.1,3.3)	(1.1,3.4)

Primary Analysis

- In total, there were 79 exacerbations in participants followed up for at least 6 months
- Of these, 60 exacerbations fitted our criteria to be included in the primary analysis
  - 18 were not treated with IV antibiotics
  - 1 did not meet any Fuchs criteria
- A total of 60 participants had at least 6 months of exacerbation data (Intervention=32, Control =28)
- 4 participants were excluded
  - 2 died (Control=2)
  - 1 withdrew consent (Control=1)
  - 1 lost to follow up before 6 months (Intervention=1)
- 35 exacerbations occurred in Intervention participants, 25 occurred in Control participants
- 33 participants experienced at least 1 exacerbation (Intervention= 19 (60%), Control= 14 (50%))

The most frequently reported Fuchs criteria (Table 9) were 'Increased cough' (n=52) and 'Change in sputum (n=48). The median number of Fuchs criteria reported per exacerbation included in the primary analysis was 4 (IQR=4,6).

The median IV course length of exacerbations included in the primary analysis was 14 days in both the intervention and usual care arm (Table 12).

As ACtiF was a pilot study, it was not powered to detect an intervention effect. However, differences between treatment arms and their 95% confidence intervals have been calculated (Table 13). The median number of exacerbations was 1 in the intervention arm and 0.5 in the usual care arm. Following adjustment for site and the number of IV days in the previous year, adjusted IRR was 1.12 (95% CI: 0.658-1.94). This demonstrates a small increase in exacerbations in the intervention arm, however the confidence intervals are relatively wide. The IRR from the offset model shows an IRR of 0.958 (95% CI: 0.615,1.5). Here, a small decrease in exacerbations can be observed. As with the previous model, the confidence interval is relatively wide.

Exacerbations summary

Number of Exacerbations

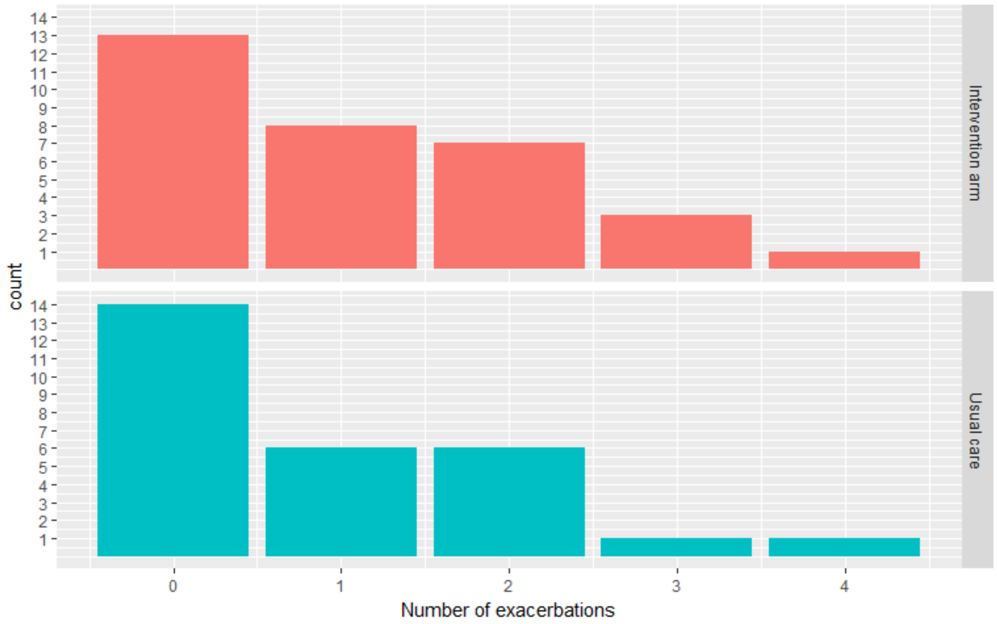

Figure 2:The number of exacerbations in participants by treatment arm in 6 months [n=60]

Fuchs Criteria

Table 8:The number of each Fuchs criterion in the exacerbations used as the primary outcome

	n (%) for exacerbations in 6 months after consent and meeting our criteria (primary outcome)	n (%) for exacerbations treated with IV antibiotics and met at least one Fuchs criteria	n (%) for any exacerbation during the study
**Change in sputum**	48 (80 %)	63 (77.8 %)	69 (69 %)
**New or increased hemoptysis**	12 (20 %)	15 (18.5 %)	16 (16 %)
**Increased cough**	52 (86.7 %)	70 (86.4 %)	77 (77 %)
**Increased dyspnea**	43 (71.7 %)	56 (69.1 %)	61 (61 %)
**Malaise, fatigue, or lethargy**	48 (80 %)	66 (81.5 %)	69 (69 %)
**Temperature above 38 °C**	13 (21.7 %)	18 (22.2 %)	20 (20 %)
**Anorexia or weight loss**	20 (33.3 %)	30 (37 %)	31 (31 %)
**Sinus pain or tenderness**	13 (21.7 %)	19 (23.5 %)	21 (21 %)
**Change in sinus discharge**	13 (21.7 %)	21 (25.9 %)	22 (22 %)
**Change in physical examination of the chest, derived from notes by site staff.**	9 (15 %)	12 (14.8 %)	13 (13 %)
**Decrease in pulmonary function by 10 percent or more from a previously recorded value, derived from notes by site staff**	12 (20 %)	17 (21 %)	19 (19 %)
**Radiographic changes indicative of pulmonary infection, derived from notes by site staff)**	2 (3.3 %)	2 (2.5 %)	2 (2 %)

Table 9:Summary of Fuchs criteria for the exacerbations that were included in the primary outcome (IV days and at least 1 Fuchs criteria in 6 month follow up period

Description	
Exacerbations included in primary analysis	
n (%) with IV and at least 1 Fuchs	60 (60 %)
Mean (SD) number of Fuchs criteria	4.8 (2.1)
Median (IQR) number of Fuchs criteria	4 (4 , 6)
Min, max number of Fuchs criteria	(1,10)
n (%) of exacerbations with at least 2 Fuchs criteria	58 (96.7 %)
n (%) of exacerbations with at least 3 Fuchs criteria	48 (80 %)
n (%) of exacerbations with at least 4 Fuchs criteria	46 (76.7 %)
n (%) of exacerbations with at least 5 Fuchs criteria	29 (48.3 %)
n (%) of exacerbations with at least 6 Fuchs criteria	20 (33.3 %)
n (%) of exacerbations with at least 7 Fuchs criteria	12 (20 %)
n (%) of exacerbations with at least 8 Fuchs criteria	8 (13.3 %)
n (%) of exacerbations with at least 9 Fuchs criteria	3 (5 %)
n (%) of exacerbations with at least 10 Fuchs criteria	1 (1.7 %)

Table 10:Summary of the exacerbations in the 6 month follow up period that were not included in the primary outcome (IV days and at least 1 Fuchs criteria) and the reasons for exclusion

Exacerbations in 6 months not meeting criteria for primary outcome	
Total exacerbations excluded	19 (24 %)
n (%) with IV days but no Fuchs criteria met	1 (1 %)
n (%) with no IV but at least 1 Fuchs	7 (8 %)
n (%) no IV days or Fuchs recorded (missing values)	11 (14 %)

Length of IV course

Table 11:Summary of IV length by exacerbation and participant

	Intervention	Usual Care
IV days per exacerbation in 6 months		
n	35	25
Mean (SD)	13.6(4.2)	13.7(3.3)
Median (IQR)	14(13,14)	14(13,15)
Min, Max	(2,30)	(7,21)
IV days per participant with exacerbations in 6 months		
n	19	14
Mean (SD)	13.4(2.7)	13.6(3.2)
Median (IQR)	14(11,14)	14(13,15)
Min, Max	(9,21.7)	(8,20)
IV days per exacerbation in whole study		
n	45	36
Mean (SD)	13.7(4.1)	13.9(3.1)
Median (IQR)	14(13,14)	14(13,15)
Min, Max	(2,30)	(7,21)

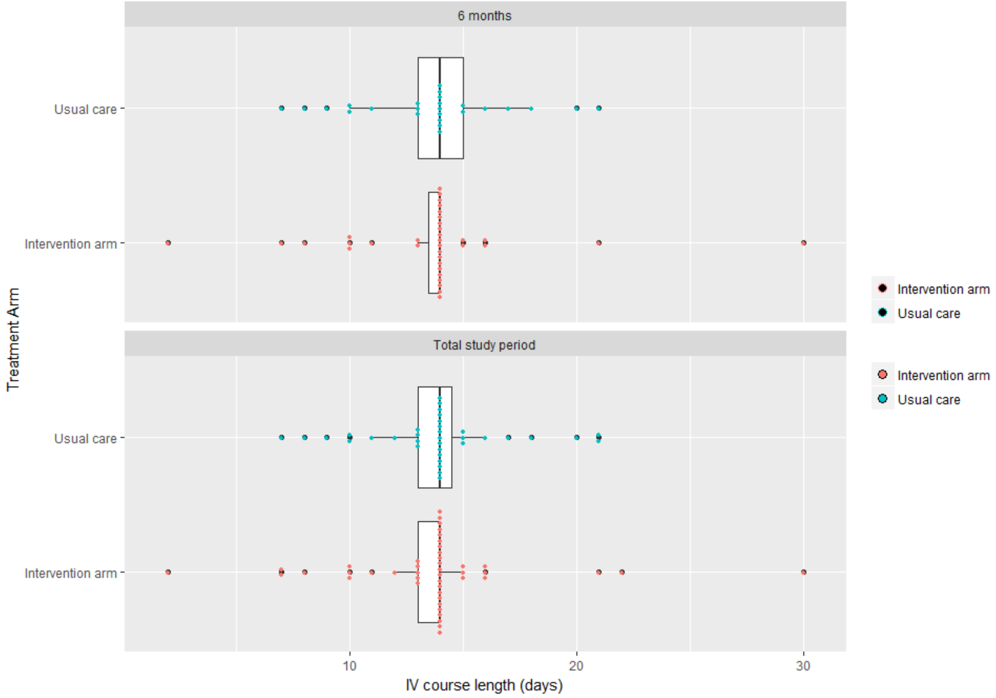

Figure 3:The length on IV courses by treatment arm

Analysis models

6 month model

Table 12:Analysis of the primary clinical outcome, the number of exacerbations treated with IV antibiotics with at least 1 Fuchs criteria in a 6 month period adjusted for site and the number of IV days in the previous year.

	Intervention n	Mean (SD)	Median (IQR)	Control n	Mean (SD)	Median (IQR)	IRR	95% CI
Unadjusted	32	1.1 (1.1)	1 (0 , 2)	28	0.9 (1.1)	0.5 (0 , 2)	1.22	(0.686,2.21)
Adjusted							1.12	(0.658,1.94)

Offset model

Table 13:A sensitivity analysis using all exacerbations treated with IV antibiotics with at least 1 Fuchs criteria that occurred during the study with the number of days of data collection included as an offset in the model. Adjusted for site and number of IV days in the previous year

	Intervention n	Total exacerbations (min,max)	Mean (SD) days followed up	Mean (SD) exacerbations per month	Control n	Total exacerbations (min,max)	Mean (SD) days followed up	Mean (SD) exacerbations per month	IRR	95% CI
**Adjusted, Offset model**	33	46(0,5)	263.2(47.2)	0.17(0.16)	31	40(0,5)	250.5(74.8)	0.2(0.28)	0.958	(0.615,1.5)

Secondary analysis

Tables 15-16 show the results of the secondary analyses. As this is a pilot study, we have not powered to detect any effect. Key results are described below.

- Adjusted mean difference of 5% (95% CI: -2-12%) in FEV % predicted. This is an encouraging difference in the intervention arm.
- No notable differences in any of the other secondary outcomes but this is not of great concern as it is a pilot study.
- Fewer participants had BMI recorded than other outcomes (Intervention=18, Control=15).
- Small reduction in BMQ Concerns score in intervention arm (Mean difference=-0.21, 95% CI: -0.38,-0.048).

Figure 4 shows the distribution of the secondary outcome measures at baseline and follow up by treatment arm.

Table 14:Results of secondary effectiveness analysis

	n Intervention	Median (IQR)	Mean (SD)	n Control	Median (IQR)	Mean (SD)	Mean Diff	95% CI
FEV1 Unadjusted	30	1.8(1.17,2.83)	2(0.9)	27	1.9(1.46,2.83)	2.2(1)	-0.21	(-0.73,0.3)
FEV1 Adjusted							0.22	(-0.062,0.51)
FEV1 % Unadjusted	30	51.8(33.46,71.26)	54.2(21.1)	27	50.9(42.49,77.97)	59(23.9)	-4.8	(-17,7.1)
FEV1 % Adjusted							5	(-2,12)
BMI Unadjusted	18	20.5(19.5,26)	22.1(4.2)	15	23.4(20.7,26.2)	23.8(3.5)	-1.7	(-4.5,1.1)
BMI Adjusted							-0.08	(-1,0.89)
EQ5D-5L Unadjusted	31	0.9(0.76,0.95)	0.9(0.2)	27	0.9(0.77,1)	0.9(0.2)	-0.00062	(-0.084,0.083)
EQ5D-5L Adjusted							-0.016	(-0.087,0.055)

PAM-13 Unadjusted	31	63.1(51,67.8)	58.5(14.3)	28	58.1(51,63.1)	57.9(9.9)	0.56	(-5.9,7)
PAM-13 Adjusted							0.046	(-5.8,5.9)
CHAOS Unadjusted	31	9(7,13)	9.9(3.9)	28	9(7.5,11.5)	9.4(3.3)	0.55	(-1.4,2.4)
CHAOS Adjusted							0.79	(-0.47,2.1)
MAD-3 Unadjusted	31	12(9,13)	10.8(3.9)	26	9.5(7,13)	9.4(3.6)	1.4	(-0.58,3.4)
MAD-3 Adjusted							0.82	(-0.51,2.1)
SRBAI Unadjusted	31	13(8,16)	12.1(5.3)	28	10.5(6,15.5)	10.6(5)	1.4	(-1.3,4.1)
SRBAI Adjusted							0.15	(-1.8,2.1)
GAD-7 Unadjusted	31	3(1,6)	4.1(4.1)	28	2.5(0,7)	4.2(4.4)	-0.05	(-2.3,2.2)
GAD-7 Adjusted							-0.31	(-1.9,1.3)
PHQ-8 Unadjusted	31	7(4,12)	7.3(5.2)	28	4(1.5,7)	5.3(5.1)	2	(-0.68,4.7)
PHQ-8 Adjusted							0.97	(-0.96,2.9)
COM-BMQ Concerns Unadjusted	31	2(1.5,2.3)	1.9(0.5)	27	2.1(1.9,2.4)	2.1(0.5)	-0.22	(-0.48,0.026)
COM-BMQ Concerns Adjusted							-0.21	(-0.38,-0.048)
COM BMQ Necessities Unadjusted	31	3.4(3,4)	3.5(0.6)	27	3.4(2.9,4)	3.5(0.7)	0.011	(-0.35,0.37)

COM BMQ Necessities Adjusted	0.12	(-0.16,0.4)

Table 15:Results of secondary effectiveness analysis

	n Intervention	Median (IQR)	Mean (SD)	n Control	Median (IQR)	Mean (SD)	Mean Diff	95% CI
CFQ-R Physical Unadjusted	31	54(25,88)	54.4(31.6)	28	62.5(33,92)	60.9(31.2)	-6.4	(-23,10)
CFQ-R Physical Adjusted							-2.6	(-13,7.4)
CFQ-R Emotional State Unadjusted	31	67(53,93)	68.3(23.4)	28	73(56.5,90)	72.3(22.7)	-4	(-16,8)
CFQ-R Emotional State Adjusted							-7.7	(-16,0.55)
CFQ-R Eating Unadjusted	31	89(67,100)	80.7(21.6)	28	83.5(67,100)	79.9(20.7)	0.85	(-10,12)
CFQ-R Eating Adjusted							1.1	(-6.5,8.7)
CFQ-R Social Unadjusted	31	67(56,78)	65.4(15.8)	28	64(50,83)	66.4(20.9)	-1	(-11,8.6)
CFQ-R Social Adjusted							-3.7	(-10,2.8)
CFQ-R Body Image Unadjusted	31	78(67,89)	73.3(23.8)	28	78(56,100)	73.1(25.5)	0.19	(-13,13)
CFQ-R Body Image Adjusted							0.62	(-7.2,8.5)
CFQ-R Treatment Burden Unadjusted	31	56(44,67)	56.5(16.6)	28	56(44,67)	57.3(19.9)	-0.83	(-10,8.7)
CFQ-R Treatment Burden Adjusted							1.2	(-6.4,8.8)
CFQ-R Respiratory Unadjusted	31	67(44,78)	59.5(25.2)	27	67(50,83)	65.6(22.7)	-6.1	(-19,6.6)
CFQ-R Respiratory Adjusted							-4.4	(-14,4.8)

CFQ-R Digestion Unadjusted	31	89(67,100)	81.1(18.4)	27	89(78,100)	84.4(23.5)	-3.3	(-14,7.7)
CFQ-R Digestion Adjusted							-2.3	(-11,6.2)
CFQ-R Role Unadjusted	31	75(33,83)	64.8(26.1)	27	75(56,92)	70.3(21.5)	-5.6	(-18,7.1)
CFQ-R Role Adjusted							-8.2	(-17,0.4)
CFQ-R Vital Unadjusted	31	42(25,42)	38.5(19.5)	28	50(33,62.5)	48.7(23)	-10	(-21,0.81)
CFQ-R Vital Adjusted							-7	(-15,0.99)
CFQ-R Health Unadjusted	31	44(22,67)	45.5(25.4)	28	61.5(33,72.5)	56.8(27.6)	-11	(-25,2.6)
CFQ-R Health Adjusted							-6.5	(-16,2.8)
CFQ-R Weight Unadjusted	31	89(67,100)	81.1(18.4)	27	89(78,100)	84.4(23.5)	-3.3	(-14,7.7)
CFQ-R Weight Adjusted							-2.3	(-11,6.2)

BMJ Publishing Group Limited (BMJ) disclaims all liability and responsibility arising from any reliance placed on this supplemental material which has been supplied by the author(s)

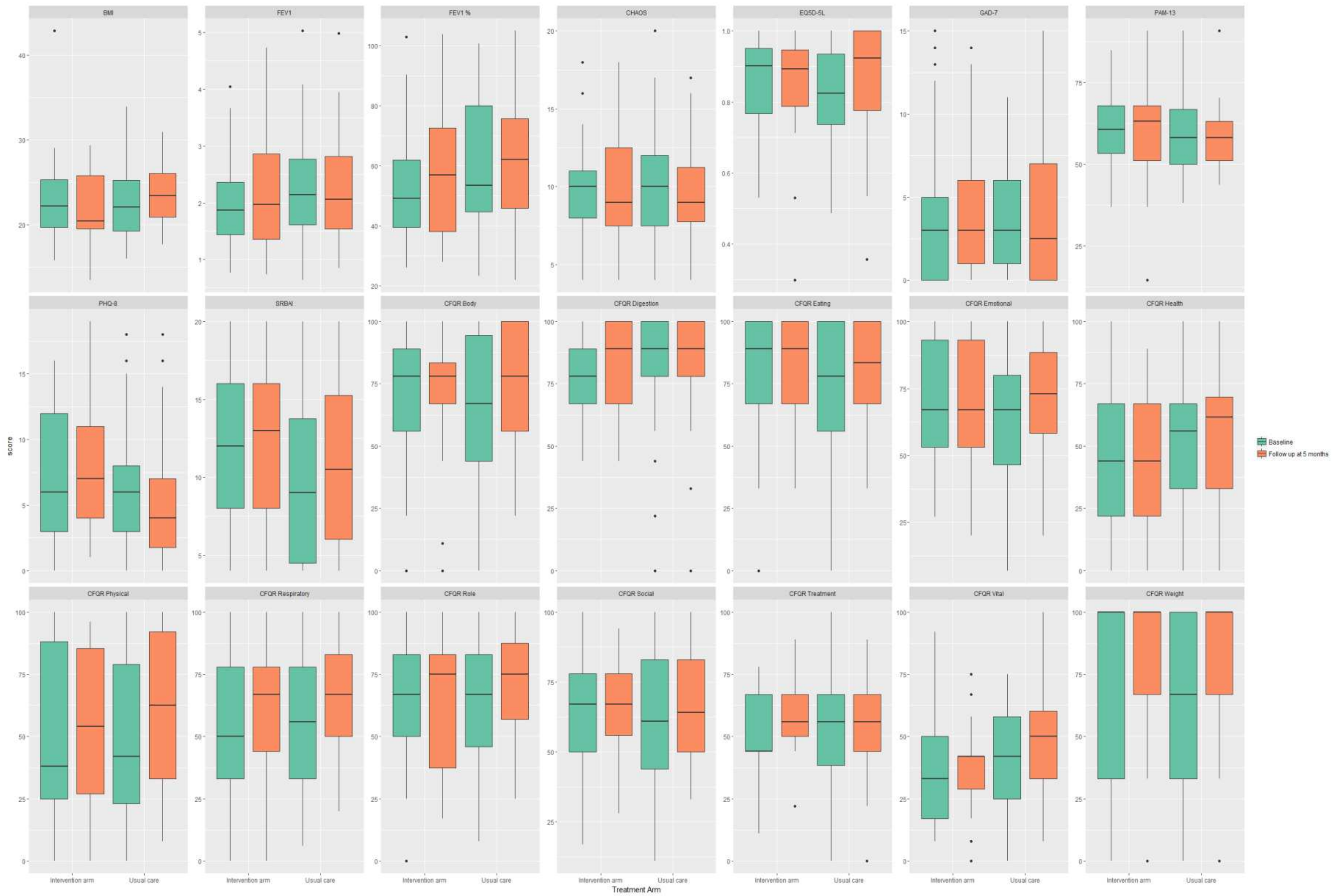

Figure 4:Box plots showing the distribution of secondary outcomes by treatment arm

Adherence to CF medication

During the trial, 8 participants withdrew from adherence data collection (Intervention=4, Control=4). An exact date of withdrawal was not recorded but could be seen from inhalation data (last non zero number of daily inhalations). This has been improved for the main trial and date of adherence data collection withdrawal will be recorded.

Participants who withdrew from adherence data collection were removed from summaries of adherence for 6 months as they did not have 6 months' worth of data. Where possible, inhalation data collected before withdrawal was included in the mean adherence by arm in the monthly table and the plot by week. The number included in each of these estimates can be seen in Table 18.

Table 17 shows the mean adherence by treatment arm for the 6 months post randomisation. Adherence is greater in the intervention arm for each of the different adherence measures. A difference of 10% (95% CI: -5.2 to 25.2) in simple normative adherence with numerator adjustment can be observed in the intervention arm. Table 18 shows the difference in simple normative adherence with numerator adjustment by treatment arm for each individual month in the study. Adherence is greater in the Intervention arm in month 1 (mean difference=2.6, 95% CI: -13.5,18.6). Following month 1, adherence is consistently higher in the intervention arm with the greatest difference observed in month 5 (mean difference: 13%, 95% CI: -4.8, 30.8). These differences would indicate a potentially clinically important difference between the intervention and usual care arms.

The difference in adherence has been presented by weeks post randomisation in Figure 5. There is a difference in numerator adjusted normative adherence with greater adherence observed in the intervention arm. This difference becomes clear after week 4 which coincides with use of the intervention around week 2-3.

Table 16:Summary of average adherences in 6 months following consent by intervention arm and the difference in means with 95% confidence intervals

	n Intervention	Mean Intervention	n Control	Mean Control	Mean Difference (95% CI)
Baseline (first 2 weeks)	29	25.9(31.4)	26	23.2(29)	2.6(-13.9,19.2)
Total doses	29	222.4(233.1)	26	245.7(238.6)	-23.3(-151.2,104.6)
Unadjusted adherence	29	47.7(33.8)	26	37.7(27.1)	10(-6.5,26.4)
Simple normative	29	45.5(32.8)	26	34.7(27)	10.8(-5.4,27)

Sophisticated normative	29	41.6(33.4)	26	34.2(27.1)	7.5(-8.9,23.9)
Simple normative with numerator adjustment	29	43.6(30.4)	26	33.6(25.9)	10(-5.2,25.2)
Sophisticated normative with numerator adjustment	29	39.9(30.9)	26	33.2(25.9)	6.8(-8.6,22.2)

Table 17:Summary of average adherences in each month from following consent from 1 to 6 months by intervention arm

	n Intervention	Mean Intervention	n Control	Mean Control	Mean Difference (95% CI)
Month 1	32	29.7(34.5)	28	27.2(27.5)	2.6(-13.5,18.6)
Month 2	31	42.1(33.1)	28	33.7(31.5)	8.4(-8.5,25.2)
Month 3	30	42.3(33.7)	28	33.3(34.8)	9(-9,27.1)
Month 4	29	42.7(34.7)	27	34.5(30.5)	8.2(-9.3,25.7)
Month 5	29	42.8(36.2)	27	29.8(30.1)	13(-4.8,30.8)
Month 6	29	41.3(36.5)	27	32.9(28.5)	8.4(-9.1,25.9)

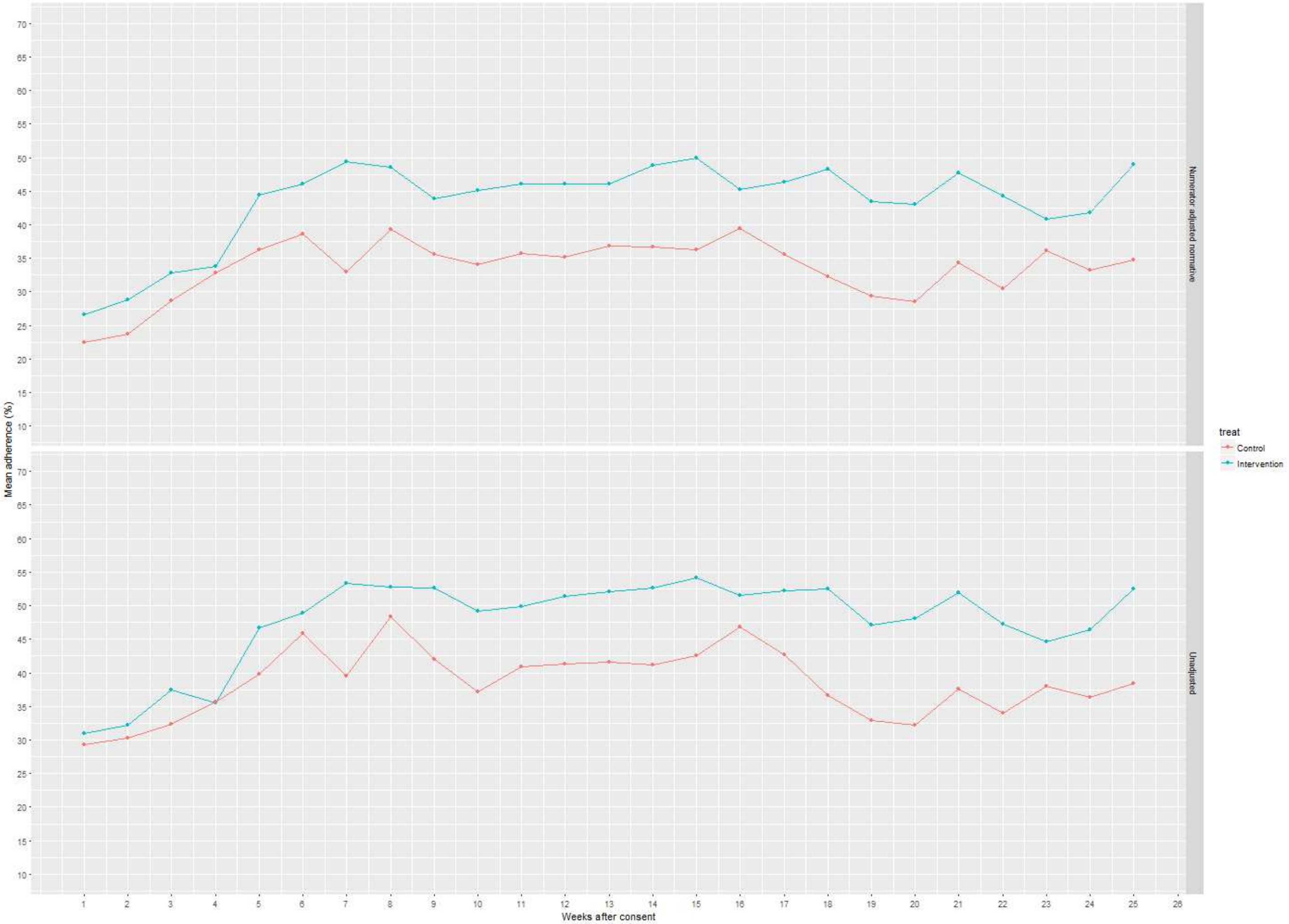

Figure 5:Mean weekly adherence by treatment arm

Intervention adherence (Participants)

Table 19 shows the median number of CFHH interactions was 3 (IQR: 1-8). 3 participants had no interactions with CFHH and the maximum number of interactions was 44. The mean total duration of interaction time across the study was 49.3 (SD= 44.8) minutes. The mean length of an interaction by participant was 12.4 (SD=9.6) minutes and the mean length of all interactions was 6.6 (SD=11) minutes. The median number of days in the trial with interactions was 2 (IQR=1,7) by participant. Figure 6 shows the wide range of values across participants, particularly for the total duration of interactions.

Figure 7 shows when interactions occurred in days for each participant. Some participants were interacting fairly regularly, however most participants were inconsistent with their interactions. Figure 8 shows that the 'How am I doing?' pages were the most frequently visited in terms of the total number of clicks during the trial. 30 (90.9%) of participants visited the 'How am I doing?', 'Treatment' and 'Videos' page at least once (Table 20). 224 (91.4%) sessions included a visit to the 'How am I doing?' page.

Table 18:Summary of clicks in CFHH. An interaction is defined as a series of clicks with no greater than a 15 minute lag between clicks

Interactions with CFHH by participant	
n	33
Mean (SD)	7.4(11.6)
Median (IQR)	3(1,8)
Min, Max	(0,44)
Total duration of interactions by participant	
n	33
Mean (SD)	49.3(44.8)
Median (IQR)	38(26,55)
Min, Max	(0,177)
Mean duration of interactions by participant	
n	33
Mean (SD)	12.4(9.6)
Median (IQR)	10.7(4.3,19)
Min, Max	(0,37)
Days with interactions by participant	
n	33
Mean (SD)	5.7(8.2)
Median (IQR)	2(1,7)
Min, Max	(0,32)
Duration of interactions	
n	245
Mean (SD)	6.6(11)
Median (IQR)	1(0,8)
Min, Max	(0,57)

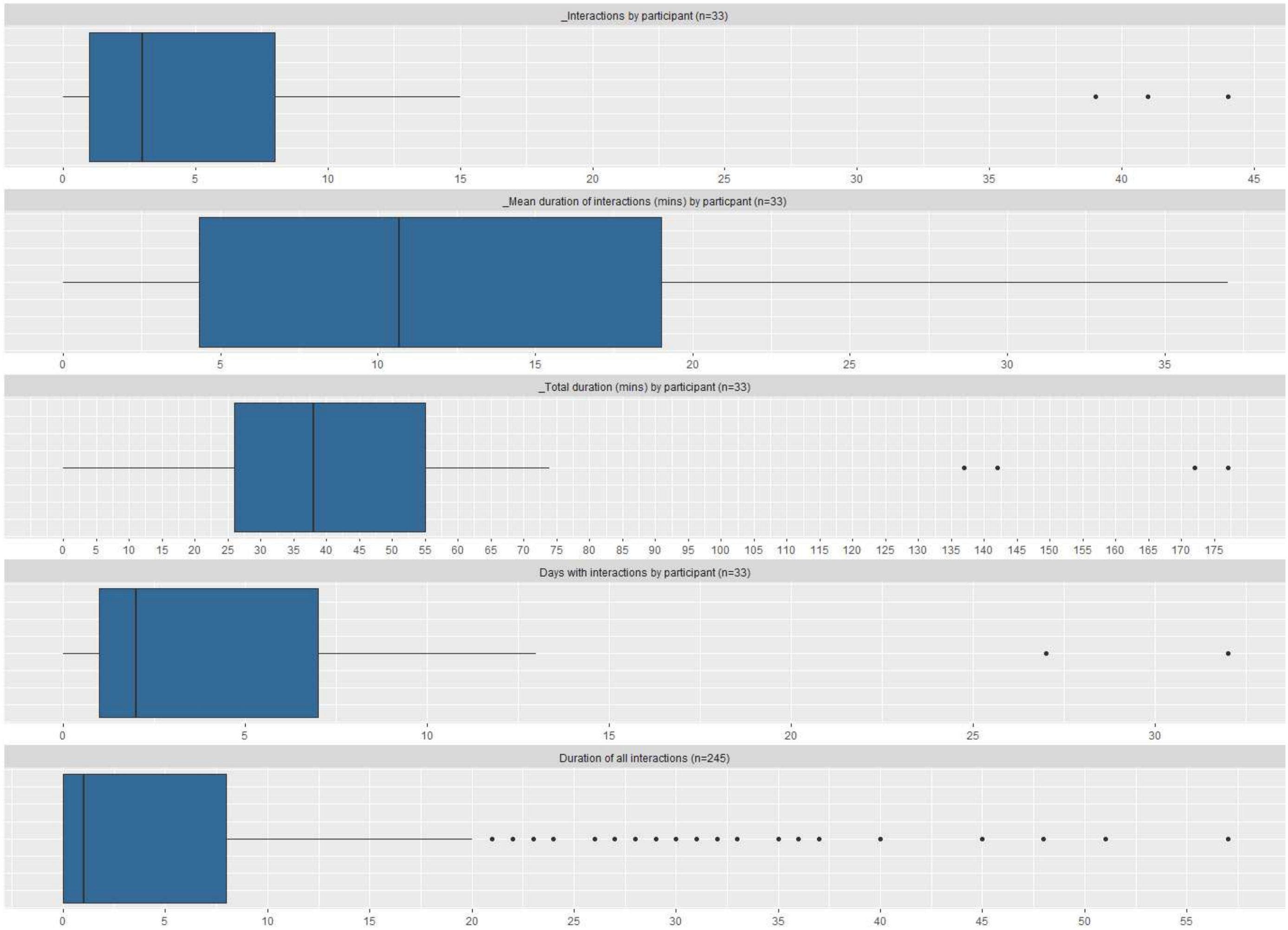
_Interactions by participant (n=33)
_Mean duration of interactions (mins) by particpant (n=33)
_Total duration (mins) by participant (n=33)
Days with interactions by participant (n=33)
Duration of all interactions (n=245)

Figure 6:Boxplots showing summaries of click analytics in CFHH

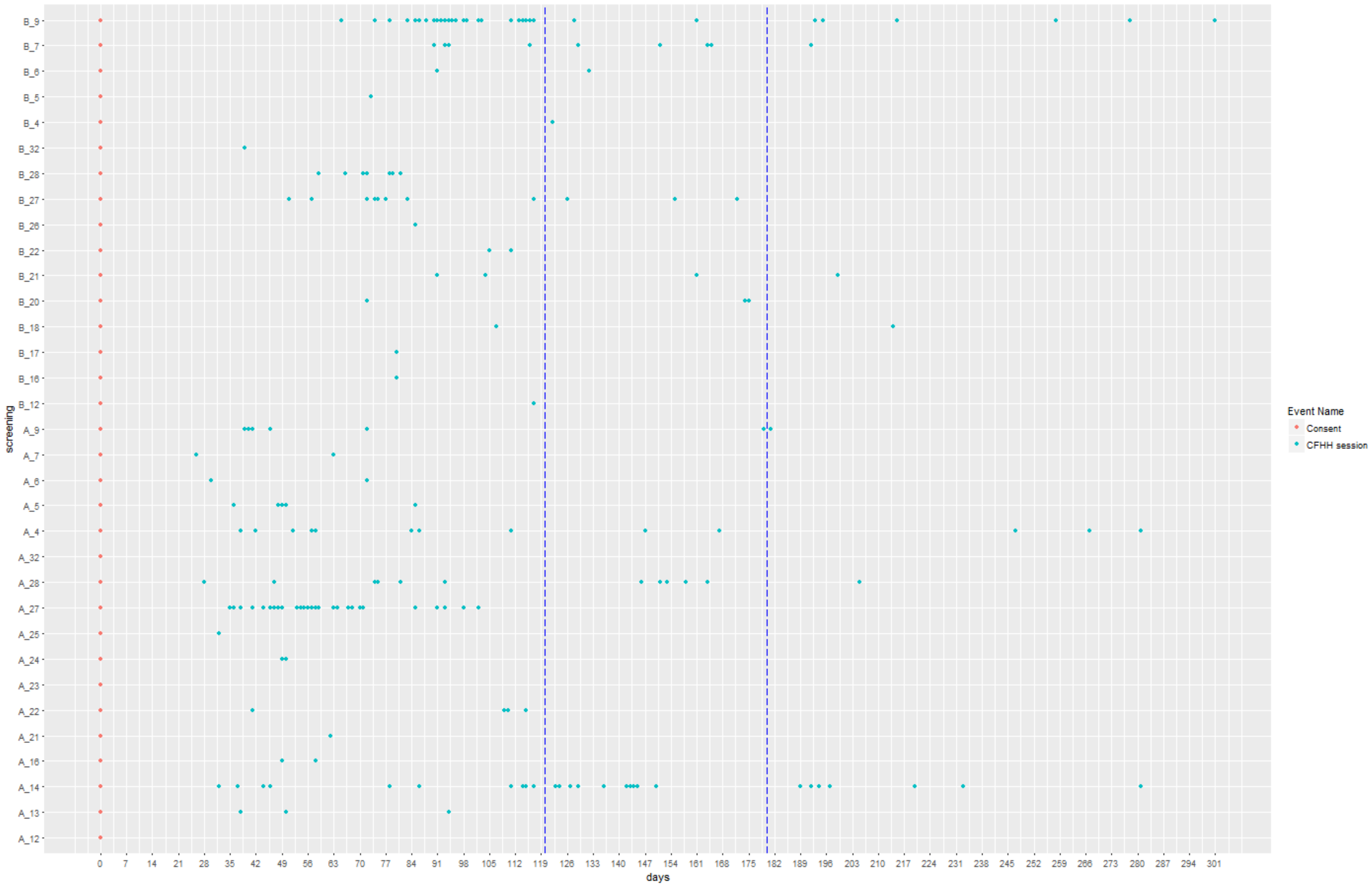

Figure 7:Timing in days of interactions with CFHH

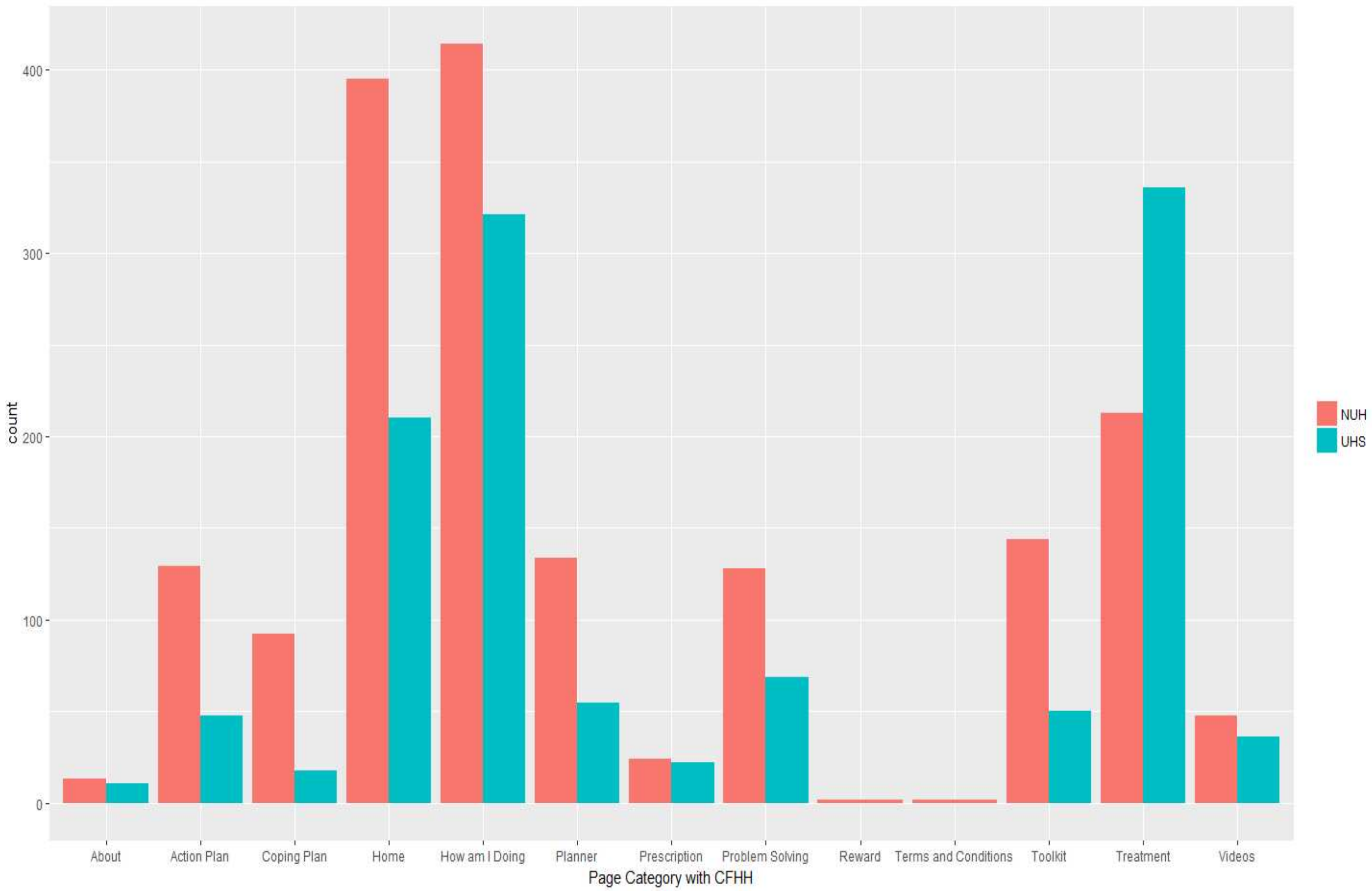

Figure 8:Frequency of clicks by CFHH categories

Table 19:Summary of clicks by page categories in CFHH

	Total (%) clicks	Participants (%) with at least one click	Sessions (%) with at least one click
**About**	24(0.8%)	13(39.4%)	20(8.2%)
**Action Plan**	177(6.1%)	28(84.8%)	53(21.6%)
**Coping Plan**	110(3.8%)	24(72.7%)	38(15.5%)
**Home**	605(20.8%)	30(90.9%)	244(99.6%)
**How am I Doing**	735(25.2%)	30(90.9%)	224(91.4%)
**Planner**	189(6.5%)	21(63.6%)	39(15.9%)
**Prescription**	46(1.6%)	22(66.7%)	42(17.1%)
**Problem Solving**	197(6.8%)	24(72.7%)	44(18%)
**Reward**	2(0.1%)	2(6.1%)	2(0.8%)
**Terms and Conditions**	2(0.1%)	2(6.1%)	2(0.8%)
**Toolkit**	194(6.7%)	24(72.7%)	66(26.9%)
**Treatment**	549(18.8%)	30(90.9%)	87(35.5%)
**Videos**	84(2.9%)	30(90.9%)	62(25.3%)

Intervention fidelity (Clinicians)

Table 21 shows the median number of intervention sessions per participant was 3 (IQR= 2,4) with a mean duration of 36.1 (SD=23.9) minutes.

Table 20:Summary of intervention sessions received by intervention participants during the study

Sessions per participant	
n	33
Mean (SD)	3(1.6)
Median (IQR)	3(2,4)
Min, Max	(0,6)
Total time by participant	
n	33
Mean (SD)	114.2(46.9)
Median (IQR)	100.5(90,125)
Min, Max	(40,249)
Time per session by participant	
n	33
Mean (SD)	37.3(14.2)
Median (IQR)	31.3(28.3,48)
Min, Max	(18,65)
Time per session	
n	99
Mean (SD)	36.1(23.9)
Median (IQR)	30(15,55)
Min, Max	(4,119)
Intervention session per participant	
n	33
Mean (SD)	0.9(0.3)
Median (IQR)	1(1,1)
Min, Max	(0,1)
Total Intervention session time per participant	
n	29
Mean (SD)	58.1(14.2)
Median (IQR)	60(48,60)
Min, Max	(35,90)

Review session per participant	
n	33
Mean (SD)	1(0.5)
Median (IQR)	1(1,1)
Min, Max	(0,2)
Total Review session time per participant	
n	29
Mean (SD)	43.2(30.6)
Median (IQR)	40(20,55)
Min, Max	(10,154)
Preparation session per participant	
n	33
Mean (SD)	0.7(0.9)
Median (IQR)	0(0,1)
Min, Max	(0,3)
Total Preparation session time per participant	
n	14
Mean (SD)	18.4(9.7)
Median (IQR)	15(15,30)
Min, Max	(4,35)
Ad hoc sessions per participant	
n	33
Mean (SD)	0.4(0.6)
Median (IQR)	0(0,1)
Min, Max	(0,2)
Total ad hoc session time per participant	
n	12
Mean (SD)	19.2(6.7)
Median (IQR)	15(15,25)
Min, Max	(15,30)

Clinic visits

Participants completed a median of 2 clinic visits. This was consistent across treatment arms. The number of clinic visits by participant is similar across treatment arms (Figure 9).

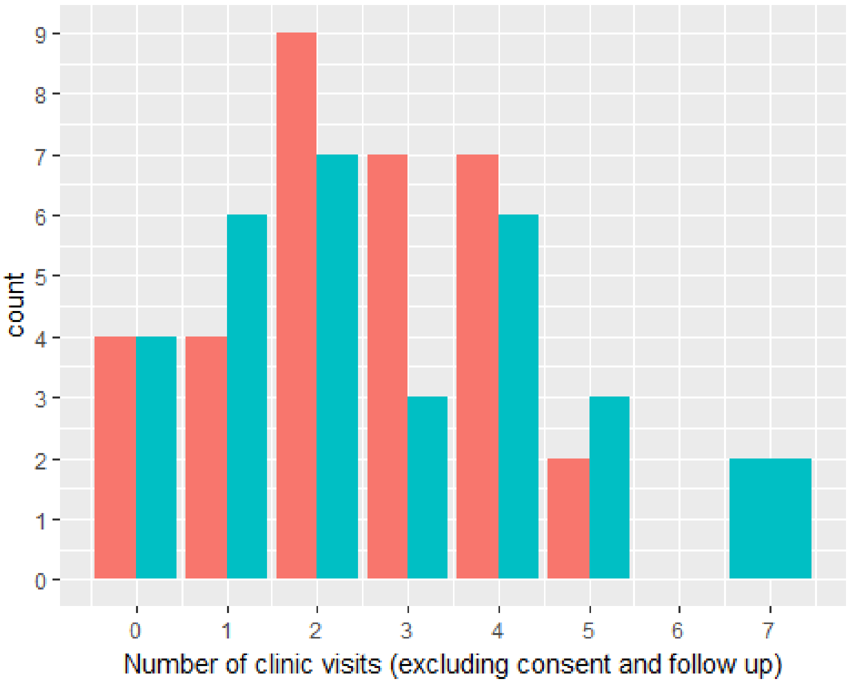

Figure 9:Barplot showing the number of participants for each number of clinic visits by treatment arm

Safety analysis

A total of 8 adverse events (AEs) occurred during the trial and 7 participants (10.9%) had a least one AE (Table 22). 5 of these were deemed to be Serious Adverse Events (SAEs). None of the SAEs were related to the intervention.

Table 21:Summary of adverse events recorded during the study

	Intervention n (%)	Control n (%)	Overall n (%)
All Adverse Events	5	3	8
Participants with at least 1 AE	4(12.1%)	3(9.7%)	7(10.9%)
Type of Adverse Event			
Chest pain or chest discomfort	1(25%)	0(0%)	1(14.3%)
Voice change or Alteration	0(0%)	0(0%)	1(14.3%)
Other	4(100%)	2(66.7%)	6(85.7%)

Table 22:Summary of serious adverse events recorded during the study

	Intervention n (%)	Control n (%)	Overall n (%)
All Serious Adverse events	3(9.1%)	2(6.5%)	5(7.8%)
Level of Seriousness			
Death	0(0%)	2(100%)	2(40%)
Hospitalisation	2(66.7%)	0(0%)	2(40%)
Persistent or significant disability/incapacity	1(33.3%)	0(0%)	1(20%)
Frequency			
Isolated	2(66.7%)	2(100%)	4(80%)
Continuous	1(33.3%)	0(0%)	1(20%)
Intensity			
Moderate	3(100%)	0(0%)	3(60%)
Severe	0(0%)	2(100%)	2(40%)
Outcome			
Recovered	1(33.3%)	0(0%)	1(20%)
Improved	2(66.7%)	0(0%)	2(40%)
Death	0(0%)	2(100%)	2(40%)
Expected SAE			
No	3(100%)	2(100%)	5(100%)
Related to Intervention			
No	3(100%)	2(100%)	5(100%)

Table 23:Description of serious adverse events recorded during the study (table has been redacted to maintain anonymity)

Participant ID	Description of event	Serious
xxx_15	Patient admitted on xx.xx.16 with acute exacerbation, developed type 2 respiratory failure. Despite maximal treatment of IV antibiotics, oxygen and NIV the patient continued to deteriorate and decision made to palliate. The patient died shortly afterwards.	Yes
xxx_14	Patient was having a kidney biopsy and had a bleed as a result, so had been kept in hospital on xxxxx ward at xxx city campus.	Yes
xxx_23	Patient admitted xx/xx/2016 with worsening disease and type 2 respiratory failure. Treated with non -invasive ventilation and intravenous antibiotics. deteriorated despite treatment and passed away xx/xx/2016	Yes
xxx_17	Rash reoccurred after re-trying oral antibiotic medication. Advised to stop again	No
xxx_17	Patient on holiday. Telephoned to report rash on both legs after starting new oral antibiotics. Advised to discontinue	No
xxx_20	Patient was admitted with influenza and CF. Exacerbation treated with iv antibiotics, discharged with home IV's. readmitted on the xx xxx with AKI (Acute Kidney Injury)<br>Assumed secondary to dehydration. Dornase stopped	Yes

Protocol non-compliances

In total, there were 9 protocol non compliances during the trial. 6 (67%) of these were follow up visits conducted outside of the calculated window (5 +/-1 month). 3 (33%) of these were participants ticking statements on the consent form rather than initialling. All of these protocol non compliances were assessed as minor non-compliances.

Summary of missing data

Exacerbation data was collected for 6 months in 60/64 participants (94%). Adherence was collected for at least 6 months for 58/64 participants (90%).

The number of missing scores for questionnaires completed at baseline and 5 month follow up was very low (Table 25). Completion rate was 100% for the majority of baseline questionnaires and at least 89% for 5 month questionnaires. Missing scores were due to drop out(described in section 2.1). Such high completion rates are reassuring for the main trial.

Table 24:Summary of missing scores and items within questionnaires

	Time	Total	%	Intervention Median (min,max)	Control Median (min,max)	Overall Median (min,max)
**EQ5D-5L**	Baseline	64	100 %	5 (5 , 5)	5 (5 , 5)	5 (5 , 5)
**5 items**	5 (+/-1) months	58	90.6 %	5 (0 , 5)	5 (0 , 5)	5 (0 , 5)
**PAM-13**	Baseline	64	100 %	13 (13 , 13)	13 (13 , 13)	13 (13 , 13)
**13 item**	5 (+/-1) months	59	92.2 %	13 (0 , 13)	13 (0 , 13)	13 (0 , 13)
**CHAOS**	Baseline	64	100 %	4 (4 , 4)	4 (4 , 4)	4 (4 , 4)
**4 items**	5 (+/-1) months	59	92.2 %	4 (0 , 4)	4 (0 , 4)	4 (0 , 4)
**MAD-3**	Baseline	62	96.9 %	3 (1 , 3)	3 (0 , 3)	3 (0 , 3)
**3 items**	5 (+/-1) months	57	89.1 %	3 (0 , 3)	3 (0 , 3)	3 (0 , 3)
**SRBAI**	Baseline	63	98.4 %	4 (0 , 4)	4 (4 , 4)	4 (0 , 4)
**4 items**	5 (+/-1) months	59	92.2 %	4 (0 , 4)	4 (0 , 4)	4 (0 , 4)
**GAD-7**	Baseline	64	100 %	7 (7 , 7)	7 (7 , 7)	7 (7 , 7)
**7 items**	5 (+/-1) months	59	92.2 %	7 (0 , 7)	7 (0 , 7)	7 (0 , 7)
**PHQ-8**	Baseline	64	100 %	8 (8 , 8)	8 (8 , 8)	8 (8 , 8)
**8 items**	5 (+/-1) months	59	92.2 %	8 (0 , 8)	8 (0 , 8)	8 (0 , 8)

Recommendations for Main Trial/ Points for discussion

- For the primary analysis in the main trial, we would recommend the use of the offset adjusted model as this will allow the use of more data and allows the inclusion of potentially important participants over a greater amount of time. For example, our original model excluded participants who died, however doing so means we have lost key information.

- This is a pilot study, not powered to detect an effect
- The nature of the data means that small changes appear to influence the result greatly

Appendix

Description of the patient reported outcomes

Name	Score range	Description	Interpretation of score
**EQ-5D-5L**	-0.224-1	Measure of health status	A score of zero means death, 1 is full health, negative score is a state worse than death
**PAM-13**	0-100	Measures patient activation e.g. ability and willingness to manage their health. 13 items with scoring spreadsheet	0= low patient activation<br>100= high patient activation
**CHAOS-6**	0-24	Measures confusion, hubbub and order. 6 item questionnaire	0= low level of chaos<br>24= high level of chaos
**SRBAI**	0-28	Measure of habit and automaticity 4 item, 7 point likert scale	0= low level of automaticity<br>28= high level of automaticity
**CFQ-R**	0-100	8 domains each score 0-100. The domains are:<br>Physical, Emotion, Social, Eating, Body, Treatment Burden, Respiratory, Digestion	0= low<br>100= high
**PHQ-8**	0-24	Measure of depression. 8 item questionnaire, 0-3 for each item	0= No or minimal depression<br>24= Severe depression
**GAD-7**	0-21	Measure of anxiety. 7 item questionnaire	0= No anxiety<br>21= Severe anxiety
**COM-BBQ**			
Specific Necessities	2-5	Measure of perceived personal need for medication	Direction of effect would be an increase in score
Specific Concerns	1-3	Measure of perceived concerns about the negative effects of the medicine they are taking	Direction of effect would be a decrease in score
**MAD-3**	3-15	Specifically made 3 item questionnaire to measure perceived medication adherence	3= low<br>15= high
