# Supplementary material for: Feasibility study for supporting medication adherence for adults with cystic fibrosis: mixed-methods process evaluation
Source: BMJ Open. 2020 Oct 27;10(10):e039089. doi: 10.1136/bmjopen-2020-039089 (PMC7592300; doi:10.1136/bmjopen-2020-039089)
Supplement: Supplementary data [file bmjopen-2020-039089supp007.pdf]

Additional File 07. Changes to intervention procedures

Change Number	Problem type	Problem Identified	Solutions implemented in full-scale trial (hashed numbers - # - refer to logic model constructs)	Timing of change implementation
**CFHealthHub IT component**				
1	Real World and Trial	Interventionists having difficulty identifying videos (#22) appropriate for a patient's needs or interests.	Descriptions were provided with each video. The PPI group agreed with this change and assisted with writing descriptions for each video.	During the feasibility study
2	Real World and Trial	Adherence charts (#14, #20) were showing >100% adherence. This appeared to be more common in patients with alternating regimes, or taking medications *pro re nata* (PRN, meaning 'as needed').	Prescription flow amended with the addition of PRN or alternating regime alerts, which will assist the data management team in highlighting any data discrepancies.	Post-feasibility study
3	Real World and Trial	Clinician functionality (amending prescriptions/ treatment targets (#3, #23) inaccessible through participant view (used in intervention sessions).	Participant view functionality implemented to facilitate intervention sessions. Clinicians are now able to run intervention sessions using CFHH through participant view but easily switch to clinician view to change prescriptions and to set goals.	Post-feasibility study

Hind D, *et al. BMJ Open* 2020; 10:e039089. doi: 10.1136/bmjopen-2020-039089

4	Real World and Trial	The lead psychologist identified the need to determine which participants were receiving push notifications as this relates to dose and rewards for adherence.	The option to export data about number of push notifications sent to participants from the app (#16).	Post-feasibility study
5	Real World and Trial	Originally the normative adherence was used to come up with the percentage adherence. It was identified this did not always match what participants were actually prescribed and this made the graphs difficult to interpret. The capping of the weekly graph at 100% also made interpretation difficult.	To improve interpretability of adherence data (#14), percentages are now calculated against the actual treatments prescribed and graphs are not capped at 100% to aid any interpretation of graphs and trouble shooting.	Post-feasibility study
**Other IT infrastructure**				
6	Real World and Trial	Flatlines at the beginning of some participant adherence run charts were identified to relate to the date registered at the time the nebuliser (#4) is paired with the Qualcomm Hub (#5). Flatlines at the end of the feasibility study were also observed (#14, #35).	To achieve quality assurance of adherence data (#4, #5, #14, #35), hardware is now paired at the factory. The full-scale trial has been monitoring for, and has not found, such instances. Flatlines at the end of run charts established as genuine through triangulation with self-report quantitative and qualitative data.	Post-feasibility study

Hind D, *et al. BMJ Open* 2020; 10:e039089. doi: 10.1136/bmjopen-2020-039089

**Interventionist training and manual**				
7	Real World and Trial	Training packages were initially developed for physiotherapists. This led to interventionist recruitment problems.	The job specification and training was redeveloped to suit non-physiotherapists (#9, #12), to enable any member of the MDT to be trained up to deliver the intervention. A suitably qualified individual such as a postgraduate psychologist could be supported by the MDT to deliver the intervention.	Post-feasibility study
8	Real World and Trial	The interventionist job specification did not reflect the flexibility needed to carry out the interventionist role- e.g. flexibility in working patterns, skills in motivational interviewing and extensive travel.	The research team, with input from the interventionists, revised the job specification for the interventionist role based on experience of delivering the intervention in the pilot in order to better manage expectations of the role **(#12).**	Post-feasibility study
9	Real World and Trial	Pilot study interventionists felt that training was good but could be helped by introducing case studies with real world data, in CFHealthHub.	Realistic case studies with data to support interventionist training / role plays for using website were developed to provide training more applicable to real CF patients (#9). This model is generally used in a healthcare training setting.	Post-feasibility study
10	Real World and Trial	Sporadic training over six weeks, whilst also conducting research procedures was	Training was condensed into an intensive course over ten days, focusing solely on intervention delivery (#9).	Post-feasibility study

		overwhelming for interventionists.		
11	Real World and Trial	Assessment of intervention fidelity identified that some of the active ingredients of the intervention were absent e.g. negotiating goals and letting participants take ownership of choices.	The recruitment and training process was modified to incorporate role play at the interview; explaining fidelity assessment criteria during training and also on-going assessment to ensure that any issues are identified quickly (#9).	Post-feasibility study
12	Real World and Trial	The focus of interventionists during intervention delivery was not always on the aspects that evidence would indicate are the most active ingredients for example goal setting, action planning and coping planning.	Emphasis was placed on the main 'active ingredients' in the manual and in training (#8, #9).	Post-feasibility study
13	Real World and Trial	During the course of the trial, it became apparent that participants were not being followed up and engaged in a manner to allow them to build a habit.	Focus on habit formation / revised logic model will be implemented by a 6-8 week period of habit formation sessions (#8).	Post-feasibility study
14	Real World and Trial	It was identified that after some participants last review visit, their adherence to treatment dropped.	For the full RCT, intervention visits are now triggered if the participant is having an exacerbation/IV, has a drop of 20% or more adherence in the last 4 weeks and if the participant requests additional support.	Post-feasibility study

Hind D, *et al. BMJ Open* 2020; 10:e039089. doi: 10.1136/bmjopen-2020-039089

			These will be termed 'intervention triggers' (#8).	

Hind D, *et al. BMJ Open* 2020; 10:e039089. doi: 10.1136/bmjopen-2020-039089
