# Supplementary material for: Feasibility study for supporting medication adherence for adults with cystic fibrosis: mixed-methods process evaluation
Source: BMJ Open. 2020 Oct 27;10(10):e039089. doi: 10.1136/bmjopen-2020-039089 (PMC7592300; doi:10.1136/bmjopen-2020-039089)
Supplement: Author's manuscript [file bmjopen-2020-039089.draft_revisions.pdf]

BMJ Open is committed to open peer review. As part of this commitment we make the peer review history of every article we publish publicly available.

When an article is published we post the peer reviewers' comments and the authors' responses online. We also post the versions of the paper that were used during peer review. These are the versions that the peer review comments apply to.

The versions of the paper that follow are the versions that were submitted during the peer review process. They are not the versions of record or the final published versions. They should not be cited or distributed as the published version of this manuscript.

BMJ Open is an open access journal and the full, final, typeset and author-corrected version of record of the manuscript is available on our site with no access controls, subscription charges or pay-per-view fees (http://bmjopen.bmj.com).

If you have any questions on BMJ Open's open peer review process please email info.bmjopen@bmj.com

BMJ Open

Feasibility study for supporting medication adherence for adults with cystic fibrosis: mixed-methods process evaluation

Journal:	*BMJ Open*
Manuscript ID	bmjopen-2020-039089
Article Type:	Original research
Date Submitted by the Author:	06-Apr-2020
Complete List of Authors:	Hind, Daniel; The University of Sheffield, ScHARR<br>Drabble, Sarah; The University of Sheffield, Medical Care Research Unit, School of Health and Related Research (ScHARR)<br>Arden, Madelynne; Sheffield Hallam University, Centre for Behavioural Science and Applied Psychology<br>Mandefield, Laura; University of York, Health Sciences<br>Waterhouse, Simon; The University of Sheffield, CTRU<br>Maguire, Chin; University of Sheffield,<br>Cantrill, Hannah; The University of Sheffield, CTRU<br>Robinson, Louisa; The University of Sheffield<br>Beever, Daniel; The University of Sheffield, CTRU<br>Scott, Alex; The University of Sheffield, School of Health and Related Research<br>Keating, Sam; The University of Sheffield<br>Hutchings, Marlene; Sheffield Teaching Hospitals NHS Foundation Trust<br>Bradley, Judy; Queen's University Belfast, School of Medicine, Dentistry and Biomedical Sciences<br>Nightingale, Julia ; University Hospital Southampton NHS Foundation Trust<br>Allenby, Mark; University Hospital Southampton NHS Foundation Trust<br>Dewar, Jane; Nottingham University Hospitals NHS Trust<br>Whelan, Pauline; The University of Manchester, Health e-Research Centre<br>Ainsworth, John; The University of Manchester, Division of Informatics, Imaging & Data Sciences<br>Walters, Stephen; University of Sheffield, ScHARR<br>O'Cathain, Alicia; University of Sheffield, School of Health and Related Research<br>Wildman, Martin; Sheffield Teaching Hospitals NHS Foundation Trust
Keywords:	Cystic fibrosis < THORACIC MEDICINE, Health informatics < BIOTECHNOLOGY & BIOINFORMATICS, Clinical trials < THERAPEUTICS, STATISTICS & RESEARCH METHODS, SOCIAL MEDICINE, Quality in health care < HEALTH SERVICES ADMINISTRATION & MANAGEMENT

SCHOLARONE™
Manuscripts

I, the Submitting Author has the right to grant and does grant on behalf of all authors of the Work (as defined in the below author licence), an exclusive licence and/or a non-exclusive licence for contributions from authors who are: i) UK Crown employees; ii) where BMJ has agreed a CC-BY licence shall apply, and/or iii) in accordance with the terms applicable for US Federal Government officers or employees acting as part of their official duties; on a worldwide, perpetual, irrevocable, royalty-free basis to BMJ Publishing Group Ltd ("BMJ") its licensees and where the relevant Journal is co-owned by BMJ to the co-owners of the Journal, to publish the Work in this journal and any other BMJ products and to exploit all rights, as set out in our licence.

The Submitting Author accepts and understands that any supply made under these terms is made by BMJ to the Submitting Author unless you are acting as an employee on behalf of your employer or a postgraduate student of an affiliated institution which is paying any applicable article publishing charge ("APC") for Open Access articles. Where the Submitting Author wishes to make the Work available on an Open Access basis (and intends to pay the relevant APC), the terms of reuse of such Open Access shall be governed by a Creative Commons licence – details of these licences and which Creative Commons licence will apply to this Work are set out in our licence referred to above.

Other than as permitted in any relevant BMJ Author's Self Archiving Policies, I confirm this Work has not been accepted for publication elsewhere, is not being considered for publication elsewhere and does not duplicate material already published. I confirm all authors consent to publication of this Work and authorise the granting of this licence.

For peer review only

Feasibility study for supporting medication adherence for adults with cystic fibrosis: mixed-methods process evaluation

Daniel Hind[1§], Sarah J Drabble[2], Madelynne A Arden[3], Laura Mandefield[4], Simon Waterhouse[1], Chin Maguire[1], Hannah Cantrill[1], Louisa Robinson[1], Daniel Beever[1], Alexander J Scott[1], Sam Keating[1], Marlene Hutchings[5], Judy Bradley[6], Julia Nightingale[7], Mark I Allenby[7], Jane Dewar[8], Pauline Whelan[9], John Ainsworth[9], Stephen J Walters[2], Martin J Wildman[2,5], Alicia O'Cathain[2].

[1]Clinical Trials Research Unit, University of Sheffield, Regent Court, 30 Regent Street, Sheffield, S1 4DA, UK

[2]School of Health and Related Research, Regent Court, 30 Regent Street, Sheffield, S1 4DA, UK

[3]Centre for Behavioural Science and Applied Psychology, Sheffield Hallam University, Collegiate Crescent, Sheffield, S10 2BQ

[4] Department of Health Sciences, University of York, University of York, Seebohm Rowntree Building, Heslington, York, YO10 5DD

[5] Sheffield Adult Cystic Fibrosis Unit Sheffield Teaching Hospitals NHS Foundation Trust, Northern General Hospital, Herries Road, Sheffield, S5 7AU

[6] Wellcome-Wolfson Institute For Experimental Medicine, School of Medicine, Dentistry and Biomedical Sciences, Queen's University Belfast, UK..

[7] Wessex Adult Cystic Fibrosis Service, University Hospital Southampton NHS Foundation Trust, Tremona Road, Southampton, Hampshire, SO16 6YD.

[8] Wolfson Cystic Fibrosis Centre, Nottingham University Hospitals NHS Trust, City Hospital, Hucknall Road, Nottingham, NG5 1PB.

For peer review only - http://bmjopen.bmj.com/site/about/guidelines.xhtml

[9] Health eResearch Centre - Division of Imaging, Informatics and Data Sciences, School of Health Sciences, Faculty of Biology, Medicine and Health, The University of Manchester, Manchester Academic Health Science Centre, Manchester, United Kingdom

[§]Corresponding author

Email addresses:

DH: d.hind@sheffield.ac.uk

SD: s.j.drabble@sheffield.ac.uk

Madelynne A: M.Arden@shu.ac.uk

LM: l.mandefield@sheffield.ac.uk

SW: s.waterhouse@sheffield.ac.uk

CM: c.maguire@sheffield.ac.uk

HC: h.cantrill@sheffield.ac.uk

LR: louisa.robinson@sheffield.ac.uk

DB: d.a.beever@sheffield.ac.uk

AS: alex.scott@sheffield.ac.uk

SK: s.m.keating@sheffield.ac.uk

MH: marlene.hutchings@sth.nhs.uk

JB: judy.bradley@qub.ac.uk

JN: julia.nightingale@uhs.nhs.uk

Mark A: mark.allenby@uhs.nhs.uk

JD: jane.dewar@nuh.nhs.uk

PW: pauline.whelan@manchester.ac.uk

JA: John.Ainsworth@manchester.ac.uk

For peer review only

SJW: s.j.walters@sheffield.ac.uk

AOC: a.ocathain@sheffield.ac.uk

MW: martin.wildman@sth.nhs.uk

For peer review only

For peer review only - http://bmjopen.bmj.com/site/about/guidelines.xhtml

Abstract

Objectives

To undertake a process evaluation of an adherence support intervention for people with cystic fibrosis (PWCF), to assess its feasibility and acceptability.

Setting

Two UK Cystic Fibrosis (CF) units

Participants

Fourteen adult PWCF; 3 professionals delivering adherence support ('interventionists'); 5 multi-disciplinary CF team members.

Interventions

Nebuliser with data recording and transfer capability, linked to a software platform, and strategies to support adherence to nebulised treatments facilitated by interventionists over five months (+/- one month).

Primary and secondary measures

Feasibility and acceptability of the intervention, assessed through semi-structured interviews, questionnaires, fidelity assessments, click analytics.

Results

Interventionists were complimentary about the intervention and training. Key barriers to intervention feasibility and acceptability were identified. Interventionists had difficulty finding clinic space and time in normal working hours to conduct review

For peer review only

visits. As a result, fewer than expected intervention visits were conducted and interviews indicated this may explain low adherence in some intervention arm participants. Adherence levels appeared to be >100% for some patients, due to inaccurate prescription data, particularly in patients with complex treatment regimens. Flatlines in adherence data at the start of the study were linked to device connectivity problems. Fidelity assessments identified that interventionists needed to focus more on the 'active ingredients' of the intervention during sessions.

Conclusions

The process evaluation led to 14 key changes to intervention procedures to overcome barriers to intervention success. With the identified changes, it is feasible and acceptable to support medication adherence with this intervention.

Registration: ISRCTN13076797; 7th June 2016.

Strengths and limitations of this study

- This is a detailed evaluation of the feasibility of an adherence support system for people with cystic fibrosis.
- The use of mixed methods provided indepth understanding of the processes involved in delivering the service, its value, and factors that might influence its use, implementation and success.
- This was a small, two-centre study.

For peer review only - http://bmjopen.bmj.com/site/about/guidelines.xhtml

Background

Cystic Fibrosis (CF) is a long-term inherited condition affecting over 80,000 people worldwide, primarily of Northern European ancestry [1]. Median survival for people with CF (PWCF) is estimated at 31 years [2–6] with progressive lung function decline, caused by regular infection and damage to airways, being one of the main disease features [2].

Preventative medications preserve lung function and reduce exacerbations [7–13].Low adherence to these medications is problematic as this predicts exacerbations requiring intravenous antibiotics (IVAB)[14,15]. Exacerbations of this nature carry a risk of systemic side effects of both increased mortality[16,17], and cost of care [18–20]. In 2012, the total spend on CF in the UK was estimated to be £100 million, with £30 million spent on inhaled antibiotics and mucolytics[21]; the UK CF population received 171,907 days of IVAB with 93,455 days received in hospital, costing an estimated £27 million[22].

Self-reported adherence to inhaled therapies underestimates objectively-measured adherence, with rates of 80% and 36% recorded, respectively[23] and systematic data collection suggests objective adherence to be closer to 30%[24]. As a result, clinicians are currently unable to identify PWCF with low adherence, in order to provide additional support.

Consistent with identified research priorities[25,26], a complex intervention was developed [Cite companion paper] to support inhaled medication adherence in PWCF. This article presents the results of a process evaluation that was undertaken alongside

For peer review only - http://bmjopen.bmj.com/site/about/guidelines.xhtml

a pilot RCT, and describes the resultant changes made to intervention procedures prior to a full-scale RCT[27,28]. The specific objectives of the process evaluation were:

1. To triangulate qualitative and quantitative data collected on intervention inputs, engagement, activities, and contextual factors, alongside immediate and intermediate outcomes recorded in the feasibility study, to understand and identify potential barriers to intervention implementation and success.
2. To document and use these findings to guide changes to intervention procedures, ahead of a future, full-scale RCT.

Methods

The wider feasibility study

The process evaluation forms one part of a wider pilot study, which also assessed the feasibility of RCT procedures (reported elsewhere[29]). The pilot RCT consisted of 33 intervention patients and 31 control patients. Three trained interventionists in two UK CF centres delivered the intervention to PWCF in the intervention arm and followed them up for 5 months, plus or minus one month.

Intervention description

The complex intervention to support adherence in CF was developed to enable PWCF to manage adherence to nebulised medication, with a view to shifting CF treatment from rescue in hospital settings to prevention, managed in the community. The full intervention development process is described in a separate article[29].

The complex intervention consists of four key elements: the eTrack, CFHealthHub server, the CFHealthHub Apps and the manualised behavioural intervention. A logic model (Figure 1) was produced to reflect, in detail, constructs and processes by which

For peer review only

the intervention was expected to function; this is in terms of inputs, engagement, activities, and outcomes. The logic model's hashed numbers (#1, #2, etc) provide a reference for linking intervention materials and processes to logic model constructs in Figure 1.

The eTrack (#4) (PARI Pharma GmbH, Starnberg, Germany) is a microchipped nebuliser, enabling real-time monitoring of adherence to nebulised medications. Timestamped records of medications administered via the eTrack are sent to a 2net Hub (Qualcomm, San Diego, USA; #5) which transmits data to PARI.

Real-time inhalation data is received by the CFHealthHub (CFHH) server infrastructure, stored securely and used for display in both a web-based interface and a mobile app (#6, see Figure 2). Each of these displays adherence data alongside tools to support behaviour change and educational content.

Participants and their interventionists had access to adherence displays for monitoring (#13, #19, #20) and other CFHH content (#21- #26), such as education about treatments (#21) and problem solving in the face of adherence barriers (#26). Interventionists would use CFHH to facilitate delivery of manualised behavioural intervention sessions (#8, #17).

Health professionals (n =3) included a clinical psychologist, a physiotherapist and a social worker. They received specific training to deliver the manualised intervention sessions (#9). Training was delivered over two days, in face-to-face workshops. This was supplemented by online learning modules and a further four-week training

For peer review only

For peer review only - http://bmjopen.bmj.com/site/about/guidelines.xhtml

schedule. Interventionists were assessed with online theory tests and in a competency assessment which examined intervention delivery within the first 5 sessions.

Sessions were delivered either face-to-face or remotely, on a one-to-one basis. All intervention arm participants received an initial intervention visit and a minimum of one additional review visit over the period of the study (#18). The content of sessions varied by participant reported motivation; sessions for those with low motivation were tailored to promote relationship / confidence building and to support the participant in the exploration of relevant CFHealthHub educational and information material (#21, #22). Relevant material could be added to the participant's personalised 'Toolkit'. Sessions conducted with participants displaying higher motivation would also involve supporting the participant to set personalised adherence goals (#23, #24), and to make action plans (#25) and engage in problem-solving including making coping plans where relevant (#26).

Design

A mixed-methods approach was used for the process evaluation. Although this pragmatic case study[30,31] primarily works at the level of the programme, we also present a nested multiple-case design, with cases at the level of the PWCF, and two embedded units of analysis – interviews with intervention participants and trial data.

Data Sources

Quantitative and qualitative data sources were triangulated to address process evaluation objectives. These are described using hashed numbers to relate data sources to aspects of the logic model (Figure1) for which they contributed data.

For peer review only - http://bmjopen.bmj.com/site/about/guidelines.xhtml

Qualitative data included: verbal reports from project staff (#1. #2, #10, #16); semi-structured interviews with interventionists and participants in the intervention and control arms of the pilot RCT (#8, #9, #12, #13, #14, #15, #16, #17, #19, #20, #21); minutes of meetings (#3); emails (#4), website development reports (#6); and fidelity assessments (#17).

Quantitative data included: implementation log entries and data management reports (#3), questionnaire data derived from secondary clinical outcome measures described in Table 1 (#7, #28, #29, #30, #31, #32, #33) an interventionist-completed structured questionnaire on interventionist confidence post-training (#9), structured interventionist fidelity assessments in which audio-recordings of intervention sessions were coded using a fidelity scoring system which assessed whether each component of the intervention was delivered and the quality of that delivery (#11, #17), CFHH click analytics (#13, #14, #15, #18, #20, #21, #22, #23, #24, #25, #26), session frequency and duration records (#15); and adherence data taken from CFHH (#35). These 23 logic model constructs were collected as part of the trial protocol from sources described in Table 1.

Semi-structured interviews, conducted face-to-face, were digitally audio-recorded and transcribed verbatim. The median length of interviews was 30 minutes (range 11 to 87) for PWCF, 86 minutes (63 to 102) for interventionists and 62 minutes (51 to 66) for CF team members.

Sampling

Participants were recruited for semi-structured interviews. Participants included intervention arm participants (n=14), interventionists (n=3, 0.8 WTE at each centre) and members of the wider, multi-disciplinary CF team (n=5). Participants were

For peer review only

For peer review only - http://bmjopen.bmj.com/site/about/guidelines.xhtml

purposively sampled based on site, age, gender, deprivation index, objective and subjective adherence levels (service-users), or site and professional category (professionals). Interventionists were interviewed twice – at the beginning and end of the study – patients once. PWCF who consented to be approached for interview were contacted by letter or email and, subsequently, telephone or email depending on preference. Professionals were contacted directly by the study team.

Data Analysis

We conducted a Framework analysis of interview transcripts[32], within NVivo (QSR International) using multiple frameworks including the Theoretical Domains Framework[33], a process evaluation framework[34], and the logic model (Figure 1).

Using a modified triangulation protocol[35], we integrated qualitative and quantitative datasets at the programme- and the case-level[36]. We used a joint display table[37] to summarise data sets for 35 logic model constructs in the Inputs (n=12), Engagement (n=6), Activities (n=7), Immediate outcomes (n=6) and Intermediate outcomes (n=2) columns (Figure 1). The fit of data integration was categorised as: 'confirmation' (quantitative and qualitative data provided similar findings); 'expansion' (the datasets addressed different or complementary aspects of the phenomenon); or, 'discordance' (the datasets were contradictory)[38]. We described similar and unique contributions , made by the two data sets, to the research question [35].

In the 14 intervention participants, for whom both qualitative and quantitative process data was available, we produced case-profiles[39], triangulating qualitative data with

For peer review only - http://bmjopen.bmj.com/site/about/guidelines.xhtml

individual-participant adherence run charts[40] (Additional File 01) and other quantitative process data (see Additional File 02 – Study protocol, pp29-31). We worked abductively, moving between behaviour change theories[41,42] and contextual observations, agreeing plausible hypotheses to explain patterns which could be tested in future work [43–46].

We produced a case-ordered descriptive matrix[47], with cases ranked by average adherence during the last month of the study, to understand how processes and outcomes were mediated by local and individual conditions. Adherence levels of >80% were assessed as high; 50-80% moderate; <50% low [14,48]. We theorised that high life chaos, as measured by the Confusion, Hubbub and Order Scale (CHAOS)[49] and low motivation would be associated with low adherence. We used four measures to understand motivation: (1) a single item, scored on a 1-7 Likert scale – "I want to do all of my nebuliser treatment" (motivation); (2) a single item, scored on a 1-7 Likert scale, which asked, "I am confident I can do all of my nebuliser treatments" ('confidence'); (3) the necessities and, (4) concerns five-point subscales of the Beliefs about Medicines Questionnaire nebuliser-specific (BMQ) instrument [50]. Interventionists assessed the participant's motivation to increase adherence on a one to seven scale after discussion with the patient; adequate motivation was necessary before participants could make action plans and do problem solving activities.

Approach taken to modifying the intervention

Modifications to the intervention fell into three categories: the software platform; other IT infrastructure; and the manual and training. Identified problems and solutions were tabulated following a modified approach of that taken by Bugge [28]. Digital

For peer review only - http://bmjopen.bmj.com/site/about/guidelines.xhtml

For peer review only

platform development was reviewed regularly using the "Must have, Should have, Could have, and Won't have but would like" (MoSCoW)[27], often used in agile software development [51,52].

Patient and Public Involvement

Recruitment for the Patient and Public Involvement (PPI) Group was achieved by advertising within CF units and on the People in Research website, as well as via group members themselves. Cross-infection between PWCF[53] was prevented by arranging meetings via teleconference. The PPI group gave feedback on intervention data-sharing policies, usability and presentation of the website/user-guide. In addition, the PPI group piloted the participant information materials and one individual gave feedback on the trial protocol and interview guides (Additional File 02).

Ethical approval

The study received approval from London Brent Research Ethics Committee (16/LO/0356). The funder was not involved in the trial design, patient recruitment, data collection, analysis, interpretation, or presentation, writing or editing of the report, or the decision to submit for publication. The corresponding author had full access to all the data in the study and had final responsibility for the decision to submit for publication.

Results

In what follows, we address contextual factors that affected implementation and participant responses, then follow the columns (inputs, engagement, activities, immediate and intermediate outcomes) of the logic model. Additional File 03 summarises quantitative process outcomes for 14 case study participants, ranked by

For peer review only - http://bmjopen.bmj.com/site/about/guidelines.xhtml

objective adherence at the end of the trial. Hashed numbers (#1, #2, etc) indicate cross references to the logic model (Figure 1) and supporting evidence in Additional File 04, which summarises data triangulation at the level of individual logic model constructs. Both qualitative and quantitative data were available for 13/34 logic model constructs, providing confirmation of (n=2) or expansion on (n=11) inferences drawn from quantitative data. A case-ordered descriptive matrix based on logic model columns (Additional File 05) and run charts annotated with key events (Additional File 01) provides an integrated analysis at the level of the participant for fourteen 'case studies', cross referenced by participant numbers (R02/52, R01/54, etc).

Contextual factors affecting implementation and participant responses

The key factor affecting implementation was the mixed economy of CF drug delivery systems: the e-Flow (PARI Pharma GmbH, Starnberg, Germany); the iNeb (Philips Healthcare, Eindhoven, Netherlands); and a number of dry powder delivery systems. The e-Flow is the only device able to deliver all the wet nebulised drugs that are used in CF care. The e-Track we used in this trial was a version of the e-Flow developed to transfer time- and date-stamped data. Most patients at site R01 used e-Flows; switching consenting participants over to the e-Track was generally unproblematic. The e-Flow's competitor, the iNeb, cannot deliver aztreonam and requires double chamber filling to deliver tobramycin, so it is not suitable for all patients. The data transfer version of the iNeb, the BiNeb, is a prototype for which limited numbers are available. We were unable to secure approval to integrate the BiNeb into CFHH in time to incorporate it into this study. At site R02 where iNebs were commonly used, those who were familiar with and liked the iNeb were less keen to swap to an alternative device; some who swapped to the e-Track, later wanted to move back to

For peer review only - http://bmjopen.bmj.com/site/about/guidelines.xhtml

the iNeb. A minority of patients use dry powder delivery systems, none of which have data transfer versions. We were unsuccessful in engaging any of the companies producing dry powders in time to get dry powder systems integrated into CFHH, meaning that dry powder users could not be recruited to this feasibility study. Making nebulisers with data recording and transfer capability available within hospitals following local delivery took prolonged engagement with medical engineering departments to obtain local safety approvals. For more than one participant, the strength of their mobile data signal affected 2net Hub connectivity with the central server (Implementation log, 19 Oct 16).

Through meetings with site staff, the team identified a range of human factors that also affected implementation. The struggle for clinic space and patient convenience resulted in more home visits than anticipated for consent and review meetings, informed by local lone-working policies. Reorganisation of one CF Centre, involving the transfer of patients from the care of one local hospital to another, had created discontent among some patients involved in the trial.

Inputs

The study chief investigator reported introducing local site investigators, centre directors and MDTs to CFHH **(#1)**. Through case reports, he conveyed that relying on FEV_1, symptoms and BMI for CF management alone is inadequate and that objective adherence data could help overcome the 'lamppost syndrome'[54], also known as the 'streetlight effect'[55,56] or 'drunkard's search'(page 11[57]) – a type of availability bias[58]. The chief investigator reported feeling that site investigators at both centres

For peer review only - http://bmjopen.bmj.com/site/about/guidelines.xhtml

were fully bought in, but that one clinician (not an investigator) believed that the disparities between subjective and objective adherence[23] were overstated **(#2)**.

Interventionists entered prescription data into CFHH based on patient records and self-reported treatment regimen **(#3)**. Occasionally, interventionists were slow to make monthly prescription checks when prompted by system alerts, resulting in apparent adherence levels of over 100%, traced to the use of alternating treatment regimens[59] (Implementation Log, 01 Dec 16, TMG minutes 10 Jan 17). Nebulisers with data recording and transfer capability **(#4)**, 2net Hubs **(#5)**, the CFHH website and mobile application **(#6)**, were made available (emails to project manager 20 May 16, 23 Jun 16). The Capability Opportunity Motivation -Beliefs about Medicines Questionnaire (COM-BMQ – see Additional File 02)[50] questionnaire data **(#7)** was collected in CFHH (Additional File 06 - Table 8).

Interventionists were complimentary about the intervention manual **(#8)** and highly satisfied with training, but suggested that future courses involved a case study approach, following a patient through the intervention to illustrate its different aspects **(#9)** (Additional File 04). A member of the research team (MH) acted as an intervention mentor to interventionists **(#10)**. Interviews (SD) and observations (MH, HC) identified differences in the way site investigators interacted with interventionists, with one giving more intensive practical support, through weekly meetings and problem-solving (not prescribed by the intervention), than the other. Fidelity data was collected on all three interventionists and the fidelity assessment instrument was modified before use in the full RCT **(#11)**. During interviews, interventionists were enthusiastic about intervention processes **(#12)**. As sites

For peer review only - http://bmjopen.bmj.com/site/about/guidelines.xhtml

struggled to find space or time for consent / intervention encounters in clinic, the study team requested an increase in the number of home visits (Implementation log 19 Oct 2016). As a result of initial problems in contacting participants and the need for flexibility in arranging meetings out of usual clinic hours, the study team requested flexible working in which the team worked 12:00-20:00 two days a week (interviews & TMG minutes 29 Nov 2016).

Engagement

Interviews and click analytics showed that MDT members did not access adherence data **(#13)**, aside from in the form of bar charts brought to MDT meetings by interventionists. It is important to note that extending the use of CFHH to the MDT was not an objective of the trial and no training was given in this regard. Click analytics showed that interventionists tracked adherence **(#14)**. Of 14 case study participants, three did not contribute complete adherence data: R02/42 and R02/02 withdrew, while R02/03 was lost to follow-up. In other participants, flatlines in adherence data caused concern (Additional File 01). Flatlines at the beginning of the study (e.g. R01/39, R01/48) indicated technical problems with pairing nebulisers and hubs. Flatlines at the end of the study period (e.g. R01/42, R01/44, R02/12) were confirmed as the genuine recording of non-adherence through the use of adherence data beyond the end of the study period, interview data, self-report subjective adherence and the MAD-3 (Additional File 03 – Table f).

Click analytics showed the median number of participant CFHH sessions was three **(#15)** (Additional File 03 – Table c). Of those with low usage, initial technical problems (R01/02, R01/48) and initial lack of availability of a mobile application (#6)

For peer review only - http://bmjopen.bmj.com/site/about/guidelines.xhtml

were potential contributing factors. Some case study participants showed moderate (R02/52, R01/54 and R01/40: 9-13 sessions) or high use (R02/12 and R01/42: >40 sessions). Push notifications - user-defined messages from the server which give participants congratulations or reminders about adherence behaviour – were not available in the pilot trial **(#16)**.

Based on fidelity assessment of intervention session recordings, the *content* fidelity of face-to-face interactions, was excellent (100%) – with all aspects delivered as per the manual **(#17)**. Delivery *quality* fidelity was more variable (60-92%). The generation of goals and action plans was sometimes too directive rather than negotiated and supportive. Interviews demonstrated that assessing the true level of motivation to adhere to treatment was challenging; sometimes those with insufficient intrinsic motivation (e.g. R01/48, R01/54 and R02/03) were assessed as having sufficient motivation and inappropriately tasked with setting and reviewing goals, making action plans and problem solving (see below #23-26). These individuals were variably motivated by wanting to prove themselves to MDT members, who had doubted their adherence (R01/49 and R01/54, Additional File 05), or by helping the research:

> *"I made that special effort 'cause I was taking part in this trial… I didn't see how it was going to make me better" (R01/48).*

Interaction with these individuals should have been confined to relationship-building and trust-building. Fidelity assessment of recordings identified that, in interactions with the adequately motivated, the focus was not always on the most active ingredients – goal-setting, action planning (habit formation) and problem-

For peer review only - http://bmjopen.bmj.com/site/about/guidelines.xhtml

solving/coping planning. Participant run charts (Additional File 01) revealed a disparity in whether and when review visits happened **(#18)**.

Activities

In interviews, CF team clinicians (as distinct from the interventionists) confirmed they were not monitoring adherence as part of usual care **(#19)**. Participant R01/02 complained that the research focus on adherence was "*parallel rather than integrated*" with mainstream clinical management. However the intervention was designed to be interventionist delivered allowing individual randomisation in a system without contamination of controls rather than an intervention aimed at achieving system change which would have required a cluster trial design. Participants' clicks (median 11) on the CFHH "How am I doing?" (run charts) page sometimes related to a limited number of sessions. In interviews, one moderately frequent user (R01/54) only accessed this page to check their data was uploading. Other moderate/frequent users described this page as important for adherence self- monitoring **(#20)**, even when their grasp of their own adherence was poor (R01/49).

In interviews with participants, for tailored education about treatment **(#21)**, participants accessed particular education pages for specific issues, such as nebuliser malfunction, which was viewed as, "more down to earth" than technical manuals. In particular a video about the treatment action of Dornase alfa, was often praised, as a means of educating others about CF; 'Talking heads' videos (in these videos people with CF described strategies for successful nebuliser use) **(#22)** divided opinion: for some, the opportunity for social comparison[60] provided relief and reassurance; those who were less appreciative were those who found comparisons with people

For peer review only - http://bmjopen.bmj.com/site/about/guidelines.xhtml

healthier than themselves could make them feel as though they were not doing well and comparisons with those less healthy could make them fearful of the future.

Other activities (#23 to #26 on the logic model) required participants to have adequate levels of motivation. Interventionists classified all but one case study participant (R01/44) as having adequate motivation (Additional File 03, Table b) and therefore eligible for further tailored intervention. But, as detailed above (see #17 in the engagement section), this was sometimes based on inadequate discussion with the participants. In interviews, participants generally reported setting goals **(#23)**, but fidelity assessment showed that goals were sometimes formulated by interventionists rather than by participants (see #17). The mean number of review sessions **(#24)** over five months was 1 (Additional File 03 – Table e); this was fewer than intended, likely reflecting a failure of the study team to set appropriate expectations and a lack of time created by the high pace of recruitment (problem log entries: 31-Jan-17; 13-Feb-17). Two individuals (R01/39 and R01/40) received their first face-to-face session with an interventionist over halfway through the study period (Additional File 01). CFHH action plan **(#25)**, problem solving and coping plan **(#26)** pages were accessed a median of two, three and one times, respectively (Additional File 03 – Table e). Interviews data suggests action / coping plans were completed during intervention visits but not accessed by participants otherwise. In interviews, some participants said they were reassured by the presence of, and sometimes reported insights from, problem-solving modules, such as what to do when going on holiday. However, the use of action plans was disliked by some participants who found writing down the action plans like "*going back to school*". This dislike at least partly reflected the

For peer review only - http://bmjopen.bmj.com/site/about/guidelines.xhtml

generation of action and coping plans by interventionists rather than by the participants themselves (see #17).

Immediate outcomes

The pilot was not designed to disseminate the intervention across the centre and with minimal monitoring by professionals within the wider CF team (see #19) routine medical care was not informed by adherence **(#27)**. Unsurprisingly, given the lower than expected face-to-face contact **(#18, #24)**, intervention arm group averages for immediate (process) outcomes **(#28-33)** changed little over five months, with the exception that there was a mean reduction of 1.84 (SD 3.44) barriers to adherence per person **(#33)**, which could be the outcome of problem solving and education about treatment processes (Additional file 03 – Table f). Frequent use of CFHH and self-monitoring in particular (see above, #20) did not necessarily mean that self-reported subjective adherence and electronically-captured objective adherence were well aligned **(#28)** (Additional file 03 – Tables f and g). A *post hoc* paired comparison of subjective and objective adherence at 5 (+/-1) months (Figure 3) suggests that higher adherers were more uniformly accurate in their understanding of their own adherence, whereas low adherers could be overly optimistic.

Intermediate outcomes

Item **#34** of the logic model, treatment optimisation, is defined by NICE as, "a person-centred approach to safe and effective medicines use" to ensure best outcomes[61]. Treatment optimisation is a service-level objective, which was beyond the scope of our patient-focused intervention but is the subject of related ongoing research (see Discussion). During interviews, RCT participants in the intervention

For peer review only - http://bmjopen.bmj.com/site/about/guidelines.xhtml

arm described behaviours that would affect treatment optimisation, for instance taking holidays from their treatment. Levels of CF treatment adherence **(#35)** were 10% (95% CI: -5.2 to 25.2) higher in the intervention arm (Additional File 06, Table 17). We developed a number of theories about why some intervention patients did or did not increase their adherence **(#35)** during the analysis. In some cases the run charts illustrated, in line with Control Theory, the goal-directed nature of behaviour and how it is regulated by feedback control processes[62]. For example, R01/39 and R01/49 seemed to show improvement shortly before planned face-to-face visits from interventionists (Additional File 01). R01/39, who seemed intrinsically motivated when interviewed, sustained improvement in adherence beyond the trial period through what they described as positive interaction with the interventionist. Others, who seemed more extrinsically motivated in interviews (R01/49, R01/54, R01/48: see #17), did not sustain adherence, with charts suggesting an effortful, 'all-or-nothing' pattern. At baseline, R02/07 had no well-established routine (CHAOS score of 10: Additional File 01), implying substantial self-regulatory effort to achieve higher adherence. In their interview, this participant reported finding habit formation parts, such as goal-setting, helpful which may have enabled him to maintain high adherence with reduced effort, as measured by increased habit and reduced life chaos and barriers (change scores -5 and -3 respectively: Additional File 03 - Table f). Finally, it is important to understand that individual-level adherence can be unstable over time (Additional File 01, see especially, R01/54, R01/48) highlighting the problem of assessing adherence as a 'snapshot' in a pre-/post-test analysis, rather than in a continuous assessment over time.

For peer review only - http://bmjopen.bmj.com/site/about/guidelines.xhtml

Several participants with low baseline adherence appeared to have responded well to the intervention. R01/40 had high motivation (Additional File 03 – Table b; Additional File 01), possibly due to the salience of a recent hospitalisation for IVAB treatment of an exacerbation. Click analytic data showed high engagement, with independent access of the website and use of problem-solving tools. However in other patients, case study run charts (Additional File 01) showed that measuring change in average objective adherence between baseline and five months sometimes masked periods of success in between (e.g. R01/02, and R02/12). Without looking at adherence graphs, and only measuring objective adherence at baseline and five months, this would have been missed (see Discussion). Interview data offered some reasons for improved adherence. While R01/49 had not made an action plan and their subjective adherence was optimistic (Additional file 03 – Table f), their objective adherence increased from low to moderate over the trial period (Additional File 01); their motivation also increased and self-reported barriers decreased (Additional file 03 – Table f), potentially through their high use of problem-solving modules and self-monitoring (Additional File 03, Table d). R01/02's run chart also showed a period of improvement, ending after the last review visit (Additional File 01); nonetheless, reduced life chaos (Additional file 03 – Table f) and interview data suggested an established routine and reduced barriers associated with intensive face-to-face therapist interaction and action/coping plans (Additional File 03, Table d). The tailing off of adherence after the end of the trial in some case study participants may indicate that adherence remained effortful or participation in the trial was motivated by altruism not help-seeking (see quotation from R01/48, above).

For peer review only - http://bmjopen.bmj.com/site/about/guidelines.xhtml

Modifications to the intervention

Additional file 07 documents 14 technical changes that will be made for the full-scale RCT, based on the process evaluation findings, to CFHH (n=5), IT infrastructure (n=1) and to the interventionist training, manual and procedures (n=8). To prevent adherence data flatlines, nebulisers **(#4)** and 2net Hubs **(#5)** will be paired at the factory. Three changes to CFHH **(#6)** will make it easier for interventionists to view/edit prescription data and to handle alternating treatment regimens **(#3)**. Other changes to CFHH will include making graphs more easily interpretable and, based on interview data and PPI feedback, adding descriptions to videos. Changes to the interventionist manual **(#8)** will increase the emphasis on 'active ingredients', introduce intervention triggers for reduced adherence or exacerbations and introduce new habit formation sessions. The need for increased numbers of protocolised intervention review sessions arose because, in the feasibility study, a focus on RCT recruitment targets gave interventionists inadequate time to deliver review visits **(#18, #24)**, critical for updating personalised action plans **(#25)** and updating coping plans **(#26)**. Training **(#9)** in the full-scale trial will be delivered as an intensive one-week course, with more explicit focus on intervention fidelity, supported by new case study data and role plays to ensure baseline competency **(#17)**.

Discussion

The process evaluation identified elements of the intervention which could be improved and 14 changes were documented. The complex intervention was developed using mixed methods research with an inter-disciplinary, person-centred and iterative approach[63–69]. The mere usage of a digital behaviour change intervention may not indicate engagement or lead to desired outcomes[63,68,70–73]; there is no simple

For peer review only - http://bmjopen.bmj.com/site/about/guidelines.xhtml

dose-response relationship[74]. In fact, for those with low motivation and low confidence, evidence of non-adherence can be threatening[75]. With different baseline motivation and life chaos, a population-level definition of “effective engagement”[65] may be infeasible, but contextual and motivational data may still explain patterns observed in run charts[76]. What may matter more than defining engagement is the correct assessment and tailoring of management to different psychosocial barriers[64,77–85]. Our study suggests that digital systems cannot replace, only complement, face-to-face interaction between health professionals and patients[86–88], potentially creating a sense of ‘accountability’ consistent with control theory[42,89]. However, it is important to recognise that in the absence of objective adherence data clinicians and patients will find it difficult to even begin to engage with behaviour change.

Our use of objective adherence measurement overcomes the limitations of previous studies[90] and confirms that subjective and objective adherence are poorly aligned[23]. This process evaluation has succeeded in demonstrating that delivery of this intervention is possible in busy clinical settings; participant uptake was high and, with further development on the basis of these findings, the process of gathering objective adherence data and implementing it alongside a behavioural intervention is both possible and effective.

Given the known difficulties with nebuliser use among PWCF, interventions that can make it less effortful are important[91]. In particular, healthy behaviours are better predicted by a patient’s level of automatic behavioural repetition than their beliefs or experiences, meaning a focus on increasing habit strength is critical for chronic disease self-management[92]. Through delivery of intervention components designed

For peer review only - http://bmjopen.bmj.com/site/about/guidelines.xhtml

to promote habit formation, we intend to reduce effort with the CFHH intervention. We are limited in drawing conclusions as to the impact of habit formation components of the intervention from this analysis; this is mostly due to the limited time constraints of the feasibility study leaving insufficient opportunity for habit formation[93]. However, there was some indication that habit components were useful and we have elsewhere demonstrated the importance of habit in high adherence [94,95]. It has also been indicated that adherence interventions focusing on habit formation are the most effective[96].

Successful habit formation will reduce burden by making sustained self-care automatic. The CFHH intervention aims to deliver the fall in burden highlighted by the Lind alliance prioritisation exercise as the most important goal of CF research.

To date, there is little previous research showing the effects of giving patients access to their data, with respect to health outcomes and cost-effectiveness. Amidst the evidence that does exist, the research is generally poor and lacks information about context and implementation[97,98]. Following modifications made to our complex intervention, the full scale RCT across 19 UK centres (ISRCTN55504164) will provide high quality evidence, indicating the impact of adherence data on sustained self-care. The full-scale RCT will include a further process evaluation and health-economic modelling. Furthermore, the CFHealthHub Data Observatory (ISRCTN14464661) following on from the RCT will address the issue of how to embed the use of adherence data in routine practice for healthcare professionals[99–103] . The sites involved in the reported pilot study have now transitioned into the Data Observatory, eventually to be joined by sites involved in the full-scale RCT. Data collected in the data observatory quality improvement project will be used in the

For peer review only

For peer review only - http://bmjopen.bmj.com/site/about/guidelines.xhtml

development of generalisable theory and practical guidance about the collaborative use of adherence data [104,105], with a focus on optimising the use of health care resources and improving patient care [61]. The Observatory will act as a platform for efficient trials[106,107], providing an opportunity to share processes and improvement activities to enable participating CF clinical research teams to meet the demands of future research [108].

Conclusions

We have developed a theory-based complex intervention to help PWCF adhere to their medication and form habits of sustained self-care. The process evaluation identified potential sources of intervention failure and modifications have been made accordingly. With improved intervention processes, it is feasible and acceptable to support sustained self-care via medication adherence through the application of behaviour change theory delivered through digital and human components.

Declarations

List of abbreviations

CF – Cystic Fibrosis
CFHH – CFHealthHub complex intervention
CFQ-R – Cystic Fibrosis Questionnaire-Revised
CHAOS - Confusion, Hubbub, and Order Scale
COM- BMQ – The Capability Opportunity Motivation Behaviour Beliefs Questionnaire
EQ-5D-5L – EuroQol generic health status measure
FEV1 - Forced expiratory volume in 1 second
GAD-7 – General Anxiety Disorder 7-item
IVAB - Intravenous Antibiotics
MAD-3 Medication Adherence Data
MDT – Multi-Disciplinary Team
PAM-13 - Patient Activation Measure
PHQ-8 – Patient Health Questionnaire depression scale
PWCF – People With Cystic Fibrosis
RCT – Randomised Controlled Trial
SRBAI – Self-Report Behavioural Automaticity Index

For peer review only

For peer review only - http://bmjopen.bmj.com/site/about/guidelines.xhtml

Ethics, consent and permissions

Written informed consent was obtained prior to participation.

Ethical approval

The study received ethical approval from the London Brent Research Ethics Committee (16/LO/0356).

Consent for publication

In the consent form, participants signed a statement to confirm consent for publication of anonymised quotes, in reports, conference presentations and journal publications.

Availability of data and materials

Requests for further data not available in this publication can be directed at Sheffield Clinical Trials Research Unit. Email: ctru@sheffield.ac.uk Tel: 0114 222 0866

Competing interests

Martin Wildman received funding from Zambon and support from Philips Respironics for the early intervention development work. This has not had any direct influence on the feasibility study reported here. In addition, Martin Wildman has worked with Pari to carry out studies using the e-track. This has not had any direct influence on the feasibility study reported here. The University of Manchester software team received funding from Pari to create a medication reporting component within the CFHealthHub software. This has not had any direct influence on the feasibility study reported here.

The other authors declare that they have no competing interests.

Funding

This report presents independent research funded by the NIHR under its Grants for Applied Research Programme (Grant Reference Number RP-PG-1212-20015). The views and opinions expressed by authors in this publication are those of the authors and do not necessarily reflect those of the NHS, the NIHR, Medical Research Council (MRC), Central Commissioning Facility (CCF), the Programme Grants for Applied Research Programme, or the Department of Health and Social Care.

Authors' contributions

Daniel Hind (Assistant Director, CTRU), Sarah Drabble (Research Associate) and Laura Mandefield (Statistician), together produced the first draft of the report.

The following conceived of or designed the work: Martin Wildman (Consultant Respiratory Physician), Alicia O'Cathain (Professor of Health Services Research), Stephen Walters (Professor of Medical Statistics and Clinical Trials), Madelynne Arden (Professor of Health Psychology), Marlene Hutchings (Physiotherapist), Judy Bradley (Professor of

For peer review only - http://bmjopen.bmj.com/site/about/guidelines.xhtml

For peer review only

Physiotherapy), John Ainsworth (Professor of Health Informatics), Daniel Beever (Patient and Public Involvement Representative) and Daniel Hind.

The following were involved in the acquisition of data for the work: Julia Nightingale (Consultant in Respiratory Medicine), Mark Allenby (Consultant in Respiratory Medicine), Jane Dewar (Consultant in Respiratory Medicine), Sarah Drabble, Madelynne Arden, Chin Maguire, Hannah Cantrill, Alexander Scott (Research Associate), Sam Keating (Research Associate), Marlene Hutchings and Pauline Whelan (mHealth Applications Manager).

The following were involved in the analysis of data: Daniel Hind, Sarah Drabble, Alicia O'Cathain, Daniel Beever, Alexander Scott, Sam Keating, Madelynne Arden, Laura Mandefield, Simon Waterhouse (Data Manager).

Daniel Hind, Sarah Drabble, Madelynne Arden, Laura Mandefield, Simon Waterhouse, Chin Maguire, Hannah Cantrill, Louisa Robinson¸ Marlene Hutchings, Judy Bradley, Julia Nightingale, Mark Allenby, Jane Dewar, Daniel Beever, Stephen Walters, Alicia O'Cathain, Martin J Wildman, were involved in the interpretation of data for the work.

All authors were involved in the final approval of the version to be published.

All authors agree to be accountable for all aspects of the work in ensuring that questions related to the accuracy or integrity of any part of the work are appropriately investigated and resolved.

Acknowledgements

We would like to thank the trial participants who offered us their time. We gratefully acknowledge the clerical and other input of Helen Wakefield, Heather Dakin, Katie Shore and Louise Turner, trial support officers.

We would also like to thank Claire Oliver, Fiona Haynes and Lisa Evans for their commitment and hard work in delivering the intervention and supporting the study.

Professor Walters is a National Institute for Health Research (NIHR) Senior Investigator. The views expressed in this article are those of the author(s) and not necessarily those of the NHS, the NIHR, or the Department of Health and Social Care.

For peer review only - http://bmjopen.bmj.com/site/about/guidelines.xhtml

References

1 Mowat A. Why does cystic fibrosis display the prevalence and distribution observed in human populations? *Curr Pediatr Res* 2017;**21**:164–71.

2 Jeffery A, Charman S, Cosgriff R, *et al. UK Cystic Fibrosis Registry Annual Data Report 2016*. London: : Cystic Fibrosis Trust 2017.

3 Cystic Fibrosis Foundation. *Annual Data Report 2016 Cystic Fibrosis Foundation Patient Registry*. Bethesda, Maryland: : Cystic Fibrosis Foundation Patient 2017. https://www.cff.org/Research/Researcher-Resources/Patient-Registry/2016-Patient-Registry-Annual-Data-Report.pdf

4 European Cystic Fibrosis Society. *ECFS Patient Registry Annual Data Report*. Karup, Denmark: : European Cystic Fibrosis Society 2013.

5 Ahern S, Sims G, Tacey M, *et al. The Australian Cystic Fibrosis Data Registry Annual Report, 2015. Monash University, Department of Epidemiology and Preventive Medicine, October 2017, Report No 18.* Melbourne, Victoria: : Australian Cystic Fibrosis Data Registry 2017.

6 Cystic Fibrosis Canada. *The Canadian CF Registry 2016 Annual Data Report*. Toronto: : Cystic Fibrosis Canada 2017. http://www.cysticfibrosis.ca/uploads/2016 Registry Annual Data Report.pdf

7 Southern KW, Barker PM, Solis-Moya A, *et al.* Macrolide antibiotics for cystic fibrosis. In: Southern KW, ed. *Cochrane Database of Systematic Reviews*. Chichester, UK: : John Wiley & Sons, Ltd 2011. doi:10.1002/14651858.CD002203.pub3

8 Ramsey BW, Davies J, McElvaney NG, *et al.* A CFTR Potentiator in Patients with Cystic Fibrosis and the *G551D* Mutation. *N Engl J Med* 2011;**365**:1663–72. doi:10.1056/NEJMoa1105185

9 McCoy KS, Quittner AL, Oermann CM, *et al.* Inhaled aztreonam lysine for

For peer review only - http://bmjopen.bmj.com/site/about/guidelines.xhtml

chronic airway Pseudomonas aeruginosa in cystic fibrosis. *Am J Respir Crit Care Med* 2008;**178**:921–8.

10 Ryan G, Mukhopadhyay S, Singh M. Nebulised anti-pseudomonal antibiotics for cystic fibrosis. In: Ryan G, ed. *Cochrane Database of Systematic Reviews*. Chichester, UK: : John Wiley & Sons, Ltd 2003. doi:10.1002/14651858.CD001021

11 Jones AP, Wallis C. Dornase alfa for cystic fibrosis. In: Jones AP, ed. *Cochrane Database of Systematic Reviews*. Chichester, UK: : John Wiley & Sons, Ltd 2010. doi:10.1002/14651858.CD001127.pub2

12 Wark P, McDonald VM. Nebulised hypertonic saline for cystic fibrosis. In: Wark P, ed. *Cochrane Database of Systematic Reviews*. Chichester, UK: : John Wiley & Sons, Ltd 2009. doi:10.1002/14651858.CD001506.pub3

13 Ryan G, Singh M, Dwan K. Inhaled antibiotics for long-term therapy in cystic fibrosis. *Cochrane database Syst Rev* 2011;:CD001021. doi:10.1002/14651858.CD001021.pub2

14 Eakin MN, Bilderback A, Boyle MP, *et al.* Longitudinal association between medication adherence and lung health in people with cystic fibrosis. *J Cyst Fibros* 2011;**10**:258. doi:10.1016/j.jcf.2011.03.005

15 Briesacher BA, Quittner AL, Saiman L, *et al.* Adherence with tobramycin inhaled solution and health care utilization. *BMC Pulm Med* 2011;**11**:5. doi:10.1186/1471-2466-11-5

16 Smyth A, Lewis S, Bertenshaw C, *et al.* Case-control study of acute renal failure in patients with cystic fibrosis in the UK. *Thorax* 2008;**63**:532–5. doi:10.1136/thx.2007.088757

17 Mayer-Hamblett N, Rosenfeld M, Emerson J, *et al.* Developing Cystic Fibrosis

For peer review only

For peer review only - http://bmjopen.bmj.com/site/about/guidelines.xhtml

Lung Transplant Referral Criteria Using Predictors of 2-Year Mortality. *Am J Respir Crit Care Med* 2002;**166**:1550–5. doi:10.1164/rccm.200202-087OC

18 Shiers D, Rafi I, Cooper S, *et al.* 2014 update (with acknowledgement to the late Helen Lester for her contribution to the original 2012 version) Positive Cardiometabolic Health Resource: an intervention framework for patients with psychosis and schizophrenia. *R Coll Psychiatr* 2014;:London.

19 Quittner AL, Zhang J, Marynchenko M, *et al.* Pulmonary medication adherence and health-care use in cystic fibrosis. *Chest* 2014;**146**:142–51. doi:10.1378/chest.13-1926

20 Narayanan S, Mainz JG, Gala S, *et al.* Adherence to therapies in cystic fibrosis: a targeted literature review. *Expert Rev Respir Med* 2017;**11**:129–45. doi:10.1080/17476348.2017.1280399

21 McManus P. Lead Pharmacist - specialised services (South Yorkshire and Bassetlaw) NHS England.

22 McNeil S. Personal communication from Stephanie McNeil, Chief Statistician of UK CF Registry.

23 Daniels T, Goodacre L, Sutton C, *et al.* Accurate assessment of adherence: self-report and clinician report vs electronic monitoring of nebulizers. *Chest* 2011;**140**:425–32. doi:10.1378/chest.09-3074

24 Hoo Z, Curley R, Walters S, *et al.* Exploring the implications of different approaches to estimate centre-level adherence using objective adherence data in an adult cystic fibrosis centre - a retrospective observational study. *J Cyst Fibros* 2020;**19**:162–7. doi:10.1016/j.jcf.2019.10.008

25 Horne RW, Barber N, Elliot R, *et al.* Concordance, adherance and compliance in medicine taking: A conceptual map and research priorities. National Co-

For peer review only

For peer review only - http://bmjopen.bmj.com/site/about/guidelines.xhtml

ordinating Centre for NHS Service Delivery and Organisational R&D (NCCSDO). 2005.

26 James Lind Alliance. Priority Setting Partnership: Cystic Fibrosis. 2017.http://www.jla.nihr.ac.uk/priority-setting-partnerships/cystic-fibrosis/ (accessed 2 Jun 2017).

27 Clegg D, Barker R. *Case Method Fast-Track: A RAD Approach*. Boston, MA: : Addison-Wesley 1994.

28 Bugge C, Williams B, Hagen S, *et al.* A process for Decision-making after Pilot and feasibility Trials (ADePT): development following a feasibility study of a complex intervention for pelvic organ prolapse. *Trials* 2013;**14**:353. doi:10.1186/1745-6215-14-353

29 Hind D, Drabble SJ, Arden MA, *et al.* Supporting medication adherence for adults with cystic fibrosis: a randomised feasibility study. *BMC Pulm Med* 2019;**19**:77. doi:10.1186/s12890-019-0834-6

30 Cherryholmes CH. Notes on Pragmatism and Scientific Realism. *Educ Res* 1992;**21**:13–7. doi:10.3102/0013189X021006013

31 Yin RK. *Case Study Research: Design and Methods*. London: : Sage 2014.

32 Ritchie J, Spencer L. Qualitative data analysis for applied policy research. In: Bryman A, Burgess RG, eds. *Analysing qualitative data*. Routledge 1994. 173–94.

33 Cane J, O'Connor D, Michie S. Validation of the theoretical domains framework for use in behaviour change and implementation research. *Implement Sci* 2012;**7**:37. doi:10.1186/1748-5908-7-37

34 Linnan L, Steckler A. Process evaluation for public health interventions and research: an overview. In: Linnan L, Steckler A, eds. *Process evaluation for*

For peer review only

For peer review only - http://bmjopen.bmj.com/site/about/guidelines.xhtml

public health interventions and research. San Francisco: : Jossey-Bass 2002. 1–23.http://media.johnwiley.com.au/product_data/excerpt/66/07879597/0787959766.pdf (accessed 6 May 2014).

35 Farmer T, Robinson K, Elliott SJ, *et al.* Developing and implementing a triangulation protocol for qualitative health research. *Qual Health Res* 2006;**16**:377–94. doi:10.1177/1049732305285708

36 Yin RK. Analyzing Case Study Evidence: How to Start Your Analysis , Your Analytic Choices , and How They Work. In: *Case Study Research: Design and Methods*. London: : Sage 2014. 133–76. doi:10.1111/bjd.13028

37 Guetterman TC, Fetters MD, Creswell JW. Integrating quantitative and qualitative results in health science mixed methods research through joint displays. *Ann Fam Med* 2015;**13**:554–61. doi:10.1370/afm.1865

38 Fetters MD, Curry LA, Creswell JW. Achieving integration in mixed methods designs - Principles and practices. *Health Serv Res* 2013;**48**:2134–56. doi:10.1111/1475-6773.12117

39 Simons H. Case Study Research: In-Depth Understanding in Context. In: Leavy P, ed. *The Oxford Handbook of Qualitative Research*. Oxford: : Oxford University Press 2014. 455–70.

40 Perla RJ, Provost LP, Murray SK. The run chart: a simple analytical tool for learning from variation in healthcare processes. *BMJ Qual Saf* 2011;**20**:46–51. doi:10.1136/bmjqs.2009.037895

41 Bandura A. Social Cognitive Theory: An Agentic Perspective. *Annu Rev Psychol* 2001;**52**:1–26. doi:10.1146/annurev.psych.52.1.1

42 Carver CS, Scheier MF. Control theory: a useful conceptual framework for

For peer review only

For peer review only - http://bmjopen.bmj.com/site/about/guidelines.xhtml

personality-social, clinical, and health psychology. *Psychol Bull* 1982;**92**:111–35. doi:10.1037/0033-2909.92.1.111

43 Ho YC. Abduction ? Deduction ? Induction ? Is there a Logic of Exploratory Data Analysis ? *Proc Annu Meet Am Educ Res Assoc* 1994;:28. doi:http://citeseerx.ist.psu.edu/viewdoc/download?doi=10.1.1.135.3507&rep=rep1&type=pdf

44 Peirce CS. How to make our ideas clear. *Pop Sci Mon* 1878;**12**:286–302.

45 Swedberg R. Before theory comes theorizing or how to make social science more interesting. *Br J Sociol* 2016;**67**. doi:10.1111/1468-4446.12184

46 Timmermans S, Tavory I. Theory Construction in Qualitative Research: From Grounded Theory to Abductive Analysis. *Sociol Theory* 2012;**30**:167–86. doi:10.1177/0735275112457914

47 Miles MB, Huberman AM, Saldaña J. Methods of Ordering. In: *Qualitative Data Analysis: A Methods Sourcebook*. London: 2014. 193–220.

48 Hoo ZH, Boote J, Wildman MJ, *et al.* Determinants of objective adherence to nebulised medications among adults with cystic fibrosis: an exploratory mixed methods study comparing low and high adherers. *Heal Psychol Behav Med* 2017;**5**:299–316. doi:10.1080/21642850.2017.1338958

49 Wong MD, Sarkisian C a., Davis C, *et al.* The Association Between Life Chaos, Health Care Use, and Health Status Among HIV-Infected Persons. *J Gen Intern Med* 2007;**22**:1286–91. doi:10.1007/s11606-007-0265-6

50 Horne R, Weinman J, Hankins M. The beliefs about medicines questionnaire: The development and evaluation of a new method for assessing the cognitive representation of medication. *Psychol Health* 1999;**14**:1–24. doi:10.1080/08870449908407311

For peer review only

For peer review only - http://bmjopen.bmj.com/site/about/guidelines.xhtml

51 Bittner K, Spence I. *Use Case Modeling*. Boston, MA: : Addison-Wesley Professional 2002.

52 Craig Larman. *Agile and Iterative Development: A Manager's Guide*. Boston, MA: : Addison-Wesley 2004.

53 Govan JR. Infection control in cystic fibrosis: methicillin-resistant Staphylococcus aureus, Pseudomonas aeruginosa and the Burkholderia cepacia complex. *J R Soc Med* 2000;**93 Suppl 3**:40–5.http://www.ncbi.nlm.nih.gov/pubmed/10911818

54 Wildman MJ, Hoo ZH. Moving cystic fibrosis care from rescue to prevention by embedding adherence measurement in routine care. *Paediatr Respir Rev* 2014;**15**:16–8. doi:10.1016/j.prrv.2014.04.007

55 Schattner A. Predictors of Adherence to Inhaled Medications. *J Gen Intern Med* 2013;**28**:758–758. doi:10.1007/s11606-013-2384-6

56 Battaglia M, Atkinson MA. The Streetlight Effect in Type 1 Diabetes. *Diabetes* 2015;**64**:1081–90. doi:10.2337/db14-1208

57 Kaplan A. *The Conduct of Inquiry: Methodology for Behavioral Science*. New Brunswick, N.J.: : Transaction Publishers 1964.

58 Tversky A, Kahneman D. Availability: A heuristic for judging frequency and probability. *Cogn Psychol* 1973;**5**:207–32. doi:10.1016/0010-0285(73)90033-9

59 Flume PA, Clancy JP, Retsch-Bogart GZ, *et al.* Continuous alternating inhaled antibiotics for chronic pseudomonal infection in cystic fibrosis. *J Cyst Fibros* 2016;**15**:809–15. doi:10.1016/j.jcf.2016.05.001

60 Michie S, Atkins L, West R. *The Behaviour Change Wheel: A Guide To Designing Interventions*. Sutton: : Silverback Publishing 2014.

61 National Institute for Health and Care Excellence. *Medicines optimisation: the*

For peer review only

For peer review only - http://bmjopen.bmj.com/site/about/guidelines.xhtml

safe and effective use of medicines to enable best possible outcomes (NG5). London: : NICE 2015.

62 Carver CS, Scheier MF. *On the self-regulation of behavior*. Cambridge: : Cambridge University Press 2001.

63 Yardley L, Spring BJ, Riper H, *et al.* Understanding and Promoting Effective Engagement With Digital Behavior Change Interventions. *Am J Prev Med* 2016;**51**:833–42. doi:10.1016/j.amepre.2016.06.015

64 Allemann SS, Nieuwlaat R, van den Bemt BJF, *et al.* Matching Adherence Interventions to Patient Determinants Using the Theoretical Domains Framework. *Front Pharmacol* 2016;**7**:429. doi:10.3389/fphar.2016.00429

65 Michie S, Yardley L, West R, *et al.* Developing and evaluating digital interventions to promote behavior change in health and health care: Recommendations resulting from an international workshop. *J Med Internet Res* 2017;**19**. doi:10.2196/jmir.7126

66 Murray E, Hekler EB, Andersson G, *et al.* Evaluating Digital Health Interventions: Key Questions and Approaches. *Am J Prev Med* 2016;**51**:843–51. doi:10.1016/j.amepre.2016.06.008

67 Car J, Tan WS, Huang Z, *et al.* eHealth in the future of medications management: personalisation, monitoring and adherence. *BMC Med* 2017;**15**:73. doi:10.1186/s12916-017-0838-0

68 Baumel A, Birnbaum ML, Sucala M. A Systematic Review and Taxonomy of Published Quality Criteria Related to the Evaluation of User-Facing eHealth Programs. *J Med Syst* 2017;**41**:128. doi:10.1007/s10916-017-0776-6

69 Ryan C, Bergin M, Wells JS. Theoretical Perspectives of Adherence to Web-Based Interventions: a Scoping Review. *Int J Behav Med* Published Online

For peer review only

For peer review only - http://bmjopen.bmj.com/site/about/guidelines.xhtml

First: 20 July 2017. doi:10.1007/s12529-017-9678-8

70 Van Gemert-Pijnen JE, Kelders SM, Bohlmeijer ET. Understanding the Usage of Content in a Mental Health Intervention for Depression: An Analysis of Log Data. *J Med Internet Res* 2014;**16**:e27. doi:10.2196/jmir.2991

71 Strecher V, McClure J, Alexander G, *et al.* The Role of Engagement in a Tailored Web-Based Smoking Cessation Program: Randomized Controlled Trial. *J Med Internet Res* 2008;**10**:e36. doi:10.2196/jmir.1002

72 Danaher BG, Seeley JR. Methodological Issues in Research on Web-Based Behavioral Interventions. *Ann Behav Med* 2009;**38**:28–39. doi:10.1007/s12160-009-9129-0

73 Ainsworth B, Steele M, Stuart B, *et al.* Using an Analysis of Behavior Change to Inform Effective Digital Intervention Design: How Did the PRIMIT Website Change Hand Hygiene Behavior Across 8993 Users? *Ann Behav Med* 2017;**51**:423–31. doi:10.1007/s12160-016-9866-9

74 Hawe P. Lessons from complex interventions to improve health. *Annu Rev Public Health* 2015;**36**:307–23. doi:10.1146/annurev-publhealth-031912-114421

75 Sherman DAK, Nelson LD, Steele CM. Do Messages about Health Risks Threaten the Self? Increasing the Acceptance of Threatening Health Messages Via Self-Affirmation. *Personal Soc Psychol Bull* 2000;**26**:1046–58. doi:10.1177/01461672002611003

76 Perski O, Blandford A, West R, *et al.* Conceptualising engagement with digital behaviour change interventions: a systematic review using principles from critical interpretive synthesis. *Transl Behav Med* 2017;**7**:254–67. doi:10.1007/s13142-016-0453-1

For peer review only

77 Frazee SG, Muzina DJ, Nease RF. Strategies to Overcome Medication Nonadherence. *JAMA* 2014;**311**:1693. doi:10.1001/jama.2014.1760

78 Mohammed MA, Moles RJ, Chen TF. Medication-related burden and patients' lived experience with medicine: a systematic review and metasynthesis of qualitative studies. *BMJ Open* 2016;**6**:e010035. doi:10.1136/bmjopen-2015-010035

79 Huygens MWJ, Vermeulen J, Swinkels ICS, *et al.* Expectations and needs of patients with a chronic disease toward self-management and eHealth for self-management purposes. *BMC Health Serv Res* 2016;**16**:232. doi:10.1186/s12913-016-1484-5

80 Kelders SM, Oinas-Kukkonen H, Oörni A, *et al.* Health Behavior Change Support Systems as a research discipline; a viewpoint. *Int J Med Inform* Published Online First: 2016. doi:10.1016/j.ijmedinf.2016.06.022

81 Rathbone AP, Todd A, Jamie K, *et al.* A systematic review and thematic synthesis of patients' experience of medicines adherence. *Res Soc Adm Pharm* Published Online First: 2016. doi:10.1016/j.sapharm.2016.06.004

82 O'Connor S, Hanlon P, O'Donnell CA, *et al.* Understanding factors affecting patient and public engagement and recruitment to digital health interventions: a systematic review of qualitative studies. *BMC Med Inform Decis Mak* 2016;**16**:120. doi:10.1186/s12911-016-0359-3

83 Bond CM. Adherence: the Holy Grail? *BMJ Qual Saf* 2016;**25**:736–8. doi:10.1136/bmjqs-2015-004886

84 Cammidge SL, Duff AJA, Latchford GJ, *et al.* When Women with Cystic Fibrosis Become Mothers: Psychosocial Impact and Adjustments. *Pulm Med* 2016;**2016**:9458980. doi:10.1155/2016/9458980

For peer review only

For peer review only - http://bmjopen.bmj.com/site/about/guidelines.xhtml

85 Powell KR. Patient-Perceived Facilitators of and Barriers to Electronic Portal Use. *CIN Comput Informatics, Nurs* 2017;**35**:565–73. doi:10.1097/CIN.0000000000000377

86 Curtis LM, Mullen RJ, Russell A, *et al.* An efficacy trial of an electronic health record-based strategy to inform patients on safe medication use: The role of written and spoken communication. *Patient Educ Couns* 2016;**99**:1489–95. doi:10.1016/j.pec.2016.07.004

87 Morton K, Dennison L, May C, *et al.* Using digital interventions for self-management of chronic physical health conditions: A meta-ethnography review of published studies. *Patient Educ Couns* 2017;**100**:616–35. doi:10.1016/j.pec.2016.10.019

88 Hanlon P, Daines L, Campbell C, *et al.* Telehealth Interventions to Support Self-Management of Long-Term Conditions: A Systematic Metareview of Diabetes, Heart Failure, Asthma, Chronic Obstructive Pulmonary Disease, and Cancer. *J Med Internet Res* 2017;**19**:e172. doi:10.2196/jmir.6688

89 Oussedik, E., Foy, C.G, Masicampo, E.J., Kammrath, L.K, Anderson, R.E, Feldman SR (in press). Accountabiliy: a missing construct in models of adherence behavior and in clinical practice. *Patient Prefer Adherence* 2017;:1285–94. doi:10.2147/PPA.S135895

90 O'Donohoe R, Fullen BM. Adherence of subjects with cystic fibrosis to their home program: a systematic review. *Respir Care* 2014;**59**:1731–46. doi:10.4187/respcare.02990

91 Sawicki GS, Goldman DP, Chan W, *et al.* Patient preference for treatment administration in cystic fibrosis. *Am J Pharm Benefits* 2015;**7**:174–81.

92 Phillips LA, Cohen J, Burns E, *et al.* Self-management of chronic illness: the

For peer review only

role of 'habit' versus reflective factors in exercise and medication adherence. *J Behav Med* 2016;**39**:1076–91. doi:10.1007/s10865-016-9732-z

93 Lally P, van Jaarsveld CHM, Potts HWW, *et al.* How are habits formed: Modelling habit formation in the real world. *Eur J Soc Psychol* 2010;**40**:998–1009. doi:10.1002/ejsp.674

94 Arden MA, Drabble S, O'Cathain A, *et al.* Adherence to medication in adults with Cystic Fibrosis: An investigation using objective adherence data and the Theoretical Domains Framework. *Br J Health Psychol* Published Online First: March 2019. doi:10.1111/bjhp.12357

95 Hoo ZH, Gardner B, Arden MA, *et al.* Role of habit in treatment adherence among adults with cystic fibrosis. *Thorax* 2019;**74**:197–9. doi:10.1136/thoraxjnl-2017-211453

96 Conn VS, Ruppar TM. Medication adherence outcomes of 771 intervention trials: Systematic review and meta-analysis. *Prev Med (Baltim)* 2017;**99**:269–76. doi:10.1016/j.ypmed.2017.03.008

97 Goldzweig CL, Orshansky G, Paige NM, *et al.* Electronic patient portals: evidence on health outcomes, satisfaction, efficiency, and attitudes: a systematic review. *Ann Intern Med* 2013;**159**:677–87. doi:10.7326/0003-4819-159-10-201311190-00006

98 Lau AY, Arguel A, Dennis S, *et al.* "Why Didn't it Work?" Lessons From a Randomized Controlled Trial of a Web-based Personally Controlled Health Management System for Adults with Asthma. *J Med Internet Res* 2015;**17**:e283. doi:10.2196/jmir.4734

99 Kruse CS, Kothman K, Anerobi K, *et al.* Adoption Factors of the Electronic Health Record: A Systematic Review. *JMIR Med informatics* 2016;**4**:e19.

For peer review only

For peer review only - http://bmjopen.bmj.com/site/about/guidelines.xhtml

doi:10.2196/medinform.5525

100 Greenhalgh T, Swinglehurst D, Stones R. Rethinking resistance to 'big IT': a sociological study of why and when healthcare staff do not use nationally mandated information and communication technologies. *Heal Serv Deliv Res* 2014;**2**:1–86. doi:10.3310/hsdr02390

101 Ross J, Stevenson F, Lau R, *et al.* Factors that influence the implementation of e-health: a systematic review of systematic reviews (an update). *Implement Sci* 2016;**11**:146. doi:10.1186/s13012-016-0510-7

102 Hennemann S, Beutel ME, Zwerenz R. Ready for eHealth? Health Professionals' Acceptance and Adoption of eHealth Interventions in Inpatient Routine Care. *J Health Commun* 2017;**22**:274–84. doi:10.1080/10810730.2017.1284286

103 Harris M, Lawn SJ, Morello A, *et al.* Practice change in chronic conditions care: an appraisal of theories. *BMC Health Serv Res* 2017;**17**:170. doi:10.1186/s12913-017-2102-x

104 Ovretveit J, Leviton L, Parry G. Increasing the generalisability of improvement research with an improvement replication programme. *BMJ Qual Saf* 2011;**20**:i87–91. doi:10.1136/bmjqs.2010.046342

105 Eikey E V, Reddy MC, Kuziemsky CE. Examining the role of collaboration in studies of health information technologies in biomedical informatics: A systematic review of 25 years of research. *J Biomed Inform* 2015;**57**:263–77. doi:10.1016/j.jbi.2015.08.006

106 Relton C, Torgerson D, O'Cathain A, *et al.* Rethinking pragmatic randomised controlled trials: introducing the 'cohort multiple randomised controlled trial' design. *BMJ* 2010;**340**:c1066. doi:10.1136/bmj.c1066

For peer review only

For peer review only - http://bmjopen.bmj.com/site/about/guidelines.xhtml

107 Young-Afat DA, Verkooijen HAM, van Gils CH, *et al.* Staged-informed Consent in the Cohort Multiple Randomized Controlled Trial Design. *Epidemiology* 2016;**27**:389–92. doi:10.1097/EDE.0000000000000435

108 Retsch-Bogart GZ, Van Dalfsen JM, Marshall BC, *et al.* Highly effective cystic fibrosis clinical research teams: critical success factors. *J Gen Intern Med* 2014;**29 Suppl 3**:S714-23. doi:10.1007/s11606-014-2896-8

109 Hibbard JH, Mahoney ER, Stockard J, *et al.* Development and testing of a short form of the patient activation measure. *Health Serv Res* 2005;**40**:1918–30. doi:10.1111/j.1475-6773.2005.00438.x

110 Verplanken BAS, Ell OR, Orbell S. Reflections on past behavior: A self-report index of habit strength. *J Appl Soc Psychol* 2003;**33**:1313–30. doi:10.1111/j.1559-1816.2003.tb01951.x

…

For peer review only

Tables

Table 1 - Quantitative data contributing to the understanding of logic model constructs

#	Logic model column / construct	Quantitative
	**INPUTS**	
3	Prescription data	CFHH; problems documented in implementation log.
7	COM-BMQ questionnaire responses	Capability Opportunity Motivation Behaviour Beliefs Questionnaire (COM-BMQ), incorporating the Beliefs about Medicines Questionnaire (Nebuliser adherence)[50], one additional belief item, one intention item, one confidence item, and a list of barriers
9	Interventionist training programme	Structured questionnaire on interventionist confidence after training programme.
11	Competency/Fidelity assessment	Structured instrument for the assessment of interventionist competence.
	**ENGAGEMENT**	
13	Clinicians accessing adherence data*	CFHH click analytics.
14	Adherence data tracking	CFHH click analytics.
15	Participant accessing CFHealthHub	CFHH click analytics.

For peer review only

For peer review only - http://bmjopen.bmj.com/site/about/guidelines.xhtml

17	CFHealthHub Intervention sessions delivered according to Manual (Fidelity)	Project-specific structured fidelity assessment instrument.
18	Initial session, and then review at each clinic visit	CFHH click analytics.
	**ACTIVITIES**	
	Intervention components for all participants	
20	Self-monitoring adherence	CFHH click analytics.
21	Tailored education about treatment	CFHH click analytics.
22	Tailored patient stories (videos)	CFHH click analytics.
	Intervention components for those with adequate motivation	
23	Personalised goal-setting	CFHH click analytics.
24	Goal review	CFHH click analytics.
25	Personalised action plan	CFHH click analytics.
26	Tailored problem-solving	CFHH click analytics.
	**IMMEDIATE OUTCOMES**	
	For all participants	
28	Acute awareness of adherence /	Subjective adherence single question (self-

For peer review only - http://bmjopen.bmj.com/site/about/guidelines.xhtml

	increased Motivation	report estimate of adherence as a percentage); COM-BMQ.
29	Increased necessity and decreased concern	COM-BMQ and Patient Activation Measure (PAM-13[109])
30	Increased self-efficacy / Motivation	COM-BMQ single question about confidence to adhere; PAM-13.
	For those with adequate motivation	
31	Increased self-efficacy/ Motivation	COM-BMQ single question about confidence to adhere; PAM-13.
32	Increased habit / reduced chaos	Self-Report Behavioural Automaticity Index (SRBAI) automaticity-specific subscale of the Self Report Habit index to capture habit-based behaviour patterns[110]; Confusion, Hubbub, and Order Scale (CHAOS 6-item): measure of life chaos[49].
33	Reduced barriers	No change in the group averages for The Beliefs about Medicines Questionnaire - specific (Nebuliser adherence) (BMQ 21-item[50])
	**INTERMEDIATE OUTCOMES**	
35	Increased adherence	Nebuliser data (CFHH)

For peer review only - http://bmjopen.bmj.com/site/about/guidelines.xhtml

For peer review only

For peer review only - http://bmjopen.bmj.com/site/about/guidelines.xhtml

Figures

Figure 1 - Logic model

Figure 2 - The digital platform

Figure 3 – Objective versus subjective adherence at 5 (+/- 1) months stratified by adherence

Additional files

Additional file 01 – Participant adherence run charts for fourteen case studies

Additional file 02 – Study protocol and interview guides

Additional file 03 – Quantitative results from process evaluation

Additional file 04 – Joint display table

Additional file 05 – Case-ordered descriptive matrix for fourteen case studies

Additional file 06 – Statistical methods, outcomes and estimation

Additional file 07 – Changes to intervention procedures

For peer review only

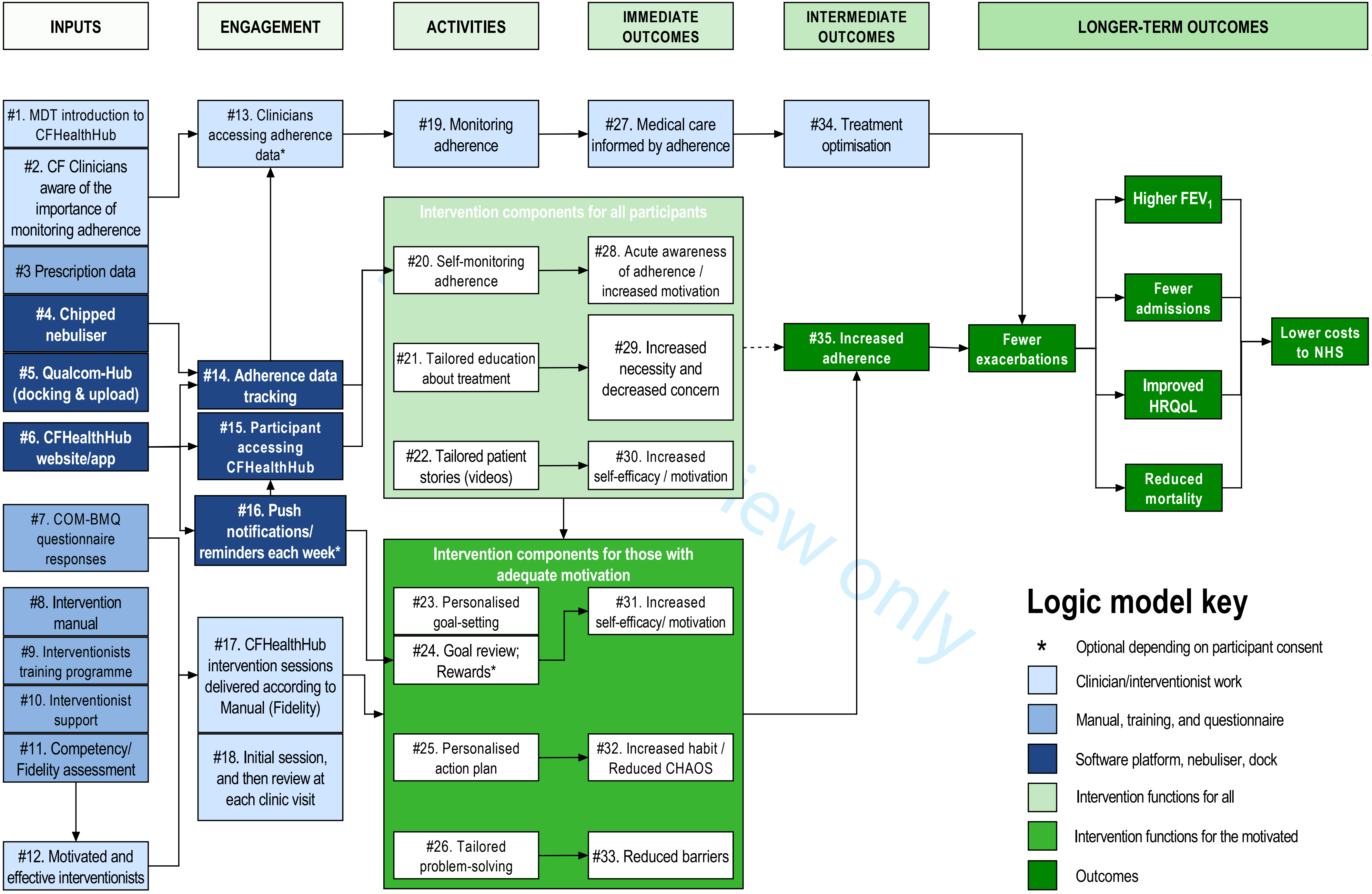
INPUTS
ENGAGEMENT
ACTIVITIES
IMMEDIATE OUTCOMES
INTERMEDIATE OUTCOMES
LONGER-TERM OUTCOMES
#1. MDT introduction to CFHealthHub
#2. CF Clinicians aware of the importance of monitoring adherence
#3 Prescription data
#4. Chipped nebuliser
#5. Qualcom-Hub (docking & upload)
#6. CFHealthHub website/app
#7. COM-BMQ questionnaire responses
#8. Intervention manual
#9. Interventionists training programme
#10. Interventionist support
#11. Competency/ Fidelity assessment
#12. Motivated and effective interventionists
#13. Clinicians accessing adherence data*
#14. Adherence data tracking
#15. Participant accessing CFHealthHub
#16. Push notifications/ reminders each week*
#17. CFHealthHub intervention sessions delivered according to Manual (Fidelity)
#18. Initial session, and then review at each clinic visit
#19. Monitoring adherence
Intervention components for all participants
#20. Self-monitoring adherence
#21. Tailored education about treatment
#22. Tailored patient stories (videos)
Intervention components for those with adequate motivation
#23. Personalised goal-setting
#24. Goal review; Rewards*
#25. Personalised action plan
#26. Tailored problem-solving
#27. Medical care informed by adherence
#28. Acute awareness of adherence / increased motivation
#29. Increased necessity and decreased concern
#30. Increased self-efficacy / motivation
#31. Increased self-efficacy/ motivation
#32. Increased habit / Reduced CHAOS
#33. Reduced barriers
#34. Treatment optimisation
#35. Increased adherence
Fewer exacerbations
Higher FEV$_1$
Fewer admissions
Improved HRQoL
Reduced mortality
Lower costs to NHS
Logic model key
* Optional depending on participant consent
Clinician/interventionist work
Manual, training, and questionnaire
Software platform, nebuliser, dock
Intervention functions for all
Intervention functions for the motivated
Outcomes

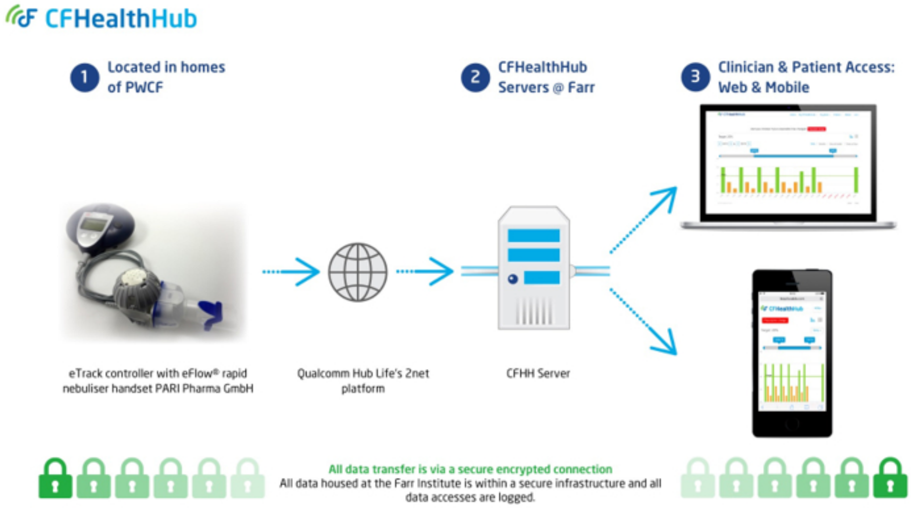

The digital platform

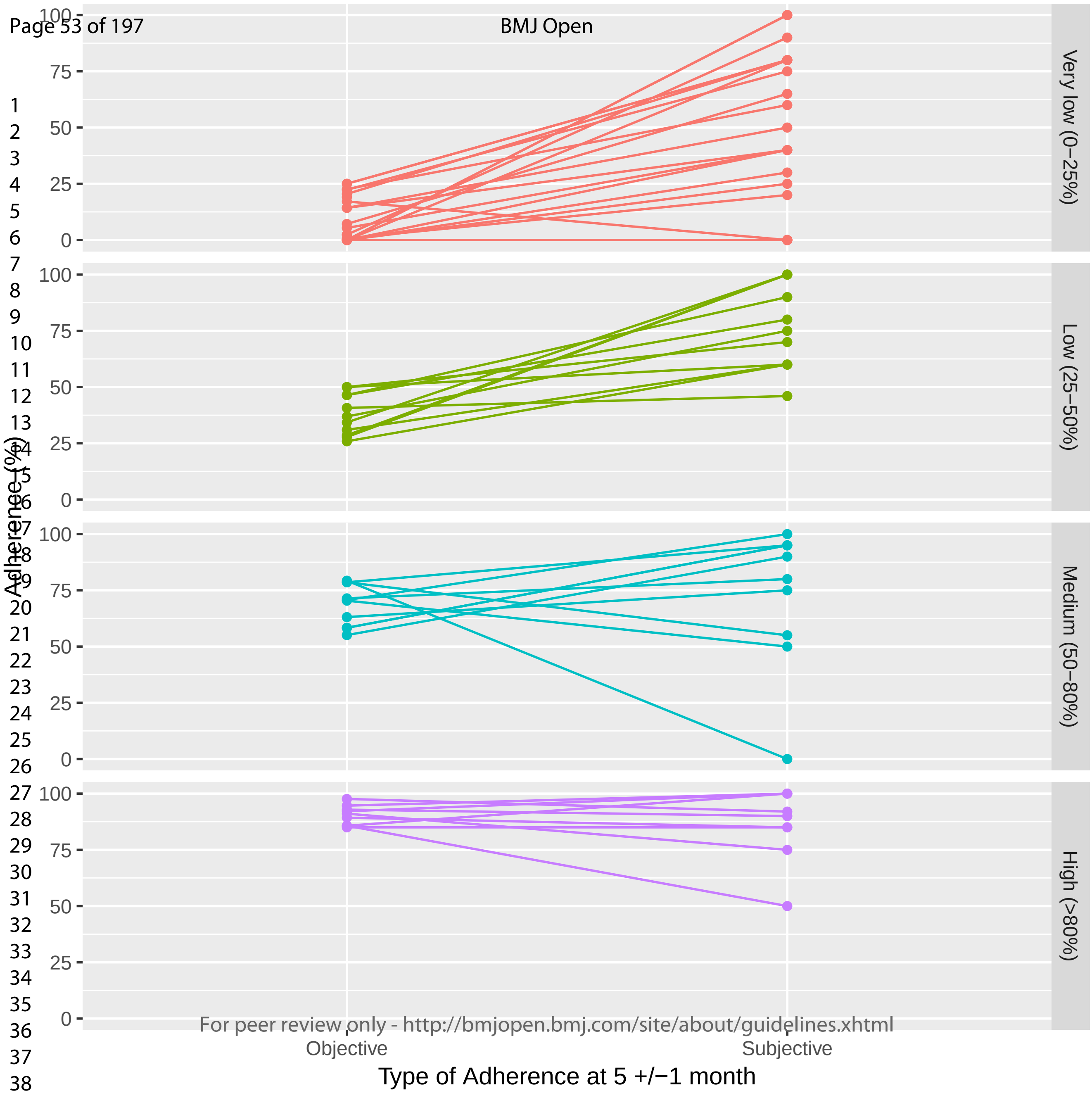
Very low (0–25%)
Low (25–50%)
Medium (50–80%)
High (>80%)
Adherence (%)
0
25
50
75
100
Objective
Subjective
Type of Adherence at 5 +/−1 month

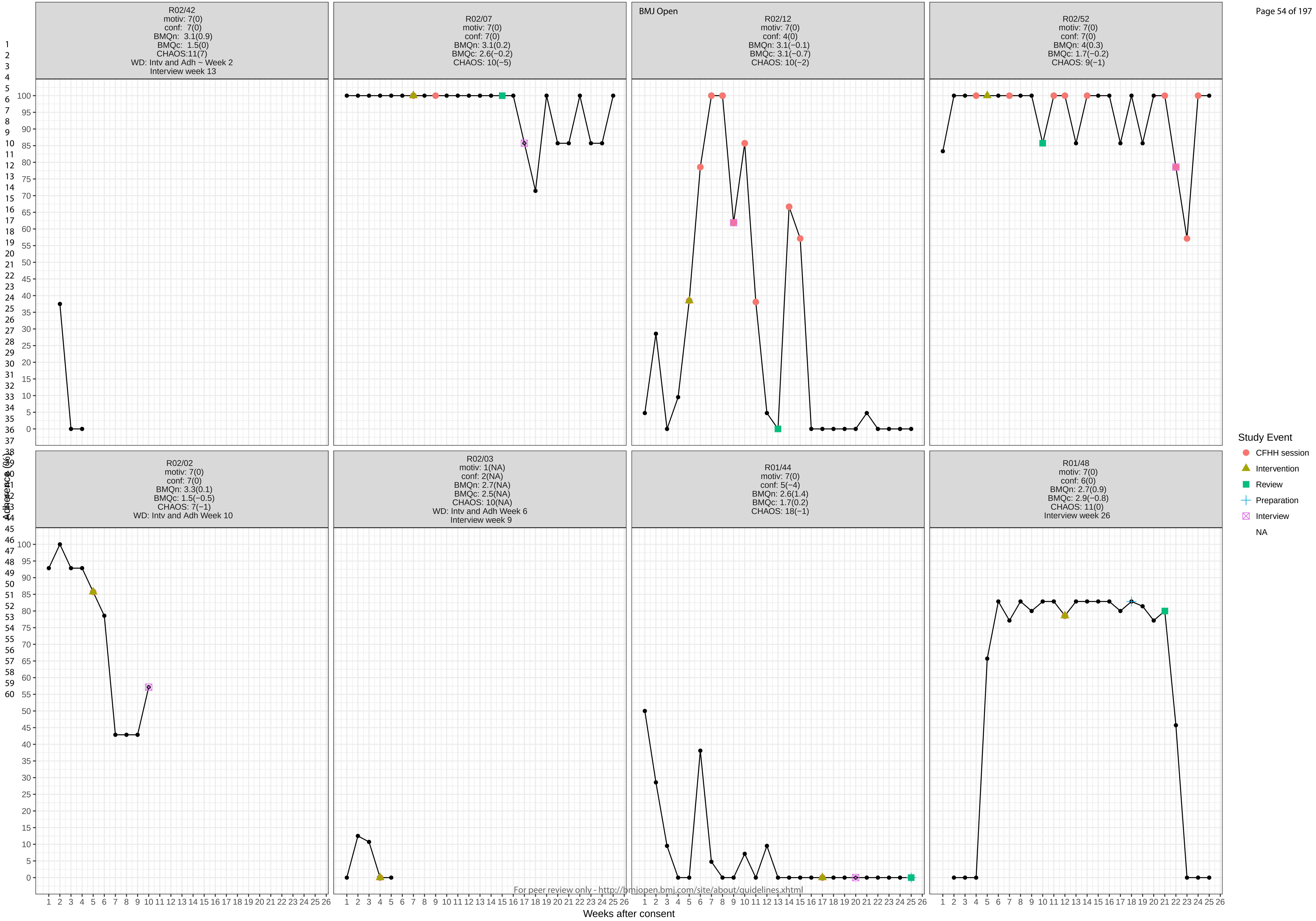
R02/42
motiv: 7(0)
conf: 7(0)
BMQn: 3.1(0.9)
BMQc: 1.5(0)
CHAOS:11(7)
WD: Intv and Adh ~ Week 2
Interview week 13
R02/07
motiv: 7(0)
conf: 7(0)
BMQn: 3.1(0.2)
BMQc: 2.6(−0.2)
CHAOS: 10(−5)
R02/12
motiv: 7(0)
conf: 4(0)
BMQn: 3.1(−0.1)
BMQc: 3.1(−0.7)
CHAOS: 10(−2)
R02/52
motiv: 7(0)
conf: 7(0)
BMQn: 4(0.3)
BMQc: 1.7(−0.2)
CHAOS: 9(−1)
R02/02
motiv: 7(0)
conf: 7(0)
BMQn: 3.3(0.1)
BMQc: 1.5(−0.5)
CHAOS: 7(−1)
WD: Intv and Adh Week 10
R02/03
motiv: 1(NA)
conf: 2(NA)
BMQn: 2.7(NA)
BMQc: 2.5(NA)
CHAOS: 10(NA)
WD: Intv and Adh Week 6
Interview week 9
R01/44
motiv: 7(0)
conf: 5(−4)
BMQn: 2.6(1.4)
BMQc: 1.7(0.2)
CHAOS: 18(−1)
R01/48
motiv: 7(0)
conf: 6(0)
BMQn: 2.7(0.9)
BMQc: 2.9(−0.8)
CHAOS: 11(0)
Interview week 26
Adherence (%)
Weeks after consent
Study Event
CFHH session
Intervention
Review
Preparation
Interview
NA

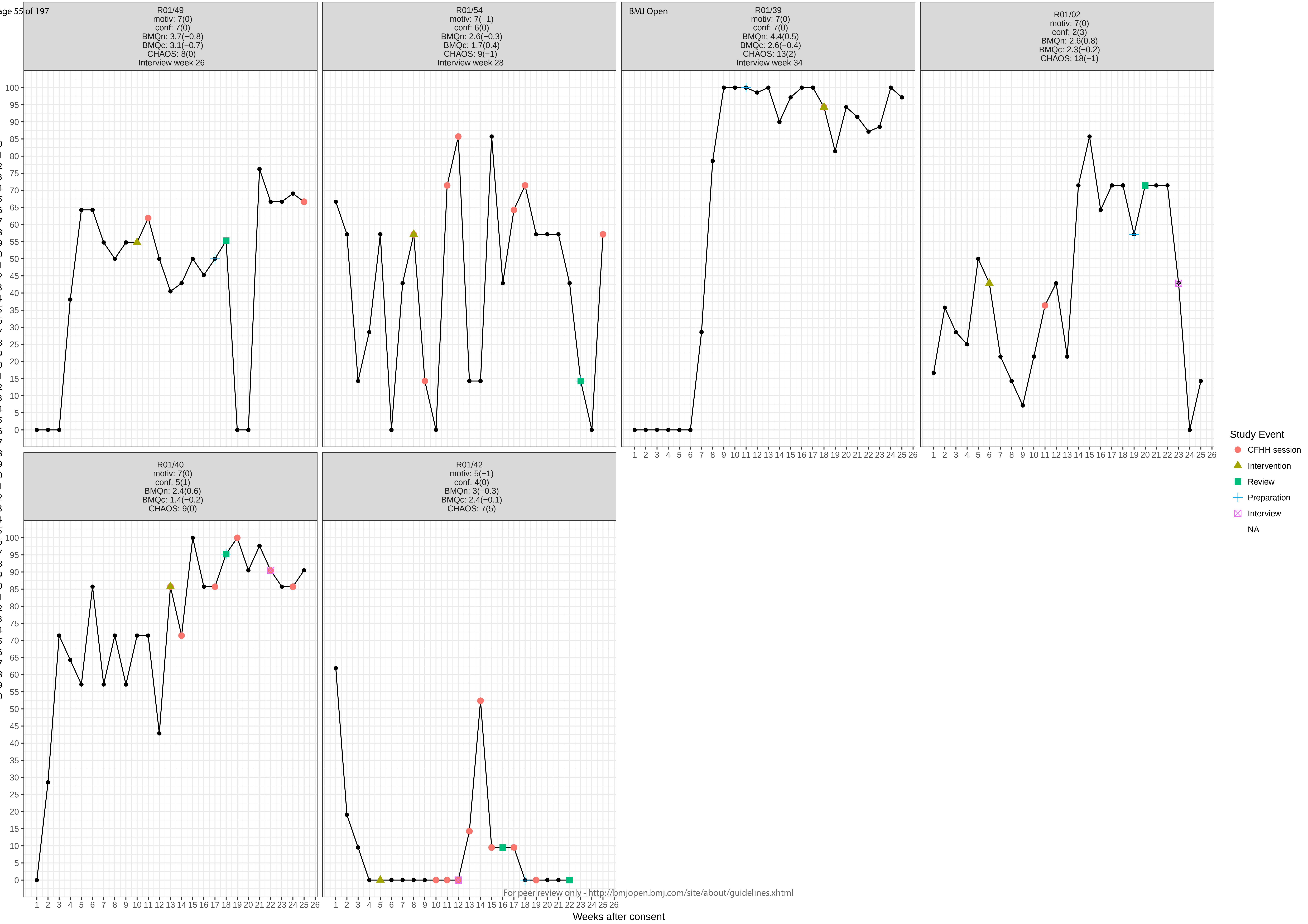
R01/49
motiv: 7(0)
conf: 7(0)
BMQn: 3.7(−0.8)
BMQc: 3.1(−0.7)
CHAOS: 8(0)
Interview week 26
R01/54
motiv: 7(−1)
conf: 6(0)
BMQn: 2.6(−0.3)
BMQc: 1.7(0.4)
CHAOS: 9(−1)
Interview week 28
R01/39
motiv: 7(0)
conf: 7(0)
BMQn: 4.4(0.5)
BMQc: 2.6(−0.4)
CHAOS: 13(2)
Interview week 34
R01/02
motiv: 7(0)
conf: 2(3)
BMQn: 2.6(0.8)
BMQc: 2.3(−0.2)
CHAOS: 18(−1)
R01/40
motiv: 7(0)
conf: 5(1)
BMQn: 2.4(0.6)
BMQc: 1.4(−0.2)
CHAOS: 9(0)
R01/42
motiv: 5(−1)
conf: 4(0)
BMQn: 3(−0.3)
BMQc: 2.4(−0.1)
CHAOS: 7(5)
Adherence (%)
Weeks after consent
Study Event
CFHH session
Intervention
Review
Preparation
Interview
NA

ACtiF

National Institute for Health Research

Sheffield Teaching Hospitals NHS Foundation Trust

The University Of Sheffield. Clinical Trials Research Unit.

Sheffield Hallam University

Development and evaluation of an intervention to support Adherence to treatment in adults with Cystic Fibrosis

A feasibility study comprised of an external pilot randomised controlled trial and process evaluation

RESEARCH PROTOCOL
V3.1 16Nov16

**Sheffield CTRU**	J13-002
**IRAS**	199775
**ISRCTN**	ISRCTN13076797
**Authorised by:**	Martin Wildman
	Alicia O'Cathain

For peer review only - http://bmjopen.bmj.com/site/about/guidelines.xhtml

Funding

This project was funded by the Programme Grants for Applied Research programme (RP-PG-1212-20015) and will be published in full in the NIHR Journals Library. This research presents independent research commissioned by the NIHR. The views and opinions expressed by authors in this publication are those of the authors and do not necessarily reflect those of the NHS, the NIHR, MRC, CCF, NETSCC, the Health Technology Assessment programme or the Department of Health.

Roles and responsibilities

Names, affiliations, and roles of protocol contributors

The following all contributed to drafting elements throughout the protocol: Martin Wildman, Consultant Respiratory Medic; Marlene Hutchings, Physiotherapist (Sheffield Teaching Hospitals NHS Foundation Trust); Madelynne Arden, Professor of Health Psychology (Sheffield Hallam University); Alicia O'Cathain, Professor of Health Services Research; Sarah Drabble, Research Associate, Stephen Walters (Professor of Medical Statistics and Clinical Trials); Paul Tappenden, Reader in Health Economics; Daniel Beever, Research Associate and Patient Representative; Pauline Wheelan mHealth Applications Manager (University of Manchester); Judy Bradley, Professor of Physiotherapy and Health Services Research; Hannah Cantrill (Research Associate); Chin Maguire (Study Manager); and, Daniel Hind, Assistant Director, Sheffield Clinical Trials Research Unit (University of Sheffield). MW and MA are guarantors of the intervention description; SW is the guarantor of the statistical design and analysis plan; DH is the guarantor of other elements of the pilot trial; AO'C is guarantor for the qualitative research components.

Sponsor

Sheffield Teaching Hospitals NHS Foundation Trust
D Floor
Clinical Research Office
Royal Hallamshire Hospital
Glossop Road
Sheffield
S10 2JF
Tel: (0114) 2256424
Fax: (0114)

Role of study sponsor and funders

Neither the funder nor the sponsor have had any role in study design, data collection and analysis, decision to publish, or preparation of manuscripts.

For peer review only - http://bmjopen.bmj.com/site/about/guidelines.xhtml

Key Contacts

Study Manager

Chin Maguire
Clinical Trials Research Unit
The University of Sheffield
School of Health and Related Research
30 Regent Street
Sheffield
S1 4DA
Tel: (0114) 222 0717
Fax: (0114) 222 0870
e-mail: c.maguire@sheffield.ac.uk

Chief Investigator

Dr Martin Wildman
Adult CF Centre
Northern General Hospital
Herries Road
Sheffield
S5 7AU

Tel: (0114) 2715212
Fax: (0114) 222 0870
e-mail: Martin.Wildman@sth.nhs.uk

Composition, roles, and responsibilities

Project Management Group

Name	Role	Institution
Dr Martin Wildman	Chief Investigator	Sheffield Teaching Hospitals NHS Foundation Trust
Professor Alicia O'Cathain	Chief Investigator	University of Sheffield
Dr Daniel Hind	CTRU Assistant Director	University of Sheffield
Chin Maguire	Study Manager	University of Sheffield
Hannah Cantrill	Research Assistant	University of Sheffield
Helen Wakefield	Trial Support Office	University of Sheffield
Dan Beever	PPI Lead	University of Sheffield
Dr Sarah Drabble	Qualitative Researcher	University of Sheffield
Professor Maddy Arden	Professor of Health Psychology	Sheffield Hallam University
Marlene Hutchings	Research Physiotherapist	Sheffield Teaching Hospitals NHS Foundation Trust
Professor Judy Bradley	Professor in Physiotherapy	Queens University Belfast

For peer review only - http://bmjopen.bmj.com/site/about/guidelines.xhtml

Independent Project Steering Committee

Name	Role	Institution
Professor Kathy Rowan	Independent Chair	London School of Hygiene and Tropical Medicine
Professor Sarah Garner	Independent member	The National Institute for Health and Care Excellence
Professor David Torgerson	Independent Member	University of York
Dr Lisa Hampson	Independent Statistician	Lancaster University
Dominic Kavanagh	PPI Representative	
Dr Martin Wildman	Chief Investigator	Sheffield Teaching Hospitals NHS Foundation Trust
Professor Alicia O'Cathain	Chief Investigator	University of Sheffield
Dr Daniel Hind	CTRU Assistant Director	University of Sheffield
Chin Maguire	Study Manager	University of Sheffield
Professor Maddy Arden	Professor of Health Psychology	Sheffield Hallam University

For peer review only - http://bmjopen.bmj.com/site/about/guidelines.xhtml

Contents

Funding 2
Names, affiliations, and roles of protocol contributors 2
Sponsor 2
Role of study sponsor and funders 2
Composition, roles, and responsibilities 3
Project Management Group 3
Independent Project Steering Committee 4
Contents 5
Figures 7
Tables 7
1. Lay summary 8
2. Introduction 9
2.1 Background 9
2.2 Rationale 9
3. Aim and objectives 11
3.1 Aims 11
3.2 Objectives 11
4. Design 12
5. Participants and study settings 13
5.1 Settings and locations where the data will be collected 13
5.2 Eligibility 13
5.2.1 Inclusion criteria for participants 13
5.2.2 Exclusion criteria for participants 13
5.2.3 Eligibility criteria for study centres 13
5.2.4 Eligibility criteria for interventionists 13
6. Interventions 14
6.1 Summary 14
6.2 Microchipped devices 15
6.2.1 The eTrack nebuliser system (Pari GmbH) 15
6.2.2 The Bi-neb AAD System from (Philips Healthcare) 15
6.3 Information technology infrastructure 15
6.3.1 The Qualcomm hub 15
6.3.2 CFHealthHub 15
6.3.3 The Bi-Neb data transfer system 16
6.4 The Behaviour Change Intervention (BCI) 17
6.4.1 Rationale and theory 17
6.4.2 Intervention providers 22
6.4.3 Materials 22
6.4.4 Procedures 22
6.5 Usual care 27
6.6 Criteria for discontinuing or modifying allocated interventions 27
6.7 Strategies to improve adherence to intervention protocols 28
6.7.1 For health professionals 28
6.7.2 For patients 28
6.8 Relevant permitted / prohibited concomitant care 28
7. Outcomes 29
7.1 Feasibility outcomes (‘stop-go’ or ‘success’ criteria for RCT) 29
7.2 Process data relating to the implementation of the trial 29

For peer review only - http://bmjopen.bmj.com/site/about/guidelines.xhtml

7.3 Process data relating to the implementation of the intervention....30
7.4 Clinical outcomes and covariates....31
7.4.1 Primary clinical outcome....31
7.4.2 Secondary clinical outcomes....32
7.4.3 Adherence to prescribed medication....33
8. Sampling....37
8.1 Quantitative components....37
8.1.1 Sites....37
8.1.2 Sample size....37
8.1.3 Approach, non-participation and recruitment....37
8.2 Qualitative components....38
9. Assignment of interventions....38
9.1 Sequence generation....38
9.2 Allocation concealment....38
9.3 Implementation....38
9.4 Blinding....38
10. Data collection, management and analysis....39
10.1 Quantitative data....39
10.1.1 Data collection methods....39
10.1.2 Data Management....40
10.1.3 Data quality assurance....40
10.2 Qualitative data....41
10.2.1 Audio recordings of consultations....41
10.2.2 Semi-structured interviews: participants....41
10.2.3 Semi-structured interviews: professionals....41
10.2.4 Undertaking the interviews....41
11. Data analysis....42
11.1 Quantitative analysis....42
11.2 Qualitative analysis....43
11.3 Combining data and findings from the different components....44
12. Monitoring....44
12.1 Oversight....44
12.2 Description of any interim analyses and stopping guidelines....45
12.3 Harms (safety assessments)....45
12.3.1 Serious Adverse Events....45
12.3.2 Adverse events we require reporting:....46
12.3.3 Expected SAEs and adverse events....46
12.3.4 Reporting....47
12.4 Auditing....47
12.5 Finance and indemnity....47
13. Ethics and dissemination....48
13.1 Approvals....48
13.2 Protocol amendments....48
13.3 Consent....48
13.4 Confidentiality....49
13.5 Declaration of Interests....49
13.6 Access to data....49
13.7 Ancilliary and post-trial care....49
13.8 Dissemination policy....49
References....51
Appendix 1. W.H.O. Trial Registration Data Set....56

For peer review only - http://bmjopen.bmj.com/site/about/guidelines.xhtml

Figures

Figure 1. Interaction between complex intervention components 14
Figure 2. Interactions between capability, opportunity and motivation 17
Figure 3. Habit formation incorporating COM-B and necessities and concerns......................18
Figure 4. Interplay between COM-B components during habit formation..............................18
Figure 5. Behaviour change intervention flow chart 26
Figure 6. Participant timeline for the external pilot RCT 36
Figure 7. Assumptions of the MRC Guidance on Process Evaluation 43

Tables

Table 1. Learning objectives of the CFHealthHub modules 16
Table 2: Intervention modules 20
Table 3. Individual-level data derived from PWCF and sites..34
Table 4. CFHealthHub data (research arm only) 35

For peer review only - http://bmjopen.bmj.com/site/about/guidelines.xhtml

1. Lay summary

Cystic Fibrosis (CF) is an inherited disease affecting 10000 people in the UK with an average age at death of 28 years in 2012. The lungs of people with CF (PWCF) are prone to infections. Daily physiotherapy and inhaled medications are needed to stay healthy. Around £30 million is spent annually on inhaled therapy but average adherence has been shown to be only 36%. Data suggest that adherence is better in younger children (71% in under 12s, falling to 50% in teenagers) but of the 10000 UK PWCF almost 6000 are now adults. PWCF who collect <50% of their medication cost the healthcare system significantly more than PWCF who collect more than 80% and most of the additional cost results from unscheduled emergency care and hospital admission. This unscheduled emergency care is distressing for PWCF and their families.

We have designed an intervention to help adult PWCF see how much treatment they use. We use dose-counting nebulisers to collect data and send it to a website where it can be displayed. We have worked with PWCF to make the information easy to understand. The website has modules which teach PWCF how to build successful treatment habits. We have developed a toolkit to help PWCF and a health professional (interventionist) work together to form habits of adherence to treatment.

The NHS should not fund this intervention without its effectiveness and value for money being evaluated in a Randomised Controlled Trial (RCT). However, there is currently insufficient information to effectively plan or justify funding a RCT on the scale required. This feasibility study is an essential preliminary to the full scale RCT. The purpose of this feasibility study is to see whether the proposed procedures for the full scale RCT are feasible and acceptable to PWCF. It will also tell us whether the intervention can be delivered by health professionals and is acceptable to PWCF, outside the NHS trust where it was developed.

We will recruit PWCF for four months at two CF units. We hope we will recruit 64 PWCF overall, but will deem the full scale RCT feasible if we recruit 48. A computer will decide whether people who consent to be in the study will receive usual care alone or also receive the intervention. Both groups have a short period of two to four weeks when data is collected through their nebulisers and fed back to the website. It is only after that period that those allocated to the intervention are allowed to use the website and receive enhanced care from the interventionist. After that point, all participants are followed up for 5 (+/-) months. Participants will complete a series of questionnaires at the outset and at 5 (+/-) months.

With appropriate consent, the interventionist or member of the multidisciplinary team (MDT) will audio record consultations between themselves and PWCF who are receiving the intervention or usual care. Qualitative researchers will conduct: 20-24 interviews with PWCF receiving the intervention; 20-24 interviews with PWCF receiving usual care; eight interviews with the four health professionals who are delivering the intervention; and eight semi-structured interviews with members of the wider MDT. These interviews are intended to help the team understand and mitigate potential sources of failure in the intervention and the proposed full-scale trial.

For peer review only - http://bmjopen.bmj.com/site/about/guidelines.xhtml

2. Introduction

2.1 Background

Cystic Fibrosis (CF) is a long term condition (LTC) in which poor adherence to high cost drugs shortens lives and increases NHS costs. CF is a LTC affecting 10,000 people in the UK with PWCF typically dying from lung damage at a median age of 28 years [1]. Randomised controlled trials show that preventative medications reduce exacerbations and/or preserve lung function, [2–8] however adherence is poor. A recent review of objective measures of adherence using medicine possession ratios (MPR: prescriptions collected over prescriptions issued) and instrumented medication monitors showed adherence ranging from 67% for oral antibiotics, 31-53% for inhaled antibiotics, 53-79% for mucolytics agents and 41-72% for hypertonic saline [9]. Accumulating evidence suggests poor adherence is associated with poor outcomes. PWCF collecting four or more courses of alternate month nebulised tobramycin per year were 60% less likely to be admitted to hospital than PWCF collecting one or less [10]. Lower composite MPR predicted exacerbations requiring intravenous antibiotics (IVAB) [9] and over a 12 month period PWCF with an MPR of 80% had significantly lower total healthcare costs than PWCF with an MPR <50% with a cost difference $14,211 per patient and most excess costs related to hospital care [11] . Rescue therapy with IVAB can cause renal failure [12]. The total 2012 UK spend for CF was estimated to be £100 million of which £30 million was spent on inhaled antibiotics and mucolytics [13]. Although patient self-reported adherence to inhaled therapy was 80%, objective measurement showed median adherence was only 36% and the clinicians were unable to predict which PWCF were able to successfully adhere [14] making adherence support difficult. In 2012, the UK CF population received 171,907 days of IVAB with the 93,455 of these that occurred in hospital costing an estimated £27 million [15] . It is recommended that adherence interventions should be targeted where adherence really matters [16] and targeting support towards the high cost inhaled preventative drugs in CF (median adherence 36%) has the potential to impact on the 171,907 days of IVAB a proportion of which will represent rescue therapy necessitated by failed prevention.

2.2 Rationale

The National Institute for Health Research have commissioned a Programme Grant for Applied Research to systematically develop and evaluate an adherence intervention for PWCF. The Programme Grant has three work packages

Work package 1: Build IT infrastructure to capture adherence data from nebulisers. Co-produce a web-portal, 'CFHealthHub', with PWCF and clinicians, in order to display routinely collected adherence data for the use of both groups.

Work package 2: Develop a toolkit based on psychological theory that can support PWCF to adhere to treatment. This will include feedback of measured adherence data and personalised interventions to increase adherence delivered through CFHealthHub. Manualise a Behaviour Change Intervention (BCI) for use by health professionals and PWCF.
All four work packages have received a favourable opinion from an NHS REC:

- Work package 2.1A: A study of the views of people with cystic fibrosis about their condition and treatments (Hampshire A REC: 14/SC/1455; IRAS: 171049);

For peer review only - http://bmjopen.bmj.com/site/about/guidelines.xhtml

- Work package 2.1C: A study to produce videos for the CFHealthHub website (Camden & Kings Cross REC: 15/LO/0944; IRAS: 182367);

- Work package 2.2B: A study to develop a Behaviour Change Intervention (BCI) to help patients with CF manage treatment adherence ((South Yorkshire REC: 15/YH/0332; IRAS: 184477); and,

- Work package 2.2B(1): A study to understand how to use the eTrack and Bi-neb nebuliser to help people with CF to manage their inhalation treatments (West of Scotland REC 5: 15/WS/0089; IRAS: 177900).

Work package 3: Evaluate the toolkit developed in work package 2. The planned definitive evaluation will take place in a large-scale, multi-centre Randomised Controlled Trial (RCT). The definitive evaluation will compare usual care plus staff training in the importance of knowledge, skills and confidence building for adherence versus the same plus the structured behaviour change in intervention (CFHealthHub plus manual).

There is too little information available to effectively plan or justify funding a full scale RCT. We wish to conduct feasibility study comprising of:

- an ‘external pilot RCT’ to establish the feasibility of recruitment to a larger, definitive study; and,

- a ‘process evaluation’ which will help us understand the strengths and weaknesses of both the intervention and research protocols, and ways of addressing any weaknesses.

For peer review only - http://bmjopen.bmj.com/site/about/guidelines.xhtml

3. Aim and objectives

3.1 Aims

The principal aims of this feasibility study are to assess the feasibility and acceptability of:

- a complex intervention, when delivered outside the team which conceived and developed it; and,
- procedures for a full-scale RCT.

3.2 Objectives

1. An external pilot randomised controlled trial to determine feasibility of a randomised controlled trial based on objective stop-go criteria (Section 7.1) related to:

(a) participant recruitment;

(b) participant retention; and,

(c) quality of primary outcome data at 5(+/- 1) month.

2. A process evaluation, relating quantitative and qualitative data on procedures to outcomes, in order to understand and mitigate potential sources of failure in:

(a) the intervention; and,

(b) the full trial.

For peer review only - http://bmjopen.bmj.com/site/about/guidelines.xhtml

4. Design

Mixed-methods study comprising of:

- Quantitative component: parallel group, open labelled, external pilot RCT;

  and,

- Qualitative component: analysis of audio-recorded consultations and interviews.

Quantitative and qualitative data will contribute to the process evaluation.

For peer review only - http://bmjopen.bmj.com/site/about/guidelines.xhtml

5. Participants and study settings

5.1 Settings and locations where the data will be collected

Nebuliser adherence data and information derived from CFHealthHub will be automatically uploaded by participants nebulisers in their own home. Data collection involving patient notes and patient reported outcome measures will take place in two specialist CF units which have not been involved in the development of the intervention. Exacerbation data will be collected by the ACtiF trial interventionist and clinicians at sites from participant notes.

5.2 Eligibility

5.2.1 Inclusion criteria for participants

1. Diagnosed with CF and with data within the CF registry
2. Aged 16 years and above
3. Taking inhaled mucolytics or antibiotics via a chipped nebuliser (e.g. eTrack or Bi-Neb) or able and willing to take via eTrack or Bi-Neb.

5.2.2 Exclusion criteria for participants

1. Post-lung transplant
2. People on the active lung transplant list
3. Patients receiving palliative care, Lacking in capacity to give informed consent
4. Using dry powder devices to take antibiotics or mucolytics

5.2.3 Eligibility criteria for study centres

1. Adult CF Centre;
2. Recognised by commissioners
3. Receiving year-of-care funding

5.2.4 Eligibility criteria for interventionists

1. Health care professional e.g. registered nurse, physiotherapist or other appropriately skilled individual such as a psychology graduate able to work at NHS Agenda for Change Band - 4 or above

For peer review only - http://bmjopen.bmj.com/site/about/guidelines.xhtml

6. Interventions

6.1 Summary

In the external pilot RCT, we will test procedures for a full trial. This involves allocation of PWCF to either a complex intervention or usual care. A 'complex intervention' is defined as one with several interacting components [17]. The complex intervention under evaluation has three broad categories of components (Figure 1):

(a) ***a microchipped device*** (nebuliser) for delivering inhaled medications, which are routinely prescribed for the control of cystic fibrosis (Section 6.2);

(b) ***information technology infrastructure*** to capture and store adherence data from the nebulisers and display it to PWCF and the CF team (Section 6.3); and,

(c) ***the behaviour change intervention***, comprising a software platform ('CFHealthHub' mobile apps and website) offering adherence feedback and tailored modules of content and tools used by the health professional in interactions with PWCF (Section 6.4) and accessed independently by PWCF via CFHealthHub

Services received as usual care described in Section 6.5.

Figure 1. Interaction between complex intervention components

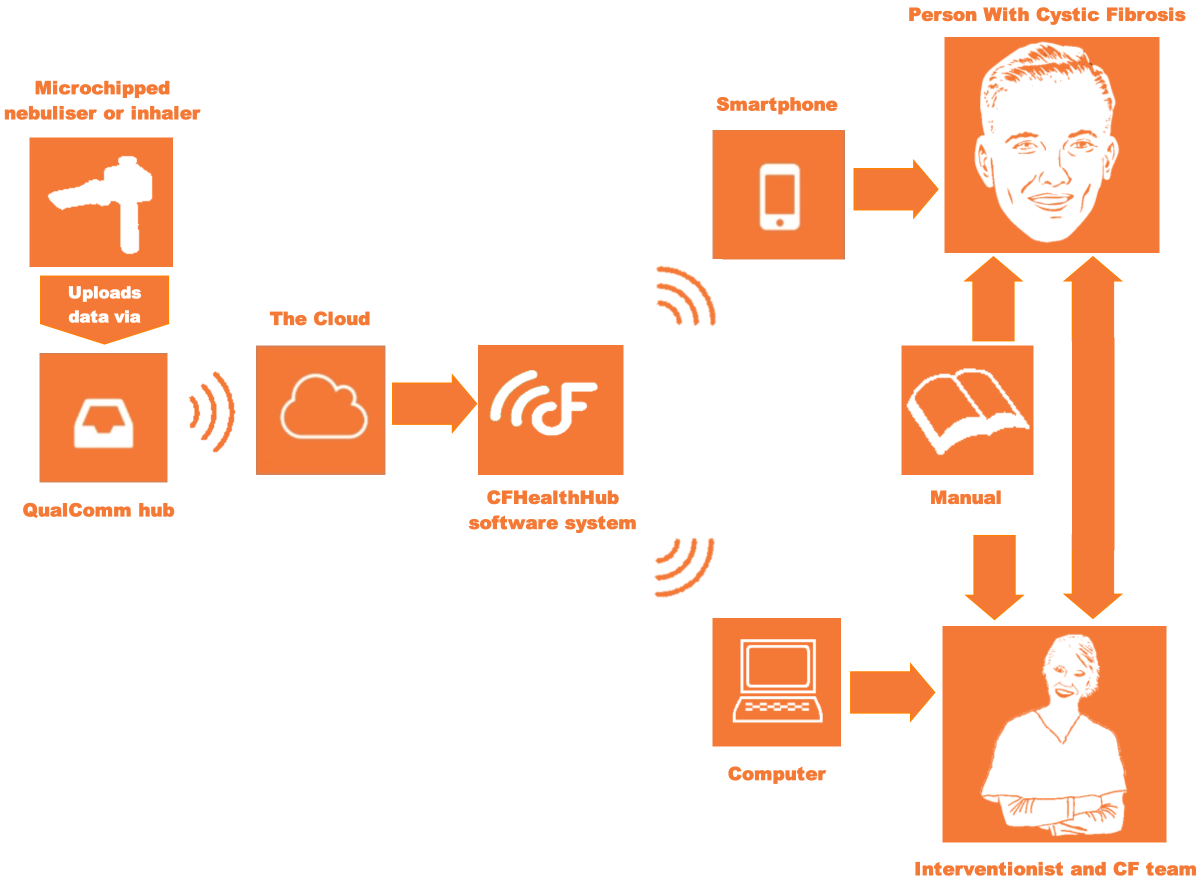

6.2 Microchipped devices

Depending on treatment strategies at different centres the participant may use an eTrack nebuliser system (Section 6.2.1), an Bi-neb AAD System from (Section 6.2.2).

6.2.1 The eTrack nebuliser system (Pari GmbH)

The eTrack controller is a modified version of the eBase controller and can be used to operate both the eFlow rapid nebulizer or Altera nebulizer. Compared to the eBase controller the eTrack is equipped with a Bluetooth chip and has a monitoring function to allow the capture of inhalation adherence data. The eFlow rapid nebuliser with eTrack controller is a CE marked medical device to be used for inhalation therapy. The device allows medications (approved for inhalation) to be transported deep into the lungs.

6.2.2 The Bi-neb AAD System from (Philips Healthcare)

The Bi-neb AAD system is a CE marked medical device which is intended for use to deliver aerosolised liquid medications for participants with cystic fibrosis. The drug delivery device is small and battery powered designed to deliver a precise dose of drug into patient's lungs. The Bi-neb AAD system is designed to deliver liquid medications that are specifically approved for use with the Bi-neb AAD System.

6.3 Information technology infrastructure

The information technology infrastructure for the complex intervention comprises:

i. The Qualcomm hub (Section 6.3.1)
ii. CFHealthHub (Section 6.3.2).
iii. The Bi-Neb data transfer system (6.3.3)

6.3.1 The Qualcomm hub

The Qualcomm hub (Qualcomm; Cambridge, UK) is a wireless device which acquires data from the chipped device and transmits it to a cloud-based data centre. It is a Class I MDD and CE registered in Europe. It is designed, developed and manufactured in accordance with a quality system compliant with ISO13485 standards, meaning it aligns with the quality requirements of international regulatory agencies in the health care industry.

6.3.2 CFHealthHub

CFHealthHub is a web-portal which displays adherence data and provides resources and tools to people with cystic fibrosis and health professionals in order to support improved nebuliser adherence. It is available on-line via computers, tablets or mobile phones.

A qualitative study (WP 2.1A) to identify the barriers and facilitators of nebuliser use in PWCF informed the development of an intervention designed to increase nebuliser adherence. Analysis of the interview data was conducted using the COM-B framework, and these findings were used to inform the development of a complex intervention centred around the feedback of objective adherence data. The intervention was further developed and refined in consultation with PWCF and clinicians. An iterative study in which prototype versions of the intervention were delivered to and reviewed by PWCF was conducted. In that iterative study we interviewed PWCF and interventionists about the usability and tailoring of the

For peer review only - http://bmjopen.bmj.com/site/about/guidelines.xhtml

intervention, and made improvements to the process and materials based on this feedback. The system has been developed to ensure it meets the requirements of the Data Protection Act 1998. It is intended that data on maintenance and relapse will be generated during the full scale trial.

CFHealthHub has a number of modules addressing barriers to adherence based on the COM-B system described in greater detail in Section 6.4.1. The objectives of the modules as mapped to the COM-B are outlined in Table 1 below.

Table 1. Learning objectives of the CFHealthHub modules

COM-B model component	Objectives
Physical capability	- Have the skills to be able to use the nebuliser correctly
Psychological capability	- Understand the importance of nebuliser use in CF treatment<br>- Be able to remember to use nebuliser<br>- Be able to self-monitor nebuliser use<br>- Be aware of a need to improve nebuliser use
Physical opportunity	- Have a realistic medication plan<br>- Have a working/functioning nebuliser<br>- Have a suitable place to use nebuliser<br>- Have the time to use nebuliser
Social opportunity	- Be/feel supported by others to use nebuliser
Reflective motivation	- Perceive benefits of nebuliser use<br>- Perceive few/no concerns about nebuliser use<br>- Understand the health consequences of use/non-use<br>- Feel confident about nebuliser use<br>- Intend to use nebuliser
Automatic motivation	- Have an established routine for nebuliser use<br>- Have a habit to use nebuliser

6.3.3 The Bi-Neb data transfer system

The Bi-Neb Bluetooth data transfer system is intended to automatically extract breathing device use (adherence data) from the device (Bi-Neb) via a Smartphone hub and a secure data server onto CFHealthHub. Providing the Bi-Neb is within the Bluetooth range within the patient's house, the system can retrieve this data once a day.

For peer review only - http://bmjopen.bmj.com/site/about/guidelines.xhtml

6.4 The Behaviour Change Intervention (BCI)

6.4.1 Rationale and theory

The rationale of the BCI is to help CF patients to self-manage their condition and to form habits that will improve adherence to their medication, thereby extending life and improving quality of life. The MRC framework for developing and evaluating complex interventions recommends that intervention development should be informed by a suitable theoretical framework and evidence base [17]. The theoretical model adopted is the COM-B model [18] which describes a 'behaviour system' of the essential and interacting conditions of Capability, Opportunity, and Motivation [18] . The model posits that non-adherence is either non-intentional (a problem of capability or opportunity or intentional (a problem of motivation). The model has been adapted to nebuliser adherence on the basis of evidence about the factors influencing nebuliser adherence in PWCF [19–32], input from expert clinicians currently delivering services to PWCF, as well as from the PPI panel and exploratory research conducted in Sheffield. It is important that interventions are tailored to individual needs and use a multi-modal approach [33]. Each of the conditions of Capability, Opportunity and Motivation has been considered in turn in the development of our intervention. The primary component of the intervention is adherence feedback delivered via the CFHealthHub. Evidence suggests that while personalised feedback can have an effect size of up to 20% in increasing adherence [34, 35], feedback is most effective when combined with additional behaviour change techniques [34].

Figure 2. Interactions between capability, opportunity and motivation

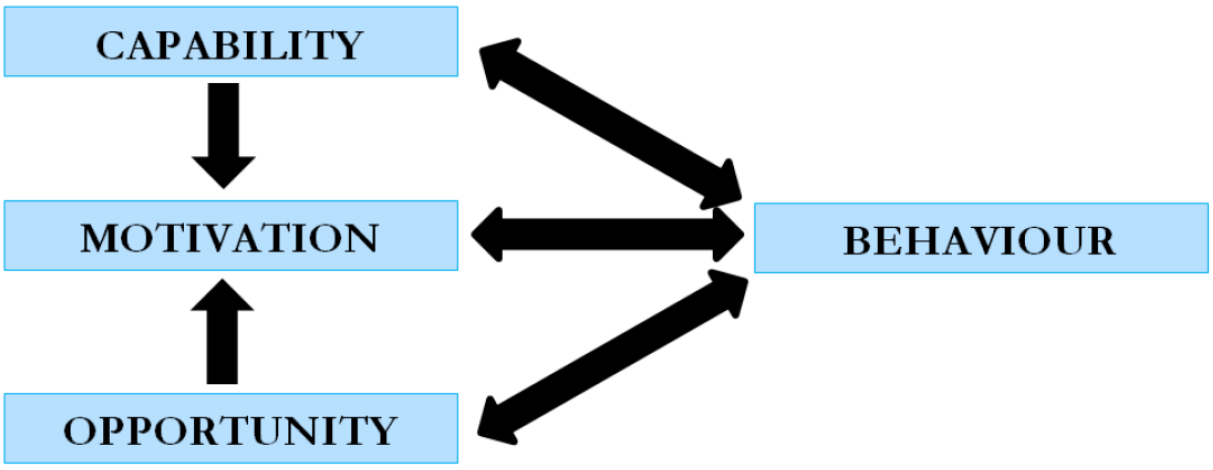

For peer review only - http://bmjopen.bmj.com/site/about/guidelines.xhtml

Figure 3. Habit formation incorporating COM-B and necessities and concerns

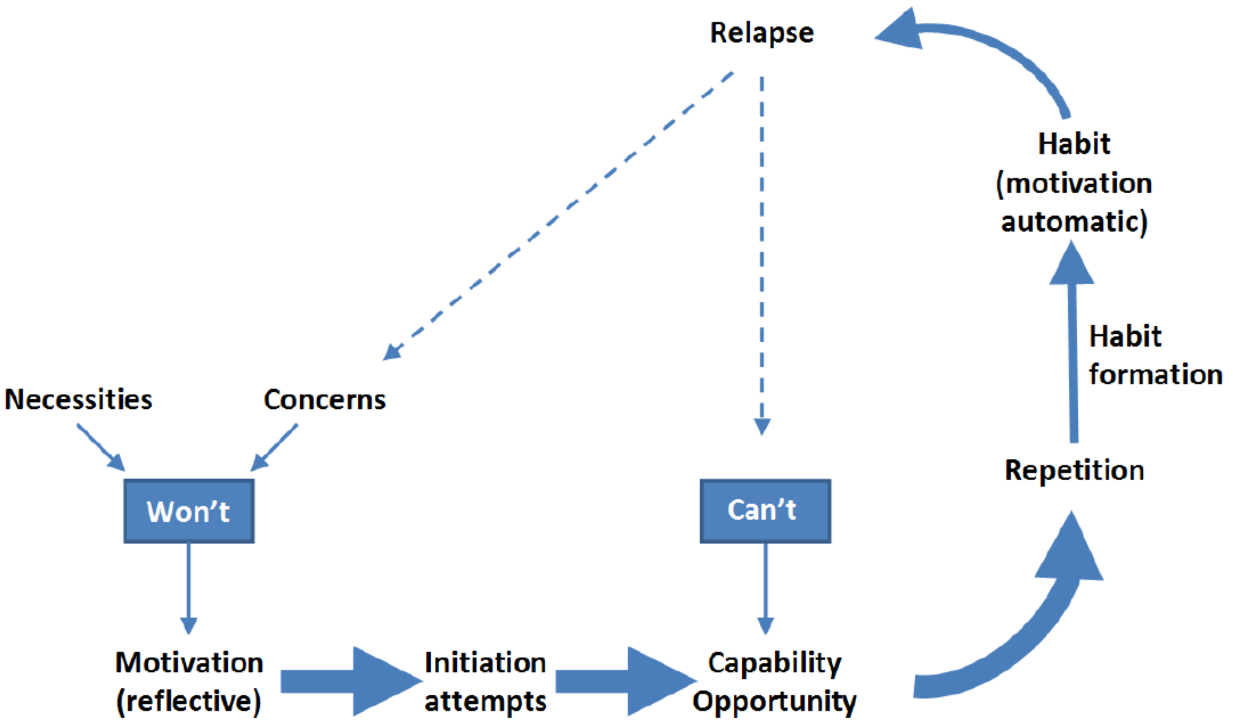

Figure 4. Interplay between COM-B components during habit formation

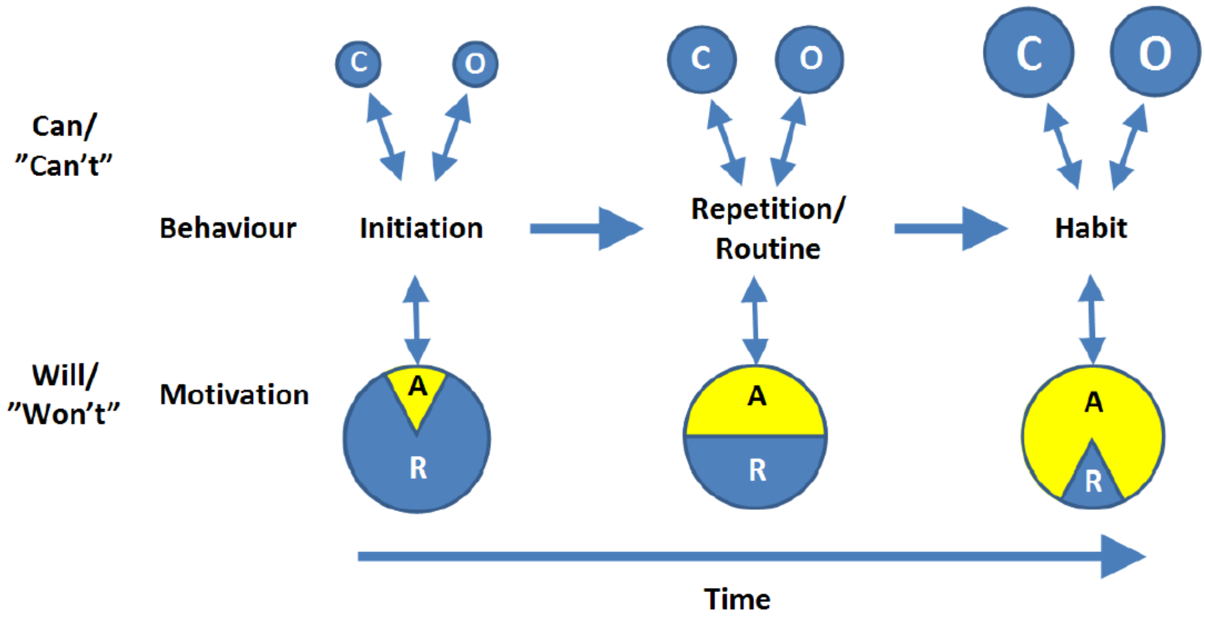

A= Automatic, R= Reflective, C= Capability, O=Opportunity

The identification and choice of appropriate behaviour change techniques has been driven by the Behaviour Change Wheel framework for the development of interventions [Michie, S. F., Atkins, L., & West, R. (2015). The behaviour change wheel: a guide to designing interventions.] which outlines a process of intervention design using the COM-B model "through the systematic evaluation of theory and evidence" (p. 13). In brief, the process involved the following steps:

1. In depth identification and analysis of the factors influencing nebuliser adherence in PWCF through an examination of the existing literature, and a qualitative study in which participants viewed charts of their objective nebuliser adherence data within an interview about factors affecting their motivation, capability and opportunity to adhere to their nebuliser treatment (study 2.1). The Theoretical Domains Framework (TDF; [36]) which analyses Capability, Opportunity and Motivation in greater detail was used as a framework to guide the analysis.
2. Identification and evaluation of potential intervention functions (e.g. education, persuasion, enablement, environmental restructuring, modelling) to address the identified factors influencing nebuliser adherence in consultation with the research team, clinicians and PPI.
3. Development of intervention modules to include specific Behaviour Change Techniques to deliver intervention functions, selection of mode of delivery, and mechanism for tailoring of BCI delivery to meet individual needs with regard to Capability, Opportunity and Motivation. The module contents have been discussed and refined as a result of discussions with clinicians and PPI.
4. Identification of potential mediators of behaviour change, and identification of tools to measure each mediator.

The intervention arrived at through this process is described in Table 2.

For peer review only - http://bmjopen.bmj.com/site/about/guidelines.xhtml

Table 2: Intervention modules

Module	COM-B	Intervention functions	Behaviour Change Techniques	Mode of Delivery
Universal parts of the intervention				
**Self-monitoring**	Psychological capability<br>Reflective Motivation	Education<br>Environmental restructuring<br>Enablement	• Self-monitoring of behaviour<br>• Adding objects to the environment (CFHealthHub)	• Charts of objective adherence data presented within CFHealthHub
**Goal setting & review**	Psychological capability<br>Automatic motivation	Enablement<br>Incentivisation	• Goal setting (behaviour)<br>• Feedback on behaviour<br>• Discrepancy between current behaviour and goal<br>• Review behavioural goals<br>• Graded tasks<br>• Social reward	• Discussion and agreement of goal with interventionist<br>• Review of goal<br>• Feedback on progress (through CFHealthHub and interventionist)<br>• Visual reward if goal met on CFHealthHub
**Treatment plan**	Psychological capability<br>Physical Opportunity Social Opportunity<br>Automatic motivation	Training<br>Environmental restructuring<br>Enablement	• Action planning<br>• Habit formation<br>• Prompts/cues (tailored)	• Action planning tool within CFHealthHub<br>• Option to set reminders
**Confidence building**	Reflective Motivation	Persuasion	• Focus on past success	• Interventionist encouraging focus on periods of higher adherence on charts

Module	COM-B	Intervention functions	Behaviour Change Techniques	Mode of Delivery
Tailored parts of the intervention (based on baseline COM beliefs and barriers questionnaire (COM-BMQ)[1] and consultation with interventionist)				
**My treatment**	Reflective Motivation<br>Psychological capability	Education<br>Persuasion<br>Modelling	• Information about health consequences<br>• Credible source<br>• Salience of consequences<br>• Demonstration of the behaviour<br>• Vicarious consequences<br>• Self-talk	• Q&A linked to information within CFHealthHub (tailored by baseline beliefs and prescription data)<br>• Presentation though text, patient stories, 'talking heads' and animation<br>• Credible sources including clinicians, PWCF and interventionist<br>• Interventionist eliciting self-talk through focus on why motivation is not lower than rating given on pre-screening questionnaire
**Confidence building**	Reflective Motivation	Modelling<br>Persuasion	• Demonstration of behaviour	• 'Talking heads' videos of coping stories within CFHealthHub
**Problem-solving (including skills training)**	Physical capability<br>Psychological capability<br>Physical opportunity<br>Social opportunity	Training<br>Environmental restructuring<br>Enablement	• Instruction on how to perform the behaviour<br>• Demonstration of the behaviour<br>• Behavioural practice/rehearsal<br>• Problem solving<br>• Restructure the physical environment<br>• self-talk<br>• social support (practical)	• Tailored problem solving guided by interventionist<br>• Solution bank within CFHealthHub.<br>• Construction of if-then coping plans<br>• Videos demonstrating correct use of nebulisers within CFHealthHub

1 Incorporating the Beliefs about Medicines Questionnaire (BMQ-specific nebuliser treatment) Horne, 2010

6.4.2 Intervention providers

Interventionists may already be working at, or be new to participating organisations or be the ACtiF interventionist employed to deliver the trial locally at the site. Externally appointed staff will be recruited through a formal job interview. Suitable individuals will include registered nurses or other member of the multidisciplinary team or a ; graduate in a suitable subject such as psychology or, other relevant profession who holds relevant skills / experience. Candidates for the post will ideally have a minimum of two years postgraduate experience which might include delivering a research project to time and target. They will be employed on the Project to work to NHS Agenda for Change Band 4 or above. They must have access to a car for work purposes e.g. participant home visits.

Interventionists will be supported in the delivery of the intervention by members of the Multidisciplinary team (MDT) at the site in which they are based. MDTs will receive training about the approach of the intervention, and the way in which they can support its delivery (see page 28).

Training for interventionists in how to deliver the intervention according to the specifications of the behaviour change manual will be provided by Marlene Hutchings with oversight provided by Madelynne Arden and/or Judy Bradley. A comprehensive training manual and training programme will be developed to facilitate this. A certificate of competence will be provided prior to the interventionist being able to use CFHealthHub with participants.

An additional trained regional interventionist will offer support to trial sites. This on occasion will involve input to patients (face to face or telephone contact), and assisting with problem solving via liaising with the nebuliser company. They will be named on the local site delegation log.

6.4.3 Materials

The BCI contains two broad categories of components:

i. CFHealthHub behaviour change modules including adherence feedback used by PWCF and health professionals

ii. The behaviour change manual and toolkit used by the interventionist in interactions with PWCF in order to understand the specific barriers to adherence for that individual, and to tailor and personalise delivery of the behaviour change modules accordingly.

6.4.4 Procedures

The BCI will be delivered over a 4 to 6 month period through a combination of face-to-face sessions and contact via telephone with an interventionist, and through participant interaction with different modules of content available on CFHealthHub. The interventionist will discuss participant data with members of the MDT to ensure that care is informed by objective adherence data. If any concerns become apparent as the interventionists collect data and work with participants, these concerns will be passed onto the clinical team. The clinical team will

For peer review only - http://bmjopen.bmj.com/site/about/guidelines.xhtml

follow their standard procedures in relation to any concerns raised. The intervention content and delivery flow are outlined in Figure 5 and described below:

6.4.4.1 Consent Visit (all participants)

At the consent visit participants will be given a chipped nebuliser (eTrack) and Qualcom hub or the participants will receive a visit from a clinical trainer who will convert the participant's I-neb to a Bi-neb by adding a Bluetooth chip and providing a Smartphone hub. The clinical trainer may set up the Bi-neb in the patient's home or at hospital either during the main consent visit or at a separate visit after consent has been obtained. Both the eTrack and Bi-neb will connect to CFHealthHub which will enable adherence data be collected. The interventionist will input the participant's prescription details into CFHealthHub. Together these will allow the system to generate adherence charts for that participant. At this visit participants will complete a range of baseline measures (see Table 3) including the COM beliefs and barriers questionnaire (COM-BMQ) which will be entered into CFHealthHub. The responses to this questionnaire will be used to populate the 'My toolkit' section of CFHealthHub with specific tailored elements from the 'My treatment' modules prior to the Initial Intervention Visit. The participant's pseudomonas status will be clarified at baseline and confirmed by the PI with the opportunity to compare the participant's prescription with the pseudomonas status.

6.4.4.2 Initial Intervention Visit (intervention arm only)

Participants will be introduced to CFHealthHub. They will be asked to complete an online consent form on behalf of their NHS trust in which they will specify what additional data they would be willing for CFHealthHub to record and display (e.g. name, and uploaded photographs) and what functional options they would like access to (e.g. push notifications). Permissions may be changed at any time. The participant will have the option to upload their own "patient story" into CFHealthHub after completion of the online consent form.

The interventionist will discuss their motivation to adhere to their nebuliser treatment, will address beliefs associated with poor adherence and will refer back to answers on the COM-BMQ to elicit the participants beliefs associated with adherence. Participants will be shown 'My toolkit' which will have been prepopulated with tailored motivational content (see consent visit).

The interventionist and participant will look at and discuss the adherence charts on CFHealthHub with a focus on period of higher adherence. The interventionist will note any barriers raised by participants during this discussion.

The interventionist will support the participant to identify where and when additional nebuliser treatments could be fitted into their schedule and support them to make an action plan using the online tool available on CFHealthHub. This action plan will be saved to the 'My toolkit' zone. The interventionist will then agree a % adherence goal for the next four to six weeks based on the number of additional treatments that have been planned. This will be recorded on CFHealthHub and will be represented by a target line on the adherence charts.

If motivation is so low that participants are reluctant to set an action plan/goal then the interventionist will spend further time discussing motivation and will skip to confidence building (see below).

The interventionist will encourage participants to focus on likely problems or issues that might disrupt the achievement of the adherence goal and will use the Problem-solving module on CFHealthHub to address each of these anticipated problems. The Problem-solving module includes solutions based on educational content, practical support (e.g. model letters to

For peer review only - http://bmjopen.bmj.com/site/about/guidelines.xhtml

employers) and interactive tools. Relevant solutions will be saved to the 'My Toolkit' zone of CFHealthHub.

The interventionist will discuss the participant's confidence to meet their goal and will identify 2-3 'talking heads' videos showing other people with CF addressing and overcoming similar barriers to nebuliser adherence.

The visit will conclude with a review of the goal and the tailored and personalised contents saved to the 'My toolkit' zone of CFHealthHub. The interventionist will encourage a learning mindset, emphasising that even if adherence doesn't increase starting to think about adherence will produce learning that will make subsequent attempts to change easier.

6.4.4.3 Participant Independent access to CFHealthHub (intervention arm)

Participants will have independent access to CFHealthHub at all times following the Baseline visit. They can, at any time, access their adherence charts, 'My toolkit' contents, and can browse the other areas of content as they wish. Frequency of access to each area of CFHealthHub will be monitored and recorded.

Adherence charts will provide colour -coded feedback about participant achievement towards their adherence goal so that they are provided with immediate, easy to recognise information about their achievements. Subject to consent, participants will be sent encouraging messages via push notifications, or alternatively when they access CFHealthHub, to match the progress made e.g. congratulations on achieving their goal, congratulations on having made progress towards their goal, encouragement to remember their action plan.

6.4.4.4 Review visit (Visit 3 - intervention arm)

At the review visit, the interventionist and participant will look at and discuss the adherence charts on CFHealthHub and goal achievement with a focus on progress made and periods of higher adherence.

If the adherence goal was met then the participant will be encouraged to set a new higher adherence goal or to a goal to maintain their current level of adherence which will be recorded on CFHealthHub. Following this the participant and interventionist will review the contents of 'My toolkit' and revise action plans, problems/solutions as required. If issues of motivation are still a concern the interventionist may recommend additional/alternate elements of content from 'My treatment' or 'Talking heads' to go into 'My toolkit'.

If the adherence goal was not met then the interventionist and participants will discuss the barriers to goal achievement (motivation, capability, opportunity). The interventionist will address beliefs associated with poor adherence and will add/revise the elements of content from 'My treatment' or 'Confidence building' to go into 'My toolkit'.

If no goal was previously set then the interventionist will review motivation and confidence and then will consider if the participant is ready to action plan and set a goal. If not they will spend more time reviewing motivation and confidence.

The participant will be encouraged to set a realistic % adherence goal for the next four to six weeks and this will be recorded on CFHealthHub. The interventionist will support the participant to revise their action plan as needed and save this to the 'My toolkit' zone. Based on the earlier discussion about the barriers that prevented goal achievement the Problem-solving module on CFHealthHub will be used to address each of the problems encountered, and any that are anticipated. Relevant solutions will be saved to the 'My Toolkit' zone of CFHealthHub.

The visit will conclude with a review of the goal and the tailored and personalised contents saved to the 'My toolkit' zone of CFHealthHub. The interventionist will re-emphasise a

For peer review only - http://bmjopen.bmj.com/site/about/guidelines.xhtml

learning mindset, emphasising that the participant cannot fail, but can learn from the process so that they can work together on the adherence challenge.

Participating centres will provide participants with contact details, typically telephone numbers, but other methods may be volunteered by centres. Contact details will be provided so that participants can contact the centre if they have queries or problems regarding CFHealthHub between visits. The interventionist will be able to feedback any information from the intervention delivery **after** the baseline intervention visit to members of the wider CF team. This may include adherence data from sessions with the participant's clinician and MDT particularly if the participant raises any concerns or issues e.g. side effects of a drug to allow their usual clinician to discuss this with them at their next clinic visit.

6.4.4.5 Subsequent Review (intervention arm)

Following these two sessions the amount of interaction which each PWCF has with the interventionist will be tailored to their needs and requirements although it is anticipated that these will normally marry with routine clinic visits: They may have additional face-to-face sessions or contact via telephone or e-mail. No more than one monthly face-to-face session will be conducted because of the research protocol; if the participant requests additional support, the centre may accommodate this at their discretion. Review meetings will take 30 minutes and be conducted over the 5month (+/- 1 month) of the follow-up period. The structure of review sessions will follow the same pattern as for 6.4.4.4.

6.4.4.6 Final research visit (5 months +/- 1 month from consent)

All participants will complete a final research visit 4-6 months from the date of consent. At this visit the interventionist will collect the primary and secondary outcome data (see table 3) including demography data, health care resource use and the participant completed questionnaires. At this final research visit the interventionist will re-check that all adherence data has been transferred to CFHealthHub. The eTrack can store approximately 6 months of treatment data, ensuring all the data is transferred at this visit should help to prevent missing data.

Following the final 4-6 month post-consent research visit, we will continue to collect: adherence data from CFHealthHub; exacerbations; FEV1 and ask participants the subjective adherence question until, 30th April 2017. At this point the study closes and the involvement of all participants ceases. After the trial ends (30/4/17), the aspiration is to allow participants in the control to have access to the intervention for which negotiations are ongoing. Currently funding is in place for the trial interventionists at study sites to deliver the intervention only over a 12-month period i.e. up to 30/4/17. It is anticipated that CFHealthHub used outside the trial would be delivered within the existing resources of the MDT so using CFHealthHub outside the trial should not need the trust to employ any additional staff members. As this is a pilot feasibility study where we are testing the intervention in participants, there is an expectation that further iteration of CFHealthHub may occur.

For peer review only - http://bmjopen.bmj.com/site/about/guidelines.xhtml

Figure 5. Behaviour change intervention flow chart

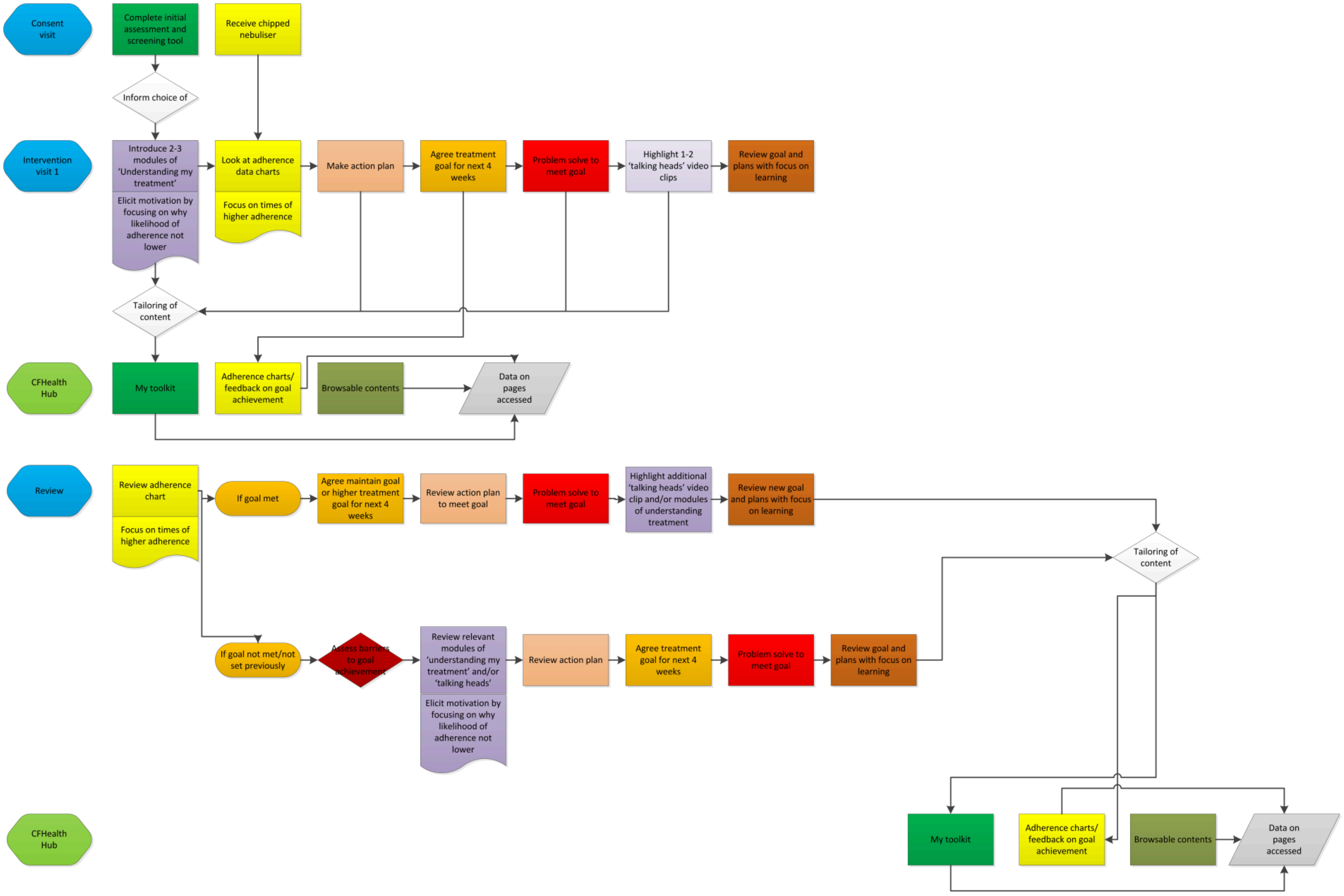

For peer review only - http://bmjopen.bmj.com/site/about/guidelines.xhtml

6.5 Usual care

Patients in both arms will receive usual care. Usual care is heterogeneous within and between centres, based on the needs of patients and the skills and interests of CF Unit staff. To better understand the configuration of usual care at participating centres a survey tool will be administered by the CTRU to the lead clinician at the centre. This will identify the spectrum of clinical and behaviour change interventions that are in use in the management and self-management of CF.

A minor component of the intervention is to train all members of the MDT in awareness of patient activation so that they are open to addressing issues raised for PWCF in the intervention arm. In addition, a staff member in the MDT will help to deliver the intervention. There is the possibility that the awareness of patient activation will have some effect on PWCF in both the intervention and control arms, and of leakage of the learning from the behaviour change component of the intervention to controls. We will investigate this possibility during the process evaluation.

Members of the MDT at each centre will receive one half-day, on-site, face-to-face training about the importance of objective nebuliser adherence data in the management of CF, and awareness of the importance of building patients' knowledge, skills and confidence to enable them to self-manage their treatment. This will include training in the interpretation of graphs and charts of objective adherence data produced by CFHealthHub, and the rationale for reducing target adherence in poor adherers in order to increase confidence. This will be delivered by designated members of the ACtiF research team.

Participants in the control arm will use a microchipped nebulizer but will not be able to access adherence data or other content and tools through CFHealthHub, neither will they receive the structured CFHealthHub intervention as described in the intervention manual. Control arm participants using Bi-neb nebulizers might have access to their data as part of routine care but this will not be in the user friendly format provided by the intervention.

One function of the qualitative research interviews with staff and control participants (see Section 8 below) is to understand the extent to which the patient activation awareness training has affected staff behaviour and whether control arm participants have received some aspects of the behaviour change intervention.

6.6 Criteria for discontinuing or modifying allocated interventions

There are no criteria for discontinuing treatment. Participants will be made aware that their participation is voluntary and they may discontinue study interventions, should they wish, at any time.

If a participant wishes to withdraw from treatment they will be able to speak to a member of the site study team i.e. ACtiF interventionist. This will be documented on a participant withdrawal form, within the Case Report Form. Any data already collected during the course of the trial up to the point of withdrawal will be used in the final analysis. We will ask the participants for their permission to continue to collect the primary outcome data i.e. CF exacerbations. The participant or clinician can make the decision to discontinue the allocated study intervention for any reason.

Participants will have the following options if they wish to withdraw:

1. Withdraw from the intervention i.e. intervention delivery visits only but will remain in the study. Patients can continue to use CFHealthHub. All study data would continue to be collected at subsequent follow up time points as per protocol.
2. Withdrawal from the study. Unless the patient objects, any data collected up to this point would be retained and used in the study analysis. The local interventionist would ask the participant if they agree to the collection of primary outcome data as defined in the protocol and or adherence data If

they agree to collection of adherence data, CTRU and or interventionist will continue to follow up participants for adherence data.

3. Withdrawal from the trial entirely. Unless the patient objects, any data collected up to this point would be retained and used in the study analysis. If the patient does not wish to be contacted with regard to primary outcome data or adherence data, no further contact with regard to the study will be made. If the participant does specifically request for all their data to be removed information regarding the participant will be retained at site, as part of the patient notes, along with their withdrawal form and request to delete the data.

A participant would be classed as complete if they have continued in the study until the last protocol defined visit, however there may be missing visits and / or data.

Loss to Follow-Up

A participant would be classed as lost to follow up if the participant has 1) not completed the study or 2) been withdrawn despite attempts for further contact, as per protocol, having been made. Unless the participant withdraws from the study entirely we will continue to collect the primary outcome data when possible (i.e. from medical notes).

This withdrawal section has been developed in accordance with the CTRU Participant Discontinuation and Withdrawal of Consent Standard Operating Procedure (SSU003).

6.7 Strategies to improve adherence to intervention protocols

6.7.1 For health professionals

The intervention protocols will be described in detail in an intervention manual. Interventionists will be trained to deliver the intervention according to the manual protocols. Interventionist training (as a form of behaviour change) will focus on Capability, Opportunity and Motivation. It will utilise evidence about the importance and likely effectiveness of the intervention and will challenge common misconceptions about adherence. Skills training and an introduction to the tools available on CFHealthHub will increase staff capability, and we will work with clinics and clinicians to ensure that the practical requirements for intervention delivery are in place: space, time etc (opportunity).

CFHealthHub will record interventionist access to the site. It will also automatize some of the tailoring of the intervention according to the COM-BMQ which will be completed online. The contents of 'My Toolkit' will be recorded for each participant so that we will have records of what content they have been recommended. Interventionists will also be required to complete session records each time that they deliver the intervention to record the decisions made and the reasons for these,

6.7.2 For patients

Where participants provide consent we will send optional push notifications to encourage engagement with CFHealthHub. For example, we will send congratulatory messages when adherence improves, encouraging messages to remind participants to engage with the content. Face-to-face visits will, where possible be arranged to coincide with clinic visits as per usual care, therefore minimising the additional burden on participants.

6.8 Relevant permitted / prohibited concomitant care

No concomitant care will be denied based on the research protocol.

7. Outcomes

7.1 Feasibility outcomes ('stop-go' or 'success' criteria for RCT)

In line with proposed CONSORT extension for pilot studies [37], in this section, we state the criteria for success of the external pilot trial. The criteria are based on the primary feasibility objectives, which provide the basis for interpreting the results of the external pilot and for determining the feasibility of proceeding to the full-scale study scheduled for months 31 to 60 of the project. Depending on the funder's perspectives, the outcome of the external pilot might be:

(i) "Stop - main study not feasible";
(ii) "Continue, but modify protocol - feasible with modifications";
(iii) "Continue without modifications, but monitor closely - feasible with close monitoring"; or,
(iv) "Continue without modifications - feasible as is."[37]

We anticipate that modifications to the research protocol will be necessary as the feasibility study progresses. Some of the qualitative research will be undertaken early in the pilot trial and lessons learned about the trial procedures will be identified and acted on during the pilot trial. There are three objective stop-go criteria:

1. Feasibility of recruitment to RCT
Defined as recruitment of no fewer than 48 participants randomised at two centres over four months, 75% of the rate required in the main trial;

2. Feasibility of retaining participants in the RCT
Defined as attrition from the research protocol of no more than 15% of randomised participants at 5 (+/-1) months.

If these are met the full trial will go ahead. If these are not met overall, but are met in the last half of the pilot trial after trial procedures have been improved based on lessons learned from the early stage of the pilot trial, then the full trial will go ahead.

7.2 Process data relating to the implementation of the trial

1. Number and characteristics of eligible patients approached for the study
Collected by centres in screening logs and transferred to Prospect database

2. Reasons for refused consent
Collected by centres in screening logs and transferred to Prospect database.

3. Reach
How many participants are consented into the study, sub-grouped by socio-economic status (from CF Registry), as a proportion of:

- Those approached, expressed quantitatively, based on 'pre-screening' logs completed by ACtiF interventionist;

- Those known to be eligible, expressed quantitatively based on CF Registry.

4. Participant attrition rate
Collected by centres in screening logs and transferred to Prospect database.

For peer review only - http://bmjopen.bmj.com/site/about/guidelines.xhtml

5. Reasons for attrition
Collected by centres in screening logs and transferred to Prospect database.

6. Maintenance:
The processes by which participants are kept involved in the collection of key secondary outcome data research data:
- The extent to which adherence data is successfully uploaded from the chipped nebulisers , described quantitatively using CFHealthHub (Intervention arm only).

7. Number of missing values/incomplete cases
Assessed by data management team, based on data in Prospect database.

8. Participant,/interventionist and members of MDT views on research protocols
Assessed through qualitative interviews and to include:
- Barriers to recruitment, problems encountered in reaching participants [38];
- Perceived problems with trial procedures such as recruitment, informed consent etc.
- Acceptability
- Perceived utility and burden of outcome assessments.

9. A survey on the content of usual care at participating centres
A CTRU staff member will complete this survey with the principal investigator, a senior medic or delegate working at the participating centre.

7.3 Process data relating to the implementation of the intervention

1. Context
Definitions of 'context' tend to cluster around setting, roles, interactions and relationships [39]. It is important that context is understood as diachronic and emergent rather than synchronic and static [40, 41]. Frameworks for process evaluation have defined 'context' as:

- "aspects of the larger social, political, and economic environment that may influence intervention implementation" [42];

- "factors external to the intervention which may influence its implementation, or whether its mechanisms of impact act as intended" [43].

The context, and its interaction with implementation, mechanisms of impact, outcomes, the description of the intervention and its causal assumptions [43] will be described using qualitative data from research interviews, field notes, study management logs, minutes and e-mails. The focus will be how the context of individual CF Units affects implementation of the intervention and its potential outcomes.

2. Implementation
Definitions of 'implementation' tend to cluster around the processes or stages of adoption, the methods, means or social organisation of bringing innovative practices into use [39]. One way of describing the process of getting research into practice is to use a process model [44]. To structure our narrative of how the complex intervention was implemented we will use a process model called the Quality of Implementation Framework [45].

3. Recruitment:
Based on e-mails and minutes we will describe in narrative terms, the procedures used to approach and attract to the project NHS Trusts and interventionists [42].

For peer review only - http://bmjopen.bmj.com/site/about/guidelines.xhtml

4. Training:
The comprehensiveness of the training component of the intervention for the health professionals delivering the intervention will be assessed by a combination of audio recordings of consultations and by interview.

5. Fidelity
"The extent to which the intervention was delivered as planned. It represents the quality and integrity of the intervention as conceived by the developers. Fidelity is a function of the intervention providers."[42]

- Interaction with participant along lines recommended by manual, determined by audio recordings of consultations between the interventionist and PwCF in the intervention arm.

- Recommendation of appropriate CFHealthHub tasks by interventionist, determined by audio recordings and by data from CFHealthHub;

The fidelity assessment will be developed and based on a tool used by Borelli et al [46].

6. Use [38] ***/ dose received*** [42] ***of intervention***
Use of CFHealthHub by participant, as proposed by interventionist, determined by data capture by CFHealthHub, including the online activities started and completed, minutes spent on recommended pages and which parts the participant has picked out and put in a "my favourites" page. The number of times, frequency over time and duration with which users log on to CFHealthHub, as well as the activities they perform while logged in, described quantitatively using data from CFHealthHub.
A record of the discussion between the interventionist and the MDT will be kept. This will include who was there, brief notes of what was discussed and any agreement of treatment goals made.

7. Acceptability
The acceptability of the intervention to hospital staff and PWCF assessed through semi-structured interviews.

8. Perceived benefits and harms
Assessed through semi-structured interviews with health professionals and PWCF.

9. Leakage of intervention to controls
Assessed through audio recordings of consultations between the MDT, interventionist, and PwCF in the control arm, and semi-structured interviews with PwCF in the control arm.

7.4 Clinical outcomes and covariates

The time schedule of enrolment, interventions (including any run-ins and washouts), assessments, and visits for participants can be found in Table 3 and Table 4 below.

7.4.1 Primary clinical outcome

The primary clinical outcome is the number of pulmonary exacerbations in 5 (+/-1) month post-baseline follow-up period, defined according to the Fuchs criteria [47]. An exacerbation of respiratory symptoms will be said to have occurred when a patient was treated with parenteral antibiotics for **any one of the following 12 signs or symptoms** [48]**:**

1. change in sputum;
2. new or increased hemoptysis;
3. increased cough;
4. increased dyspnea;
5. malaise, fatigue, or lethargy;

6. temperature above 38 °C;
7. anorexia or weight loss;
8. sinus pain or tenderness;
9. change in sinus discharge.
10. change in physical examination of the chest, derived from notes by site staff.
11. decrease in pulmonary function by 10 percent or more from a previously recorded value, derived from notes by site staff; or,
12. radiographic changes indicative of pulmonary infection, derived from notes by site staff.

The trial interventionist or prescribing clinician/nurse will collect data on the "exacerbations" form at the point of a participant starting a course of IV antibiotics.

7.4.2 Secondary clinical outcomes

1. **Body Mass Index (BMI).**

2. **Forced expiratory volume in 1 second (FEV_1):** standardised spirometry as a measure of condition severity [49].

3. **EuroQol EQ-5D-5L**: generic health status measure for health economic analysis [50].

4. **The Patient Activation Measure (PAM-13) (Health Style Assessment)**: assessment of patient knowledge, skill, and confidence for self-management [51]. *PAM-13 was labelled as "Health Style Assessment" following a request from the licence owners to ensure the purpose of the questionnaire is clear for participants.

5. **Assessment of routine :** measure of life chaos [52].

6. **Self-Report Behavioural Automaticity Index (SRBAI):** automaticity-specific subscale of the Self Report Habit index to capture habit-based behaviour patterns [53].

7. **Cystic Fibrosis Questionnaire-Revised (CFQ-R):** disease specific health-related quality of life instrument [54].

8. **The Patient Health Questionnaire depression scale (PHQ-8):** severity measure for depressive disorders [55].
9. **MAD (Medication Adherence Data-3 items) :** medication adherence measure

10. **The General Anxiety Disorder 7-item anxiety scale (GAD-7):** severity measure for anxiety [56].

11. **The Capability Opportunity Motivation Behaviour Beliefs Questionnaire (COM-BMQ):** This questionnaire incorporates:

    a. **The Beliefs about Medicines Questionnaire - specific (Nebuliser adherence) (BMQ 21-item):** a validated self-report tool[57], customised by the author to identify perceived necessities and concerns for nebuliser treatment.
    b. The following project-specific items: one additional belief item, one intention item, one confidence item, and a list of barriers. These will serve as a tailoring tool for the intervention and also as a secondary outcome measure.

12. **Subjective adherence single question:** self-report estimate of adherence as a percentage. Self-reported problems: identification of capability and opportunity barriers to nebuliser adherence

For peer review only - http://bmjopen.bmj.com/site/about/guidelines.xhtml

13. **Concomitant medications:** bespoke instrument, designed for this research project.

14. **Resource use form:** interventionist collects data from a combination of hospital notes and the NHS patient electronic system to determine 1) inpatient IV days 2) Routine clinic visits 3) Unscheduled outpatient contacts 3) unscheduled inpatient stays.

15. **Exploratory analysis of habit formation**: analyses with the objective nebuliser data will be performed to explore the process of habit formation with the delivery of the adherence intervention

16. **Prescription**: a monthly prescription check to both check for data transfer to CFHealthHub and review for an indication that the prescription has changed or indication of microorganism e.g. pseudomonas (please see table 2 and 3 and refer to section 10.1.1).

17. **Adherence to prescribed medication (see 7.4.3)**

18. **Any treatment with IV antibiotics**

7.4.3 Adherence to prescribed medication

Adherence to prescribed medication will be defined in several ways including:

1. Unadjusted adherence
2. Simple normative adherence (without numerator adjustment)
3. Sophisticated normative adherence (without numerator adjustment)
4. Simple normative adherence (with numerator adjustment)
5. Sophisticated normative adherence (with numerator adjustment)

Further detail about the outcomes will be reported in the trial statistical analysis plan.

For peer review only - http://bmjopen.bmj.com/site/about/guidelines.xhtml

Table 3. Individual-level data derived from PWCF and sites

	Where?	Completed by?	Consent visit	Baseline (intervention) visit	At clinic visits	Exacerbations episode	5 months (+/- 1 month) from consent visit	Up to 30th April 2017
**Enrolment**								
Pre-screening form (before 1st visit)	Prospct	Site	-	-	-	-	-	-
Confirmation of eligibility form	Prospct	Site	●	-	-	-	-	-
Informed consent	Prospct	Site	●	-	-	-	-	-
Intravenous days in last registry year	Prospct	Site	●	-	-	-	-	-
Pseudomonas status +	Prospct	Site	●	-	-	-	-	-
**Primary outcome**								
Exacerbations form including:<br>Parenteral antibiotics<br>Change in sputum*<br>New or increased hemoptysis*<br>Increased cough*<br>Increased dyspnea*<br>Malaise, fatigue, or lethargy*<br>Temperature above 38 °C*<br>Anorexia or weight loss*<br>Sinus pain or tenderness*<br>Change: sinus discharge*<br>Change: phys. exam. chest*<br>Decrease: pulmonary function *<br>Indicative radiographic changes*	Prospct	Site	●	-	-	●	●	●
**Secondary outcomes**								
BMI (height and weight)	Prospct	Site	●	-	-	-	●	-
FEV_1	Prospct	Site	●	-	●	-	●	●
EQ-5D-5L**	Prospct	PWCF	●	-	-	●	●	-
PAM-13(Health Style Assessment)	Prospct	PWCF	●	-	-	-	●	-
Assessment of Routine	Prospct	PWCF	●	-	-	-	●	-
SRBAI	Prospct	PWCF	●	-	-	-	●	-
CFQ-R	Prospct	PWCF	●	-	-	-	●	-
PHQ-8	Prospct	PWCF	●	-	-	-	●	-
GAD-7	Prospct	PWCF	●	-	-	-	●	-
MAD-3 (Medication Adherence Data-3 items)	Prospct	PWCF	●	-	-	-	●	-
COM-BMQ	Prospct	PWCF	●	-	-	-	●	-
Objective adherence	CFHH	CFHH	●	-	●	-	●	-
Subjective adherence single question	Prospct	PWCF	●	-	●	-	●	●
Concomitant medications	Prospct	Site	●	-	-	-	●	-
Other SAEs	Prospct	Site	-	-	●	-	●	-
Resource use	Prospct	Site	-	-	-	-	●	-

+ Pseudomonas (or other microorganism) status will be checked together with the monthly prescription
* Only required where PWCF indicates they have received parenteral antibiotics

For peer review only - http://bmjopen.bmj.com/site/about/guidelines.xhtml

** EQ5D-5L collected at the start and end of every exacerbation episode

Table 4. CFHealthHub data (research arm only)

	Completed by?	Baseline (intervention) visit	At intervention visit s with interventionist	Between sessions	At clinic visits	5 months (+/- 1 month) from consent visit	Up till 30th April 2017
**Clinician metrics**							
Adherence data*	PWCF	●	●	●	●	●	●
Recommendation of modules by interventionist	Interventionist	●	●	-	●	-	x
Feed back to participant their adherence data screens (data click)	Interventionist	●	●	-	●	-	x
Check prescription with participant	Interventionist	●	●	-	●	-	x
Order of clicks	CFHH	●	●	-	●	-	x
Interventionist responds to patient changing prescription	Interventionist	-	●	●	●	●	x
Monthly check on prescription +	Interventionist / CTRU	●	●	●	●	●	x
Time in and out preparation	Interventionist /CFHH	●	●	-	-	●	x
Time in and out with patient	Interventionist /CFHH	●	●	-	-	●	x
Time in and out review	Interventionist /CFHH	●	●	-	-	●	x
**Patient metrics**							
Adherence (number of nebulized doses taken per day.) [1]	PWCF	●	●	●	●	●	x
Duration of inhalation	Nebuliser	●	●	-	-	-	x
Accessing CFHealthHub – look at adherence data	PWCF	●	●	-	-	-	x
Accessing CFHealthHub – look at 'My Toolkit'	PWCF	●	●	-	-	-	x
Accessing CFHealthHub problem solving / education / talking heads pages outside of 'My Toolkit'	PWCF	●	●	-	-	-	x
Accessing CF HealthHub – first to last click in a session	PWCF	●	●	-	-	-	x

*Adherence data collected for both research and control arms

+ Monthly prescription checked by CTRU centrally to alert local interventionists to any potential changes in control arm and potentially also intervention arm

X data continued to be collected in CFHealthHub and interventionist responds for those participants who have "opted in" to receive intervention till 30/4/17

[1]To be broken down in statistical analysis plan.

Figure 6. Participant timeline for the external pilot RCT

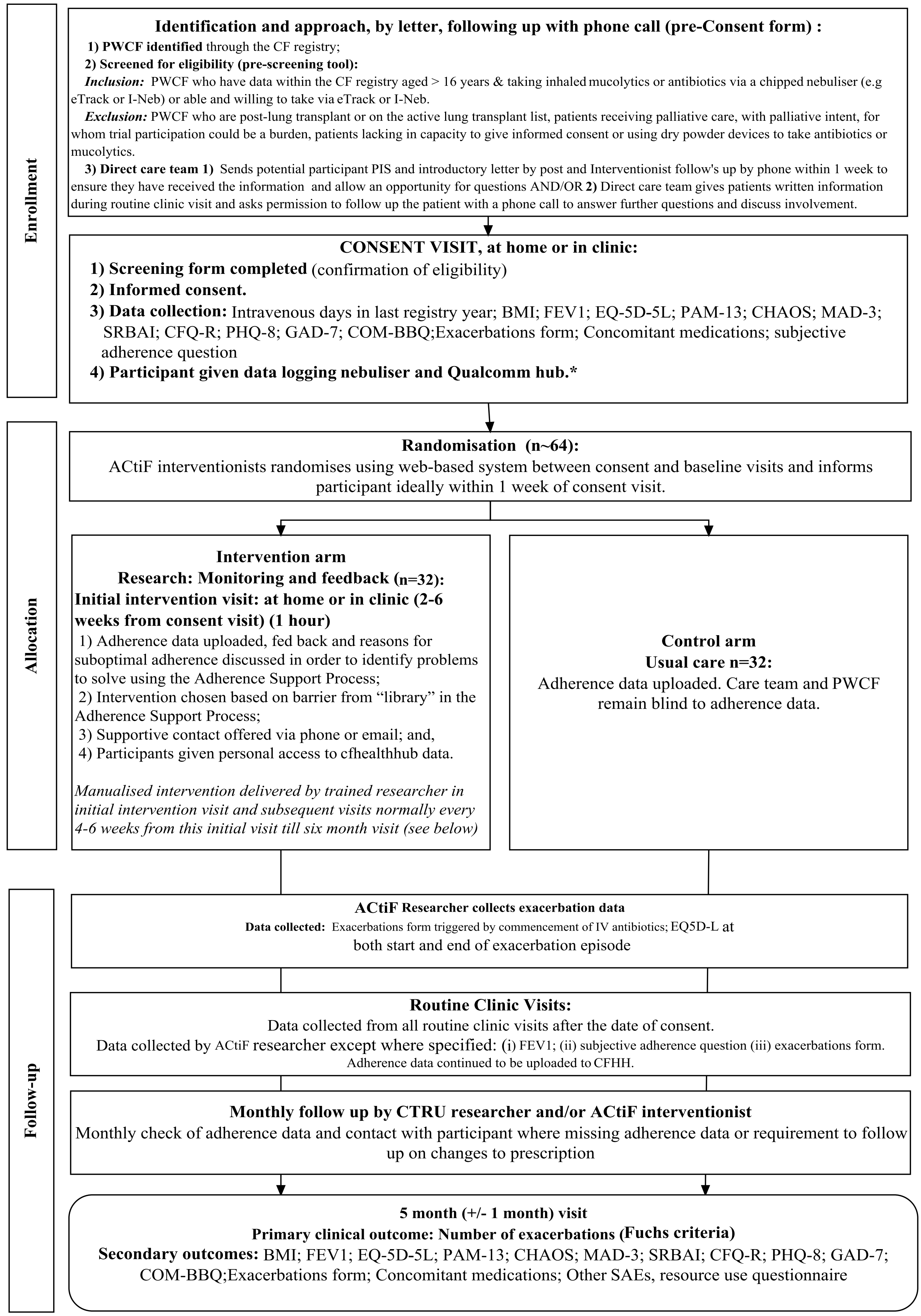

*When I-nebs are converted to Bi-nebs a representative from the company (Philips) will do this between the consent visit and first intervention visit.

For peer review only - http://bmjopen.bmj.com/site/about/guidelines.xhtml

The study recognises that flexibility in accommodating participant schedules may cause time windows to change but this will allow us to adapt the intervention for the main RCT.

8. Sampling

8.1 Quantitative components

8.1.1 Sites

Two large specialist CF centres have been screened for their ability to recruit participants based on the number of participants they have on their CF registry and their motivation to participate in the pilot trial.

8.1.2 Sample size

The sample size for a feasibility study should be adequate to estimate the uncertain critical parameters (standard deviations for continuous outcomes; consent rates, event rates, attrition rates for binary outcomes) needed to inform the design of the full RCT with sufficient precision [37, 58–60]. For the main RCT, the target sample size is 688 participants (344 per arm). We are proposing that 15 CF units recruit on average 46 patients in six months, a recruitment rate of approximately eight patients per centre per month.

To assess whether this recruitment rate is feasible the external pilot RCT will open in two CF units for 12 months, with four months recruitment, one months 'run-in' period (the period between the consent and baseline visit), and 5 (+/-1) months follow up. To match the proposed recruitment rate of the main RCT, the target sample over the four months for which the pilot RCT is open, will be 32 per centre (64 in total from the two pilot centres). We propose to recruit to time, that is for a fixed period of four months rather than to a fixed sample size. We would want to see a minimum of 75% of the recruitment target to be confident of the trial viability i.e. at least 48 patients in total consented and randomized in four months' of recruitment from two centres.

8.1.3 Approach, non-participation and recruitment

Approach: Health professionals involved in approaching and screening PWCF and collecting data will be trained in the study protocol and procedures. Additionally those taking consent will have up-to-date training in Good Clinical Practice (GCP). All study personnel will be named on the study delegation log. Health professionals working with the CF team will identify a sample of PWCF registered at the centre via the CF registry database locally. All inclusion and exclusion criteria will be assessable via patient records and they will exclude any patients who do not fit the eligibility criteria.

A member of the participant's direct clinical team will send the potential participant a PIS and introductory letter by post or give the written information during a routine clinic visit. A sticker with a website address and Quick Response code will be placed in the envelope both of which will link to a video of the researcher explaining the study. If information is provided in a routine clinic visit, the clinical care team will seek permission for the ACtiF Interventionist to follow up with a phone call in order to answer any further questions and discuss involvement. Written informed consent may be conducted at this visit where the participant is happy to take part as this is a low risk trial.

Telephone call: Up to a week after posting out the information, the ACtiF Interventionist will telephone the PWCF to discuss the study over the phone and answer any questions. If the potential participant is happy to take part, the ACtiF Interventionist will arrange an appointment to gather written informed consent.

Non-participation: Spontaneously offered reasons for non-participation in the trial will be recorded.

For peer review only - http://bmjopen.bmj.com/site/about/guidelines.xhtml

8.2 Qualitative components

At each of the two pilot sites we will undertake:

- Audio-recordings of all 16 initial assessments for PWCF in the intervention arm and 10-12 consultations between the senior interventionist from the MDT (or other MDT member) and PWCF in the control arm. Numbers will depend on numbers of PWCF giving written consent for this.
- 10-12 semi-structured face-to-face (or telephone or skype) interviews with PWCF receiving the intervention and 10-12 semi-structured face-to-face interviews with PWCF in the control arm (total n~40-48 PWCF; n~40-48 interviews);
- two semi-structured face-to-face (or telephone or skype) interviews with each of the two interventionists in each centre (total n=4 interventionists; n=8 interviews); and,
- two semi-structured (face to face, telephone or skype) interviews with two members of the MDT (total n=4 staff; n=8 interviews).

Written informed consent will be obtained from both the interventionist and the PWCF participating in the audio recording when they consent to be in the study. Separate consent will be sought from PWCF and interventionists or members of the wider CF team for semi-structured interviews.

9. Assignment of interventions

9.1 Sequence generation

Participants will be allocated in equal proportions to one of the two groups using a computer generated pseudo-random list, stratified by centre and the number of days participants have been on IV antibiotics in the previous 12 month period as collected at consent visit, with random permuted blocks of varying sizes. The two categories for stratification within the number of IV days will be (i) less than or equal to 14 days and (ii) greater than 14 days.

9.2 Allocation concealment

The allocation sequence will be hosted by the Sheffield CTRU in accordance with their standard operating procedures and will be held on a secure server. Access to the allocation sequence will be restricted to those with authorisation. The sequence will be concealed until recruitment, data collection, and analyses are complete.

9.3 Implementation

The allocation sequence will be created by a Sheffield CTRU statistician who is not otherwise associated with the trial. At the consent visit, a health professional who is named on the delegation log, will go over the patient information sheet again with the study candidate and answer any questions. If the PWCF is still willing to enter the trial, they obtain full written consent and complete the eligibility form. If the participant is eligible, then baseline assessments will be taken. The recruiting health professional will log into the remote, secure Internet-based randomisation system and enter basic demographic information, after which the allocation will be revealed.

9.4 Blinding

After revelation of the allocation, only the statisticians will be blinded to allocation as per CTRU SOPs (ST001 and ST005)

For peer review only - http://bmjopen.bmj.com/site/about/guidelines.xhtml

10. Data collection, management and analysis

10.1 Quantitative data

10.1.1 Data collection methods

Data handling and record keeping. The Sheffield CTRU will oversee data collection, management and analysis and ensure the trial is undertaken according to Good Clinical Practice Guidelines and CTRU standard operating procedures. Data will be collected and retained in accordance with the Data Protection Act 1998. Patients will be reassured that all data which are collected during the course of the research will be kept strictly confidential.

The study team will train those collecting data in the study procedures before the trial begins. Data will either be collected directly from the participants, carers, interventionist, CFHealthHub or from source documents (e.g. patient notes) and input onto the CRF or Sheffield CTRU's electronic web-based data capture system (Prospect). The Data Monitoring and Management Plan for the study will provide further guidance on the types and levels of data and how these will be monitored and verified. Some essential documents may be posted to the central team to facilitate this e.g. participant consent forms in which case this will be detailed in the appropriate participant PIS and consent forms.

The CTRU will perform checks with the participant via monthly phone calls to ensure data is being captured and alert the local interventionist if there is an indication of a prescription change and a need to check pseudomonas (or other microorganism) status. This is required for the correct denominator to assess "normative adherence". Data will be extracted from the CF registry to understand exacerbations in the preceding 12 months since prior exacerbations can have a bearing on the optimum target regimen.

Plans to promote participant retention and complete follow-up.
Participant retention will be ensured by the following procedure:

1. At each point of contact, the interventionist will check with the participant that the Qualcomm hub or Smartphone hub is plugged in and turned on. A member of CTRU who is performing data and prescription checks may alert the interventionist. They will remind the participant of the proximity required for data transfer (10 metres)
2. In the event of no data being displayed in CFHealthHub for a period of at least a week (and the participant is not known to be on holiday) the interventionist will make contact with the participant (Email/Text/Telephone call) to check that the following
    - That the Qualcomm or Smartphone hub is plugged in
    - That the Qualcomm hub is working (showing solid green and yellow lights on the display)
    - That they have been within range of the Qualcomm hub sufficient to facilitate data transfer (10 metres)
    - That the Smartphone hub is switched on (showing the locked 'password' screen when any button is pressed)
    - That the Bi-neb and Smartphone hub have been kept in the same room, or at least have been in close proximity at some point during the day.

Any participants using the Bi-neb who are still experiencing issues after following the steps above, may receive a face to face or telephone support (at home or hospital) from the clinical trainer to resolve any outstanding issues.

Troubleshooting:

For peer review only - http://bmjopen.bmj.com/site/about/guidelines.xhtml

Data capture will be monitored both by interventionist at the site and centrally by the CTRU. In the event of data not being uploaded patients will be contacted to trouble shoot problems. Patients will be offered support to suit their circumstances including home visits (conducted by the members of the site research team) where necessary.

10.1.2 Data Management

Anonymised trial data will be entered onto a validated database system designed to an agreed specification between the Chief Investigator and Sheffield CTRU. The research staff at sites (mainly the ACtiF interventionist) will be responsible for data entry locally. The Sheffield CTRU Trial Manager, research assistant and the Data Management Team will work with sites to ensure the quality of data provided. The study manager, research assistant, data manager, PI's, any research nurses and site interventionist will have access to the anonymised data on the database through the use of usernames and encrypted passwords. The system has a full electronic audit trail and will be regularly backed up. The secure data management system will incorporate quality control procedures to validate the study data. Error reports will be generated where data clarification is needed. Output for analysis will be generated in a format and at intervals to be agreed between Sheffield CTRU and the Chief Investigator.

Trial documents will be retained in a secure location during and after the trial has finished. The study will use the CTRU's in-house data management system (Prospect) for the capture and storage of participant data. Prospect stores all data in a PostgreSQL database on virtual servers hosted by Corporate Information and Computing Services (CiCS) at the University of Sheffield. Prospect uses industry standard techniques to provide security, including password authentication and encryption using SSL/TLS. Access to Prospect is controlled by usernames and encrypted passwords, and a comprehensive privilege management feature can be used to ensure that users have access to only the minimum amount of data required to complete their tasks. This can be used to restrict access to personal identifiable data.

Participants who give consent to the qualitative part of this study will also give consent to their name and address to be given to the University of Sheffield qualitative research staff in order to be contactable.

10.1.3 Data quality assurance

Prospect provides a full electronic audit trail, as well as validation and verification features which will be used to monitor study data quality, in line with CTRU SOPs and the Data Management Plan (DMP). Error reports will be generated where data clarification is required. Rates of missing data and data points which are out of the expected or allowed range will be presented to the team at monthly management group meetings.

For peer review only - http://bmjopen.bmj.com/site/about/guidelines.xhtml

10.2 Qualitative data

10.2.1 Audio recordings of consultations

All initial assessments will be audio recorded with permission (n=16 in each site). Findings from early assessments will be fed back to the interventionist so that changes can be made to the intervention delivery before subsequent assessments. Consultations between the senior interventionist and PWCF in the control arm will be audio recorded with permission (n=10-12 in each site). Encrypted digital recorders will be used and recordings sent securely to the research team for analysis.

10.2.2 Semi-structured interviews: participants

In each site we will interview 3-4 PWCF receiving the intervention who are recruited at the beginning of the pilot. We will interview them around one month into the intervention to seek views of the most intensive part of the intervention. This will identify any problems early and be fed back to the intervention development team, staff delivering the intervention, and trial staff. We will interview 5-6 PWCF around four to six months into the intervention. These PWCF will have experienced more independent use of the CFHealthHub and we can explore how to keep PWCF engaged with the intervention in the longer term. We will interview 2-3 PWCF who drop out of the intervention to explore why this occurred. We will interview 10-12 PWCF in the control arm around four to six months into the trial to explore whether they have experienced aspects of patient activation and leakage of the intervention.

10.2.3 Semi-structured interviews: professionals

The first interviews with the interventionist and senior interventionist in each site will take place after they have undertaken assessments with the first few PWCF to identify teething problems with the intervention or the trial and the comprehensiveness of the training sessions they received. The findings will be fed back to the team to consider whether changes are needed to the intervention or trial protocol. The second interviews will take place when the first few PWCF have completed the intervention to allow the interventionist to reflect back over the whole process. The interventionists may have different lengths of experience of working with CF, nebulisers or behaviour change and we will consider the influence of differences in backgrounds on their ability to implement the intervention.

We will also undertake interviews with two members of the MDT at each centre when the first few PWCF have received 2-3 months of the intervention and then again towards the end of the feasibility study when all PWCF have been recruited and received around 3 months of the intervention.

10.2.4 Undertaking the interviews

For the interviews we have developed topic guides based on our research questions and these are attached to the application. Topic guides develop throughout any qualitative interview study and our topic guides may change as the study progresses. We will audio record all interviews after receiving written permission to do so. We will use an encrypted digital recorder. Reflexive notes will be made during and after the interviews. We expect

For peer review only - http://bmjopen.bmj.com/site/about/guidelines.xhtml

interviews to last around one hour. We do not expect data saturation in pilot studies; the aim is to identify any learning that can be addressed in preparation for the full trial.

11. Data analysis

11.1 Quantitative analysis

The analysis will be performed after data lock by a CTRU statistician under the supervision of the senior study statistician. As the trial is a pragmatic parallel group RCT data will be reported and presented according to the CONSORT 2010 statement [61] with reference to proposed extension for pilot / feasibility studies [37]. As a pilot/feasibility study the main analysis will be mainly descriptive and focus on confidence interval estimation and not formal hypothesis testing [58]. We will report rates of consent, recruitment and follow-up by centre and by randomized group.

Clinical outcome measures will be summarised overall and by randomized group. Baseline demographic (age, gender), physical measurements (e.g. weight, height, BMI), and patient reported outcome measures (EQ-5D, PAM-13, Assessment of Routine, MAD-3, SRBAI, CFQ-R, GAD-7, COM-BMQ, PHQ-8), and clinical measurements (e.g. FEV1, IV days in last registry year) will be described and summarised overall and for both treatment groups.

The primary outcome is the number of pulmonary exacerbations treated with IV antibiotics over the 6 month post-randomisation follow-up period. We will also include, as part of the feasibility analysis, estimation of the effect size for the 6-month pulmonary exacerbations outcome with 95% confidence interval estimates to check that the likely effect is within a clinically relevant range (as confirmation that it is worth progressing with the full trial). For this we will use a Poisson generalised linear model (GLM). Secondary continuous outcomes such as six-month post randomisation FEV1, BMI EQ-5D, PAM-13, Assessment of Routine, MAD-3, SRBAI, CFQ-R, GAD-7, COM-BMQ, PHQ-8) will be analysed with a multiple linear regression model with the baseline value of the outcome and randomised group as covariates. The treatment group coefficient and its associated 95% confidence interval will be reported from the various multiple linear regression models. The mean level of adherence (to prescribed medication) between the intervention and control groups over the 6 month post-randomisation follow-up period will also be reported and compared between the groups and a 95% confidence interval (CI) for the mean difference in this parameter between the randomised groups will also be calculated.

Further analyses with the objective nebuliser data will be performed to explore the process of habit formation with the delivery of the adherence intervention. The analyses will include:

(a) generating objective habit scores by taking into account time of nebuliser use

(b) using statistical process control to identify when periods of stability is achieved

(c) other time-series methods, including cross-correlation between habit scores and adherence.

Adverse events will be based on serious adverse events (SAE) case report forms. A serious adverse event is defined as any adverse event or adverse reaction that results in death, is life-threatening, requires hospitalisation or prolongation of existing hospitalisation, results in persistent or significant disability or incapacity, or is a congenital anomaly or birth defect.

For peer review only - http://bmjopen.bmj.com/site/about/guidelines.xhtml

The following summaries will be presented as overall rates and stratified by AE classification:

- the number and percentages of patients reported as having Serious Adverse Events (SAE) in each treatment arm; and,

- the number and percentages recorded as having all forms of Adverse Events (AE) in each arm.

This information along with the acceptability of the study design and protocol to patients/GPs; the safety of the intervention; patient recruitment and attrition/retention rates will enable us to determine whether or not the definitive RCT is feasible within a satisfactory timescale and cost envelope using UK centres alone.

11.2 Qualitative analysis

Transcripts will be coded using the latest version of NVivo (QSR International). The analysis will use the National Centre for Social Research 'Framework' approach [62]. AO'C and SD will undertake the following stages of the analysis of patient transcripts: familiarisation; identifying a thematic framework; indexing; charting; and, mapping and interpretation. The theoretical framework for understanding intervention adherence is the Necessities-Concerns framework [63] within the COM-B system [18]. This will be used within the thematic framework. We will use the process evaluation functions of context, mechanisms and implementation to frame the analysis [43]. Within mechanisms we will use the COM-B system as stated above and consider the use of the Theoretical Domains Framework [36]. We will compare and contrast findings from each site because the different backgrounds of the interventionists, and the different contexts in which care is provided in each CF unit, may affect implementation and acceptability of the intervention.

Figure 7. Assumptions of the MRC Guidance on Process Evaluation

[39, 64]

For peer review only - http://bmjopen.bmj.com/site/about/guidelines.xhtml

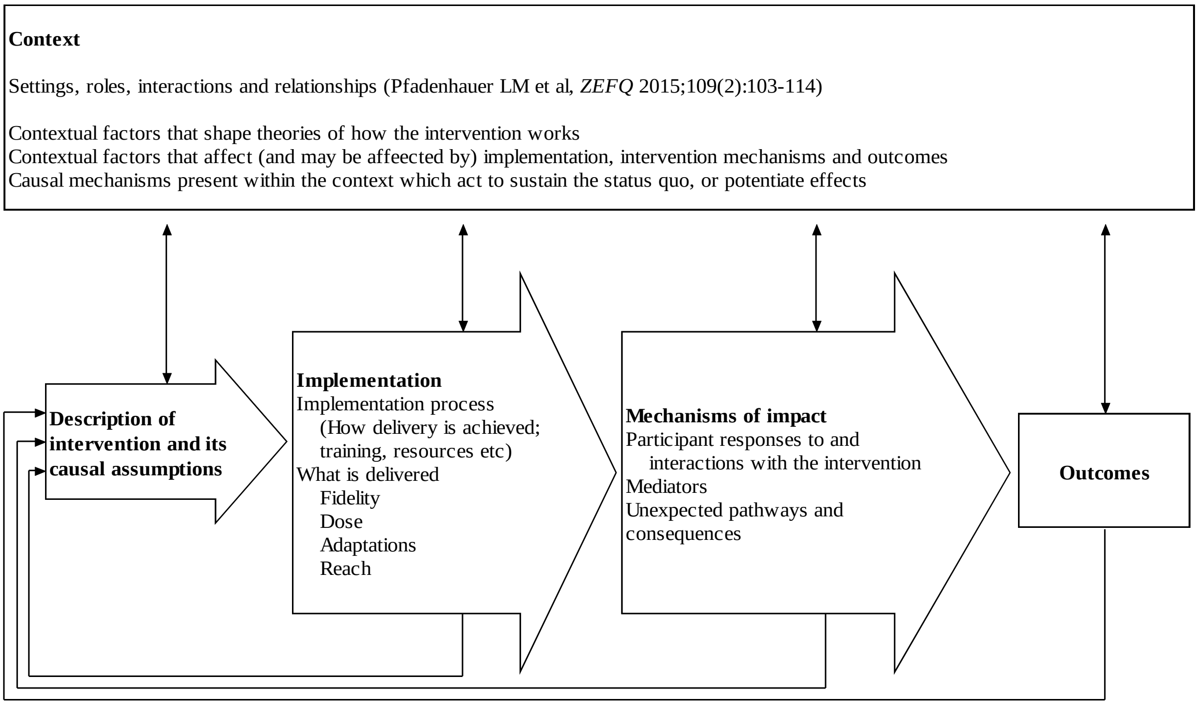

This qualitative research will:

- Inform the refinement of the intervention (e.g. CFHealthHub, training of interventionists, initial assessments, manualised instructions) and its implementation (e.g. introduction within a CF Unit) for use in the full trial.
- Inform refinement to trial procedures for the full trial.
- Inform the selection of the final secondary measures used in the full trial to ensure they address the perceived benefits of the intervention.
- Help to understand the extent of any leakage of the intervention to controls.

11.3 Combining data and findings from the different components

We will use Farmer's triangulation protocol to display the findings from each component of the study together and discuss as a team the extent to which findings converge, complement each other or contradict each other [65, 66]. For example, we will display all findings about recruitment together to consider the feasibility of recruitment for the full trial and the actions required to ensure feasibility. We will also display in a matrix the qualitative and quantitative data for individual PWCF who have received the intervention and been interviewed [66]. We will use this to consider the extent to which our secondary outcome measures identify issues raised by PWCF in the interviews.

12. Monitoring

12.1 Oversight

The CTRU SOP GOV003 Data Monitoring and Ethics Committee states "A DMEC does not need convening in studies that carry low risk to patients". This project involves

For peer review only - http://bmjopen.bmj.com/site/about/guidelines.xhtml

delivering a behaviour change intervention through the website CFHealthHub and would therefore be classified as low risk.

The overall responsibility for the study will be with Sheffield Teaching Hospitals NHS Trust who will act as sponsors for the study. The local Principal Investigator (PI) will be responsible for the study at each participating site and it will be registered and approved with each local R&D department. The study will be conducted in accordance with the protocol, GCP and Sheffield CTRU Standard Operating Procedures. The two committees which will govern the conduct of the study are:

1. Programme Steering Committee (PSC)
2. Project Management Group (PMG)

The PSC will be responsible for the overall conduct of the trial and consists of an independent chair and four other independent members including a statistician and PPI representative. The committee will meet every 6 months to monitor the study.

The PMG will comprise of the trial manager and the core research team . The PMG will meet on a monthly basis to monitor the day-to-day running of the trial. The Trial Manager will be jointly supervised by the CI and the Assistant Director of CTRU via the form of regular meetings (face to face and telephone calls). The Trial Manager will be responsible for liaising with the whole project team. Trial monitoring procedures will be assessed based on the level of risk of the study. The Site Monitoring Plan will outline the types and frequency of site monitoring activities for the study and this will be agreed with the Sponsor prior to the start of the study.

12.2 Description of any interim analyses and stopping guidelines

There are no planned interim analyses or stopping guidelines for this study.

12.3 Harms (safety assessments)

12.3.1 Serious Adverse Events

Trial sites are to report Serious Adverse Events (SAEs) in conjunction with the CTRU standard operating procedure PM004 (Adverse events and serious adverse events). The definition of an SAE is as follows:

- results in death;
- is life-threatening* (subject at immediate risk of death);
- requires in-patient hospitalisation or prolongation of existing hospitalisation;**
- results in persistent or significant disability or incapacity;
- consists of a congenital anomaly or birth defect; or,
- is another important medical event that may jeopardise the subject.***

* ‘life-threatening’ refers to an event in which the patient was at risk of death at the time of the event; it does not refer to an event which hypothetically might have caused death if it were more severe.
**Hospitalisation is defined as an inpatient admission, regardless of length of stay, even if the hospitalisation is a precautionary measure for continued observation. Hospitalisations

For peer review only - http://bmjopen.bmj.com/site/about/guidelines.xhtml

for a pre-existing condition, including elective procedures that have not worsened, do not constitute an SAE.
***Other important medical events that may not result in death, be life-threatening, or require hospitalisation may be considered a serious adverse event/experience when, based upon appropriate medical judgment, they may jeopardise the subject and may require medical or surgical intervention to prevent one of the outcomes listed in this definition.

It is not anticipated that there will be many SAEs related to the behaviour change intervention. We will report any SAEs which are deemed related to the trial intervention and unexpected to the Sponsor within the specified timeframes below (12.3.4).

12.3.2 Adverse events we require reporting:

We do require that sites report any new diagnosis of depression which requires treatment with medication or psychological therapy e.g. Cognitive Behavioural Therapy (CBT).

12.3.3 Expected SAEs and adverse events

Certain adverse events are common to CF and associated medications. Expected SAEs must be reported in the annual safety report. Hospitalisation as a result of an exacerbation will be recorded in the study database and not be reported as an SAE.

Expected AEs in relation to medications or common in patients with CF

1. Acute FEV1 drop >15% after 1st dose of medication
2. Increased productive cough
3. Nasal congestion or stuffy nose
4. Chest congestion
5. Wheezing
6. Chest pain or chest discomfort
7. Voice alteration/change
8. Dysponea (breathlessness)
9. Haemoptysis (coughing blood)
10. Rhinitis
11.
12. Headache
13. Crackles in lung
14. Throat irritation/ sore throat
15. URTI
16. Sinusitis
17. Deafness
18. Indigestion / reflux
19. Tonsillitis
20. Joint pain
21. Decreased appetite
22. Fatigue
23. Headache

For peer review only - http://bmjopen.bmj.com/site/about/guidelines.xhtml

24. Distal intestinal obstructive syndrome
25. Fever
26. Otitis media or ear infection
27. Conjunctivitis
28. Pneumothorax
29. Decreased exercise tolerance
30. Pyrexia
31. Abdominal pain
32. Influenza
33. Pseudomonas infection
34. Vomiting
35. Diabetes
36. Pneumonia

12.3.4 Reporting

Adverse events and SAEs can be reported for participants at any stage of their trial participation. A member of the site study team (interventionist, clinician or other) will enquire about any adverse events at routine clinic appointments. These will be record on the adverse event section of the paper CRF and database. The event will be assessed by the local Principal Investigator and the form will be kept in the site file. Serious adverse events will be reported in the periodic safety reports to the research ethics committee and Trial Steering committee.

All adverse events (serious or other based on the definitions above) will be recorded on the case report form and details will be **entered on the study database within 1 week of completing the paper form**. Any SAEs which are deemed related to the trial intervention, the site will complete the paper CRF and **fax details this form to the CTRU within 24 hours of becoming aware of the event** in order for the CTRU to report this event to the Sponsor and the main REC within the required timeframes (15 days).

In participants using the Bi-Neb, any Adverse or SAEs relating to the use of Promixin via this device will be reported to the Patient Support team (PSP) at Phillips as per their standard practice.

12.4 Auditing

The sponsor will permit monitoring and audits by the relevant authorities, including the Research Ethics Committee. The investigator will also allow monitoring and audits by these bodies and the sponsor, and they will provide direct access to source data and documents.

12.5 Finance and indemnity

The trial has been financed by the NIHR and details have been drawn up in a separate agreement. This is an NHS sponsored study. If there is negligent harm during the clinical

For peer review only - http://bmjopen.bmj.com/site/about/guidelines.xhtml

trial when the NHS body owes a duty of care to the person harmed, NHS Indemnity will cover NHS staff, medical academic staff with honorary contracts and those conducting the trial. NHS Indemnity does not offer no-fault compensation and is unable to agree in advance to pay compensation for non-negligent harm. Ex-gratia payments may be considered in the case of a claim.

13. Ethics and dissemination

13.1 Approvals

The trial will be conducted subject to Research Ethics Committee favourable opinion including any provisions for site specific assessment. The application will be submitted through the IRAS central allocation system. The approval letter from the ethics committee and copy of approved patient information leaflets, consent forms and any ethically approved questionnaires will be present in the site files before initiation of the study and patient recruitment. Local research governance approvals will be sought from all participating research sites. This clinical trial will be conducted in accordance with Good Clinical Practice Guidelines and CTRU standard operating procedures. MHRA approval is not required for this study.

13.2 Protocol amendments

The investigator will be updated following an amendment to the protocol or study documents. The new documents, REC approval, R&D approval, HRA assessment letter and any other appropriate documentation surrounding the amendment will be sent to the site via a "site file update". The sites will receive the documents with a site file update sheet, detailing where to file the amended documents and which documents to supersede. If there are any significant changes to the study procedures or eligibility criteria sites will be notified by a combination of email, telephone, newsletters or additional project training when required.

In relation to informing REC, if any study documents require amending, the changes will be discussed with the sponsor and either a substantial (via IRAS and HRA) or minor amendment (notification via email) will be submitted to REC and HRA. Following REC acknowledgment and approval (when applicable) other appropriate approvals will be obtained i.e. HRA and R&D approval.

If a protocol amendment requires participants to be re-consented they will be informed of the amendment by an updated participant information sheet and will be asked to re-consent to the study. Trial registries, journals and regulators will be updated regarding protocol amendments when appropriate.

13.3 Consent

Consent for the main trial:

The ACtiF trial interventionist or local PI at the site will be responsible for taking informed consent from potentially eligible trial participants face to face at home or in clinic. Any researcher or clinical member of the team taking informed consent will be trained in study procedures and GCP. Participants will have the option to specify whether they are

For peer review only - http://bmjopen.bmj.com/site/about/guidelines.xhtml

interested in being approached for the qualitative interviews and audio recordings. However, they do not have to consent to these to be involved in the main study.

Consent for the interviews:
Consent for interviews (participant, interventionist or MDT member) will separately be taken by the qualitative researcher. Participants can participate in the main trial but choose to not take part in the qualitative research.

13.4 Confidentiality

Participant confidentiality will be respected at all times. Participant names and contact details will be collected and entered on the prospect database. Access to these personal details will be restricted to users with appropriate privileges only. All users who do not require access to identifiable data will only identify data by participant ID number, and no patient identifiable data will be transferred from the database to the statistician.

Trial documents (paper and electronic) will be retained in a secure location during and after the trial has finished. All source documents will be retained for a period of 5 years following the end of the trial. Where trial related information is documented in the medical records – those records will be retained for 5 years after the last patient last visit. Each site is responsible for ensuring records are archived and the information supplied to the Chief Investigator.

Any participant data held within CFHealthHub will be stored on a secure server at the University of Manchester. CFHealthHub complies with the Data Protection Act and follows best practice guidelines on security and information governance. Encrypted channels are used to transfer any data to and from the web and mobile application platforms. All user interaction with the CFHealthHub server and each action performed by a user will be logged. An audit log contains the username of the user performing the action, the date & time of the action, short description of the action performed. All users are authenticated via a secure password a with access to the system restricted on a role basis.

13.5 Declaration of Interests

Martin Wildman has received funding from Zambon who market the Ineb to carry out research to understand the performance of the Ineb and in the past we received funding from Zambon to carry out work to understand barriers to adherence.

13.6 Access to data

The central ACtiF study team alone will have access to the final dataset details of which will be outlined in the study DMP.

13.7 Ancilliary and post-trial care

Centres will be able to continue to use CFHealthHub if they wish to do so after the end of the pilot and feasibility study. If so, participants in the control arm will be able to cross over to use the intervention at this stage.

13.8 Dissemination policy

As this is a feasibility study its main interest will be to potential researchers and funding bodies. Data will be reported according to the revised CONSORT statement (Schultz,

For peer review only - http://bmjopen.bmj.com/site/about/guidelines.xhtml

2010). The findings of this research will be available to NIHR, patient groups and other interested bodies. It will also be offered for presentation at medical meetings and will be offered for publication in peer reviewed medical journals.

For peer review only - http://bmjopen.bmj.com/site/about/guidelines.xhtml

References

1. Alexander BM, Petren EK, Grimes M, Fink A, Myers V, Sewall A: **Mission of the cystic fibrosis foundation Annual Data Report 2013 Cystic Fibrosis Foundation Patient Registry**. 2014.

2. Southern KW, Barker PM, Solis-Moya A, Patel L: **Macrolide antibiotics for cystic fibrosis**. In *Cochrane Database of Systematic Reviews*. Edited by Southern KW. Chichester, UK: John Wiley & Sons, Ltd; 2011.

3. Ramsey BW, Davies J, McElvaney NG, Tullis E, Bell SC, Dřevínek P, Griese M, McKone EF, Wainwright CE, Konstan MW, Moss R, Ratjen F, Sermet-Gaudelus I, Rowe SM, Dong Q, Rodriguez S, Yen K, Ordoñez C, Elborn JS: **A CFTR Potentiator in Patients with Cystic Fibrosis and the *G551D* Mutation**. *N Engl J Med* 2011, **365**:1663–1672.

4. McCoy KS, Quittner AL, Oermann CM, Gibson RL, Retsch-Bogart GZ, Montgomery AB: **Inhaled aztreonam lysine for chronic airway Pseudomonas aeruginosa in cystic fibrosis.** *Am J Respir Crit Care Med* 2008, **178**:921–8.

5. Ryan G, Mukhopadhyay S, Singh M: **Nebulised anti-pseudomonal antibiotics for cystic fibrosis**. In *Cochrane Database of Systematic Reviews*. Edited by Ryan G. Chichester, UK: John Wiley & Sons, Ltd; 2003.

6. Jones AP, Wallis C: **Dornase alfa for cystic fibrosis**. In *Cochrane Database of Systematic Reviews*. Edited by Jones AP. Chichester, UK: John Wiley & Sons, Ltd; 2010.

7. Wark P, McDonald VM: **Nebulised hypertonic saline for cystic fibrosis**. In *Cochrane Database of Systematic Reviews*. Edited by Wark P. Chichester, UK: John Wiley & Sons, Ltd; 2009.

8. Ryan G, Singh M, Dwan K: **Inhaled antibiotics for long-term therapy in cystic fibrosis**. In *Cochrane Database of Systematic Reviews*. Edited by Ryan G. Chichester, UK: John Wiley & Sons, Ltd; 2011.

9. Eakin MN, Bilderback A, Boyle MP, Mogayzel PJ, Riekert KA: **Longitudinal association between medication adherence and lung health in people with cystic fibrosis**. *J Cyst Fibros* 2011, **10**:258.

10. Briesacher BA, Quittner AL, Saiman L, Sacco P, Fouayzi H, Quittell LM, Accurso F, Modi A, Lim C, Yu N, Geller D, Wagner M, Quittner A, DiMatteo M, Quittner A, Barker D, Marciel K, Grimley M, Emerson J, Rosenfeld M, McNamara S, Ramsey B, Gibson R, Nixon G, Armstrong D, Carzino R, Carlin J, Olinsky A, Robertson C, Grimwood K, et al.: **Adherence with tobramycin inhaled solution and health care utilization**. *BMC Pulm Med* 2011, **11**:5.

11. AL Q, KA R, Zhang J: **Relationship between adherence to pulmonary medications and health care costs: longitudinal analyses from 2005-2011.** *Pediatr Pulmonol* 2012, **47**:198.

12. Smyth A, Lewis S, Bertenshaw C, Choonara I, McGaw J, Watson A: **Case-control study of acute renal failure in patients with cystic fibrosis in the UK.** *Thorax* 2008, **63**:532–5.

For peer review only - http://bmjopen.bmj.com/site/about/guidelines.xhtml

13. McManus P: *Lead Pharmacist - Specialised Services (South Yorkshire and Bassetlaw) NHS England*. .

14. Daniels T, Goodacre L, Sutton C, Pollard K, Conway S, Peckham D: **Accurate assessment of adherence: self-report and clinician report vs electronic monitoring of nebulizers.** *Chest* 2011, **140**:425–32.

15. McNeil S: *Personal Communication from Stephanie McNeil, Chief Statistician of UK CF Registry*. .

16. Horne RW, Barber N, Elliot R, Morgan M: *Concordance, Adherance and Compliance in Medicine Taking: A Conceptual Map and Research Priorities. National Co-Ordinating Centre for NHS Service Delivery and Organisational R&D (NCCSDO)*. 2005.

17. Craig P, Dieppe P, Macintyre S, Michie S, Nazareth I, Petticrew M: **Developing and evaluating complex interventions: the new Medical Research Council guidance**. *BMJ* 2008:a1655–a1655.

18. Michie S, van Stralen MM, West R: **The behaviour change wheel: a new method for characterising and designing behaviour change interventions.** *Implement Sci* 2011, **6**:42.

19. Parham R, Thomas S, Mills R, Al. E: **CF patient's beliefs about nebuliser treatment: implications for adherence to treatment**. *J Cyst Fibros* 2012, **11**(Suppl 1):S9.

20. Bucks RS, Hawkins K, Skinner TC, Horn S, Seddon P, Horne R: **Adherence to treatment in adolescents with cystic fibrosis: the role of illness perceptions and treatment beliefs.** *J Pediatr Psychol* 2009, **34**:893–902.

21. George M, Rand-Giovannetti D, Eakin MN, Borrelli B, Zettler M, Riekert KA: **Perceptions of barriers and facilitators: self-management decisions by older adolescents and adults with CF.** *J Cyst Fibros* 2010, **9**:425–32.

22. Dziuban EJ, Saab-Abazeed L, Chaudhry SR, Streetman DS, Nasr SZ: **Identifying barriers to treatment adherence and related attitudinal patterns in adolescents with cystic fibrosis.** *Pediatr Pulmonol* 2010, **45**:450–8.

23. Bregnballe V, Schiøtz PO, Boisen KA, Pressler T, Thastum M: **Barriers to adherence in adolescents and young adults with cystic fibrosis: a questionnaire study in young patients and their parents.** *Patient Prefer Adherence* 2011, **5**:507–15.

24. Modi AC, Quittner AL: **Barriers to treatment adherence for children with cystic fibrosis and asthma: What gets in the way?** *J Pediatr Psychol* 2006, **31**:846–858.

25. Abbott J, Dodd M, Bilton D, Webb AK: **Treatment compliance in adults with cystic fibrosis.** *Thorax* 1994, **49**:115–20.

26. Abbott J, Dodd M, Webb AK: **Health perceptions and treatment adherence in adults with cystic fibrosis.** *Thorax* 1996, **51**:1233–8.

27. Abbott J, Dodd M, Gee L, Webb K: **Ways of coping with cystic fibrosis: implications for treatment adherence.** *Disabil Rehabil* 2001, **23**:315–24.

28. Conway SP, Pond MN, Hamnett T, Watson A: **Compliance with treatment in adult patients with cystic fibrosis.** *Thorax* 1996, **51**:29–33.

For peer review only - http://bmjopen.bmj.com/site/about/guidelines.xhtml

29. Jones S, Curley R, Wildman M: **345 Systematic review of qualitative studies investigating barriers to adherence in patients with cystic fibrosis using framework analysis structured by a conceptual framework of behaviour change**. *J Cyst Fibros* 2013, **12**(Suppl 1):S136.

30. Kettler LJ, Sawyer SM, Winefield HR, Greville HW: **Determinants of adherence in adults with cystic fibrosis.** *Thorax* 2002, **57**:459–64.

31. Lask B: **Non-Adherence to Treatment in Cystic Fibrosis**. *J R Soc Med* 1994, **87**(21 Suppl):25–27.

32. Latchford G, Duff A, Quinn J, Conway S, Conner M: **Adherence to nebulised antibiotics in cystic fibrosis.** *Patient Educ Couns* 2009, **75**:141–4.

33. Horne R, Weinman J, Barber N, Elliott R: *Concordance , Adherence and Compliance in Medicine Taking: Report for the National Co-Ordinating Centre for NHS Service Delivery and Organisation R & D (NCCSDO)*. London: NCCSDO; 2005.

34. Ivers N, Jamtvedt G, Flottorp S, Young JM, Odgaard-Jensen J, French SD, O'Brien MA, Johansen M, Grimshaw J, Oxman AD: **Audit and feedback: effects on professional practice and healthcare outcomes.** *Cochrane database Syst Rev* 2012, **6**:CD000259.

35. Geraedts H, Zijlstra A, Bulstra SK, Stevens M, Zijlstra W: **Effects of remote feedback in home-based physical activity interventions for older adults: a systematic review.** *Patient Educ Couns* 2013, **91**:14–24.

36. Cane J, O'Connor D, Michie S: **Validation of the theoretical domains framework for use in behaviour change and implementation research.** *Implement Sci* 2012, **7**:37.

37. Thabane L, Ma J, Chu R, Cheng J, Ismaila A, Rios LP, Robson R, Thabane M, Giangregorio L, Goldsmith CH: **A tutorial on pilot studies: the what, why and how.** *BMC Med Res Methodol* 2010, **10**:1.

38. Baranowski T, Stables G: **Process Evaluations of the 5-a-Day Projects**. *Heal Educ Behav* 2000, **27**:157–166.

39. Pfadenhauer LM, Mozygemba K, Gerhardus A, Hofmann B, Booth A, Lysdahl KB, Tummers M, Burns J, Rehfuess EA: **Context and implementation: A concept analysis towards conceptual maturity**. *Z Evid Fortbild Qual Gesundhwes* 2015, **109**:103–114.

40. Weber K, Glynn M a.: **Making Sense with Institutions: Context, Thought and Action in Karl Weick's Theory**. *Organ Stud* 2006, **27**:1639–1660.

41. Hyatt D: **Time for a change: a critical discoursal analysis of synchronic context with diachronic relevance**. *Discourse Soc* 2005, **16**:515–534.

42. Linnan L, Steckler A: **Process evaluation for public health interventions and research: an overview**. In *Process evaluation for public health interventions and research*. 1st edition. Edited by Linnan L, Steckler A. San Francisco: Jossey-Bass; 2002:1–23.

43. Moore GF, Audrey S, Barker M, Bond L, Bonell C, Hardeman W, Moore L, O'Cathain A, Tinati T, Wight D, Baird J: **Process evaluation of complex interventions: Medical Research Council guidance.** *BMJ* 2015, **350**:h1258.

For peer review only - http://bmjopen.bmj.com/site/about/guidelines.xhtml

44. Nilsen P: **Making sense of implementation theories, models and frameworks**. *Implement Sci* 2015, **10**:1–13.

45. Meyers DC, Durlak J a., Wandersman A: **The Quality Implementation Framework: A Synthesis of Critical Steps in the Implementation Process**. *Am J Community Psychol* 2012, **50**:462–480.

46. Borrelli B: **The Assessment, Monitoring, and Enhancement of Treatment Fidelity In Public Health Clinical Trials.** *J Public Health Dent* 2011, **71**:S52–S63.

47. Fuchs HJ, Borowitz DS, Christiansen DH, Morris EM, Nash ML, Ramsey BW, Rosenstein BJ, Smith AL, Wohl ME: **Effect of aerosolized recombinant human DNase on exacerbations of respiratory symptoms and on pulmonary function in patients with cystic fibrosis. The Pulmozyme Study Group.** *N Engl J Med* 1994, **331**:637–42.

48. Ratjen F, Durham T, Navratil T, Schaberg A, Accurso FJ, Wainwright C, Barnes M, Moss RB: **Long term effects of denufosol tetrasodium in patients with cystic fibrosis.** *J Cyst Fibros* 2012, **11**:539–49.

49. Miller MR, Hankinson J, Brusasco V, Burgos F, Casaburi R, Coates A, Crapo R, Enright P, van der Grinten CPM, Gustafsson P, Jensen R, Johnson DC, MacIntyre N, McKay R, Navajas D, Pedersen OF, Pellegrino R, Viegi G, Wanger J: **Standardisation of spirometry.** *Eur Respir J* 2005, **26**:319–38.

50. Herdman M, Gudex C, Lloyd A, Janssen M, Kind P, Parkin D, Bonsel G, Badia X: **Development and preliminary testing of the new five-level version of EQ-5D (EQ-5D-5L).** *Qual Life Res* 2011, **20**:1727–36.

51. Hibbard JH, Mahoney ER, Stockard J, Tusler M: **Development and testing of a short form of the patient activation measure.** *Health Serv Res* 2005, **40**(6 Pt 1):1918–30.

52. Wong MD, Sarkisian C a., Davis C, Kinsler J, Cunningham WE: **The Association Between Life Chaos, Health Care Use, and Health Status Among HIV-Infected Persons**. *J Gen Intern Med* 2007, **22**:1286–1291.

53. Verplanken B, Orbell S: **Reflections on Past Behavior: A Self-Report Index of Habit Strength1**. *J Appl Soc Psychol* 2003, **33**:1313–1330.

54. Quittner a L, Sweeny S, Watrous M, Munzenberger P, Bearss K, Gibson Nitza a, Fisher L a, Henry B: **Translation and linguistic validation of a disease-specific quality of life measure for cystic fibrosis.** *J Pediatr Psychol* 2000, **25**:403–14.

55. Kroenke K, Strine TW, Spitzer RL, Williams JBW, Berry JT, Mokdad AH: **The PHQ-8 as a measure of current depression in the general population**. *J Affect Disord* 2009, **114**:163–173.

56. Spitzer RL, Kroenke K, Williams JBW, Löwe B: **A brief measure for assessing generalized anxiety disorder: the GAD-7.** *Arch Intern Med* 2006, **166**:1092–7.

57. Horne R, Weinman J, Hankins M: **The beliefs about medicines questionnaire: The development and evaluation of a new method for assessing the cognitive representation of medication**. *Psychol Health* 1999, **14**:1–24.

58. Lancaster GA, Dodd S, Williamson PR: **Design and analysis of pilot studies:**

For peer review only - http://bmjopen.bmj.com/site/about/guidelines.xhtml

recommendations for good practice. *J Eval Clin Pract* 2004, **10**:307–12.

59. Arain M, Campbell MJ, Cooper CL, Lancaster GA: **What is a pilot or feasibility study? A review of current practice and editorial policy.** *BMC Med Res Methodol* 2010, **10**:67.

60. Eldridge S, Bond C, Campbell M, Lancaster G, Thabane L, Hopwell S: **Definition and reporting of pilot and feasibility studies**. *Trials* 2013, **14**(Suppl 1):O18.

61. Schulz KF, Altman DG, Moher D: **CONSORT 2010 statement: Updated guidelines for reporting parallel group randomized trials**. *Annu Intern Med* 2010, **152**:726–732.

62. Ritchie J, Spencer L: **Qualitative data analysis for applied policy research**. In *Analysing qualitative data*. Edited by Bryman A, Burgess RG. Routledge; 1994:173–194.

63. Horne R: **Treatment perceptions and self-regulation**. In *The Self-regulation of Health and Illness Behaviour*. Edited by Cameron L, Leventhal H. London: Routledge; 2003:138–53.

64. Moore GF, Audrey S, Barker M, Bond L, Bonell C, Hardeman W, Moore L, O'Cathain A, Tinati T, Wight D, Baird J: **Process evaluation of complex interventions: Medical Research Council guidance**. *BMJ* 2015, **350**(mar19 6):h1258–h1258.

65. Farmer T, Robinson K, Elliott SJ, Eyles J: **Developing and implementing a triangulation protocol for qualitative health research.** *Qual Health Res* 2006, **16**:377–94.

66. O'Cathain A, Murphy E, Nicholl J, Cathain AO: **Three techniques for integrating data in mixed methods studies**. *BMJ* 2010, **341**(sep17 1):c4587–c4587.

For peer review only - http://bmjopen.bmj.com/site/about/guidelines.xhtml

Appendix 1. W.H.O. Trial Registration Data Set

DATA CATEGORY	INFORMATION
Primary registry and trial identifying number	To be added
Date of registration in primary registry	To be added
Secondary identifying numbers	NIHR: RP-PG-1212-20015<br>Sponsor (STH): STH19213
Source(s) of monetary or material support	National Institute for Health Research (NIHR) Programme Grants for Applied Research programme.
Primary sponsor	Sheffield Teaching Hospitals NHS Foundation Trust.
Secondary sponsor(s)	none
Contact for public queries	Chin Maguire<br>Trial Manager<br>Clinical Trials Research Unit<br>University of Sheffield<br>Regent Court<br>30 Regent Street<br>Sheffield<br>S1 4DA<br>Tel: (+44) (0)114 222 0717<br>Fax: (+44) (0)114 222 0870<br>email : c.maguire@sheffield.ac.uk
Contact for scientific queries	Dr Martin Wildman<br>Adult CF Centre<br>Northern General Hospital<br>Herries Road<br>Sheffield<br>S5 7AU<br>Tel: (0114) 2715212<br>Fax: (0114) 222 0870<br>email : Martin.Wildman@sth.nhs.uk
Public title	Adherence to treatment in adults with Cystic Fibrosis (ACtiF)
Scientific title	Development and evaluation of an intervention to support Adherence to treatment in adults with Cystic Fibrosis : a feasibility study comprised of an external pilot randomised controlled trial and process evaluation
Countries of recruitment	United Kingdom
Health condition(s) or problem(s) studied	Cystic Fibrosis
Intervention(s)	Usual care plus a microchipped nebuliser with or without a complex intervention. The complex intervention consists of:

For peer review only - http://bmjopen.bmj.com/site/about/guidelines.xhtml

	- A software platform, CFHealthHub mobile apps and website, which allows access to medication adherence data and education modules intended to remove barriers to adherence<br>- A manual containing a 'behaviour change toolkit' to guide interactions between health
Key inclusion and exclusion criteria	Inclusion criteria for participants<br>1.Diagnosed with CF and with data within the CF registry<br>2.Aged 16 years and above<br>3.Taking inhaled mucolytics or antibiotics via a chipped nebuliser (e.g. eTrack or Bi-Neb) or able and willing to take via eTrack or Bi-Neb.<br><br>Exclusion criteria for participants<br>1.Post-lung transplant<br>2.People on the active lung transplant list<br>3.Patients receiving palliative care,<br>4.Lacking in capacity to give informed consent<br>5.Using dry powder devices to take antibiotics or mucolytics
Study type	Feasibility study comprised of an external pilot randomised controlled trial and process evaluation
Date of first enrolment	Anticipated: 02/05/2016
Target sample size	We propose to recruit to time, that is for a fixed period of four months rather than to a fixed sample size. To match the proposed recruitment rate of the main RCT, the target sample over the four months for which the pilot RCT is open, will be n=64.
Recruitment status	Not yet open.
Primary outcome(s)	Exacerbations of cystic fibrosis as defined by the Fuchs criteria (*N Engl J Med* 1994, 331:637–42.)
Key secondary outcomes	None.

For peer review only - http://bmjopen.bmj.com/site/about/guidelines.xhtml

<u>ACtiF Pilot Study</u>

<u>Control Patient Topic guide</u>

Thank you for agreeing to take part in the interview today

Check timing ok

Check consent form filled in / received

Are you happy for the interview to be recorded?

We're interested in your experiences of the service that you receive for helping you to use your nebuliser.

1. Why did you decide to take part in the research?

2. How did you find being asked to take part in the trial? [Prompts: paperwork volume, information provided, questionnaires]

Now I'd like to ask you about the care you received before the trial started to help you use your nebuliser.

3. What types of things did the unit/hospital recommend that you do to help you use your nebuliser? [Prompts: appointments / what do you talk about? / nebulisers / skills to use your nebuliser properly / knowledge and beliefs?]

4. What types of things did the unit/hospital recommend that you do to help you use your nebuliser as much as possible? [Prompts: setting goals, solving problems, making plans, giving you information, building skills, beliefs about nebuliser medication, giving you confidence]

5. How did the care you received to help you use your nebuliser fit with any other care you received for CF more generally?

For peer review only - http://bmjopen.bmj.com/site/about/guidelines.xhtml

For peer review only

6. How could the care you received for helping you to use your nebuliser as prescribed be improved?

7. Overall how happy are you with the care you received for your nebuliser? [Prompts: what could be done better?]

Now I want to ask you about specific kinds of things that might have changed since the trial started:

8. Since you joined the trial has the care that you receive in the unit / hospital changed at all? [Prompts: Has anybody done anything different? What have they done?]

9. Since you joined the trial has anyone asked you to change how you use your nebuliser? If so, what have they suggested you do? [Prompt: capability skills / knowledge including beliefs / where has the change come from?]

10. Since you joined the trial has anyone suggested ways to help you use your nebuliser as much as possible? If so what? [Prompt: opportunity finding time to use nebuliser / making plans / setting goals / where has the change come from?]

11. Since you joined the trial has anyone helped you have more confidence to use your nebuliser as prescribed? [Prompt: where has the change come from? what have they done?]

Is there anything else that we haven't talked about that you'd like to comment on?

THANK YOU

The ACtiF Project is funded by the National Institute for Health Research's Programme Grants for Applied Research.

For peer review only - http://bmjopen.bmj.com/site/about/guidelines.xhtml

For peer review only

ACtiF Pilot Study

Intervention Patient Topic guide

Thank you for agreeing to take part in the interview today

Check timing ok

Check consent form filled in / received

Are you happy for the interview to be recorded?

We're interested in your experiences of the service that you have received from CFHealthHub including both the meetings to discuss your nebuliser medication and the website/app you have used.

1. Why did you decide to take part in the research?

2. How did you find being asked to take part in the trial? [Prompts: recruitment, paperwork volume, information provided, questionnaires]

Now I'd like to ask you about your meetings with the person who has been working with you on CFHealthHub.

3. What types of things did they recommend that you do? [prompts: setting goals, solving problems, making plans (myplan), giving you information]

4. Do you think you have had any benefit from these meetings?

If yes, what benefit and what about the service helped you to get this?

If no, what has stopped you gaining benefit?

5. What was good about how the meetings were delivered? [Prompt: what needs to be improved?]

For peer review only - http://bmjopen.bmj.com/site/about/guidelines.xhtml

Now I'd like to talk to you about the CFHealthHub website / app.

6. What was good about the website? [Prompts: my plan, how am I doing, tool kit, graphs, my treatment]

7. What needs to be improved? [Prompts: my plan, how am I doing, tool kit, graphs, my treatment]

8. Do you think you've had any benefit from using the website?

   If yes, what benefit and what about the website helped you to get this?

   If no, what has stopped you gaining benefit?

9. Have the website and/or meetings helped you to improve how often you use your nebuliser?

   If yes, how has it helped you to do this?

   If not, why not?

10. How do the CFHealthHub website and the meetings work together?

11. Has using CFHealthHub helped you to be able to use your nebuliser any better? Why / Why not? [Prompt: capability skills / knowledge including beliefs]

12. Has using CFHealthHub helped you to find the time to use your nebuliser more? Why / why not? [Prompt: opportunity / making plans]

13. Has using CFHealthHub made you want to use your nebuliser more? Why / why not? [Prompt: motivation and confidence]

14. How does the CFHealthHub service (website and meetings) fit with the care you were already receiving at the unit/hospital?

For peer review only - http://bmjopen.bmj.com/site/about/guidelines.xhtml

For peer review only

15. Do you think you would continue using CFHealthHub? [Prompt: during the study / after the study]

16. Is CFHealthHub a good thing to use in general for people with CF? Why? / Why not?

17. How have you found being part of the study?

Is there anything else that we haven't talked about that you'd like to comment on?

THANK YOU

The ACtiF Project is funded by the National Institute for Health Research's Programme Grants for Applied Research.

For peer review only - http://bmjopen.bmj.com/site/about/guidelines.xhtml

ACTIF Pilot Study

Interventionist Topic guide

Thank you for agreeing to take part in the interview today.

Check timing ok

Check consent form filled in / received

Are you happy for the interview to be recorded?

Introduction to the interview: Interested in how you've found using CFHealthhub (CFHH) with your participants and any learning from it

The trial

1. What works or could be improved about:
   a) recruiting patients to the trial?
   b) collecting data?
   c) any other aspect?

The intervention:

Now I'd like to go through each of the steps for providing the intervention to get your views on each of these

2. What works or could be improved about:
   a) how you have assessed participants' adherence levels prior to using CFHH?

   b) how you set up appointments with your participants?

   c) session 1? [Prompts: gathering data, introducing the nebuliser, entering prescription data into CFHH, completion of screening tools, patient feedback, anything else]

For peer review only - http://bmjopen.bmj.com/site/about/guidelines.xhtml

d) session 2? [Prompts: reviewing adherence data, introducing CFHH, explaining modules, setting goals, action planning, identifying suitable tailored content, technical issues, anything else]

e) session 3? [Prompts: reviewing goals, reviewing adherence plans, motivation, problem solving, anything else]

3. What works or could be improved about the training manuals and training sessions?

4. What works or could be improved about the support available from the research team? [Prompts: timing, availability, problem solving].
[**Specific prompt for MDT senior interventionist**: do you think the training has equipped you to deliver this intervention in your centre yourself after the trial ends? If no, what further training would be needed?]

5. How has the CFHH intervention been received by the rest of the team? [Prompt: how has your communication been with the rest of the team about CFHH?]

6. What sort of follow-up did participants request? How will you handle this?

7. How has the CFHH intervention helped your participants to know how to use their nebuliser? [Prompt: capability / skills, knowledge and beliefs]

8. How has the CFHH intervention helped your participants find ways to use their nebuliser more? [Prompt: opportunity]

9. How has the CFHH intervention helped to motivate your participants to use their nebuliser? [Prompt: motivation / confidence]

General questions:

10. How engaged did participants seem with CFHH? [Prompt: What feedback if any have you had from participants about CFHH?]

11. How useful do you think CFHH is for your participants?

For peer review only - http://bmjopen.bmj.com/site/about/guidelines.xhtml

12. How easy / difficult has it been to get your participants to use CFHH?

13. Have you seen any changes to the ways in which your participants use their nebulisers since starting CFHH?

14. What have you learnt from using CFHH with your participants?

15. What if any are the benefits to you and / or to your participants of using CFHH?

16. How do you think CFHH fits with the other care offered by the centre?

17. How have you found being part of the trial?

Is there anything else you'd like to say about CFHH?

THANK YOU

The ACtiF Project is funded by the National Institute for Health Research's Programme Grants for Applied Research.

For peer review only - http://bmjopen.bmj.com/site/about/guidelines.xhtml
For peer review only

ACTIF Pilot Study

MDT Topic guide

Thank you for agreeing to take part in the interview today.

Check timing ok

Check consent form filled in / received

Are you happy for the interview to be recorded?

We're interested in your views of the CFHealthHub service and how it fits into the care provided in your centre.

1. Can you describe the key things you did in your centre to help patients adhere to their nebulisers prior to the ACtiF study?

2. How does nebuliser adherence fit with the other things you do for CF patients?

3. What involvement have you had in the CFHealthHub intervention? [Prompts: website, interventionist, training of staff]

4. You had training to help you be more aware of patient activation. What did you think of the training? [Prompts: Do you think it has changed your practice in any way? If yes what changes, if no why not? Key aspects – patient knowledge including beliefs / skills / confidence]

5. Do you think CFHealthHub is a useful intervention? Why? / Why not? [Prompts: what do you think about the: website, feedback about adherence data, interventionist, training?]

6. How do you think the CFHealthHub intervention is operating in practice? [Prompt: what are the strengths / improvements needed?]

7. How does the CFHealthHub intervention fit with the care offered by your centre?

8. How does the CFHealthHub intervention help your patients to know how to use their nebuliser? [Prompts: Skills / knowledge / beliefs. How / Why doesn't it help?]

9. How does the CFHealthHub intervention help your patients to find ways to use their nebuliser more? [Prompts: How / Why doesn't it help?]

For peer review only - http://bmjopen.bmj.com/site/about/guidelines.xhtml

10. How does the CFHealthHub intervention help to motivate your patients to use their nebuliser? [Prompts: How / Why doesn't it help?]

11. Do you think CFHealthHub is helping your intervention patients to improve their adherence? If yes, what key things have helped this? If no, what if anything could be done to help this?

12. Has the CFHealthHub intervention changed anything about the way in which you and/or your team approach adherence in your centre?

i) for patients receiving the intervention?

ii) for patients not receiving the intervention?

[Prompts: MDT discussions / differences between control and intervention patients]

13. Which patient groups are most likely to benefit from CFHealthHub? Why?

14. Which patient groups are least likely to benefit from CFHealthHub? Why?

15. Would you consider continuing to use CFHealthHub in the future? Why? Why not?

16. How has it been for you / your centre taking part in the trial? [Prompt: recruitment to the study]

17. How able do you feel to go on delivering care related to improving adherence after the study ends? [Prompt: has the study changed the way you will go about this?]

18. Are there any aspects of the research that we haven't talked about that you'd like to comment on?

Is there anything else you'd like to say?

THANK YOU

The ACtiF Project is funded by the National Institute for Health Research's Programme Grants for Applied Research.

For peer review only - http://bmjopen.bmj.com/site/about/guidelines.xhtml

Additional File 03 - Quantitative results from process evaluation

Table a. Key dates in process evaluation by participant

Study ID	Interview Date	Baseline date	5 month follow up date	Date of first intervention meeting	Time in the trial at interview (days)	Time since first intervention session at interview (days)	Time in trial at follow up (days)
R02/02	13/09/2016	06/07/2016	10/11/2016	05/08/2016	69	39	127
R02/03	09/09/2016	08/07/2016	NA	05/08/2016	63	35	NA
R02/42	12/10/2016	15/07/2016	21/12/2016	NA	89	NA	159
R02/07	15/11/2016	12/07/2016	12/12/2016	09/09/2016	126	67	153
R02/12	02/11/2016	14/07/2016	03/01/2017	05/10/2016	111	28	173
R02/52	03/02/2017	04/07/2016	22/11/2016	05/10/2016	214	121	141
R01/44	01/12/2016	07/07/2016	16/11/2016	08/11/2016	147	23	132

For peer review only

R01/48	17/01/2017	04/07/2016	15/11/2016	13/10/2016	197	96	134
R01/49	30/01/2017	22/07/2016	07/12/2016	10/10/2016	192	112	138
R01/54	21/03/2017	25/07/2016	13/12/2016	02/11/2016	239	139	141
R01/39	27/02/2017	02/08/2016	21/12/2016	03/11/2016	209	116	141
R01/02	06/12/2016	31/08/2016	25/01/2017	15/08/2016	97	113	147
R01/40	05/12/2016	05/09/2016	17/02/2017	05/10/2016	91	61	165
R01/42	03/10/2016	12/09/2016	15/02/2017	15/08/2016	21	49	156

For peer review only

Table b. Interventionist-generated motivation data (intervention arm) R02/42, R02/49, R02/15 and R01/48 were all missing

Participant ID	Date	Consent Visit Motivation Rating	Was Participant motivation too low Answer Yes/No
R02/39	05.08.16	7	No
R02/40	23.08.16	4	No
R02/02	05.08.16	7	No
R02/03	03.08.16	1	No
R02/43	12.08.16	7	No
R02/05	22.08.16	5	No
R02/45	18.08.16	7	No
R02/07	09.08.16	7	No
R02/48	05.10.16	7	No
R02/10	14.09.16	7	No
R02/11	28.09.16	7	No
R02/50	26.09.16	7	No
R02/12	05.10.16	7	No
R02/52	03.10.16	7	No
R01/39	03.11.16	7	Page missing from report
R01/02	16.09.16	7	No
R01/03	03.10.16	5	No
R01/40	05.10.16	7	Page missing from report
R01/42	15.08.16	5	Page missing from report
R01/44	08.11.16	7	Page missing from report
R01/47	10.10.16	5	Yes
R01/06	10.10.16	7	Page missing from report
R01/49	17.10.16	7	No
R01/08	01.11.16	7	Page missing from report
R01/50	Missing report		
R01/53	29.11.16	7	Not ticked
R01/54	Missing report		
R01/10	10.11.16	2	Not ticked
R01/57	31.10.16	0	Yes

For peer review only

Table c. Engagement

	Adherence data collected (did not withdraw from data collection before 6m) n(%)	Total CFHH sessions Median (IQR)	Baseline adherence Median (IQR)
**Overall (n=33)**	29(88%)	3(1,8)	20(2.1,47.8)
**Qualitative case studies**			
**High adherence at end**			
R01/39	Yes	1	0
R02/07	Yes	2	96.7
R01/40	Yes	9	43.1
R02/52	Yes	13	96.6
**Moderate adherence at end**			
R01/49	Yes	4	13.2
**Low adherence at end**			
R01/54	Yes	11	44.8
R01/02	Yes	1	30.2
R01/48	Yes	3	1.8
R02/12	Yes	44	10.2
R02/03	No	3	5.4
R01/44	Yes	1	19.5
**Withdrawn**			
R01/42	Yes	41	21.1
R02/02	No	3	92.5
R02/42	No	0	4.2

Note: R02/42, R02/02 withdrew from adherence data collection and from the intervention and R02/03 was lost to follow-up. R01/42 did not withdraw from data collection until the end of the study; they did not contribute sufficient data for the 150-180 day period.

For peer review only

Table d. Activities: all participants

	Self-monitoring adherence	Tailored education about treatment	Tailored patient stories (videos)	Personalised action plan/Personalised goal-setting	Tailored problem-solving		Goal review; Rewards
	Clicks How am I doing?	Clicks Toolkit	Clicks Videos	Clicks Action Plan	Clicks Problem Solving	Clicks Coping Plan	Review sessions with Interventionist
**Mean (SD)†/Median* (IQR) overall (n=33)**	11(5 , 30)*	3(0 , 7)*	2(1 , 3)*	2(1 , 7)*	3(0 , 8)*	1(0 , 3)*	1(0.5)†
**Qualitative case studies**							
**High adherence at end**							
R01/39	8	3	1	1	0	1	1
R02/07	5	1	1	1	2	0	1
R01/40	52	0	1	1	3	0	1
R02/52	70	5	3	1	17	1	1
**Medium adherence at end**							
R01/49	30	2	1	0	1	0	1
**Low adherence at end**							
R01/54	24	4	5	3	4	2	1
R01/02	3	0	1	2	0	1	2
R01/48	38	6	2	7	7	1	1
R02/12	98	12	10	13	14	8	1
R02/03	15	12	1	25	1	14	1
R01/44	11	0	2	4	8	3	1
**Withdrawn**							
R01/42	69	18	9	16	20	3	2
R02/02	3	7	1	8	8	7	1
R02/42	0	0	0	0	0	0	0

For peer review only

Table e. Activities: highly motivated participants

(Those who answered 'No' to question, 'Was the participant motivation too low) n=17. Some of these were missing or not answered n=14, only 2 answered 'Yes'.

	Self-monitoring adherence	Tailored education about treatment	Tailored patient stories (videos)	Personalised action plan/Personalised goal-setting	Tailored problem-solving		Goal review; Rewards
	Clicks How am I doing?	Clicks Toolkit	Clicks Videos	Clicks Action Plan	Clicks Problem Solving	Clicks Coping Plan	Review sessions with Interventionist
**High motivation Mean (SD)†/Median* (IQR) overall (n=17)**	16 (5 33)*	5 (2 ,12)*	3 (1 , 4)*	4 (2 , 12)*	4(2 , 11)*	1(1 , 7)*	1.12(0.33)†
**Qualitative case studies (high motivation)**							
**R02/07**	5	1	1	1	2	0	1
**R02/52**	70	5	3	1	17	1	1
**R01/49**	30	2	1	0	1	0	1
**R01/02**	3	0	1	2	0	1	2
**R02/12**	98	12	10	13	14	8	1
**R02/03**	15	12	1	25	1	14	1
**R02/02**	3	7	1	8	8	7	1

For peer review only

Table f. Process Outcomes

	Accurate awareness of adherence	Increased Motivation	Increased necessity and decreased concern / beliefs Motivation		Increased self-efficacy /	Motivation	Increased habit /	Reduced CHAOS	Reduced barriers
	Subjective adherence (0-100): Medication Adherence Data Questionnaire	Change in BMQ question 'I want to do all my prescribed medications in the next 2 weeks (0-7)	Change in BMQ Necessities score (2-5)	Change in BMQ Concerns score (1-3)	Change in BMQ question 'I am confident I can do all my prescribed medications in the next 2 weeks (0-7)	Change in PAM activation score (0-100)	Change in SRBAI score (0-28)	Change in CHAOS score (0-24)	Change in no. of BMQ barriers ticked (0-6)
**n Overall**	30	31	31	31	31	31	31	31	31
**Mean (SD) overall**	2.07(27.87)	-0.1(1.27)	0.26(0.58)	-0.19(0.31)	0.06(1.79)	- 2.38(14.01)	0.32(3.92)	0.1(2.75)	-1.84(3.44)
**Qualitative case studies**									
	baseline(change) %	**baseline (change)**			**baseline (change)**				
**High adherence at end**									
R01/39	85(14)	7(0)	0.5	-0.4	7(0)	-5.9	-2	2	-4
R02/07	100(-2)	7(0)	0.2	-0.2	7(0)	0	1	-5	-3
R01/40	92(8)	7(0)	0.6	-0.2	5(1)	7.2	-9	0	1
R02/52	95(-25)	7(0)	0.3	-0.2	7(0)	4.9	3	-1	1

For peer review only

R01/49	100(0)	7(0)	-0.8	-0.7	7(0)	9.9	-1	0	-4
**Low adherence at end**									
R01/54	60(-10)	7(-1)	-0.3	0.4	6(0)	-7.9	1	-1	6
R01/02	55(16)	7(0)	0.8	-0.2	2(3)	0	-1	-1	-2
R01/48	0(100)	7(0)	0.9	-0.8	6(0)	0	0	0	-2
R02/12	NA	7(0)	-0.1	-0.7	4(0)	14.6	-3	-2	-5
R02/03	50(NA)	1(NA)	NA	NA	2(NA)	NA	NA	NA	NA
R01/44	0(0)	7(0)	1.4	0.2	5(-4)	-16.6	0	-1	-5
**Withdrawn**									
R01/42	0(0)	5(-1)	-0.3	-0.1	4(0)	-5	-1	5	-6
R02/02	80(10)	7(0)	0.1	-0.5	7(0)	9.2	2	-1	1
R02/42	100(0)	7(0)	0.9	0	7(0)	-12.1	1	7	-1

For peer review only

Table g. Intermediate Outcomes

	End of trial adherence (day 150-180)✦	Change in Objective adherence✦ (%)	Change in FEV1	Number of exacerbations in 6 months
**Mean (SD)⁺/ Median (IQR)* overall (n=33)**	34.7 (0.4 ,78)*	1.25(-5.8 , 36.3)*	0.1(0.51)⁺	1(0 , 2)*
**Qualitative case studies**				
**High adherence at end**				
**R01/39**	95.2	95.16	-0.02	1
**R02/07**	93.5	-3.12	NA	0
**R01/40**	88.2	45.07	0.22	0
**R02/52**	83.9	-12.68	-0.13	0
**Medium adherence at end**				
**R01/49**	68.3	55.06	-0.12	3
**Low adherence at end**				
**R01/54**	29	-15.8	-0.03	2
**R01/02**	29	-1.14	0	0
**R01/48**	5.2	3.34	1.07	0
**R02/12**	0	-10.23	-0.21	0
**R02/03**	0	-5.42	NA	NA
**R01/44**	0	-19.54	0.9	1
**Withdrawn**				
**R01/42**	NA	NA	0	0
**R02/02**	NA	NA	-0.04	3
**R02/42**	NA	NA	0.35	1

✦Normative numerator adjusted adherence

For peer review only

Additional File 04: Joint display table (data sources in **bold**)

#	Logic model column / construct	Quantitative	Qualitative	Convergence code
	**INPUTS**			
1	MDT introduction to CFHealthHub	-	**Chief investigator reported:** introducing MDT to concept behind and application of CFHH.	-
2	CF Clinicians aware of the importance of monitoring adherence	-	**Chief investigator reported:** briefing collaborating MDTs. Reported change agents at centres internalised idea; some residual scepticism among senior physicians.	-
3	Prescription data	Study team found adherence levels of over 100% **(Implementation log, 01 Dec 16)**	Late identification of prescription changes found to be responsible. **(Minutes, Trial Management Group Meeting 10 Jan 17)**	Expansion
4	Chipped nebuliser	-	Devices ordered centrally by CTRU were delivered to sites on 20th May 2016 and processed for distribution on 23rd June 2016. **(Project manager emails)**	-
5	Qualcom-Hub (docking & upload)	-	Devices ordered centrally by CTRU were delivered to sites on 20th May2016 and processed for distribution on 23rd June 2016 **(Project manager emails)**	-
6	CFHealthHub website/app	-	Available, but under development through trial **(Additional File 01)**	-
7	COM-BMQ questionnaire	COM-BMQ questionnaire data was collected at baseline for all consenting participants	-	-

For peer review only

	responses	**(Additional File 04 - Table 8)**		
8	Intervention manual	-	High levels of interventionist satisfaction with manual. R01 Interventionist 1 remarked that, "all the stuff in the manuals was really good."	-
9	Interventionist training programme	**Structured questionnaire on interventionist confidence after training programme:** Interventionists (n=5) all averaged >8 for confidence across 11 questions. Isolated scores of <8 occurred three times: viewing charts/tables, completing report forms and understanding online training/assessment.	**In interviews**, interventionists reported high levels of satisfaction; one requested for more integration of research and intervention procedures. R01 Interventionist 1 remarked "You had the manual but I was missing bits". She wanted more case studies and mock patients in the training to compensate for this. An interventionist (R01 MDT member 1), who was a social worker by background, found the training very good, indicating that it the training had acceptability beyond physiotherapists.	Expansion
10	Interventionist support	-	**Research team member (MH) reported** giving mentorship and that one site/trust received more support from the PI than the other. The main interventionist at the other site received support from the part-time interventionist who was a member of the multi-disciplinary team.	-
11	Competency/Fidelity assessment	**Structured instrument for the assessment of interventionist competence:** Digital recordings were made and assessed for fidelity by MA, MH and JB. Fidelity assessment instrument modified after discussion, in advance of use on full-scale RCT.	-	-

For peer review only

12	Motivated and effective interventionists	-	**In interviews**, interventionists reported that they were enthusiastic about the intervention	-
	**ENGAGEMENT**			
13	Clinicians accessing adherence data*	Clinicians did not access CFHH. **(CFHH Click analytics)**	**In interviews**, interventionists talked about run charts occasionally being viewed when brought to MDT meetings by interventionists.	Confirmation
14	Adherence data tracking	**CFHH click analytics** showed interventionists accessing data before meetings	This was confirmed **in interviews**.	Confirmation
15	Participant accessing CFHealthHub	**Click analytics:** The median number of sessions over 5 (+/- 1) months was 3 (interquartile range 1 to 8, range 1-44, **Additional File 05 - Table c**), with a mean duration of 36.1 (SD=23.9) minutes. The mean total duration of interaction time across the study was 49.3 (SD 44.8) minutes. The mean length of an interaction was 12.4 (SD=9.6) minutes. The median number of days in the trial with interactions was 2 (IQR=1,7).	Lack of usability was explained **in interviews** by initially difficult login procedures and the lack of a mobile app for most of the pilot trial, leading participants to access an unsatisfactory desktop version on their mobile.	Expansion
16	Push notifications/reminders each week*	-	**Programmer reported that** automated push notifications not available during pilot trial. **In interviews**, one participant and one interventionist, reported the spontaneous development of informal push notifications in which the interventionist was ringing up and praising the participant for accomplishments, thereby building the relationship.	-
17	CFHealthHub Intervention sessions delivered according	**Collected via project-specific structured fidelity assessment instrument (#11).** After discussion	**Fidelity observations indicated:** limited discussion of motivations; communication style	Expansion

For peer review only - http://bmjopen.bmj.com/site/about/guidelines.xhtml

	to Manual (Fidelity)	between MA, MH and JB summary scores were agreed for delivery of content 100% and quality of delivery: 60-92%. Co-author Judy Bradley is intending to publish this work elsewhere.	sometimes paternalistic rather than autonomy-enabling; insufficient attention to most active ingredients.	
18	Initial session, and then review at each clinic visit	**Collected via click analytics.** Patient run charts reveal a disparity in when and whether these happened **(Additional File 07)**.	-	-
	**ACTIVITIES**			
19	Clinicians monitor adherence	-	Clinician access to adherence data was sporadic (see #13) and **staff interviews confirmed** that it was not monitored. **In an interview**, participant R01/02 described the research intervention as "parallel rather than integrated" with mainstream clinical management.	-
	Intervention components for all participants			
20	Self-monitoring adherence	**Click analytics:** 'How am I doing?' pages were the most frequently visited in terms of the total number of clicks during the trial. 30 (90.9%) participants clicked a median of 11 (range 5-30) times in 5 months, but sometimes in a single session **(Additional File 05 – Table d)**. Access did not always result in good alignment between subjective and objective adherence **(Additional File 05 – Tables f and g respectively)**.	**In interviews**, moderate and frequent users said they mostly valued this page for self-monitoring.	Expansion
21	Tailored education about treatment	**Click analytics:** Toolkit clicked a median 3 (range 0-7) times **(Additional File 05 – Table d)**.	**In participant interviews**, the DNASE video was popular. Other pages were accessed	Expansion

For peer review only

			infrequently or when issues arose, when the information was viewed as "more down to earth" (R02/07) than technical manuals.	
22	Tailored patient stories (videos)	**Click analytics:** 'Talking heads' videos accessed a median 2 (range 1-3) times **(Additional File 05 – Table d)**.	**In participant interviews**, these videos divided opinion. Some participants liked to know that they were not alone; others did not want to see videos of others with CF.	Expansion
	Intervention components for those with adequate motivation			
23	Personalised goal-setting	**Click analytics:** Participants set target adherence levels in CFHH **(Additional File 05 – Table 3)**.	**In interviews**, participants reported goal-setting, but it was not clear how much it came from patients and how much from interventionists.	Expansion
24	Goal review	**Click analytics:** Mean (SD) review sessions 1 (0.5) **(Additional File 05 – Table e)**.	-	-
25	Personalised action plan	**Click analytics:** Action plan pages clicked on median 2 (inter-quartile range 1-7) times **(Additional File 05 – Table e)**.	Disliked by some participants who, the interventionist from centre R01 **reported during an interview**, found writing down action plans like "being at school"	Expansion
26	Tailored problem-solving	**Click analytics:** Problem solving and coping plan pages clicked on median 3 (inter-quartile range 0-8) and 1 (0-3) times respectively **(Additional File 05 – Table e)**.	**In interviews**, one participant realised that when she goes to her friend's house, rather than missing a treatment she could do it in the car or anywhere. One interventionist from centre R02 thought it important that the information was	Expansion

			"there if you need it" for patients.	
	**IMMEDIATE OUTCOMES**			
27	Medical care informed by adherence	-	**Interviews** with PIs found that the trial and intervention ran alongside usual care rather than being informed by it (see also #13, #19).	-
	For all participants			
28	Acute awareness of adherence / increased Motivation	Answers to the **subjective adherence question (Additional File 05 – Table f)** were well aligned with **run charts (Additional File 07)** in those with high adherence. Alignment was more variable in those with moderate and poor adherence.	**In interviews**, some with high adherence used the CFHH "How am I doing page" (run charts) as a check (R02/07, R01/40); other high adherers did not (R01/49). Some felt that it increased their adherence, acknowledging that monitoring meant that they had, "…better make an effort here".	Expansion
29	Increased necessity and decreased concern	No change in the group averages for the **COM-BMQ** (incorporating Beliefs about Medicines Questionnaire - specific (Nebuliser adherence) 21-item validated self-report tool[1]) or Patient Activation Measure (**PAM-13**) (Health Style Assessment) assessment of patient knowledge, skill, and confidence for self-management[2]. **(Additional File 05 – Table f)**	-	-
30	Increased self-efficacy / Motivation	No change in the group averages for a single question about confidence to adhere or the PAM-13. **(Additional File 05 – Table f)**	-	-
	For those with adequate			

For peer review only

	motivation			
31	Increased self-efficacy/ Motivation	No change in the group averages for a single question about confidence to adhere or the **PAM-13**. **(Additional File 05 – Table f)**	-	-
32	Increased habit / Reduced CHAOS	No change in the group averages for **Self-Report Behavioural Automaticity Index (SRBAI)** automaticity-specific subscale of the Self Report Habit index to capture habit-based behaviour patterns[3] or in the assessment of routine measure of **life chaos**[4]. **(Additional File 05 – Table f)**	-	-
33	Reduced barriers	No change in the group averages for The Beliefs about Medicines Questionnaire - specific (Nebuliser adherence) (**BMQ 21-item**) **(Additional File 05 – Table f)**	The tailored problem-solving modules (#26) were not widely used but, **in interviews**, party plans and nebuliser guides were cited as having removed barriers by those who did use this content. For instance, one participant was able to find the technical name for a part of a nebuliser for which he needed to order a replacement.	Expansion
	**INTERMEDIATE OUTCOMES**			
34	Treatment optimisation	-	**Interview data** revealed patients to be behaving in unexpected ways, for instance taking holidays from their treatment or not taking medication as prescribed.	-
35	Increased adherence	**Nebuliser data via CFHH:** Mean adherence across all participants was 10 (95% CI: -5.2 to 25.2) percent higher in the intervention than in the	-	-

		control arm. Within the case study participants (all intervention), an increase of 7.5% (95% CI: -8.2-23.1) in simple normative adherence with numerator adjustment can be observed in the intervention arm. Following month 1, adherence is consistently higher in the intervention arm with the greatest difference observed in month 5 (mean difference: 10.8, 95% CI: -11.44, 22.9). These differences would indicate a potentially clinically important difference between the intervention and usual care arms.		

[1] R. Horne, J. Weinman, M. Hankins, The beliefs about medicines questionnaire: The development and evaluation of a new method for assessing the cognitive representation of medication, Psychol. Health. 14 (1999) 1–24. doi:10.1080/08870449908407311.

[2] J.H. Hibbard, E.R. Mahoney, J. Stockard, M. Tusler, Development and testing of a short form of the patient activation measure., Health Serv. Res. 40 (2005) 1918–30. doi:10.1111/j.1475-6773.2005.00438.x.

[3] B. Verplanken, S. Orbell, Reflections on Past Behavior: A Self-Report Index of Habit Strength1, J. Appl. Soc. Psychol. 33 (2003) 1313–1330. doi:10.1111/j.1559-1816.2003.tb01951.x.

[4] M.D. Wong, C. a. Sarkisian, C. Davis, J. Kinsler, W.E. Cunningham, The Association Between Life Chaos, Health Care Use, and Health Status Among HIV-Infected Persons, J. Gen. Intern. Med. 22 (2007) 1286–1291. doi:10.1007/s11606-007-0265-6.

For peer review only

Additional File 05 - Case-ordered descriptive matrix for fourteen case studies

Qualitative findings in italics. Otherwise, motivation, confidence, necessities, concerns, life chaos and subjective adherence (baselines and process outcomes) from self-report instruments (see Methods and Additional File 04). Engagement, activities and data captured by CFHealthHub.

Case / baselines / context	Engagement	Activities	Process outcomes	Intermediate outcomes
**High adherence (average >80%) in last month of trial**				
**R01/39.** High motivation, confidence and necessities, medium concerns, quite high chaos. *They got a lot of information about CF from other websites.*	Used CFHH once. *Very engaged with interventionist and trial.*	*Didn't make plans – felt it was her responsibility to adapt her life; found others monitoring helpful. Didn't like videos or social aspects of website because of the reminder of her mortality.*	*Knowledge that clinicians could access treatment adherence information provided extra motivation to adhere.*	End of trial adherence 95% (95% improvement).
**R02/07.** High motivation, and confidence, medium-high necessity, medium concerns and chaos. Existing high adherer, *sees treatment as a "plan for longevity" rather than a "chore".*	Used CFHH twice. *Didn't find it useful or like the videos (doesn't want to see negative side of CF).*	Made action plan, accessed some modules once. *Found goal-setting with interventionist helpful.*	Little change as already, motivated. Reduced CHAOS and barriers.	End of trial adherence 93% (3% decline).
**R01/40.** High motivation, medium confidence and necessities, low concerns, medium-to-low chaos. *Was recruited soon after exacerbation.*	Had nine CFHH sessions. *"I've been logging on to track my progress... every two weeks to a month". Finds others monitoring him helpful.*	Frequent self-monitoring. *Compensates for slippages by planning to do the rest of his doses.*	Motivation already high, but habit lacking. *Intervention has made him think about adherence more than he did before.*	End of trial adherence 88% (45% improvement). Variance over trial, but trajectory.
**R02/52.** High motivation, confidence and necessity, low concerns, low-medium chaos. Existing good adherer; *wanted something like a fitness tracker with feedback - messages on performance.*	13 CFHH sessions. *Liked the more portable nebuliser, could take it away on work.* CFHH session that precedes interventionist visit explained by interventionist testing login details.	Frequent self-monitoring, regular use of tailored education and problem solving (fixing nebuliser problems) and some use of videos. *Wanted it expanding to physical activity.*	Motivation already high. Increased habit.	End of trial adherence 83% (12% decline).

For peer review only - http://bmjopen.bmj.com/site/about/guidelines.xhtml

Case / baselines / context	Engagement	Activities	Process outcomes	Intermediate outcomes
**Moderate adherence (average 50-80%) in last month of trial**				
**R01/49.** High motivation, confidence, medium-high necessity and concerns low chaos. *Participated to 'prove' themselves to their physiotherapist*; poor awareness of own adherence not improved over course of trial.	4 CFHH sessions	Used problem-solving modules and self-monitoring, but no action plan.	Increased motivation, reduced barriers.	End of trial adherence 68% (55% improvement). An important improvement from low adherence, but subjective adherence still poorly 'calibrated' with objective adherence.
**Poor adherence (>50%) in last month of trial**				
**R01/54.** Professed high motivation and confidence, medium necessity, low concerns, medium to low chaos. *Wants the doctor "to notice" that they are adherent to their treatment, demotivated by the fact they don't.*	44 CFHH sessions. *Appreciative of extrinsic motivation from face-to-face contact with interventionist.*	Frequent self-monitoring; initially high use of action plans and problem solving. *Dislikes 'talking heads' videos.*	More barriers by the end of the trial.	End of trial adherence 29% (16% decline), but run chart shows huge variance week by week.
**R01/02.** High motivation, low confidence, medium necessity and concerns, high chaos. *Dissatisfaction at service reconfiguration: moved across from Poole to Southampton during trial. Upset that wider team isn't noticing their adherence.*	Used CFHH once *but had technical problems. Appreciative of interventionist: "Having a personal contact and someone to guide you through it is really useful" Wider team not talking about adherence: "parallel rather than integrated".*	Two review sessions with interventionist.	Reduced CHAOS and barriers; increased self-efficacy	Lack of pre-post change not contradicted by the run chart which shows improvement.

For peer review only - http://bmjopen.bmj.com/site/about/guidelines.xhtml

Case / baselines / context	Engagement	Activities	Process outcomes	Intermediate outcomes
**R01/48.** professed high motivation and confidence, medium-high necessities and concerns; medium chaos. *This 69-year old doesn't like nebulising; "can't teach an old dog new tricks". No belief in benefit of nebulised medication. Poor awareness of own adherence. Altruistic trial participant.*	Used CFHH three times. *Access problems (passwords, etc) - gave up.*	Some engagement with toolkit, action plans and problem-solving, *didn't like the videos.* Engagement drops off as soon as the last meeting over.	No change in process outcomes.	End of trial adherence 5% (3% improvement). *Said was making an effort for the trial.* In line with this, objective adherence was high (~80%) for weeks 6-21
**R02/12.** High motivation, medium to low confidence, medium to high necessity and concerns, medium chaos.	Started off engaged, lots of CFHH use and two intervention sessions in first 100 days, nothing thereafter.	Made plans, liked website, checked graphs. *Liked face-to-face interaction with interventionist.*	Decreased chaos and barriers but also decreased habit.	Initial improvement in adherence (up to 100% between weeks seven and nine after first intervention not sustained over time. Review stimulates brief improvement at week 15, again not sustained.

For peer review only - http://bmjopen.bmj.com/site/about/guidelines.xhtml

Case / baselines / context	Engagement	Activities	Process outcomes	Intermediate outcomes
**R02/03.** Low motivation and confidence, medium necessities, concerns and chaos. *Treatment is something that he has to do but doesn't want to do it, or think about CF. Forgets about treatment because of busy lifestyle. Prioritises other things above health. Knows that this doesn't end well, but no readiness to change.*	Minimal short-term engagement with CFHH. *Interventionist notes that participant has always been difficult to get hold of.*	Made action and coping plans, checked graphs.	No process data at follow-up.	Withdrew from treatment early.
**R01/44.** High motivation, medium confidence, necessities, low concerns, high chaos *("I can't seem to get into a routine"). Recruited during exacerbation:* baseline artificially high. Intervention 1 visit didn't happen until Week 17. *Participant describes self as "uncompliant" except around inpatient stays.*	One CFHH session (at intervention visit 1). Interventionist appears not to have done correct preparation. Only participant rated by an interventionist as having inadequate motivation.	*Participant confirms that he made action plan, coping plan and checked graphs with interventionist but chaotic lifestyle and low motivation prevented further use. Admits only has a routine in hospital.*	No change in process variables.	Initial spikes of adherence not sustained over time.

For peer review only - http://bmjopen.bmj.com/site/about/guidelines.xhtml

**Withdrawn**				
**Case / baselines / context**	**Engagement**	**Activities**	**Process outcomes**	**Intermediate outcomes**
**R01/42.** Medium motivation, low confidence, medium-high necessity, medium concerns, low chaos. *Originally an i-neb user. Does not think nebulising three times a day is achievable. Moved house during study. No broadband – so didn't do nebulisations.*	*Loved the website and shared it.* 41 CFHH sessions. *Intervention visit 1 reported to be chaotic.*	Made action plan.	Little change in process variables.	Interview might have triggered brief increase in nebuliser use, when *participant realised nebulisations were being logged even when he wasn't plugging it in.*
**R02/02.** High motivation and confidence, medium-high necessity low concerns and chaos. *Interview shows them to be motivated by interventionist visit and qualitative interview (Hawthorne effect).* Subjective adherence poorly aligned to objective adherence.	Limited engagement. Three CFHH Sessions all on the same day.	Made an action plan *but reported that she didn't set goals because she thought she her adherence was already good.*	Little change in process variables.	Adherence run chart starts off high, but drops off quickly. Interview might have triggered brief increase in nebuliser use. Withdrew from collection of nebuliser data collection.
**R02/42.** High motivation and confidence, medium to high necessity, low concerns, medium chaos	Withdrew - *didn't like the eTrac nebuliser - delivering the drug too quickly made them cough. Interventionist encouraged discontinuation.*	*Didn't look at the website.*	No change in process outcomes	Assumed no change in adherence, but objective lacking.

ACtiF Pilot Statistical Report

L Mandefield

Methods

Outcomes

Feasibility outcomes

The primary objective of this study was to determine the feasibility of proceeding to a definitive trial. An external pilot randomised controlled trial to determine feasibility of a randomised controlled trial based on objective stop-go criteria related to:

(a) participant recruitment;

(b) participant retention; and,

(c) quality of primary outcome data at 5 (+/- 1) months post randomisation.

These were assessed by

i. The number of screened, eligible and recruited participants per month, per centre and overall;

ii. The number and percentage of participants who complete their 5(+/-1) month post randomisation follow up;

iii. The number of Fuchs criteria by exacerbation.

Clinical outcomes

The primary clinical outcome measure was the number of pulmonary exacerbations in the 5 (+/-1) month post-baseline follow-up period, defined according to a modified version of the Fuchs criteria. The original Fuchs criteria was 4 out of 16 symptoms leading to IV antibiotic treatment. An exacerbation of respiratory symptoms will be said to have occurred when a participant was treated with parenteral antibiotics for any one of the following 12 signs or symptoms:

1. change in sputum;
2. new or increased hemoptysis;
3. increased cough;
4. increased dyspnea;
5. malaise, fatigue, or lethargy;
6. temperature above 38 °C;
7. anorexia or weight loss;

8. sinus pain or tenderness;

9. change in sinus discharge.

10. change in physical examination of the chest, derived from notes by site staff.

11. decrease in pulmonary function by 10 percent or more from a previously recorded value, derived from notes by site staff; or,

12. radiographic changes indicative of pulmonary infection, derived from notes by site staff.

The trial interventionist or prescribing clinician/nurse will collect data on the "exacerbations" form at the point of a participant starting a course of IV antibiotics.

The following secondary outcomes were also collected at baseline and 5 (+/-1) month follow up:

1. Body Mass Index (BMI).
2. Forced expiratory volume in 1 second (FEV1): standardised spirometry as a measure of condition severity.
3. EuroQol EQ-5D-5L: generic health status measure for health economic analysis.
4. The Patient Activation Measure (PAM-13): assessment of patient knowledge, skill, and confidence for self-management.
5. Confusion, Hubbub, and Order Scale (CHAOS 6-item): measure of life chaos.
6. Medication Adherence Data-3 items (MAD-3)
7. Self-Report Behavioural Automaticity Index (SRBAI)
8. Cystic Fibrosis Questionnaire-Revised (CFQ-R): disease specific health-related quality of life instrument.
9. The Patient Health Questionnaire depression scale (PHQ-8): severity measure for depressive disorders.
10. The General Anxiety Disorder 7-item anxiety scale (GAD-7): severity measure for anxiety.
11. The Capability Opportunity Motivation Behaviour Beliefs Questionnaire (COM- BMQ): This questionnaire incorporates:

a. The Beliefs about Medicines Questionnaire - specific (Nebuliser adherence) (BMQ 21-item): a validated self-report tool, customised by the author to identify perceived necessities and concerns for nebuliser treatment.

b. The following project-specific items: one additional belief item, one intention item, one confidence item, and a list of barriers. These will serve as a tailoring tool for the intervention and also as a secondary outcome measure. 12.Subjective adherence single question: self-report estimate of adherence as a percentage. Self-reported problems: identification of capability and opportunity barriers to nebuliser adherence

13. Concomitant medications: bespoke instrument, designed for this research project.
14. Resource use form: interventionist collects data from a combination of hospital notes and the NHS patient electronic system to determine 1) inpatient IV days 2) Routine clinic visits 3) Unscheduled outpatient contacts 3) unscheduled inpatient stays.
15. Prescription: a monthly prescription check to both check for data transfer to CFHealthHub and review for an indication that the prescription has changed or indication of microorganism e.g.
16. Adherence to prescribed medication
17. Any treatment with IV antibiotics

For peer review only

Sample Size

Sample size calculation was based on estimating parameters within a certain amount of precision rather than hypothesis testing. The sample size for a feasibility study should be adequate to estimate the uncertain critical parameters (standard deviations for continuous outcomes; consent rates, event rates, attrition rates for binary outcomes) needed to inform the design of the full RCT with sufficient precision.

To assess recruitment rate, the external pilot RCT ran in two CF units for 12 months, with four months recruitment, one months 'run-in' period (the period between the consent and baseline visit), and 5 (+/-1) months follow up. To match the proposed recruitment rate of the main RCT, the target sample over the four months for which the pilot RCT was open, was 32 per centre (64 in total from the two pilot centres). We aimed to see a minimum of 75% of the recruitment target to be confident of the trial viability i.e. at least 48 patients in total consented and randomized in four months' of recruitment from two centres.

Randomisation

Randomisation was conducted using a computer generated pseudo-random list with random permuted blocks of varying sizes, created and hosted by the Sheffield CTRU in accordance with their Standard Operating Procedures (SOPs) and was held on a secure server. ACtiF participants will be randomised in a 1:1 ratio, intervention to control arms, stratified by:

- Site;
- Number of IV days in previous 12 months as collected at consent visit (two categories will be (i) less than or equal to 14 days and (ii) greater than 14 days).

Study researchers accessed the allocation for each participant by logging in to the remote, secure internet-based randomisation system. Once a participant had consented to the study, the researcher logged into the randomisation system and entered basic demographic information. After this information had been entered the allocation for that participant was then revealed to the researcher.

Block randomisation with randomly varying block size of 2, 4 and 6 was used so that the sequence of allocation could not be predicted. The block sizes were determined by the trial statistician and block size was not revealed to any other member of the study team.

Blinding

The trial statisticians remained blind until data freeze, at which point unblinded data was presented to them so checks could be carried out.

Statistical Methods

All statistical analyses were performed in R version 3.3.1.

Analysis Populations

The ITT population includes all participants for whom consent was obtained and who were randomised to treatment, regardless of whether they received the intervention or not. This is the

For peer review only

primary analysis set and endpoints were summarised for the ITT population unless otherwise stated.

Participant Flow

A CONSORT flow diagram was used to display data completeness and patient flow from first contact to final follow up.

The number of participants recruited at each centre each month was presented. The number of participants who withdrew consent from the trial, withdrew from the intervention, withdrew from collection of the primary outcome, withdrew consent from adherence data collection and who were lost to follow up were presented overall, by treatment arm and site. The reasons for attrition, where given, were presented.

Patient reported outcome measures (PROMS)

The following PROMS were completed at baseline and 5 (+/-1) month follow up visit. For detailed methods of how these questionnaires were scored, please see the appendix.

Data completeness

A CONSORT flow diagram was used to display data completeness and patient throughput from first contact to final follow up.

Baseline characteristics

Participants' demographics (age, sex, ethnicity, IMD decile), physical measurements (weight, height, BMI), clinical measurements (FEV1, IV days in last registry year, Pseudomonas status, Adherence in first 2 weeks, Subjective adherence, Medication, Treatment burden) patient reported outcomes (EQ-5D-5L, PAM-13, CHAOS, MAD-3,SRBAI, CFQ-R, GAD-7, COMBMQ, PHQ-8). Imbalance between treatment arms was not tested statistically but were reported descriptively.

Primary effectiveness analysis of clinical outcomes

The primary endpoint of the study is the number of exacerbations in a 5 (+/- 1) month period. Exacerbations were defined as being treated with IV antibiotics and meeting at least 1 Fuchs criteria.

The number of exacerbations by participant were presented. The number and percentage of exacerbations with each Fuchs criteria were presented. The length of IV course was summarised by intervention arm for all exacerbations and for participants experiencing exacerbations.

The primary effectiveness analysis used a negative binomial model and included all exacerbations in a 6 month follow up period. Participants who were not followed for this length were excluded. An adjusted model included IV days in the previous 12 months as a covariate. Although not prespecified, a further sensitivity analysis was carried out. This model included the number of days followed up as an offset. This allowed all consenting participants to be included. An adjusted offset model included IV days in the previous 12 months as a covariate.

For peer review only

Secondary effectiveness analysis of clinical outcomes

Patient reported outcome measures

Secondary outcomes were measured at baseline and 5 (+/-1) months post randomisation. The mean difference between treatment arms was calculated for each of the secondary outcomes, along with 95% confidence intervals using a multiple linear regression model. Adjustment for baseline and site was carried out and both unadjusted and adjusted results were presented.

Adherence to medication

The time of inhalations of medication was recorded via chipped nebulisers. This data along with prescription data was used to calculate a number of different adherence measures. Adherence in people with CF is of key importance. For this reason, it was decided that 7 separate measures of adherence to prescribed medication were to be presented:

1. Total doses;
2. Unadjusted adherence;
3. Simple normative adherence (without numerator adjustment);
4. Sophisticated normative adherence (without numerator adjustment);
5. Simple normative adherence (with numerator adjustment);
6. Sophisticated normative adherence (with numerator adjustment);
7. Subjective single adherence.

Measures 1-6 are calculated daily based on the chipped nebuliser data and the dose prescribed that day. Means can be calculated for set periods, e.g. weekly.

The specific calculations of these adherence measured are described below.

Total doses taken

As a basic, unadjusted measure of adherence, the total number of doses taken for the time period will be calculated.

Unadjusted adherence

Adherence is typically calculated as the dose taken divided by the dose described per day.

Simple normative adherence (without numerator adjustment)

Quality of adherence reporting is dependent on the PWCF being prescribed the appropriate medications. Adjusting the denominator of the adherence calculation controls for treatment rationalisation to try reduce treatment burden, which is an approach often seen in people in CF. The simple normative adherence is calculated as follows:

1. If the participant does not have pseudomonas
- Minimum denominator is set at 1 treatment/day.

For peer review only

2. If the participant has chronic pseudomonas
- Minimum denominator is set at 3 treatments/day
3. The participant has chronic pseudomonas and intermittent inhaled antibiotic regimens
- Minimum denominator is 3 treatments/day during 28 day 'on' period
- Minimum denominator is 1 treatment/day during 28 day 'off' period
4. The participant has intermittent pseudomonas
- Minimum denominator is 3 treatments/day for 1 or 3 months depending on the eradication regime
- Minimum denominator is 1 treatment/day for the rest of the time

In calculating normative adherence an expected minimum prescription based on a patient's health state is needed. Most patients take a dose of a mucolytic, and patients meeting the criteria will take two doses of antibiotics. In adherence calculations, participants had their denominator amended to reflect their prescription. A complication arises in denominator adjustments when the antibiotic prescribed is one that is expected to be used in an alternating fashion (e.g. 28 days use, 28 days off). The antibiotic medications Aztreonam Lysine and Tobramycin are normally prescribed in this way; for patients with prescriptions for these medications with periods of more than 28 days without a prescription for an antibiotic, the denominator was adjusted to add in 2 doses / day. After 28 days of substituted antibiotic use, a 28 'day off' cycle was programmed. This cycle was continued until such time as another antibiotic prescription was present.

Sophisticated normative adherence (without numerator adjustment)

The sophisticated normative adherence is calculated as follows:

1. If someone has 'mild genotype', is pancreatic sufficient and has FEV1 > 90%, without Pseudomonas and used <= 14 days intravenous antibiotics in the past 1 year.
- There is no minimum target. Denominator is determined by the agreed prescription between clinicians and participants.
2. If someone is homozygous for class I-III CFTR mutation OR pancreatic insufficient OR FEV1 <= 90%, but without Pseudomonas and used <= 14 days intravenous antibiotics in the past 1 year. Minimum denominator is set at 1 treatment/day.
3. If the person has chronic pseudomonas AND/OR
- the person used > 14 days intravenous antibiotics in the previous year Minimum denominator is set at 3 treatments/day
4. If the person has chronic pseudomonas AND/OR used > 14 days intravenous antibiotics in the previous year but is on intermittent inhaled antibiotic regimens
- Minimum denominator is 3 treatments/day during 28 day 'on' period
- Minimum denominator is 1 treatment/day during 28 day 'off' period
5. If someone has intermittent pseudomonas but used <= 14 days intravenous antibiotics in the past 1 year

For peer review only

- Minimum denominator is 3 treatments/day for 1 or 3 months depending on the eradication regime
- Minimum denominator is 1 treatment/day (or 0, i.e. no minimum target) depending on their genotype, pancreatic status and FEV1 for the rest of the time.

Numerator adjustment in simple and sophisticated normative case

Numerator adjustment occurs only if a daily adherence measure is greater than 100%, thus the maximum daily adherence is set at 100%.

Subjective single adherence

All participants will be asked to estimate their adherence as a percentage at baseline, clinic visits, 5(+/-1) months and any further visits up to 30th April 2017. These subjective measures were presented separately. The question referred to the previous 2 weeks.

Adherence summaries

The mean and SD was calculated for each month of the trial by treatment arm. Weekly numerator adjusted normative adherence was calculated and a mean by treatment arm was calculated and presented as a line graph for the first 25 weeks from randomisation.

Intervention adherence

The intervention comprised of:

(a) a chipped nebuliser to collect adherence data

(b) access for participants and interventionist to the adherence data summaries

(c) an online platform (CFHealthHub) offering summaries of adherence and tailored modules to be used by the health professional when interacting with the participant and independently by the participant.

A number of metrics were collected from CFHealthHub including the timing and date of clicks and the page/module that was clicked on. Interactions with CFHH were defined as a series of clicks with no greater that 15 minute gaps between clicks. Length of each session was calculated and days with interactions were calculated by participant.

The mean, standard deviation (SD), median and interquartile range (IQR) for the CFHH metrics were calculated and presented by participant. The same summary statistics were also presented for length of all sessions. The timing of CFHH interactions in days from randomisation was plotted by participant. The number of clicks per page category (Home, How am I doing?, Treatment etc) was plotted in a bar chart and also presented in a table by participant and by session.

Date and time of sessions with the interventionist were also recorded. The number of sessions with an interventionist and the length of sessions by participant were summarised in a table.

For peer review only

Clinic visits

The number of clinic visits completed by each participant excluding consent and 5 month follow up was recorded. Summary statistics were presented by treatment arm to assess whether ascertainment bias occurred in the intervention arm.

Safety analysis

The number of Adverse Events (AEs) and Serious Adverse Events (SAEs) was recorded and presented by treatment arm. These events were further categorised by the type of adverse event and whether they were related to the intervention.

Protocol non compliances

The number and type of protocol non compliances were presented descriptively.

Summary of missing data

The number of missing values or scores for each of the primary and secondary outcomes was presented by baseline and 5 (+/-1) months post randomisation and by treatment arm. Furthermore, the number and percentage of missing items was presented for each of these questionnaires.

For peer review only

Results

Participant Flow

Participants were recruited for 4 months across 2 sites. The CONSORT flow diagram (Fig.1) shows the flow of participants through the trial. 32 participants were randomised at each site. 33 participants were randomised to the intervention arm and 31 participants were randomised to usual care. A total of 59 participants completed the 5 (+/- 1) month follow up visit (Intervention = 31, Usual care = 28).

A total of 8 participants discontinued the trial before the follow up visit (Intervention = 4, Usual care = 4). Of these discontinuations, 5 no longer had their adherence data collected and the same 5 participants did not have their primary outcome collected. Of those who did not continue with primary outcome collection, 2 participants died, 1 withdrew consent and 2 were lost to follow up.

Following the 5 (+/-1) month visit, adherence data and primary outcome data was collected. 2 participants withdrew from adherence data collection during this time (Intervention =1, Usual care =1). 59 participants completed primary outcome data collection up to study completion on 30th April 2017 (Intervention = 31, Usual care =28).

For peer review only

Recruitment by centre and month

For peer review only

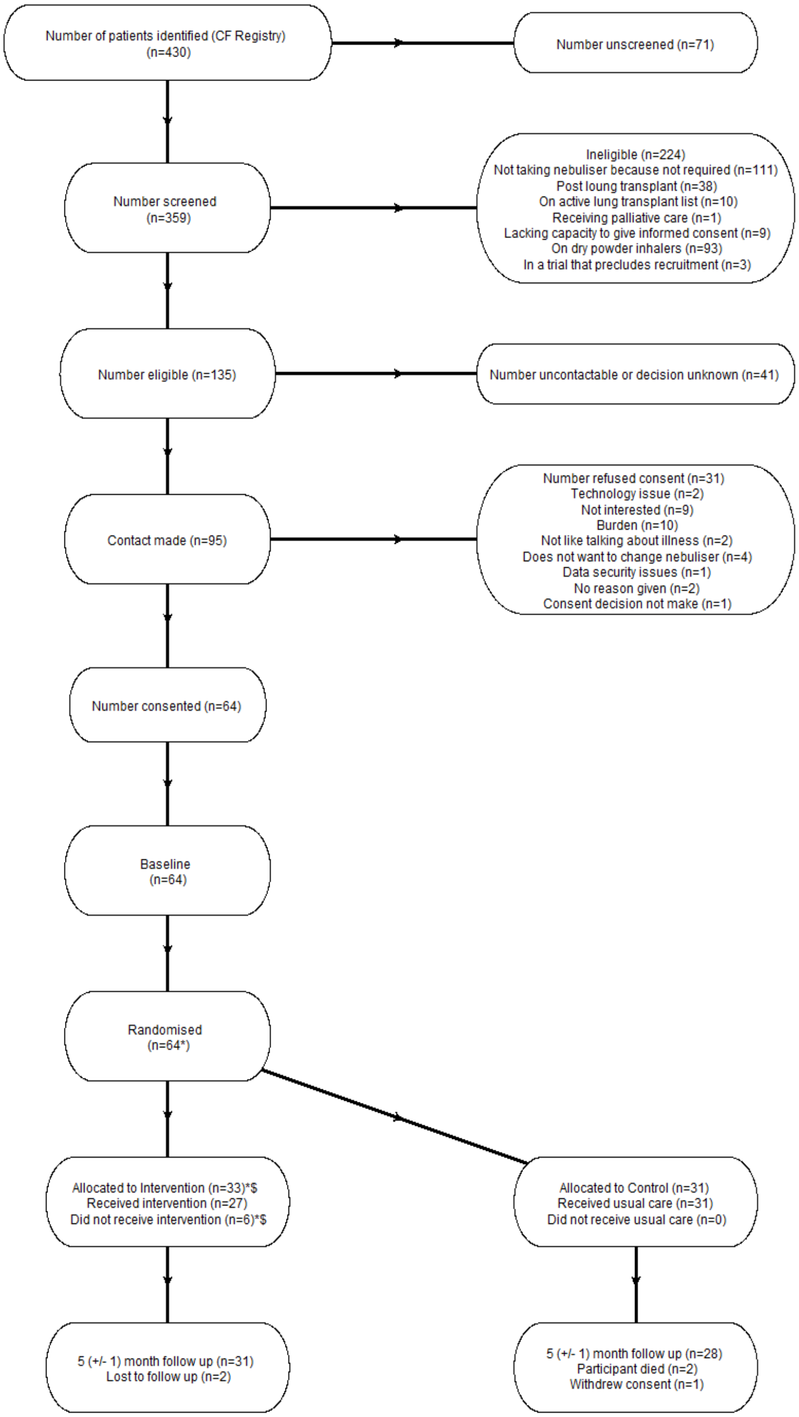

Figure 1: CONSORT flow diagram for ACtiF pilot study.

Table 1: Participants consented by centre and by month

	June 16	July 16	Aug 16	Sept 15	Total
Site A	4	16	7	5	32
Site B	2	17	5	8	32

Attrition by Centre and Treatment arm

Table 2: Attrition presented by treatment arm and site.

		n	Withdrew Consent (%)	Died (%)	Lost to Follow up (%)	Overall (%)
**Overall**		64	1(17%)	2(33%)	2(40%)	5(7.8%)
**Treatment arm**	Intervention	33	0(0%)	0(0%)	2(40%)	2(6.1%)
	Usual Care	31	1(20%)	2(40%)	0(0%)	3(9.7%)
**Site**	Site A	32	0(0%)	1(20%)	1(20%)	2(6.2%)
	Site B	32	1(20%)	1(20%)	1(20%)	3(9.4%)

For peer review only

Baseline characteristics

Table 4 shows the baseline characteristics of participants randomised by treatment arm. 33 participants were randomised to the intervention and 31 were randomised to usual care. The average age of participants was 29.7 (SD=11.5). Participants in the intervention arm were slightly older (median=28, IQR=(21,37)) than those in the usual care arm (median=26, IQR=(20,34)). Table 5 shows the CF measures presented by treatment arm. Tables 6-7 show the baseline questionnaire scores presented by treatment arm.

Baseline demographics

Table 3: Baseline demographics by treatment arm

	Intervention	Control	Overall
Age			
n	33	31	64
Mean(SD)	31.6(13.3)	27.8(8.9)	29.7(11.5)
Median(IQR)	28(21,37)	26(20,34)	27(21,36)
Min,Max	(16,69)	(16,50)	(16,69)
Sex			
Male	18(54.5%)	18(58.1%)	36(56.2%)
Female	15(45.5%)	13(41.9%)	28(43.8%)
Socioeconomic Status			
Most deprived	6(18.2%)	1(3.2%)	7(10.9%)
High deprivation	4(12.1%)	7(22.6%)	11(17.2%)
Average	8(24.2%)	8(25.8%)	16(25%)
Low deprivation	6(18.2%)	9(29%)	15(23.4%)
Least deprived	9(27.3%)	6(19.4%)	15(23.4%)
Weight (KG)			
n	33	31	64
Mean(SD)	65.5(18)	63.7(15.6)	64.6(16.8)
Median(IQR)	63(53,76)	62.9(49,74)	63(52.9,74.3)
Min,Max	(35,128)	(35.6,103.7)	(35,128)
Height (cm)			
n	33	31	64
Mean(SD)	168.6(10.5)	167.7(9.6)	168.2(10)
Median(IQR)	170(162,177)	168(159,175)	168.5(160.5,175.5)
Min,Max	(147,193)	(149,186)	(147,193)
BMI			
n	33	31	64
Mean(SD)	22.8(5)	22.4(4.3)	22.6(4.6)

For peer review only

Median(IQR)	22.2(19.7,25.3)	22.1(19.1,25.4)	22.1(19.55,25.35)
Min,Max	(15.8,42.8)	(16,33.9)	(15.8,42.8)

Table 4: Baseline CF measures by treatment arm

	Intervention	Control	Overall
No. of IV days in previous 12 months			
n	33	31	64
Mean(SD)	26.3(25.7)	26(22.1)	26.2(23.8)
Median(IQR)	17(7,44)	28(0,44)	17(7,44)
Min,Max	(0,117)	(0,70)	(0,117)
No. of participants requiring IV days in previous 12 months			
At least 1 IV day	26(78.8%)	23(74.2%)	49(76.6%)
Days since last IV start date			
n	31	28	59
Mean(SD)	168.7(245.2)	202.3(325.2)	184.6(283.9)
Median(IQR)	75(45,194)	100(24.5,219.5)	91(39,213)
Min,Max	(6,1085)	(7,1575)	(6,1575)
FEV1			
n	33	31	64
Mean(SD)	2(0.8)	2.3(1)	2.1(0.9)
Median(IQR)	1.9(1.4,2.4)	2.1(1.6,2.8)	1.9(1.5,2.7)
Min,Max	(0.8,4)	(0.6,5)	(0.6,5)
FEV1 % Predicted			
n	33	31	64
Mean(SD)	53.4(19.4)	61.4(22.7)	57.3(21.3)
Median(IQR)	49.2(39.4,61.9)	53.4(43,80)	49.6(41.9,76.7)
Min,Max	(26,103)	(23.2,100.7)	(23.2,103)
Clinician pseudomonas status			
Negative	15(45.5%)	8(26.7%)	23(36.5%)
Intermittent	3(9.1%)	3(10%)	6(9.5%)
Chronic	15(45.5%)	19(63.3%)	34(54%)
Leeds Criteria pseudomonas status			
Negative	15(45.5%)	10(33.3%)	25(39.7%)
Intermittent	4(12.1%)	4(13.3%)	8(12.7%)
Chronic	14(42.4%)	16(53.3%)	30(47.6%)

For peer review only

Subjective adherence			
n	23	20	43
Mean(SD)	65.6(40.1)	67.8(35.4)	66.6(37.6)
Median(IQR)	90(20,99)	80(45,99.5)	90(35,99)
Min,Max	(0,100)	(0,100)	(0,100)
Simple normative adherence (first 2 weeks)			
n	33	31	64
Mean(SD)	0.5(0)	0.5(0)	0.5(0)
Median(IQR)	0.5(0.5,0.5)	0.5(0.5,0.5)	0.5(0.5,0.5)
Min,Max	(0.5,0.5)	(0.5,0.5)	(0.5,0.5)
Treatment Burden			
Low	10(30.3%)	11(35.5%)	21(32.8%)
Medium	16(48.5%)	12(38.7%)	28(43.8%)
High	2(6.1%)	5(16.1%)	7(10.9%)

Baseline outcome measures

Table 5: Baseline outcome measures by treatment arm

	Intervention	Control	Overall
EQ5D-5L			
n	33	31	64
Mean(SD)	0.866(0.121)	0.822(0.151)	0.845(0.137)
Median(IQR)	0.901(0.767,0.951)	0.825(0.737,0.942)	0.872(0.752,0.946)
Min,Max	(0.53,1)	(0.486,1)	(0.486,1)
PAM-13			
n	33	31	64
Mean(SD)	60.4(11.2)	60(13.2)	60.2(12.1)
Median(IQR)	60.6(53.2,67.8)	58.1(48.9,67.8)	60.6(51,67.8)
Min,Max	(36.8,84.8)	(38.1,90.7)	(36.8,90.7)
CHAOS			
n	33	31	64
Mean(SD)	9.8(3.4)	10.1(4)	10(3.7)
Median(IQR)	10(8,11)	10(7,12)	10(8,11)
Min,Max	(4,18)	(4,20)	(4,20)
MAD-3			
n	32	30	62
Mean(SD)	9.8(3.3)	9(3.4)	9.4(3.4)

For peer review only

Median(IQR)	9(8,12.5)	9.5(6,11)	9(8,12)
Min,Max	(3,15)	(3,15)	(3,15)
SRBAI			
n	33	30	63
Mean(SD)	11.5(4.9)	10.2(5.6)	10.9(5.2)
Median(IQR)	12(8,16)	9(4,14)	10(7,15)
Min,Max	(4,20)	(4,20)	(4,20)
GAD-7			
n	33	31	64
Mean(SD)	4.1(4.5)	3.8(3.6)	3.9(4)
Median(IQR)	3(0,5)	3(1,7)	3(0.5,5.5)
Min,Max	(0,15)	(0,11)	(0,15)
PHQ-8			
n	33	31	64
Mean(SD)	7(4.9)	6.5(5.2)	6.8(5)
Median(IQR)	6(3,12)	6(3,8)	6(3,10.5)
Min,Max	(0,16)	(0,18)	(0,18)

Table 6: Baseline CFQR domains by treatment arm

	Intervention	Control	Overall
Physical Functioning			
n	33	31	64
Mean(SD)	48.5(34.8)	49.2(30.8)	48.9(32.7)
Median(IQR)	38(25,88)	42(17,83)	42(21,85.5)
Min,Max	(0,100)	(0,100)	(0,100)
Emotional Functioning			
n	33	31	64
Mean(SD)	70.2(21.1)	62.3(26.1)	66.4(23.8)
Median(IQR)	67(53,93)	67(40,80)	67(53,87)
Min,Max	(27,100)	(7,100)	(7,100)
Eating			
n	33	31	64
Mean(SD)	79.9(24.8)	74.6(27.7)	77.3(26.2)
Median(IQR)	89(67,100)	78(56,100)	89(61.5,100)
Min,Max	(0,100)	(0,100)	(0,100)
Social Functioning			
n	33	31	64

For peer review only

Mean(SD)	65(20.3)	59.6(26.2)	62.4(23.3)
Median(IQR)	67(50,78)	61(44,83)	67(44,83)
Min,Max	(17,100)	(11,100)	(11,100)
Body Image			
n	33	31	64
Mean(SD)	68.5(27.3)	64.9(31.7)	66.7(29.3)
Median(IQR)	78(56,89)	67(44,100)	78(44,89)
Min,Max	(0,100)	(0,100)	(0,100)
Treatment Burden			
n	33	31	64
Mean(SD)	50.5(16.5)	51.6(25.9)	51(21.4)
Median(IQR)	44(44,67)	56(33,67)	50(44,67)
Min,Max	(11,78)	(0,100)	(0,100)
Respiratory			
n	33	31	64
Mean(SD)	53.5(27.5)	54(27.3)	53.7(27.2)
Median(IQR)	50(33,78)	56(33,78)	56(33,78)
Min,Max	(0,100)	(6,100)	(0,100)
Digestion			
n	33	31	64
Mean(SD)	77.9(16.9)	80.4(26.4)	79.1(21.9)
Median(IQR)	78(67,89)	89(78,100)	89(67,100)
Min,Max	(44,100)	(0,100)	(0,100)
Role Functioning			
n	33	31	64
Mean(SD)	65.2(24.3)	64(25.9)	64.6(24.9)
Median(IQR)	67(50,83)	67(42,83)	67(50,83)
Min,Max	(0,100)	(8,100)	(0,100)
Vitality			
n	33	31	64
Mean(SD)	37.8(22.8)	40.6(22)	39.2(22.3)
Median(IQR)	33(17,50)	42(25,58)	42(25,58)
Min,Max	(8,92)	(0,75)	(0,92)
Health Perceptions			
n	33	31	64
Mean(SD)	47.8(27.7)	51.6(24.9)	49.6(26.3)
Median(IQR)	44(22,67)	56(33,67)	44(33,67)

For peer review only

Min,Max	(0,100)	(0,100)	(0,100)
Weight			
n	33	31	64
Mean(SD)	70.7(36.1)	63.4(39.8)	67.2(37.9)
Median(IQR)	100(33,100)	67(33,100)	83.5(33,100)
Min,Max	(0,100)	(0,100)	(0,100)

Table 7: Baseline COM-BMQ domains by treatment arm

	Intervention	Control	Overall
COM BMQ Necessities			
n	33	31	64
Mean(SD)	3.2(0.7)	3.4(0.8)	3.3(0.8)
Median(IQR)	3.1(2.7,3.7)	3.3(2.9,4.1)	3.1(2.7,4)
Min,Max	(2,4.9)	(2,4.7)	(2,4.9)
COM BMQ Concerns			
n	33	31	64
Mean(SD)	2.1(0.6)	2.2(0.6)	2.1(0.6)
Median(IQR)	2.1(1.5,2.6)	2.1(1.7,2.6)	2.1(1.6,2.6)
Min,Max	(1.2,3.4)	(1.1,3.3)	(1.1,3.4)

Primary Analysis

- In total, there were 79 exacerbations in participants followed up for at least 6 months
- Of these, 60 exacerbations fitted our criteria to be included in the primary analysis
  - 18 were not treated with IV antibiotics
  - 1 did not meet any Fuchs criteria
- A total of 60 participants had at least 6 months of exacerbation data (Intervention=32, Control =28)
- 4 participants were excluded
  - 2 died (Control=2)
  - 1 withdrew consent (Control=1)
  - 1 lost to follow up before 6 months (Intervention=1)
- 35 exacerbations occurred in Intervention participants, 25 occurred in Control participants
- 33 participants experienced at least 1 exacerbation (Intervention= 19 (60%), Control= 14 (50%))

The most frequently reported Fuchs criteria (Table 9) were 'Increased cough' (n=52) and 'Change in sputum (n=48). The median number of Fuchs criteria reported per exacerbation included in the primary analysis was 4 (IQR=4,6).

The median IV course length of exacerbations included in the primary analysis was 14 days in both the intervention and usual care arm (Table 12).

For peer review only

As ACtiF was a pilot study, it was not powered to detect an intervention effect. However, differences between treatment arms and their 95% confidence intervals have been calculated (Table 13). The median number of exacerbations was 1 in the intervention arm and 0.5 in the usual care arm. Following adjustment for site and the number of IV days in the previous year, adjusted IRR was 1.12 (95% CI: 0.658-1.94). This demonstrates a small increase in exacerbations in the intervention arm, however the confidence intervals are relatively wide. The IRR from the offset model shows an IRR of 0.958 (95% CI: 0.615,1.5). Here, a small decrease in exacerbations can be observed. As with the previous model, the confidence interval is relatively wide.

For peer review only

Exacerbations summary

Number of Exacerbations

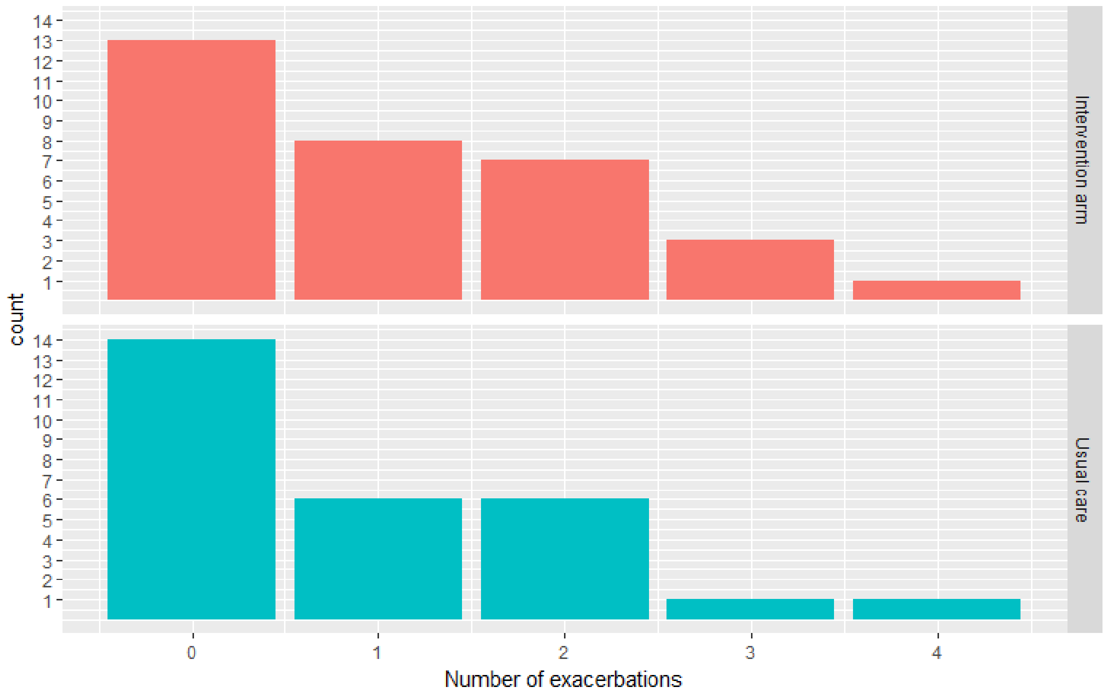

Figure 2:The number of exacerbations in participants by treatment arm in 6 months [n=60]

review only

Fuchs Criteria

Table 8:The number of each Fuchs criterion in the exacerbations used as the primary outcome

	n (%) for exacerbations in 6 months after consent and meeting our criteria (primary outcome)	n (%) for exacerbations treated with IV antibiotics and met at least one Fuchs criteria	n (%) for any exacerbation during the study
**Change in sputum**	48 (80 %)	63 (77.8 %)	69 (69 %)
**New or increased hemoptysis**	12 (20 %)	15 (18.5 %)	16 (16 %)
**Increased cough**	52 (86.7 %)	70 (86.4 %)	77 (77 %)
**Increased dyspnea**	43 (71.7 %)	56 (69.1 %)	61 (61 %)
**Malaise, fatigue, or lethargy**	48 (80 %)	66 (81.5 %)	69 (69 %)
**Temperature above 38 °C**	13 (21.7 %)	18 (22.2 %)	20 (20 %)
**Anorexia or weight loss**	20 (33.3 %)	30 (37 %)	31 (31 %)
**Sinus pain or tenderness**	13 (21.7 %)	19 (23.5 %)	21 (21 %)
**Change in sinus discharge**	13 (21.7 %)	21 (25.9 %)	22 (22 %)
**Change in physical examination of the chest, derived from notes by site staff.**	9 (15 %)	12 (14.8 %)	13 (13 %)
**Decrease in pulmonary function by 10 percent or more from a previously recorded value, derived from notes by site staff**	12 (20 %)	17 (21 %)	19 (19 %)
**Radiographic changes indicative of pulmonary infection, derived from notes by site staff)**	2 (3.3 %)	2 (2.5 %)	2 (2 %)

For peer review only

Table 9:Summary of Fuchs criteria for the exacerbations that were included in the primary outcome (IV days and at least 1 Fuchs criteria in 6 month follow up period

Description	
Exacerbations included in primary analysis	
n (%) with IV and at least 1 Fuchs	60 (60 %)
Mean (SD) number of Fuchs criteria	4.8 (2.1)
Median (IQR) number of Fuchs criteria	4 (4 , 6)
Min, max number of Fuchs criteria	(1,10)
n (%) of exacerbations with at least 2 Fuchs criteria	58 (96.7 %)
n (%) of exacerbations with at least 3 Fuchs criteria	48 (80 %)
n (%) of exacerbations with at least 4 Fuchs criteria	46 (76.7 %)
n (%) of exacerbations with at least 5 Fuchs criteria	29 (48.3 %)
n (%) of exacerbations with at least 6 Fuchs criteria	20 (33.3 %)
n (%) of exacerbations with at least 7 Fuchs criteria	12 (20 %)
n (%) of exacerbations with at least 8 Fuchs criteria	8 (13.3 %)
n (%) of exacerbations with at least 9 Fuchs criteria	3 (5 %)
n (%) of exacerbations with at least 10 Fuchs criteria	1 (1.7 %)

Table 10:Summary of the exacerbations in the 6 month follow up period that were not included in the primary outcome (IV days and at least 1 Fuchs criteria) and the reasons for exclusion

Exacerbations in 6 months not meeting criteria for primary outcome	
Total exacerbations excluded	19 (24 %)
n (%) with IV days but no Fuchs criteria met	1 (1 %)
n (%) with no IV but at least 1 Fuchs	7 (8 %)
n (%) no IV days or Fuchs recorded (missing values)	11 (14 %)

For peer review only

Length of IV course

Table 11:Summary of IV length by exacerbation and participant

	Intervention	Usual Care
IV days per exacerbation in 6 months		
n	35	25
Mean (SD)	13.6(4.2)	13.7(3.3)
Median (IQR)	14(13,14)	14(13,15)
Min, Max	(2,30)	(7,21)
IV days per participant with exacerbations in 6 months		
n	19	14
Mean (SD)	13.4(2.7)	13.6(3.2)
Median (IQR)	14(11,14)	14(13,15)
Min, Max	(9,21.7)	(8,20)
IV days per exacerbation in whole study		
n	45	36
Mean (SD)	13.7(4.1)	13.9(3.1)
Median (IQR)	14(13,14)	14(13,15)
Min, Max	(2,30)	(7,21)

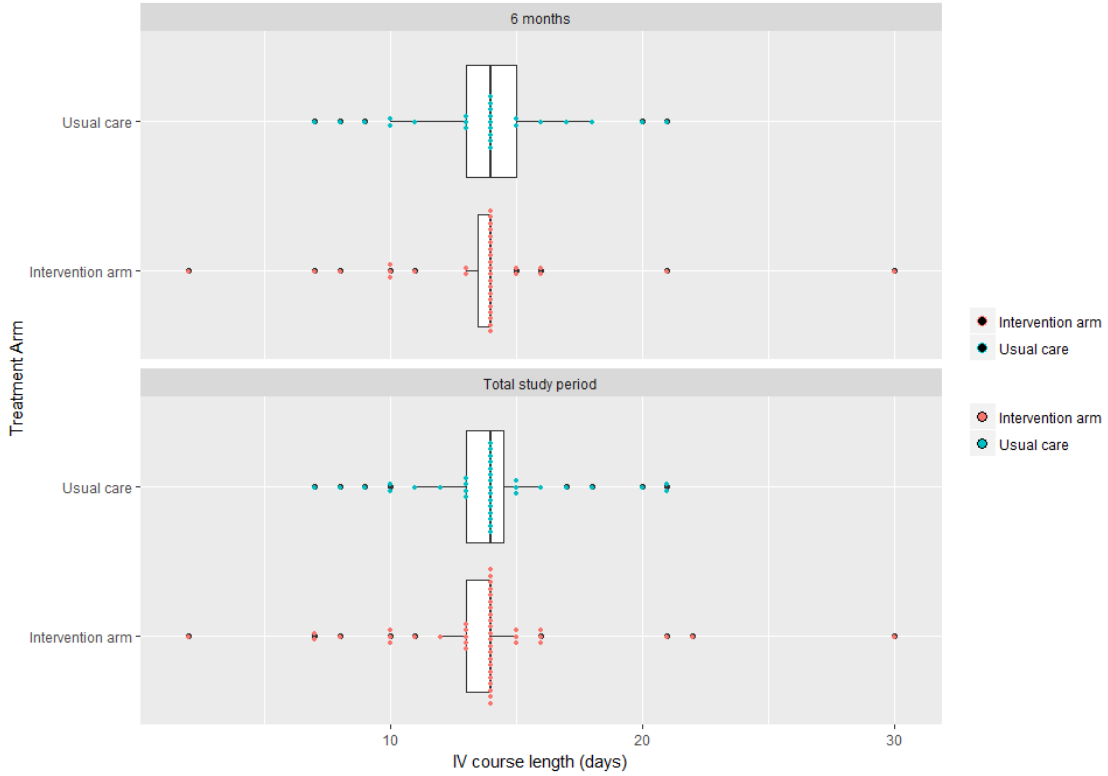

Figure 3:The length on IV courses by treatment arm

Analysis models

6 month model

Table 12:Analysis of the primary clinical outcome, the number of exacerbations treated with IV antibiotics with at least 1 Fuchs criteria in a 6 month period adjusted for site and the number of IV days in the previous year.

	Intervention n	Mean (SD)	Median (IQR)	Control n	Mean (SD)	Median (IQR)	IRR	95% CI
Unadjusted	32	1.1 (1.1)	1 (0 , 2)	28	0.9 (1.1)	0.5 (0 , 2)	1.22	(0.686,2.21)
Adjusted							1.12	(0.658,1.94)

Offset model

Table 13:A sensitivity analysis using all exacerbations treated with IV antibiotics with at least 1 Fuchs criteria that occurred during the study with the number of days of data collection included as an offset in the model. Adjusted for site and number of IV days in the previous year

	Intervention n	Total exacerbations (min,max)	Mean (SD) days followed up	Mean (SD) exacerbations per month	Control n	Total exacerbations (min,max)	Mean (SD) days followed up	Mean (SD) exacerbations per month	IRR	95% CI
**Adjusted, Offset model**	33	46(0,5)	263.2(47.2)	0.17(0.16)	31	40(0,5)	250.5(74.8)	0.2(0.28)	0.958	(0.615,1.5)

For peer review only

Secondary analysis

Tables 15-16 show the results of the secondary analyses. As this is a pilot study, we have not powered to detect any effect. Key results are described below.

- Adjusted mean difference of 5% (95% CI: -2-12%) in FEV % predicted. This is an encouraging difference in the intervention arm.
- No notable differences in any of the other secondary outcomes but this is not of great concern as it is a pilot study.
- Fewer participants had BMI recorded than other outcomes (Intervention=18, Control=15).
- Small reduction in BMQ Concerns score in intervention arm (Mean difference=-0.21, 95% CI: -0.38,-0.048).

Figure 4 shows the distribution of the secondary outcome measures at baseline and follow up by treatment arm.

Table 14:Results of secondary effectiveness analysis

	n Intervention	Median (IQR)	Mean (SD)	n Control	Median (IQR)	Mean (SD)	Mean Diff	95% CI
FEV1 Unadjusted	30	1.8(1.17,2.83)	2(0.9)	27	1.9(1.46,2.83)	2.2(1)	-0.21	(-0.73,0.3)
FEV1 Adjusted							0.22	(-0.062,0.51)
FEV1 % Unadjusted	30	51.8(33.46,71.26)	54.2(21.1)	27	50.9(42.49,77.97)	59(23.9)	-4.8	(-17,7.1)
FEV1 % Adjusted							5	(-2,12)
BMI Unadjusted	18	20.5(19.5,26)	22.1(4.2)	15	23.4(20.7,26.2)	23.8(3.5)	-1.7	(-4.5,1.1)
BMI Adjusted							-0.08	(-1,0.89)
EQ5D-5L Unadjusted	31	0.9(0.76,0.95)	0.9(0.2)	27	0.9(0.77,1)	0.9(0.2)	-0.00062	(-0.084,0.083)
EQ5D-5L Adjusted							-0.016	(-0.087,0.055)

For peer review only

PAM-13 Unadjusted	31	63.1(51,67.8)	58.5(14.3)	28	58.1(51,63.1)	57.9(9.9)	0.56	(-5.9,7)
PAM-13 Adjusted							0.046	(-5.8,5.9)
CHAOS Unadjusted	31	9(7,13)	9.9(3.9)	28	9(7.5,11.5)	9.4(3.3)	0.55	(-1.4,2.4)
CHAOS Adjusted							0.79	(-0.47,2.1)
MAD-3 Unadjusted	31	12(9,13)	10.8(3.9)	26	9.5(7,13)	9.4(3.6)	1.4	(-0.58,3.4)
MAD-3 Adjusted							0.82	(-0.51,2.1)
SRBAI Unadjusted	31	13(8,16)	12.1(5.3)	28	10.5(6,15.5)	10.6(5)	1.4	(-1.3,4.1)
SRBAI Adjusted							0.15	(-1.8,2.1)
GAD-7 Unadjusted	31	3(1,6)	4.1(4.1)	28	2.5(0,7)	4.2(4.4)	-0.05	(-2.3,2.2)
GAD-7 Adjusted							-0.31	(-1.9,1.3)
PHQ-8 Unadjusted	31	7(4,12)	7.3(5.2)	28	4(1.5,7)	5.3(5.1)	2	(-0.68,4.7)
PHQ-8 Adjusted							0.97	(-0.96,2.9)
COM-BMQ Concerns Unadjusted	31	2(1.5,2.3)	1.9(0.5)	27	2.1(1.9,2.4)	2.1(0.5)	-0.22	(-0.48,0.026)
COM-BMQ Concerns Adjusted							-0.21	(-0.38,-0.048)
COM BMQ Necessities Unadjusted	31	3.4(3,4)	3.5(0.6)	27	3.4(2.9,4)	3.5(0.7)	0.011	(-0.35,0.37)

For peer review only

COM BMQ Necessities Adjusted							0.12	(-0.16,0.4)

Table 15:Results of secondary effectiveness analysis

	n Intervention	Median (IQR)	Mean (SD)	n Control	Median (IQR)	Mean (SD)	Mean Diff	95% CI
CFQ-R Physical Unadjusted	31	54(25,88)	54.4(31.6)	28	62.5(33,92)	60.9(31.2)	-6.4	(-23,10)
CFQ-R Physical Adjusted							-2.6	(-13,7.4)
CFQ-R Emotional State Unadjusted	31	67(53,93)	68.3(23.4)	28	73(56.5,90)	72.3(22.7)	-4	(-16,8)
CFQ-R Emotional State Adjusted							-7.7	(-16,0.55)
CFQ-R Eating Unadjusted	31	89(67,100)	80.7(21.6)	28	83.5(67,100)	79.9(20.7)	0.85	(-10,12)
CFQ-R Eating Adjusted							1.1	(-6.5,8.7)
CFQ-R Social Unadjusted	31	67(56,78)	65.4(15.8)	28	64(50,83)	66.4(20.9)	-1	(-11,8.6)
CFQ-R Social Adjusted							-3.7	(-10,2.8)
CFQ-R Body Image Unadjusted	31	78(67,89)	73.3(23.8)	28	78(56,100)	73.1(25.5)	0.19	(-13,13)
CFQ-R Body Image Adjusted							0.62	(-7.2,8.5)
CFQ-R Treatment Burden Unadjusted	31	56(44,67)	56.5(16.6)	28	56(44,67)	57.3(19.9)	-0.83	(-10,8.7)
CFQ-R Treatment Burden Adjusted							1.2	(-6.4,8.8)
CFQ-R Respiratory Unadjusted	31	67(44,78)	59.5(25.2)	27	67(50,83)	65.6(22.7)	-6.1	(-19,6.6)
CFQ-R Respiratory Adjusted							-4.4	(-14,4.8)

For peer review only

CFQ-R Digestion Unadjusted	31	89(67,100)	81.1(18.4)	27	89(78,100)	84.4(23.5)	-3.3	(-14,7.7)
CFQ-R Digestion Adjusted							-2.3	(-11,6.2)
CFQ-R Role Unadjusted	31	75(33,83)	64.8(26.1)	27	75(56,92)	70.3(21.5)	-5.6	(-18,7.1)
CFQ-R Role Adjusted							-8.2	(-17,0.4)
CFQ-R Vital Unadjusted	31	42(25,42)	38.5(19.5)	28	50(33,62.5)	48.7(23)	-10	(-21,0.81)
CFQ-R Vital Adjusted							-7	(-15,0.99)
CFQ-R Health Unadjusted	31	44(22,67)	45.5(25.4)	28	61.5(33,72.5)	56.8(27.6)	-11	(-25,2.6)
CFQ-R Health Adjusted							-6.5	(-16,2.8)
CFQ-R Weight Unadjusted	31	89(67,100)	81.1(18.4)	27	89(78,100)	84.4(23.5)	-3.3	(-14,7.7)
CFQ-R Weight Adjusted							-2.3	(-11,6.2)

For peer review only

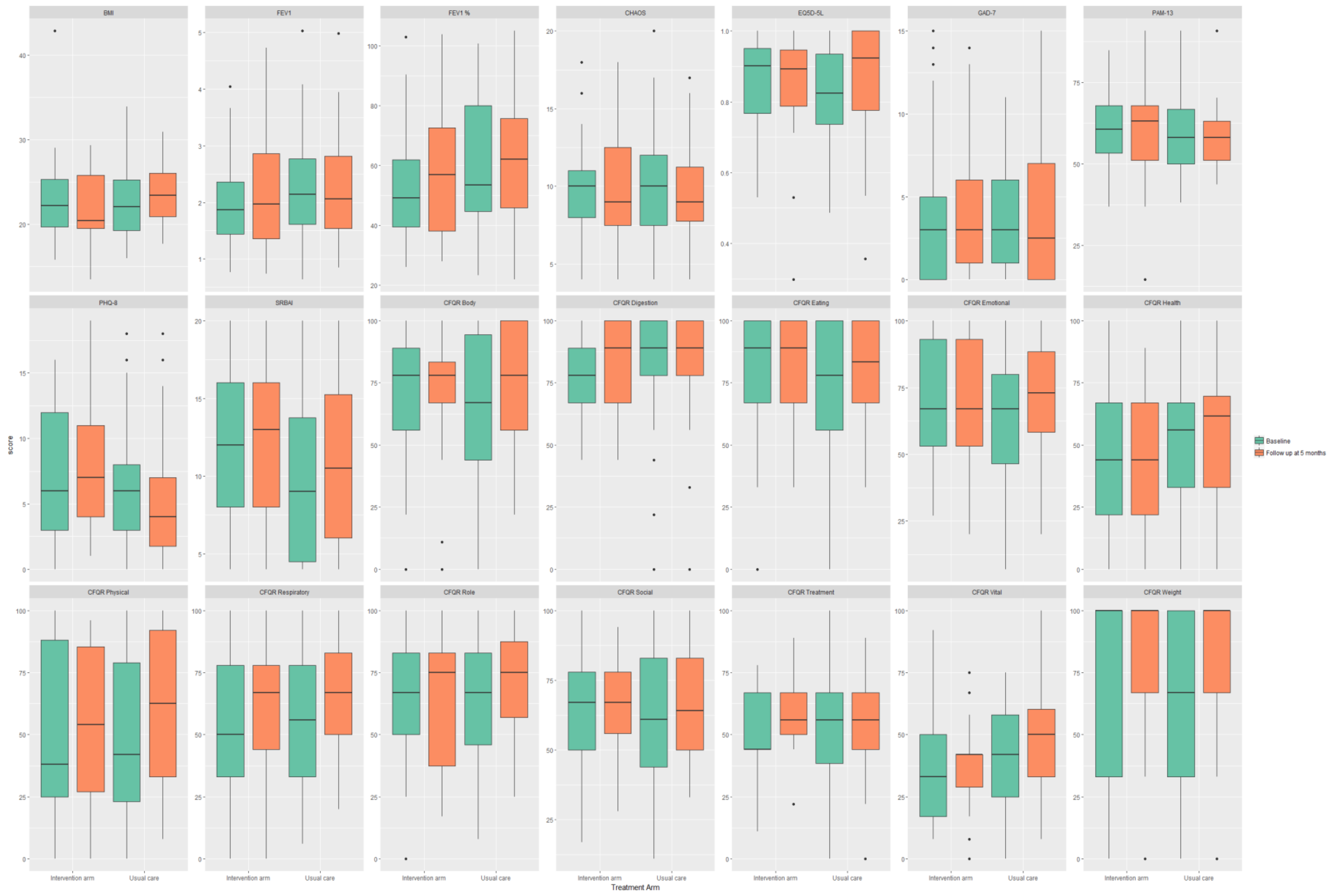

Figure 4:Box plots showing the distribution of secondary outcomes by treatment arm

For peer review only

Adherence to CF medication

During the trial, 8 participants withdrew from adherence data collection (Intervention=4, Control=4). An exact date of withdrawal was not recorded but could be seen from inhalation data (last non zero number of daily inhalations). This has been improved for the main trial and date of adherence data collection withdrawal will be recorded.

Participants who withdrew from adherence data collection were removed from summaries of adherence for 6 months as they did not have 6 months' worth of data. Where possible, inhalation data collected before withdrawal was included in the mean adherence by arm in the monthly table and the plot by week. The number included in each of these estimates can be seen in Table 18.

Table 17 shows the mean adherence by treatment arm for the 6 months post randomisation. Adherence is greater in the intervention arm for each of the different adherence measures. A difference of 10% (95% CI: -5.2 to 25.2) in simple normative adherence with numerator adjustment can be observed in the intervention arm. Table 18 shows the difference in simple normative adherence with numerator adjustment by treatment arm for each individual month in the study. Adherence is greater in the Intervention arm in month 1 (mean difference=2.6, 95% CI: -13.5,18.6). Following month 1, adherence is consistently higher in the intervention arm with the greatest difference observed in month 5 (mean difference: 13%, 95% CI: -4.8, 30.8). These differences would indicate a potentially clinically important difference between the intervention and usual care arms.

The difference in adherence has been presented by weeks post randomisation in Figure 5. There is a difference in numerator adjusted normative adherence with greater adherence observed in the intervention arm. This difference becomes clear after week 4 which coincides with use of the intervention around week 2-3.

Table 16:Summary of average adherences in 6 months following consent by intervention arm and the difference in means with 95% confidence intervals

	n Intervention	Mean Intervention	n Control	Mean Control	Mean Difference (95% CI)
Baseline (first 2 weeks)	29	25.9(31.4)	26	23.2(29)	2.6(-13.9,19.2)
Total doses	29	222.4(233.1)	26	245.7(238.6)	-23.3(-151.2,104.6)
Unadjusted adherence	29	47.7(33.8)	26	37.7(27.1)	10(-6.5,26.4)
Simple normative	29	45.5(32.8)	26	34.7(27)	10.8(-5.4,27)

For peer review only

Sophisticated normative	29	41.6(33.4)	26	34.2(27.1)	7.5(-8.9,23.9)
Simple normative with numerator adjustment	29	43.6(30.4)	26	33.6(25.9)	10(-5.2,25.2)
Sophisticated normative with numerator adjustment	29	39.9(30.9)	26	33.2(25.9)	6.8(-8.6,22.2)

Table 17:Summary of average adherences in each month from following consent from 1 to 6 months by intervention arm

	n Intervention	Mean Intervention	n Control	Mean Control	Mean Difference (95% CI)
Month 1	32	29.7(34.5)	28	27.2(27.5)	2.6(-13.5,18.6)
Month 2	31	42.1(33.1)	28	33.7(31.5)	8.4(-8.5,25.2)
Month 3	30	42.3(33.7)	28	33.3(34.8)	9(-9,27.1)
Month 4	29	42.7(34.7)	27	34.5(30.5)	8.2(-9.3,25.7)
Month 5	29	42.8(36.2)	27	29.8(30.1)	13(-4.8,30.8)
Month 6	29	41.3(36.5)	27	32.9(28.5)	8.4(-9.1,25.9)

For peer review only

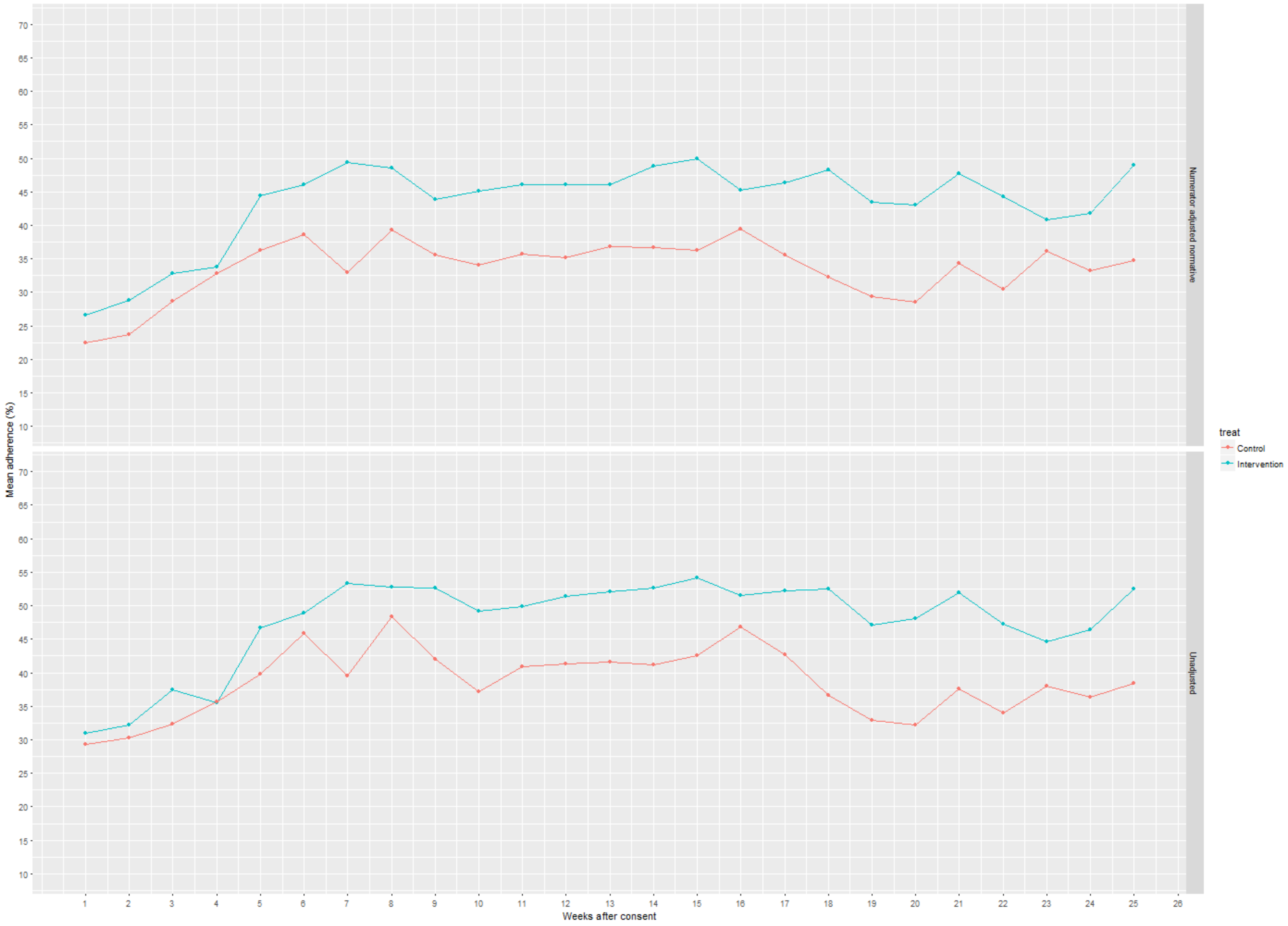

Figure 5:Mean weekly adherence by treatment arm

For peer review only

Intervention adherence (Participants)

Table 19 shows the median number of CFHH interactions was 3 (IQR: 1-8). 3 participants had no interactions with CFHH and the maximum number of interactions was 44. The mean total duration of interaction time across the study was 49.3 (SD= 44.8) minutes. The mean length of an interaction by participant was 12.4 (SD=9.6) minutes and the mean length of all interactions was 6.6 (SD=11) minutes. The median number of days in the trial with interactions was 2 (IQR=1,7) by participant. Figure 6 shows the wide range of values across participants, particularly for the total duration of interactions.

Figure 7 shows when interactions occurred in days for each participant. Some participants were interacting fairly regularly, however most participants were inconsistent with their interactions. Figure 8 shows that the 'How am I doing?' pages were the most frequently visited in terms of the total number of clicks during the trial. 30 (90.9%) of participants visited the 'How am I doing?', 'Treatment' and 'Videos' page at least once (Table 20). 224 (91.4%) sessions included a visit to the 'How am I doing?' page.

For peer review only

Table 18:Summary of clicks in CFHH. An interaction is defined as a series of clicks with no greater than a 15 minute lag between clicks

Interactions with CFHH by participant	
n	33
Mean (SD)	7.4(11.6)
Median (IQR)	3(1,8)
Min, Max	(0,44)
Total duration of interactions by participant	
n	33
Mean (SD)	49.3(44.8)
Median (IQR)	38(26,55)
Min, Max	(0,177)
Mean duration of interactions by participant	
n	33
Mean (SD)	12.4(9.6)
Median (IQR)	10.7(4.3,19)
Min, Max	(0,37)
Days with interactions by participant	
n	33
Mean (SD)	5.7(8.2)
Median (IQR)	2(1,7)
Min, Max	(0,32)
Duration of interactions	
n	245
Mean (SD)	6.6(11)
Median (IQR)	1(0,8)
Min, Max	(0,57)

For peer review only

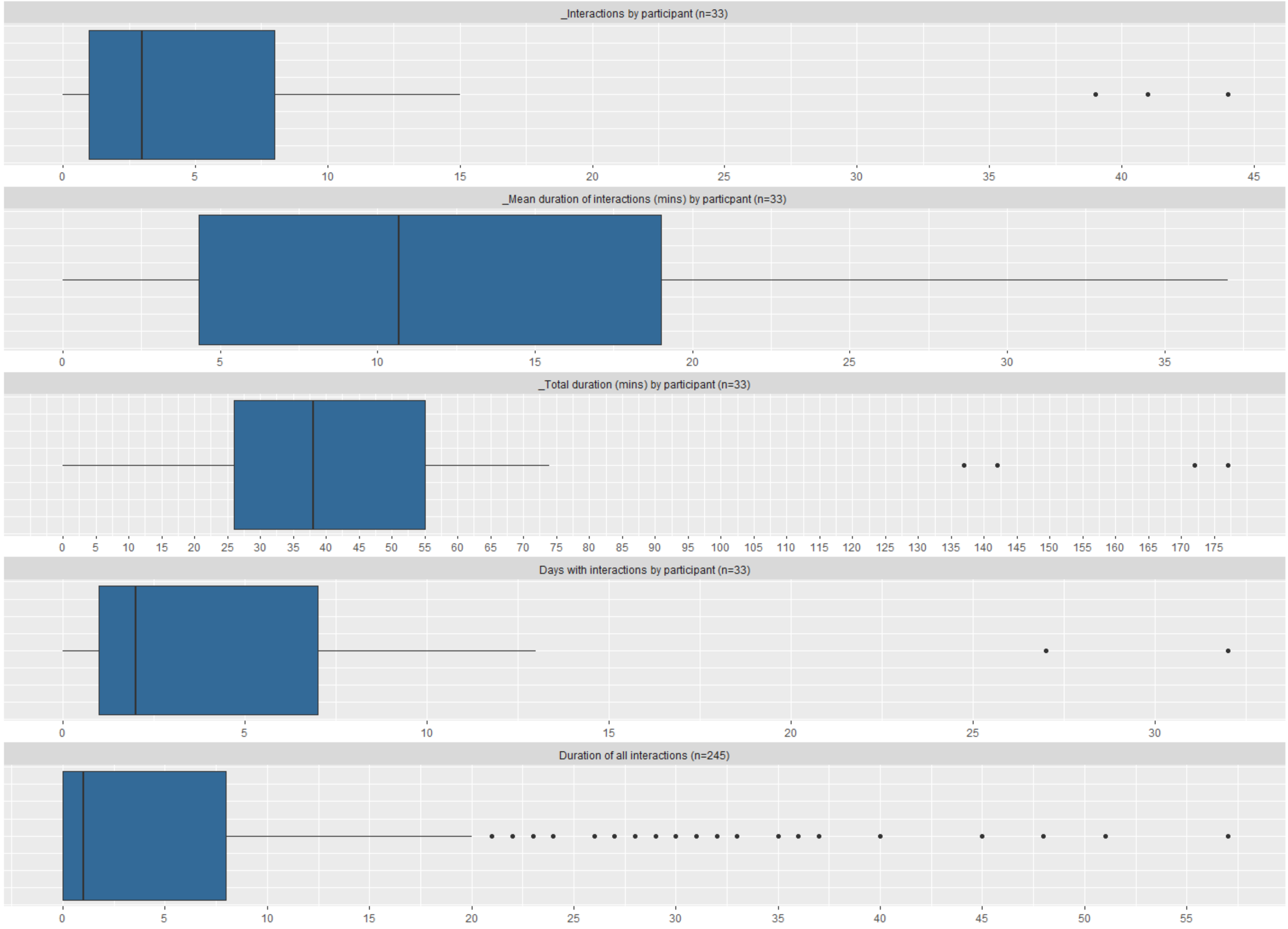
_Interactions by participant (n=33)
0 5 10 15 20 25 30 35 40 45
_Mean duration of interactions (mins) by particpant (n=33)
0 5 10 15 20 25 30 35
_Total duration (mins) by participant (n=33)
0 5 10 15 20 25 30 35 40 45 50 55 60 65 70 75 80 85 90 95 100 105 110 115 120 125 130 135 140 145 150 155 160 165 170 175
Days with interactions by participant (n=33)
0 5 10 15 20 25 30
Duration of all interactions (n=245)
0 5 10 15 20 25 30 35 40 45 50 55

Figure 6:Boxplots showing summaries of click analytics in CFHH

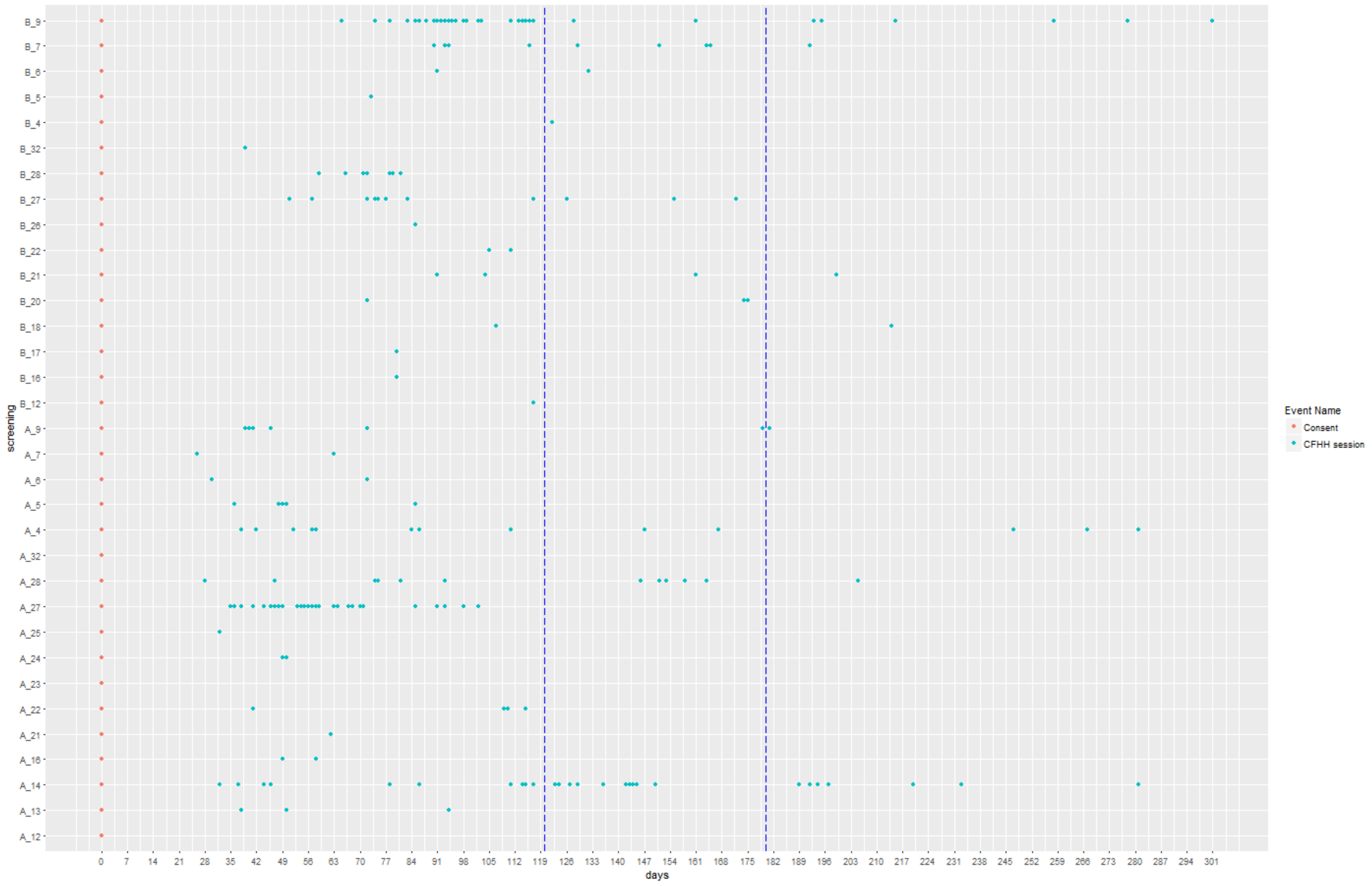

Figure 7:Timing in days of interactions with CFHH

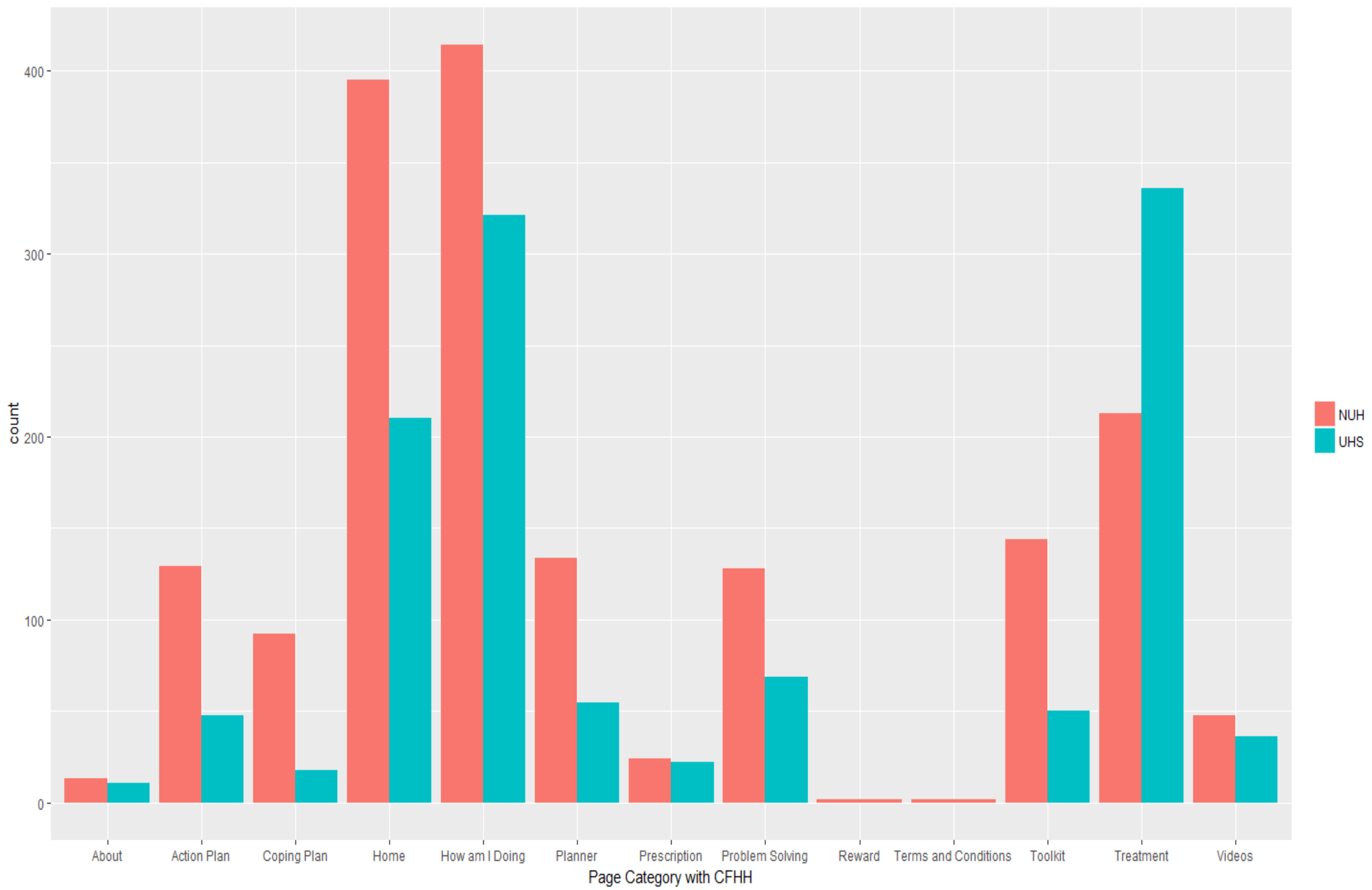

Figure 8:Frequency of clicks by CFHH categories

Table 19:Summary of clicks by page categories in CFHH

	Total (%) clicks	Participants (%) with at least one click	Sessions (%) with at least one click
**About**	24(0.8%)	13(39.4%)	20(8.2%)
**Action Plan**	177(6.1%)	28(84.8%)	53(21.6%)
**Coping Plan**	110(3.8%)	24(72.7%)	38(15.5%)
**Home**	605(20.8%)	30(90.9%)	244(99.6%)
**How am I Doing**	735(25.2%)	30(90.9%)	224(91.4%)
**Planner**	189(6.5%)	21(63.6%)	39(15.9%)
**Prescription**	46(1.6%)	22(66.7%)	42(17.1%)
**Problem Solving**	197(6.8%)	24(72.7%)	44(18%)
**Reward**	2(0.1%)	2(6.1%)	2(0.8%)
**Terms and Conditions**	2(0.1%)	2(6.1%)	2(0.8%)
**Toolkit**	194(6.7%)	24(72.7%)	66(26.9%)
**Treatment**	549(18.8%)	30(90.9%)	87(35.5%)
**Videos**	84(2.9%)	30(90.9%)	62(25.3%)

For peer review only

Intervention fidelity (Clinicians)

Table 21 shows the median number of intervention sessions per participant was 3 (IQR= 2,4) with a mean duration of 36.1 (SD=23.9) minutes.

Table 20:Summary of intervention sessions received by intervention participants during the study

Sessions per participant	
n	33
Mean (SD)	3(1.6)
Median (IQR)	3(2,4)
Min, Max	(0,6)
Total time by participant	
n	33
Mean (SD)	114.2(46.9)
Median (IQR)	100.5(90,125)
Min, Max	(40,249)
Time per session by participant	
n	33
Mean (SD)	37.3(14.2)
Median (IQR)	31.3(28.3,48)
Min, Max	(18,65)
Time per session	
n	99
Mean (SD)	36.1(23.9)
Median (IQR)	30(15,55)
Min, Max	(4,119)
Intervention session per participant	
n	33
Mean (SD)	0.9(0.3)
Median (IQR)	1(1,1)
Min, Max	(0,1)
Total Intervention session time per participant	
n	29
Mean (SD)	58.1(14.2)
Median (IQR)	60(48,60)
Min, Max	(35,90)

For peer review only

Review session per participant	
n	33
Mean (SD)	1(0.5)
Median (IQR)	1(1,1)
Min, Max	(0,2)
Total Review session time per participant	
n	29
Mean (SD)	43.2(30.6)
Median (IQR)	40(20,55)
Min, Max	(10,154)
Preparation session per participant	
n	33
Mean (SD)	0.7(0.9)
Median (IQR)	0(0,1)
Min, Max	(0,3)
Total Preparation session time per participant	
n	14
Mean (SD)	18.4(9.7)
Median (IQR)	15(15,30)
Min, Max	(4,35)
Ad hoc sessions per participant	
n	33
Mean (SD)	0.4(0.6)
Median (IQR)	0(0,1)
Min, Max	(0,2)
Total ad hoc session time per participant	
n	12
Mean (SD)	19.2(6.7)
Median (IQR)	15(15,25)
Min, Max	(15,30)

For peer review only

Clinic visits

Participants completed a median of 2 clinic visits. This was consistent across treatment arms. The number of clinic visits by participant is similar across treatment arms (Figure 9).

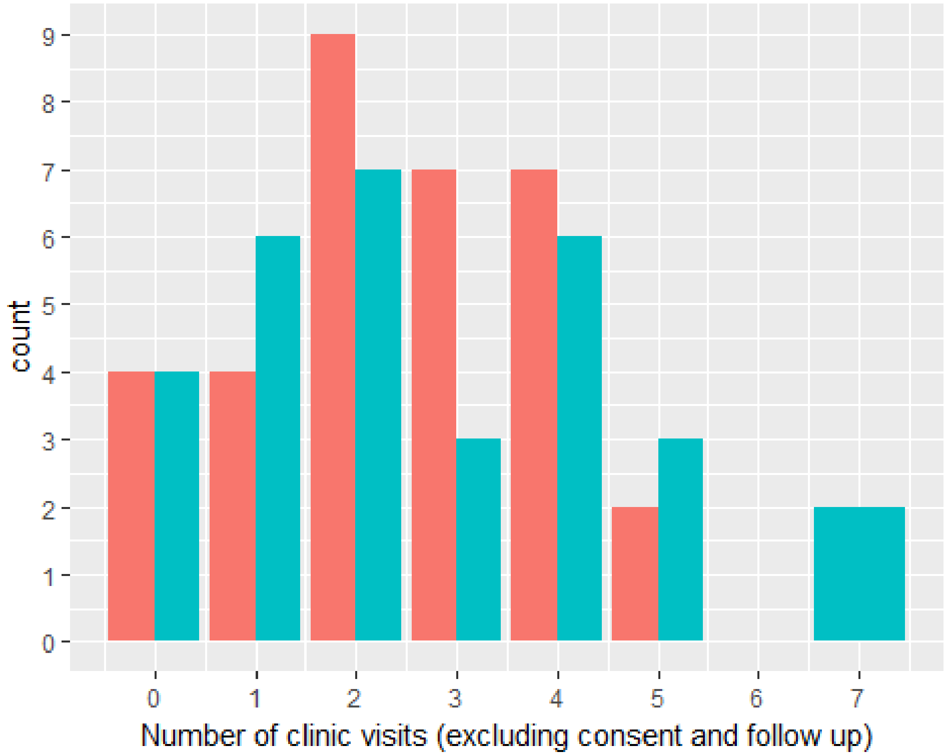

Figure 9:Barplot showing the number of participants for each number of clinic visits by treatment arm

For peer review only - http://bmjopen.bmj.com/site/about/guidelines.xhtml

Safety analysis

A total of 8 adverse events (AEs) occurred during the trial and 7 participants (10.9%) had a least one AE (Table 22). 5 of these were deemed to be Serious Adverse Events (SAEs). None of the SAEs were related to the intervention.

Table 21:Summary of adverse events recorded during the study

	Intervention n (%)	Control n (%)	Overall n (%)
All Adverse Events	5	3	8
Participants with at least 1 AE	4(12.1%)	3(9.7%)	7(10.9%)
Type of Adverse Event			
Chest pain or chest discomfort	1(25%)	0(0%)	1(14.3%)
Voice change or Alteration	0(0%)	0(0%)	1(14.3%)
Other	4(100%)	2(66.7%)	6(85.7%)

Table 22:Summary of serious adverse events recorded during the study

	Intervention n (%)	Control n (%)	Overall n (%)
All Serious Adverse events	3(9.1%)	2(6.5%)	5(7.8%)
Level of Seriousness			
Death	0(0%)	2(100%)	2(40%)
Hospitalisation	2(66.7%)	0(0%)	2(40%)
Persistent or significant disability/incapacity	1(33.3%)	0(0%)	1(20%)
Frequency			
Isolated	2(66.7%)	2(100%)	4(80%)
Continuous	1(33.3%)	0(0%)	1(20%)
Intensity			
Moderate	3(100%)	0(0%)	3(60%)
Severe	0(0%)	2(100%)	2(40%)
Outcome			
Recovered	1(33.3%)	0(0%)	1(20%)
Improved	2(66.7%)	0(0%)	2(40%)
Death	0(0%)	2(100%)	2(40%)
Expected SAE			
No	3(100%)	2(100%)	5(100%)
Related to Intervention			
No	3(100%)	2(100%)	5(100%)

For peer review only

For peer review only

Table 23:Description of serious adverse events recorded during the study (table has been redacted to maintain anonymity)

Participant ID	Description of event	Serious
xxx_15	Patient admitted on xx.xx.16 with acute exacerbation, developed type 2 respiratory failure. Despite maximal treatment of IV antibiotics, oxygen and NIV the patient continued to deteriorate and decision made to palliate. The patient died shortly afterwards.	Yes
xxx_14	Patient was having a kidney biopsy and had a bleed as a result, so had been kept in hospital on xxxxx ward at xxx city campus.	Yes
xxx_23	Patient admitted xx/xx/2016 with worsening disease and type 2 respiratory failure. Treated with non -invasive ventilation and intravenous antibiotics. deteriorated despite treatment and passed away xx/xx/2016	Yes
xxx_17	Rash reoccurred after re-trying oral antibiotic medication. Advised to stop again	No
xxx_17	Patient on holiday. Telephoned to report rash on both legs after starting new oral antibiotics. Advised to discontinue	No
xxx_20	Patient was admitted with influenza and CF. Exacerbation treated with iv antibiotics, discharged with home IV's. readmitted on the xx xxx with AKI (Acute Kidney Injury)<br>Assumed secondary to dehydration. Dornase stopped	Yes

Protocol non-compliances

In total, there were 9 protocol non compliances during the trial. 6 (67%) of these were follow up visits conducted outside of the calculated window (5 +/-1 month). 3 (33%) of these were participants ticking statements on the consent form rather than initialling. All of these protocol non compliances were assessed as minor non-compliances.

For peer review only

Summary of missing data

Exacerbation data was collected for 6 months in 60/64 participants (94%). Adherence was collected for at least 6 months for 58/64 participants (90%).

The number of missing scores for questionnaires completed at baseline and 5 month follow up was very low (Table 25). Completion rate was 100% for the majority of baseline questionnaires and at least 89% for 5 month questionnaires. Missing scores were due to drop out(described in section 2.1). Such high completion rates are reassuring for the main trial.

Table 24:Summary of missing scores and items within questionnaires

	Time	Total	%	Intervention Median (min,max)	Control Median (min,max)	Overall Median (min,max)
**EQ5D-5L**	Baseline	64	100 %	5 (5 , 5)	5 (5 , 5)	5 (5 , 5)
**5 items**	5 (+/-1) months	58	90.6 %	5 (0 , 5)	5 (0 , 5)	5 (0 , 5)
**PAM-13**	Baseline	64	100 %	13 (13 , 13)	13 (13 , 13)	13 (13 , 13)
**13 item**	5 (+/-1) months	59	92.2 %	13 (0 , 13)	13 (0 , 13)	13 (0 , 13)
**CHAOS**	Baseline	64	100 %	4 (4 , 4)	4 (4 , 4)	4 (4 , 4)
**4 items**	5 (+/-1) months	59	92.2 %	4 (0 , 4)	4 (0 , 4)	4 (0 , 4)
**MAD-3**	Baseline	62	96.9 %	3 (1 , 3)	3 (0 , 3)	3 (0 , 3)
**3 items**	5 (+/-1) months	57	89.1 %	3 (0 , 3)	3 (0 , 3)	3 (0 , 3)
**SRBAI**	Baseline	63	98.4 %	4 (0 , 4)	4 (4 , 4)	4 (0 , 4)
**4 items**	5 (+/-1) months	59	92.2 %	4 (0 , 4)	4 (0 , 4)	4 (0 , 4)
**GAD-7**	Baseline	64	100 %	7 (7 , 7)	7 (7 , 7)	7 (7 , 7)
**7 items**	5 (+/-1) months	59	92.2 %	7 (0 , 7)	7 (0 , 7)	7 (0 , 7)
**PHQ-8**	Baseline	64	100 %	8 (8 , 8)	8 (8 , 8)	8 (8 , 8)
**8 items**	5 (+/-1) months	59	92.2 %	8 (0 , 8)	8 (0 , 8)	8 (0 , 8)

For peer review only

Recommendations for Main Trial/ Points for discussion

- For the primary analysis in the main trial, we would recommend the use of the offset adjusted model as this will allow the use of more data and allows the inclusion of potentially important participants over a greater amount of time. For example, our original model excluded participants who died, however doing so means we have lost key information.
- This is a pilot study, not powered to detect an effect
- The nature of the data means that small changes appear to influence the result greatly

For peer review only

Appendix

Description of the patient reported outcomes

Name	Score range	Description	Interpretation of score
**EQ-5D-5L**	-0.224-1	Measure of health status	A score of zero means death, 1 is full health, negative score is a state worse than death
**PAM-13**	0-100	Measures patient activation e.g. ability and willingness to manage their health. 13 items with scoring spreadsheet	0= low patient activation<br>100= high patient activation
**CHAOS-6**	0-24	Measures confusion, hubbub and order. 6 item questionnaire	0= low level of chaos<br>24= high level of chaos
**SRBAI**	0-28	Measure of habit and automaticity 4 item, 7 point likert scale	0= low level of automaticity<br>28= high level of automaticity
**CFQ-R**	0-100	8 domains each score 0-100. The domains are:<br>Physical, Emotion, Social, Eating, Body, Treatment Burden, Respiratory, Digestion	0= low<br>100= high
**PHQ-8**	0-24	Measure of depression. 8 item questionnaire, 0-3 for each item	0= No or minimal depression<br>24= Severe depression
**GAD-7**	0-21	Measure of anxiety. 7 item questionnaire	0= No anxiety<br>21= Severe anxiety
**COM-BBQ**			
Specific Necessities	2-5	Measure of perceived personal need for medication	Direction of effect would be an increase in score
Specific Concerns	1-3	Measure of perceived concerns about the negative effects of the medicine they are taking	Direction of effect would be a decrease in score
**MAD-3**	3-15	Specifically made 3 item questionnaire to measure perceived medication adherence	3= low<br>15= high

For peer review only

Additional File 07. Changes to intervention procedures

Change Number	Problem type	Problem Identified	Solutions implemented in full-scale trial (hashed numbers - # - refer to logic model constructs)	Timing of change implementation
**CFHealthHub IT component**				
1	Real World and Trial	Interventionists having difficulty identifying videos (#22) appropriate for a patient's needs or interests.	Descriptions were provided with each video. The PPI group agreed with this change and assisted with writing descriptions for each video.	During the feasibility study
2	Real World and Trial	Adherence charts (#14, #20) were showing >100% adherence. This appeared to be more common in patients with alternating regimes, or taking medications *pro re nata* (PRN, meaning 'as needed').	Prescription flow amended with the addition of PRN or alternating regime alerts, which will assist the data management team in highlighting any data discrepancies.	Post-feasibility study
3	Real World and Trial	Clinician functionality (amending prescriptions/ treatment targets (#3, #23) inaccessible through participant view (used in intervention sessions).	Participant view functionality implemented to facilitate intervention sessions. Clinicians are now able to run intervention sessions using CFHH through participant view but easily switch to clinician view to change prescriptions and to set goals.	Post-feasibility study

For peer review only - http://bmjopen.bmj.com/site/about/guidelines.xhtml

4	Real World and Trial	The lead psychologist identified the need to determine which participants were receiving push notifications as this relates to dose and rewards for adherence.	The option to export data about number of push notifications sent to participants from the app (#16).	Post-feasibility study
5	Real World and Trial	Originally the normative adherence was used to come up with the percentage adherence. It was identified this did not always match what participants were actually prescribed and this made the graphs difficult to interpret. The capping of the weekly graph at 100% also made interpretation difficult.	To improve interpretability of adherence data (#14), percentages are now calculated against the actual treatments prescribed and graphs are not capped at 100% to aid any interpretation of graphs and trouble shooting.	Post-feasibility study
**Other IT infrastructure**				
6	Real World and Trial	Flatlines at the beginning of some participant adherence run charts were identified to relate to the date registered at the time the nebuliser (#4) is paired with the Qualcomm Hub (#5). Flatlines at the end of the feasibility study were also observed (#14, #35).	To achieve quality assurance of adherence data (#4, #5, #14, #35), hardware is now paired at the factory. The full-scale trial has been monitoring for, and has not found, such instances. Flatlines at the end of run charts established as genuine through triangulation with self-report quantitative and qualitative data.	Post-feasibility study

For peer review only - http://bmjopen.bmj.com/site/about/guidelines.xhtml

**Interventionist training and manual**				
7	Real World and Trial	Training packages were initially developed for physiotherapists. This led to interventionist recruitment problems.	The job specification and training was redeveloped to suit non-physiotherapists (#9, #12), to enable any member of the MDT to be trained up to deliver the intervention. A suitably qualified individual such as a postgraduate psychologist could be supported by the MDT to deliver the intervention.	Post-feasibility study
8	Real World and Trial	The interventionist job specification did not reflect the flexibility needed to carry out the interventionist role- e.g. flexibility in working patterns, skills in motivational interviewing and extensive travel.	The research team, with input from the interventionists, revised the job specification for the interventionist role based on experience of delivering the intervention in the pilot in order to better manage expectations of the role **(#12).**	Post-feasibility study
9	Real World and Trial	Pilot study interventionists felt that training was good but could be helped by introducing case studies with real world data, in CFHealthHub.	Realistic case studies with data to support interventionist training / role plays for using website were developed to provide training more applicable to real CF patients (#9). This model is generally used in a healthcare training setting.	Post-feasibility study
10	Real World and Trial	Sporadic training over six weeks, whilst also conducting research procedures was	Training was condensed into an intensive course over ten days, focusing solely on intervention delivery (#9).	Post-feasibility study

For peer review only - http://bmjopen.bmj.com/site/about/guidelines.xhtml

		overwhelming for interventionists.		
11	Real World and Trial	Assessment of intervention fidelity identified that some of the active ingredients of the intervention were absent e.g. negotiating goals and letting participants take ownership of choices.	The recruitment and training process was modified to incorporate role play at the interview; explaining fidelity assessment criteria during training and also on-going assessment to ensure that any issues are identified quickly (#9).	Post-feasibility study
12	Real World and Trial	The focus of interventionists during intervention delivery was not always on the aspects that evidence would indicate are the most active ingredients for example goal setting, action planning and coping planning.	Emphasis was placed on the main 'active ingredients' in the manual and in training (#8, #9).	Post-feasibility study
13	Real World and Trial	During the course of the trial, it became apparent that participants were not being followed up and engaged in a manner to allow them to build a habit.	Focus on habit formation / revised logic model will be implemented by a 6-8 week period of habit formation sessions (#8).	Post-feasibility study
14	Real World and Trial	It was identified that after some participants last review visit, their adherence to treatment dropped.	For the full RCT, intervention visits are now triggered if the participant is having an exacerbation/IV, has a drop of 20% or more adherence in the last 4 weeks and if the participant requests additional support.	Post-feasibility study

For peer review only - http://bmjopen.bmj.com/site/about/guidelines.xhtml

			These will be termed 'intervention triggers' (#8).	

For peer review only

For peer review only - http://bmjopen.bmj.com/site/about/guidelines.xhtml

Good Reporting of A Mixed Methods Study (GRAMMS).

O'Cathain A, Murphy E, Nicholl J. The quality of mixed methods studies in health services research. *J Health Serv Res Policy* 2008;13:92–8. doi:10.1258/jhsrp.2007.007074

Added to the EQUATOR Network database 26/09/2013.

(1) Describe the justification for using a mixed methods approach to the research question.

p5; lines 93-95.

(2) Describe the design in terms of the purpose, priority and sequence of methods

p11; line 240: we used a modified triangulation protocol; the study is described as nested, indicating, that the methods were used concurrently (p9; line 192).

(3) Describe each method in terms of sampling, data collection and analysis

Pages 9-12; 195-273.

(4) Describe where integration has occurred, how it has occurred and who has participated in it

Pages 11-12; Lines 240-273.

(5) Describe any limitation of one method associated with the present of the other method

Page 25; Lines 576-582.

(6) Describe any insights gained from mixing or integrating methods

p5; lines 93-95. p25; lines 573-611.

For peer review only

BMJ Open

Feasibility study for supporting medication adherence for adults with cystic fibrosis: mixed-methods process evaluation

Journal:	*BMJ Open*
Manuscript ID	bmjopen-2020-039089.R1
Article Type:	Original research
Date Submitted by the Author:	27-Jul-2020
Complete List of Authors:	Hind, Daniel; The University of Sheffield, ScHARR<br>Drabble, Sarah; The University of Sheffield, Medical Care Research Unit, School of Health and Related Research (ScHARR)<br>Arden, Madelynne; Sheffield Hallam University, Centre for Behavioural Science and Applied Psychology<br>Mandefield, Laura; University of York, Health Sciences<br>Waterhouse, Simon; The University of Sheffield, CTRU<br>Maguire, Chin; University of Sheffield,<br>Cantrill, Hannah; The University of Sheffield, CTRU<br>Robinson, Louisa; The University of Sheffield<br>Beever, Daniel; The University of Sheffield, CTRU<br>Scott, Alex; Sheffield University, School of Health and Related Research<br>Keating, Sam; The University of Sheffield<br>Hutchings, Marlene; Sheffield Teaching Hospitals NHS Foundation Trust<br>Bradley, Judy; Queen's University Belfast, School of Medicine, Dentistry and Biomedical Sciences<br>Nightingale, Julia ; University Hospital Southampton NHS Foundation Trust<br>Allenby, Mark; University Hospital Southampton NHS Foundation Trust<br>Dewar, Jane; Nottingham University Hospitals NHS Trust<br>Whelan, Pauline; The University of Manchester, Health e-Research Centre<br>Ainsworth, John; The University of Manchester, Division of Informatics, Imaging & Data Sciences<br>Walters, Stephen; University of Sheffield, ScHARR<br>Wildman, Martin; Sheffield Teaching Hospitals NHS Foundation Trust<br>O'Cathain, Alicia; University of Sheffield, School of Health and Related Research
**Primary Subject Heading**:	Respiratory medicine
Secondary Subject Heading:	Evidence based practice, Health informatics, Health services research, Patient-centred medicine, Qualitative research
Keywords:	Cystic fibrosis < THORACIC MEDICINE, Health informatics < BIOTECHNOLOGY & BIOINFORMATICS, Clinical trials < THERAPEUTICS, STATISTICS & RESEARCH METHODS, SOCIAL MEDICINE, Quality in health care < HEALTH SERVICES ADMINISTRATION & MANAGEMENT

SCHOLARONE™
Manuscripts

I, the Submitting Author has the right to grant and does grant on behalf of all authors of the Work (as defined in the below author licence), an exclusive licence and/or a non-exclusive licence for contributions from authors who are: i) UK Crown employees; ii) where BMJ has agreed a CC-BY licence shall apply, and/or iii) in accordance with the terms applicable for US Federal Government officers or employees acting as part of their official duties; on a worldwide, perpetual, irrevocable, royalty-free basis to BMJ Publishing Group Ltd ("BMJ") its licensees and where the relevant Journal is co-owned by BMJ to the co-owners of the Journal, to publish the Work in this journal and any other BMJ products and to exploit all rights, as set out in our licence.

The Submitting Author accepts and understands that any supply made under these terms is made by BMJ to the Submitting Author unless you are acting as an employee on behalf of your employer or a postgraduate student of an affiliated institution which is paying any applicable article publishing charge ("APC") for Open Access articles. Where the Submitting Author wishes to make the Work available on an Open Access basis (and intends to pay the relevant APC), the terms of reuse of such Open Access shall be governed by a Creative Commons licence – details of these licences and which Creative Commons licence will apply to this Work are set out in our licence referred to above.

Other than as permitted in any relevant BMJ Author's Self Archiving Policies, I confirm this Work has not been accepted for publication elsewhere, is not being considered for publication elsewhere and does not duplicate material already published. I confirm all authors consent to publication of this Work and authorise the granting of this licence.

For peer review only

Feasibility study for supporting medication adherence for adults with cystic fibrosis: mixed-methods process evaluation

Daniel Hind[1§], Sarah J Drabble[2], Madelynne A Arden[3], Laura Mandefield[4], Simon Waterhouse[1], Chin Maguire[1], Hannah Cantrill[1], Louisa Robinson[1], Daniel Beever[1], Alex Scott[1], Sam Keating[1], Marlene Hutchings[5], Judy Bradley[6], Julia Nightingale[7], Mark I Allenby[7], Jane Dewar[8], Pauline Whelan[9], John Ainsworth[9], Stephen J Walters[2], Martin J Wildman[2,5], Alicia O'Cathain[2].

[1]Clinical Trials Research Unit, University of Sheffield, Regent Court, 30 Regent Street, Sheffield, S1 4DA, UK

[2]School of Health and Related Research, Regent Court, 30 Regent Street, Sheffield, S1 4DA, UK

[3]Centre for Behavioural Science and Applied Psychology, Sheffield Hallam University, Collegiate Crescent, Sheffield, S10 2BQ

[4] Department of Health Sciences, University of York, University of York, Seebohm Rowntree Building, Heslington, York, YO10 5DD

[5] Sheffield Adult Cystic Fibrosis Unit Sheffield Teaching Hospitals NHS Foundation Trust, Northern General Hospital, Herries Road, Sheffield, S5 7AU

[6] Wellcome-Wolfson Institute For Experimental Medicine, School of Medicine, Dentistry and Biomedical Sciences, Queen's University Belfast, UK..

[7] Wessex Adult Cystic Fibrosis Service, University Hospital Southampton NHS Foundation Trust, Tremona Road, Southampton, Hampshire, SO16 6YD.

[8] Wolfson Cystic Fibrosis Centre, Nottingham University Hospitals NHS Trust, City Hospital, Hucknall Road, Nottingham, NG5 1PB.

For peer review only

For peer review only - http://bmjopen.bmj.com/site/about/guidelines.xhtml

[9] Health eResearch Centre - Division of Imaging, Informatics and Data Sciences, School of Health Sciences, Faculty of Biology, Medicine and Health, The University of Manchester, Manchester Academic Health Science Centre, Manchester, United Kingdom

[§] Corresponding author: Daniel Hind, Clinical Trials Research Unit, University of Sheffield, Regent Court, 30 Regent Street, Sheffield, S1 4DA, UK.

d.hind@sheffield.ac.uk

Email addresses:

DH: d.hind@sheffield.ac.uk

SD: s.j.drabble@sheffield.ac.uk

Madelynne A: M.Arden@shu.ac.uk

LM: l.mandefield@sheffield.ac.uk

SW: s.waterhouse@sheffield.ac.uk

CM: c.maguire@sheffield.ac.uk

HC: h.cantrill@sheffield.ac.uk

LR: louisa.robinson@sheffield.ac.uk

DB: d.a.beever@sheffield.ac.uk

AS: alex.scott@sheffield.ac.uk

SK: s.m.keating@sheffield.ac.uk

MH: marlene.hutchings@sth.nhs.uk

JB: judy.bradley@qub.ac.uk

JN: julia.nightingale@uhs.nhs.uk

Mark A: mark.allenby@uhs.nhs.uk

JD: jane.dewar@nuh.nhs.uk

For peer review only

PW: pauline.whelan@manchester.ac.uk

JA: John.Ainsworth@manchester.ac.uk

SJW: s.j.walters@sheffield.ac.uk

AOC: a.ocathain@sheffield.ac.uk

MW: martin.wildman@sth.nhs.uk

For peer review only

For peer review only - http://bmjopen.bmj.com/site/about/guidelines.xhtml

Abstract

Objectives

To undertake a process evaluation of an adherence support intervention for people with cystic fibrosis (PWCF), to assess its feasibility and acceptability.

Setting

Two UK Cystic Fibrosis (CF) units

Participants

Fourteen adult PWCF; 3 professionals delivering adherence support (‘interventionists’); 5 multi-disciplinary CF team members.

Interventions

Nebuliser with data recording and transfer capability, linked to a software platform, and strategies to support adherence to nebulised treatments facilitated by interventionists over five months (+/- one month).

Primary and secondary measures

Feasibility and acceptability of the intervention, assessed through semi-structured interviews, questionnaires, fidelity assessments, click analytics.

Results

Interventionists were complimentary about the intervention and training. Key barriers to intervention feasibility and acceptability were identified. Interventionists had difficulty finding clinic space and time in normal working hours to conduct review

For peer review only

For peer review only - http://bmjopen.bmj.com/site/about/guidelines.xhtml

visits. As a result, fewer than expected intervention visits were conducted and interviews indicated this may explain low adherence in some intervention arm participants. Adherence levels appeared to be >100% for some patients, due to inaccurate prescription data, particularly in patients with complex treatment regimens. Flatlines in adherence data at the start of the study were linked to device connectivity problems. Content and delivery quality fidelity were 100% and 60-92% respectively, indicating that interventionists needed to focus more on intervention 'active ingredients' during sessions.

Conclusions

The process evaluation led to 14 key changes to intervention procedures to overcome barriers to intervention success. With the identified changes, it is feasible and acceptable to support medication adherence with this intervention.

Registration: ISRCTN13076797; 7th June 2016.

Strengths and limitations of this study

- This is a detailed evaluation of the feasibility of an adherence support system for people with cystic fibrosis.
- The use of mixed methods provided indepth understanding of the processes involved in delivering the service, its value, and factors that might influence its use, implementation and success.
- This was a small, two-centre study.

For peer review only - http://bmjopen.bmj.com/site/about/guidelines.xhtml

Background

Cystic Fibrosis (CF) is a life-threatening, inherited condition affecting over 90,000 people worldwide, primarily of Northern European ancestry[1]. Median survival for people with CF (PWCF) is estimated at 31 years [2–6] with progressive lung function decline, caused by regular infection and damage to airways, being one of the main disease features [2].

Preventative medications preserve lung function and reduce exacerbations [7–13].Low adherence to these medications is problematic as this predicts exacerbations requiring intravenous antibiotics (IVAB)[14,15]. Exacerbations of this nature carry a risk of systemic side effects of both increased mortality[16,17], and cost of care [18–20]. In 2012, the total spend on CF in the UK was estimated to be £100 million, with £30 million spent on inhaled antibiotics and mucolytics[21]; the UK CF population received 171,907 days of IVAB with 93,455 days received in hospital, costing an estimated £27 million[22].

Self-reported adherence to inhaled therapies underestimates objectively-measured adherence, with rates of 80% and 36% recorded, respectively[23] and systematic data collection suggests objective adherence to be closer to 30%[24]. As a result, clinicians are currently unable to identify PWCF with low adherence, in order to provide additional support. Hitherto, the most objective surrogate measure of adherence has been the medicines possession ratio (MPR). However, based on the experience of a CF service in Leeds UK, MPR rates of 63%[25] considerably over-estimate adherence compared with nebuliser download data of 36%[26].

For peer review only - http://bmjopen.bmj.com/site/about/guidelines.xhtml

Treatment burden has long been recognised as a key barrier to medication adherence in CF[27], and reducing treatment burden is a key research priority for PWCF and clinicians, identified by the Cystic Fibrosis Foundation and the James Lind Alliance[28,29]. In response, a complex intervention was developed to support inhaled medication adherence in PWCF[30]. This article presents the results of a process evaluation that was undertaken alongside a pilot RCT, the objectives of which were to determine the feasibility of a full-scale RCT[30]. Here, we describe the resultant changes made to intervention procedures prior to that full-scale RCT[31]. The specific objectives of the process evaluation were:

1. To triangulate qualitative and quantitative data collected on intervention inputs, engagement, activities, and contextual factors, alongside immediate and intermediate outcomes recorded in the feasibility study, to understand and identify potential barriers to intervention implementation and success.
2. To document and use these findings to guide changes to intervention procedures, ahead of a future, full-scale RCT.

Methods

The wider feasibility study

The process evaluation forms one part of a wider pilot study, which also assessed the feasibility of RCT procedures and mechanisms of action (reported elsewhere[30,32]). The pilot RCT consisted of 33 intervention patients and 31 control patients. Three trained interventionists in two UK CF centres delivered the intervention to PWCF in the intervention arm and followed them up for 5 months, plus or minus one month.

Intervention description

The complex intervention to support adherence in CF was developed to enable PWCF to manage adherence to nebulised medication, with a view to shifting CF treatment

For peer review only - http://bmjopen.bmj.com/site/about/guidelines.xhtml

from rescue in hospital settings to prevention, managed in the community. The full intervention development process is described in a separate article[30].

The complex intervention consists of four key elements: the eTrack, CFHealthHub server, the CFHealthHub Apps and the manualised behavioural intervention. A logic model (Figure 1) was produced to reflect, in detail, constructs and processes by which the intervention was expected to function; this is in terms of inputs, engagement, activities, and outcomes. The logic model's hashed numbers (#1, #2, etc) provide a reference for linking intervention materials and processes to logic model constructs in Figure 1.

The eTrack (#4) (PARI Pharma GmbH, Starnberg, Germany) is a microchipped nebuliser, enabling real-time monitoring of adherence to nebulised medications. Timestamped records of medications administered via the eTrack are sent to a 2net Hub (Qualcomm, San Diego, USA; #5) which transmits data to PARI.

Real-time inhalation data is received by the CFHealthHub (CFHH) server infrastructure, stored securely and used for display in both a web-based interface and a mobile app (#6, see Figure 2). Each of these displays adherence data alongside tools to support behaviour change and educational content[33]. Educational modules within CFHH include: 'What is Cystic Fibrosis?'; 'What does my IV treatment do?'; 'I'm not convinced that my nebuliser treatment works'; 'What does my nebuliser treatment do and why should I take it?'; 'Why is it important that I do my nebuliser treatment every day?'; and, 'I have concerns about my nebuliser treatments'. The

For peer review only

For peer review only - http://bmjopen.bmj.com/site/about/guidelines.xhtml

nebuliser medication information displayed to the user in these sections are tailored to them based on a baseline assessment of motivation, so as not to overwhelm them.

Participants and their interventionists had access to adherence displays for monitoring (#13, #19, #20) and other CFHH content (#21- #26), such as education about treatments (#21) and problem solving in the face of adherence barriers (#26). Interventionists would use CFHH to facilitate delivery of manualised behavioural intervention sessions (#8, #17).

Interventionists (n =3) included a clinical psychologist, a physiotherapist and a social worker. They received specific training to deliver the manualised intervention sessions (#9). Training was delivered over two days, in face-to-face workshops. This was supplemented by online learning modules and a further four-week training schedule. Interventionists were assessed with online theory tests and in a competency assessment which examined intervention delivery within the first 5 sessions.

Sessions were delivered either face-to-face or remotely, on a one-to-one basis. All intervention arm participants received an initial intervention visit and a minimum of one additional review visit over the period of the study (#18). The content of sessions varied by participant reported motivation; sessions for those with low motivation were tailored to promote relationship / confidence building and to support the participant in the exploration of relevant CFHealthHub educational and information material (#21, #22). Relevant material could be added to the participant's personalised 'Toolkit'. Sessions conducted with participants displaying higher motivation would also involve supporting the participant to set personalised adherence goals (#23, #24), and to make

For peer review only - http://bmjopen.bmj.com/site/about/guidelines.xhtml

action plans (#25) and engage in problem-solving including making coping plans where relevant (#26).

Design

A mixed-methods approach was used for the process evaluation. Although this pragmatic case study[34,35] primarily works at the level of the programme, we also present a nested multiple-case design, with cases at the level of the PWCF, and two embedded units of analysis – interviews with intervention participants and trial data.

Data Sources

Quantitative and qualitative data sources were triangulated to address process evaluation objectives. These are described using hashed numbers to relate data sources to aspects of the logic model (Figure1) for which they contributed data.

Qualitative data included: verbal reports from project staff (#1. #2, #10, #16); semi-structured interviews with interventionists and participants in the intervention and control arms of the pilot RCT (#8, #9, #12, #13, #14, #15, #16, #17, #19, #20, #21); minutes of meetings (#3); emails (#4), website development reports (#6); and fidelity assessments (#17). Semi-structured interviews, conducted face-to-face, were digitally audio-recorded and transcribed verbatim. The median length of interviews was 30 minutes (range 11 to 87) for PWCF, 86 minutes (63 to 102) for interventionists and 62 minutes (51 to 66) for CF team members.

Quantitative data included: implementation log entries and data management reports (#3), questionnaire data derived from secondary clinical outcome measures described

For peer review only - http://bmjopen.bmj.com/site/about/guidelines.xhtml

in Table 1 (#7, #28, #29, #30, #31, #32, #33) an interventionist-completed structured questionnaire on interventionist confidence post-training (#9), structured interventionist fidelity assessments in which audio-recordings of intervention sessions were coded using a fidelity scoring system which assessed whether each component of the intervention was delivered and the quality of that delivery (#11, #17), CFHH click analytics (#13, #14, #15, #18, #20, #21, #22, #23, #24, #25, #26), session frequency and duration records (#15); and adherence data taken from CFHH (#35). Quantitative or descriptive data was collected for the 23 logic model constructs listed in this paragraph as part of the trial protocol, as described in Table 1.

Sampling

Participants were recruited for semi-structured interviews. Participants included intervention arm participants (n=14), interventionists (n=3, 0.8 WTE at each centre) and members of the wider, multi-disciplinary CF team (n=5). Participants were purposively sampled based on site, age, gender, deprivation index, objective and subjective adherence levels (service-users), or site and professional category (professionals). Interventionists were interviewed twice – at the beginning and end of the study – patients once. PWCF who consented to be approached for interview were contacted by letter or email and, subsequently, telephone or email depending on preference. Professionals were contacted directly by the study team.

Data Analysis

We conducted a Framework analysis of interview transcripts[36], within NVivo (QSR International) using multiple frameworks including the Theoretical Domains Framework[37], a process evaluation framework[38], and the logic model (Figure 1).

For peer review only - http://bmjopen.bmj.com/site/about/guidelines.xhtml

Using a modified triangulation protocol[39], we integrated qualitative and quantitative datasets at the programme- and the case-level[40]. We used a joint display table[41] to summarise data sets for 35 logic model constructs in the Inputs (n=12), Engagement (n=6), Activities (n=7), Immediate outcomes (n=6) and Intermediate outcomes (n=2) columns (Figure 1). The fit of data integration was categorised as: 'confirmation' (quantitative and qualitative data provided similar findings); 'expansion' (the datasets addressed different or complementary aspects of the phenomenon); or, 'discordance' (the datasets were contradictory)[42]. We described similar and unique contributions , made by the two data sets, to the research question [39].

In the 14 intervention participants, for whom both qualitative and quantitative process data was available, we produced case-profiles[43], triangulating qualitative data with individual-participant adherence run charts[44] (Additional File 01) and other quantitative process data (see Additional File 02 – Study protocol, pp29-31). We worked abductively, moving between behaviour change theories[45,46] and contextual observations, agreeing plausible hypotheses to explain patterns which could be tested in future work [47–50].

We produced a case-ordered descriptive matrix[51], with cases ranked by average adherence during the last month of the study, to understand how processes and outcomes were mediated by local and individual conditions. Adherence levels of >80% were assessed as high; 50-80% moderate; <50% low [14,52]. We theorised that high life chaos, as measured by the Confusion, Hubbub and Order Scale

For peer review only - http://bmjopen.bmj.com/site/about/guidelines.xhtml

(CHAOS)[53] and low motivation would be associated with low adherence. We used four measures to understand motivation: (1) a single item, scored on a 1-7 Likert scale – "I want to do all of my nebuliser treatment" (motivation); (2) a single item, scored on a 1-7 Likert scale, which asked, "I am confident I can do all of my nebuliser treatments" ('confidence'); (3) the necessities and, (4) concerns five-point subscales of the Beliefs about Medicines Questionnaire nebuliser-specific (BMQ) instrument [54]. Interventionists assessed the participant's motivation to increase adherence on a one to seven scale after discussion with the patient; adequate motivation was necessary before participants could make action plans and do problem solving activities.

Approach taken to modifying the intervention

Modifications to the intervention fell into three categories: the software platform; other Information Technology (IT) infrastructure; and the manual and training. Identified problems and solutions were tabulated following a modified approach of that taken by Bugge [31]. Digital platform development was reviewed regularly using the "Must have, Should have, Could have, and Won't have but would like" (MoSCoW)[55], often used in agile software development [56,57].

Patient and Public Involvement

Recruitment for the Patient and Public Involvement (PPI) Group was achieved by advertising within CF units and on the People in Research website, as well as via group members themselves. Cross-infection between PWCF[58] was prevented by arranging meetings via teleconference. The PPI group gave feedback on intervention data-sharing policies, usability and presentation of the website/user-guide. In addition,

For peer review only

For peer review only - http://bmjopen.bmj.com/site/about/guidelines.xhtml

the PPI group piloted the participant information materials and one individual gave feedback on the trial protocol and interview guides (Additional File 02).

Ethical approval

The study received approval from London Brent Research Ethics Committee (16/LO/0356). The funder was not involved in the trial design, patient recruitment, data collection, analysis, interpretation, or presentation, writing or editing of the report, or the decision to submit for publication. The corresponding author had full access to all the data in the study and had final responsibility for the decision to submit for publication.

Results

In what follows, we address contextual factors that affected implementation and participant responses, then follow the columns (inputs, engagement, activities, immediate and intermediate outcomes) of the logic model. Additional File 03 (Tables A-G) summarises quantitative process outcomes for 14 case study participants, ranked by objective adherence at the end of the trial. Hashed numbers (#1, #2, etc) indicate cross references to the logic model (Figure 1) and supporting evidence in Additional File 04, which summarises data triangulation at the level of individual logic model constructs. Both qualitative and quantitative data were available for 13/34 logic model constructs, providing confirmation of (n=2) or expansion on (n=11) inferences drawn from quantitative data. A case-ordered descriptive matrix based on logic model columns (Additional File 05) and run charts annotated with key events (Additional File 01) provides an integrated analysis at the level of the participant for fourteen 'case studies', cross referenced by participant numbers (R02/52, R01/54, etc).

For peer review only - http://bmjopen.bmj.com/site/about/guidelines.xhtml

For peer review only

Contextual factors affecting implementation and participant responses

The key factor affecting implementation was the mixed economy of CF drug delivery systems: the e-Flow (PARI Pharma GmbH, Starnberg, Germany); the iNeb (Philips Healthcare, Eindhoven, Netherlands); and a number of dry powder delivery systems. The e-Flow is the only device able to deliver all the wet nebulised drugs that are used in CF care. The e-Track we used in this trial was a version of the e-Flow developed to transfer time- and date-stamped data. Most patients at site R01 used e-Flows; switching consenting participants over to the e-Track was generally unproblematic. The e-Flow's competitor, the iNeb, cannot deliver aztreonam and requires double chamber filling to deliver tobramycin, so it is not suitable for all patients. The data transfer version of the iNeb, the BiNeb, is a prototype for which limited numbers are available. We were unable to secure approval to integrate the BiNeb into CFHH in time to incorporate it into this study. At site R02 where iNebs were commonly used, those who were familiar with and liked the iNeb were less keen to swap to an alternative device; some who swapped to the e-Track, later wanted to move back to the iNeb. A minority of patients use dry powder delivery systems, none of which have data transfer versions. We were unsuccessful in engaging any of the companies producing dry powders in time to get dry powder systems integrated into CFHH, meaning that dry powder users could not be recruited to this feasibility study. Making nebulisers with data recording and transfer capability available within hospitals following local delivery took prolonged engagement with medical engineering departments to obtain local safety approvals. For more than one participant, the strength of their mobile data signal affected 2net Hub connectivity with the central server (Implementation log, 19 Oct 16).

For peer review only - http://bmjopen.bmj.com/site/about/guidelines.xhtml

Through meetings with site staff, the team identified a range of human factors that also affected implementation, in particular: the availability of out-patient rooms; the need to clean rooms after each consultation for cross-infection control purposes; and, the expectation that, during hospital visits, outpatients will see the whole each member of the multidisciplinary team separately. The struggle for clinic space and patient convenience resulted in more home visits than anticipated for consent and review meetings, informed by local lone-working policies. Reorganisation of one CF Centre, involving the transfer of patients from the care of one local hospital to another, had created discontent among some patients involved in the trial.

Inputs

The study chief investigator reported introducing local site investigators, centre directors and Multi-Disciplinary Teams (MDTs) to CFHH **(#1)**. Through case reports, he conveyed that relying on FEV_1, symptoms and BMI for CF management alone is inadequate and that objective adherence data could help overcome the 'lamppost syndrome'[59], also known as the 'streetlight effect'[60,61] or 'drunkard's search'(page 11[62]) – a type of availability bias[63]. The chief investigator reported feeling that site investigators at both centres were fully bought in, but that one clinician (not an investigator) believed that the disparities between subjective and objective adherence[23] were overstated **(#2)**.

Interventionists entered prescription data into CFHH based on patient records and self-reported treatment regimen **(#3)**. Occasionally, interventionists were slow to make monthly prescription checks when prompted by system alerts, resulting in

For peer review only - http://bmjopen.bmj.com/site/about/guidelines.xhtml

apparent adherence levels of over 100%, traced to the use of alternating treatment regimens[64] (Implementation Log, 01 Dec 16, TMG minutes 10 Jan 17). Nebulisers with data recording and transfer capability **(#4)**, 2net Hubs **(#5)**, the CFHH website and mobile application **(#6)**, were made available (emails to project manager 20 May 16, 23 Jun 16). The Capability Opportunity Motivation -Beliefs about Medicines Questionnaire (COM-BMQ – see Additional File 02)[54] questionnaire data **(#7)** was collected in CFHH (Additional File 06, Tables 1-22, Figures 1-9).

Interventionists were complimentary about the intervention manual **(#8)** and highly satisfied with training, but suggested that future courses involved a case study approach, following a patient through the intervention to illustrate its different aspects **(#9)** (Additional File 04). A member of the research team (MH) acted as an intervention mentor to interventionists **(#10)**. Interviews (SD) and observations (MH, HC) identified differences in the way site investigators interacted with interventionists, with one giving more intensive practical support, through weekly meetings and problem-solving (not prescribed by the intervention), than the other. Fidelity data was collected on all three interventionists and the fidelity assessment instrument was modified before use in the full RCT **(#11)**. During interviews, interventionists were enthusiastic about intervention processes **(#12)**. As sites struggled to find space or time for consent / intervention encounters in clinic, the study team requested an increase in the number of home visits (Implementation log 19 Oct 2016). As a result of initial problems in contacting participants and the need for flexibility in arranging meetings out of usual clinic hours, the study team requested flexible working in which the team worked 12:00-20:00 two days a week (interviews & TMG minutes 29 Nov 2016).

For peer review only - http://bmjopen.bmj.com/site/about/guidelines.xhtml

Engagement

Interviews and click analytics showed that MDT members did not access adherence data **(#13)**, aside from in the form of bar charts brought to MDT meetings by interventionists. It is important to note that extending the use of CFHH to the MDT was not an objective of the trial and no training was given in this regard. Click analytics showed that interventionists tracked adherence **(#14)**. Of 14 case study participants, three did not contribute complete adherence data: R02/42 and R02/02 withdrew, while R02/03 was lost to follow-up. In other participants, flatlines in adherence data caused concern (Additional File 01). Flatlines at the beginning of the study (e.g. R01/39, R01/48) indicated technical problems with pairing nebulisers and hubs. Flatlines at the end of the study period (e.g. R01/42, R01/44, R02/12) were confirmed as the genuine recording of non-adherence through the use of adherence data beyond the end of the study period, interview data, self-report subjective adherence and the MAD-3 (Additional File 03 – Table f).

Click analytics showed the median number of participant CFHH sessions was three **(#15)** (Additional File 03 – Table c). Of those with low usage, initial technical problems (R01/02, R01/48) and initial lack of availability of a mobile application (#6) were potential contributing factors. Some case study participants showed moderate (R02/52, R01/54 and R01/40: 9-13 sessions) or high use (R02/12 and R01/42: >40 sessions). Push notifications - user-defined messages from the server which give participants congratulations or reminders about adherence behaviour – were not available in the pilot trial **(#16)**.

For peer review only - http://bmjopen.bmj.com/site/about/guidelines.xhtml

Based on fidelity assessment of intervention session recordings, the *content* fidelity of face-to-face interactions, was excellent (100%) – with all aspects delivered as per the manual **(#17)**. Delivery *quality* fidelity was more variable (60-92%). The generation of goals and action plans was sometimes too directive rather than negotiated and supportive. Interviews demonstrated that assessing the true level of motivation to adhere to treatment was challenging; sometimes those with insufficient intrinsic motivation (e.g. R01/48, R01/54 and R02/03) were assessed as having sufficient motivation and inappropriately tasked with setting and reviewing goals, making action plans and problem solving (see below #23-26). These individuals were variably motivated by wanting to prove themselves to MDT members, who had doubted their adherence (R01/49 and R01/54, Additional File 05), or by helping the research:

> *"I made that special effort 'cause I was taking part in this trial… I didn't see how it was going to make me better" (R01/48).*

Interaction with these individuals should have been confined to relationship-building and trust-building. Fidelity assessment of recordings identified that, in interactions with the adequately motivated, the focus was not always on the most active ingredients – goal-setting, action planning (habit formation) and problem-solving/coping planning. Participant run charts (Additional File 01) revealed a disparity in whether and when review visits happened **(#18)**.

Activities

In interviews, CF team clinicians (as distinct from the interventionists) confirmed they were not monitoring adherence as part of usual care **(#19)**. Participant R01/02

For peer review only - http://bmjopen.bmj.com/site/about/guidelines.xhtml

complained that the research focus on adherence was "*parallel rather than integrated*" with mainstream clinical management. However, the intervention was designed to be interventionist delivered allowing individual randomisation in a system without contamination of controls rather than an intervention aimed at achieving system change which would have required a cluster trial design. Participants' clicks (median 11) on the CFHH "How am I doing?" (run charts) page sometimes related to a limited number of sessions. In interviews, one moderately frequent user (R01/54) only accessed this page to check their data was uploading. Other moderate/frequent users described this page as important for adherence self- monitoring **(#20)**, even when their grasp of their own adherence was poor (R01/49).

In interviews with participants, for tailored education about treatment **(#21)**, participants accessed particular education pages for specific issues, such as nebuliser malfunction, which was viewed as, "more down to earth" than technical manuals. In particular a video about the treatment action of Dornase alfa, was often praised, as a means of educating others about CF; 'Talking heads' videos (in these videos people with CF described strategies for successful nebuliser use) **(#22)** divided opinion: for some, the opportunity for social comparison[65] provided relief and reassurance; those who were less appreciative were those who found comparisons with people healthier than themselves could make them feel as though they were not doing well and comparisons with those less healthy could make them fearful of the future.

Other activities (#23 to #26 on the logic model) required participants to have adequate levels of motivation. Interventionists classified all but one case study participant (R01/44) as having adequate motivation (Additional File 03, Table b) and therefore

For peer review only - http://bmjopen.bmj.com/site/about/guidelines.xhtml

eligible for further tailored intervention. But, as detailed above (see #17 in the engagement section), this was sometimes based on inadequate discussion with the participants. In interviews, participants generally reported setting goals **(#23)**, but fidelity assessment showed that goals were sometimes formulated by interventionists rather than by participants (see #17). The mean number of review sessions **(#24)** over five months was 1 (Additional File 03 – Table e); this was fewer than intended, likely reflecting a failure of the study team to set appropriate expectations and a lack of time created by the high pace of recruitment (problem log entries: 31-Jan-17; 13-Feb-17). Two individuals (R01/39 and R01/40) received their first face-to-face session with an interventionist over halfway through the study period (Additional File 01). CFHH action plan **(#25)**, problem solving and coping plan **(#26)** pages were accessed a median of two, three and one times, respectively (Additional File 03 – Table e). Interviews data suggests action / coping plans were completed during intervention visits but not accessed by participants otherwise. In interviews, some participants said they were reassured by the presence of, and sometimes reported insights from, problem-solving modules, such as what to do when going on holiday. However, the use of action plans was disliked by some participants who found writing down the action plans like "*going back to school*". This dislike at least partly reflected the generation of action and coping plans by interventionists rather than by the participants themselves (see #17).

Immediate outcomes

The pilot was not designed to disseminate the intervention across the centre and with minimal monitoring by professionals within the wider CF team (see #19) routine medical care was not informed by adherence **(#27)**. Unsurprisingly, given the lower

For peer review only - http://bmjopen.bmj.com/site/about/guidelines.xhtml

than expected face-to-face contact **(#18, #24)**, intervention arm group averages for immediate (process) outcomes **(#28-33)** changed little over five months, with the exception that there was a mean reduction of 1.84 (SD 3.44) barriers to adherence per person **(#33)**, which could be the outcome of problem solving and education about treatment processes (Additional file 03 – Table f). Frequent use of CFHH and self-monitoring in particular (see above, #20) did not necessarily mean that self-reported subjective adherence and electronically-captured objective adherence were well aligned **(#28)** (Additional file 03 – Tables f and g). A *post hoc* paired comparison of subjective and objective adherence at 5 (+/-1) months (Figure 3) suggests that higher adherers were more uniformly accurate in their understanding of their own adherence, whereas low adherers could be overly optimistic.

Intermediate outcomes

Item **#34** of the logic model, treatment optimisation, is defined by NICE as, "a person-centred approach to safe and effective medicines use" to ensure best outcomes[66]. Treatment optimisation is a service-level objective, which was beyond the scope of our patient-focused intervention but is the subject of related ongoing research (see Discussion). During interviews, RCT participants in the intervention arm described behaviours that would affect treatment optimisation, for instance taking holidays from their treatment. Levels of CF treatment adherence **(#35)** were 10% (95% CI: -5.2 to 25.2) higher in the intervention arm (Additional File 06). We developed a number of theories about why some intervention patients did or did not increase their adherence **(#35)** during the analysis. In some cases the run charts illustrated, in line with Control Theory, the goal-directed nature of behaviour and how it is regulated by feedback control processes[67]. For example, R01/39 and R01/49

For peer review only

For peer review only - http://bmjopen.bmj.com/site/about/guidelines.xhtml

seemed to show improvement shortly before planned face-to-face visits from interventionists (Additional File 01). R01/39, who seemed intrinsically motivated when interviewed, sustained improvement in adherence beyond the trial period through what they described as positive interaction with the interventionist. Others, who seemed more extrinsically motivated in interviews (R01/49, R01/54, R01/48: see #17), did not sustain adherence, with charts suggesting an effortful, ‘all-or-nothing’ pattern. At baseline, R02/07 had no well-established routine (CHAOS score of 10: Additional File 01), implying substantial self-regulatory effort to achieve higher adherence. In their interview, this participant reported finding habit formation parts, such as goal-setting, helpful which may have enabled him to maintain high adherence with reduced effort, as measured by increased habit and reduced life chaos and barriers (change scores -5 and -3 respectively: Additional File 03 - Table f). Finally, it is important to understand that individual-level adherence can be unstable over time (Additional File 01, see especially, R01/54, R01/48) highlighting the problem of assessing adherence as a ‘snapshot’ in a pre-/post-test analysis, rather than in a continuous assessment over time.

Several participants with low baseline adherence appeared to have responded well to the intervention. R01/40 had high motivation (Additional File 03 – Table b; Additional File 01), possibly due to the salience of a recent hospitalisation for IVAB treatment of an exacerbation. Click analytic data showed high engagement, with independent access of the website and use of problem-solving tools. However in other patients, case study run charts (Additional File 01) showed that measuring change in average objective adherence between baseline and five months sometimes masked periods of success in between (e.g. R01/02, and R02/12). Without looking at

For peer review only

adherence graphs, and only measuring objective adherence at baseline and five months, this would have been missed (see Discussion). Interview data offered some reasons for improved adherence. While R01/49 had not made an action plan and their subjective adherence was optimistic (Additional file 03 – Table f), their objective adherence increased from low to moderate over the trial period (Additional File 01); their motivation also increased and self-reported barriers decreased (Additional file 03 – Table f), potentially through their high use of problem-solving modules and self-monitoring (Additional File 03, Table d). R01/02's run chart also showed a period of improvement, ending after the last review visit (Additional File 01); nonetheless, reduced life chaos (Additional file 03 – Table f) and interview data suggested an established routine and reduced barriers associated with intensive face-to-face therapist interaction and action/coping plans (Additional File 03, Table d). The tailing off of adherence after the end of the trial in some case study participants may indicate that adherence remained effortful or participation in the trial was motivated by altruism not help-seeking (see quotation from R01/48, above).

Modifications to the intervention

Additional file 07 documents 14 technical changes that will be made for the full-scale RCT, based on the process evaluation findings, to CFHH (n=5), IT infrastructure (n=1) and to the interventionist training, manual and procedures (n=8). To prevent adherence data flatlines, nebulisers **(#4)** and 2net Hubs **(#5)** will be paired at the factory. Three changes to CFHH **(#6)** will make it easier for interventionists to view/edit prescription data and to handle alternating treatment regimens **(#3)**. Other changes to CFHH will include making graphs more easily interpretable and, based on interview data and PPI feedback, adding descriptions to videos. Changes to the

For peer review only - http://bmjopen.bmj.com/site/about/guidelines.xhtml

interventionist manual **(#8)** will increase the emphasis on 'active ingredients', introduce intervention triggers for reduced adherence or exacerbations and introduce new habit formation sessions. The need for increased numbers of protocolised intervention review sessions arose because, in the feasibility study, a focus on RCT recruitment targets gave interventionists inadequate time to deliver review visits **(#18, #24)**, critical for updating personalised action plans **(#25)** and updating coping plans **(#26)**. Training **(#9)** in the full-scale trial will be delivered as an intensive one-week course, with more explicit focus on intervention fidelity, supported by new case study data and role plays to ensure baseline competency **(#17)**.

Discussion

The process evaluation identified elements of the intervention which could be improved and 14 changes were documented. The complex intervention was developed using mixed methods research with an inter-disciplinary, person-centred and iterative approach[68–74]. The mere usage of a digital behaviour change intervention may not indicate engagement or lead to desired outcomes[68,73,75–78]; there is no simple dose-response relationship[79]. In fact, for those with low motivation and low confidence, evidence of non-adherence can be threatening[80,81]. With different baseline motivation and life chaos, a population-level definition of "effective engagement"[70] may be infeasible, but contextual and motivational data may still explain patterns observed in run charts[82]. What may matter more than defining engagement is the correct assessment and tailoring of management to different psychosocial barriers[69,83–91]. Our study suggests that digital systems cannot replace, only complement, face-to-face interaction between health professionals and patients[92–95], potentially creating a sense of 'accountability' consistent with

For peer review only - http://bmjopen.bmj.com/site/about/guidelines.xhtml

control theory[46,96]. However, it is important to recognise that in the absence of objective adherence data clinicians and patients will find it difficult to even begin to engage with behaviour change.

Chronic disease self-management is a complex and multi-factorial problem and, we were unable to cover all of the analyses that many would consider relevant. For instance, although the intervention is meant to increase health literacy through education, we cannot rule out that baseline socio-economic status, known to affect health literacy, outcomes and self-management[97–99], was not a factor. Another limitation of this study is that we interviewed just over only one quarter of the pilot trial sample. Given a relatively homogeneous population, narrow, exploratory study aims and the use of established theory, fourteen interviews should be adequate to discern common perceptions and experiences[100,101]. In the full-scale evaluation of this intervention (see below), the process evaluation will involve a user acceptability survey of ~250 intervention users from 19 centres and face-to-face interviews with over 50 intervention users, interventionists and clinicians. As in many other process evaluations, we will use maximum variation sampling on sociodemographic characteristics and baseline adherence, alongside triangulation, to minimise the risk of bias[102]. Additionally, readers should be aware that small scale feasibility work does not generalise in every regard when scaled up in larger scale studies[103,104]. Finally, early health economic modelling of the cost-effectiveness[105], was not updated as part of this feasibility work, but will be revisited in 2021 as part of the full-scale evaluation.

For peer review only - http://bmjopen.bmj.com/site/about/guidelines.xhtml

Our use of objective adherence measurement overcomes the limitations of previous studies[106] and confirms that subjective and objective adherence are poorly aligned[23]. This process evaluation has succeeded in demonstrating that delivery of this intervention is possible in busy clinical settings; participant uptake was high and, with further development on the basis of these findings, the process of gathering objective adherence data and implementing it alongside a behavioural intervention is both possible and effective.

Given the known difficulties with nebuliser use among PWCF, interventions that can make it less effortful are important[107]. In particular, healthy behaviours are better predicted by a patient's level of automatic behavioural repetition than their beliefs or experiences, meaning a focus on increasing habit strength is critical for chronic disease self-management[108]. Through delivery of intervention components designed to promote habit formation, we intend to reduce effort with the CFHH intervention. We are limited in drawing conclusions as to the impact of habit formation components of the intervention from this analysis; this is mostly due to the limited time constraints of the feasibility study leaving insufficient opportunity for habit formation[109]. However, there was some indication that habit components were useful and we have elsewhere demonstrated the importance of habit in high adherence [110,111]. It has also been indicated that adherence interventions focusing on habit formation are the most effective[112].

Successful habit formation will reduce burden by making sustained self-care automatic. The CFHH intervention aims to deliver the fall in burden highlighted by the Lind alliance prioritisation exercise as the most important goal of CF research.

For peer review only

For peer review only - http://bmjopen.bmj.com/site/about/guidelines.xhtml

To date, there is little previous research showing the effects of giving patients access to their data, with respect to health outcomes and cost-effectiveness. Amidst the evidence that does exist, the research is generally poor and lacks information about context and implementation[113,114]. Following modifications made to our complex intervention, the full scale RCT across 19 UK centres (ISRCTN55504164) will provide high quality evidence, indicating the impact of adherence data on sustained self-care. The full-scale RCT will include a further process evaluation and health-economic modelling. Furthermore, the CFHealthHub Data Observatory (ISRCTN14464661) following on from the RCT will address the issue of how to embed the use of adherence data in routine practice for healthcare professionals[115–119] . The sites involved in the reported pilot study have now transitioned into the Data Observatory, eventually to be joined by sites involved in the full-scale RCT. Data collected in the data observatory quality improvement project will be used in the development of generalisable theory and practical guidance about the collaborative use of adherence data [120–122], with a focus on optimising the use of health care resources and improving patient care [66,123]. The Observatory will act as a platform for efficient trials[124,125], providing an opportunity to share processes and improvement activities to enable participating CF clinical research teams to meet the demands of future research [126].

Conclusions

We have developed a theory-based complex intervention to help PWCF adhere to their medication and form habits of sustained self-care. The process evaluation identified potential sources of intervention failure and modifications have been made accordingly. With improved intervention processes, it is feasible and acceptable to

For peer review only - http://bmjopen.bmj.com/site/about/guidelines.xhtml

support sustained self-care via medication adherence through the application of behaviour change theory delivered through digital and human components.

Declarations

List of abbreviations

CF – Cystic Fibrosis
CFHH – CFHealthHub complex intervention
CFQ-R – Cystic Fibrosis Questionnaire-Revised
CHAOS - Confusion, Hubbub, and Order Scale
COM- BMQ – The Capability Opportunity Motivation Behaviour Beliefs Questionnaire
EQ-5D-5L – EuroQol generic health status measure
FEV1 - Forced expiratory volume in 1 second
GAD-7 – General Anxiety Disorder 7-item
IT – Information Technology
IVAB - Intravenous Antibiotics
MAD-3 Medication Adherence Data
MDT – Multi-Disciplinary Team
PAM-13 - Patient Activation Measure
PHQ-8 – Patient Health Questionnaire depression scale
PWCF – People With Cystic Fibrosis
RCT – Randomised Controlled Trial
SRBAI – Self-Report Behavioural Automaticity Index

Ethics, consent and permissions

Written informed consent was obtained prior to participation.

Ethical approval

The study received ethical approval from the London Brent Research Ethics Committee (16/LO/0356).

For peer review only

Consent for publication

In the consent form, participants signed a statement to confirm consent for publication of anonymised quotes, in reports, conference presentations and journal publications.

Availability of data and materials

Requests for further data not available in this publication can be directed at Sheffield Clinical Trials Research Unit. Email: ctru@sheffield.ac.uk Tel: 0114 222 0866

Competing interests

Martin J Wildman received funding from Zambon and support from Philips Respironics for the early intervention development work. This has not had any direct influence on the feasibility study reported here. In addition, Martin Wildman has

For peer review only - http://bmjopen.bmj.com/site/about/guidelines.xhtml

worked with Pari to carry out studies using the e-track. This has not had any direct influence on the feasibility study reported here. The University of Manchester software team received funding from Pari to create a medication reporting component within the CFHealthHub software. This has not had any direct influence on the feasibility study reported here.

The other authors declare that they have no competing interests.

Funding

This report presents independent research funded by the NIHR under its Grants for Applied Research Programme (Grant Reference Number RP-PG-1212-20015). The views and opinions expressed by authors in this publication are those of the authors and do not necessarily reflect those of the NHS, the NIHR, Medical Research Council (MRC), Central Commissioning Facility (CCF), the Programme Grants for Applied Research Programme, or the Department of Health and Social Care.

Authors' contributions

Daniel Hind (Assistant Director, CTRU), Sarah Drabble (Research Associate) and Laura Mandefield (Statistician), together produced the first draft of the report.

The following conceived of or designed the work: Martin Wildman (Consultant Respiratory Physician), Alicia O'Cathain (Professor of Health Services Research), Stephen Walters (Professor of Medical Statistics and Clinical Trials), Madelynne Arden (Professor of Health Psychology), Marlene Hutchings (Physiotherapist), Judy Bradley (Professor of Physiotherapy), John Ainsworth (Professor of Health Informatics), Daniel Beever (Patient and Public Involvement Representative) and Daniel Hind.

The following were involved in the acquisition of data for the work: Julia Nightingale (Consultant in Respiratory Medicine), Mark Allenby (Consultant in Respiratory Medicine), Jane Dewar (Consultant in Respiratory Medicine), Sarah Drabble, Madelynne Arden, Chin Maguire, Hannah Cantrill, Alexander Scott (Research Associate), Sam Keating (Research Associate), Marlene Hutchings and Pauline Whelan (mHealth Applications Manager).

The following were involved in the analysis of data: Daniel Hind, Sarah Drabble, Alicia O'Cathain, Daniel Beever, Alexander Scott, Sam Keating, Madelynne Arden, Laura Mandefield, Simon Waterhouse (Data Manager).

Daniel Hind, Sarah Drabble, Madelynne Arden, Laura Mandefield, Simon Waterhouse, Chin Maguire, Hannah Cantrill, Louisa Robinson¸ Marlene Hutchings, Judy Bradley, Julia Nightingale, Mark Allenby, Jane Dewar, Daniel Beever, Stephen Walters, Alicia O'Cathain, Martin J Wildman, were involved in the interpretation of data for the work.

All authors were involved in the final approval of the version to be published.

All authors agree to be accountable for all aspects of the work in ensuring that questions related to the accuracy or integrity of any part of the work are appropriately investigated and resolved.

For peer review only

For peer review only - http://bmjopen.bmj.com/site/about/guidelines.xhtml

Acknowledgements

We would like to thank the trial participants who offered us their time. We gratefully acknowledge the clerical and other input of Helen Wakefield, Heather Dakin, Katie Shore and Louise Turner, trial support officers.

We would also like to thank Claire Oliver, Fiona Haynes and Lisa Evans for their commitment and hard work in delivering the intervention and supporting the study.

Professor Walters is a National Institute for Health Research (NIHR) Senior Investigator. The views expressed in this article are those of the author(s) and not necessarily those of the NHS, the NIHR, or the Department of Health and Social Care.

For peer review only

For peer review only - http://bmjopen.bmj.com/site/about/guidelines.xhtml

References

1 Bell SC, Mall MA, Gutierrez H, *et al.* The future of cystic fibrosis care: a global perspective. *Lancet Respir Med* 2020;**8**:65–124. doi:10.1016/S2213-2600(19)30337-6

2 Jeffery A, Charman S, Cosgriff R, *et al.* *UK Cystic Fibrosis Registry Annual Data Report 2016*. London: : Cystic Fibrosis Trust 2017.

3 Cystic Fibrosis Foundation. *Annual Data Report 2016 Cystic Fibrosis Foundation Patient Registry*. Bethesda, Maryland: : Cystic Fibrosis Foundation Patient 2017. https://www.cff.org/Research/Researcher-Resources/Patient-Registry/2016-Patient-Registry-Annual-Data-Report.pdf

4 European Cystic Fibrosis Society. *ECFS Patient Registry Annual Data Report*. Karup, Denmark: : European Cystic Fibrosis Society 2013.

5 Ahern S, Sims G, Tacey M, *et al. The Australian Cystic Fibrosis Data Registry Annual Report, 2015. Monash University, Department of Epidemiology and Preventive Medicine, October 2017, Report No 18.* Melbourne, Victoria: : Australian Cystic Fibrosis Data Registry 2017.

6 Cystic Fibrosis Canada. *The Canadian CF Registry 2016 Annual Data Report*. Toronto: : Cystic Fibrosis Canada 2017. http://www.cysticfibrosis.ca/uploads/2016 Registry Annual Data Report.pdf

7 Southern KW, Barker PM, Solis-Moya A, *et al.* Macrolide antibiotics for cystic fibrosis. In: Southern KW, ed. *Cochrane Database of Systematic Reviews*. Chichester, UK: : John Wiley & Sons, Ltd 2011. doi:10.1002/14651858.CD002203.pub3

8 Ramsey BW, Davies J, McElvaney NG, *et al.* A CFTR Potentiator in Patients with Cystic Fibrosis and the *G551D* Mutation. *N Engl J Med* 2011;**365**:1663–72. doi:10.1056/NEJMoa1105185

For peer review only

For peer review only - http://bmjopen.bmj.com/site/about/guidelines.xhtml

9 McCoy KS, Quittner AL, Oermann CM, *et al.* Inhaled aztreonam lysine for chronic airway Pseudomonas aeruginosa in cystic fibrosis. *Am J Respir Crit Care Med* 2008;**178**:921–8.

10 Ryan G, Mukhopadhyay S, Singh M. Nebulised anti-pseudomonal antibiotics for cystic fibrosis. In: Ryan G, ed. *Cochrane Database of Systematic Reviews*. Chichester, UK: : John Wiley & Sons, Ltd 2003. doi:10.1002/14651858.CD001021

11 Jones AP, Wallis C. Dornase alfa for cystic fibrosis. In: Jones AP, ed. *Cochrane Database of Systematic Reviews*. Chichester, UK: : John Wiley & Sons, Ltd 2010. doi:10.1002/14651858.CD001127.pub2

12 Wark P, McDonald VM. Nebulised hypertonic saline for cystic fibrosis. In: Wark P, ed. *Cochrane Database of Systematic Reviews*. Chichester, UK: : John Wiley & Sons, Ltd 2009. doi:10.1002/14651858.CD001506.pub3

13 Ryan G, Singh M, Dwan K. Inhaled antibiotics for long-term therapy in cystic fibrosis. *Cochrane database Syst Rev* 2011;:CD001021. doi:10.1002/14651858.CD001021.pub2

14 Eakin MN, Bilderback A, Boyle MP, *et al.* Longitudinal association between medication adherence and lung health in people with cystic fibrosis. *J Cyst Fibros* 2011;**10**:258. doi:10.1016/j.jcf.2011.03.005

15 Briesacher BA, Quittner AL, Saiman L, *et al.* Adherence with tobramycin inhaled solution and health care utilization. *BMC Pulm Med* 2011;**11**:5. doi:10.1186/1471-2466-11-5

16 Smyth A, Lewis S, Bertenshaw C, *et al.* Case-control study of acute renal failure in patients with cystic fibrosis in the UK. *Thorax* 2008;**63**:532–5. doi:10.1136/thx.2007.088757

For peer review only

17 Mayer-Hamblett N, Rosenfeld M, Emerson J, *et al.* Developing Cystic Fibrosis Lung Transplant Referral Criteria Using Predictors of 2-Year Mortality. *Am J Respir Crit Care Med* 2002;**166**:1550–5. doi:10.1164/rccm.200202-087OC

18 Shiers D, Rafi I, Cooper S, *et al.* 2014 update (with acknowledgement to the late Helen Lester for her contribution to the original 2012 version) Positive Cardiometabolic Health Resource: an intervention framework for patients with psychosis and schizophrenia. *R Coll Psychiatr* 2014;:London.

19 Quittner AL, Zhang J, Marynchenko M, *et al.* Pulmonary medication adherence and health-care use in cystic fibrosis. *Chest* 2014;**146**:142–51. doi:10.1378/chest.13-1926

20 Narayanan S, Mainz JG, Gala S, *et al.* Adherence to therapies in cystic fibrosis: a targeted literature review. *Expert Rev Respir Med* 2017;**11**:129–45. doi:10.1080/17476348.2017.1280399

21 McManus P. Lead Pharmacist - specialised services (South Yorkshire and Bassetlaw) NHS England.

22 McNeil S. Personal communication from Stephanie McNeil, Chief Statistician of UK CF Registry.

23 Daniels T, Goodacre L, Sutton C, *et al.* Accurate assessment of adherence: self-report and clinician report vs electronic monitoring of nebulizers. *Chest* 2011;**140**:425–32. doi:10.1378/chest.09-3074

24 Hoo Z, Curley R, Walters S, *et al.* Exploring the implications of different approaches to estimate centre-level adherence using objective adherence data in an adult cystic fibrosis centre - a retrospective observational study. *J Cyst Fibros* 2020;**19**:162–7. doi:10.1016/j.jcf.2019.10.008

25 White H, Shaw N, Denman S, *et al.* Variation in lung function as a marker of

For peer review only

adherence to oral and inhaled medication in cystic fibrosis. *Eur Respir J* 2017;**49**:1600987. doi:10.1183/13993003.00987-2016

26 Daniels T, Goodacre L, Sutton C, *et al.* Accurate Assessment of Adherence. *Chest* 2011;**140**:425–32. doi:10.1378/chest.09-3074

27 Sawicki GS, Sellers DE, Robinson WM. High treatment burden in adults with cystic fibrosis: Challenges to disease self-management. *J Cyst Fibros* 2009;**8**:91–6. doi:10.1016/j.jcf.2008.09.007

28 Horne RW, Barber N, Elliot R, *et al.* Concordance, adherance and compliance in medicine taking: A conceptual map and research priorities. National Co-ordinating Centre for NHS Service Delivery and Organisational R&D (NCCSDO). 2005.

29 James Lind Alliance. Priority Setting Partnership: Cystic Fibrosis. 2017.http://www.jla.nihr.ac.uk/priority-setting-partnerships/cystic-fibrosis/ (accessed 2 Jun 2017).

30 Hind D, Drabble SJ, Arden MA, *et al.* Supporting medication adherence for adults with cystic fibrosis: a randomised feasibility study. *BMC Pulm Med* 2019;**19**:77. doi:10.1186/s12890-019-0834-6

31 Bugge C, Williams B, Hagen S, *et al.* A process for Decision-making after Pilot and feasibility Trials (ADePT): development following a feasibility study of a complex intervention for pelvic organ prolapse. *Trials* 2013;**14**:353. doi:10.1186/1745-6215-14-353

32 Drabble S, O'Cathain A, Scott A, *et al.* Mechanisms of relationship, visibility and fit within a complex intervention to improve nebuliser adherence in adults with cystic fibrosis: a qualitative interview study (in press). *J Internet Med Res* 2020.

For peer review only

For peer review only - http://bmjopen.bmj.com/site/about/guidelines.xhtml

33 Arden M, Hind D, Drabble S. Applying the Theoretical Domains Framework: adherence to nebuliser treatment in adults with cystic fibrosis. In: *Complex interventions in a complex world: applications of Behavioural Medicine: 12th UKSBM Annual Scientific Meeting, Cardiff, 1st-2nd Dec 2016* . 2016. 0021.

34 Cherryholmes CH. Notes on Pragmatism and Scientific Realism. *Educ Res* 1992;**21**:13–7. doi:10.3102/0013189X021006013

35 Yin RK. *Case Study Research: Design and Methods*. London: : Sage 2014.

36 Ritchie J, Spencer L. Qualitative data analysis for applied policy research. In: Bryman A, Burgess RG, eds. *Analysing qualitative data*. Routledge 1994. 173–94.

37 Cane J, O'Connor D, Michie S. Validation of the theoretical domains framework for use in behaviour change and implementation research. *Implement Sci* 2012;**7**:37. doi:10.1186/1748-5908-7-37

38 Linnan L, Steckler A. Process evaluation for public health interventions and research: an overview. In: Linnan L, Steckler A, eds. *Process evaluation for public health interventions and research*. San Francisco: : Jossey-Bass 2002. 1–23.http://media.johnwiley.com.au/product_data/excerpt/66/07879597/0787959766.pdf (accessed 6 May 2014).

39 Farmer T, Robinson K, Elliott SJ, *et al.* Developing and implementing a triangulation protocol for qualitative health research. *Qual Health Res* 2006;**16**:377–94. doi:10.1177/1049732305285708

40 Yin RK. Analyzing Case Study Evidence: How to Start Your Analysis , Your Analytic Choices , and How They Work. In: *Case Study Research: Design and Methods*. London: : Sage 2014. 133–76. doi:10.1111/bjd.13028

For peer review only

For peer review only - http://bmjopen.bmj.com/site/about/guidelines.xhtml

41 Guetterman TC, Fetters MD, Creswell JW. Integrating quantitative and qualitative results in health science mixed methods research through joint displays. *Ann Fam Med* 2015;**13**:554–61. doi:10.1370/afm.1865

42 Fetters MD, Curry LA, Creswell JW. Achieving integration in mixed methods designs - Principles and practices. *Health Serv Res* 2013;**48**:2134–56. doi:10.1111/1475-6773.12117

43 Simons H. Case Study Research: In-Depth Understanding in Context. In: Leavy P, ed. *The Oxford Handbook of Qualitative Research*. Oxford: : Oxford University Press 2014. 455–70.

44 Perla RJ, Provost LP, Murray SK. The run chart: a simple analytical tool for learning from variation in healthcare processes. *BMJ Qual Saf* 2011;**20**:46–51. doi:10.1136/bmjqs.2009.037895

45 Bandura A. Social Cognitive Theory: An Agentic Perspective. *Annu Rev Psychol* 2001;**52**:1–26. doi:10.1146/annurev.psych.52.1.1

46 Carver CS, Scheier MF. Control theory: a useful conceptual framework for personality-social, clinical, and health psychology. *Psychol Bull* 1982;**92**:111–35. doi:10.1037/0033-2909.92.1.111

47 Ho YC. Abduction ? Deduction ? Induction ? Is there a Logic of Exploratory Data Analysis ? *Proc Annu Meet Am Educ Res Assoc* 1994;:28. doi:http://citeseerx.ist.psu.edu/viewdoc/download?doi=10.1.1.135.3507&rep=rep1&type=pdf

48 Peirce CS. How to make our ideas clear. *Pop Sci Mon* 1878;**12**:286–302.

49 Swedberg R. Before theory comes theorizing or how to make social science more interesting. *Br J Sociol* 2016;**67**. doi:10.1111/1468-4446.12184

50 Timmermans S, Tavory I. Theory Construction in Qualitative Research: From

For peer review only

For peer review only - http://bmjopen.bmj.com/site/about/guidelines.xhtml

Grounded Theory to Abductive Analysis. *Sociol Theory* 2012;**30**:167–86. doi:10.1177/0735275112457914

51 Miles MB, Huberman AM, Saldaña J. Methods of Ordering. In: *Qualitative Data Analysis: A Methods Sourcebook*. London: 2014. 193–220.

52 Hoo ZH, Boote J, Wildman MJ, *et al.* Determinants of objective adherence to nebulised medications among adults with cystic fibrosis: an exploratory mixed methods study comparing low and high adherers. *Heal Psychol Behav Med* 2017;**5**:299–316. doi:10.1080/21642850.2017.1338958

53 Wong MD, Sarkisian C a., Davis C, *et al.* The Association Between Life Chaos, Health Care Use, and Health Status Among HIV-Infected Persons. *J Gen Intern Med* 2007;**22**:1286–91. doi:10.1007/s11606-007-0265-6

54 Horne R, Weinman J, Hankins M. The beliefs about medicines questionnaire: The development and evaluation of a new method for assessing the cognitive representation of medication. *Psychol Health* 1999;**14**:1–24. doi:10.1080/08870449908407311

55 Clegg D, Barker R. *Case Method Fast-Track: A RAD Approach*. Boston, MA: : Addison-Wesley 1994.

56 Bittner K, Spence I. *Use Case Modeling*. Boston, MA: : Addison-Wesley Professional 2002.

57 Craig Larman. *Agile and Iterative Development: A Manager's Guide*. Boston, MA: : Addison-Wesley 2004.

58 Govan JR. Infection control in cystic fibrosis: methicillin-resistant Staphylococcus aureus, Pseudomonas aeruginosa and the Burkholderia cepacia complex. *J R Soc Med* 2000;**93 Suppl 3**:40–5.http://www.ncbi.nlm.nih.gov/pubmed/10911818

For peer review only

For peer review only - http://bmjopen.bmj.com/site/about/guidelines.xhtml

59 Wildman MJ, Hoo ZH. Moving cystic fibrosis care from rescue to prevention by embedding adherence measurement in routine care. *Paediatr Respir Rev* 2014;**15**:16–8. doi:10.1016/j.prrv.2014.04.007

60 Schattner A. Predictors of Adherence to Inhaled Medications. *J Gen Intern Med* 2013;**28**:758–758. doi:10.1007/s11606-013-2384-6

61 Battaglia M, Atkinson MA. The Streetlight Effect in Type 1 Diabetes. *Diabetes* 2015;**64**:1081–90. doi:10.2337/db14-1208

62 Kaplan A. *The Conduct of Inquiry: Methodology for Behavioral Science*. New Brunswick, N.J.: : Transaction Publishers 1964.

63 Tversky A, Kahneman D. Availability: A heuristic for judging frequency and probability. *Cogn Psychol* 1973;**5**:207–32. doi:10.1016/0010-0285(73)90033-9

64 Flume PA, Clancy JP, Retsch-Bogart GZ, *et al.* Continuous alternating inhaled antibiotics for chronic pseudomonal infection in cystic fibrosis. *J Cyst Fibros* 2016;**15**:809–15. doi:10.1016/j.jcf.2016.05.001

65 Michie S, Atkins L, West R. *The Behaviour Change Wheel: A Guide To Designing Interventions*. Sutton: : Silverback Publishing 2014.

66 National Institute for Health and Care Excellence. *Medicines optimisation: the safe and effective use of medicines to enable best possible outcomes (NG5)*. London: : NICE 2015.

67 Carver CS, Scheier MF. *On the self-regulation of behavior*. Cambridge: : Cambridge University Press 2001.

68 Yardley L, Spring BJ, Riper H, *et al.* Understanding and Promoting Effective Engagement With Digital Behavior Change Interventions. *Am J Prev Med* 2016;**51**:833–42. doi:10.1016/j.amepre.2016.06.015

69 Allemann SS, Nieuwlaat R, van den Bemt BJF, *et al.* Matching Adherence

For peer review only

For peer review only - http://bmjopen.bmj.com/site/about/guidelines.xhtml

Interventions to Patient Determinants Using the Theoretical Domains Framework. *Front Pharmacol* 2016;**7**:429. doi:10.3389/fphar.2016.00429

70 Michie S, Yardley L, West R, *et al.* Developing and evaluating digital interventions to promote behavior change in health and health care: Recommendations resulting from an international workshop. *J Med Internet Res* 2017;**19**. doi:10.2196/jmir.7126

71 Murray E, Hekler EB, Andersson G, *et al.* Evaluating Digital Health Interventions: Key Questions and Approaches. *Am J Prev Med* 2016;**51**:843–51. doi:10.1016/j.amepre.2016.06.008

72 Car J, Tan WS, Huang Z, *et al.* eHealth in the future of medications management: personalisation, monitoring and adherence. *BMC Med* 2017;**15**:73. doi:10.1186/s12916-017-0838-0

73 Baumel A, Birnbaum ML, Sucala M. A Systematic Review and Taxonomy of Published Quality Criteria Related to the Evaluation of User-Facing eHealth Programs. *J Med Syst* 2017;**41**:128. doi:10.1007/s10916-017-0776-6

74 Ryan C, Bergin M, Wells JS. Theoretical Perspectives of Adherence to Web-Based Interventions: a Scoping Review. *Int J Behav Med* Published Online First: 20 July 2017. doi:10.1007/s12529-017-9678-8

75 Van Gemert-Pijnen JE, Kelders SM, Bohlmeijer ET. Understanding the Usage of Content in a Mental Health Intervention for Depression: An Analysis of Log Data. *J Med Internet Res* 2014;**16**:e27. doi:10.2196/jmir.2991

76 Strecher V, McClure J, Alexander G, *et al.* The Role of Engagement in a Tailored Web-Based Smoking Cessation Program: Randomized Controlled Trial. *J Med Internet Res* 2008;**10**:e36. doi:10.2196/jmir.1002

77 Danaher BG, Seeley JR. Methodological Issues in Research on Web-Based

For peer review only

For peer review only - http://bmjopen.bmj.com/site/about/guidelines.xhtml

Behavioral Interventions. *Ann Behav Med* 2009;**38**:28–39. doi:10.1007/s12160-009-9129-0

78 Ainsworth B, Steele M, Stuart B, *et al.* Using an Analysis of Behavior Change to Inform Effective Digital Intervention Design: How Did the PRIMIT Website Change Hand Hygiene Behavior Across 8993 Users? *Ann Behav Med* 2017;**51**:423–31. doi:10.1007/s12160-016-9866-9

79 Hawe P. Lessons from complex interventions to improve health. *Annu Rev Public Health* 2015;**36**:307–23. doi:10.1146/annurev-publhealth-031912-114421

80 Sherman DAK, Nelson LD, Steele CM. Do Messages about Health Risks Threaten the Self? Increasing the Acceptance of Threatening Health Messages Via Self-Affirmation. *Personal Soc Psychol Bull* 2000;**26**:1046–58. doi:10.1177/01461672002611003

81 Drabble SJ, O'Cathain A, Arden MA, *et al.* When Is Forgetting Not Forgetting? A Discursive Analysis of Differences in Forgetting Talk Between Adults With Cystic Fibrosis With Different Levels of Adherence to Nebulizer Treatments. *Qual Health Res* 2019;**29**:2119–31. doi:10.1177/1049732319856580

82 Perski O, Blandford A, West R, *et al.* Conceptualising engagement with digital behaviour change interventions: a systematic review using principles from critical interpretive synthesis. *Transl Behav Med* 2017;**7**:254–67. doi:10.1007/s13142-016-0453-1

83 Frazee SG, Muzina DJ, Nease RF. Strategies to Overcome Medication Nonadherence. *JAMA* 2014;**311**:1693. doi:10.1001/jama.2014.1760

84 Mohammed MA, Moles RJ, Chen TF. Medication-related burden and patients'

For peer review only

For peer review only - http://bmjopen.bmj.com/site/about/guidelines.xhtml

lived experience with medicine: a systematic review and metasynthesis of qualitative studies. *BMJ Open* 2016;**6**:e010035. doi:10.1136/bmjopen-2015-010035

85 Huygens MWJ, Vermeulen J, Swinkels ICS, *et al.* Expectations and needs of patients with a chronic disease toward self-management and eHealth for self-management purposes. *BMC Health Serv Res* 2016;**16**:232. doi:10.1186/s12913-016-1484-5

86 Kelders SM, Oinas-Kukkonen H, Oörni A, *et al.* Health Behavior Change Support Systems as a research discipline; a viewpoint. *Int J Med Inform* Published Online First: 2016. doi:10.1016/j.ijmedinf.2016.06.022

87 Rathbone AP, Todd A, Jamie K, *et al.* A systematic review and thematic synthesis of patients' experience of medicines adherence. *Res Soc Adm Pharm* Published Online First: 2016. doi:10.1016/j.sapharm.2016.06.004

88 O'Connor S, Hanlon P, O'Donnell CA, *et al.* Understanding factors affecting patient and public engagement and recruitment to digital health interventions: a systematic review of qualitative studies. *BMC Med Inform Decis Mak* 2016;**16**:120. doi:10.1186/s12911-016-0359-3

89 Bond CM. Adherence: the Holy Grail? *BMJ Qual Saf* 2016;**25**:736–8. doi:10.1136/bmjqs-2015-004886

90 Cammidge SL, Duff AJA, Latchford GJ, *et al.* When Women with Cystic Fibrosis Become Mothers: Psychosocial Impact and Adjustments. *Pulm Med* 2016;**2016**:9458980. doi:10.1155/2016/9458980

91 Powell KR. Patient-Perceived Facilitators of and Barriers to Electronic Portal Use. *CIN Comput Informatics, Nurs* 2017;**35**:565–73. doi:10.1097/CIN.0000000000000377

For peer review only

For peer review only - http://bmjopen.bmj.com/site/about/guidelines.xhtml

92 Curtis LM, Mullen RJ, Russell A, *et al.* An efficacy trial of an electronic health record-based strategy to inform patients on safe medication use: The role of written and spoken communication. *Patient Educ Couns* 2016;**99**:1489–95. doi:10.1016/j.pec.2016.07.004

93 Morton K, Dennison L, May C, *et al.* Using digital interventions for self-management of chronic physical health conditions: A meta-ethnography review of published studies. *Patient Educ Couns* 2017;**100**:616–35. doi:10.1016/j.pec.2016.10.019

94 Hanlon P, Daines L, Campbell C, *et al.* Telehealth Interventions to Support Self-Management of Long-Term Conditions: A Systematic Metareview of Diabetes, Heart Failure, Asthma, Chronic Obstructive Pulmonary Disease, and Cancer. *J Med Internet Res* 2017;**19**:e172. doi:10.2196/jmir.6688

95 O'Cathain A, Drabble SJ, Foster A, *et al.* Being Human: A Qualitative Interview Study Exploring Why a Telehealth Intervention for Management of Chronic Conditions Had a Modest Effect. *J Med Internet Res* 2016;**18**:e163. doi:10.2196/jmir.5879

96 Oussedik, E., Foy, C.G, Masicampo, E.J., Kammrath, L.K, Anderson, R.E, Feldman SR (in press). Accountabiliy: a missing construct in models of adherence behavior and in clinical practice. *Patient Prefer Adherence* 2017;:1285–94. doi:10.2147/PPA.S135895

97 Rudd RE. Health literacy skills of U.S. adults. *Am J Health Behav* 2007;**31 Suppl 1**:S8-18. doi:10.5555/ajhb.2007.31.supp.S8

98 DeWalt DA, Pignone M, Malone R, *et al.* Development and pilot testing of a disease management program for low literacy patients with heart failure. *Patient Educ Couns* 2004;**55**:78–86. doi:10.1016/j.pec.2003.06.002

For peer review only - http://bmjopen.bmj.com/site/about/guidelines.xhtml

99 Sarkar U, Fisher L, Schillinger D. Is Self-Efficacy Associated With Diabetes Self-Management Across Race/Ethnicity and Health Literacy? *Diabetes Care* 2006;**29**:823–9. doi:10.2337/diacare.29.04.06.dc05-1615

100 Guest G, Bunce A, Johnson L. How Many Interviews Are Enough?: An Experiment with Data Saturation and Variability. *Field methods* 2006;**18**:59–82. doi:10.1177/1525822X05279903

101 Malterud K, Siersma VD, Guassora AD. Sample Size in Qualitative Interview Studies: Guided by Information Power. *Qual Health Res* 2015;:1–8. doi:10.1177/1049732315617444

102 Liu H, Mohammed A, Shanthosh J, *et al.* Process evaluations of primary care interventions addressing chronic disease: a systematic review. *BMJ Open* 2019;**9**:e025127. doi:10.1136/bmjopen-2018-025127

103 Beets MW, Weaver RG, Ioannidis JPA, *et al.* Identification and evaluation of risk of generalizability biases in pilot versus efficacy/effectiveness trials: a systematic review and meta-analysis. *Int J Behav Nutr Phys Act* 2020;**17**:19. doi:10.1186/s12966-020-0918-y

104 Cooper CL, Whitehead A, Pottrill E, *et al.* Are pilot trials useful for predicting randomisation and attrition rates in definitive studies: A review of publicly funded trials. *Clin Trials* 2018;**15**:189–96. doi:10.1177/1740774517752113

105 Tappenden P, Sadler S, Wildman M. An Early Health Economic Analysis of the Potential Cost Effectiveness of an Adherence Intervention to Improve Outcomes for Patients with Cystic Fibrosis. *Pharmacoeconomics* 2017;**35**:647–59. doi:10.1007/s40273-017-0500-x

106 O'Donohoe R, Fullen BM. Adherence of subjects with cystic fibrosis to their home program: a systematic review. *Respir Care* 2014;**59**:1731–46.

For peer review only - http://bmjopen.bmj.com/site/about/guidelines.xhtml

doi:10.4187/respcare.02990

107 Sawicki GS, Goldman DP, Chan W, *et al.* Patient preference for treatment administration in cystic fibrosis. *Am J Pharm Benefits* 2015;**7**:174–81.

108 Phillips LA, Cohen J, Burns E, *et al.* Self-management of chronic illness: the role of 'habit' versus reflective factors in exercise and medication adherence. *J Behav Med* 2016;**39**:1076–91. doi:10.1007/s10865-016-9732-z

109 Lally P, van Jaarsveld CHM, Potts HWW, *et al.* How are habits formed: Modelling habit formation in the real world. *Eur J Soc Psychol* 2010;**40**:998–1009. doi:10.1002/ejsp.674

110 Arden MA, Drabble S, O'Cathain A, *et al.* Adherence to medication in adults with Cystic Fibrosis: An investigation using objective adherence data and the Theoretical Domains Framework. *Br J Health Psychol* Published Online First: March 2019. doi:10.1111/bjhp.12357

111 Hoo ZH, Gardner B, Arden MA, *et al.* Role of habit in treatment adherence among adults with cystic fibrosis. *Thorax* 2019;**74**:197–9. doi:10.1136/thoraxjnl-2017-211453

112 Conn VS, Ruppar TM. Medication adherence outcomes of 771 intervention trials: Systematic review and meta-analysis. *Prev Med (Baltim)* 2017;**99**:269–76. doi:10.1016/j.ypmed.2017.03.008

113 Goldzweig CL, Orshansky G, Paige NM, *et al.* Electronic patient portals: evidence on health outcomes, satisfaction, efficiency, and attitudes: a systematic review. *Ann Intern Med* 2013;**159**:677–87. doi:10.7326/0003-4819-159-10-201311190-00006

114 Lau AY, Arguel A, Dennis S, *et al.* "Why Didn't it Work?" Lessons From a Randomized Controlled Trial of a Web-based Personally Controlled Health

For peer review only

For peer review only - http://bmjopen.bmj.com/site/about/guidelines.xhtml

Management System for Adults with Asthma. *J Med Internet Res* 2015;**17**:e283. doi:10.2196/jmir.4734

115 Kruse CS, Kothman K, Anerobi K, *et al.* Adoption Factors of the Electronic Health Record: A Systematic Review. *JMIR Med informatics* 2016;**4**:e19. doi:10.2196/medinform.5525

116 Greenhalgh T, Swinglehurst D, Stones R. Rethinking resistance to 'big IT': a sociological study of why and when healthcare staff do not use nationally mandated information and communication technologies. *Heal Serv Deliv Res* 2014;**2**:1–86. doi:10.3310/hsdr02390

117 Ross J, Stevenson F, Lau R, *et al.* Factors that influence the implementation of e-health: a systematic review of systematic reviews (an update). *Implement Sci* 2016;**11**:146. doi:10.1186/s13012-016-0510-7

118 Hennemann S, Beutel ME, Zwerenz R. Ready for eHealth? Health Professionals' Acceptance and Adoption of eHealth Interventions in Inpatient Routine Care. *J Health Commun* 2017;**22**:274–84. doi:10.1080/10810730.2017.1284286

119 Harris M, Lawn SJ, Morello A, *et al.* Practice change in chronic conditions care: an appraisal of theories. *BMC Health Serv Res* 2017;**17**:170. doi:10.1186/s12913-017-2102-x

120 Ovretveit J, Leviton L, Parry G. Increasing the generalisability of improvement research with an improvement replication programme. *BMJ Qual Saf* 2011;**20**:i87–91. doi:10.1136/bmjqs.2010.046342

121 Eikey E V, Reddy MC, Kuziemsky CE. Examining the role of collaboration in studies of health information technologies in biomedical informatics: A systematic review of 25 years of research. *J Biomed Inform* 2015;**57**:263–77.

For peer review only

For peer review only - http://bmjopen.bmj.com/site/about/guidelines.xhtml

doi:10.1016/j.jbi.2015.08.006

122 Girling C, Hind D, Arden MA, *et al.* Developing an implementation strategy for the use of objective adherence data in routine clinical practice: a case study in cystic fibrosis clinics. *Implement Sci* 2019;**14**:P9. doi:10.1186/s13012-019-0911-5

123 Bevan A, Tahir M, Miller B, *et al.* P337 Easy Medicines for Burden Reduction and Care Enhancement: using real time adherence data to optimise inhaled therapies in adults with cystic fibrosis. The UK National EMBRACE programme. *J Cyst Fibros* 2020;**19**:S150. doi:10.1016/S1569-1993(20)30666-4

124 Relton C, Torgerson D, O'Cathain A, *et al.* Rethinking pragmatic randomised controlled trials: introducing the 'cohort multiple randomised controlled trial' design. *BMJ* 2010;**340**:c1066. doi:10.1136/bmj.c1066

125 Young-Afat DA, Verkooijen HAM, van Gils CH, *et al.* Staged-informed Consent in the Cohort Multiple Randomized Controlled Trial Design. *Epidemiology* 2016;**27**:389–92. doi:10.1097/EDE.0000000000000435

126 Retsch-Bogart GZ, Van Dalfsen JM, Marshall BC, *et al.* Highly effective cystic fibrosis clinical research teams: critical success factors. *J Gen Intern Med* 2014;**29 Suppl 3**:S714-23. doi:10.1007/s11606-014-2896-8

127 Hibbard JH, Mahoney ER, Stockard J, *et al.* Development and testing of a short form of the patient activation measure. *Health Serv Res* 2005;**40**:1918–30. doi:10.1111/j.1475-6773.2005.00438.x

128 Verplanken BAS, Ell OR, Orbell S. Reflections on past behavior: A self-report index of habit strength. *J Appl Soc Psychol* 2003;**33**:1313–30. doi:10.1111/j.1559-1816.2003.tb01951.x

For peer review only

For peer review only - http://bmjopen.bmj.com/site/about/guidelines.xhtml

…

For peer review only

For peer review only - http://bmjopen.bmj.com/site/about/guidelines.xhtml

Tables

Table 1 - Quantitative data contributing to the understanding of logic model constructs

#	Logic model column / construct	Quantitative
	**INPUTS**	
3	Prescription data	CFHH; problems documented in implementation log.
7	COM-BMQ questionnaire responses	Capability Opportunity Motivation Behaviour Beliefs Questionnaire (COM-BMQ), incorporating the Beliefs about Medicines Questionnaire (Nebuliser adherence)[54], one additional belief item, one intention item, one confidence item, and a list of barriers
9	Interventionist training programme	Structured questionnaire on interventionist confidence after training programme.
11	Competency/Fidelity assessment	Structured instrument for the assessment of interventionist competence.
	**ENGAGEMENT**	
13	Clinicians accessing adherence data*	CFHH click analytics.
14	Adherence data tracking	CFHH click analytics.
15	Participant accessing CFHealthHub	CFHH click analytics.

For peer review only - http://bmjopen.bmj.com/site/about/guidelines.xhtml

17	CFHealthHub Intervention sessions delivered according to Manual (Fidelity)	Project-specific structured fidelity assessment instrument.
18	Initial session, and then review at each clinic visit	CFHH click analytics.
	**ACTIVITIES**	
	Intervention components for all participants	
20	Self-monitoring adherence	CFHH click analytics.
21	Tailored education about treatment	CFHH click analytics.
22	Tailored patient stories (videos)	CFHH click analytics.
	Intervention components for those with adequate motivation	
23	Personalised goal-setting	CFHH click analytics.
24	Goal review	CFHH click analytics.
25	Personalised action plan	CFHH click analytics.
26	Tailored problem-solving	CFHH click analytics.
	**IMMEDIATE OUTCOMES**	
	For all participants	

For peer review only - http://bmjopen.bmj.com/site/about/guidelines.xhtml

28	Acute awareness of adherence / increased Motivation	Subjective adherence single question (self-report estimate of adherence as a percentage); COM-BMQ.
29	Increased necessity and decreased concern	COM-BMQ and Patient Activation Measure (PAM-13[127])
30	Increased self-efficacy / Motivation	COM-BMQ single question about confidence to adhere; PAM-13.
	For those with adequate motivation	
31	Increased self-efficacy/ Motivation	COM-BMQ single question about confidence to adhere; PAM-13.
32	Increased habit / reduced chaos	Self-Report Behavioural Automaticity Index (SRBAI) automaticity-specific subscale of the Self Report Habit index to capture habit-based behaviour patterns[128]; Confusion, Hubbub, and Order Scale (CHAOS 6-item): measure of life chaos[53].
33	Reduced barriers	No change in the group averages for The Beliefs about Medicines Questionnaire - specific (Nebuliser adherence) (BMQ 21-item[54])
	**INTERMEDIATE OUTCOMES**	

For peer review only - http://bmjopen.bmj.com/site/about/guidelines.xhtml

35	Increased adherence	Nebuliser data (CFHH)

For peer review only - http://bmjopen.bmj.com/site/about/guidelines.xhtml

Figures

Figure 1 - Logic model

Figure 2 - The digital platform

Figure 3 – Objective versus subjective adherence at 5 (+/- 1) months stratified by adherence

Additional files

Additional file 01 – Participant adherence run charts for fourteen case studies

Additional file 02 – Study protocol and interview guides

Additional file 03 – Quantitative results from process evaluation

Additional file 04 – Joint display table

Additional file 05 – Case-ordered descriptive matrix for fourteen case studies

Additional file 06 – Statistical methods, outcomes and estimation

Additional file 07 – Changes to intervention procedures

For peer review only

For peer review only - http://bmjopen.bmj.com/site/about/guidelines.xhtml

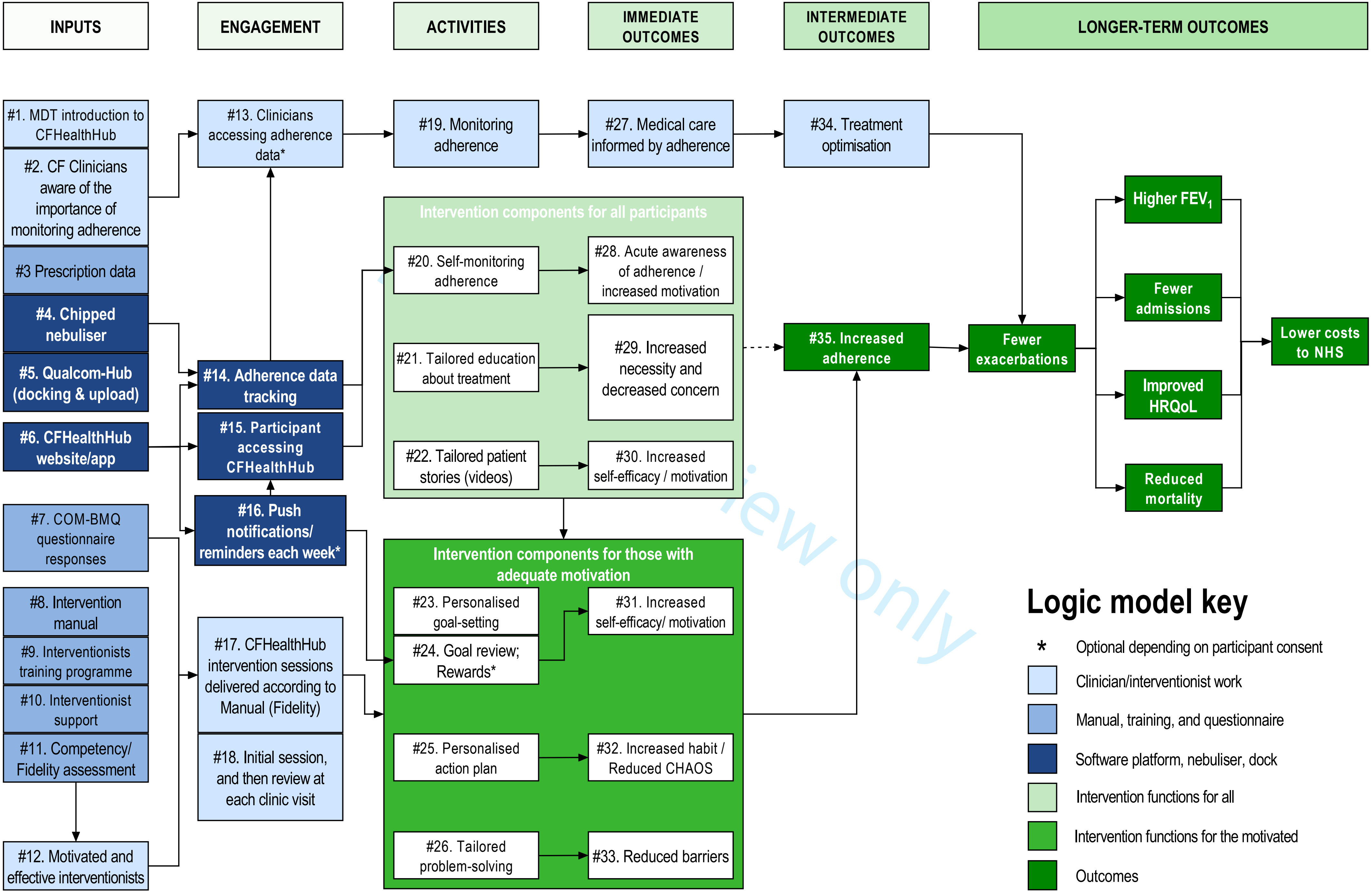
INPUTS
ENGAGEMENT
ACTIVITIES
IMMEDIATE OUTCOMES
INTERMEDIATE OUTCOMES
LONGER-TERM OUTCOMES
#1. MDT introduction to CFHealthHub
#2. CF Clinicians aware of the importance of monitoring adherence
#3 Prescription data
#4. Chipped nebuliser
#5. Qualcom-Hub (docking & upload)
#6. CFHealthHub website/app
#7. COM-BMQ questionnaire responses
#8. Intervention manual
#9. Interventionists training programme
#10. Interventionist support
#11. Competency/ Fidelity assessment
#12. Motivated and effective interventionists
#13. Clinicians accessing adherence data*
#14. Adherence data tracking
#15. Participant accessing CFHealthHub
#16. Push notifications/ reminders each week*
#17. CFHealthHub intervention sessions delivered according to Manual (Fidelity)
#18. Initial session, and then review at each clinic visit
#19. Monitoring adherence
#27. Medical care informed by adherence
#34. Treatment optimisation
Intervention components for all participants
#20. Self-monitoring adherence
#21. Tailored education about treatment
#22. Tailored patient stories (videos)
#28. Acute awareness of adherence / increased motivation
#29. Increased necessity and decreased concern
#30. Increased self-efficacy / motivation
Intervention components for those with adequate motivation
#23. Personalised goal-setting
#24. Goal review; Rewards*
#25. Personalised action plan
#26. Tailored problem-solving
#31. Increased self-efficacy/ motivation
#32. Increased habit / Reduced CHAOS
#33. Reduced barriers
#35. Increased adherence
Fewer exacerbations
Higher FEV1
Fewer admissions
Improved HRQoL
Reduced mortality
Lower costs to NHS
Logic model key
* Optional depending on participant consent
Clinician/interventionist work
Manual, training, and questionnaire
Software platform, nebuliser, dock
Intervention functions for all
Intervention functions for the motivated
Outcomes

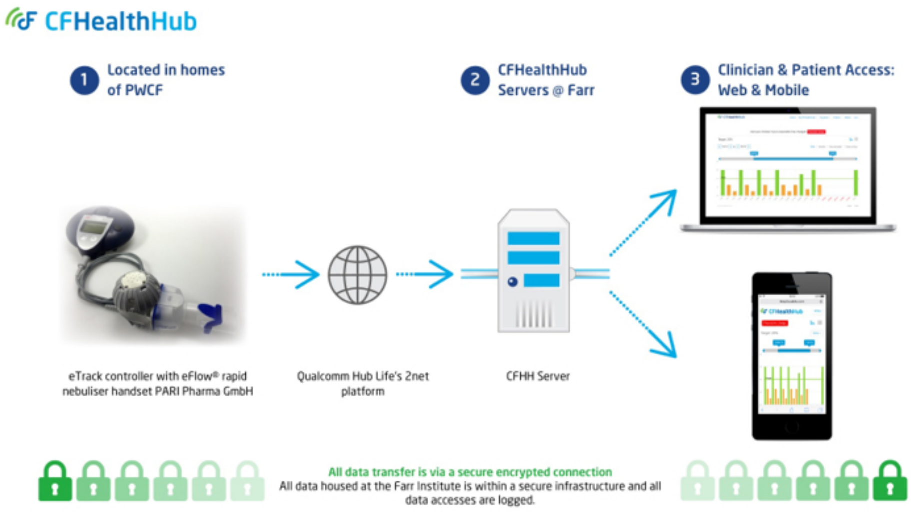

The digital platform

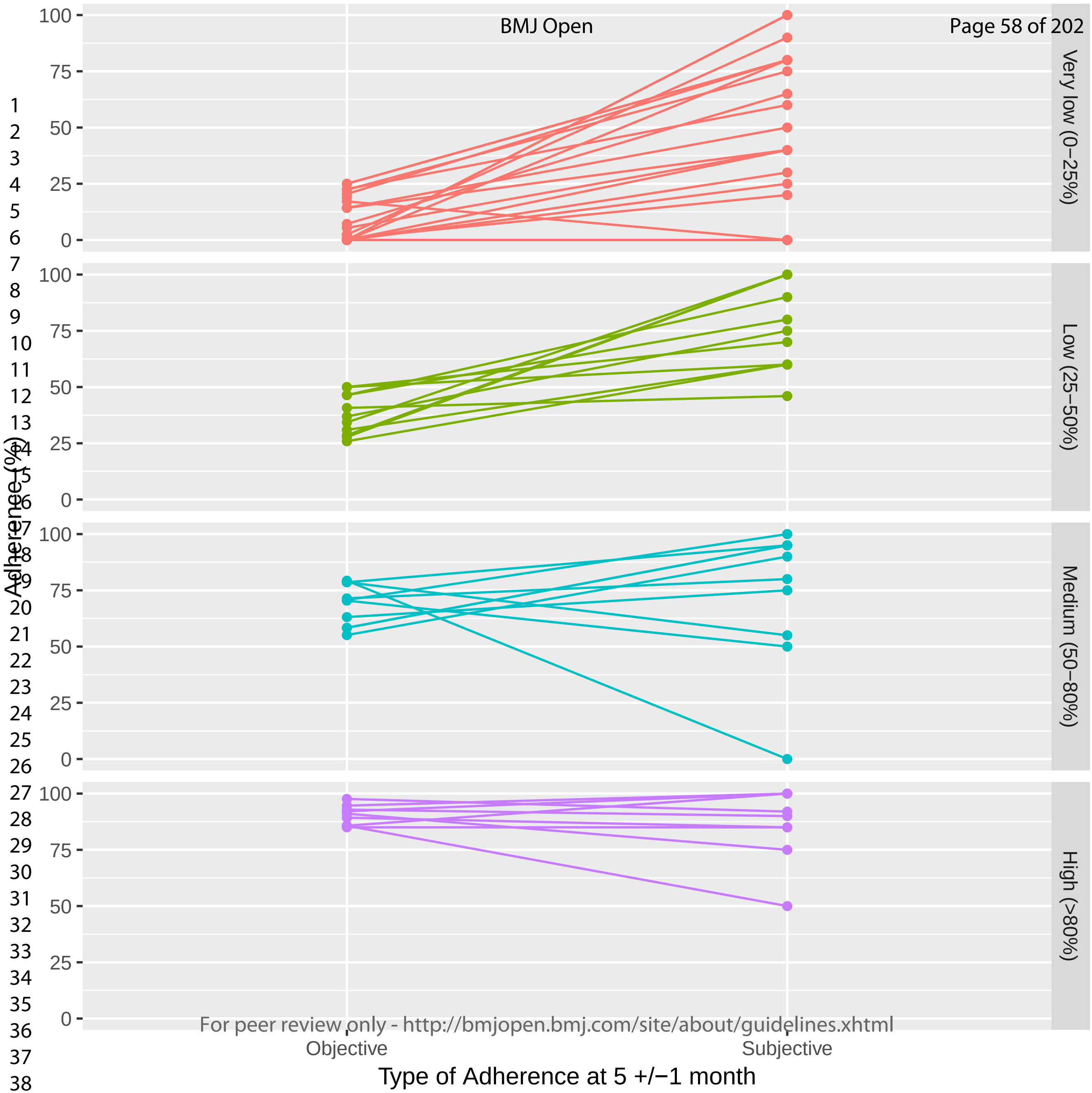

For peer review only - http://bmjopen.bmj.com/site/about/guidelines.xhtml

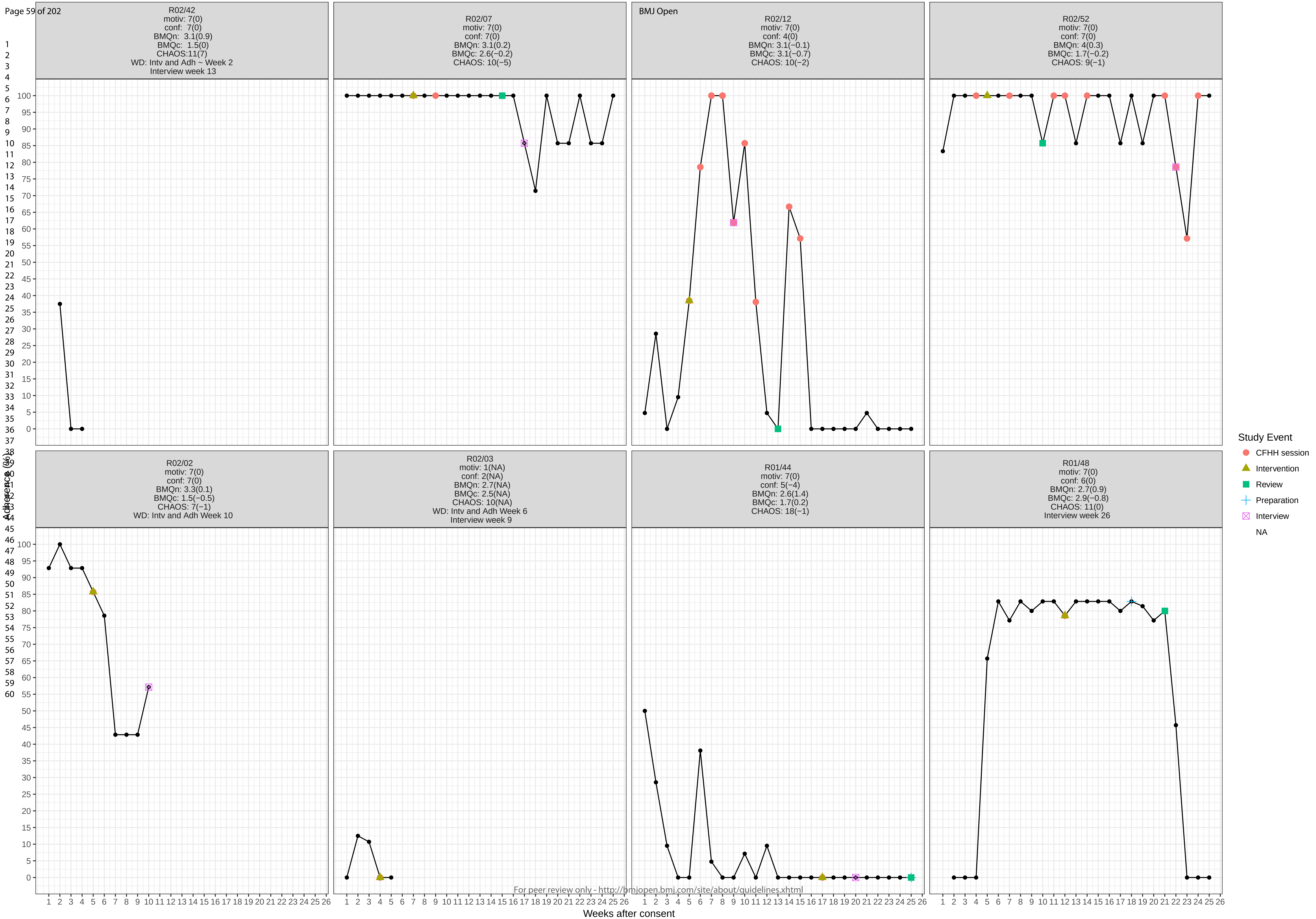
R02/42
motiv: 7(0)
conf: 7(0)
BMQn: 3.1(0.9)
BMQc: 1.5(0)
CHAOS:11(7)
WD: Intv and Adh ~ Week 2
Interview week 13
R02/07
motiv: 7(0)
conf: 7(0)
BMQn: 3.1(0.2)
BMQc: 2.6(−0.2)
CHAOS: 10(−5)
R02/12
motiv: 7(0)
conf: 4(0)
BMQn: 3.1(−0.1)
BMQc: 3.1(−0.7)
CHAOS: 10(−2)
R02/52
motiv: 7(0)
conf: 7(0)
BMQn: 4(0.3)
BMQc: 1.7(−0.2)
CHAOS: 9(−1)
R02/02
motiv: 7(0)
conf: 7(0)
BMQn: 3.3(0.1)
BMQc: 1.5(−0.5)
CHAOS: 7(−1)
WD: Intv and Adh Week 10
R02/03
motiv: 1(NA)
conf: 2(NA)
BMQn: 2.7(NA)
BMQc: 2.5(NA)
CHAOS: 10(NA)
WD: Intv and Adh Week 6
Interview week 9
R01/44
motiv: 7(0)
conf: 5(−4)
BMQn: 2.6(1.4)
BMQc: 1.7(0.2)
CHAOS: 18(−1)
R01/48
motiv: 7(0)
conf: 6(0)
BMQn: 2.7(0.9)
BMQc: 2.9(−0.8)
CHAOS: 11(0)
Interview week 26
Adherence (%)
Weeks after consent
Study Event
CFHH session
Intervention
Review
Preparation
Interview
NA

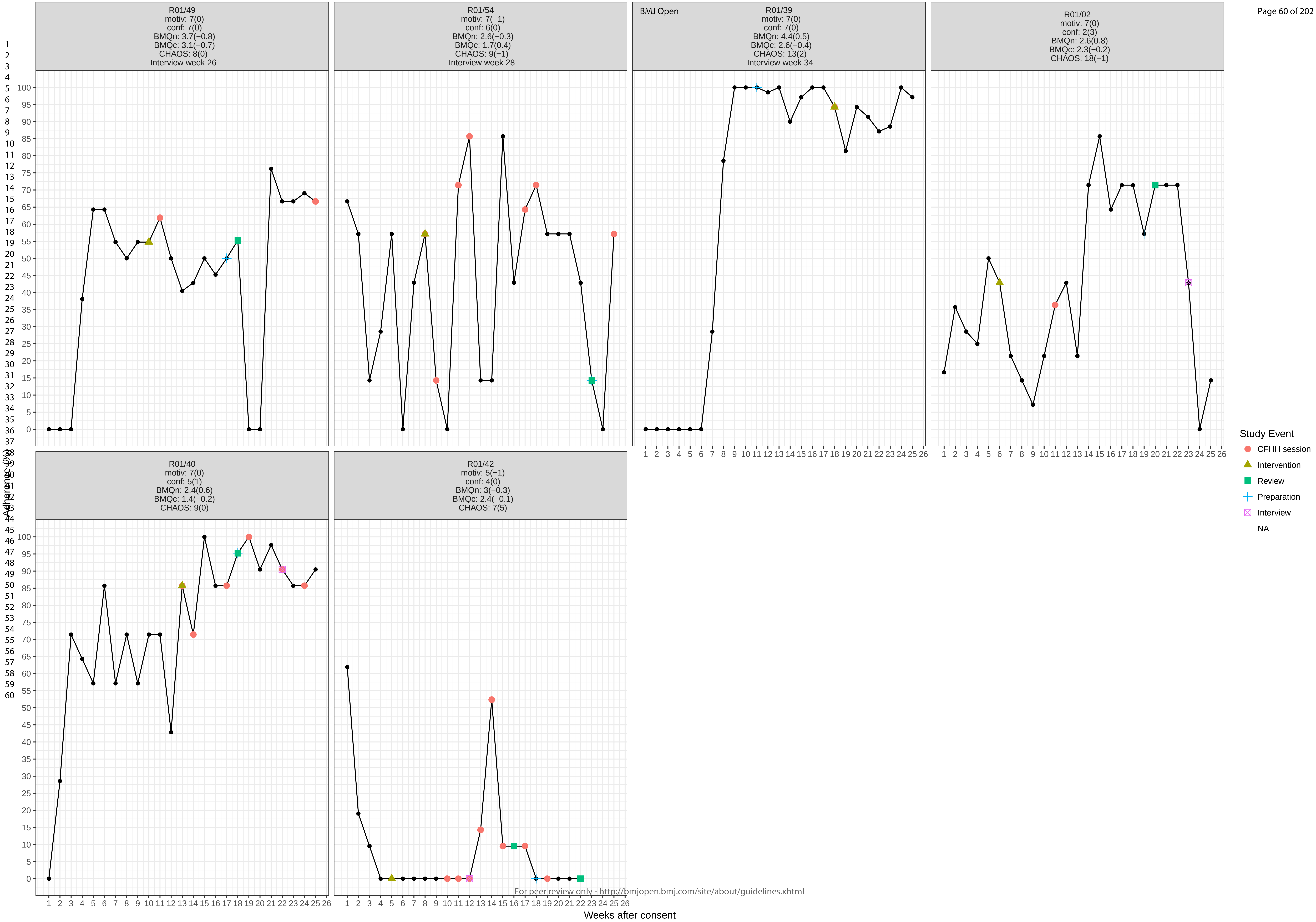
R01/49
motiv: 7(0)
conf: 7(0)
BMQn: 3.7(−0.8)
BMQc: 3.1(−0.7)
CHAOS: 8(0)
Interview week 26
R01/54
motiv: 7(−1)
conf: 6(0)
BMQn: 2.6(−0.3)
BMQc: 1.7(0.4)
CHAOS: 9(−1)
Interview week 28
R01/39
motiv: 7(0)
conf: 7(0)
BMQn: 4.4(0.5)
BMQc: 2.6(−0.4)
CHAOS: 13(2)
Interview week 34
R01/02
motiv: 7(0)
conf: 2(3)
BMQn: 2.6(0.8)
BMQc: 2.3(−0.2)
CHAOS: 18(−1)
R01/40
motiv: 7(0)
conf: 5(1)
BMQn: 2.4(0.6)
BMQc: 1.4(−0.2)
CHAOS: 9(0)
R01/42
motiv: 5(−1)
conf: 4(0)
BMQn: 3(−0.3)
BMQc: 2.4(−0.1)
CHAOS: 7(5)
Adherence (%)
Weeks after consent
Study Event
CFHH session
Intervention
Review
Preparation
Interview
NA

ACtiF

Development and evaluation of an intervention to support Adherence to treatment in adults with Cystic Fibrosis

A feasibility study comprised of an external pilot randomised controlled trial and process evaluation

RESEARCH PROTOCOL
V3.1 16Nov16

**Sheffield CTRU**	J13-002
**IRAS**	199775
**ISRCTN**	ISRCTN13076797
**Authorised by:**	Martin Wildman
	Alicia O'Cathain

For peer review only - http://bmjopen.bmj.com/site/about/guidelines.xhtml

Funding

This project was funded by the Programme Grants for Applied Research programme (RP-PG-1212-20015) and will be published in full in the NIHR Journals Library. This research presents independent research commissioned by the NIHR. The views and opinions expressed by authors in this publication are those of the authors and do not necessarily reflect those of the NHS, the NIHR, MRC, CCF, NETSCC, the Health Technology Assessment programme or the Department of Health.

Roles and responsibilities

Names, affiliations, and roles of protocol contributors

The following all contributed to drafting elements throughout the protocol: Martin Wildman, Consultant Respiratory Medic; Marlene Hutchings, Physiotherapist (Sheffield Teaching Hospitals NHS Foundation Trust); Madelynne Arden, Professor of Health Psychology (Sheffield Hallam University); Alicia O'Cathain, Professor of Health Services Research; Sarah Drabble, Research Associate, Stephen Walters (Professor of Medical Statistics and Clinical Trials); Paul Tappenden, Reader in Health Economics; Daniel Beever, Research Associate and Patient Representative; Pauline Wheelan mHealth Applications Manager (University of Manchester); Judy Bradley, Professor of Physiotherapy and Health Services Research; Hannah Cantrill (Research Associate); Chin Maguire (Study Manager); and, Daniel Hind, Assistant Director, Sheffield Clinical Trials Research Unit (University of Sheffield). MW and MA are guarantors of the intervention description; SW is the guarantor of the statistical design and analysis plan; DH is the guarantor of other elements of the pilot trial; AO'C is guarantor for the qualitative research components.

Sponsor

Sheffield Teaching Hospitals NHS Foundation Trust
D Floor
Clinical Research Office
Royal Hallamshire Hospital
Glossop Road
Sheffield
S10 2JF
Tel: (0114) 2256424
Fax: (0114)

Role of study sponsor and funders

Neither the funder nor the sponsor have had any role in study design, data collection and analysis, decision to publish, or preparation of manuscripts.

For peer review only - http://bmjopen.bmj.com/site/about/guidelines.xhtml

Key Contacts

Study Manager

Chin Maguire
Clinical Trials Research Unit
The University of Sheffield
School of Health and Related Research
30 Regent Street
Sheffield
S1 4DA
Tel: (0114) 222 0717
Fax: (0114) 222 0870
e-mail: c.maguire@sheffield.ac.uk

Chief Investigator

Dr Martin Wildman
Adult CF Centre
Northern General Hospital
Herries Road
Sheffield
S5 7AU

Tel: (0114) 2715212
Fax: (0114) 222 0870
e-mail: Martin.Wildman@sth.nhs.uk

Composition, roles, and responsibilities

Project Management Group

Name	Role	Institution
Dr Martin Wildman	Chief Investigator	Sheffield Teaching Hospitals NHS Foundation Trust
Professor Alicia O'Cathain	Chief Investigator	University of Sheffield
Dr Daniel Hind	CTRU Assistant Director	University of Sheffield
Chin Maguire	Study Manager	University of Sheffield
Hannah Cantrill	Research Assistant	University of Sheffield
Helen Wakefield	Trial Support Office	University of Sheffield
Dan Beever	PPI Lead	University of Sheffield
Dr Sarah Drabble	Qualitative Researcher	University of Sheffield
Professor Maddy Arden	Professor of Health Psychology	Sheffield Hallam University
Marlene Hutchings	Research Physiotherapist	Sheffield Teaching Hospitals NHS Foundation Trust
Professor Judy Bradley	Professor in Physiotherapy	Queens University Belfast

For peer review only - http://bmjopen.bmj.com/site/about/guidelines.xhtml

Independent Project Steering Committee

Name	Role	Institution
Professor Kathy Rowan	Independent Chair	London School of Hygiene and Tropical Medicine
Professor Sarah Garner	Independent member	The National Institute for Health and Care Excellence
Professor David Torgerson	Independent Member	University of York
Dr Lisa Hampson	Independent Statistician	Lancaster University
Dominic Kavanagh	PPI Representative	
Dr Martin Wildman	Chief Investigator	Sheffield Teaching Hospitals NHS Foundation Trust
Professor Alicia O'Cathain	Chief Investigator	University of Sheffield
Dr Daniel Hind	CTRU Assistant Director	University of Sheffield
Chin Maguire	Study Manager	University of Sheffield
Professor Maddy Arden	Professor of Health Psychology	Sheffield Hallam University

For peer review only - http://bmjopen.bmj.com/site/about/guidelines.xhtml

Contents

Funding 2
Names, affiliations, and roles of protocol contributors 2
Sponsor 2
Role of study sponsor and funders..2
Composition, roles, and responsibilities 3
Project Management Group 3
Independent Project Steering Committee 4
Contents 5
Figures 7
Tables..7
1. Lay summary 8
2. Introduction..9
2.1 Background..9
2.2 Rationale 9
3. Aim and objectives 11
3.1 Aims..11
3.2 Objectives 11
4. Design 12
5. Participants and study settings 13
5.1 Settings and locations where the data will be collected.....................................13
5.2 Eligibility 13
5.2.1 Inclusion criteria for participants...13
5.2.2 Exclusion criteria for participants..13
5.2.3 Eligibility criteria for study centres 13
5.2.4 Eligibility criteria for interventionists..13
6. Interventions 14
6.1 Summary..14
6.2 Microchipped devices 15
6.2.1 The eTrack nebuliser system (Pari GmbH) 15
6.2.2 The Bi-neb AAD System from (Philips Healthcare) 15
6.3 Information technology infrastructure 15
6.3.1 The Qualcomm hub 15
6.3.2 CFHealthHub..15
6.3.3 The Bi-Neb data transfer system 16
6.4 The Behaviour Change Intervention (BCI) 17
6.4.1 Rationale and theory 17
6.4.2 Intervention providers..22
6.4.3 Materials 22
6.4.4 Procedures..22
6.5 Usual care 27
6.6 Criteria for discontinuing or modifying allocated interventions 27
6.7 Strategies to improve adherence to intervention protocols..................................28
6.7.1 For health professionals..28
6.7.2 For patients 28
6.8 Relevant permitted / prohibited concomitant care 28
7. Outcomes 29
7.1 Feasibility outcomes (‘stop-go’ or ‘success’ criteria for RCT) 29
7.2 Process data relating to the implementation of the trial......................................29

For peer review only - http://bmjopen.bmj.com/site/about/guidelines.xhtml

7.3 Process data relating to the implementation of the intervention....30
7.4 Clinical outcomes and covariates 31
7.4.1 Primary clinical outcome 31
7.4.2 Secondary clinical outcomes 32
7.4.3 Adherence to prescribed medication....33
8. Sampling 37
8.1 Quantitative components 37
8.1.1 Sites....37
8.1.2 Sample size 37
8.1.3 Approach, non-participation and recruitment 37
8.2 Qualitative components 38
9. Assignment of interventions 38
9.1 Sequence generation 38
9.2 Allocation concealment 38
9.3 Implementation 38
9.4 Blinding 38
10. Data collection, management and analysis 39
10.1 Quantitative data 39
10.1.1 Data collection methods....39
10.1.2 Data Management 40
10.1.3 Data quality assurance 40
10.2 Qualitative data 41
10.2.1 Audio recordings of consultations 41
10.2.2 Semi-structured interviews: participants 41
10.2.3 Semi-structured interviews: professionals 41
10.2.4 Undertaking the interviews 41
11. Data analysis 42
11.1 Quantitative analysis....42
11.2 Qualitative analysis....43
11.3 Combining data and findings from the different components 44
12. Monitoring 44
12.1 Oversight....44
12.2 Description of any interim analyses and stopping guidelines....45
12.3 Harms (safety assessments) 45
12.3.1 Serious Adverse Events 45
12.3.2 Adverse events we require reporting: 46
12.3.3 Expected SAEs and adverse events 46
12.3.4 Reporting 47
12.4 Auditing 47
12.5 Finance and indemnity....47
13. Ethics and dissemination 48
13.1 Approvals....48
13.2 Protocol amendments....48
13.3 Consent 48
13.4 Confidentiality 49
13.5 Declaration of Interests 49
13.6 Access to data 49
13.7 Ancilliary and post-trial care 49
13.8 Dissemination policy 49
References....51
Appendix 1. W.H.O. Trial Registration Data Set 56

For peer review only - http://bmjopen.bmj.com/site/about/guidelines.xhtml

Figures

Figure 1. Interaction between complex intervention components 14
Figure 2. Interactions between capability, opportunity and motivation 17
Figure 3. Habit formation incorporating COM-B and necessities and concerns 18
Figure 4. Interplay between COM-B components during habit formation.............................. 18
Figure 5. Behaviour change intervention flow chart 26
Figure 6. Participant timeline for the external pilot RCT 36
Figure 7. Assumptions of the MRC Guidance on Process Evaluation 43

Tables

Table 1. Learning objectives of the CFHealthHub modules 16
Table 2: Intervention modules 20
Table 3. Individual-level data derived from PWCF and sites.. 34
Table 4. CFHealthHub data (research arm only) 35

For peer review only - http://bmjopen.bmj.com/site/about/guidelines.xhtml

1. Lay summary

Cystic Fibrosis (CF) is an inherited disease affecting 10000 people in the UK with an average age at death of 28 years in 2012. The lungs of people with CF (PWCF) are prone to infections. Daily physiotherapy and inhaled medications are needed to stay healthy. Around £30 million is spent annually on inhaled therapy but average adherence has been shown to be only 36%. Data suggest that adherence is better in younger children (71% in under 12s, falling to 50% in teenagers) but of the 10000 UK PWCF almost 6000 are now adults. PWCF who collect <50% of their medication cost the healthcare system significantly more than PWCF who collect more than 80% and most of the additional cost results from unscheduled emergency care and hospital admission. This unscheduled emergency care is distressing for PWCF and their families.

We have designed an intervention to help adult PWCF see how much treatment they use. We use dose-counting nebulisers to collect data and send it to a website where it can be displayed. We have worked with PWCF to make the information easy to understand. The website has modules which teach PWCF how to build successful treatment habits. We have developed a toolkit to help PWCF and a health professional (interventionist) work together to form habits of adherence to treatment.

The NHS should not fund this intervention without its effectiveness and value for money being evaluated in a Randomised Controlled Trial (RCT). However, there is currently insufficient information to effectively plan or justify funding a RCT on the scale required. This feasibility study is an essential preliminary to the full scale RCT. The purpose of this feasibility study is to see whether the proposed procedures for the full scale RCT are feasible and acceptable to PWCF. It will also tell us whether the intervention can be delivered by health professionals and is acceptable to PWCF, outside the NHS trust where it was developed.

We will recruit PWCF for four months at two CF units. We hope we will recruit 64 PWCF overall, but will deem the full scale RCT feasible if we recruit 48. A computer will decide whether people who consent to be in the study will receive usual care alone or also receive the intervention. Both groups have a short period of two to four weeks when data is collected through their nebulisers and fed back to the website. It is only after that period that those allocated to the intervention are allowed to use the website and receive enhanced care from the interventionist. After that point, all participants are followed up for 5 (+/-) months. Participants will complete a series of questionnaires at the outset and at 5 (+/-) months.

With appropriate consent, the interventionist or member of the multidisciplinary team (MDT) will audio record consultations between themselves and PWCF who are receiving the intervention or usual care. Qualitative researchers will conduct: 20-24 interviews with PWCF receiving the intervention; 20-24 interviews with PWCF receiving usual care; eight interviews with the four health professionals who are delivering the intervention; and eight semi-structured interviews with members of the wider MDT. These interviews are intended to help the team understand and mitigate potential sources of failure in the intervention and the proposed full-scale trial.

For peer review only - http://bmjopen.bmj.com/site/about/guidelines.xhtml

2. Introduction

2.1 Background

Cystic Fibrosis (CF) is a long term condition (LTC) in which poor adherence to high cost drugs shortens lives and increases NHS costs. CF is a LTC affecting 10,000 people in the UK with PWCF typically dying from lung damage at a median age of 28 years [1]. Randomised controlled trials show that preventative medications reduce exacerbations and/or preserve lung function, [2–8] however adherence is poor. A recent review of objective measures of adherence using medicine possession ratios (MPR: prescriptions collected over prescriptions issued) and instrumented medication monitors showed adherence ranging from 67% for oral antibiotics, 31-53% for inhaled antibiotics, 53-79% for mucolytics agents and 41-72% for hypertonic saline [9]. Accumulating evidence suggests poor adherence is associated with poor outcomes. PWCF collecting four or more courses of alternate month nebulised tobramycin per year were 60% less likely to be admitted to hospital than PWCF collecting one or less [10]. Lower composite MPR predicted exacerbations requiring intravenous antibiotics (IVAB) [9] and over a 12 month period PWCF with an MPR of 80% had significantly lower total healthcare costs than PWCF with an MPR <50% with a cost difference $14,211 per patient and most excess costs related to hospital care [11] . Rescue therapy with IVAB can cause renal failure [12]. The total 2012 UK spend for CF was estimated to be £100 million of which £30 million was spent on inhaled antibiotics and mucolytics [13]. Although patient self-reported adherence to inhaled therapy was 80%, objective measurement showed median adherence was only 36% and the clinicians were unable to predict which PWCF were able to successfully adhere [14] making adherence support difficult. In 2012, the UK CF population received 171,907 days of IVAB with the 93,455 of these that occurred in hospital costing an estimated £27 million [15] . It is recommended that adherence interventions should be targeted where adherence really matters [16] and targeting support towards the high cost inhaled preventative drugs in CF (median adherence 36%) has the potential to impact on the 171,907 days of IVAB a proportion of which will represent rescue therapy necessitated by failed prevention.

2.2 Rationale

The National Institute for Health Research have commissioned a Programme Grant for Applied Research to systematically develop and evaluate an adherence intervention for PWCF. The Programme Grant has three work packages

Work package 1: Build IT infrastructure to capture adherence data from nebulisers. Co-produce a web-portal, ‘CFHealthHub’, with PWCF and clinicians, in order to display routinely collected adherence data for the use of both groups.

Work package 2: Develop a toolkit based on psychological theory that can support PWCF to adhere to treatment. This will include feedback of measured adherence data and personalised interventions to increase adherence delivered through CFHealthHub. Manualise a Behaviour Change Intervention (BCI) for use by health professionals and PWCF.
All four work packages have received a favourable opinion from an NHS REC:

- Work package 2.1A: A study of the views of people with cystic fibrosis about their condition and treatments (Hampshire A REC: 14/SC/1455; IRAS: 171049);

For peer review only - http://bmjopen.bmj.com/site/about/guidelines.xhtml

- Work package 2.1C: A study to produce videos for the CFHealthHub website (Camden & Kings Cross REC: 15/LO/0944; IRAS: 182367);

- Work package 2.2B: A study to develop a Behaviour Change Intervention (BCI) to help patients with CF manage treatment adherence ((South Yorkshire REC: 15/YH/0332; IRAS: 184477); and,

- Work package 2.2B(1): A study to understand how to use the eTrack and Bi-neb nebuliser to help people with CF to manage their inhalation treatments (West of Scotland REC 5: 15/WS/0089; IRAS: 177900).

Work package 3: Evaluate the toolkit developed in work package 2. The planned definitive evaluation will take place in a large-scale, multi-centre Randomised Controlled Trial (RCT). The definitive evaluation will compare usual care plus staff training in the importance of knowledge, skills and confidence building for adherence versus the same plus the structured behaviour change in intervention (CFHealthHub plus manual).

There is too little information available to effectively plan or justify funding a full scale RCT. We wish to conduct feasibility study comprising of:

- an ‘external pilot RCT’ to establish the feasibility of recruitment to a larger, definitive study; and,

- a ‘process evaluation’ which will help us understand the strengths and weaknesses of both the intervention and research protocols, and ways of addressing any weaknesses.

For peer review only - http://bmjopen.bmj.com/site/about/guidelines.xhtml

3. Aim and objectives

3.1 Aims

The principal aims of this feasibility study are to assess the feasibility and acceptability of:

- a complex intervention, when delivered outside the team which conceived and developed it; and,
- procedures for a full-scale RCT.

3.2 Objectives

1. An external pilot randomised controlled trial to determine feasibility of a randomised controlled trial based on objective stop-go criteria (Section 7.1) related to:

(a) participant recruitment;

(b) participant retention; and,

(c) quality of primary outcome data at 5(+/- 1) month.

2. A process evaluation, relating quantitative and qualitative data on procedures to outcomes, in order to understand and mitigate potential sources of failure in:

(a) the intervention; and,

(b) the full trial.

For peer review only - http://bmjopen.bmj.com/site/about/guidelines.xhtml

4. Design

Mixed-methods study comprising of:

- Quantitative component: parallel group, open labelled, external pilot RCT;

  and,

- Qualitative component: analysis of audio-recorded consultations and interviews.

Quantitative and qualitative data will contribute to the process evaluation.

For peer review only - http://bmjopen.bmj.com/site/about/guidelines.xhtml

5. Participants and study settings

5.1 Settings and locations where the data will be collected

Nebuliser adherence data and information derived from CFHealthHub will be automatically uploaded by participants nebulisers in their own home. Data collection involving patient notes and patient reported outcome measures will take place in two specialist CF units which have not been involved in the development of the intervention. Exacerbation data will be collected by the ACtiF trial interventionist and clinicians at sites from participant notes.

5.2 Eligibility

5.2.1 Inclusion criteria for participants

1. Diagnosed with CF and with data within the CF registry
2. Aged 16 years and above
3. Taking inhaled mucolytics or antibiotics via a chipped nebuliser (e.g. eTrack or Bi-Neb) or able and willing to take via eTrack or Bi-Neb.

5.2.2 Exclusion criteria for participants

1. Post-lung transplant
2. People on the active lung transplant list
3. Patients receiving palliative care, Lacking in capacity to give informed consent
4. Using dry powder devices to take antibiotics or mucolytics

5.2.3 Eligibility criteria for study centres

1. Adult CF Centre;
2. Recognised by commissioners
3. Receiving year-of-care funding

5.2.4 Eligibility criteria for interventionists

1. Health care professional e.g. registered nurse, physiotherapist or other appropriately skilled individual such as a psychology graduate able to work at NHS Agenda for Change Band - 4 or above

For peer review only - http://bmjopen.bmj.com/site/about/guidelines.xhtml

6. Interventions

6.1 Summary

In the external pilot RCT, we will test procedures for a full trial. This involves allocation of PWCF to either a complex intervention or usual care. A 'complex intervention' is defined as one with several interacting components [17]. The complex intervention under evaluation has three broad categories of components (Figure 1):

(a) ***a microchipped device*** (nebuliser) for delivering inhaled medications, which are routinely prescribed for the control of cystic fibrosis (Section 6.2);

(b) ***information technology infrastructure*** to capture and store adherence data from the nebulisers and display it to PWCF and the CF team (Section 6.3); and,

(c) ***the behaviour change intervention***, comprising a software platform ('CFHealthHub' mobile apps and website) offering adherence feedback and tailored modules of content and tools used by the health professional in interactions with PWCF (Section 6.4) and accessed independently by PWCF via CFHealthHub

Services received as usual care described in Section 6.5.

Figure 1. Interaction between complex intervention components

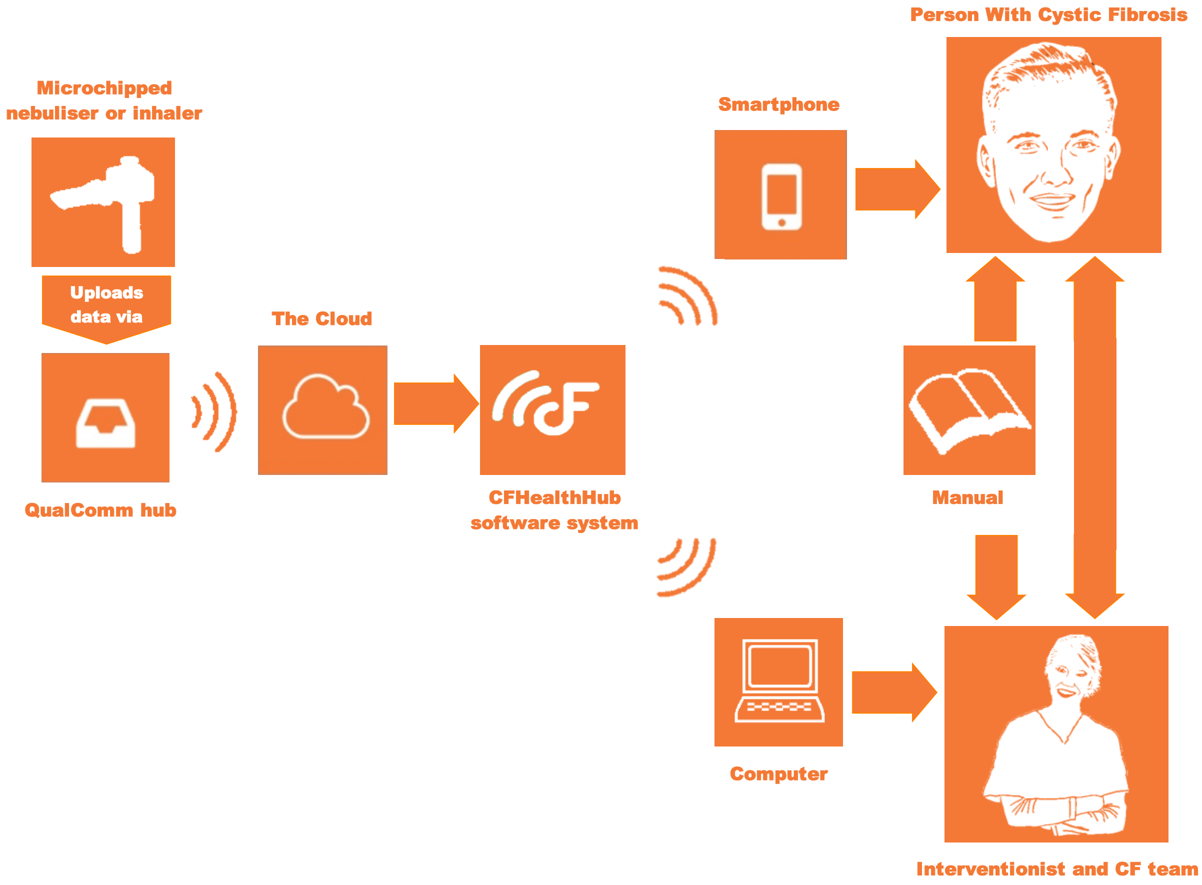

For peer review only - http://bmjopen.bmj.com/site/about/guidelines.xhtml

6.2 Microchipped devices

Depending on treatment strategies at different centres the participant may use an eTrack nebuliser system (Section 6.2.1), an Bi-neb AAD System from (Section 6.2.2).

6.2.1 The eTrack nebuliser system (Pari GmbH)

The eTrack controller is a modified version of the eBase controller and can be used to operate both the eFlow rapid nebulizer or Altera nebulizer. Compared to the eBase controller the eTrack is equipped with a Bluetooth chip and has a monitoring function to allow the capture of inhalation adherence data. The eFlow rapid nebuliser with eTrack controller is a CE marked medical device to be used for inhalation therapy. The device allows medications (approved for inhalation) to be transported deep into the lungs.

6.2.2 The Bi-neb AAD System from (Philips Healthcare)

The Bi-neb AAD system is a CE marked medical device which is intended for use to deliver aerosolised liquid medications for participants with cystic fibrosis. The drug delivery device is small and battery powered designed to deliver a precise dose of drug into patient’s lungs. The Bi-neb AAD system is designed to deliver liquid medications that are specifically approved for use with the Bi-neb AAD System.

6.3 Information technology infrastructure

The information technology infrastructure for the complex intervention comprises:

i. The Qualcomm hub (Section 6.3.1)
ii. CFHealthHub (Section 6.3.2).
iii. The Bi-Neb data transfer system (6.3.3)

6.3.1 The Qualcomm hub

The Qualcomm hub (Qualcomm; Cambridge, UK) is a wireless device which acquires data from the chipped device and transmits it to a cloud-based data centre. It is a Class I MDD and CE registered in Europe. It is designed, developed and manufactured in accordance with a quality system compliant with ISO13485 standards, meaning it aligns with the quality requirements of international regulatory agencies in the health care industry.

6.3.2 CFHealthHub

CFHealthHub is a web-portal which displays adherence data and provides resources and tools to people with cystic fibrosis and health professionals in order to support improved nebuliser adherence. It is available on-line via computers, tablets or mobile phones.

A qualitative study (WP 2.1A) to identify the barriers and facilitators of nebuliser use in PWCF informed the development of an intervention designed to increase nebuliser adherence. Analysis of the interview data was conducted using the COM-B framework, and these findings were used to inform the development of a complex intervention centred around the feedback of objective adherence data. The intervention was further developed and refined in consultation with PWCF and clinicians. An iterative study in which prototype versions of the intervention were delivered to and reviewed by PWCF was conducted. In that iterative study we interviewed PWCF and interventionists about the usability and tailoring of the

For peer review only - http://bmjopen.bmj.com/site/about/guidelines.xhtml

intervention, and made improvements to the process and materials based on this feedback. The system has been developed to ensure it meets the requirements of the Data Protection Act 1998. It is intended that data on maintenance and relapse will be generated during the full scale trial.

CFHealthHub has a number of modules addressing barriers to adherence based on the COM-B system described in greater detail in Section 6.4.1. The objectives of the modules as mapped to the COM-B are outlined in Table 1 below.

Table 1. Learning objectives of the CFHealthHub modules

COM-B model component	Objectives
Physical capability	- Have the skills to be able to use the nebuliser correctly
Psychological capability	- Understand the importance of nebuliser use in CF treatment<br>- Be able to remember to use nebuliser<br>- Be able to self-monitor nebuliser use<br>- Be aware of a need to improve nebuliser use
Physical opportunity	- Have a realistic medication plan<br>- Have a working/functioning nebuliser<br>- Have a suitable place to use nebuliser<br>- Have the time to use nebuliser
Social opportunity	- Be/feel supported by others to use nebuliser
Reflective motivation	- Perceive benefits of nebuliser use<br>- Perceive few/no concerns about nebuliser use<br>- Understand the health consequences of use/non-use<br>- Feel confident about nebuliser use<br>- Intend to use nebuliser
Automatic motivation	- Have an established routine for nebuliser use<br>- Have a habit to use nebuliser

6.3.3 The Bi-Neb data transfer system

The Bi-Neb Bluetooth data transfer system is intended to automatically extract breathing device use (adherence data) from the device (Bi-Neb) via a Smartphone hub and a secure data server onto CFHealthHub. Providing the Bi-Neb is within the Bluetooth range within the patient's house, the system can retrieve this data once a day.

For peer review only - http://bmjopen.bmj.com/site/about/guidelines.xhtml

6.4 The Behaviour Change Intervention (BCI)

6.4.1 Rationale and theory

The rationale of the BCI is to help CF patients to self-manage their condition and to form habits that will improve adherence to their medication, thereby extending life and improving quality of life. The MRC framework for developing and evaluating complex interventions recommends that intervention development should be informed by a suitable theoretical framework and evidence base [17]. The theoretical model adopted is the COM-B model [18] which describes a ‘behaviour system’ of the essential and interacting conditions of Capability, Opportunity, and Motivation [18] . The model posits that non-adherence is either non-intentional (a problem of capability or opportunity or intentional (a problem of motivation). The model has been adapted to nebuliser adherence on the basis of evidence about the factors influencing nebuliser adherence in PWCF [19–32], input from expert clinicians currently delivering services to PWCF, as well as from the PPI panel and exploratory research conducted in Sheffield. It is important that interventions are tailored to individual needs and use a multi-modal approach [33]. Each of the conditions of Capability, Opportunity and Motivation has been considered in turn in the development of our intervention. The primary component of the intervention is adherence feedback delivered via the CFHealthHub. Evidence suggests that while personalised feedback can have an effect size of up to 20% in increasing adherence [34, 35], feedback is most effective when combined with additional behaviour change techniques [34].

Figure 2. Interactions between capability, opportunity and motivation

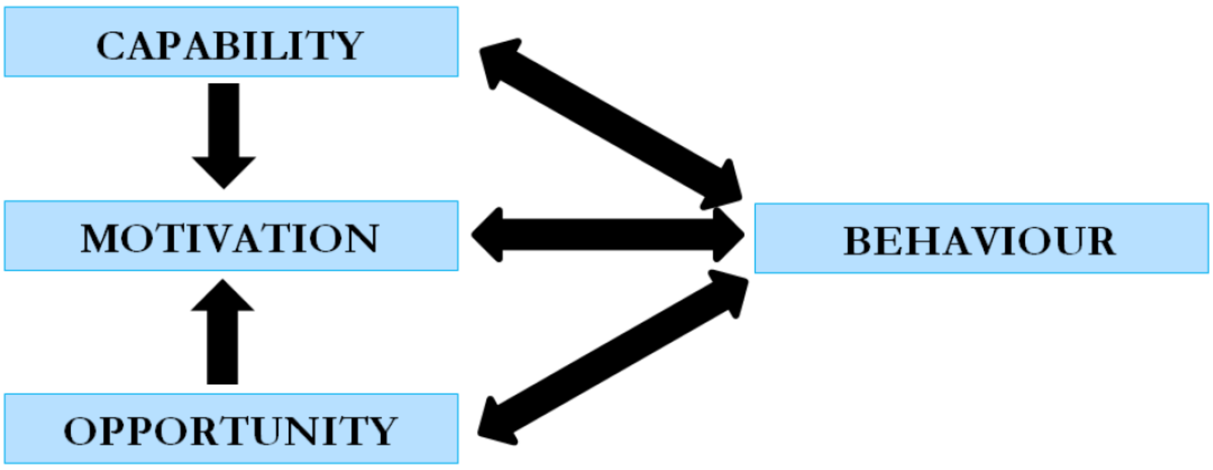

Figure 3. Habit formation incorporating COM-B and necessities and concerns

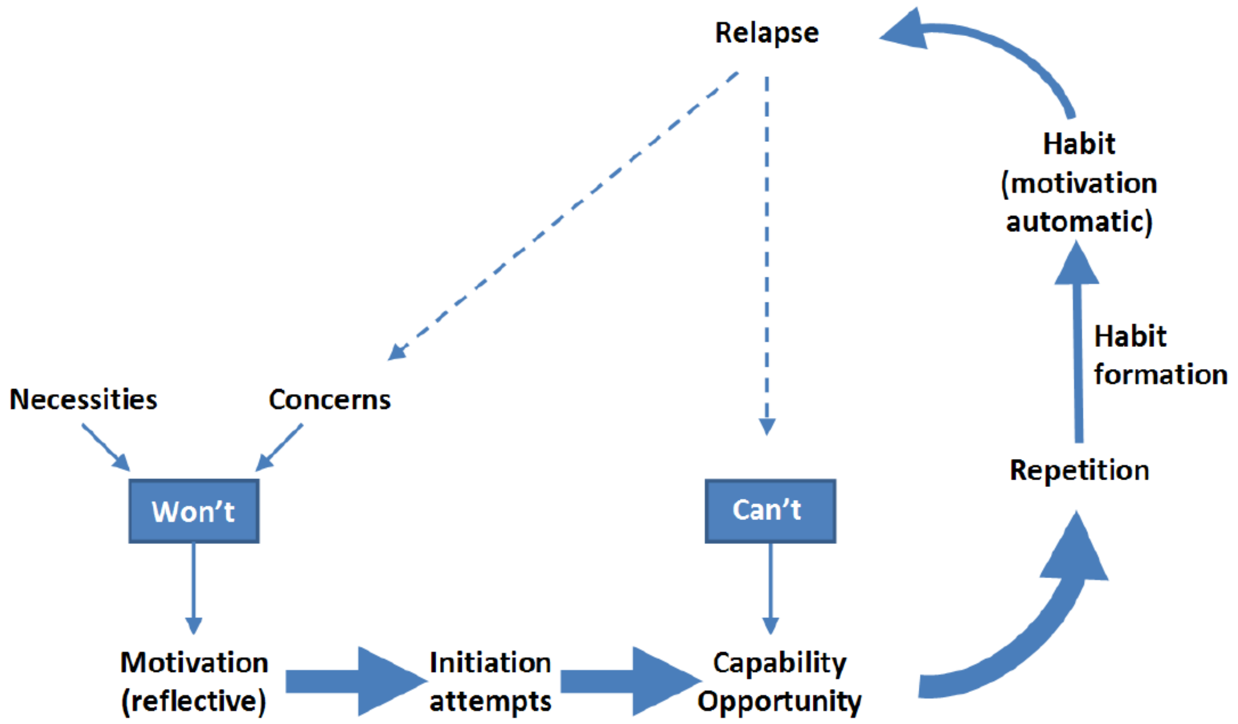

Figure 4. Interplay between COM-B components during habit formation

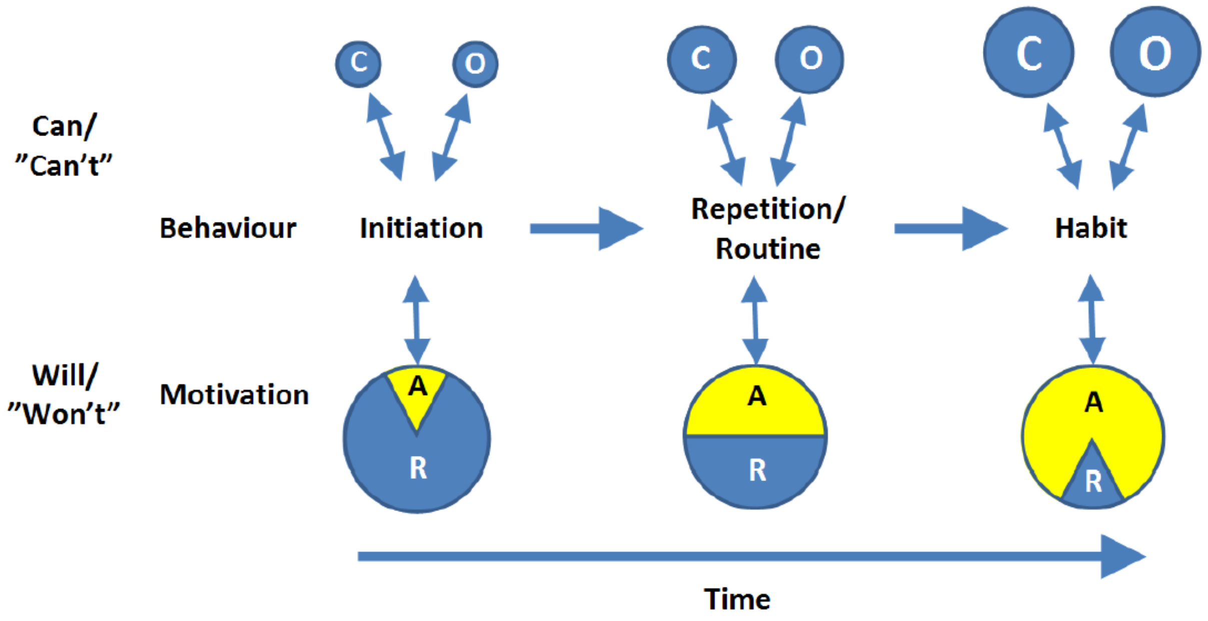

A= Automatic, R= Reflective, C= Capability, O=Opportunity

The identification and choice of appropriate behaviour change techniques has been driven by the Behaviour Change Wheel framework for the development of interventions [Michie, S. F., Atkins, L., & West, R. (2015). The behaviour change wheel: a guide to designing interventions.] which outlines a process of intervention design using the COM-B model "through the systematic evaluation of theory and evidence" (p. 13). In brief, the process involved the following steps:

1. In depth identification and analysis of the factors influencing nebuliser adherence in PWCF through an examination of the existing literature, and a qualitative study in which participants viewed charts of their objective nebuliser adherence data within an interview about factors affecting their motivation, capability and opportunity to adhere to their nebuliser treatment (study 2.1). The Theoretical Domains Framework (TDF; [36]) which analyses Capability, Opportunity and Motivation in greater detail was used as a framework to guide the analysis.
2. Identification and evaluation of potential intervention functions (e.g. education, persuasion, enablement, environmental restructuring, modelling) to address the identified factors influencing nebuliser adherence in consultation with the research team, clinicians and PPI.
3. Development of intervention modules to include specific Behaviour Change Techniques to deliver intervention functions, selection of mode of delivery, and mechanism for tailoring of BCI delivery to meet individual needs with regard to Capability, Opportunity and Motivation. The module contents have been discussed and refined as a result of discussions with clinicians and PPI.
4. Identification of potential mediators of behaviour change, and identification of tools to measure each mediator.

The intervention arrived at through this process is described in Table 2.

For peer review only - http://bmjopen.bmj.com/site/about/guidelines.xhtml

Table 2: Intervention modules

Module	COM-B	Intervention functions	Behaviour Change Techniques	Mode of Delivery
Universal parts of the intervention				
**Self-monitoring**	Psychological capability<br>Reflective Motivation	Education<br>Environmental restructuring<br>Enablement	• Self-monitoring of behaviour<br>• Adding objects to the environment (CFHealthHub)	• Charts of objective adherence data presented within CFHealthHub
**Goal setting & review**	Psychological capability<br>Automatic motivation	Enablement<br>Incentivisation	• Goal setting (behaviour)<br>• Feedback on behaviour<br>• Discrepancy between current behaviour and goal<br>• Review behavioural goals<br>• Graded tasks<br>• Social reward	• Discussion and agreement of goal with interventionist<br>• Review of goal<br>• Feedback on progress (through CFHealthHub and interventionist)<br>• Visual reward if goal met on CFHealthHub
**Treatment plan**	Psychological capability<br>Physical Opportunity Social Opportunity<br>Automatic motivation	Training<br>Environmental restructuring<br>Enablement	• Action planning<br>• Habit formation<br>• Prompts/cues (tailored)	• Action planning tool within CFHealthHub<br>• Option to set reminders
**Confidence building**	Reflective Motivation	Persuasion	• Focus on past success	• Interventionist encouraging focus on periods of higher adherence on charts

Module	COM-B	Intervention functions	Behaviour Change Techniques	Mode of Delivery
Tailored parts of the intervention (based on baseline COM beliefs and barriers questionnaire (COM-BMQ)[1] and consultation with interventionist)				
**My treatment**	Reflective Motivation<br>Psychological capability	Education<br>Persuasion<br>Modelling	• Information about health consequences<br>• Credible source<br>• Salience of consequences<br>• Demonstration of the behaviour<br>• Vicarious consequences<br>• Self-talk	• Q&A linked to information within CFHealthHub (tailored by baseline beliefs and prescription data)<br>• Presentation though text, patient stories, 'talking heads' and animation<br>• Credible sources including clinicians, PWCF and interventionist<br>• Interventionist eliciting self-talk through focus on why motivation is not lower than rating given on pre-screening questionnaire
**Confidence building**	Reflective Motivation	Modelling<br>Persuasion	• Demonstration of behaviour	• 'Talking heads' videos of coping stories within CFHealthHub
**Problem-solving (including skills training)**	Physical capability<br>Psychological capability<br>Physical opportunity<br>Social opportunity	Training<br>Environmental restructuring<br>Enablement	• Instruction on how to perform the behaviour<br>• Demonstration of the behaviour<br>• Behavioural practice/rehearsal<br>• Problem solving<br>• Restructure the physical environment<br>• self-talk<br>• social support (practical)	• Tailored problem solving guided by interventionist<br>• Solution bank within CFHealthHub.<br>• Construction of if-then coping plans<br>• Videos demonstrating correct use of nebulisers within CFHealthHub

1 Incorporating the Beliefs about Medicines Questionnaire (BMQ-specific nebuliser treatment) Horne, 2010

For peer review only - http://bmjopen.bmj.com/site/about/guidelines.xhtml

6.4.2 Intervention providers

Interventionists may already be working at, or be new to participating organisations or be the ACtiF interventionist employed to deliver the trial locally at the site. Externally appointed staff will be recruited through a formal job interview. Suitable individuals will include registered nurses or other member of the multidisciplinary team or a ; graduate in a suitable subject such as psychology or, other relevant profession who holds relevant skills / experience. Candidates for the post will ideally have a minimum of two years postgraduate experience which might include delivering a research project to time and target. They will be employed on the Project to work to NHS Agenda for Change Band 4 or above. They must have access to a car for work purposes e.g. participant home visits.

Interventionists will be supported in the delivery of the intervention by members of the Multidisciplinary team (MDT) at the site in which they are based. MDTs will receive training about the approach of the intervention, and the way in which they can support its delivery (see page 28).

Training for interventionists in how to deliver the intervention according to the specifications of the behaviour change manual will be provided by Marlene Hutchings with oversight provided by Madelynne Arden and/or Judy Bradley. A comprehensive training manual and training programme will be developed to facilitate this. A certificate of competence will be provided prior to the interventionist being able to use CFHealthHub with participants.

An additional trained regional interventionist will offer support to trial sites. This on occasion will involve input to patients (face to face or telephone contact), and assisting with problem solving via liaising with the nebuliser company. They will be named on the local site delegation log.

6.4.3 Materials

The BCI contains two broad categories of components:

i. CFHealthHub behaviour change modules including adherence feedback used by PWCF and health professionals

ii. The behaviour change manual and toolkit used by the interventionist in interactions with PWCF in order to understand the specific barriers to adherence for that individual, and to tailor and personalise delivery of the behaviour change modules accordingly.

6.4.4 Procedures

The BCI will be delivered over a 4 to 6 month period through a combination of face-to-face sessions and contact via telephone with an interventionist, and through participant interaction with different modules of content available on CFHealthHub. The interventionist will discuss participant data with members of the MDT to ensure that care is informed by objective adherence data. If any concerns become apparent as the interventionists collect data and work with participants, these concerns will be passed onto the clinical team. The clinical team will

For peer review only - http://bmjopen.bmj.com/site/about/guidelines.xhtml

follow their standard procedures in relation to any concerns raised. The intervention content and delivery flow are outlined in Figure 5 and described below:

6.4.4.1 Consent Visit (all participants)

At the consent visit participants will be given a chipped nebuliser (eTrack) and Qualcom hub or the participants will receive a visit from a clinical trainer who will convert the participant's I-neb to a Bi-neb by adding a Bluetooth chip and providing a Smartphone hub. The clinical trainer may set up the Bi-neb in the patient's home or at hospital either during the main consent visit or at a separate visit after consent has been obtained. Both the eTrack and Bi-neb will connect to CFHealthHub which will enable adherence data be collected. The interventionist will input the participant's prescription details into CFHealthHub. Together these will allow the system to generate adherence charts for that participant. At this visit participants will complete a range of baseline measures (see Table 3) including the COM beliefs and barriers questionnaire (COM-BMQ) which will be entered into CFHealthHub. The responses to this questionnaire will be used to populate the 'My toolkit' section of CFHealthHub with specific tailored elements from the 'My treatment' modules prior to the Initial Intervention Visit. The participant's pseudomonas status will be clarified at baseline and confirmed by the PI with the opportunity to compare the participant's prescription with the pseudomonas status.

6.4.4.2 Initial Intervention Visit (intervention arm only)

Participants will be introduced to CFHealthHub. They will be asked to complete an online consent form on behalf of their NHS trust in which they will specify what additional data they would be willing for CFHealthHub to record and display (e.g. name, and uploaded photographs) and what functional options they would like access to (e.g. push notifications). Permissions may be changed at any time. The participant will have the option to upload their own "patient story" into CFHealthHub after completion of the online consent form.

The interventionist will discuss their motivation to adhere to their nebuliser treatment, will address beliefs associated with poor adherence and will refer back to answers on the COM-BMQ to elicit the participants beliefs associated with adherence. Participants will be shown 'My toolkit' which will have been prepopulated with tailored motivational content (see consent visit).

The interventionist and participant will look at and discuss the adherence charts on CFHealthHub with a focus on period of higher adherence. The interventionist will note any barriers raised by participants during this discussion.

The interventionist will support the participant to identify where and when additional nebuliser treatments could be fitted into their schedule and support them to make an action plan using the online tool available on CFHealthHub. This action plan will be saved to the 'My toolkit' zone. The interventionist will then agree a % adherence goal for the next four to six weeks based on the number of additional treatments that have been planned. This will be recorded on CFHealthHub and will be represented by a target line on the adherence charts.

If motivation is so low that participants are reluctant to set an action plan/goal then the interventionist will spend further time discussing motivation and will skip to confidence building (see below).

The interventionist will encourage participants to focus on likely problems or issues that might disrupt the achievement of the adherence goal and will use the Problem-solving module on CFHealthHub to address each of these anticipated problems. The Problem-solving module includes solutions based on educational content, practical support (e.g. model letters to

For peer review only - http://bmjopen.bmj.com/site/about/guidelines.xhtml

employers) and interactive tools. Relevant solutions will be saved to the 'My Toolkit' zone of CFHealthHub.

The interventionist will discuss the participant's confidence to meet their goal and will identify 2-3 'talking heads' videos showing other people with CF addressing and overcoming similar barriers to nebuliser adherence.

The visit will conclude with a review of the goal and the tailored and personalised contents saved to the 'My toolkit' zone of CFHealthHub. The interventionist will encourage a learning mindset, emphasising that even if adherence doesn’t increase starting to think about adherence will produce learning that will make subsequent attempts to change easier.

6.4.4.3 Participant Independent access to CFHealthHub (intervention arm)

Participants will have independent access to CFHealthHub at all times following the Baseline visit. They can, at any time, access their adherence charts, 'My toolkit' contents, and can browse the other areas of content as they wish. Frequency of access to each area of CFHealthHub will be monitored and recorded.

Adherence charts will provide colour -coded feedback about participant achievement towards their adherence goal so that they are provided with immediate, easy to recognise information about their achievements. Subject to consent, participants will be sent encouraging messages via push notifications, or alternatively when they access CFHealthHub, to match the progress made e.g. congratulations on achieving their goal, congratulations on having made progress towards their goal, encouragement to remember their action plan.

6.4.4.4 Review visit (Visit 3 - intervention arm)

At the review visit, the interventionist and participant will look at and discuss the adherence charts on CFHealthHub and goal achievement with a focus on progress made and periods of higher adherence.

If the adherence goal was met then the participant will be encouraged to set a new higher adherence goal or to a goal to maintain their current level of adherence which will be recorded on CFHealthHub. Following this the participant and interventionist will review the contents of 'My toolkit' and revise action plans, problems/solutions as required. If issues of motivation are still a concern the interventionist may recommend additional/alternate elements of content from 'My treatment' or 'Talking heads' to go into 'My toolkit'.

If the adherence goal was not met then the interventionist and participants will discuss the barriers to goal achievement (motivation, capability, opportunity). The interventionist will address beliefs associated with poor adherence and will add/revise the elements of content from 'My treatment' or 'Confidence building' to go into 'My toolkit'.

If no goal was previously set then the interventionist will review motivation and confidence and then will consider if the participant is ready to action plan and set a goal. If not they will spend more time reviewing motivation and confidence.

The participant will be encouraged to set a realistic % adherence goal for the next four to six weeks and this will be recorded on CFHealthHub. The interventionist will support the participant to revise their action plan as needed and save this to the 'My toolkit' zone. Based on the earlier discussion about the barriers that prevented goal achievement the Problem-solving module on CFHealthHub will be used to address each of the problems encountered, and any that are anticipated. Relevant solutions will be saved to the 'My Toolkit' zone of CFHealthHub.

The visit will conclude with a review of the goal and the tailored and personalised contents saved to the 'My toolkit' zone of CFHealthHub. The interventionist will re-emphasise a

For peer review only - http://bmjopen.bmj.com/site/about/guidelines.xhtml

learning mindset, emphasising that the participant cannot fail, but can learn from the process so that they can work together on the adherence challenge.

Participating centres will provide participants with contact details, typically telephone numbers, but other methods may be volunteered by centres. Contact details will be provided so that participants can contact the centre if they have queries or problems regarding CFHealthHub between visits. The interventionist will be able to feedback any information from the intervention delivery **after** the baseline intervention visit to members of the wider CF team. This may include adherence data from sessions with the participant's clinician and MDT particularly if the participant raises any concerns or issues e.g. side effects of a drug to allow their usual clinician to discuss this with them at their next clinic visit.

6.4.4.5 Subsequent Review (intervention arm)

Following these two sessions the amount of interaction which each PWCF has with the interventionist will be tailored to their needs and requirements although it is anticipated that these will normally marry with routine clinic visits: They may have additional face-to-face sessions or contact via telephone or e-mail. No more than one monthly face-to-face session will be conducted because of the research protocol; if the participant requests additional support, the centre may accommodate this at their discretion. Review meetings will take 30 minutes and be conducted over the 5month (+/- 1 month) of the follow-up period. The structure of review sessions will follow the same pattern as for 6.4.4.4.

6.4.4.6 Final research visit (5 months +/- 1 month from consent)

All participants will complete a final research visit 4-6 months from the date of consent. At this visit the interventionist will collect the primary and secondary outcome data (see table 3) including demography data, health care resource use and the participant completed questionnaires. At this final research visit the interventionist will re-check that all adherence data has been transferred to CFHealthHub. The eTrack can store approximately 6 months of treatment data, ensuring all the data is transferred at this visit should help to prevent missing data.

Following the final 4-6 month post-consent research visit, we will continue to collect: adherence data from CFHealthHub; exacerbations; FEV1 and ask participants the subjective adherence question until, 30th April 2017. At this point the study closes and the involvement of all participants ceases. After the trial ends (30/4/17), the aspiration is to allow participants in the control to have access to the intervention for which negotiations are ongoing. Currently funding is in place for the trial interventionists at study sites to deliver the intervention only over a 12-month period i.e. up to 30/4/17. It is anticipated that CFHealthHub used outside the trial would be delivered within the existing resources of the MDT so using CFHealthHub outside the trial should not need the trust to employ any additional staff members. As this is a pilot feasibility study where we are testing the intervention in participants, there is an expectation that further iteration of CFHealthHub may occur.

For peer review only - http://bmjopen.bmj.com/site/about/guidelines.xhtml

Figure 5. Behaviour change intervention flow chart

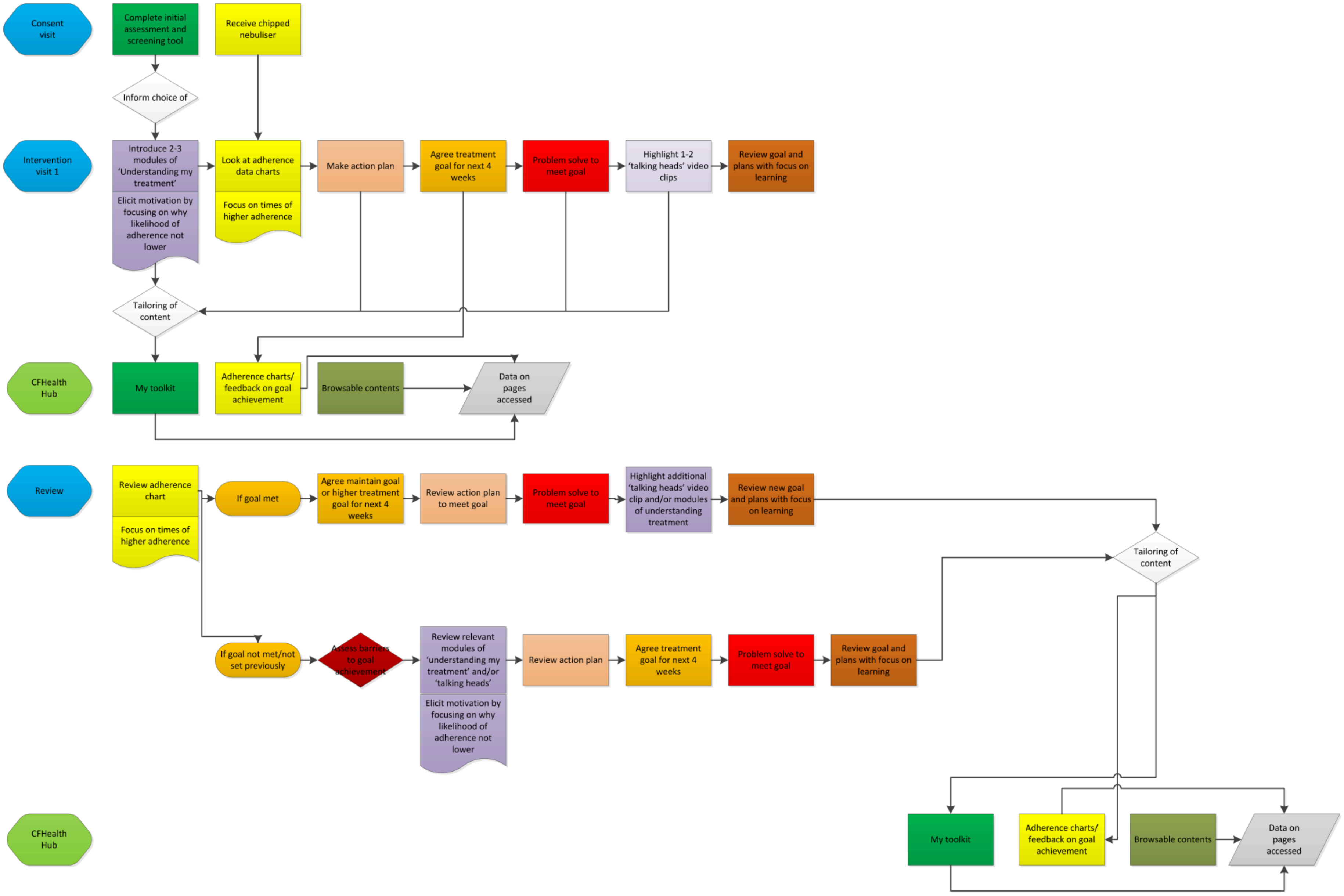

6.5 Usual care

Patients in both arms will receive usual care. Usual care is heterogeneous within and between centres, based on the needs of patients and the skills and interests of CF Unit staff. To better understand the configuration of usual care at participating centres a survey tool will be administered by the CTRU to the lead clinician at the centre. This will identify the spectrum of clinical and behaviour change interventions that are in use in the management and self-management of CF.

A minor component of the intervention is to train all members of the MDT in awareness of patient activation so that they are open to addressing issues raised for PWCF in the intervention arm. In addition, a staff member in the MDT will help to deliver the intervention. There is the possibility that the awareness of patient activation will have some effect on PWCF in both the intervention and control arms, and of leakage of the learning from the behaviour change component of the intervention to controls. We will investigate this possibility during the process evaluation.

Members of the MDT at each centre will receive one half-day, on-site, face-to-face training about the importance of objective nebuliser adherence data in the management of CF, and awareness of the importance of building patients' knowledge, skills and confidence to enable them to self-manage their treatment. This will include training in the interpretation of graphs and charts of objective adherence data produced by CFHealthHub, and the rationale for reducing target adherence in poor adherers in order to increase confidence. This will be delivered by designated members of the ACtiF research team.

Participants in the control arm will use a microchipped nebulizer but will not be able to access adherence data or other content and tools through CFHealthHub, neither will they receive the structured CFHealthHub intervention as described in the intervention manual. Control arm participants using Bi-neb nebulizers might have access to their data as part of routine care but this will not be in the user friendly format provided by the intervention.

One function of the qualitative research interviews with staff and control participants (see Section 8 below) is to understand the extent to which the patient activation awareness training has affected staff behaviour and whether control arm participants have received some aspects of the behaviour change intervention.

6.6 Criteria for discontinuing or modifying allocated interventions

There are no criteria for discontinuing treatment. Participants will be made aware that their participation is voluntary and they may discontinue study interventions, should they wish, at any time.

If a participant wishes to withdraw from treatment they will be able to speak to a member of the site study team i.e. ACtiF interventionist. This will be documented on a participant withdrawal form, within the Case Report Form. Any data already collected during the course of the trial up to the point of withdrawal will be used in the final analysis. We will ask the participants for their permission to continue to collect the primary outcome data i.e. CF exacerbations. The participant or clinician can make the decision to discontinue the allocated study intervention for any reason.

Participants will have the following options if they wish to withdraw:

1. Withdraw from the intervention i.e. intervention delivery visits only but will remain in the study. Patients can continue to use CFHealthHub. All study data would continue to be collected at subsequent follow up time points as per protocol.
2. Withdrawal from the study. Unless the patient objects, any data collected up to this point would be retained and used in the study analysis. The local interventionist would ask the participant if they agree to the collection of primary outcome data as defined in the protocol and or adherence data If

they agree to collection of adherence data, CTRU and or interventionist will continue to follow up participants for adherence data.

3. Withdrawal from the trial entirely. Unless the patient objects, any data collected up to this point would be retained and used in the study analysis. If the patient does not wish to be contacted with regard to primary outcome data or adherence data, no further contact with regard to the study will be made. If the participant does specifically request for all their data to be removed information regarding the participant will be retained at site, as part of the patient notes, along with their withdrawal form and request to delete the data.

A participant would be classed as complete if they have continued in the study until the last protocol defined visit, however there may be missing visits and / or data.

Loss to Follow-Up

A participant would be classed as lost to follow up if the participant has 1) not completed the study or 2) been withdrawn despite attempts for further contact, as per protocol, having been made. Unless the participant withdraws from the study entirely we will continue to collect the primary outcome data when possible (i.e. from medical notes).

This withdrawal section has been developed in accordance with the CTRU Participant Discontinuation and Withdrawal of Consent Standard Operating Procedure (SSU003).

6.7 Strategies to improve adherence to intervention protocols

6.7.1 For health professionals

The intervention protocols will be described in detail in an intervention manual. Interventionists will be trained to deliver the intervention according to the manual protocols. Interventionist training (as a form of behaviour change) will focus on Capability, Opportunity and Motivation. It will utilise evidence about the importance and likely effectiveness of the intervention and will challenge common misconceptions about adherence. Skills training and an introduction to the tools available on CFHealthHub will increase staff capability, and we will work with clinics and clinicians to ensure that the practical requirements for intervention delivery are in place: space, time etc (opportunity).

CFHealthHub will record interventionist access to the site. It will also automatize some of the tailoring of the intervention according to the COM-BMQ which will be completed online. The contents of 'My Toolkit' will be recorded for each participant so that we will have records of what content they have been recommended. Interventionists will also be required to complete session records each time that they deliver the intervention to record the decisions made and the reasons for these,

6.7.2 For patients

Where participants provide consent we will send optional push notifications to encourage engagement with CFHealthHub. For example, we will send congratulatory messages when adherence improves, encouraging messages to remind participants to engage with the content. Face-to-face visits will, where possible be arranged to coincide with clinic visits as per usual care, therefore minimising the additional burden on participants.

6.8 Relevant permitted / prohibited concomitant care

No concomitant care will be denied based on the research protocol.

For peer review only - http://bmjopen.bmj.com/site/about/guidelines.xhtml

7. Outcomes

7.1 Feasibility outcomes ('stop-go' or 'success' criteria for RCT)

In line with proposed CONSORT extension for pilot studies [37], in this section, we state the criteria for success of the external pilot trial. The criteria are based on the primary feasibility objectives, which provide the basis for interpreting the results of the external pilot and for determining the feasibility of proceeding to the full-scale study scheduled for months 31 to 60 of the project. Depending on the funder's perspectives, the outcome of the external pilot might be:

(i) "Stop - main study not feasible";
(ii) "Continue, but modify protocol - feasible with modifications";
(iii) "Continue without modifications, but monitor closely - feasible with close monitoring"; or,
(iv) "Continue without modifications - feasible as is."[37]

We anticipate that modifications to the research protocol will be necessary as the feasibility study progresses. Some of the qualitative research will be undertaken early in the pilot trial and lessons learned about the trial procedures will be identified and acted on during the pilot trial. There are three objective stop-go criteria:

1. Feasibility of recruitment to RCT
Defined as recruitment of no fewer than 48 participants randomised at two centres over four months, 75% of the rate required in the main trial;

2. Feasibility of retaining participants in the RCT
Defined as attrition from the research protocol of no more than 15% of randomised participants at 5 (+/-1) months.

If these are met the full trial will go ahead. If these are not met overall, but are met in the last half of the pilot trial after trial procedures have been improved based on lessons learned from the early stage of the pilot trial, then the full trial will go ahead.

7.2 Process data relating to the implementation of the trial

1. Number and characteristics of eligible patients approached for the study
Collected by centres in screening logs and transferred to Prospect database

2. Reasons for refused consent
Collected by centres in screening logs and transferred to Prospect database.

3. Reach
How many participants are consented into the study, sub-grouped by socio-economic status (from CF Registry), as a proportion of:

- Those approached, expressed quantitatively, based on 'pre-screening' logs completed by ACtiF interventionist;
- Those known to be eligible, expressed quantitatively based on CF Registry.

4. Participant attrition rate
Collected by centres in screening logs and transferred to Prospect database.

For peer review only - http://bmjopen.bmj.com/site/about/guidelines.xhtml

5. Reasons for attrition

Collected by centres in screening logs and transferred to Prospect database.

6. Maintenance:

The processes by which participants are kept involved in the collection of key secondary outcome data research data:

- The extent to which adherence data is successfully uploaded from the chipped nebulisers , described quantitatively using CFHealthHub (Intervention arm only).

7. Number of missing values/incomplete cases

Assessed by data management team, based on data in Prospect database.

8. Participant,/interventionist and members of MDT views on research protocols

Assessed through qualitative interviews and to include:

- Barriers to recruitment, problems encountered in reaching participants [38];
- Perceived problems with trial procedures such as recruitment, informed consent etc.
- Acceptability
- Perceived utility and burden of outcome assessments.

9. A survey on the content of usual care at participating centres

A CTRU staff member will complete this survey with the principal investigator, a senior medic or delegate working at the participating centre.

7.3 Process data relating to the implementation of the intervention

1. Context

Definitions of 'context' tend to cluster around setting, roles, interactions and relationships [39]. It is important that context is understood as diachronic and emergent rather than synchronic and static [40, 41]. Frameworks for process evaluation have defined 'context' as:

- "aspects of the larger social, political, and economic environment that may influence intervention implementation" [42];

- "factors external to the intervention which may influence its implementation, or whether its mechanisms of impact act as intended" [43].

The context, and its interaction with implementation, mechanisms of impact, outcomes, the description of the intervention and its causal assumptions [43] will be described using qualitative data from research interviews, field notes, study management logs, minutes and e-mails. The focus will be how the context of individual CF Units affects implementation of the intervention and its potential outcomes.

2. Implementation

Definitions of 'implementation' tend to cluster around the processes or stages of adoption, the methods, means or social organisation of bringing innovative practices into use [39]. One way of describing the process of getting research into practice is to use a process model [44]. To structure our narrative of how the complex intervention was implemented we will use a process model called the Quality of Implementation Framework [45].

3. Recruitment:

Based on e-mails and minutes we will describe in narrative terms, the procedures used to approach and attract to the project NHS Trusts and interventionists [42].

For peer review only - http://bmjopen.bmj.com/site/about/guidelines.xhtml

4. Training:
The comprehensiveness of the training component of the intervention for the health professionals delivering the intervention will be assessed by a combination of audio recordings of consultations and by interview.

5. Fidelity
"The extent to which the intervention was delivered as planned. It represents the quality and integrity of the intervention as conceived by the developers. Fidelity is a function of the intervention providers."[42]

- Interaction with participant along lines recommended by manual, determined by audio recordings of consultations between the interventionist and PwCF in the intervention arm.

- Recommendation of appropriate CFHealthHub tasks by interventionist, determined by audio recordings and by data from CFHealthHub;

The fidelity assessment will be developed and based on a tool used by Borelli et al [46].

6. Use [38] ***/ dose received*** [42] ***of intervention***
Use of CFHealthHub by participant, as proposed by interventionist, determined by data capture by CFHealthHub, including the online activities started and completed, minutes spent on recommended pages and which parts the participant has picked out and put in a "my favourites" page. The number of times, frequency over time and duration with which users log on to CFHealthHub, as well as the activities they perform while logged in, described quantitatively using data from CFHealthHub.
A record of the discussion between the interventionist and the MDT will be kept. This will include who was there, brief notes of what was discussed and any agreement of treatment goals made.

7. Acceptability
The acceptability of the intervention to hospital staff and PWCF assessed through semi-structured interviews.

8. Perceived benefits and harms
Assessed through semi-structured interviews with health professionals and PWCF.

9. Leakage of intervention to controls
Assessed through audio recordings of consultations between the MDT, interventionist, and PwCF in the control arm, and semi-structured interviews with PwCF in the control arm.

7.4 Clinical outcomes and covariates

The time schedule of enrolment, interventions (including any run-ins and washouts), assessments, and visits for participants can be found in Table 3 and Table 4 below.

7.4.1 Primary clinical outcome

The primary clinical outcome is the number of pulmonary exacerbations in 5 (+/-1) month post-baseline follow-up period, defined according to the Fuchs criteria [47]. An exacerbation of respiratory symptoms will be said to have occurred when a patient was treated with parenteral antibiotics for **any one of the following 12 signs or symptoms** [48]**:**

1. change in sputum;
2. new or increased hemoptysis;
3. increased cough;
4. increased dyspnea;
5. malaise, fatigue, or lethargy;

For peer review only - http://bmjopen.bmj.com/site/about/guidelines.xhtml

6. temperature above 38 °C;
7. anorexia or weight loss;
8. sinus pain or tenderness;
9. change in sinus discharge.
10. change in physical examination of the chest, derived from notes by site staff.
11. decrease in pulmonary function by 10 percent or more from a previously recorded value, derived from notes by site staff; or,
12. radiographic changes indicative of pulmonary infection, derived from notes by site staff.

The trial interventionist or prescribing clinician/nurse will collect data on the "exacerbations" form at the point of a participant starting a course of IV antibiotics.

7.4.2 Secondary clinical outcomes

1. **Body Mass Index (BMI).**

2. **Forced expiratory volume in 1 second (FEV_1):** standardised spirometry as a measure of condition severity [49].

3. **EuroQol EQ-5D-5L**: generic health status measure for health economic analysis [50].

4. **The Patient Activation Measure (PAM-13) (Health Style Assessment)**: assessment of patient knowledge, skill, and confidence for self-management [51]. *PAM-13 was labelled as "Health Style Assessment" following a request from the licence owners to ensure the purpose of the questionnaire is clear for participants.

5. **Assessment of routine :** measure of life chaos [52].

6. **Self-Report Behavioural Automaticity Index (SRBAI):** automaticity-specific subscale of the Self Report Habit index to capture habit-based behaviour patterns [53].

7. **Cystic Fibrosis Questionnaire-Revised (CFQ-R):** disease specific health-related quality of life instrument [54].

8. **The Patient Health Questionnaire depression scale (PHQ-8):** severity measure for depressive disorders [55].
9. **MAD (Medication Adherence Data-3 items) :** medication adherence measure

10. **The General Anxiety Disorder 7-item anxiety scale (GAD-7):** severity measure for anxiety [56].

11. **The Capability Opportunity Motivation Behaviour Beliefs Questionnaire (COM-BMQ):** This questionnaire incorporates:

    a. **The Beliefs about Medicines Questionnaire - specific (Nebuliser adherence) (BMQ 21-item):** a validated self-report tool[57], customised by the author to identify perceived necessities and concerns for nebuliser treatment.
    b. The following project-specific items: one additional belief item, one intention item, one confidence item, and a list of barriers. These will serve as a tailoring tool for the intervention and also as a secondary outcome measure.

12. **Subjective adherence single question:** self-report estimate of adherence as a percentage. Self-reported problems: identification of capability and opportunity barriers to nebuliser adherence

13. **Concomitant medications:** bespoke instrument, designed for this research project.

14. **Resource use form:** interventionist collects data from a combination of hospital notes and the NHS patient electronic system to determine 1) inpatient IV days 2) Routine clinic visits 3) Unscheduled outpatient contacts 3) unscheduled inpatient stays.

15. **Exploratory analysis of habit formation**: analyses with the objective nebuliser data will be performed to explore the process of habit formation with the delivery of the adherence intervention

16. **Prescription**: a monthly prescription check to both check for data transfer to CFHealthHub and review for an indication that the prescription has changed or indication of microorganism e.g. pseudomonas (please see table 2 and 3 and refer to section 10.1.1).

17. **Adherence to prescribed medication (see 7.4.3)**

18. **Any treatment with IV antibiotics**

7.4.3 Adherence to prescribed medication

Adherence to prescribed medication will be defined in several ways including:

1. Unadjusted adherence
2. Simple normative adherence (without numerator adjustment)
3. Sophisticated normative adherence (without numerator adjustment)
4. Simple normative adherence (with numerator adjustment)
5. Sophisticated normative adherence (with numerator adjustment)

Further detail about the outcomes will be reported in the trial statistical analysis plan.

For peer review only - http://bmjopen.bmj.com/site/about/guidelines.xhtml

Table 3. Individual-level data derived from PWCF and sites

	Where?	Completed by?	Consent visit	Baseline (intervention) visit	At clinic visits	Exacerbations episode	5 months (+/- 1 month) from consent visit	Up to 30th April 2017
**Enrolment**								
Pre-screening form (before 1st visit)	Prospct	Site	-	-	-	-	-	-
Confirmation of eligibility form	Prospct	Site	●	-	-	-	-	-
Informed consent	Prospct	Site	●	-	-	-	-	-
Intravenous days in last registry year	Prospct	Site	●	-	-	-	-	-
Pseudomonas status +	Prospct	Site	●	-	-	-	-	-
**Primary outcome**								
Exacerbations form including:<br>Parenteral antibiotics<br>Change in sputum*<br>New or increased hemoptysis*<br>Increased cough*<br>Increased dyspnea*<br>Malaise, fatigue, or lethargy*<br>Temperature above 38 °C*<br>Anorexia or weight loss*<br>Sinus pain or tenderness*<br>Change: sinus discharge*<br>Change: phys. exam. chest*<br>Decrease: pulmonary function *<br>Indicative radiographic changes*	Prospct	Site	●	-	-	●	●	●
**Secondary outcomes**								
BMI (height and weight)	Prospct	Site	●	-	-	-	●	-
FEV_1	Prospct	Site	●	-	●	-	●	●
EQ-5D-5L**	Prospct	PWCF	●	-	-	●	●	-
PAM-13(Health Style Assessment)	Prospct	PWCF	●	-	-	-	●	-
Assessment of Routine	Prospct	PWCF	●	-	-	-	●	-
SRBAI	Prospct	PWCF	●	-	-	-	●	-
CFQ-R	Prospct	PWCF	●	-	-	-	●	-
PHQ-8	Prospct	PWCF	●	-	-	-	●	-
GAD-7	Prospct	PWCF	●	-	-	-	●	-
MAD-3 (Medication Adherence Data-3 items)	Prospct	PWCF	●	-	-	-	●	-
COM-BMQ	Prospct	PWCF	●	-	-	-	●	-
Objective adherence	CFHH	CFHH	●	-	●	-	●	-
Subjective adherence single question	Prospct	PWCF	●	-	●	-	●	●
Concomitant medications	Prospct	Site	●	-	-	-	●	-
Other SAEs	Prospct	Site	-	-	●	-	●	-
Resource use	Prospct	Site	-	-	-	-	●	-

+ Pseudomonas (or other microorganism) status will be checked together with the monthly prescription
* Only required where PWCF indicates they have received parenteral antibiotics

For peer review only - http://bmjopen.bmj.com/site/about/guidelines.xhtml

** EQ5D-5L collected at the start and end of every exacerbation episode

Table 4. CFHealthHub data (research arm only)

	Completed by?	Baseline (intervention) visit	At intervention visit s with interventionist	Between sessions	At clinic visits	5 months (+/- 1 month) from consent visit	Up till 30th April 2017
**Clinician metrics**							
Adherence data*	PWCF	●	●	●	●	●	●
Recommendation of modules by interventionist	Interventionist	●	●	-	●	-	x
Feed back to participant their adherence data screens (data click)	Interventionist	●	●	-	●	-	x
Check prescription with participant	Interventionist	●	●	-	●	-	x
Order of clicks	CFHH	●	●	-	●	-	x
Interventionist responds to patient changing prescription	Interventionist	-	●	●	●	●	x
Monthly check on prescription +	Interventionist / CTRU	●	●	●	●	●	x
Time in and out preparation	Interventionist /CFHH	●	●	-	-	●	x
Time in and out with patient	Interventionist /CFHH	●	●	-	-	●	x
Time in and out review	Interventionist /CFHH	●	●	-	-	●	x
**Patient metrics**							
Adherence (number of nebulized doses taken per day.) [1]	PWCF	●	●	●	●	●	x
Duration of inhalation	Nebuliser	●	●	-	-	-	x
Accessing CFHealthHub – look at adherence data	PWCF	●	●	-	-	-	x
Accessing CFHealthHub – look at ‘My Toolkit’	PWCF	●	●	-	-	-	x
Accessing CFHealthHub problem solving / education / talking heads pages outside of ‘My Toolkit’	PWCF	●	●	-	-	-	x
Accessing CF HealthHub – first to last click in a session	PWCF	●	●	-	-	-	x

*Adherence data collected for both research and control arms
+ Monthly prescription checked by CTRU centrally to alert local interventionists to any potential changes in control arm and potentially also intervention arm
X data continued to be collected in CFHealthHub and interventionist responds for those participants who have “opted in” to receive intervention till 30/4/17
[1]To be broken down in statistical analysis plan.

For peer review only - http://bmjopen.bmj.com/site/about/guidelines.xhtml

Figure 6. Participant timeline for the external pilot RCT

Enrollment

Identification and approach, by letter, following up with phone call (pre-Consent form) :

1) PWCF identified through the CF registry;

2) Screened for eligibility (pre-screening tool):

Inclusion: PWCF who have data within the CF registry aged > 16 years & taking inhaled mucolytics or antibiotics via a chipped nebuliser (e.g eTrack or I-Neb) or able and willing to take via eTrack or I-Neb.

Exclusion: PWCF who are post-lung transplant or on the active lung transplant list, patients receiving palliative care, with palliative intent, for whom trial participation could be a burden, patients lacking in capacity to give informed consent or using dry powder devices to take antibiotics or mucolytics.

3) Direct care team 1) Sends potential participant PIS and introductory letter by post and Interventionist follow's up by phone within 1 week to ensure they have received the information and allow an opportunity for questions AND/OR **2)** Direct care team gives patients written information during routine clinic visit and asks permission to follow up the patient with a phone call to answer further questions and discuss involvement.

↓

CONSENT VISIT, at home or in clinic:

1) Screening form completed (confirmation of eligibility)

2) Informed consent.

3) Data collection: Intravenous days in last registry year; BMI; FEV1; EQ-5D-5L; PAM-13; CHAOS; MAD-3; SRBAI; CFQ-R; PHQ-8; GAD-7; COM-BBQ;Exacerbations form; Concomitant medications; subjective adherence question

4) Participant given data logging nebuliser and Qualcomm hub.*

↓

Allocation

Randomisation (n~64):
ACtiF interventionists randomises using web-based system between consent and baseline visits and informs participant ideally within 1 week of consent visit.

Intervention arm
Research: Monitoring and feedback (n=32):
Initial intervention visit: at home or in clinic (2-6 weeks from consent visit) (1 hour)

1) Adherence data uploaded, fed back and reasons for suboptimal adherence discussed in order to identify problems to solve using the Adherence Support Process;
2) Intervention chosen based on barrier from "library" in the Adherence Support Process;
3) Supportive contact offered via phone or email; and,
4) Participants given personal access to cfhealthhub data.

Manualised intervention delivered by trained researcher in initial intervention visit and subsequent visits normally every 4-6 weeks from this initial visit till six month visit (see below)

Control arm
Usual care n=32:
Adherence data uploaded. Care team and PWCF remain blind to adherence data.

Follow-up

ACtiF Researcher collects exacerbation data
Data collected: Exacerbations form triggered by commencement of IV antibiotics; EQ5D-L at both start and end of exacerbation episode

Routine Clinic Visits:
Data collected from all routine clinic visits after the date of consent.
Data collected by ACtiF researcher except where specified: (i) FEV1; (ii) subjective adherence question (iii) exacerbations form.
Adherence data continued to be uploaded to CFHH.

Monthly follow up by CTRU researcher and/or ACtiF interventionist
Monthly check of adherence data and contact with participant where missing adherence data or requirement to follow up on changes to prescription

↓

5 month (+/- 1 month) visit
Primary clinical outcome: Number of exacerbations (Fuchs criteria)
Secondary outcomes: BMI; FEV1; EQ-5D-5L; PAM-13; CHAOS; MAD-3; SRBAI; CFQ-R; PHQ-8; GAD-7; COM-BBQ;Exacerbations form; Concomitant medications; Other SAEs, resource use questionnaire

*When I-nebs are converted to Bi-nebs a representative from the company (Philips) will do this between the consent visit and first intervention visit.

The study recognises that flexibility in accommodating participant schedules may cause time windows to change but this will allow us to adapt the intervention for the main RCT.

8. Sampling

8.1 Quantitative components

8.1.1 Sites

Two large specialist CF centres have been screened for their ability to recruit participants based on the number of participants they have on their CF registry and their motivation to participate in the pilot trial.

8.1.2 Sample size

The sample size for a feasibility study should be adequate to estimate the uncertain critical parameters (standard deviations for continuous outcomes; consent rates, event rates, attrition rates for binary outcomes) needed to inform the design of the full RCT with sufficient precision [37, 58–60]. For the main RCT, the target sample size is 688 participants (344 per arm). We are proposing that 15 CF units recruit on average 46 patients in six months, a recruitment rate of approximately eight patients per centre per month.

To assess whether this recruitment rate is feasible the external pilot RCT will open in two CF units for 12 months, with four months recruitment, one months 'run-in' period (the period between the consent and baseline visit), and 5 (+/-1) months follow up. To match the proposed recruitment rate of the main RCT, the target sample over the four months for which the pilot RCT is open, will be 32 per centre (64 in total from the two pilot centres). We propose to recruit to time, that is for a fixed period of four months rather than to a fixed sample size. We would want to see a minimum of 75% of the recruitment target to be confident of the trial viability i.e. at least 48 patients in total consented and randomized in four months' of recruitment from two centres.

8.1.3 Approach, non-participation and recruitment

Approach: Health professionals involved in approaching and screening PWCF and collecting data will be trained in the study protocol and procedures. Additionally those taking consent will have up-to-date training in Good Clinical Practice (GCP). All study personnel will be named on the study delegation log. Health professionals working with the CF team will identify a sample of PWCF registered at the centre via the CF registry database locally. All inclusion and exclusion criteria will be assessable via patient records and they will exclude any patients who do not fit the eligibility criteria.

A member of the participant's direct clinical team will send the potential participant a PIS and introductory letter by post or give the written information during a routine clinic visit. A sticker with a website address and Quick Response code will be placed in the envelope both of which will link to a video of the researcher explaining the study. If information is provided in a routine clinic visit, the clinical care team will seek permission for the ACtiF Interventionist to follow up with a phone call in order to answer any further questions and discuss involvement. Written informed consent may be conducted at this visit where the participant is happy to take part as this is a low risk trial.

Telephone call: Up to a week after posting out the information, the ACtiF Interventionist will telephone the PWCF to discuss the study over the phone and answer any questions. If the potential participant is happy to take part, the ACtiF Interventionist will arrange an appointment to gather written informed consent.

Non-participation: Spontaneously offered reasons for non-participation in the trial will be recorded.

For peer review only - http://bmjopen.bmj.com/site/about/guidelines.xhtml

8.2 Qualitative components

<u>*At each of the two pilot sites*</u> we will undertake:

- Audio-recordings of all 16 initial assessments for PWCF in the intervention arm and 10-12 consultations between the senior interventionist from the MDT (or other MDT member) and PWCF in the control arm. Numbers will depend on numbers of PWCF giving written consent for this.
- 10-12 semi-structured face-to-face (or telephone or skype) interviews with PWCF receiving the intervention and 10-12 semi-structured face-to-face interviews with PWCF in the control arm (total n~40-48 PWCF; n~40-48 interviews);
- two semi-structured face-to-face (or telephone or skype) interviews with each of the two interventionists in each centre (total n=4 interventionists; n=8 interviews); and,
- two semi-structured (face to face, telephone or skype) interviews with two members of the MDT (total n=4 staff; n=8 interviews).

Written informed consent will be obtained from both the interventionist and the PWCF participating in the audio recording when they consent to be in the study. Separate consent will be sought from PWCF and interventionists or members of the wider CF team for semi-structured interviews.

9. Assignment of interventions

9.1 Sequence generation

Participants will be allocated in equal proportions to one of the two groups using a computer generated pseudo-random list, stratified by centre and the number of days participants have been on IV antibiotics in the previous 12 month period as collected at consent visit, with random permuted blocks of varying sizes. The two categories for stratification within the number of IV days will be (i) less than or equal to 14 days and (ii) greater than 14 days.

9.2 Allocation concealment

The allocation sequence will be hosted by the Sheffield CTRU in accordance with their standard operating procedures and will be held on a secure server. Access to the allocation sequence will be restricted to those with authorisation. The sequence will be concealed until recruitment, data collection, and analyses are complete.

9.3 Implementation

The allocation sequence will be created by a Sheffield CTRU statistician who is not otherwise associated with the trial. At the consent visit, a health professional who is named on the delegation log, will go over the patient information sheet again with the study candidate and answer any questions. If the PWCF is still willing to enter the trial, they obtain full written consent and complete the eligibility form. If the participant is eligible, then baseline assessments will be taken. The recruiting health professional will log into the remote, secure Internet-based randomisation system and enter basic demographic information, after which the allocation will be revealed.

9.4 Blinding

After revelation of the allocation, only the statisticians will be blinded to allocation as per CTRU SOPs (ST001 and ST005)

For peer review only - http://bmjopen.bmj.com/site/about/guidelines.xhtml

10. Data collection, management and analysis

10.1 Quantitative data

10.1.1 Data collection methods

Data handling and record keeping. The Sheffield CTRU will oversee data collection, management and analysis and ensure the trial is undertaken according to Good Clinical Practice Guidelines and CTRU standard operating procedures. Data will be collected and retained in accordance with the Data Protection Act 1998. Patients will be reassured that all data which are collected during the course of the research will be kept strictly confidential.

The study team will train those collecting data in the study procedures before the trial begins. Data will either be collected directly from the participants, carers, interventionist, CFHealthHub or from source documents (e.g. patient notes) and input onto the CRF or Sheffield CTRU's electronic web-based data capture system (Prospect). The Data Monitoring and Management Plan for the study will provide further guidance on the types and levels of data and how these will be monitored and verified. Some essential documents may be posted to the central team to facilitate this e.g. participant consent forms in which case this will be detailed in the appropriate participant PIS and consent forms.

The CTRU will perform checks with the participant via monthly phone calls to ensure data is being captured and alert the local interventionist if there is an indication of a prescription change and a need to check pseudomonas (or other microorganism) status. This is required for the correct denominator to assess "normative adherence". Data will be extracted from the CF registry to understand exacerbations in the preceding 12 months since prior exacerbations can have a bearing on the optimum target regimen.

Plans to promote participant retention and complete follow-up.
Participant retention will be ensured by the following procedure:

1. At each point of contact, the interventionist will check with the participant that the Qualcomm hub or Smartphone hub is plugged in and turned on. A member of CTRU who is performing data and prescription checks may alert the interventionist. They will remind the participant of the proximity required for data transfer (10 metres)
2. In the event of no data being displayed in CFHealthHub for a period of at least a week (and the participant is not known to be on holiday) the interventionist will make contact with the participant (Email/Text/Telephone call) to check that the following
    - That the Qualcomm or Smartphone hub is plugged in
    - That the Qualcomm hub is working (showing solid green and yellow lights on the display)
    - That they have been within range of the Qualcomm hub sufficient to facilitate data transfer (10 metres)
    - That the Smartphone hub is switched on (showing the locked 'password' screen when any button is pressed)
    - That the Bi-neb and Smartphone hub have been kept in the same room, or at least have been in close proximity at some point during the day.

Any participants using the Bi-neb who are still experiencing issues after following the steps above, may receive a face to face or telephone support (at home or hospital) from the clinical trainer to resolve any outstanding issues.

Troubleshooting:

For peer review only - http://bmjopen.bmj.com/site/about/guidelines.xhtml

Data capture will be monitored both by interventionist at the site and centrally by the CTRU. In the event of data not being uploaded patients will be contacted to trouble shoot problems. Patients will be offered support to suit their circumstances including home visits (conducted by the members of the site research team) where necessary.

10.1.2 Data Management

Anonymised trial data will be entered onto a validated database system designed to an agreed specification between the Chief Investigator and Sheffield CTRU. The research staff at sites (mainly the ACtiF interventionist) will be responsible for data entry locally. The Sheffield CTRU Trial Manager, research assistant and the Data Management Team will work with sites to ensure the quality of data provided. The study manager, research assistant, data manager, PI's, any research nurses and site interventionist will have access to the anonymised data on the database through the use of usernames and encrypted passwords. The system has a full electronic audit trail and will be regularly backed up. The secure data management system will incorporate quality control procedures to validate the study data. Error reports will be generated where data clarification is needed. Output for analysis will be generated in a format and at intervals to be agreed between Sheffield CTRU and the Chief Investigator.

Trial documents will be retained in a secure location during and after the trial has finished. The study will use the CTRU's in-house data management system (Prospect) for the capture and storage of participant data. Prospect stores all data in a PostgreSQL database on virtual servers hosted by Corporate Information and Computing Services (CiCS) at the University of Sheffield. Prospect uses industry standard techniques to provide security, including password authentication and encryption using SSL/TLS. Access to Prospect is controlled by usernames and encrypted passwords, and a comprehensive privilege management feature can be used to ensure that users have access to only the minimum amount of data required to complete their tasks. This can be used to restrict access to personal identifiable data.

Participants who give consent to the qualitative part of this study will also give consent to their name and address to be given to the University of Sheffield qualitative research staff in order to be contactable.

10.1.3 Data quality assurance

Prospect provides a full electronic audit trail, as well as validation and verification features which will be used to monitor study data quality, in line with CTRU SOPs and the Data Management Plan (DMP). Error reports will be generated where data clarification is required. Rates of missing data and data points which are out of the expected or allowed range will be presented to the team at monthly management group meetings.

For peer review only - http://bmjopen.bmj.com/site/about/guidelines.xhtml

10.2 Qualitative data

10.2.1 Audio recordings of consultations

All initial assessments will be audio recorded with permission (n=16 in each site). Findings from early assessments will be fed back to the interventionist so that changes can be made to the intervention delivery before subsequent assessments. Consultations between the senior interventionist and PWCF in the control arm will be audio recorded with permission (n=10-12 in each site). Encrypted digital recorders will be used and recordings sent securely to the research team for analysis.

10.2.2 Semi-structured interviews: participants

In each site we will interview 3-4 PWCF receiving the intervention who are recruited at the beginning of the pilot. We will interview them around one month into the intervention to seek views of the most intensive part of the intervention. This will identify any problems early and be fed back to the intervention development team, staff delivering the intervention, and trial staff. We will interview 5-6 PWCF around four to six months into the intervention. These PWCF will have experienced more independent use of the CFHealthHub and we can explore how to keep PWCF engaged with the intervention in the longer term. We will interview 2-3 PWCF who drop out of the intervention to explore why this occurred. We will interview 10-12 PWCF in the control arm around four to six months into the trial to explore whether they have experienced aspects of patient activation and leakage of the intervention.

10.2.3 Semi-structured interviews: professionals

The first interviews with the interventionist and senior interventionist in each site will take place after they have undertaken assessments with the first few PWCF to identify teething problems with the intervention or the trial and the comprehensiveness of the training sessions they received. The findings will be fed back to the team to consider whether changes are needed to the intervention or trial protocol. The second interviews will take place when the first few PWCF have completed the intervention to allow the interventionist to reflect back over the whole process. The interventionists may have different lengths of experience of working with CF, nebulisers or behaviour change and we will consider the influence of differences in backgrounds on their ability to implement the intervention.

We will also undertake interviews with two members of the MDT at each centre when the first few PWCF have received 2-3 months of the intervention and then again towards the end of the feasibility study when all PWCF have been recruited and received around 3 months of the intervention.

10.2.4 Undertaking the interviews

For the interviews we have developed topic guides based on our research questions and these are attached to the application. Topic guides develop throughout any qualitative interview study and our topic guides may change as the study progresses. We will audio record all interviews after receiving written permission to do so. We will use an encrypted digital recorder. Reflexive notes will be made during and after the interviews. We expect

interviews to last around one hour. We do not expect data saturation in pilot studies; the aim is to identify any learning that can be addressed in preparation for the full trial.

11. Data analysis

11.1 Quantitative analysis

The analysis will be performed after data lock by a CTRU statistician under the supervision of the senior study statistician. As the trial is a pragmatic parallel group RCT data will be reported and presented according to the CONSORT 2010 statement [61] with reference to proposed extension for pilot / feasibility studies [37]. As a pilot/feasibility study the main analysis will be mainly descriptive and focus on confidence interval estimation and not formal hypothesis testing [58]. We will report rates of consent, recruitment and follow-up by centre and by randomized group.

Clinical outcome measures will be summarised overall and by randomized group. Baseline demographic (age, gender), physical measurements (e.g. weight, height, BMI), and patient reported outcome measures (EQ-5D, PAM-13, Assessment of Routine, MAD-3, SRBAI, CFQ-R, GAD-7, COM-BMQ, PHQ-8), and clinical measurements (e.g. FEV1, IV days in last registry year) will be described and summarised overall and for both treatment groups.

The primary outcome is the number of pulmonary exacerbations treated with IV antibiotics over the 6 month post-randomisation follow-up period. We will also include, as part of the feasibility analysis, estimation of the effect size for the 6-month pulmonary exacerbations outcome with 95% confidence interval estimates to check that the likely effect is within a clinically relevant range (as confirmation that it is worth progressing with the full trial). For this we will use a Poisson generalised linear model (GLM). Secondary continuous outcomes such as six-month post randomisation FEV1, BMI EQ-5D, PAM-13, Assessment of Routine, MAD-3, SRBAI, CFQ-R, GAD-7, COM-BMQ, PHQ-8) will be analysed with a multiple linear regression model with the baseline value of the outcome and randomised group as covariates. The treatment group coefficient and its associated 95% confidence interval will be reported from the various multiple linear regression models. The mean level of adherence (to prescribed medication) between the intervention and control groups over the 6 month post-randomisation follow-up period will also be reported and compared between the groups and a 95% confidence interval (CI) for the mean difference in this parameter between the randomised groups will also be calculated.

Further analyses with the objective nebuliser data will be performed to explore the process of habit formation with the delivery of the adherence intervention. The analyses will include:

(a) generating objective habit scores by taking into account time of nebuliser use
(b) using statistical process control to identify when periods of stability is achieved
(c) other time-series methods, including cross-correlation between habit scores and adherence.

Adverse events will be based on serious adverse events (SAE) case report forms. A serious adverse event is defined as any adverse event or adverse reaction that results in death, is life-threatening, requires hospitalisation or prolongation of existing hospitalisation, results in persistent or significant disability or incapacity, or is a congenital anomaly or birth defect.

For peer review only - http://bmjopen.bmj.com/site/about/guidelines.xhtml

The following summaries will be presented as overall rates and stratified by AE classification:

- the number and percentages of patients reported as having Serious Adverse Events (SAE) in each treatment arm; and,

- the number and percentages recorded as having all forms of Adverse Events (AE) in each arm.

This information along with the acceptability of the study design and protocol to patients/GPs; the safety of the intervention; patient recruitment and attrition/retention rates will enable us to determine whether or not the definitive RCT is feasible within a satisfactory timescale and cost envelope using UK centres alone.

11.2 Qualitative analysis

Transcripts will be coded using the latest version of NVivo (QSR International). The analysis will use the National Centre for Social Research 'Framework' approach [62]. AO'C and SD will undertake the following stages of the analysis of patient transcripts: familiarisation; identifying a thematic framework; indexing; charting; and, mapping and interpretation. The theoretical framework for understanding intervention adherence is the Necessities-Concerns framework [63] within the COM-B system [18]. This will be used within the thematic framework. We will use the process evaluation functions of context, mechanisms and implementation to frame the analysis [43]. Within mechanisms we will use the COM-B system as stated above and consider the use of the Theoretical Domains Framework [36]. We will compare and contrast findings from each site because the different backgrounds of the interventionists, and the different contexts in which care is provided in each CF unit, may affect implementation and acceptability of the intervention.

Figure 7. Assumptions of the MRC Guidance on Process Evaluation

[39, 64]

For peer review only - http://bmjopen.bmj.com/site/about/guidelines.xhtml

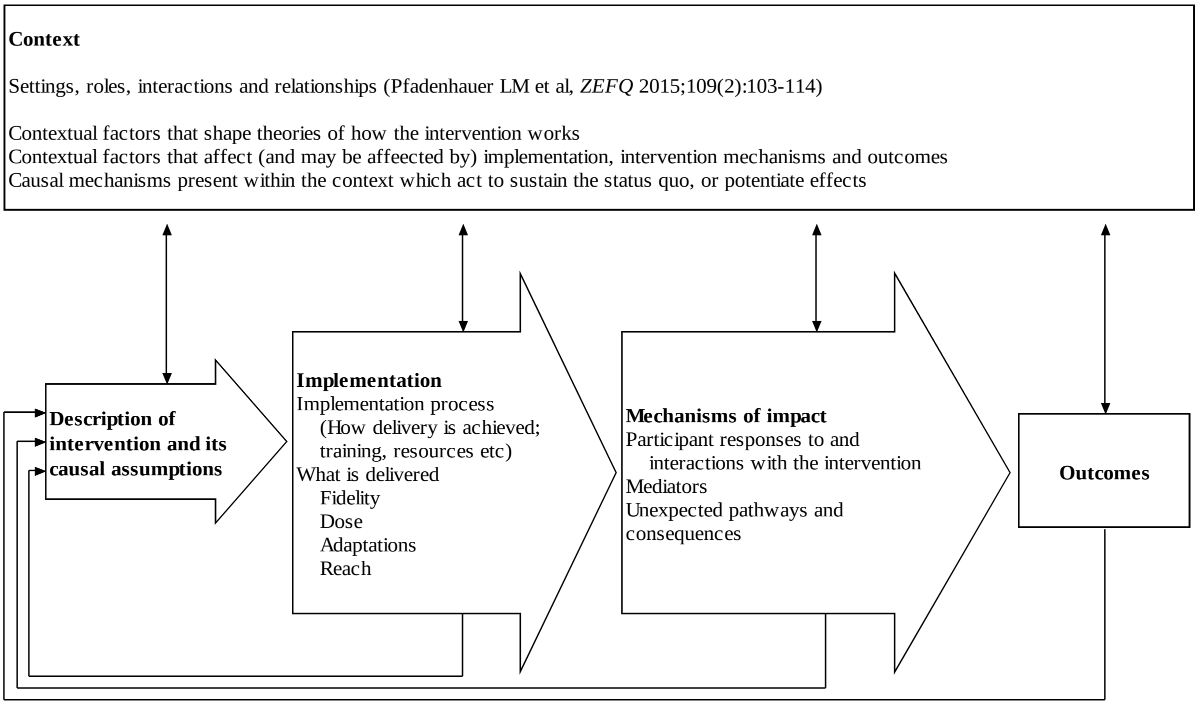

This qualitative research will:

- Inform the refinement of the intervention (e.g. CFHealthHub, training of interventionists, initial assessments, manualised instructions) and its implementation (e.g. introduction within a CF Unit) for use in the full trial.
- Inform refinement to trial procedures for the full trial.
- Inform the selection of the final secondary measures used in the full trial to ensure they address the perceived benefits of the intervention.
- Help to understand the extent of any leakage of the intervention to controls.

11.3 Combining data and findings from the different components

We will use Farmer's triangulation protocol to display the findings from each component of the study together and discuss as a team the extent to which findings converge, complement each other or contradict each other [65, 66]. For example, we will display all findings about recruitment together to consider the feasibility of recruitment for the full trial and the actions required to ensure feasibility. We will also display in a matrix the qualitative and quantitative data for individual PWCF who have received the intervention and been interviewed [66]. We will use this to consider the extent to which our secondary outcome measures identify issues raised by PWCF in the interviews.

12. Monitoring

12.1 Oversight

The CTRU SOP GOV003 Data Monitoring and Ethics Committee states "A DMEC does not need convening in studies that carry low risk to patients". This project involves

For peer review only - http://bmjopen.bmj.com/site/about/guidelines.xhtml

delivering a behaviour change intervention through the website CFHealthHub and would therefore be classified as low risk.

The overall responsibility for the study will be with Sheffield Teaching Hospitals NHS Trust who will act as sponsors for the study. The local Principal Investigator (PI) will be responsible for the study at each participating site and it will be registered and approved with each local R&D department. The study will be conducted in accordance with the protocol, GCP and Sheffield CTRU Standard Operating Procedures. The two committees which will govern the conduct of the study are:

1. Programme Steering Committee (PSC)
2. Project Management Group (PMG)

The PSC will be responsible for the overall conduct of the trial and consists of an independent chair and four other independent members including a statistician and PPI representative. The committee will meet every 6 months to monitor the study.

The PMG will comprise of the trial manager and the core research team . The PMG will meet on a monthly basis to monitor the day-to-day running of the trial. The Trial Manager will be jointly supervised by the CI and the Assistant Director of CTRU via the form of regular meetings (face to face and telephone calls). The Trial Manager will be responsible for liaising with the whole project team. Trial monitoring procedures will be assessed based on the level of risk of the study. The Site Monitoring Plan will outline the types and frequency of site monitoring activities for the study and this will be agreed with the Sponsor prior to the start of the study.

12.2 Description of any interim analyses and stopping guidelines

There are no planned interim analyses or stopping guidelines for this study.

12.3 Harms (safety assessments)

12.3.1 Serious Adverse Events

Trial sites are to report Serious Adverse Events (SAEs) in conjunction with the CTRU standard operating procedure PM004 (Adverse events and serious adverse events). The definition of an SAE is as follows:

- results in death;
- is life-threatening* (subject at immediate risk of death);
- requires in-patient hospitalisation or prolongation of existing hospitalisation;**
- results in persistent or significant disability or incapacity;
- consists of a congenital anomaly or birth defect; or,
- is another important medical event that may jeopardise the subject.***

* ‘life-threatening’ refers to an event in which the patient was at risk of death at the time of the event; it does not refer to an event which hypothetically might have caused death if it were more severe.
**Hospitalisation is defined as an inpatient admission, regardless of length of stay, even if the hospitalisation is a precautionary measure for continued observation. Hospitalisations

For peer review only - http://bmjopen.bmj.com/site/about/guidelines.xhtml

for a pre-existing condition, including elective procedures that have not worsened, do not constitute an SAE.
***Other important medical events that may not result in death, be life-threatening, or require hospitalisation may be considered a serious adverse event/experience when, based upon appropriate medical judgment, they may jeopardise the subject and may require medical or surgical intervention to prevent one of the outcomes listed in this definition.

It is not anticipated that there will be many SAEs related to the behaviour change intervention. We will report any SAEs which are deemed related to the trial intervention and unexpected to the Sponsor within the specified timeframes below (12.3.4).

12.3.2 Adverse events we require reporting:

We do require that sites report any new diagnosis of depression which requires treatment with medication or psychological therapy e.g. Cognitive Behavioural Therapy (CBT).

12.3.3 Expected SAEs and adverse events

Certain adverse events are common to CF and associated medications. Expected SAEs must be reported in the annual safety report. Hospitalisation as a result of an exacerbation will be recorded in the study database and not be reported as an SAE.

Expected AEs in relation to medications or common in patients with CF

1. Acute FEV1 drop >15% after 1st dose of medication
2. Increased productive cough
3. Nasal congestion or stuffy nose
4. Chest congestion
5. Wheezing
6. Chest pain or chest discomfort
7. Voice alteration/change
8. Dysponea (breathlessness)
9. Haemoptysis (coughing blood)
10. Rhinitis
11.
12. Headache
13. Crackles in lung
14. Throat irritation/ sore throat
15. URTI
16. Sinusitis
17. Deafness

18. Indigestion / reflux
19. Tonsillitis
20. Joint pain
21. Decreased appetite
22. Fatigue
23. Headache

For peer review only - http://bmjopen.bmj.com/site/about/guidelines.xhtml

24. Distal intestinal obstructive syndrome
25. Fever
26. Otitis media or ear infection
27. Conjunctivitis
28. Pneumothorax
29. Decreased exercise tolerance
30. Pyrexia
31. Abdominal pain
32. Influenza
33. Pseudomonas infection
34. Vomiting
35. Diabetes
36. Pneumonia

12.3.4 Reporting

Adverse events and SAEs can be reported for participants at any stage of their trial participation. A member of the site study team (interventionist, clinician or other) will enquire about any adverse events at routine clinic appointments. These will be record on the adverse event section of the paper CRF and database. The event will be assessed by the local Principal Investigator and the form will be kept in the site file. Serious adverse events will be reported in the periodic safety reports to the research ethics committee and Trial Steering committee.

All adverse events (serious or other based on the definitions above) will be recorded on the case report form and details will be **entered on the study database within 1 week of completing the paper form**. Any SAEs which are deemed related to the trial intervention, the site will complete the paper CRF and **fax details this form to the CTRU within 24 hours of becoming aware of the event** in order for the CTRU to report this event to the Sponsor and the main REC within the required timeframes (15 days).

In participants using the Bi-Neb, any Adverse or SAEs relating to the use of Promixin via this device will be reported to the Patient Support team (PSP) at Phillips as per their standard practice.

12.4 Auditing

The sponsor will permit monitoring and audits by the relevant authorities, including the Research Ethics Committee. The investigator will also allow monitoring and audits by these bodies and the sponsor, and they will provide direct access to source data and documents.

12.5 Finance and indemnity

The trial has been financed by the NIHR and details have been drawn up in a separate agreement. This is an NHS sponsored study. If there is negligent harm during the clinical

For peer review only - http://bmjopen.bmj.com/site/about/guidelines.xhtml

trial when the NHS body owes a duty of care to the person harmed, NHS Indemnity will cover NHS staff, medical academic staff with honorary contracts and those conducting the trial. NHS Indemnity does not offer no-fault compensation and is unable to agree in advance to pay compensation for non-negligent harm. Ex-gratia payments may be considered in the case of a claim.

13. Ethics and dissemination

13.1 Approvals

The trial will be conducted subject to Research Ethics Committee favourable opinion including any provisions for site specific assessment. The application will be submitted through the IRAS central allocation system. The approval letter from the ethics committee and copy of approved patient information leaflets, consent forms and any ethically approved questionnaires will be present in the site files before initiation of the study and patient recruitment. Local research governance approvals will be sought from all participating research sites. This clinical trial will be conducted in accordance with Good Clinical Practice Guidelines and CTRU standard operating procedures. MHRA approval is not required for this study.

13.2 Protocol amendments

The investigator will be updated following an amendment to the protocol or study documents. The new documents, REC approval, R&D approval, HRA assessment letter and any other appropriate documentation surrounding the amendment will be sent to the site via a “site file update”. The sites will receive the documents with a site file update sheet, detailing where to file the amended documents and which documents to supersede. If there are any significant changes to the study procedures or eligibility criteria sites will be notified by a combination of email, telephone, newsletters or additional project training when required.

In relation to informing REC, if any study documents require amending, the changes will be discussed with the sponsor and either a substantial (via IRAS and HRA) or minor amendment (notification via email) will be submitted to REC and HRA. Following REC acknowledgment and approval (when applicable) other appropriate approvals will be obtained i.e. HRA and R&D approval.

If a protocol amendment requires participants to be re-consented they will be informed of the amendment by an updated participant information sheet and will be asked to re-consent to the study. Trial registries, journals and regulators will be updated regarding protocol amendments when appropriate.

13.3 Consent

Consent for the main trial:
The ACtiF trial interventionist or local PI at the site will be responsible for taking informed consent from potentially eligible trial participants face to face at home or in clinic. Any researcher or clinical member of the team taking informed consent will be trained in study procedures and GCP. Participants will have the option to specify whether they are

For peer review only - http://bmjopen.bmj.com/site/about/guidelines.xhtml

interested in being approached for the qualitative interviews and audio recordings. However, they do not have to consent to these to be involved in the main study.

Consent for the interviews:
Consent for interviews (participant, interventionist or MDT member) will separately be taken by the qualitative researcher. Participants can participate in the main trial but choose to not take part in the qualitative research.

13.4 Confidentiality

Participant confidentiality will be respected at all times. Participant names and contact details will be collected and entered on the prospect database. Access to these personal details will be restricted to users with appropriate privileges only. All users who do not require access to identifiable data will only identify data by participant ID number, and no patient identifiable data will be transferred from the database to the statistician.

Trial documents (paper and electronic) will be retained in a secure location during and after the trial has finished. All source documents will be retained for a period of 5 years following the end of the trial. Where trial related information is documented in the medical records – those records will be retained for 5 years after the last patient last visit. Each site is responsible for ensuring records are archived and the information supplied to the Chief Investigator.

Any participant data held within CFHealthHub will be stored on a secure server at the University of Manchester. CFHealthHub complies with the Data Protection Act and follows best practice guidelines on security and information governance. Encrypted channels are used to transfer any data to and from the web and mobile application platforms. All user interaction with the CFHealthHub server and each action performed by a user will be logged. An audit log contains the username of the user performing the action, the date & time of the action, short description of the action performed. All users are authenticated via a secure password a with access to the system restricted on a role basis.

13.5 Declaration of Interests

Martin Wildman has received funding from Zambon who market the Ineb to carry out research to understand the performance of the Ineb and in the past we received funding from Zambon to carry out work to understand barriers to adherence.

13.6 Access to data

The central ACtiF study team alone will have access to the final dataset details of which will be outlined in the study DMP.

13.7 Ancilliary and post-trial care

Centres will be able to continue to use CFHealthHub if they wish to do so after the end of the pilot and feasibility study. If so, participants in the control arm will be able to cross over to use the intervention at this stage.

13.8 Dissemination policy

As this is a feasibility study its main interest will be to potential researchers and funding bodies. Data will be reported according to the revised CONSORT statement (Schultz,

For peer review only - http://bmjopen.bmj.com/site/about/guidelines.xhtml

2010). The findings of this research will be available to NIHR, patient groups and other interested bodies. It will also be offered for presentation at medical meetings and will be offered for publication in peer reviewed medical journals.

For peer review only - http://bmjopen.bmj.com/site/about/guidelines.xhtml

References

1. Alexander BM, Petren EK, Grimes M, Fink A, Myers V, Sewall A: **Mission of the cystic fibrosis foundation Annual Data Report 2013 Cystic Fibrosis Foundation Patient Registry**. 2014.

2. Southern KW, Barker PM, Solis-Moya A, Patel L: **Macrolide antibiotics for cystic fibrosis**. In *Cochrane Database of Systematic Reviews*. Edited by Southern KW. Chichester, UK: John Wiley & Sons, Ltd; 2011.

3. Ramsey BW, Davies J, McElvaney NG, Tullis E, Bell SC, Dřevínek P, Griese M, McKone EF, Wainwright CE, Konstan MW, Moss R, Ratjen F, Sermet-Gaudelus I, Rowe SM, Dong Q, Rodriguez S, Yen K, Ordoñez C, Elborn JS: **A CFTR Potentiator in Patients with Cystic Fibrosis and the *G551D* Mutation**. *N Engl J Med* 2011, **365**:1663–1672.

4. McCoy KS, Quittner AL, Oermann CM, Gibson RL, Retsch-Bogart GZ, Montgomery AB: **Inhaled aztreonam lysine for chronic airway Pseudomonas aeruginosa in cystic fibrosis.** *Am J Respir Crit Care Med* 2008, **178**:921–8.

5. Ryan G, Mukhopadhyay S, Singh M: **Nebulised anti-pseudomonal antibiotics for cystic fibrosis**. In *Cochrane Database of Systematic Reviews*. Edited by Ryan G. Chichester, UK: John Wiley & Sons, Ltd; 2003.

6. Jones AP, Wallis C: **Dornase alfa for cystic fibrosis**. In *Cochrane Database of Systematic Reviews*. Edited by Jones AP. Chichester, UK: John Wiley & Sons, Ltd; 2010.

7. Wark P, McDonald VM: **Nebulised hypertonic saline for cystic fibrosis**. In *Cochrane Database of Systematic Reviews*. Edited by Wark P. Chichester, UK: John Wiley & Sons, Ltd; 2009.

8. Ryan G, Singh M, Dwan K: **Inhaled antibiotics for long-term therapy in cystic fibrosis**. In *Cochrane Database of Systematic Reviews*. Edited by Ryan G. Chichester, UK: John Wiley & Sons, Ltd; 2011.

9. Eakin MN, Bilderback A, Boyle MP, Mogayzel PJ, Riekert KA: **Longitudinal association between medication adherence and lung health in people with cystic fibrosis**. *J Cyst Fibros* 2011, **10**:258.

10. Briesacher BA, Quittner AL, Saiman L, Sacco P, Fouayzi H, Quittell LM, Accurso F, Modi A, Lim C, Yu N, Geller D, Wagner M, Quittner A, DiMatteo M, Quittner A, Barker D, Marciel K, Grimley M, Emerson J, Rosenfeld M, McNamara S, Ramsey B, Gibson R, Nixon G, Armstrong D, Carzino R, Carlin J, Olinsky A, Robertson C, Grimwood K, et al.: **Adherence with tobramycin inhaled solution and health care utilization**. *BMC Pulm Med* 2011, **11**:5.

11. AL Q, KA R, Zhang J: **Relationship between adherence to pulmonary medications and health care costs: longitudinal analyses from 2005-2011.** *Pediatr Pulmonol* 2012, **47**:198.

12. Smyth A, Lewis S, Bertenshaw C, Choonara I, McGaw J, Watson A: **Case-control study of acute renal failure in patients with cystic fibrosis in the UK.** *Thorax* 2008, **63**:532–5.

For peer review only - http://bmjopen.bmj.com/site/about/guidelines.xhtml

13. McManus P: *Lead Pharmacist - Specialised Services (South Yorkshire and Bassetlaw) NHS England*. .

14. Daniels T, Goodacre L, Sutton C, Pollard K, Conway S, Peckham D: **Accurate assessment of adherence: self-report and clinician report vs electronic monitoring of nebulizers.** *Chest* 2011, **140**:425–32.

15. McNeil S: *Personal Communication from Stephanie McNeil, Chief Statistician of UK CF Registry*. .

16. Horne RW, Barber N, Elliot R, Morgan M: *Concordance, Adherance and Compliance in Medicine Taking: A Conceptual Map and Research Priorities. National Co-Ordinating Centre for NHS Service Delivery and Organisational R&D (NCCSDO)*. 2005.

17. Craig P, Dieppe P, Macintyre S, Michie S, Nazareth I, Petticrew M: **Developing and evaluating complex interventions: the new Medical Research Council guidance**. *BMJ* 2008:a1655–a1655.

18. Michie S, van Stralen MM, West R: **The behaviour change wheel: a new method for characterising and designing behaviour change interventions.** *Implement Sci* 2011, **6**:42.

19. Parham R, Thomas S, Mills R, Al. E: **CF patient's beliefs about nebuliser treatment: implications for adherence to treatment**. *J Cyst Fibros* 2012, **11**(Suppl 1):S9.

20. Bucks RS, Hawkins K, Skinner TC, Horn S, Seddon P, Horne R: **Adherence to treatment in adolescents with cystic fibrosis: the role of illness perceptions and treatment beliefs.** *J Pediatr Psychol* 2009, **34**:893–902.

21. George M, Rand-Giovannetti D, Eakin MN, Borrelli B, Zettler M, Riekert KA: **Perceptions of barriers and facilitators: self-management decisions by older adolescents and adults with CF.** *J Cyst Fibros* 2010, **9**:425–32.

22. Dziuban EJ, Saab-Abazeed L, Chaudhry SR, Streetman DS, Nasr SZ: **Identifying barriers to treatment adherence and related attitudinal patterns in adolescents with cystic fibrosis.** *Pediatr Pulmonol* 2010, **45**:450–8.

23. Bregnballe V, Schiøtz PO, Boisen KA, Pressler T, Thastum M: **Barriers to adherence in adolescents and young adults with cystic fibrosis: a questionnaire study in young patients and their parents.** *Patient Prefer Adherence* 2011, **5**:507–15.

24. Modi AC, Quittner AL: **Barriers to treatment adherence for children with cystic fibrosis and asthma: What gets in the way?** *J Pediatr Psychol* 2006, **31**:846–858.

25. Abbott J, Dodd M, Bilton D, Webb AK: **Treatment compliance in adults with cystic fibrosis.** *Thorax* 1994, **49**:115–20.

26. Abbott J, Dodd M, Webb AK: **Health perceptions and treatment adherence in adults with cystic fibrosis.** *Thorax* 1996, **51**:1233–8.

27. Abbott J, Dodd M, Gee L, Webb K: **Ways of coping with cystic fibrosis: implications for treatment adherence.** *Disabil Rehabil* 2001, **23**:315–24.

28. Conway SP, Pond MN, Hamnett T, Watson A: **Compliance with treatment in adult patients with cystic fibrosis.** *Thorax* 1996, **51**:29–33.

For peer review only - http://bmjopen.bmj.com/site/about/guidelines.xhtml

29. Jones S, Curley R, Wildman M: **345 Systematic review of qualitative studies investigating barriers to adherence in patients with cystic fibrosis using framework analysis structured by a conceptual framework of behaviour change**. *J Cyst Fibros* 2013, **12**(Suppl 1):S136.

30. Kettler LJ, Sawyer SM, Winefield HR, Greville HW: **Determinants of adherence in adults with cystic fibrosis.** *Thorax* 2002, **57**:459–64.

31. Lask B: **Non-Adherence to Treatment in Cystic Fibrosis**. *J R Soc Med* 1994, **87**(21 Suppl):25–27.

32. Latchford G, Duff A, Quinn J, Conway S, Conner M: **Adherence to nebulised antibiotics in cystic fibrosis.** *Patient Educ Couns* 2009, **75**:141–4.

33. Horne R, Weinman J, Barber N, Elliott R: *Concordance , Adherence and Compliance in Medicine Taking: Report for the National Co-Ordinating Centre for NHS Service Delivery and Organisation R & D (NCCSDO)*. London: NCCSDO; 2005.

34. Ivers N, Jamtvedt G, Flottorp S, Young JM, Odgaard-Jensen J, French SD, O'Brien MA, Johansen M, Grimshaw J, Oxman AD: **Audit and feedback: effects on professional practice and healthcare outcomes.** *Cochrane database Syst Rev* 2012, **6**:CD000259.

35. Geraedts H, Zijlstra A, Bulstra SK, Stevens M, Zijlstra W: **Effects of remote feedback in home-based physical activity interventions for older adults: a systematic review.** *Patient Educ Couns* 2013, **91**:14–24.

36. Cane J, O'Connor D, Michie S: **Validation of the theoretical domains framework for use in behaviour change and implementation research.** *Implement Sci* 2012, **7**:37.

37. Thabane L, Ma J, Chu R, Cheng J, Ismaila A, Rios LP, Robson R, Thabane M, Giangregorio L, Goldsmith CH: **A tutorial on pilot studies: the what, why and how.** *BMC Med Res Methodol* 2010, **10**:1.

38. Baranowski T, Stables G: **Process Evaluations of the 5-a-Day Projects**. *Heal Educ Behav* 2000, **27**:157–166.

39. Pfadenhauer LM, Mozygemba K, Gerhardus A, Hofmann B, Booth A, Lysdahl KB, Tummers M, Burns J, Rehfuess EA: **Context and implementation: A concept analysis towards conceptual maturity**. *Z Evid Fortbild Qual Gesundhwes* 2015, **109**:103–114.

40. Weber K, Glynn M a.: **Making Sense with Institutions: Context, Thought and Action in Karl Weick's Theory**. *Organ Stud* 2006, **27**:1639–1660.

41. Hyatt D: **Time for a change: a critical discoursal analysis of synchronic context with diachronic relevance**. *Discourse Soc* 2005, **16**:515–534.

42. Linnan L, Steckler A: **Process evaluation for public health interventions and research: an overview**. In *Process evaluation for public health interventions and research*. 1st edition. Edited by Linnan L, Steckler A. San Francisco: Jossey-Bass; 2002:1–23.

43. Moore GF, Audrey S, Barker M, Bond L, Bonell C, Hardeman W, Moore L, O'Cathain A, Tinati T, Wight D, Baird J: **Process evaluation of complex interventions: Medical Research Council guidance.** *BMJ* 2015, **350**:h1258.

For peer review only - http://bmjopen.bmj.com/site/about/guidelines.xhtml

44. Nilsen P: **Making sense of implementation theories, models and frameworks**. *Implement Sci* 2015, **10**:1–13.

45. Meyers DC, Durlak J a., Wandersman A: **The Quality Implementation Framework: A Synthesis of Critical Steps in the Implementation Process**. *Am J Community Psychol* 2012, **50**:462–480.

46. Borrelli B: **The Assessment, Monitoring, and Enhancement of Treatment Fidelity In Public Health Clinical Trials.** *J Public Health Dent* 2011, **71**:S52–S63.

47. Fuchs HJ, Borowitz DS, Christiansen DH, Morris EM, Nash ML, Ramsey BW, Rosenstein BJ, Smith AL, Wohl ME: **Effect of aerosolized recombinant human DNase on exacerbations of respiratory symptoms and on pulmonary function in patients with cystic fibrosis. The Pulmozyme Study Group.** *N Engl J Med* 1994, **331**:637–42.

48. Ratjen F, Durham T, Navratil T, Schaberg A, Accurso FJ, Wainwright C, Barnes M, Moss RB: **Long term effects of denufosol tetrasodium in patients with cystic fibrosis.** *J Cyst Fibros* 2012, **11**:539–49.

49. Miller MR, Hankinson J, Brusasco V, Burgos F, Casaburi R, Coates A, Crapo R, Enright P, van der Grinten CPM, Gustafsson P, Jensen R, Johnson DC, MacIntyre N, McKay R, Navajas D, Pedersen OF, Pellegrino R, Viegi G, Wanger J: **Standardisation of spirometry.** *Eur Respir J* 2005, **26**:319–38.

50. Herdman M, Gudex C, Lloyd A, Janssen M, Kind P, Parkin D, Bonsel G, Badia X: **Development and preliminary testing of the new five-level version of EQ-5D (EQ-5D-5L).** *Qual Life Res* 2011, **20**:1727–36.

51. Hibbard JH, Mahoney ER, Stockard J, Tusler M: **Development and testing of a short form of the patient activation measure.** *Health Serv Res* 2005, **40**(6 Pt 1):1918–30.

52. Wong MD, Sarkisian C a., Davis C, Kinsler J, Cunningham WE: **The Association Between Life Chaos, Health Care Use, and Health Status Among HIV-Infected Persons**. *J Gen Intern Med* 2007, **22**:1286–1291.

53. Verplanken B, Orbell S: **Reflections on Past Behavior: A Self-Report Index of Habit Strength1**. *J Appl Soc Psychol* 2003, **33**:1313–1330.

54. Quittner a L, Sweeny S, Watrous M, Munzenberger P, Bearss K, Gibson Nitza a, Fisher L a, Henry B: **Translation and linguistic validation of a disease-specific quality of life measure for cystic fibrosis.** *J Pediatr Psychol* 2000, **25**:403–14.

55. Kroenke K, Strine TW, Spitzer RL, Williams JBW, Berry JT, Mokdad AH: **The PHQ-8 as a measure of current depression in the general population**. *J Affect Disord* 2009, **114**:163–173.

56. Spitzer RL, Kroenke K, Williams JBW, Löwe B: **A brief measure for assessing generalized anxiety disorder: the GAD-7.** *Arch Intern Med* 2006, **166**:1092–7.

57. Horne R, Weinman J, Hankins M: **The beliefs about medicines questionnaire: The development and evaluation of a new method for assessing the cognitive representation of medication**. *Psychol Health* 1999, **14**:1–24.

58. Lancaster GA, Dodd S, Williamson PR: **Design and analysis of pilot studies:**

For peer review only - http://bmjopen.bmj.com/site/about/guidelines.xhtml

recommendations for good practice. *J Eval Clin Pract* 2004, **10**:307–12.

59. Arain M, Campbell MJ, Cooper CL, Lancaster GA: **What is a pilot or feasibility study? A review of current practice and editorial policy.** *BMC Med Res Methodol* 2010, **10**:67.

60. Eldridge S, Bond C, Campbell M, Lancaster G, Thabane L, Hopwell S: **Definition and reporting of pilot and feasibility studies**. *Trials* 2013, **14**(Suppl 1):O18.

61. Schulz KF, Altman DG, Moher D: **CONSORT 2010 statement: Updated guidelines for reporting parallel group randomized trials**. *Annu Intern Med* 2010, **152**:726–732.

62. Ritchie J, Spencer L: **Qualitative data analysis for applied policy research**. In *Analysing qualitative data*. Edited by Bryman A, Burgess RG. Routledge; 1994:173–194.

63. Horne R: **Treatment perceptions and self-regulation**. In *The Self-regulation of Health and Illness Behaviour*. Edited by Cameron L, Leventhal H. London: Routledge; 2003:138–53.

64. Moore GF, Audrey S, Barker M, Bond L, Bonell C, Hardeman W, Moore L, O'Cathain A, Tinati T, Wight D, Baird J: **Process evaluation of complex interventions: Medical Research Council guidance**. *BMJ* 2015, **350**(mar19 6):h1258–h1258.

65. Farmer T, Robinson K, Elliott SJ, Eyles J: **Developing and implementing a triangulation protocol for qualitative health research.** *Qual Health Res* 2006, **16**:377–94.

66. O'Cathain A, Murphy E, Nicholl J, Cathain AO: **Three techniques for integrating data in mixed methods studies**. *BMJ* 2010, **341**(sep17 1):c4587–c4587.

For peer review only - http://bmjopen.bmj.com/site/about/guidelines.xhtml

Appendix 1. W.H.O. Trial Registration Data Set

DATA CATEGORY	INFORMATION
Primary registry and trial identifying number	To be added
Date of registration in primary registry	To be added
Secondary identifying numbers	NIHR: RP-PG-1212-20015<br>Sponsor (STH): STH19213
Source(s) of monetary or material support	National Institute for Health Research (NIHR) Programme Grants for Applied Research programme.
Primary sponsor	Sheffield Teaching Hospitals NHS Foundation Trust.
Secondary sponsor(s)	none
Contact for public queries	Chin Maguire<br>Trial Manager<br>Clinical Trials Research Unit<br>University of Sheffield<br>Regent Court<br>30 Regent Street<br>Sheffield<br>S1 4DA<br>Tel: (+44) (0)114 222 0717<br>Fax: (+44) (0)114 222 0870<br>email : c.maguire@sheffield.ac.uk
Contact for scientific queries	Dr Martin Wildman<br>Adult CF Centre<br>Northern General Hospital<br>Herries Road<br>Sheffield<br>S5 7AU<br>Tel: (0114) 2715212<br>Fax: (0114) 222 0870<br>email : Martin.Wildman@sth.nhs.uk
Public title	Adherence to treatment in adults with Cystic Fibrosis (ACtiF)
Scientific title	Development and evaluation of an intervention to support Adherence to treatment in adults with Cystic Fibrosis : a feasibility study comprised of an external pilot randomised controlled trial and process evaluation
Countries of recruitment	United Kingdom
Health condition(s) or problem(s) studied	Cystic Fibrosis
Intervention(s)	Usual care plus a microchipped nebuliser with or without a complex intervention. The complex intervention consists of:

	- A software platform, CFHealthHub mobile apps and website, which allows access to medication adherence data and education modules intended to remove barriers to adherence<br>- A manual containing a 'behaviour change toolkit' to guide interactions between health
Key inclusion and exclusion criteria	Inclusion criteria for participants<br>1.Diagnosed with CF and with data within the CF registry<br>2.Aged 16 years and above<br>3.Taking inhaled mucolytics or antibiotics via a chipped nebuliser (e.g. eTrack or Bi-Neb) or able and willing to take via eTrack or Bi-Neb.<br><br>Exclusion criteria for participants<br>1.Post-lung transplant<br>2.People on the active lung transplant list<br>3.Patients receiving palliative care,<br>4.Lacking in capacity to give informed consent<br>5.Using dry powder devices to take antibiotics or mucolytics
Study type	Feasibility study comprised of an external pilot randomised controlled trial and process evaluation
Date of first enrolment	Anticipated: 02/05/2016
Target sample size	We propose to recruit to time, that is for a fixed period of four months rather than to a fixed sample size. To match the proposed recruitment rate of the main RCT, the target sample over the four months for which the pilot RCT is open, will be n=64.
Recruitment status	Not yet open.
Primary outcome(s)	Exacerbations of cystic fibrosis as defined by the Fuchs criteria (*N Engl J Med* 1994, 331:637–42.)
Key secondary outcomes	None.

For peer review only - http://bmjopen.bmj.com/site/about/guidelines.xhtml

ACtiF Pilot Study

Control Patient Topic guide

Thank you for agreeing to take part in the interview today

Check timing ok

Check consent form filled in / received

Are you happy for the interview to be recorded?

We're interested in your experiences of the service that you receive for helping you to use your nebuliser.

1. Why did you decide to take part in the research?

2. How did you find being asked to take part in the trial? [Prompts: paperwork volume, information provided, questionnaires]

Now I'd like to ask you about the care you received before the trial started to help you use your nebuliser.

3. What types of things did the unit/hospital recommend that you do to help you use your nebuliser? [Prompts: appointments / what do you talk about? / nebulisers / skills to use your nebuliser properly / knowledge and beliefs?]

4. What types of things did the unit/hospital recommend that you do to help you use your nebuliser as much as possible? [Prompts: setting goals, solving problems, making plans, giving you information, building skills, beliefs about nebuliser medication, giving you confidence]

5. How did the care you received to help you use your nebuliser fit with any other care you received for CF more generally?

For peer review only - http://bmjopen.bmj.com/site/about/guidelines.xhtml

6. How could the care you received for helping you to use your nebuliser as prescribed be improved?

7. Overall how happy are you with the care you received for your nebuliser? [Prompts: what could be done better?]

Now I want to ask you about specific kinds of things that might have changed since the trial started:

8. Since you joined the trial has the care that you receive in the unit / hospital changed at all? [Prompts: Has anybody done anything different? What have they done?]

9. Since you joined the trial has anyone asked you to change how you use your nebuliser? If so, what have they suggested you do? [Prompt: capability skills / knowledge including beliefs / where has the change come from?]

10. Since you joined the trial has anyone suggested ways to help you use your nebuliser as much as possible? If so what? [Prompt: opportunity finding time to use nebuliser / making plans / setting goals / where has the change come from?]

11. Since you joined the trial has anyone helped you have more confidence to use your nebuliser as prescribed? [Prompt: where has the change come from? what have they done?]

Is there anything else that we haven't talked about that you'd like to comment on?

THANK YOU

The ACtiF Project is funded by the National Institute for Health Research's Programme Grants for Applied Research.

For peer review only - http://bmjopen.bmj.com/site/about/guidelines.xhtml

Sheffield Hallam University
The University Of Sheffield.
Sheffield Teaching Hospitals NHS
NHS Foundation Trust

ACtiF Pilot Study

Intervention Patient Topic guide

Thank you for agreeing to take part in the interview today

Check timing ok

Check consent form filled in / received

Are you happy for the interview to be recorded?

We're interested in your experiences of the service that you have received from CFHealthHub including both the meetings to discuss your nebuliser medication and the website/app you have used.

1. Why did you decide to take part in the research?

2. How did you find being asked to take part in the trial? [Prompts: recruitment, paperwork volume, information provided, questionnaires]

Now I'd like to ask you about your meetings with the person who has been working with you on CFHealthHub.

3. What types of things did they recommend that you do? [prompts: setting goals, solving problems, making plans (myplan), giving you information]

4. Do you think you have had any benefit from these meetings?

If yes, what benefit and what about the service helped you to get this?

If no, what has stopped you gaining benefit?

5. What was good about how the meetings were delivered? [Prompt: what needs to be improved?]

For peer review only - http://bmjopen.bmj.com/site/about/guidelines.xhtml

Now I'd like to talk to you about the CFHealthHub website / app.

6. What was good about the website? [Prompts: my plan, how am I doing, tool kit, graphs, my treatment]

7. What needs to be improved? [Prompts: my plan, how am I doing, tool kit, graphs, my treatment]

8. Do you think you've had any benefit from using the website?

   If yes, what benefit and what about the website helped you to get this?

   If no, what has stopped you gaining benefit?

9. Have the website and/or meetings helped you to improve how often you use your nebuliser?

   If yes, how has it helped you to do this?

   If not, why not?

10. How do the CFHealthHub website and the meetings work together?

11. Has using CFHealthHub helped you to be able to use your nebuliser any better? Why / Why not? [Prompt: capability skills / knowledge including beliefs]

12. Has using CFHealthHub helped you to find the time to use your nebuliser more? Why / why not? [Prompt: opportunity / making plans]

13. Has using CFHealthHub made you want to use your nebuliser more? Why / why not? [Prompt: motivation and confidence]

14. How does the CFHealthHub service (website and meetings) fit with the care you were already receiving at the unit/hospital?

For peer review only - http://bmjopen.bmj.com/site/about/guidelines.xhtml

15. Do you think you would continue using CFHealthHub? [Prompt: during the study / after the study]

16. Is CFHealthHub a good thing to use in general for people with CF? Why? / Why not?

17. How have you found being part of the study?

Is there anything else that we haven't talked about that you'd like to comment on?

THANK YOU

The ACtiF Project is funded by the National Institute for Health Research's Programme Grants for Applied Research.

For peer review only - http://bmjopen.bmj.com/site/about/guidelines.xhtml

ACTIF Pilot Study

Interventionist Topic guide

Thank you for agreeing to take part in the interview today.

Check timing ok

Check consent form filled in / received

Are you happy for the interview to be recorded?

Introduction to the interview: Interested in how you've found using CFHealthhub (CFHH) with your participants and any learning from it

The trial

1. What works or could be improved about:

   a) recruiting patients to the trial?

   b) collecting data?

   c) any other aspect?

The intervention:

Now I'd like to go through each of the steps for providing the intervention to get your views on each of these

2. What works or could be improved about:

   a) how you have assessed participants' adherence levels prior to using CFHH?

   b) how you set up appointments with your participants?

   c) session 1? [Prompts: gathering data, introducing the nebuliser, entering prescription data into CFHH, completion of screening tools, patient feedback, anything else]

For peer review only - http://bmjopen.bmj.com/site/about/guidelines.xhtml

d) session 2? [Prompts: reviewing adherence data, introducing CFHH, explaining modules, setting goals, action planning, identifying suitable tailored content, technical issues, anything else]

e) session 3? [Prompts: reviewing goals, reviewing adherence plans, motivation, problem solving, anything else]

3. What works or could be improved about the training manuals and training sessions?

4. What works or could be improved about the support available from the research team? [Prompts: timing, availability, problem solving].
[**Specific prompt for MDT senior interventionist**: do you think the training has equipped you to deliver this intervention in your centre yourself after the trial ends? If no, what further training would be needed?]

5. How has the CFHH intervention been received by the rest of the team? [Prompt: how has your communication been with the rest of the team about CFHH?]

6. What sort of follow-up did participants request? How will you handle this?

7. How has the CFHH intervention helped your participants to know how to use their nebuliser? [Prompt: capability / skills, knowledge and beliefs]

8. How has the CFHH intervention helped your participants find ways to use their nebuliser more? [Prompt: opportunity]

9. How has the CFHH intervention helped to motivate your participants to use their nebuliser? [Prompt: motivation / confidence]

General questions:

10. How engaged did participants seem with CFHH? [Prompt: What feedback if any have you had from participants about CFHH?]

11. How useful do you think CFHH is for your participants?

For peer review only - http://bmjopen.bmj.com/site/about/guidelines.xhtml

12. How easy / difficult has it been to get your participants to use CFHH?

13. Have you seen any changes to the ways in which your participants use their nebulisers since starting CFHH?

14. What have you learnt from using CFHH with your participants?

15. What if any are the benefits to you and / or to your participants of using CFHH?

16. How do you think CFHH fits with the other care offered by the centre?

17. How have you found being part of the trial?

Is there anything else you'd like to say about CFHH?

THANK YOU

For peer review only

The ACtiF Project is funded by the National Institute for Health Research's Programme Grants for Applied Research.

For peer review only - http://bmjopen.bmj.com/site/about/guidelines.xhtml

Sheffield Hallam University
The University Of Sheffield.
Sheffield Teaching Hospitals NHS
NHS Foundation Trust

ACTIF Pilot Study

MDT Topic guide

Thank you for agreeing to take part in the interview today.

Check timing ok

Check consent form filled in / received

Are you happy for the interview to be recorded?

We're interested in your views of the CFHealthHub service and how it fits into the care provided in your centre.

1. Can you describe the key things you did in your centre to help patients adhere to their nebulisers prior to the ACtiF study?

2. How does nebuliser adherence fit with the other things you do for CF patients?

3. What involvement have you had in the CFHealthHub intervention? [Prompts: website, interventionist, training of staff]

4. You had training to help you be more aware of patient activation. What did you think of the training? [Prompts: Do you think it has changed your practice in any way? If yes what changes, if no why not? Key aspects – patient knowledge including beliefs / skills / confidence]

5. Do you think CFHealthHub is a useful intervention? Why? / Why not? [Prompts: what do you think about the: website, feedback about adherence data, interventionist, training?]

6. How do you think the CFHealthHub intervention is operating in practice? [Prompt: what are the strengths / improvements needed?]

7. How does the CFHealthHub intervention fit with the care offered by your centre?

8. How does the CFHealthHub intervention help your patients to know how to use their nebuliser? [Prompts: Skills / knowledge / beliefs. How / Why doesn't it help?]

9. How does the CFHealthHub intervention help your patients to find ways to use their nebuliser more? [Prompts: How / Why doesn't it help?]

For peer review only - http://bmjopen.bmj.com/site/about/guidelines.xhtml

10. How does the CFHealthHub intervention help to motivate your patients to use their nebuliser? [Prompts: How / Why doesn't it help?]

11. Do you think CFHealthHub is helping your intervention patients to improve their adherence? If yes, what key things have helped this? If no, what if anything could be done to help this?

12. Has the CFHealthHub intervention changed anything about the way in which you and/or your team approach adherence in your centre?

i) for patients receiving the intervention?

ii) for patients not receiving the intervention?

[Prompts: MDT discussions / differences between control and intervention patients]

13. Which patient groups are most likely to benefit from CFHealthHub? Why?

14. Which patient groups are least likely to benefit from CFHealthHub? Why?

15. Would you consider continuing to use CFHealthHub in the future? Why? Why not?

16. How has it been for you / your centre taking part in the trial? [Prompt: recruitment to the study]

17. How able do you feel to go on delivering care related to improving adherence after the study ends? [Prompt: has the study changed the way you will go about this?]

18. Are there any aspects of the research that we haven't talked about that you'd like to comment on?

Is there anything else you'd like to say?

THANK YOU

The ACtiF Project is funded by the National Institute for Health Research's Programme Grants for Applied Research.

For peer review only - http://bmjopen.bmj.com/site/about/guidelines.xhtml

Additional File 03 - Quantitative results from process evaluation

Table a. Key dates in process evaluation by participant

Study ID	Interview Date	Baseline date	5 month follow up date	Date of first intervention meeting	Time in the trial at interview (days)	Time since first intervention session at interview (days)	Time in trial at follow up (days)
R02/02	13/09/2016	06/07/2016	10/11/2016	05/08/2016	69	39	127
R02/03	09/09/2016	08/07/2016	NA	05/08/2016	63	35	NA
R02/42	12/10/2016	15/07/2016	21/12/2016	NA	89	NA	159
R02/07	15/11/2016	12/07/2016	12/12/2016	09/09/2016	126	67	153
R02/12	02/11/2016	14/07/2016	03/01/2017	05/10/2016	111	28	173
R02/52	03/02/2017	04/07/2016	22/11/2016	05/10/2016	214	121	141
R01/44	01/12/2016	07/07/2016	16/11/2016	08/11/2016	147	23	132

For peer review only

R01/48	17/01/2017	04/07/2016	15/11/2016	13/10/2016	197	96	134
R01/49	30/01/2017	22/07/2016	07/12/2016	10/10/2016	192	112	138
R01/54	21/03/2017	25/07/2016	13/12/2016	02/11/2016	239	139	141
R01/39	27/02/2017	02/08/2016	21/12/2016	03/11/2016	209	116	141
R01/02	06/12/2016	31/08/2016	25/01/2017	15/08/2016	97	113	147
R01/40	05/12/2016	05/09/2016	17/02/2017	05/10/2016	91	61	165
R01/42	03/10/2016	12/09/2016	15/02/2017	15/08/2016	21	49	156

For peer review only

Table b. Interventionist-generated motivation data (intervention arm) R02/42, R02/49, R02/15 and R01/48 were all missing

Participant ID	Date	Consent Visit Motivation Rating	Was Participant motivation too low Answer Yes/No
R02/39	05.08.16	7	No
R02/40	23.08.16	4	No
R02/02	05.08.16	7	No
R02/03	03.08.16	1	No
R02/43	12.08.16	7	No
R02/05	22.08.16	5	No
R02/45	18.08.16	7	No
R02/07	09.08.16	7	No
R02/48	05.10.16	7	No
R02/10	14.09.16	7	No
R02/11	28.09.16	7	No
R02/50	26.09.16	7	No
R02/12	05.10.16	7	No
R02/52	03.10.16	7	No
R01/39	03.11.16	7	Page missing from report
R01/02	16.09.16	7	No
R01/03	03.10.16	5	No
R01/40	05.10.16	7	Page missing from report
R01/42	15.08.16	5	Page missing from report
R01/44	08.11.16	7	Page missing from report
R01/47	10.10.16	5	Yes
R01/06	10.10.16	7	Page missing from report
R01/49	17.10.16	7	No
R01/08	01.11.16	7	Page missing from report
R01/50	Missing report		
R01/53	29.11.16	7	Not ticked
R01/54	Missing report		
R01/10	10.11.16	2	Not ticked
R01/57	31.10.16	0	Yes

For peer review only

Table c. Engagement

	Adherence data collected (did not withdraw from data collection before 6m) n(%)	Total CFHH sessions Median (IQR)	Baseline adherence Median (IQR)
**Overall (n=33)**	29(88%)	3(1,8)	20(2.1,47.8)
**Qualitative case studies**			
**High adherence at end**			
R01/39	Yes	1	0
R02/07	Yes	2	96.7
R01/40	Yes	9	43.1
R02/52	Yes	13	96.6
**Moderate adherence at end**			
R01/49	Yes	4	13.2
**Low adherence at end**			
R01/54	Yes	11	44.8
R01/02	Yes	1	30.2
R01/48	Yes	3	1.8
R02/12	Yes	44	10.2
R02/03	No	3	5.4
R01/44	Yes	1	19.5
**Withdrawn**			
R01/42	Yes	41	21.1
R02/02	No	3	92.5
R02/42	No	0	4.2

Note: R02/42, R02/02 withdrew from adherence data collection and from the intervention and R02/03 was lost to follow-up. R01/42 did not withdraw from data collection until the end of the study; they did not contribute sufficient data for the 150-180 day period.

For peer review only

Table d. Activities: all participants

	Self-monitoring adherence	Tailored education about treatment	Tailored patient stories (videos)	Personalised action plan/Personalised goal-setting	Tailored problem-solving		Goal review; Rewards
	Clicks How am I doing?	Clicks Toolkit	Clicks Videos	Clicks Action Plan	Clicks Problem Solving	Clicks Coping Plan	Review sessions with Interventionist
**Mean (SD)†/Median* (IQR) overall (n=33)**	11(5 , 30)*	3(0 , 7)*	2(1 , 3)*	2(1 , 7)*	3(0 , 8)*	1(0 , 3)*	1(0.5)†
**Qualitative case studies**							
**High adherence at end**							
R01/39	8	3	1	1	0	1	1
R02/07	5	1	1	1	2	0	1
R01/40	52	0	1	1	3	0	1
R02/52	70	5	3	1	17	1	1
**Medium adherence at end**							
R01/49	30	2	1	0	1	0	1
**Low adherence at end**							
R01/54	24	4	5	3	4	2	1
R01/02	3	0	1	2	0	1	2
R01/48	38	6	2	7	7	1	1
R02/12	98	12	10	13	14	8	1
R02/03	15	12	1	25	1	14	1
R01/44	11	0	2	4	8	3	1
**Withdrawn**							
R01/42	69	18	9	16	20	3	2
R02/02	3	7	1	8	8	7	1
R02/42	0	0	0	0	0	0	0

For peer review only

Table e. Activities: highly motivated participants

(Those who answered 'No' to question, 'Was the participant motivation too low) n=17. Some of these were missing or not answered n=14, only 2 answered 'Yes'.

	Self-monitoring adherence	Tailored education about treatment	Tailored patient stories (videos)	Personalised action plan/Personalised goal-setting	Tailored problem-solving		Goal review; Rewards
	Clicks How am I doing?	Clicks Toolkit	Clicks Videos	Clicks Action Plan	Clicks Problem Solving	Clicks Coping Plan	Review sessions with Interventionist
**High motivation Mean (SD)†/Median* (IQR) overall (n=17)**	16 (5 33)*	5 (2 ,12)*	3 (1 , 4)*	4 (2 , 12)*	4(2 , 11)*	1(1 , 7)*	1.12(0.33)†
**Qualitative case studies (high motivation)**							
**R02/07**	5	1	1	1	2	0	1
**R02/52**	70	5	3	1	17	1	1
**R01/49**	30	2	1	0	1	0	1
**R01/02**	3	0	1	2	0	1	2
**R02/12**	98	12	10	13	14	8	1
**R02/03**	15	12	1	25	1	14	1
**R02/02**	3	7	1	8	8	7	1

Table f. Process Outcomes

	Accurate awareness of adherence	Increased Motivation	Increased necessity and decreased concern / beliefs Motivation		Increased self-efficacy /	Motivation	Increased habit /	Reduced CHAOS	Reduced barriers
	Subjective adherence (0-100): Medication Adherence Data Questionnaire	Change in BMQ question 'I want to do all my prescribed medications in the next 2 weeks (0-7)	Change in BMQ Necessities score (2-5)	Change in BMQ Concerns score (1-3)	Change in BMQ question 'I am confident I can do all my prescribed medications in the next 2 weeks (0-7)	Change in PAM activation score (0-100)	Change in SRBAI score (0-28)	Change in CHAOS score (0-24)	Change in no. of BMQ barriers ticked (0-6)
**n Overall**	30	31	31	31	31	31	31	31	31
**Mean (SD) overall**	2.07(27.87)	-0.1(1.27)	0.26(0.58)	-0.19(0.31)	0.06(1.79)	- 2.38(14.01)	0.32(3.92)	0.1(2.75)	-1.84(3.44)
**Qualitative case studies**									
	baseline(change) %	**baseline (change)**			**baseline (change)**				
**High adherence at end**									
R01/39	85(14)	7(0)	0.5	-0.4	7(0)	-5.9	-2	2	-4
R02/07	100(-2)	7(0)	0.2	-0.2	7(0)	0	1	-5	-3
R01/40	92(8)	7(0)	0.6	-0.2	5(1)	7.2	-9	0	1
R02/52	95(-25)	7(0)	0.3	-0.2	7(0)	4.9	3	-1	1

For peer review only

R01/49	100(0)	7(0)	-0.8	-0.7	7(0)	9.9	-1	0	-4
**Low adherence at end**									
R01/54	60(-10)	7(-1)	-0.3	0.4	6(0)	-7.9	1	-1	6
R01/02	55(16)	7(0)	0.8	-0.2	2(3)	0	-1	-1	-2
R01/48	0(100)	7(0)	0.9	-0.8	6(0)	0	0	0	-2
R02/12	NA	7(0)	-0.1	-0.7	4(0)	14.6	-3	-2	-5
R02/03	50(NA)	1(NA)	NA	NA	2(NA)	NA	NA	NA	NA
R01/44	0(0)	7(0)	1.4	0.2	5(-4)	-16.6	0	-1	-5
**Withdrawn**									
R01/42	0(0)	5(-1)	-0.3	-0.1	4(0)	-5	-1	5	-6
R02/02	80(10)	7(0)	0.1	-0.5	7(0)	9.2	2	-1	1
R02/42	100(0)	7(0)	0.9	0	7(0)	-12.1	1	7	-1

For peer review only

Table g. Intermediate Outcomes

	End of trial adherence (day 150-180)✦	Change in Objective adherence✦ (%)	Change in FEV1	Number of exacerbations in 6 months
**Mean (SD)⁺/ Median (IQR)* overall (n=33)**	34.7 (0.4 ,78)*	1.25(-5.8 , 36.3)*	0.1(0.51)⁺	1(0 , 2)*
**Qualitative case studies**				
**High adherence at end**				
**R01/39**	95.2	95.16	-0.02	1
**R02/07**	93.5	-3.12	NA	0
**R01/40**	88.2	45.07	0.22	0
**R02/52**	83.9	-12.68	-0.13	0
**Medium adherence at end**				
**R01/49**	68.3	55.06	-0.12	3
**Low adherence at end**				
**R01/54**	29	-15.8	-0.03	2
**R01/02**	29	-1.14	0	0
**R01/48**	5.2	3.34	1.07	0
**R02/12**	0	-10.23	-0.21	0
**R02/03**	0	-5.42	NA	NA
**R01/44**	0	-19.54	0.9	1
**Withdrawn**				
**R01/42**	NA	NA	0	0
**R02/02**	NA	NA	-0.04	3
**R02/42**	NA	NA	0.35	1

✦Normative numerator adjusted adherence

For peer review only

Additional File 04: Joint display table (data sources in **bold**)

#	Logic model column / construct	Quantitative	Qualitative	Convergence code
	**INPUTS**			
1	MDT introduction to CFHealthHub	-	**Chief investigator reported:** introducing MDT to concept behind and application of CFHH.	-
2	CF Clinicians aware of the importance of monitoring adherence	-	**Chief investigator reported:** briefing collaborating MDTs. Reported change agents at centres internalised idea; some residual scepticism among senior physicians.	-
3	Prescription data	Study team found adherence levels of over 100% **(Implementation log, 01 Dec 16)**	Late identification of prescription changes found to be responsible. **(Minutes, Trial Management Group Meeting 10 Jan 17)**	Expansion
4	Chipped nebuliser	-	Devices ordered centrally by CTRU were delivered to sites on 20th May 2016 and processed for distribution on 23rd June 2016. **(Project manager emails)**	-
5	Qualcom-Hub (docking & upload)	-	Devices ordered centrally by CTRU were delivered to sites on 20th May2016 and processed for distribution on 23rd June 2016 **(Project manager emails)**	-
6	CFHealthHub website/app	-	Available, but under development through trial **(Additional File 01)**	-
7	COM-BMQ questionnaire	COM-BMQ questionnaire data was collected at baseline for all consenting participants	-	-

For peer review only

	responses	**(Additional File 04 - Table 8)**		
8	Intervention manual	-	High levels of interventionist satisfaction with manual. R01 Interventionist 1 remarked that, "all the stuff in the manuals was really good."	-
9	Interventionist training programme	**Structured questionnaire on interventionist confidence after training programme:** Interventionists (n=5) all averaged >8 for confidence across 11 questions. Isolated scores of <8 occurred three times: viewing charts/tables, completing report forms and understanding online training/assessment.	**In interviews**, interventionists reported high levels of satisfaction; one requested for more integration of research and intervention procedures. R01 Interventionist 1 remarked "You had the manual but I was missing bits". She wanted more case studies and mock patients in the training to compensate for this. An interventionist (R01 MDT member 1), who was a social worker by background, found the training very good, indicating that it the training had acceptability beyond physiotherapists.	Expansion
10	Interventionist support	-	**Research team member (MH) reported** giving mentorship and that one site/trust received more support from the PI than the other. The main interventionist at the other site received support from the part-time interventionist who was a member of the multi-disciplinary team.	-
11	Competency/Fidelity assessment	**Structured instrument for the assessment of interventionist competence:** Digital recordings were made and assessed for fidelity by MA, MH and JB. Fidelity assessment instrument modified after discussion, in advance of use on full-scale RCT.	-	-

For peer review only

12	Motivated and effective interventionists	-	**In interviews**, interventionists reported that they were enthusiastic about the intervention	-
	**ENGAGEMENT**			
13	Clinicians accessing adherence data*	Clinicians did not access CFHH. **(CFHH Click analytics)**	**In interviews**, interventionists talked about run charts occasionally being viewed when brought to MDT meetings by interventionists.	Confirmation
14	Adherence data tracking	**CFHH click analytics** showed interventionists accessing data before meetings	This was confirmed **in interviews**.	Confirmation
15	Participant accessing CFHealthHub	**Click analytics:** The median number of sessions over 5 (+/- 1) months was 3 (interquartile range 1 to 8, range 1-44, **Additional File 05 - Table c**), with a mean duration of 36.1 (SD=23.9) minutes. The mean total duration of interaction time across the study was 49.3 (SD 44.8) minutes. The mean length of an interaction was 12.4 (SD=9.6) minutes. The median number of days in the trial with interactions was 2 (IQR=1,7).	Lack of usability was explained **in interviews** by initially difficult login procedures and the lack of a mobile app for most of the pilot trial, leading participants to access an unsatisfactory desktop version on their mobile.	Expansion
16	Push notifications/reminders each week*	-	**Programmer reported that** automated push notifications not available during pilot trial. **In interviews**, one participant and one interventionist, reported the spontaneous development of informal push notifications in which the interventionist was ringing up and praising the participant for accomplishments, thereby building the relationship.	-
17	CFHealthHub Intervention sessions delivered according	**Collected via project-specific structured fidelity assessment instrument (#11).** After discussion	**Fidelity observations indicated:** limited discussion of motivations; communication style	Expansion

For peer review only

	to Manual (Fidelity)	between MA, MH and JB summary scores were agreed for delivery of content 100% and quality of delivery: 60-92%. Co-author Judy Bradley is intending to publish this work elsewhere.	sometimes paternalistic rather than autonomy-enabling; insufficient attention to most active ingredients.	
18	Initial session, and then review at each clinic visit	**Collected via click analytics.** Patient run charts reveal a disparity in when and whether these happened **(Additional File 07)**.	-	-
	**ACTIVITIES**			
19	Clinicians monitor adherence	-	Clinician access to adherence data was sporadic (see #13) and **staff interviews confirmed** that it was not monitored. **In an interview**, participant R01/02 described the research intervention as “parallel rather than integrated” with mainstream clinical management.	-
	Intervention components for all participants			
20	Self-monitoring adherence	**Click analytics:** 'How am I doing?' pages were the most frequently visited in terms of the total number of clicks during the trial. 30 (90.9%) participants clicked a median of 11 (range 5-30) times in 5 months, but sometimes in a single session **(Additional File 05 – Table d)**. Access did not always result in good alignment between subjective and objective adherence **(Additional File 05 – Tables f and g respectively)**.	**In interviews**, moderate and frequent users said they mostly valued this page for self-monitoring.	Expansion
21	Tailored education about treatment	**Click analytics:** Toolkit clicked a median 3 (range 0-7) times **(Additional File 05 – Table d)**.	**In participant interviews**, the DNASE video was popular. Other pages were accessed	Expansion

			infrequently or when issues arose, when the information was viewed as "more down to earth" (R02/07) than technical manuals.	
22	Tailored patient stories (videos)	**Click analytics:** 'Talking heads' videos accessed a median 2 (range 1-3) times **(Additional File 05 – Table d)**.	**In participant interviews**, these videos divided opinion. Some participants liked to know that they were not alone; others did not want to see videos of others with CF.	Expansion
	Intervention components for those with adequate motivation			
23	Personalised goal-setting	**Click analytics:** Participants set target adherence levels in CFHH **(Additional File 05 – Table 3)**.	**In interviews**, participants reported goal-setting, but it was not clear how much it came from patients and how much from interventionists.	Expansion
24	Goal review	**Click analytics:** Mean (SD) review sessions 1 (0.5) **(Additional File 05 – Table e)**.	-	-
25	Personalised action plan	**Click analytics:** Action plan pages clicked on median 2 (inter-quartile range 1-7) times **(Additional File 05 – Table e)**.	Disliked by some participants who, the interventionist from centre R01 **reported during an interview**, found writing down action plans like "being at school"	Expansion
26	Tailored problem-solving	**Click analytics:** Problem solving and coping plan pages clicked on median 3 (inter-quartile range 0-8) and 1 (0-3) times respectively **(Additional File 05 – Table e)**.	**In interviews**, one participant realised that when she goes to her friend's house, rather than missing a treatment she could do it in the car or anywhere. One interventionist from centre R02 thought it important that the information was	Expansion

For peer review only

			"there if you need it" for patients.	
	**IMMEDIATE OUTCOMES**			
27	Medical care informed by adherence	-	**Interviews** with PIs found that the trial and intervention ran alongside usual care rather than being informed by it (see also #13, #19).	-
	For all participants			
28	Acute awareness of adherence / increased Motivation	Answers to the **subjective adherence question (Additional File 05 – Table f)** were well aligned with **run charts (Additional File 07)** in those with high adherence. Alignment was more variable in those with moderate and poor adherence.	**In interviews**, some with high adherence used the CFHH "How am I doing page" (run charts) as a check (R02/07, R01/40); other high adherers did not (R01/49). Some felt that it increased their adherence, acknowledging that monitoring meant that they had, "…better make an effort here".	Expansion
29	Increased necessity and decreased concern	No change in the group averages for the **COM-BMQ** (incorporating Beliefs about Medicines Questionnaire - specific (Nebuliser adherence) 21-item validated self-report tool[1]) or Patient Activation Measure (**PAM-13**) (Health Style Assessment) assessment of patient knowledge, skill, and confidence for self-management[2]. **(Additional File 05 – Table f)**	-	-
30	Increased self-efficacy / Motivation	No change in the group averages for a single question about confidence to adhere or the PAM-13. **(Additional File 05 – Table f)**	-	-
	For those with adequate			

For peer review only

	motivation			
31	Increased self-efficacy/ Motivation	No change in the group averages for a single question about confidence to adhere or the **PAM-13**. **(Additional File 05 – Table f)**	-	-
32	Increased habit / Reduced CHAOS	No change in the group averages for **Self-Report Behavioural Automaticity Index (SRBAI)** automaticity-specific subscale of the Self Report Habit index to capture habit-based behaviour patterns[3] or in the assessment of routine measure of **life chaos**[4]. **(Additional File 05 – Table f)**	-	-
33	Reduced barriers	No change in the group averages for The Beliefs about Medicines Questionnaire - specific (Nebuliser adherence) (**BMQ 21-item**) **(Additional File 05 – Table f)**	The tailored problem-solving modules (#26) were not widely used but, **in interviews**, party plans and nebuliser guides were cited as having removed barriers by those who did use this content. For instance, one participant was able to find the technical name for a part of a nebuliser for which he needed to order a replacement.	Expansion
	**INTERMEDIATE OUTCOMES**			
34	Treatment optimisation	-	**Interview data** revealed patients to be behaving in unexpected ways, for instance taking holidays from their treatment or not taking medication as prescribed.	-
35	Increased adherence	**Nebuliser data via CFHH:** Mean adherence across all participants was 10 (95% CI: -5.2 to 25.2) percent higher in the intervention than in the	-	-

		control arm. Within the case study participants (all intervention), an increase of 7.5% (95% CI: -8.2-23.1) in simple normative adherence with numerator adjustment can be observed in the intervention arm. Following month 1, adherence is consistently higher in the intervention arm with the greatest difference observed in month 5 (mean difference: 10.8, 95% CI: -11.44, 22.9). These differences would indicate a potentially clinically important difference between the intervention and usual care arms.		

[1] R. Horne, J. Weinman, M. Hankins, The beliefs about medicines questionnaire: The development and evaluation of a new method for assessing the cognitive representation of medication, Psychol. Health. 14 (1999) 1–24. doi:10.1080/08870449908407311.

[2] J.H. Hibbard, E.R. Mahoney, J. Stockard, M. Tusler, Development and testing of a short form of the patient activation measure., Health Serv. Res. 40 (2005) 1918–30. doi:10.1111/j.1475-6773.2005.00438.x.

[3] B. Verplanken, S. Orbell, Reflections on Past Behavior: A Self-Report Index of Habit Strength1, J. Appl. Soc. Psychol. 33 (2003) 1313–1330. doi:10.1111/j.1559-1816.2003.tb01951.x.

[4] M.D. Wong, C. a. Sarkisian, C. Davis, J. Kinsler, W.E. Cunningham, The Association Between Life Chaos, Health Care Use, and Health Status Among HIV-Infected Persons, J. Gen. Intern. Med. 22 (2007) 1286–1291. doi:10.1007/s11606-007-0265-6.

For peer review only

Additional File 05 - Case-ordered descriptive matrix for fourteen case studies

Qualitative findings in italics. Otherwise, motivation, confidence, necessities, concerns, life chaos and subjective adherence (baselines and process outcomes) from self-report instruments (see Methods and Additional File 04). Engagement, activities and data captured by CFHealthHub.

Case / baselines / context	Engagement	Activities	Process outcomes	Intermediate outcomes
**High adherence (average >80%) in last month of trial**				
**R01/39.** High motivation, confidence and necessities, medium concerns, quite high chaos. *They got a lot of information about CF from other websites.*	Used CFHH once. *Very engaged with interventionist and trial.*	*Didn't make plans – felt it was her responsibility to adapt her life; found others monitoring helpful. Didn't like videos or social aspects of website because of the reminder of her mortality.*	*Knowledge that clinicians could access treatment adherence information provided extra motivation to adhere.*	End of trial adherence 95% (95% improvement).
**R02/07.** High motivation, and confidence, medium-high necessity, medium concerns and chaos. Existing high adherer, *sees treatment as a "plan for longevity" rather than a "chore".*	Used CFHH twice. *Didn't find it useful or like the videos (doesn't want to see negative side of CF).*	Made action plan, accessed some modules once. *Found goal-setting with interventionist helpful.*	Little change as already, motivated. Reduced CHAOS and barriers.	End of trial adherence 93% (3% decline).
**R01/40.** High motivation, medium confidence and necessities, low concerns, medium-to-low chaos. *Was recruited soon after exacerbation.*	Had nine CFHH sessions. *"I've been logging on to track my progress... every two weeks to a month". Finds others monitoring him helpful.*	Frequent self-monitoring. *Compensates for slippages by planning to do the rest of his doses.*	Motivation already high, but habit lacking. *Intervention has made him think about adherence more than he did before.*	End of trial adherence 88% (45% improvement). Variance over trial, but trajectory.
**R02/52.** High motivation, confidence and necessity, low concerns, low-medium chaos. Existing good adherer; *wanted something like a fitness tracker with feedback - messages on performance.*	13 CFHH sessions. *Liked the more portable nebuliser, could take it away on work.* CFHH session that precedes interventionist visit explained by interventionist testing login details.	Frequent self-monitoring, regular use of tailored education and problem solving (fixing nebuliser problems) and some use of videos. *Wanted it expanding to physical activity.*	Motivation already high. Increased habit.	End of trial adherence 83% (12% decline).

**Case / baselines / context**	**Engagement**	**Activities**	**Process outcomes**	**Intermediate outcomes**
**Moderate adherence (average 50-80%) in last month of trial**				
**R01/49.** High motivation, confidence, medium-high necessity and concerns low chaos. *Participated to 'prove' themselves to their physiotherapist*; poor awareness of own adherence not improved over course of trial.	4 CFHH sessions	Used problem-solving modules and self-monitoring, but no action plan.	Increased motivation, reduced barriers.	End of trial adherence 68% (55% improvement). An important improvement from low adherence, but subjective adherence still poorly 'calibrated' with objective adherence.
**Poor adherence (>50%) in last month of trial**				
**R01/54.** Professed high motivation and confidence, medium necessity, low concerns, medium to low chaos. *Wants the doctor "to notice" that they are adherent to their treatment, demotivated by the fact they don't.*	44 CFHH sessions. *Appreciative of extrinsic motivation from face-to-face contact with interventionist.*	Frequent self-monitoring; initially high use of action plans and problem solving. *Dislikes 'talking heads' videos.*	More barriers by the end of the trial.	End of trial adherence 29% (16% decline), but run chart shows huge variance week by week.
**R01/02.** High motivation, low confidence, medium necessity and concerns, high chaos. *Dissatisfaction at service reconfiguration: moved across from Poole to Southampton during trial. Upset that wider team isn't noticing their adherence.*	Used CFHH once *but had technical problems. Appreciative of interventionist: "Having a personal contact and someone to guide you through it is really useful" Wider team not talking about adherence: "parallel rather than integrated".*	Two review sessions with interventionist.	Reduced CHAOS and barriers; increased self-efficacy	Lack of pre-post change not contradicted by the run chart which shows improvement.

For peer review only - http://bmjopen.bmj.com/site/about/guidelines.xhtml

Case / baselines / context	Engagement	Activities	Process outcomes	Intermediate outcomes
**R01/48.** professed high motivation and confidence, medium-high necessities and concerns; medium chaos. *This 69-year old doesn't like nebulising; "can't teach an old dog new tricks". No belief in benefit of nebulised medication. Poor awareness of own adherence. Altruistic trial participant.*	Used CFHH three times. *Access problems (passwords, etc) - gave up.*	Some engagement with toolkit, action plans and problem-solving, *didn't like the videos.* Engagement drops off as soon as the last meeting over.	No change in process outcomes.	End of trial adherence 5% (3% improvement). *Said was making an effort for the trial.* In line with this, objective adherence was high (~80%) for weeks 6-21
**R02/12.** High motivation, medium to low confidence, medium to high necessity and concerns, medium chaos.	Started off engaged, lots of CFHH use and two intervention sessions in first 100 days, nothing thereafter.	Made plans, liked website, checked graphs. *Liked face-to-face interaction with interventionist.*	Decreased chaos and barriers but also decreased habit.	Initial improvement in adherence (up to 100% between weeks seven and nine after first intervention not sustained over time. Review stimulates brief improvement at week 15, again not sustained.

For peer review only

Case / baselines / context	Engagement	Activities	Process outcomes	Intermediate outcomes
**R02/03.** Low motivation and confidence, medium necessities, concerns and chaos. *Treatment is something that he has to do but doesn't want to do it, or think about CF. Forgets about treatment because of busy lifestyle. Prioritises other things above health. Knows that this doesn't end well, but no readiness to change.*	Minimal short-term engagement with CFHH. *Interventionist notes that participant has always been difficult to get hold of.*	Made action and coping plans, checked graphs.	No process data at follow-up.	Withdrew from treatment early.
**R01/44.** High motivation, medium confidence, necessities, low concerns, high chaos (*"I can't seem to get into a routine"*). *Recruited during exacerbation:* baseline artificially high. Intervention 1 visit didn't happen until Week 17. *Participant describes self as "uncompliant" except around inpatient stays.*	One CFHH session (at intervention visit 1). Interventionist appears not to have done correct preparation. Only participant rated by an interventionist as having inadequate motivation.	*Participant confirms that he made action plan, coping plan and checked graphs with interventionist but chaotic lifestyle and low motivation prevented further use. Admits only has a routine in hospital.*	No change in process variables.	Initial spikes of adherence not sustained over time.

For peer review only - http://bmjopen.bmj.com/site/about/guidelines.xhtml

Withdrawn				
**Case / baselines / context**	**Engagement**	**Activities**	**Process outcomes**	**Intermediate outcomes**
**R01/42.** Medium motivation, low confidence, medium-high necessity, medium concerns, low chaos. *Originally an i-neb user. Does not think nebulising three times a day is achievable. Moved house during study. No broadband – so didn't do nebulisations.*	*Loved the website and shared it.* 41 CFHH sessions. *Intervention visit 1 reported to be chaotic.*	Made action plan.	Little change in process variables.	Interview might have triggered brief increase in nebuliser use, when *participant realised nebulisations were being logged even when he wasn't plugging it in.*
**R02/02.** High motivation and confidence, medium-high necessity low concerns and chaos. *Interview shows them to be motivated by interventionist visit and qualitative interview (Hawthorne effect).* Subjective adherence poorly aligned to objective adherence.	Limited engagement. Three CFHH Sessions all on the same day.	Made an action plan *but reported that she didn't set goals because she thought she her adherence was already good.*	Little change in process variables.	Adherence run chart starts off high, but drops off quickly. Interview might have triggered brief increase in nebuliser use. Withdrew from collection of nebuliser data collection.
**R02/42.** High motivation and confidence, medium to high necessity, low concerns, medium chaos	Withdrew - *didn't like the eTrac nebuliser - delivering the drug too quickly made them cough. Interventionist encouraged discontinuation.*	*Didn't look at the website.*	No change in process outcomes	Assumed no change in adherence, but objective lacking.

For peer review only - http://bmjopen.bmj.com/site/about/guidelines.xhtml

ACtiF Pilot Statistical Report

L Mandefield

Methods

Outcomes

Feasibility outcomes

The primary objective of this study was to determine the feasibility of proceeding to a definitive trial. An external pilot randomised controlled trial to determine feasibility of a randomised controlled trial based on objective stop-go criteria related to:

(a) participant recruitment;

(b) participant retention; and,

(c) quality of primary outcome data at 5 (+/- 1) months post randomisation.

These were assessed by

i. The number of screened, eligible and recruited participants per month, per centre and overall;

ii. The number and percentage of participants who complete their 5(+/-1) month post randomisation follow up;

iii. The number of Fuchs criteria by exacerbation.

Clinical outcomes

The primary clinical outcome measure was the number of pulmonary exacerbations in the 5 (+/-1) month post-baseline follow-up period, defined according to a modified version of the Fuchs criteria. The original Fuchs criteria was 4 out of 16 symptoms leading to IV antibiotic treatment. An exacerbation of respiratory symptoms will be said to have occurred when a participant was treated with parenteral antibiotics for any one of the following 12 signs or symptoms:

1. change in sputum;
2. new or increased hemoptysis;
3. increased cough;
4. increased dyspnea;
5. malaise, fatigue, or lethargy;
6. temperature above 38 °C;
7. anorexia or weight loss;

For peer review only

8. sinus pain or tenderness;

9. change in sinus discharge.

10. change in physical examination of the chest, derived from notes by site staff.

11. decrease in pulmonary function by 10 percent or more from a previously recorded value, derived from notes by site staff; or,

12. radiographic changes indicative of pulmonary infection, derived from notes by site staff.

The trial interventionist or prescribing clinician/nurse will collect data on the "exacerbations" form at the point of a participant starting a course of IV antibiotics.

The following secondary outcomes were also collected at baseline and 5 (+/-1) month follow up:

1. Body Mass Index (BMI).
2. Forced expiratory volume in 1 second (FEV1): standardised spirometry as a measure of condition severity.
3. EuroQol EQ-5D-5L: generic health status measure for health economic analysis.
4. The Patient Activation Measure (PAM-13): assessment of patient knowledge, skill, and confidence for self-management.
5. Confusion, Hubbub, and Order Scale (CHAOS 6-item): measure of life chaos.
6. Medication Adherence Data-3 items (MAD-3)
7. Self-Report Behavioural Automaticity Index (SRBAI)
8. Cystic Fibrosis Questionnaire-Revised (CFQ-R): disease specific health-related quality of life instrument.
9. The Patient Health Questionnaire depression scale (PHQ-8): severity measure for depressive disorders.
10. The General Anxiety Disorder 7-item anxiety scale (GAD-7): severity measure for anxiety.
11. The Capability Opportunity Motivation Behaviour Beliefs Questionnaire (COM- BMQ): This questionnaire incorporates:
    a. The Beliefs about Medicines Questionnaire - specific (Nebuliser adherence) (BMQ 21-item): a validated self-report tool, customised by the author to identify perceived necessities and concerns for nebuliser treatment.
    b. The following project-specific items: one additional belief item, one intention item, one confidence item, and a list of barriers. These will serve as a tailoring tool for the intervention and also as a secondary outcome measure. 12.Subjective adherence single question: self-report estimate of adherence as a percentage. Self-reported problems: identification of capability and opportunity barriers to nebuliser adherence
13. Concomitant medications: bespoke instrument, designed for this research project.
14. Resource use form: interventionist collects data from a combination of hospital notes and the NHS patient electronic system to determine 1) inpatient IV days 2) Routine clinic visits 3) Unscheduled outpatient contacts 3) unscheduled inpatient stays.
15. Prescription: a monthly prescription check to both check for data transfer to CFHealthHub and review for an indication that the prescription has changed or indication of microorganism e.g.
16. Adherence to prescribed medication
17. Any treatment with IV antibiotics

For peer review only

Sample Size

Sample size calculation was based on estimating parameters within a certain amount of precision rather than hypothesis testing. The sample size for a feasibility study should be adequate to estimate the uncertain critical parameters (standard deviations for continuous outcomes; consent rates, event rates, attrition rates for binary outcomes) needed to inform the design of the full RCT with sufficient precision.

To assess recruitment rate, the external pilot RCT ran in two CF units for 12 months, with four months recruitment, one months 'run-in' period (the period between the consent and baseline visit), and 5 (+/-1) months follow up. To match the proposed recruitment rate of the main RCT, the target sample over the four months for which the pilot RCT was open, was 32 per centre (64 in total from the two pilot centres). We aimed to see a minimum of 75% of the recruitment target to be confident of the trial viability i.e. at least 48 patients in total consented and randomized in four months' of recruitment from two centres.

Randomisation

Randomisation was conducted using a computer generated pseudo-random list with random permuted blocks of varying sizes, created and hosted by the Sheffield CTRU in accordance with their Standard Operating Procedures (SOPs) and was held on a secure server. ACtiF participants will be randomised in a 1:1 ratio, intervention to control arms, stratified by:

- Site;
- Number of IV days in previous 12 months as collected at consent visit (two categories will be (i) less than or equal to 14 days and (ii) greater than 14 days).

Study researchers accessed the allocation for each participant by logging in to the remote, secure internet-based randomisation system. Once a participant had consented to the study, the researcher logged into the randomisation system and entered basic demographic information. After this information had been entered the allocation for that participant was then revealed to the researcher.

Block randomisation with randomly varying block size of 2, 4 and 6 was used so that the sequence of allocation could not be predicted. The block sizes were determined by the trial statistician and block size was not revealed to any other member of the study team.

Blinding

The trial statisticians remained blind until data freeze, at which point unblinded data was presented to them so checks could be carried out.

Statistical Methods

All statistical analyses were performed in R version 3.3.1.

Analysis Populations

The ITT population includes all participants for whom consent was obtained and who were randomised to treatment, regardless of whether they received the intervention or not. This is the

For peer review only

primary analysis set and endpoints were summarised for the ITT population unless otherwise stated.

Participant Flow

A CONSORT flow diagram was used to display data completeness and patient flow from first contact to final follow up.

The number of participants recruited at each centre each month was presented. The number of participants who withdrew consent from the trial, withdrew from the intervention, withdrew from collection of the primary outcome, withdrew consent from adherence data collection and who were lost to follow up were presented overall, by treatment arm and site. The reasons for attrition, where given, were presented.

Patient reported outcome measures (PROMS)

The following PROMS were completed at baseline and 5 (+/-1) month follow up visit. For detailed methods of how these questionnaires were scored, please see the appendix.

Data completeness

A CONSORT flow diagram was used to display data completeness and patient throughput from first contact to final follow up.

Baseline characteristics

Participants' demographics (age, sex, ethnicity, IMD decile), physical measurements (weight, height, BMI), clinical measurements (FEV1, IV days in last registry year, Pseudomonas status, Adherence in first 2 weeks, Subjective adherence, Medication, Treatment burden) patient reported outcomes (EQ-5D-5L, PAM-13, CHAOS, MAD-3,SRBAI, CFQ-R, GAD-7, COMBMQ, PHQ-8). Imbalance between treatment arms was not tested statistically but were reported descriptively.

Primary effectiveness analysis of clinical outcomes

The primary endpoint of the study is the number of exacerbations in a 5 (+/- 1) month period. Exacerbations were defined as being treated with IV antibiotics and meeting at least 1 Fuchs criteria.

The number of exacerbations by participant were presented. The number and percentage of exacerbations with each Fuchs criteria were presented. The length of IV course was summarised by intervention arm for all exacerbations and for participants experiencing exacerbations.

The primary effectiveness analysis used a negative binomial model and included all exacerbations in a 6 month follow up period. Participants who were not followed for this length were excluded. An adjusted model included IV days in the previous 12 months as a covariate. Although not prespecified, a further sensitivity analysis was carried out. This model included the number of days followed up as an offset. This allowed all consenting participants to be included. An adjusted offset model included IV days in the previous 12 months as a covariate.

For peer review only

Secondary effectiveness analysis of clinical outcomes

Patient reported outcome measures

Secondary outcomes were measured at baseline and 5 (+/-1) months post randomisation. The mean difference between treatment arms was calculated for each of the secondary outcomes, along with 95% confidence intervals using a multiple linear regression model. Adjustment for baseline and site was carried out and both unadjusted and adjusted results were presented.

Adherence to medication

The time of inhalations of medication was recorded via chipped nebulisers. This data along with prescription data was used to calculate a number of different adherence measures. Adherence in people with CF is of key importance. For this reason, it was decided that 7 separate measures of adherence to prescribed medication were to be presented:

1. Total doses;
2. Unadjusted adherence;
3. Simple normative adherence (without numerator adjustment);
4. Sophisticated normative adherence (without numerator adjustment);
5. Simple normative adherence (with numerator adjustment);
6. Sophisticated normative adherence (with numerator adjustment);
7. Subjective single adherence.

Measures 1-6 are calculated daily based on the chipped nebuliser data and the dose prescribed that day. Means can be calculated for set periods, e.g. weekly.

The specific calculations of these adherence measured are described below.

Total doses taken

As a basic, unadjusted measure of adherence, the total number of doses taken for the time period will be calculated.

Unadjusted adherence

Adherence is typically calculated as the dose taken divided by the dose described per day.

Simple normative adherence (without numerator adjustment)

Quality of adherence reporting is dependent on the PWCF being prescribed the appropriate medications. Adjusting the denominator of the adherence calculation controls for treatment rationalisation to try reduce treatment burden, which is an approach often seen in people in CF. The simple normative adherence is calculated as follows:

1. If the participant does not have pseudomonas
- Minimum denominator is set at 1 treatment/day.

For peer review only

2. If the participant has chronic pseudomonas
- Minimum denominator is set at 3 treatments/day
3. The participant has chronic pseudomonas and intermittent inhaled antibiotic regimens
- Minimum denominator is 3 treatments/day during 28 day 'on' period
- Minimum denominator is 1 treatment/day during 28 day 'off' period
4. The participant has intermittent pseudomonas
- Minimum denominator is 3 treatments/day for 1 or 3 months depending on the eradication regime
- Minimum denominator is 1 treatment/day for the rest of the time

In calculating normative adherence an expected minimum prescription based on a patient's health state is needed. Most patients take a dose of a mucolytic, and patients meeting the criteria will take two doses of antibiotics. In adherence calculations, participants had their denominator amended to reflect their prescription. A complication arises in denominator adjustments when the antibiotic prescribed is one that is expected to be used in an alternating fashion (e.g. 28 days use, 28 days off). The antibiotic medications Aztreonam Lysine and Tobramycin are normally prescribed in this way; for patients with prescriptions for these medications with periods of more than 28 days without a prescription for an antibiotic, the denominator was adjusted to add in 2 doses / day. After 28 days of substituted antibiotic use, a 28 'day off' cycle was programmed. This cycle was continued until such time as another antibiotic prescription was present.

Sophisticated normative adherence (without numerator adjustment)

The sophisticated normative adherence is calculated as follows:

1. If someone has 'mild genotype', is pancreatic sufficient and has FEV1 > 90%, without Pseudomonas and used <= 14 days intravenous antibiotics in the past 1 year.
- There is no minimum target. Denominator is determined by the agreed prescription between clinicians and participants.
2. If someone is homozygous for class I-III CFTR mutation OR pancreatic insufficient OR FEV1 <= 90%, but without Pseudomonas and used <= 14 days intravenous antibiotics in the past 1 year. Minimum denominator is set at 1 treatment/day.
3. If the person has chronic pseudomonas AND/OR
- the person used > 14 days intravenous antibiotics in the previous year Minimum denominator is set at 3 treatments/day
4. If the person has chronic pseudomonas AND/OR used > 14 days intravenous antibiotics in the previous year but is on intermittent inhaled antibiotic regimens
- Minimum denominator is 3 treatments/day during 28 day 'on' period
- Minimum denominator is 1 treatment/day during 28 day 'off' period
5. If someone has intermittent pseudomonas but used <= 14 days intravenous antibiotics in the past 1 year

For peer review only

- Minimum denominator is 3 treatments/day for 1 or 3 months depending on the eradication regime
- Minimum denominator is 1 treatment/day (or 0, i.e. no minimum target) depending on their genotype, pancreatic status and FEV1 for the rest of the time.

Numerator adjustment in simple and sophisticated normative case

Numerator adjustment occurs only if a daily adherence measure is greater than 100%, thus the maximum daily adherence is set at 100%.

Subjective single adherence

All participants will be asked to estimate their adherence as a percentage at baseline, clinic visits, 5(+/-1) months and any further visits up to 30th April 2017. These subjective measures were presented separately. The question referred to the previous 2 weeks.

Adherence summaries

The mean and SD was calculated for each month of the trial by treatment arm. Weekly numerator adjusted normative adherence was calculated and a mean by treatment arm was calculated and presented as a line graph for the first 25 weeks from randomisation.

Intervention adherence

The intervention comprised of:

(a) a chipped nebuliser to collect adherence data

(b) access for participants and interventionist to the adherence data summaries

(c) an online platform (CFHealthHub) offering summaries of adherence and tailored modules to be used by the health professional when interacting with the participant and independently by the participant.

A number of metrics were collected from CFHealthHub including the timing and date of clicks and the page/module that was clicked on. Interactions with CFHH were defined as a series of clicks with no greater that 15 minute gaps between clicks. Length of each session was calculated and days with interactions were calculated by participant.

The mean, standard deviation (SD), median and interquartile range (IQR) for the CFHH metrics were calculated and presented by participant. The same summary statistics were also presented for length of all sessions. The timing of CFHH interactions in days from randomisation was plotted by participant. The number of clicks per page category (Home, How am I doing?, Treatment etc) was plotted in a bar chart and also presented in a table by participant and by session.

Date and time of sessions with the interventionist were also recorded. The number of sessions with an interventionist and the length of sessions by participant were summarised in a table.

For peer review only

Clinic visits

The number of clinic visits completed by each participant excluding consent and 5 month follow up was recorded. Summary statistics were presented by treatment arm to assess whether ascertainment bias occurred in the intervention arm.

Safety analysis

The number of Adverse Events (AEs) and Serious Adverse Events (SAEs) was recorded and presented by treatment arm. These events were further categorised by the type of adverse event and whether they were related to the intervention.

Protocol non compliances

The number and type of protocol non compliances were presented descriptively.

Summary of missing data

The number of missing values or scores for each of the primary and secondary outcomes was presented by baseline and 5 (+/-1) months post randomisation and by treatment arm. Furthermore, the number and percentage of missing items was presented for each of these questionnaires.

For peer review only

Results

Participant Flow

Participants were recruited for 4 months across 2 sites. The CONSORT flow diagram (Fig.1) shows the flow of participants through the trial. 32 participants were randomised at each site. 33 participants were randomised to the intervention arm and 31 participants were randomised to usual care. A total of 59 participants completed the 5 (+/- 1) month follow up visit (Intervention = 31, Usual care = 28).

A total of 8 participants discontinued the trial before the follow up visit (Intervention = 4, Usual care = 4). Of these discontinuations, 5 no longer had their adherence data collected and the same 5 participants did not have their primary outcome collected. Of those who did not continue with primary outcome collection, 2 participants died, 1 withdrew consent and 2 were lost to follow up.

Following the 5 (+/-1) month visit, adherence data and primary outcome data was collected. 2 participants withdrew from adherence data collection during this time (Intervention =1, Usual care =1). 59 participants completed primary outcome data collection up to study completion on 30th April 2017 (Intervention = 31, Usual care =28).

For peer review only

Recruitment by centre and month

For peer review only

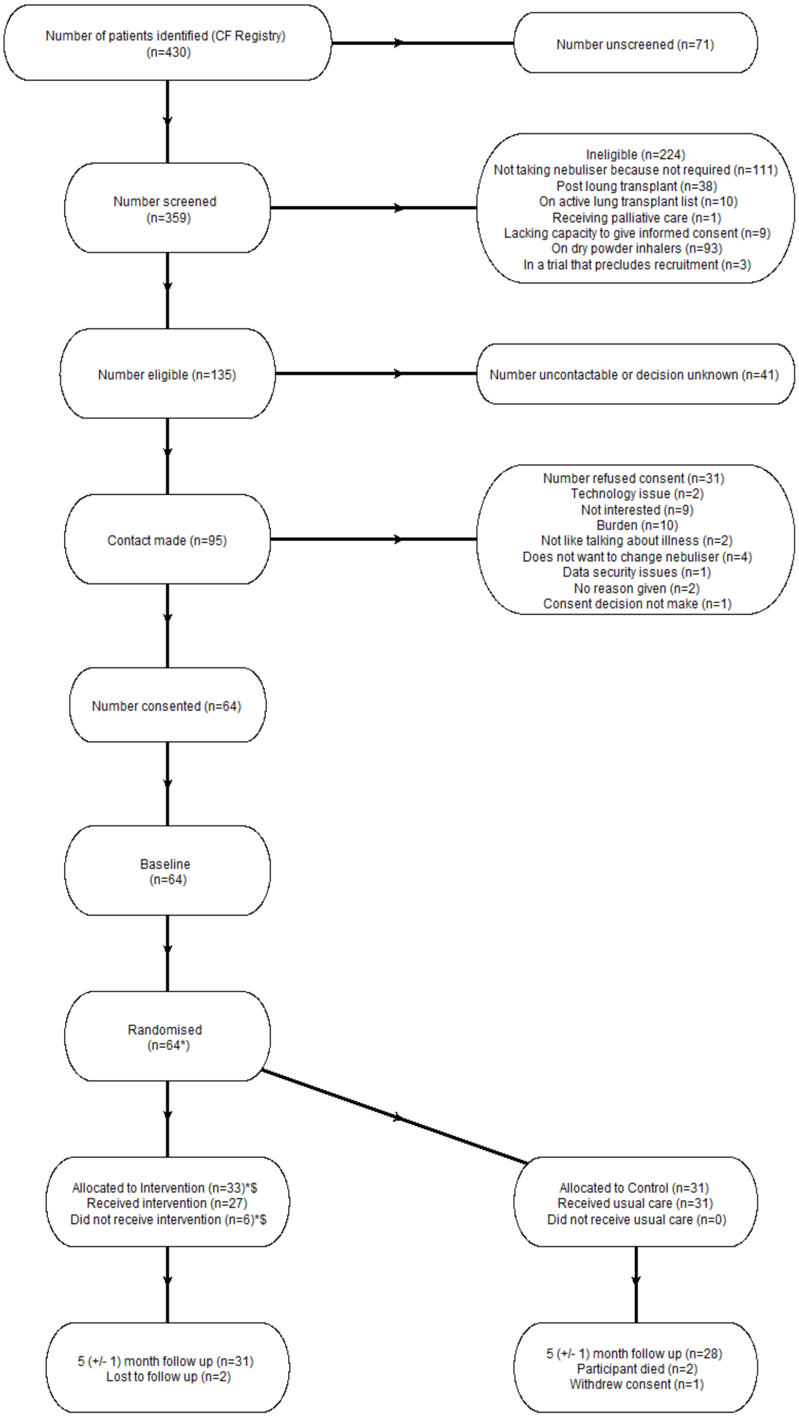

Figure 1: CONSORT flow diagram for ACtiF pilot study.

Table 1: Participants consented by centre and by month

	June 16	July 16	Aug 16	Sept 15	Total
Site A	4	16	7	5	32
Site B	2	17	5	8	32

Attrition by Centre and Treatment arm

Table 2: Attrition presented by treatment arm and site.

		n	Withdrew Consent (%)	Died (%)	Lost to Follow up (%)	Overall (%)
**Overall**		64	1(17%)	2(33%)	2(40%)	5(7.8%)
**Treatment arm**	Intervention	33	0(0%)	0(0%)	2(40%)	2(6.1%)
	Usual Care	31	1(20%)	2(40%)	0(0%)	3(9.7%)
**Site**	Site A	32	0(0%)	1(20%)	1(20%)	2(6.2%)
	Site B	32	1(20%)	1(20%)	1(20%)	3(9.4%)

For peer review only

Baseline characteristics

Table 4 shows the baseline characteristics of participants randomised by treatment arm. 33 participants were randomised to the intervention and 31 were randomised to usual care. The average age of participants was 29.7 (SD=11.5). Participants in the intervention arm were slightly older (median=28, IQR=(21,37)) than those in the usual care arm (median=26, IQR=(20,34)). Table 5 shows the CF measures presented by treatment arm. Tables 6-7 show the baseline questionnaire scores presented by treatment arm.

Baseline demographics

Table 3: Baseline demographics by treatment arm

	Intervention	Control	Overall
Age			
n	33	31	64
Mean(SD)	31.6(13.3)	27.8(8.9)	29.7(11.5)
Median(IQR)	28(21,37)	26(20,34)	27(21,36)
Min,Max	(16,69)	(16,50)	(16,69)
Sex			
Male	18(54.5%)	18(58.1%)	36(56.2%)
Female	15(45.5%)	13(41.9%)	28(43.8%)
Socioeconomic Status			
Most deprived	6(18.2%)	1(3.2%)	7(10.9%)
High deprivation	4(12.1%)	7(22.6%)	11(17.2%)
Average	8(24.2%)	8(25.8%)	16(25%)
Low deprivation	6(18.2%)	9(29%)	15(23.4%)
Least deprived	9(27.3%)	6(19.4%)	15(23.4%)
Weight (KG)			
n	33	31	64
Mean(SD)	65.5(18)	63.7(15.6)	64.6(16.8)
Median(IQR)	63(53,76)	62.9(49,74)	63(52.9,74.3)
Min,Max	(35,128)	(35.6,103.7)	(35,128)
Height (cm)			
n	33	31	64
Mean(SD)	168.6(10.5)	167.7(9.6)	168.2(10)
Median(IQR)	170(162,177)	168(159,175)	168.5(160.5,175.5)
Min,Max	(147,193)	(149,186)	(147,193)
BMI			
n	33	31	64
Mean(SD)	22.8(5)	22.4(4.3)	22.6(4.6)

For peer review only

Median(IQR)	22.2(19.7,25.3)	22.1(19.1,25.4)	22.1(19.55,25.35)
Min,Max	(15.8,42.8)	(16,33.9)	(15.8,42.8)

Table 4: Baseline CF measures by treatment arm

	Intervention	Control	Overall
No. of IV days in previous 12 months			
n	33	31	64
Mean(SD)	26.3(25.7)	26(22.1)	26.2(23.8)
Median(IQR)	17(7,44)	28(0,44)	17(7,44)
Min,Max	(0,117)	(0,70)	(0,117)
No. of participants requiring IV days in previous 12 months			
At least 1 IV day	26(78.8%)	23(74.2%)	49(76.6%)
Days since last IV start date			
n	31	28	59
Mean(SD)	168.7(245.2)	202.3(325.2)	184.6(283.9)
Median(IQR)	75(45,194)	100(24.5,219.5)	91(39,213)
Min,Max	(6,1085)	(7,1575)	(6,1575)
FEV1			
n	33	31	64
Mean(SD)	2(0.8)	2.3(1)	2.1(0.9)
Median(IQR)	1.9(1.4,2.4)	2.1(1.6,2.8)	1.9(1.5,2.7)
Min,Max	(0.8,4)	(0.6,5)	(0.6,5)
FEV1 % Predicted			
n	33	31	64
Mean(SD)	53.4(19.4)	61.4(22.7)	57.3(21.3)
Median(IQR)	49.2(39.4,61.9)	53.4(43,80)	49.6(41.9,76.7)
Min,Max	(26,103)	(23.2,100.7)	(23.2,103)
Clinician pseudomonas status			
Negative	15(45.5%)	8(26.7%)	23(36.5%)
Intermittent	3(9.1%)	3(10%)	6(9.5%)
Chronic	15(45.5%)	19(63.3%)	34(54%)
Leeds Criteria pseudomonas status			
Negative	15(45.5%)	10(33.3%)	25(39.7%)
Intermittent	4(12.1%)	4(13.3%)	8(12.7%)
Chronic	14(42.4%)	16(53.3%)	30(47.6%)

For peer review only

Subjective adherence			
n	23	20	43
Mean(SD)	65.6(40.1)	67.8(35.4)	66.6(37.6)
Median(IQR)	90(20,99)	80(45,99.5)	90(35,99)
Min,Max	(0,100)	(0,100)	(0,100)
Simple normative adherence (first 2 weeks)			
n	33	31	64
Mean(SD)	0.5(0)	0.5(0)	0.5(0)
Median(IQR)	0.5(0.5,0.5)	0.5(0.5,0.5)	0.5(0.5,0.5)
Min,Max	(0.5,0.5)	(0.5,0.5)	(0.5,0.5)
Treatment Burden			
Low	10(30.3%)	11(35.5%)	21(32.8%)
Medium	16(48.5%)	12(38.7%)	28(43.8%)
High	2(6.1%)	5(16.1%)	7(10.9%)

Baseline outcome measures

Table 5: Baseline outcome measures by treatment arm

	Intervention	Control	Overall
EQ5D-5L			
n	33	31	64
Mean(SD)	0.866(0.121)	0.822(0.151)	0.845(0.137)
Median(IQR)	0.901(0.767,0.951)	0.825(0.737,0.942)	0.872(0.752,0.946)
Min,Max	(0.53,1)	(0.486,1)	(0.486,1)
PAM-13			
n	33	31	64
Mean(SD)	60.4(11.2)	60(13.2)	60.2(12.1)
Median(IQR)	60.6(53.2,67.8)	58.1(48.9,67.8)	60.6(51,67.8)
Min,Max	(36.8,84.8)	(38.1,90.7)	(36.8,90.7)
CHAOS			
n	33	31	64
Mean(SD)	9.8(3.4)	10.1(4)	10(3.7)
Median(IQR)	10(8,11)	10(7,12)	10(8,11)
Min,Max	(4,18)	(4,20)	(4,20)
MAD-3			
n	32	30	62
Mean(SD)	9.8(3.3)	9(3.4)	9.4(3.4)

For peer review only

Median(IQR)	9(8,12.5)	9.5(6,11)	9(8,12)
Min,Max	(3,15)	(3,15)	(3,15)
SRBAI			
n	33	30	63
Mean(SD)	11.5(4.9)	10.2(5.6)	10.9(5.2)
Median(IQR)	12(8,16)	9(4,14)	10(7,15)
Min,Max	(4,20)	(4,20)	(4,20)
GAD-7			
n	33	31	64
Mean(SD)	4.1(4.5)	3.8(3.6)	3.9(4)
Median(IQR)	3(0,5)	3(1,7)	3(0.5,5.5)
Min,Max	(0,15)	(0,11)	(0,15)
PHQ-8			
n	33	31	64
Mean(SD)	7(4.9)	6.5(5.2)	6.8(5)
Median(IQR)	6(3,12)	6(3,8)	6(3,10.5)
Min,Max	(0,16)	(0,18)	(0,18)

Table 6: Baseline CFQR domains by treatment arm

	Intervention	Control	Overall
Physical Functioning			
n	33	31	64
Mean(SD)	48.5(34.8)	49.2(30.8)	48.9(32.7)
Median(IQR)	38(25,88)	42(17,83)	42(21,85.5)
Min,Max	(0,100)	(0,100)	(0,100)
Emotional Functioning			
n	33	31	64
Mean(SD)	70.2(21.1)	62.3(26.1)	66.4(23.8)
Median(IQR)	67(53,93)	67(40,80)	67(53,87)
Min,Max	(27,100)	(7,100)	(7,100)
Eating			
n	33	31	64
Mean(SD)	79.9(24.8)	74.6(27.7)	77.3(26.2)
Median(IQR)	89(67,100)	78(56,100)	89(61.5,100)
Min,Max	(0,100)	(0,100)	(0,100)
Social Functioning			
n	33	31	64

For peer review only

Mean(SD)	65(20.3)	59.6(26.2)	62.4(23.3)
Median(IQR)	67(50,78)	61(44,83)	67(44,83)
Min,Max	(17,100)	(11,100)	(11,100)
Body Image			
n	33	31	64
Mean(SD)	68.5(27.3)	64.9(31.7)	66.7(29.3)
Median(IQR)	78(56,89)	67(44,100)	78(44,89)
Min,Max	(0,100)	(0,100)	(0,100)
Treatment Burden			
n	33	31	64
Mean(SD)	50.5(16.5)	51.6(25.9)	51(21.4)
Median(IQR)	44(44,67)	56(33,67)	50(44,67)
Min,Max	(11,78)	(0,100)	(0,100)
Respiratory			
n	33	31	64
Mean(SD)	53.5(27.5)	54(27.3)	53.7(27.2)
Median(IQR)	50(33,78)	56(33,78)	56(33,78)
Min,Max	(0,100)	(6,100)	(0,100)
Digestion			
n	33	31	64
Mean(SD)	77.9(16.9)	80.4(26.4)	79.1(21.9)
Median(IQR)	78(67,89)	89(78,100)	89(67,100)
Min,Max	(44,100)	(0,100)	(0,100)
Role Functioning			
n	33	31	64
Mean(SD)	65.2(24.3)	64(25.9)	64.6(24.9)
Median(IQR)	67(50,83)	67(42,83)	67(50,83)
Min,Max	(0,100)	(8,100)	(0,100)
Vitality			
n	33	31	64
Mean(SD)	37.8(22.8)	40.6(22)	39.2(22.3)
Median(IQR)	33(17,50)	42(25,58)	42(25,58)
Min,Max	(8,92)	(0,75)	(0,92)
Health Perceptions			
n	33	31	64
Mean(SD)	47.8(27.7)	51.6(24.9)	49.6(26.3)
Median(IQR)	44(22,67)	56(33,67)	44(33,67)

For peer review only

Min,Max	(0,100)	(0,100)	(0,100)
Weight			
n	33	31	64
Mean(SD)	70.7(36.1)	63.4(39.8)	67.2(37.9)
Median(IQR)	100(33,100)	67(33,100)	83.5(33,100)
Min,Max	(0,100)	(0,100)	(0,100)

Table 7: Baseline COM-BMQ domains by treatment arm

	Intervention	Control	Overall
COM BMQ Necessities			
n	33	31	64
Mean(SD)	3.2(0.7)	3.4(0.8)	3.3(0.8)
Median(IQR)	3.1(2.7,3.7)	3.3(2.9,4.1)	3.1(2.7,4)
Min,Max	(2,4.9)	(2,4.7)	(2,4.9)
COM BMQ Concerns			
n	33	31	64
Mean(SD)	2.1(0.6)	2.2(0.6)	2.1(0.6)
Median(IQR)	2.1(1.5,2.6)	2.1(1.7,2.6)	2.1(1.6,2.6)
Min,Max	(1.2,3.4)	(1.1,3.3)	(1.1,3.4)

Primary Analysis

- In total, there were 79 exacerbations in participants followed up for at least 6 months
- Of these, 60 exacerbations fitted our criteria to be included in the primary analysis
  - 18 were not treated with IV antibiotics
  - 1 did not meet any Fuchs criteria
- A total of 60 participants had at least 6 months of exacerbation data (Intervention=32, Control =28)
- 4 participants were excluded
  - 2 died (Control=2)
  - 1 withdrew consent (Control=1)
  - 1 lost to follow up before 6 months (Intervention=1)
- 35 exacerbations occurred in Intervention participants, 25 occurred in Control participants
- 33 participants experienced at least 1 exacerbation (Intervention= 19 (60%), Control= 14 (50%))

The most frequently reported Fuchs criteria (Table 9) were 'Increased cough' (n=52) and 'Change in sputum (n=48). The median number of Fuchs criteria reported per exacerbation included in the primary analysis was 4 (IQR=4,6).

The median IV course length of exacerbations included in the primary analysis was 14 days in both the intervention and usual care arm (Table 12).

For peer review only

As ACtiF was a pilot study, it was not powered to detect an intervention effect. However, differences between treatment arms and their 95% confidence intervals have been calculated (Table 13). The median number of exacerbations was 1 in the intervention arm and 0.5 in the usual care arm. Following adjustment for site and the number of IV days in the previous year, adjusted IRR was 1.12 (95% CI: 0.658-1.94). This demonstrates a small increase in exacerbations in the intervention arm, however the confidence intervals are relatively wide. The IRR from the offset model shows an IRR of 0.958 (95% CI: 0.615,1.5). Here, a small decrease in exacerbations can be observed. As with the previous model, the confidence interval is relatively wide.

For peer review only

Exacerbations summary

Number of Exacerbations

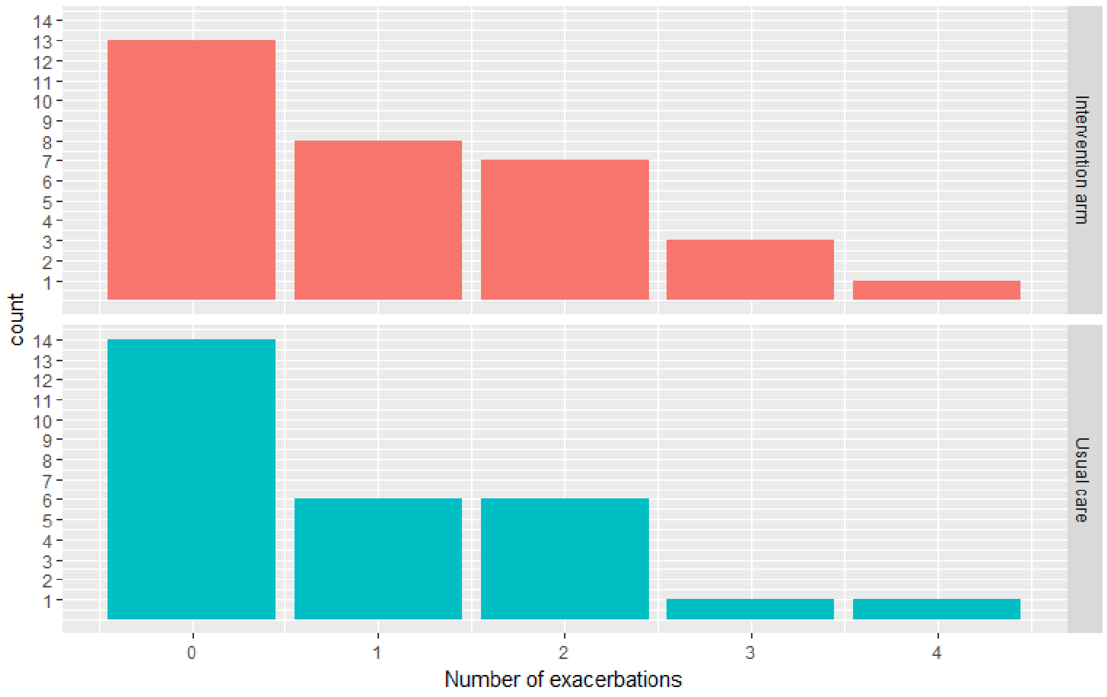

Figure 2:The number of exacerbations in participants by treatment arm in 6 months [n=60]

Fuchs Criteria

Table 8:The number of each Fuchs criterion in the exacerbations used as the primary outcome

	n (%) for exacerbations in 6 months after consent and meeting our criteria (primary outcome)	n (%) for exacerbations treated with IV antibiotics and met at least one Fuchs criteria	n (%) for any exacerbation during the study
**Change in sputum**	48 (80 %)	63 (77.8 %)	69 (69 %)
**New or increased hemoptysis**	12 (20 %)	15 (18.5 %)	16 (16 %)
**Increased cough**	52 (86.7 %)	70 (86.4 %)	77 (77 %)
**Increased dyspnea**	43 (71.7 %)	56 (69.1 %)	61 (61 %)
**Malaise, fatigue, or lethargy**	48 (80 %)	66 (81.5 %)	69 (69 %)
**Temperature above 38 °C**	13 (21.7 %)	18 (22.2 %)	20 (20 %)
**Anorexia or weight loss**	20 (33.3 %)	30 (37 %)	31 (31 %)
**Sinus pain or tenderness**	13 (21.7 %)	19 (23.5 %)	21 (21 %)
**Change in sinus discharge**	13 (21.7 %)	21 (25.9 %)	22 (22 %)
**Change in physical examination of the chest, derived from notes by site staff.**	9 (15 %)	12 (14.8 %)	13 (13 %)
**Decrease in pulmonary function by 10 percent or more from a previously recorded value, derived from notes by site staff**	12 (20 %)	17 (21 %)	19 (19 %)
**Radiographic changes indicative of pulmonary infection, derived from notes by site staff)**	2 (3.3 %)	2 (2.5 %)	2 (2 %)

For peer review only

Table 9:Summary of Fuchs criteria for the exacerbations that were included in the primary outcome (IV days and at least 1 Fuchs criteria in 6 month follow up period

Description	
Exacerbations included in primary analysis	
n (%) with IV and at least 1 Fuchs	60 (60 %)
Mean (SD) number of Fuchs criteria	4.8 (2.1)
Median (IQR) number of Fuchs criteria	4 (4 , 6)
Min, max number of Fuchs criteria	(1,10)
n (%) of exacerbations with at least 2 Fuchs criteria	58 (96.7 %)
n (%) of exacerbations with at least 3 Fuchs criteria	48 (80 %)
n (%) of exacerbations with at least 4 Fuchs criteria	46 (76.7 %)
n (%) of exacerbations with at least 5 Fuchs criteria	29 (48.3 %)
n (%) of exacerbations with at least 6 Fuchs criteria	20 (33.3 %)
n (%) of exacerbations with at least 7 Fuchs criteria	12 (20 %)
n (%) of exacerbations with at least 8 Fuchs criteria	8 (13.3 %)
n (%) of exacerbations with at least 9 Fuchs criteria	3 (5 %)
n (%) of exacerbations with at least 10 Fuchs criteria	1 (1.7 %)

Table 10:Summary of the exacerbations in the 6 month follow up period that were not included in the primary outcome (IV days and at least 1 Fuchs criteria) and the reasons for exclusion

Exacerbations in 6 months not meeting criteria for primary outcome	
Total exacerbations excluded	19 (24 %)
n (%) with IV days but no Fuchs criteria met	1 (1 %)
n (%) with no IV but at least 1 Fuchs	7 (8 %)
n (%) no IV days or Fuchs recorded (missing values)	11 (14 %)

For peer review only

Length of IV course

Table 11:Summary of IV length by exacerbation and participant

	Intervention	Usual Care
IV days per exacerbation in 6 months		
n	35	25
Mean (SD)	13.6(4.2)	13.7(3.3)
Median (IQR)	14(13,14)	14(13,15)
Min, Max	(2,30)	(7,21)
IV days per participant with exacerbations in 6 months		
n	19	14
Mean (SD)	13.4(2.7)	13.6(3.2)
Median (IQR)	14(11,14)	14(13,15)
Min, Max	(9,21.7)	(8,20)
IV days per exacerbation in whole study		
n	45	36
Mean (SD)	13.7(4.1)	13.9(3.1)
Median (IQR)	14(13,14)	14(13,15)
Min, Max	(2,30)	(7,21)

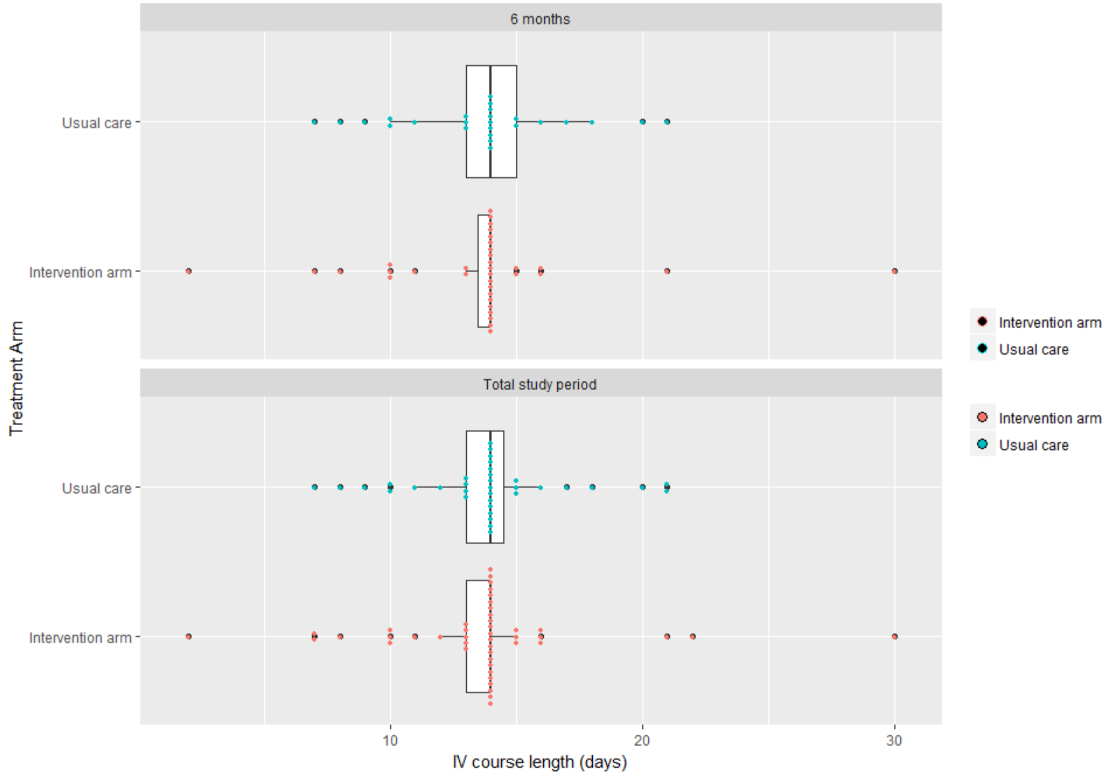

Figure 3:The length on IV courses by treatment arm

Analysis models

6 month model

Table 12:Analysis of the primary clinical outcome, the number of exacerbations treated with IV antibiotics with at least 1 Fuchs criteria in a 6 month period adjusted for site and the number of IV days in the previous year.

	Intervention n	Mean (SD)	Median (IQR)	Control n	Mean (SD)	Median (IQR)	IRR	95% CI
Unadjusted	32	1.1 (1.1)	1 (0 , 2)	28	0.9 (1.1)	0.5 (0 , 2)	1.22	(0.686,2.21)
Adjusted							1.12	(0.658,1.94)

Offset model

Table 13:A sensitivity analysis using all exacerbations treated with IV antibiotics with at least 1 Fuchs criteria that occurred during the study with the number of days of data collection included as an offset in the model. Adjusted for site and number of IV days in the previous year

	Intervention n	Total exacerbations (min,max)	Mean (SD) days followed up	Mean (SD) exacerbations per month	Control n	Total exacerbations (min,max)	Mean (SD) days followed up	Mean (SD) exacerbations per month	IRR	95% CI
**Adjusted, Offset model**	33	46(0,5)	263.2(47.2)	0.17(0.16)	31	40(0,5)	250.5(74.8)	0.2(0.28)	0.958	(0.615,1.5)

For peer review only

Secondary analysis

Tables 15-16 show the results of the secondary analyses. As this is a pilot study, we have not powered to detect any effect. Key results are described below.

- Adjusted mean difference of 5% (95% CI: -2-12%) in FEV % predicted. This is an encouraging difference in the intervention arm.
- No notable differences in any of the other secondary outcomes but this is not of great concern as it is a pilot study.
- Fewer participants had BMI recorded than other outcomes (Intervention=18, Control=15).
- Small reduction in BMQ Concerns score in intervention arm (Mean difference=-0.21, 95% CI: -0.38,-0.048).

Figure 4 shows the distribution of the secondary outcome measures at baseline and follow up by treatment arm.

Table 14:Results of secondary effectiveness analysis

	n Intervention	Median (IQR)	Mean (SD)	n Control	Median (IQR)	Mean (SD)	Mean Diff	95% CI
FEV1 Unadjusted	30	1.8(1.17,2.83)	2(0.9)	27	1.9(1.46,2.83)	2.2(1)	-0.21	(-0.73,0.3)
FEV1 Adjusted							0.22	(-0.062,0.51)
FEV1 % Unadjusted	30	51.8(33.46,71.26)	54.2(21.1)	27	50.9(42.49,77.97)	59(23.9)	-4.8	(-17,7.1)
FEV1 % Adjusted							5	(-2,12)
BMI Unadjusted	18	20.5(19.5,26)	22.1(4.2)	15	23.4(20.7,26.2)	23.8(3.5)	-1.7	(-4.5,1.1)
BMI Adjusted							-0.08	(-1,0.89)
EQ5D-5L Unadjusted	31	0.9(0.76,0.95)	0.9(0.2)	27	0.9(0.77,1)	0.9(0.2)	-0.00062	(-0.084,0.083)
EQ5D-5L Adjusted							-0.016	(-0.087,0.055)

For peer review only

PAM-13 Unadjusted	31	63.1(51,67.8)	58.5(14.3)	28	58.1(51,63.1)	57.9(9.9)	0.56	(-5.9,7)
PAM-13 Adjusted							0.046	(-5.8,5.9)
CHAOS Unadjusted	31	9(7,13)	9.9(3.9)	28	9(7.5,11.5)	9.4(3.3)	0.55	(-1.4,2.4)
CHAOS Adjusted							0.79	(-0.47,2.1)
MAD-3 Unadjusted	31	12(9,13)	10.8(3.9)	26	9.5(7,13)	9.4(3.6)	1.4	(-0.58,3.4)
MAD-3 Adjusted							0.82	(-0.51,2.1)
SRBAI Unadjusted	31	13(8,16)	12.1(5.3)	28	10.5(6,15.5)	10.6(5)	1.4	(-1.3,4.1)
SRBAI Adjusted							0.15	(-1.8,2.1)
GAD-7 Unadjusted	31	3(1,6)	4.1(4.1)	28	2.5(0,7)	4.2(4.4)	-0.05	(-2.3,2.2)
GAD-7 Adjusted							-0.31	(-1.9,1.3)
PHQ-8 Unadjusted	31	7(4,12)	7.3(5.2)	28	4(1.5,7)	5.3(5.1)	2	(-0.68,4.7)
PHQ-8 Adjusted							0.97	(-0.96,2.9)
COM-BMQ Concerns Unadjusted	31	2(1.5,2.3)	1.9(0.5)	27	2.1(1.9,2.4)	2.1(0.5)	-0.22	(-0.48,0.026)
COM-BMQ Concerns Adjusted							-0.21	(-0.38,-0.048)
COM BMQ Necessities Unadjusted	31	3.4(3,4)	3.5(0.6)	27	3.4(2.9,4)	3.5(0.7)	0.011	(-0.35,0.37)

For peer review only

COM BMQ Necessities Adjusted							0.12	(-0.16,0.4)

Table 15:Results of secondary effectiveness analysis

	n Intervention	Median (IQR)	Mean (SD)	n Control	Median (IQR)	Mean (SD)	Mean Diff	95% CI
CFQ-R Physical Unadjusted	31	54(25,88)	54.4(31.6)	28	62.5(33,92)	60.9(31.2)	-6.4	(-23,10)
CFQ-R Physical Adjusted							-2.6	(-13,7.4)
CFQ-R Emotional State Unadjusted	31	67(53,93)	68.3(23.4)	28	73(56.5,90)	72.3(22.7)	-4	(-16,8)
CFQ-R Emotional State Adjusted							-7.7	(-16,0.55)
CFQ-R Eating Unadjusted	31	89(67,100)	80.7(21.6)	28	83.5(67,100)	79.9(20.7)	0.85	(-10,12)
CFQ-R Eating Adjusted							1.1	(-6.5,8.7)
CFQ-R Social Unadjusted	31	67(56,78)	65.4(15.8)	28	64(50,83)	66.4(20.9)	-1	(-11,8.6)
CFQ-R Social Adjusted							-3.7	(-10,2.8)
CFQ-R Body Image Unadjusted	31	78(67,89)	73.3(23.8)	28	78(56,100)	73.1(25.5)	0.19	(-13,13)
CFQ-R Body Image Adjusted							0.62	(-7.2,8.5)
CFQ-R Treatment Burden Unadjusted	31	56(44,67)	56.5(16.6)	28	56(44,67)	57.3(19.9)	-0.83	(-10,8.7)
CFQ-R Treatment Burden Adjusted							1.2	(-6.4,8.8)
CFQ-R Respiratory Unadjusted	31	67(44,78)	59.5(25.2)	27	67(50,83)	65.6(22.7)	-6.1	(-19,6.6)
CFQ-R Respiratory Adjusted							-4.4	(-14,4.8)

For peer review only

CFQ-R Digestion Unadjusted	31	89(67,100)	81.1(18.4)	27	89(78,100)	84.4(23.5)	-3.3	(-14,7.7)
CFQ-R Digestion Adjusted							-2.3	(-11,6.2)
CFQ-R Role Unadjusted	31	75(33,83)	64.8(26.1)	27	75(56,92)	70.3(21.5)	-5.6	(-18,7.1)
CFQ-R Role Adjusted							-8.2	(-17,0.4)
CFQ-R Vital Unadjusted	31	42(25,42)	38.5(19.5)	28	50(33,62.5)	48.7(23)	-10	(-21,0.81)
CFQ-R Vital Adjusted							-7	(-15,0.99)
CFQ-R Health Unadjusted	31	44(22,67)	45.5(25.4)	28	61.5(33,72.5)	56.8(27.6)	-11	(-25,2.6)
CFQ-R Health Adjusted							-6.5	(-16,2.8)
CFQ-R Weight Unadjusted	31	89(67,100)	81.1(18.4)	27	89(78,100)	84.4(23.5)	-3.3	(-14,7.7)
CFQ-R Weight Adjusted							-2.3	(-11,6.2)

For peer review only

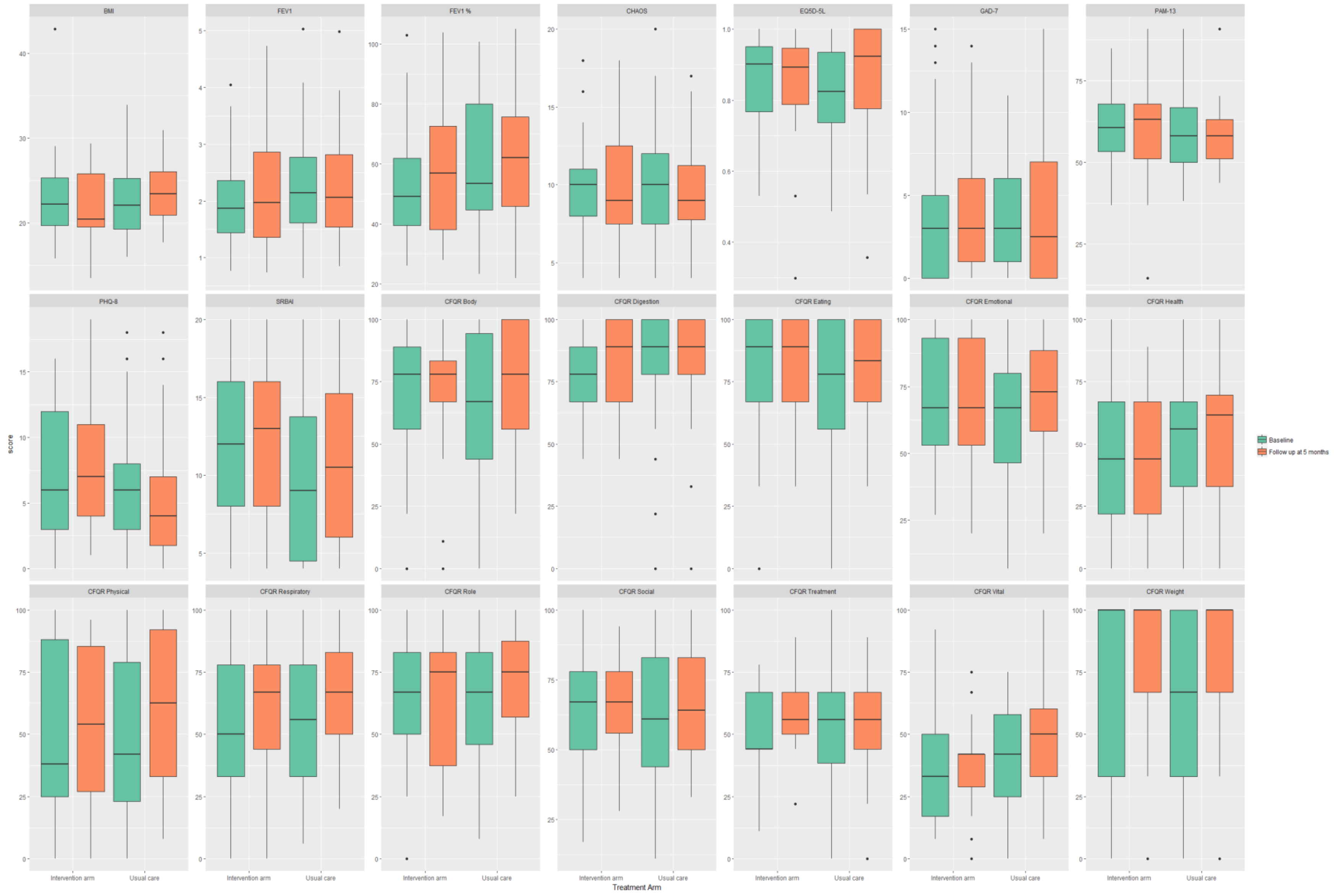

Figure 4:Box plots showing the distribution of secondary outcomes by treatment arm

For peer review only

Adherence to CF medication

During the trial, 8 participants withdrew from adherence data collection (Intervention=4, Control=4). An exact date of withdrawal was not recorded but could be seen from inhalation data (last non zero number of daily inhalations). This has been improved for the main trial and date of adherence data collection withdrawal will be recorded.

Participants who withdrew from adherence data collection were removed from summaries of adherence for 6 months as they did not have 6 months' worth of data. Where possible, inhalation data collected before withdrawal was included in the mean adherence by arm in the monthly table and the plot by week. The number included in each of these estimates can be seen in Table 18.

Table 17 shows the mean adherence by treatment arm for the 6 months post randomisation. Adherence is greater in the intervention arm for each of the different adherence measures. A difference of 10% (95% CI: -5.2 to 25.2) in simple normative adherence with numerator adjustment can be observed in the intervention arm. Table 18 shows the difference in simple normative adherence with numerator adjustment by treatment arm for each individual month in the study. Adherence is greater in the Intervention arm in month 1 (mean difference=2.6, 95% CI: -13.5,18.6). Following month 1, adherence is consistently higher in the intervention arm with the greatest difference observed in month 5 (mean difference: 13%, 95% CI: -4.8, 30.8). These differences would indicate a potentially clinically important difference between the intervention and usual care arms.

The difference in adherence has been presented by weeks post randomisation in Figure 5. There is a difference in numerator adjusted normative adherence with greater adherence observed in the intervention arm. This difference becomes clear after week 4 which coincides with use of the intervention around week 2-3.

Table 16:Summary of average adherences in 6 months following consent by intervention arm and the difference in means with 95% confidence intervals

	n Intervention	Mean Intervention	n Control	Mean Control	Mean Difference (95% CI)
Baseline (first 2 weeks)	29	25.9(31.4)	26	23.2(29)	2.6(-13.9,19.2)
Total doses	29	222.4(233.1)	26	245.7(238.6)	-23.3(-151.2,104.6)
Unadjusted adherence	29	47.7(33.8)	26	37.7(27.1)	10(-6.5,26.4)
Simple normative	29	45.5(32.8)	26	34.7(27)	10.8(-5.4,27)

For peer review only

Sophisticated normative	29	41.6(33.4)	26	34.2(27.1)	7.5(-8.9,23.9)
Simple normative with numerator adjustment	29	43.6(30.4)	26	33.6(25.9)	10(-5.2,25.2)
Sophisticated normative with numerator adjustment	29	39.9(30.9)	26	33.2(25.9)	6.8(-8.6,22.2)

Table 17:Summary of average adherences in each month from following consent from 1 to 6 months by intervention arm

	n Intervention	Mean Intervention	n Control	Mean Control	Mean Difference (95% CI)
Month 1	32	29.7(34.5)	28	27.2(27.5)	2.6(-13.5,18.6)
Month 2	31	42.1(33.1)	28	33.7(31.5)	8.4(-8.5,25.2)
Month 3	30	42.3(33.7)	28	33.3(34.8)	9(-9,27.1)
Month 4	29	42.7(34.7)	27	34.5(30.5)	8.2(-9.3,25.7)
Month 5	29	42.8(36.2)	27	29.8(30.1)	13(-4.8,30.8)
Month 6	29	41.3(36.5)	27	32.9(28.5)	8.4(-9.1,25.9)

For peer review only

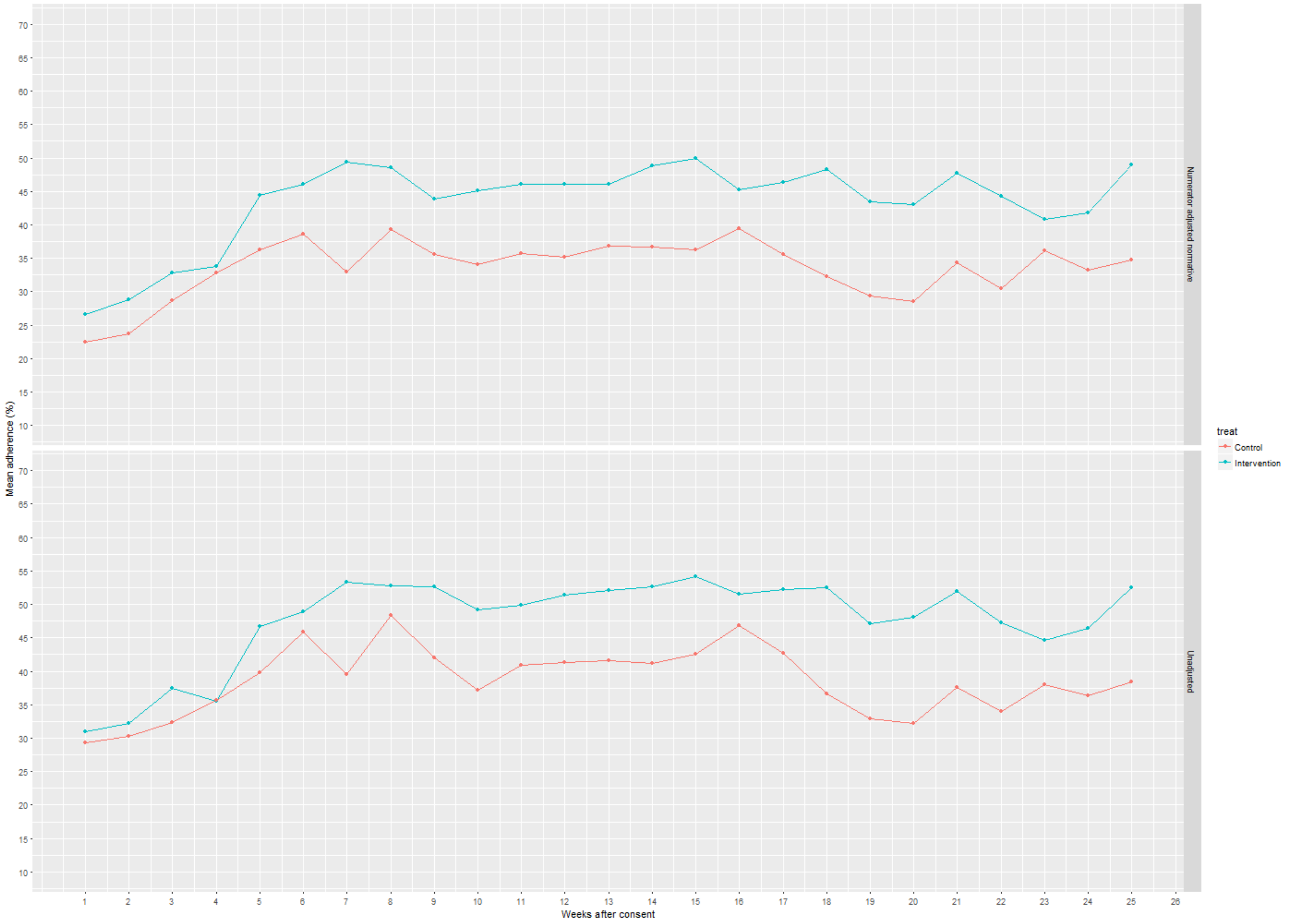

Figure 5:Mean weekly adherence by treatment arm

For peer review only

Intervention adherence (Participants)

Table 19 shows the median number of CFHH interactions was 3 (IQR: 1-8). 3 participants had no interactions with CFHH and the maximum number of interactions was 44. The mean total duration of interaction time across the study was 49.3 (SD= 44.8) minutes. The mean length of an interaction by participant was 12.4 (SD=9.6) minutes and the mean length of all interactions was 6.6 (SD=11) minutes. The median number of days in the trial with interactions was 2 (IQR=1,7) by participant. Figure 6 shows the wide range of values across participants, particularly for the total duration of interactions.

Figure 7 shows when interactions occurred in days for each participant. Some participants were interacting fairly regularly, however most participants were inconsistent with their interactions. Figure 8 shows that the 'How am I doing?' pages were the most frequently visited in terms of the total number of clicks during the trial. 30 (90.9%) of participants visited the 'How am I doing?', 'Treatment' and 'Videos' page at least once (Table 20). 224 (91.4%) sessions included a visit to the 'How am I doing?' page.

For peer review only

Table 18:Summary of clicks in CFHH. An interaction is defined as a series of clicks with no greater than a 15 minute lag between clicks

Interactions with CFHH by participant	
n	33
Mean (SD)	7.4(11.6)
Median (IQR)	3(1,8)
Min, Max	(0,44)
Total duration of interactions by participant	
n	33
Mean (SD)	49.3(44.8)
Median (IQR)	38(26,55)
Min, Max	(0,177)
Mean duration of interactions by participant	
n	33
Mean (SD)	12.4(9.6)
Median (IQR)	10.7(4.3,19)
Min, Max	(0,37)
Days with interactions by participant	
n	33
Mean (SD)	5.7(8.2)
Median (IQR)	2(1,7)
Min, Max	(0,32)
Duration of interactions	
n	245
Mean (SD)	6.6(11)
Median (IQR)	1(0,8)
Min, Max	(0,57)

For peer review only

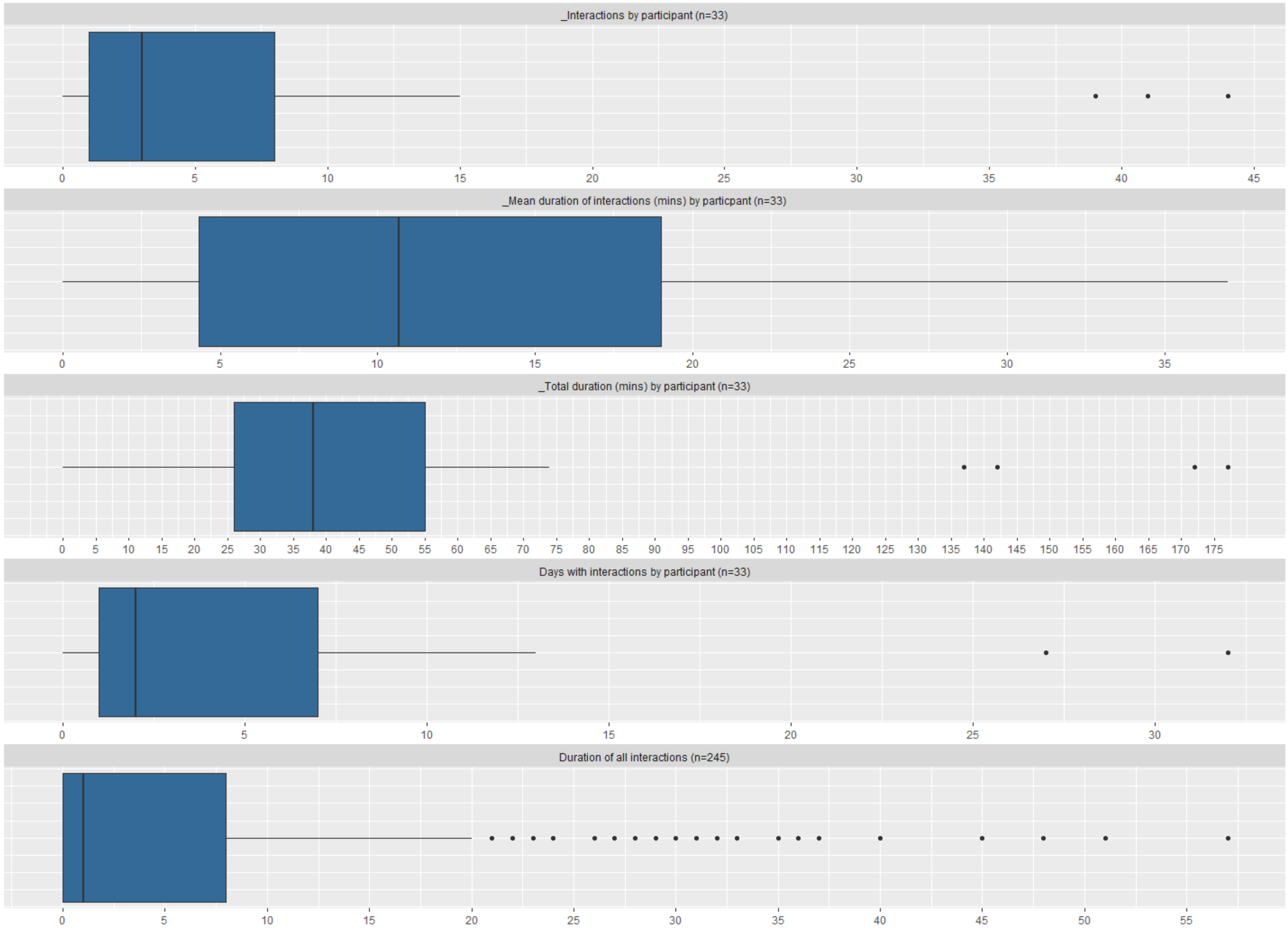
_Interactions by participant (n=33)
0 5 10 15 20 25 30 35 40 45
_Mean duration of interactions (mins) by particpant (n=33)
0 5 10 15 20 25 30 35
_Total duration (mins) by participant (n=33)
0 5 10 15 20 25 30 35 40 45 50 55 60 65 70 75 80 85 90 95 100 105 110 115 120 125 130 135 140 145 150 155 160 165 170 175
Days with interactions by participant (n=33)
0 5 10 15 20 25 30
Duration of all interactions (n=245)
0 5 10 15 20 25 30 35 40 45 50 55

Figure 6:Boxplots showing summaries of click analytics in CFHH

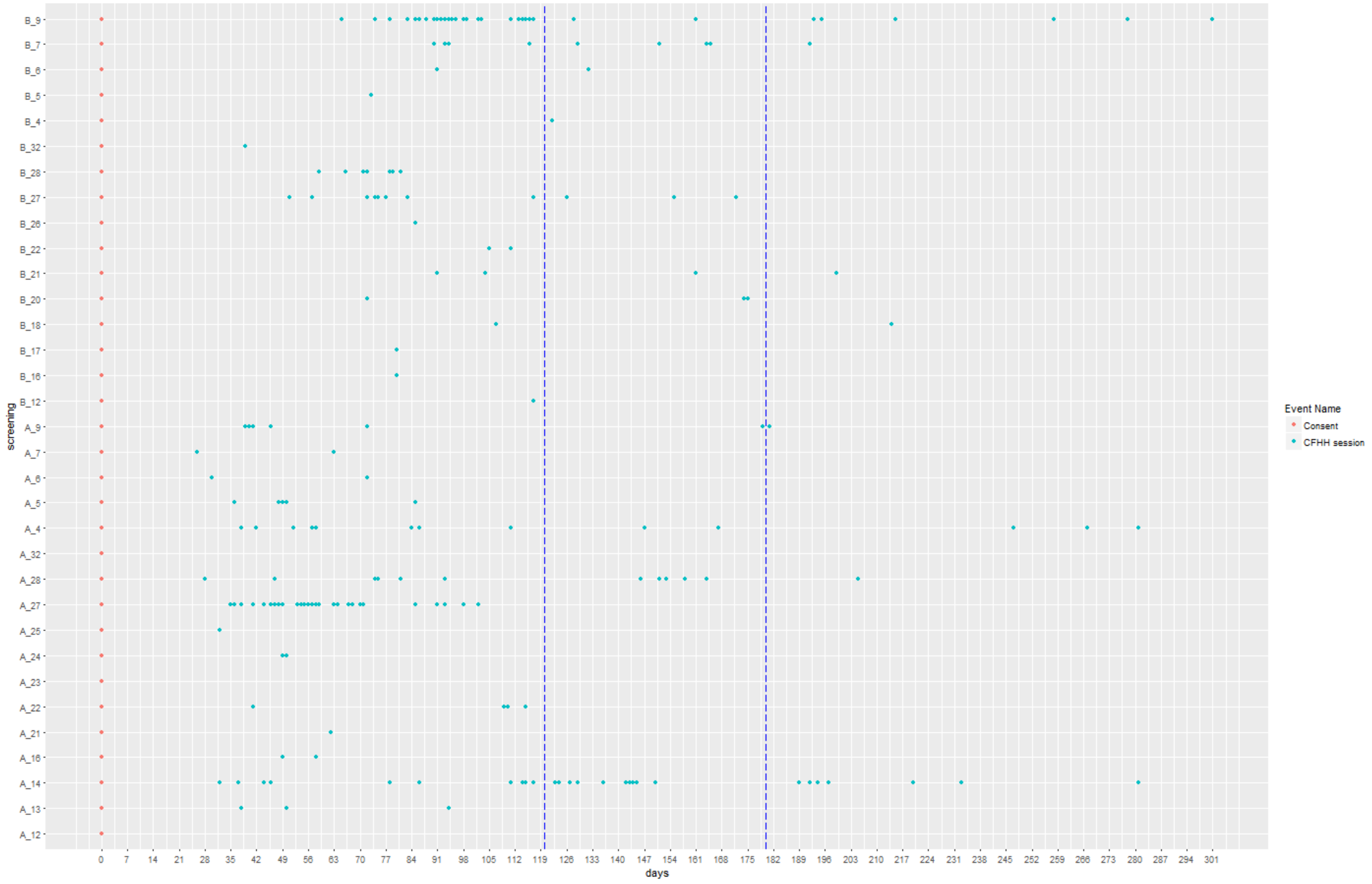

Figure 7:Timing in days of interactions with CFHH

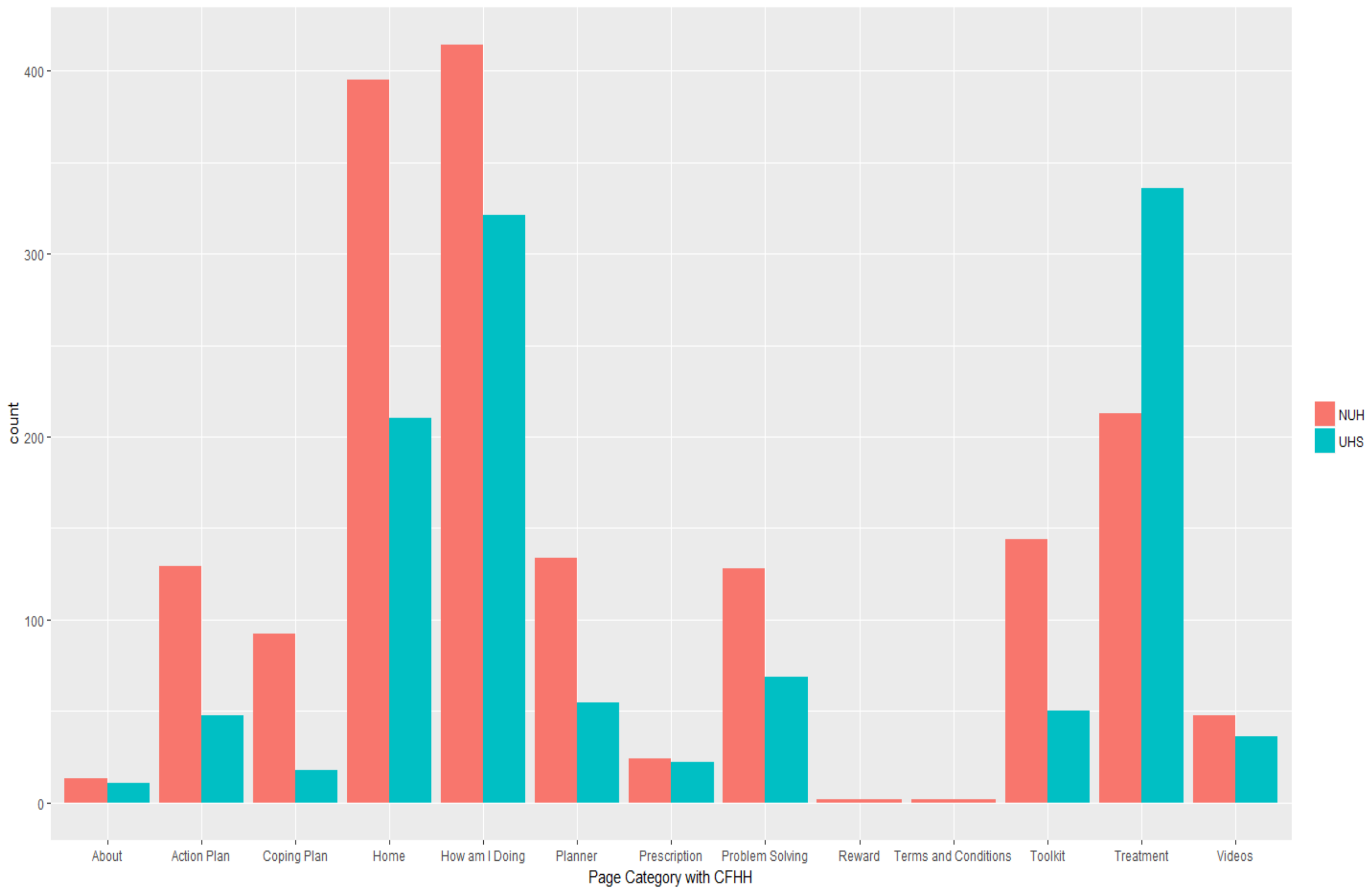

Figure 8:Frequency of clicks by CFHH categories

Table 19:Summary of clicks by page categories in CFHH

	Total (%) clicks	Participants (%) with at least one click	Sessions (%) with at least one click
**About**	24(0.8%)	13(39.4%)	20(8.2%)
**Action Plan**	177(6.1%)	28(84.8%)	53(21.6%)
**Coping Plan**	110(3.8%)	24(72.7%)	38(15.5%)
**Home**	605(20.8%)	30(90.9%)	244(99.6%)
**How am I Doing**	735(25.2%)	30(90.9%)	224(91.4%)
**Planner**	189(6.5%)	21(63.6%)	39(15.9%)
**Prescription**	46(1.6%)	22(66.7%)	42(17.1%)
**Problem Solving**	197(6.8%)	24(72.7%)	44(18%)
**Reward**	2(0.1%)	2(6.1%)	2(0.8%)
**Terms and Conditions**	2(0.1%)	2(6.1%)	2(0.8%)
**Toolkit**	194(6.7%)	24(72.7%)	66(26.9%)
**Treatment**	549(18.8%)	30(90.9%)	87(35.5%)
**Videos**	84(2.9%)	30(90.9%)	62(25.3%)

For peer review only

Intervention fidelity (Clinicians)

Table 21 shows the median number of intervention sessions per participant was 3 (IQR= 2,4) with a mean duration of 36.1 (SD=23.9) minutes.

Table 20:Summary of intervention sessions received by intervention participants during the study

Sessions per participant	
n	33
Mean (SD)	3(1.6)
Median (IQR)	3(2,4)
Min, Max	(0,6)
Total time by participant	
n	33
Mean (SD)	114.2(46.9)
Median (IQR)	100.5(90,125)
Min, Max	(40,249)
Time per session by participant	
n	33
Mean (SD)	37.3(14.2)
Median (IQR)	31.3(28.3,48)
Min, Max	(18,65)
Time per session	
n	99
Mean (SD)	36.1(23.9)
Median (IQR)	30(15,55)
Min, Max	(4,119)
Intervention session per participant	
n	33
Mean (SD)	0.9(0.3)
Median (IQR)	1(1,1)
Min, Max	(0,1)
Total Intervention session time per participant	
n	29
Mean (SD)	58.1(14.2)
Median (IQR)	60(48,60)
Min, Max	(35,90)

For peer review only

Review session per participant	
n	33
Mean (SD)	1(0.5)
Median (IQR)	1(1,1)
Min, Max	(0,2)
Total Review session time per participant	
n	29
Mean (SD)	43.2(30.6)
Median (IQR)	40(20,55)
Min, Max	(10,154)
Preparation session per participant	
n	33
Mean (SD)	0.7(0.9)
Median (IQR)	0(0,1)
Min, Max	(0,3)
Total Preparation session time per participant	
n	14
Mean (SD)	18.4(9.7)
Median (IQR)	15(15,30)
Min, Max	(4,35)
Ad hoc sessions per participant	
n	33
Mean (SD)	0.4(0.6)
Median (IQR)	0(0,1)
Min, Max	(0,2)
Total ad hoc session time per participant	
n	12
Mean (SD)	19.2(6.7)
Median (IQR)	15(15,25)
Min, Max	(15,30)

For peer review only

Clinic visits

Participants completed a median of 2 clinic visits. This was consistent across treatment arms. The number of clinic visits by participant is similar across treatment arms (Figure 9).

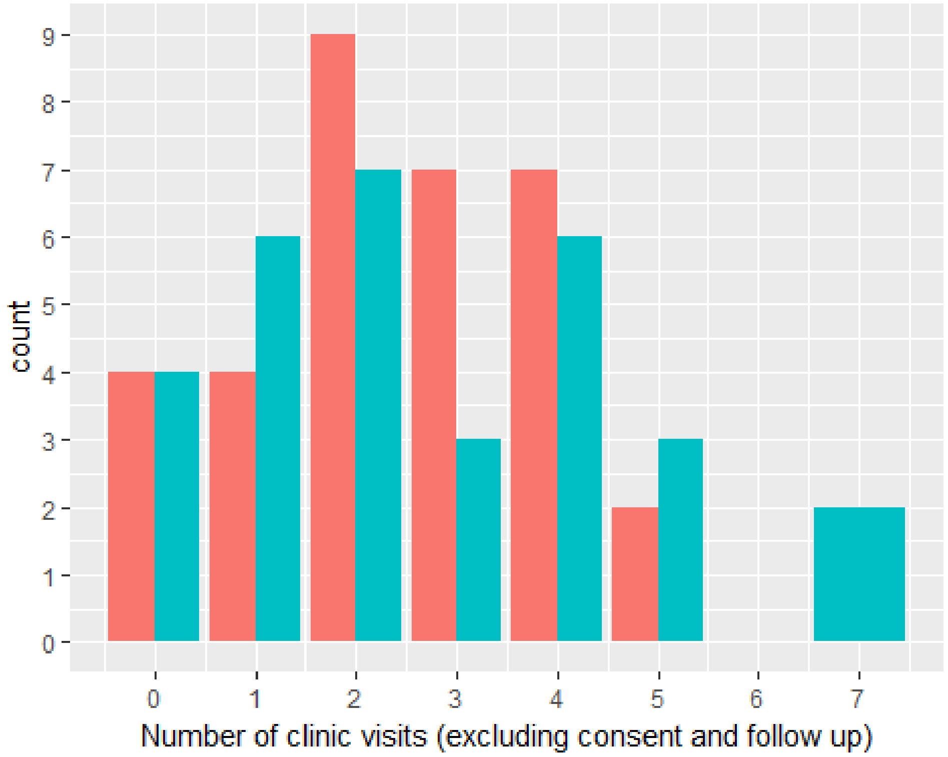

Figure 9:Barplot showing the number of participants for each number of clinic visits by treatment arm

Safety analysis

A total of 8 adverse events (AEs) occurred during the trial and 7 participants (10.9%) had a least one AE (Table 22). 5 of these were deemed to be Serious Adverse Events (SAEs). None of the SAEs were related to the intervention.

Table 21:Summary of adverse events recorded during the study

	Intervention n (%)	Control n (%)	Overall n (%)
All Adverse Events	5	3	8
Participants with at least 1 AE	4(12.1%)	3(9.7%)	7(10.9%)
Type of Adverse Event			
Chest pain or chest discomfort	1(25%)	0(0%)	1(14.3%)
Voice change or Alteration	0(0%)	0(0%)	1(14.3%)
Other	4(100%)	2(66.7%)	6(85.7%)

Table 22:Summary of serious adverse events recorded during the study

	Intervention n (%)	Control n (%)	Overall n (%)
All Serious Adverse events	3(9.1%)	2(6.5%)	5(7.8%)
Level of Seriousness			
Death	0(0%)	2(100%)	2(40%)
Hospitalisation	2(66.7%)	0(0%)	2(40%)
Persistent or significant disability/incapacity	1(33.3%)	0(0%)	1(20%)
Frequency			
Isolated	2(66.7%)	2(100%)	4(80%)
Continuous	1(33.3%)	0(0%)	1(20%)
Intensity			
Moderate	3(100%)	0(0%)	3(60%)
Severe	0(0%)	2(100%)	2(40%)
Outcome			
Recovered	1(33.3%)	0(0%)	1(20%)
Improved	2(66.7%)	0(0%)	2(40%)
Death	0(0%)	2(100%)	2(40%)
Expected SAE			
No	3(100%)	2(100%)	5(100%)
Related to Intervention			
No	3(100%)	2(100%)	5(100%)

For peer review only

For peer review only

Table 23:Description of serious adverse events recorded during the study (table has been redacted to maintain anonymity)

Participant ID	Description of event	Serious
xxx_15	Patient admitted on xx.xx.16 with acute exacerbation, developed type 2 respiratory failure. Despite maximal treatment of IV antibiotics, oxygen and NIV the patient continued to deteriorate and decision made to palliate. The patient died shortly afterwards.	Yes
xxx_14	Patient was having a kidney biopsy and had a bleed as a result, so had been kept in hospital on xxxxx ward at xxx city campus.	Yes
xxx_23	Patient admitted xx/xx/2016 with worsening disease and type 2 respiratory failure. Treated with non -invasive ventilation and intravenous antibiotics. deteriorated despite treatment and passed away xx/xx/2016	Yes
xxx_17	Rash reoccurred after re-trying oral antibiotic medication. Advised to stop again	No
xxx_17	Patient on holiday. Telephoned to report rash on both legs after starting new oral antibiotics. Advised to discontinue	No
xxx_20	Patient was admitted with influenza and CF. Exacerbation treated with iv antibiotics, discharged with home IV's. readmitted on the xx xxx with AKI (Acute Kidney Injury)<br>Assumed secondary to dehydration. Dornase stopped	Yes

Protocol non-compliances

In total, there were 9 protocol non compliances during the trial. 6 (67%) of these were follow up visits conducted outside of the calculated window (5 +/-1 month). 3 (33%) of these were participants ticking statements on the consent form rather than initialling. All of these protocol non compliances were assessed as minor non-compliances.

For peer review only

Summary of missing data

Exacerbation data was collected for 6 months in 60/64 participants (94%). Adherence was collected for at least 6 months for 58/64 participants (90%).

The number of missing scores for questionnaires completed at baseline and 5 month follow up was very low (Table 25). Completion rate was 100% for the majority of baseline questionnaires and at least 89% for 5 month questionnaires. Missing scores were due to drop out(described in section 2.1). Such high completion rates are reassuring for the main trial.

Table 24:Summary of missing scores and items within questionnaires

	Time	Total	%	Intervention Median (min,max)	Control Median (min,max)	Overall Median (min,max)
**EQ5D-5L**	Baseline	64	100 %	5 (5 , 5)	5 (5 , 5)	5 (5 , 5)
**5 items**	5 (+/-1) months	58	90.6 %	5 (0 , 5)	5 (0 , 5)	5 (0 , 5)
**PAM-13**	Baseline	64	100 %	13 (13 , 13)	13 (13 , 13)	13 (13 , 13)
**13 item**	5 (+/-1) months	59	92.2 %	13 (0 , 13)	13 (0 , 13)	13 (0 , 13)
**CHAOS**	Baseline	64	100 %	4 (4 , 4)	4 (4 , 4)	4 (4 , 4)
**4 items**	5 (+/-1) months	59	92.2 %	4 (0 , 4)	4 (0 , 4)	4 (0 , 4)
**MAD-3**	Baseline	62	96.9 %	3 (1 , 3)	3 (0 , 3)	3 (0 , 3)
**3 items**	5 (+/-1) months	57	89.1 %	3 (0 , 3)	3 (0 , 3)	3 (0 , 3)
**SRBAI**	Baseline	63	98.4 %	4 (0 , 4)	4 (4 , 4)	4 (0 , 4)
**4 items**	5 (+/-1) months	59	92.2 %	4 (0 , 4)	4 (0 , 4)	4 (0 , 4)
**GAD-7**	Baseline	64	100 %	7 (7 , 7)	7 (7 , 7)	7 (7 , 7)
**7 items**	5 (+/-1) months	59	92.2 %	7 (0 , 7)	7 (0 , 7)	7 (0 , 7)
**PHQ-8**	Baseline	64	100 %	8 (8 , 8)	8 (8 , 8)	8 (8 , 8)
**8 items**	5 (+/-1) months	59	92.2 %	8 (0 , 8)	8 (0 , 8)	8 (0 , 8)

For peer review only

Recommendations for Main Trial/ Points for discussion

- For the primary analysis in the main trial, we would recommend the use of the offset adjusted model as this will allow the use of more data and allows the inclusion of potentially important participants over a greater amount of time. For example, our original model excluded participants who died, however doing so means we have lost key information.

- This is a pilot study, not powered to detect an effect
- The nature of the data means that small changes appear to influence the result greatly

For peer review only

Appendix

Description of the patient reported outcomes

Name	Score range	Description	Interpretation of score
**EQ-5D-5L**	-0.224-1	Measure of health status	A score of zero means death, 1 is full health, negative score is a state worse than death
**PAM-13**	0-100	Measures patient activation e.g. ability and willingness to manage their health. 13 items with scoring spreadsheet	0= low patient activation<br>100= high patient activation
**CHAOS-6**	0-24	Measures confusion, hubbub and order. 6 item questionnaire	0= low level of chaos<br>24= high level of chaos
**SRBAI**	0-28	Measure of habit and automaticity 4 item, 7 point likert scale	0= low level of automaticity<br>28= high level of automaticity
**CFQ-R**	0-100	8 domains each score 0-100. The domains are:<br>Physical, Emotion, Social, Eating, Body, Treatment Burden, Respiratory, Digestion	0= low<br>100= high
**PHQ-8**	0-24	Measure of depression. 8 item questionnaire, 0-3 for each item	0= No or minimal depression<br>24= Severe depression
**GAD-7**	0-21	Measure of anxiety. 7 item questionnaire	0= No anxiety<br>21= Severe anxiety
**COM-BBQ**			
Specific Necessities	2-5	Measure of perceived personal need for medication	Direction of effect would be an increase in score
Specific Concerns	1-3	Measure of perceived concerns about the negative effects of the medicine they are taking	Direction of effect would be a decrease in score
**MAD-3**	3-15	Specifically made 3 item questionnaire to measure perceived medication adherence	3= low<br>15= high

For peer review only

Additional File 07. Changes to intervention procedures

Change Number	Problem type	Problem Identified	Solutions implemented in full-scale trial (hashed numbers - # - refer to logic model constructs)	Timing of change implementation
**CFHealthHub IT component**				
1	Real World and Trial	Interventionists having difficulty identifying videos (#22) appropriate for a patient's needs or interests.	Descriptions were provided with each video. The PPI group agreed with this change and assisted with writing descriptions for each video.	During the feasibility study
2	Real World and Trial	Adherence charts (#14, #20) were showing >100% adherence. This appeared to be more common in patients with alternating regimes, or taking medications *pro re nata* (PRN, meaning 'as needed').	Prescription flow amended with the addition of PRN or alternating regime alerts, which will assist the data management team in highlighting any data discrepancies.	Post-feasibility study
3	Real World and Trial	Clinician functionality (amending prescriptions/ treatment targets (#3, #23) inaccessible through participant view (used in intervention sessions).	Participant view functionality implemented to facilitate intervention sessions. Clinicians are now able to run intervention sessions using CFHH through participant view but easily switch to clinician view to change prescriptions and to set goals.	Post-feasibility study

For peer review only - http://bmjopen.bmj.com/site/about/guidelines.xhtml

4	Real World and Trial	The lead psychologist identified the need to determine which participants were receiving push notifications as this relates to dose and rewards for adherence.	The option to export data about number of push notifications sent to participants from the app (#16).	Post-feasibility study
5	Real World and Trial	Originally the normative adherence was used to come up with the percentage adherence. It was identified this did not always match what participants were actually prescribed and this made the graphs difficult to interpret. The capping of the weekly graph at 100% also made interpretation difficult.	To improve interpretability of adherence data (#14), percentages are now calculated against the actual treatments prescribed and graphs are not capped at 100% to aid any interpretation of graphs and trouble shooting.	Post-feasibility study
**Other IT infrastructure**				
6	Real World and Trial	Flatlines at the beginning of some participant adherence run charts were identified to relate to the date registered at the time the nebuliser (#4) is paired with the Qualcomm Hub (#5). Flatlines at the end of the feasibility study were also observed (#14, #35).	To achieve quality assurance of adherence data (#4, #5, #14, #35), hardware is now paired at the factory. The full-scale trial has been monitoring for, and has not found, such instances. Flatlines at the end of run charts established as genuine through triangulation with self-report quantitative and qualitative data.	Post-feasibility study

For peer review only - http://bmjopen.bmj.com/site/about/guidelines.xhtml

**Interventionist training and manual**				
7	Real World and Trial	Training packages were initially developed for physiotherapists. This led to interventionist recruitment problems.	The job specification and training was redeveloped to suit non-physiotherapists (#9, #12), to enable any member of the MDT to be trained up to deliver the intervention. A suitably qualified individual such as a postgraduate psychologist could be supported by the MDT to deliver the intervention.	Post-feasibility study
8	Real World and Trial	The interventionist job specification did not reflect the flexibility needed to carry out the interventionist role- e.g. flexibility in working patterns, skills in motivational interviewing and extensive travel.	The research team, with input from the interventionists, revised the job specification for the interventionist role based on experience of delivering the intervention in the pilot in order to better manage expectations of the role **(#12).**	Post-feasibility study
9	Real World and Trial	Pilot study interventionists felt that training was good but could be helped by introducing case studies with real world data, in CFHealthHub.	Realistic case studies with data to support interventionist training / role plays for using website were developed to provide training more applicable to real CF patients (#9). This model is generally used in a healthcare training setting.	Post-feasibility study
10	Real World and Trial	Sporadic training over six weeks, whilst also conducting research procedures was	Training was condensed into an intensive course over ten days, focusing solely on intervention delivery (#9).	Post-feasibility study

For peer review only - http://bmjopen.bmj.com/site/about/guidelines.xhtml

		overwhelming for interventionists.		
11	Real World and Trial	Assessment of intervention fidelity identified that some of the active ingredients of the intervention were absent e.g. negotiating goals and letting participants take ownership of choices.	The recruitment and training process was modified to incorporate role play at the interview; explaining fidelity assessment criteria during training and also on-going assessment to ensure that any issues are identified quickly (#9).	Post-feasibility study
12	Real World and Trial	The focus of interventionists during intervention delivery was not always on the aspects that evidence would indicate are the most active ingredients for example goal setting, action planning and coping planning.	Emphasis was placed on the main 'active ingredients' in the manual and in training (#8, #9).	Post-feasibility study
13	Real World and Trial	During the course of the trial, it became apparent that participants were not being followed up and engaged in a manner to allow them to build a habit.	Focus on habit formation / revised logic model will be implemented by a 6-8 week period of habit formation sessions (#8).	Post-feasibility study
14	Real World and Trial	It was identified that after some participants last review visit, their adherence to treatment dropped.	For the full RCT, intervention visits are now triggered if the participant is having an exacerbation/IV, has a drop of 20% or more adherence in the last 4 weeks and if the participant requests additional support.	Post-feasibility study

For peer review only - http://bmjopen.bmj.com/site/about/guidelines.xhtml

			These will be termed 'intervention triggers' (#8).	

For peer review only

For peer review only - http://bmjopen.bmj.com/site/about/guidelines.xhtml

Good Reporting of A Mixed Methods Study (GRAMMS).

O'Cathain A, Murphy E, Nicholl J. The quality of mixed methods studies in health services research. *J Health Serv Res Policy* 2008;13:92–8. doi:10.1258/jhsrp.2007.007074

Added to the EQUATOR Network database 26/09/2013.

(1) Describe the justification for using a mixed methods approach to the research question.

p5; lines 93-95.

(2) Describe the design in terms of the purpose, priority and sequence of methods

p11; line 240: we used a modified triangulation protocol; the study is described as nested, indicating, that the methods were used concurrently (p9; line 192).

(3) Describe each method in terms of sampling, data collection and analysis

Pages 9-12; 195-273.

(4) Describe where integration has occurred, how it has occurred and who has participated in it

Pages 11-12; Lines 240-273.

(5) Describe any limitation of one method associated with the present of the other method

Page 25; Lines 576-582.

(6) Describe any insights gained from mixing or integrating methods

p5; lines 93-95. p25; lines 573-611.

For peer review only
